# The saprotrophic *Pleurotus ostreatus* species complex: late Eocene origin in East Asia, multiple dispersal, and complex speciation

**DOI:** 10.1186/s43008-020-00031-1

**Published:** 2020-06-08

**Authors:** Jing Li, Li-Hong Han, Xiao-Bin Liu, Zhi-Wei Zhao, Zhu L. Yang

**Affiliations:** 1 CAS Key Laboratory for Plant Diversity and Biogeography of East Asia, Kunming Institute of Botany, Chinese Academy of Science, Kunming, 650201 Yunnan China; 2 Yunnan Key Laboratory for Fungal Diversity and Green Development, Kunming, 650201 Yunnan China; 3 State Key Laboratory of Conservation and Utilization for Bioresources in Yunnan, Yunnan University, Kunming, 650091 Yunnan China; 4 College of Biological Resource and Food Engineering, Qujing Normal University, Qujing, 655011 Yunnan China

**Keywords:** Illumina MiSeq, Molecular phylogeny, Species recognition, East Asian origin, Diversification, Saprotrophic mushrooms

## Abstract

The *Pleurotus ostreatus* species complex is saprotrophic and of significant economic and ecological importance. However, species delimitation has long been problematic because of phenotypic plasticity and morphological stasis. In addition, the evolutionary history is poorly understood due to limited sampling and insufficient gene fragments employed for phylogenetic analyses. Comprehensive sampling from Asia, Europe, North and South America and Africa was used to run phylogenetic analyses of the *P. ostreatus* species complex based on 40 nuclear single-copy orthologous genes using maximum likelihood and Bayesian inference analyses. Here, we present a robust phylogeny of the *P. ostreatus* species complex, fully resolved from the deepest nodes to species level. The *P. ostreatus* species complex was strongly supported as monophyletic, and 20 phylogenetic species were recognized, with seven putatively new species. Data from our molecular clock analyses suggested that divergence of the genus *Pleurotus* probably occurred in the late Jurassic, while the most recent common ancestor of the *P. ostreatus* species complex diversified about 39 Ma in East Asia. Species of the *P. ostreatus* complex might migrate from the East Asia into North America across the North Atlantic Land Bridge or the Bering Land Bridge at different times during the late Oligocene, late Miocene and late Pliocene, and then diversified in the Old and New Worlds simultaneously through multiple dispersal and vicariance events. The dispersal from East Asia to South America in the middle Oligocene was probably achieved by a long-distance dispersal event. Intensification of aridity and climate cooling events in the late Miocene and Quaternary glacial cycling probably had a significant influence on diversification patterns of the complex. The disjunctions among East Asia, Europe, North America and Africa within Clade IIc are hypothesized to be a result of allopatric speciation. Substrate transitions to Apiaceae probably occurred no earlier than 6 Ma. Biogeographic analyses suggested that the global cooling of the late Eocene, intensification of aridity caused by rapid uplift of the QTP and retreat of the Tethys Sea in the late Miocene, climate cooling events in Quaternary glacial cycling, and substrate transitions have contributed jointly to diversification of the species complex.

## INTRODUCTION

The origin and evolution of species is one of the most important issues in biological research (Seehausen et al. 2014; Wiens 2004), and biogeographic studies aim to reconstruct the origin, speciation and distribution patterns of organisms (AI-Tanlimi et al. 2003; Schluter 2000). To date, there have been a number of biogeographical-evolutionary studies in higher plants and animals (Carranza and Arnold 2004; Conti et al. 2002; Dobigny et al. 2005; Sanmartín and Ronquist 2004; Yi et al. 2015). Mushrooms, or macrofungi, represent one of the most diverse groups of organisms in the world, but have been the subject only very limited biogeographical analysis, perhaps because of limited morphological species recognition (Taylor et al. 2006), incomplete sampling in many regions of the world (Schmit & Mueller 2007), the scanty fungal fossil record (Taylor and Berbee 2006), and the view that mushrooms are able to overcome geographic barriers by virtue of their airborne spores (Hibbett 2001; Vilgalys and Sun 1994a). It’s worth noting that the limited numbers of molecular phylogenetic studies involving mushrooms have gained new insights into the historical dynamics of macrofungi and show that macrofungi provide interesting subjects for biogeographic studies (Coetzee et al. 2000; James et al. 2001; Lumbsch et al. 2008; Nagy et al. 2011).

Molecular-based biogeographic studies of macrofungi have mainly focused on ectomycorrhizal fungi (EMF) with wide geographic distributions (Cai et al. 2014; Feng et al. 2012; Han et al. 2018; Hibbett and Matheny 2009; Hosaka et al. 2008; Sánchez-Ramríez et al. 2015; Truong et al. 2017). Saprotrophic fungi (SPF) have a worldwide distribution and also play critical roles in ecosystem function, soil health, and human and animal nutrition (Austin et al. 2004; McGonigle 1995; Rayner and Boddy 1988; Wolfe et al. 2012). The biogeographic structures of various saprotrophic agarics in the Northern Hemisphere or with broad geographical distribution have recently started to be uncovered (Hibbett et al. 1998; Methven et al. 2000). However, few of the above studies specifically included SPF from both the Southern and Northern Hemispheres (Carlsen et al. 2011; James et al. 1999) and even fewer have attempted molecular clock dating and diversification methods to investigate the date of origin and historical diversification (Moncalvo and Buchanan 2008; Qin et al. 2018; Skrede et al. 2011). In general, the spatio-temporal dynamics and mechanisms of distribution and diversification of global saprotrophic fungal species are poorly understood.

The *Pleurotus ostreatus* species complex harbors most of the species of *Pleurotus* sect. *Pleurotus* as defined by Singer (1986), all of which possess a monomitic hyphal system. Specifically, *P. ostreatus* (Jacq.) P. Kumm., *P. pulmonarius* (Fr.) Quél. and *P. placentodes* (Berk.) Sacc. and their allies belong to the complex. Because of its nutritional and medical properties, as well as diverse biotechnological applications, the *P. ostreatus* species complex is one of the three most actively cultivated mushroom species in the world (Baker et al. 2018; Cardwell et al. 2018; Golak-Siwulska et al. 2018). As commonly found saprotrophic mushrooms, species within the *P. ostreatus* complex constitute essential components in forest and grassland ecosystems because of their roles as decomposers of a large array of lignocellulosic substrates, such as deciduous trees (Kay and Vilgalys 1992; Petersen and Krisai-Greilhuber 1996), coniferous trees (Petersen and Hughes 1997; Vilgalys et al. 1993), as well as herbs in the Apiaceae family (Zervakis et al. 2014). These fungi have also adapted to a wide variety of environments, ranging from mesic to semiarid conditions and temperate forest to subalpine.

Because this is an economically and ecologically important group, species diversity in the *P. ostreatus* species complex has been estimated using traditional morphological criteria and mating incompatibility tests (Hilber 1982; Petersen and Hughes 1993; Singer 1986; Vilgalys et al. 1993; Zervakis and Balis 1996; Zervakis et al. 2014). Based on morphological features and mating compatibility, geographic distribution patterns and host ranges of the *P. ostreatus* species complex in Europe and North America have been elucidated (Vilgalys et al. 1993). An interesting study on the biogeography of the whole genus *Pleurotus* has suggested an ancient origin for some intersterile groups with worldwide distributions, while other intersterile groups in the Northern Hemisphere underwent more recent radiation and allopatric speciation (Vilgalys and Sun 1994b). Although a series of taxonomic and phylogenetic studies based on limited molecular markers have been published recently (Avin et al. 2014; Gao et al. 2008a; He et al. 2016; Kawai et al. 2008; Li et al. 2017; Thorn et al. 2000; Vilgalys et al. 1993; Zhao et al. 2016b; Zheng et al. 2006), a deep understanding of the diversity within this group is still lacking.

The advent of next-generation sequencing (NGS) has changed history diversification and molecular phylogenetic studies (Binder et al. 2013; Egan et al. 2012; McCormack et al. 2011; Yu et al. 2017). Nuclear single-copy orthologous genes have been demonstrated to be promising molecular markers for phylogenetic and evolutionary inferences (Sato et al. 2017; Sato and Toju 2019). We selected the *P. ostreatus* species complex to avoid incomplete taxon sampling, which often occurs during the analysis of large clades and could introduce serious biases into the estimation of the diversification rates (Brock et al. 2011; Rabosky 2010; Sato et al. 2017).

Deeper insight into phylogeny, species diversity, and diversification of the *P. ostreatus* species complex should be achieved by the analyses of multiple nuclear single-copy orthologous sequences in combination with appropriate models of sequence evolution. The main objectives of this study are: (1) to reconstruct a well-resolved molecular phylogeny with comprehensive taxon sampling for the *P. ostreatus* species complex, based on multiple single-copy nuclear markers, using next generation sequencing; (2) to determine times of molecular divergence events within the *P. ostreatus* species complex and its major lineages, using phylogenetic analyses at the species complex and species levels; and (3) to reconstruct the geographic origins, the history of biogeographic diversification and the potential migration routes of the *P. ostreatus* species complex, based on paleontological evidence, palaeoclimatic records and estimated divergence times for the lineages within the complex.

## MATERIALS AND METHODS

### Screening of taxa used in the phylogenetic analysis for a large dataset

A total of 51 specimens of *Pleurotus* and *Hohenbuehelia* Schulzer (as an outgroup) were included in this study (Table 1). Specific information on voucher specimens and strains, including GenBank accession numbers, geographical locations, and substrates is in Table 1. Species belonging to *Pleurotus* have been reported from the temperate areas of East and West Asia, North and South America, Europe, Africa and the tropical areas of Southeast Asia (Albertó et al. 2002; Petersen and Hughes 1997; Petersen and Krisai-Greilhuber 1999; Vilgalys et al. 1993; Vilgalys and Sun 1994b; Zervakis and Balis 1996; Zervakis et al. 2004, 2014), whereas those belonging to *Hohenbuehelia* has been reported as the allied group of *Pleurotus* (Gao et al. 2008b; Li and Yao 2004; Li et al. 2017; Menolli Jr. et al. 2014; Singer 1986; Thorn et al. 2000). Most mushrooms analyzed here were chosen to cover as much of the known distribution range of the *P. ostreatus* species complex in the world as possible. Voucher specimens and strains for these samples are kept in the Herbarium of Cryptogams, Kunming Institute of Botany of the Chinese Academy of Sciences (HKAS), China Center for Mushroom Spawn Standards and Control (CCMSSC), the Herbarium of the Laboratory of the General and Agricultural University of Athens (ACAM), and the Westerdijk Fungal Biodiversity Institute (CBS).

**Table 1:** List of specimens used to infer the phylogeny of *Pleurotus ostreatus* species complex

Taxon	Specimen	Locality	Substrate	GenBank accession numbers	
				ITS	*RPB2*
*Pleurotus abalonus*	HKAS81197^a^	Yunnan, China	*Quercus* sp.	MN546043	MT138447
*P. abieticola*	HKAS89521^a^	Sichuan, China	*Picea* sp.	MN546039	–
	HKAS89541	Gansu, China	*Picea* sp.	MN546040	MT138444
*P. albidus*	CBS119924^a^	Argentina	*Quercus palustris*	MN546041	MT138443
*P. citrinopileatus*	HKAS93365	Jilin, China	hardwood	MN546044	MT138448
	HKAS94429	Jilin, China	*Populus* sp.	–	MT138449
*P. cystidiosus*	HKAS106470	Hunan, China	–	MN546042	MT138446
	HKAS97644	Yunnan, China	–	–	MT138445
*P. djamor*	HKAS94069	Sri Lanka	–	KX061789	–
	HKAS94070	Sri Lanka	–	MN546045	–
*P. dryinus*	HKAS94448	Finland	–	MN546046	–
*P. eryngii* var. *elaeoselini*	PN13^a^	Italy	*Elaeoselinum asclepium*	KF743831	MT138430
*P. eryngii*. var. *eryngii*	CCMSSC00692	Spain	*Eryngium* sp.	KX836357	KX870362
	CCMSSC00467^a^	Italy	*Eryngium* sp.	–	MT138431
*P. eryngii* var. *ferulae*	CCMSSC04223^a^	Italy	*Ferula communis*	KU612927	MH374115
	CCMSSC00647	Netherlands	*Ferula communis*	KU612924	MH473116
	CCMSSC03175^a^	Xinjiang, China	*Ferula communis*	KU612920	MH374117
	CCMSSC03217	Xinjiang, China	*Ferula communis*	KU612916	–
*P. ferulaginis*	HIK133^a^	Italy	*Ferulago campestris*	KF743826	–
*P. fossulatus*	HIK127^a^	Armenia	*Prangos ferulacea*	HM998828	–
*P. nebrodensis*	CCMSSC04220^a^	Netherlands	*Prangos ferulacea*	KU612942	–
	CCMSSC00646	Netherlands	*Prangos ferulacea*	KU612943	KX870371
*P. opuntiae*	CBS102543	Mexico	–	MN546047	MT138450
*P. ostreatus*	HKAS84903^a^	Germany	*Fagus* sp.	KP867913	KP867874
	CCMSSC00338	Germany	*Fagus sylvatica*	KX836103	KX870204
	HKAS93337	France	*Sabina chinensis*	–	MT138429
*P. placentodes*	HKAS94410^a^	Tibet, China	–	KX836665	–
	HKAS57781	Yunnan, China	*Picea* sp.	KR827694	KR827698
	YAASM3153	Yunnan, China	–	–	KX870444
	YAASM2083	Yunnan, China	–	–	KX870443
*P. populinus*	CBS109622^a^	USA	–	MN546031	MT138442
	CBS666.85	Canada	*Populus* sp.	MN546032	MT138441
*P. pulmonarius*	HKAS56524^a^	Germany	*Fagus sylvatica*	MN546036	–
	CCMSSC00500	Greece	–	KU612947	MT138435
*P. tuoliensis*	CCMSSC03105^a^	Xinjiang, China	*Ferula* sp.	KU612906	MH374113
	CCMSSC03212	Xinjiang, China	*Ferula* sp.	KU612908	MH374112
*Pleurotus* sp. 1	HKAS106471(Kejia1)	Hebei, China	–	KX836252	KX870271
	HKAS106472(Pinggu2026) ^a^	Hebei, China	–	KX836264	KX870223
*Pleurotus* sp. 2	CCMSSC00324^a^	Japan	–	KX836142	KX870232
	HKAS106473(YAASM2072) ^a^	Yunnan, China	*Betula* sp.	KX836134	KX870243
*Pleurotus* sp. 3	HKAS93372	USA	*Quercus* sp.	MN546030	MT138434
	HKAS106474(ZP742) ^a^	Canada	–	KX836193	KX870345
	CBS195.92	USA	–	MN546028	MT138433
*Pleurotus* sp. 4	CCMSSC00346^a^	Jilin, China	–	MN546029	MT138432
*Pleurotus* sp. 5	HKAS92312^a^	Hubei, China	Fagaceae	KX836303	KX870433
	HKAS91310	Jilin, China	Fagaceae	KX836300	KX870384
*Pleurotus* sp. 6	HKAS94322^a^	Canada	–	MN546037	MT138439
	HKAS94336	Canada	–	MN546038	MT138440
*Pleurotus* sp. 7	HKAS94249^a^	Benin	*Monotes kerstingii*	MN546033	MT138437
	HKAS94228	Benin	*Nauclea* sp.	MN546034	MT138436
	HKAS93854	Benin	*Monotes kerstingii*	MN546035	MT138438
*H. portegna*	HKAS74040	Yunnan, China	–	KY426797	–
*H. unguicularis*	HKAS90443	Yunnan, China	–	MN546048	–

Samples used for diversification analyses and ancestral state reconstruction are labeled with ^a^

Total DNA was isolated from the mycelia or specimen using the cetyltrimethyl ammonium bromide (CTAB) method (Doyle and Doyle 1987). The quality of the DNA was evaluated on agarose gels, and the concentrations of DNA were determined using an ND-2000 spectrophotometer (NanoDrop Technologies, Wilmington, DE, USA). The final DNA concentrations were adjusted to 25 ng/L and they were stored at − 20 °C until use.

### Selection of nuclear single-copy orthologous genes

Amino acid sequences of single-copy genes from *Phanerochaete chrysosporium* Burds. (Phanerochaetaceae/Polyporales/Agaricomycetes) were downloaded from the FUNYBASE database of fungal nuclear single-copy orthologous gene sequences, and the PCR primers that amplify single-copy genes of Agaricomycetes were designed by Sato et al. (2017). To screen for PCR amplification rate, 96 markers from their study with the expected product size ranging from 280 to 450 bp were initially tested and screened against ten diverse specimens taken from different species within the *P. ostreatus* species complex recognized by previous studies based on morphological characteristics and molecular analyses (Bao et al. 2004; Hilber 1982; Li et al. 2017; Liu et al. 2015, 2016; Singer 1986; Zervakis et al. 2014; Zhao et al. 2016a, 2016b). The amplification reactions were performed in an ABI 2720 Thermal Cycler or a SimpliAmp Thermal Cycler (Applied Biosystems, Foster City, CA, USA) with the following protocol: one cycle at 94 °C for 3 min; followed by 35 cycles at 94 °C for 30 s, an annealing step at 50, 52 or 54 °C for 30 s, 72 °C for 1 min; and a final extension step of 72 °C for 7 min. During marker screening, priority was given to single-copy markers with PCR amplification rate of greater than 80%. The primer sets for the 50 markers with greatest PCR amplification rates and single strong bands were modified for further experiments (Additional file 1).

### Sequencing of nuclear single-copy orthologous genes

For next-generation sequencing, specimens were selected from each of the provisionally putative species of *Pleurotus* and *Hohenbuehelia*. A two-step PCR was performed for these representative samples followed by Sato et al. (2017). The PCR products were quantified using a Qubit 4 fluorometer (Invitrogen Corporation, California, USA), following which the concentrations of the PCR products were normalized. The adjusted PCR products were pooled, following which amplicons of length 450–600 bp were excised and extracted using a Zymo DNA Clean & Concentrator-5 and Zymoclean Gel DNA Recovery Kit (Zymo Research Corporation, Irvine City, CA, USA). The amplicon library was sequenced using paired-end sequencing on a MiSeq platform using a MiSeq v.3 Reagent Kit according to the manufacturer’s instructions.

### Bioinformatic analyses

BCL2FASTQ v.1.8.4 (Illumina, San Diego, CA, USA) was used to convert the base calls into forward, index1, index2 and reverse FASTQ files. In order to obtain more accurate and reliable results in subsequent bioinformatics analysis, the raw data was pre-processed using an in-house procedure as following: 1) Sequence reads not having an average quality of 20 over a 30 bps sliding window based on the phred algorithm were truncated, and trimmed reads having less than 75% of their original length, as well as their paired reads, were removed; 2) Removal of reads contaminated by adapters; 3) Removal of reads with ambiguous bases (N bases), and their paired reads; 4) Removal of reads with low complexity. Paired-end reads were generated using the Illumina MiSeq platform, and the reads with sequencing adapters, N bases, poly-bases, and low quality bases were filtered out using default parameters. If two paired-end reads overlapped with 1) a minimum overlap of 15 bp and 2) a mismatching ratio in the overlapped region <= 0.1, the consensus sequence was generated by FLASH v1.2.11 (Magoč and Salzberg 2011). Paired-end reads without overlaps were removed.

### Construction of molecular phylogenetic trees and recognition of phylogenetic species

Before multilocus molecular analysis, we distinguished the lineages of *Pleurotus*, which probably represent reproductively isolated species. To do this, nucleotide sequences of the internal transcribed spacer (ITS) region and the gene for RNA polymerase II second largest subunit (*RPB2*) newly generated and from GenBank were listed in Table 1 and Additional file 2. ITS and *RPB2* sequences were aligned using the multiple sequence alignment algorithm FFT-NS-2 implemented in MAFFT v.7.245 (Katoh et al. 2002), respectively. The resulting alignment of ITS and *RPB2* were subjected to molecular phylogenetic inference based on the maximum likelihood (ML) method using RAxML v.8.1.5 (Stamatakis 2006). The selected substitution models for the four partitions were as follows: GTR + G for ITS, SYM + I + G for *RPB2*. Bootstrap support values were calculated from 1000 standard bootstrap replications, as implemented in RAxML. According to recognition terminal clades of ITS phylogenetic tree, *RPB2* sequence variations among specimens within each of several known species were compared and a cutoff value was then proposed to define species limits. Specifically, in our study, five species, *Pleurotus abieticola* R.H. Petersen & K.W. Hughes, *P. citrinopileatus* Singer, *P. eryngii* var. *ferulae* (Lanzi) Sacc., *P. eryngii* (DC.) Quél., and *P. placentodes*, were used to identify the range of intra-specific variation and establish a conservative cutoff value for phylogenetic species identification, with the highest intraspecific variation of these five species chosen as our cutoff value. These species were selected here because they have been well studied both morphologically and phylogenetically by other researchers (Li et al. 2017; Zhao et al. 2016b) or by our research group (unpublished data). Based on the phylogenetic tree generated from the *RPB2* data, each terminal branch with a high statistical support was treated provisionally as one species. Subsequently, intra- and inter-specific variations of these provisionally adopted species were then calculated in MEGA 5 (Tamura et al. 2011). Any provisionally adopted species was accepted as a valid species if it showed greater divergence from its closest sister taxa than the cutoff value. In contrast, the taxa with divergence lower than the cutoff value were considered as belonging to the same species.

The tags obtained from the next-generation sequencing from each gene were separately aligned with nucleotide sequences of the same gene from *Strobilomyces* aff. *confusu*s (GenBank accession numbers: LC082671–LC084587) using MAFFT v.7.245 (Katoh et al. 2002), by which non-orthologous sequences (sequences obtained from non-specific amplification) were removed. Exon-intron boundaries were also identified with the aligned orthologous sequences. The aligned sequences of introns were separately cleaned using GBLOCKS v.0.91b (Castresana 2000), with options ‘Allow smaller final blocks’ and ‘Allow gap positions within the final blocks’. The nuclear single-copy orthologous genes whose sequences were not detected in more than half of the total samples were removed to minimize potential biases caused by missing data or alignment gaps. The remaining 40 nuclear single-copy orthologous genes were subjected to subsequent analyses.

Phylogenetic inference based on the ML method was performed using RAxML as mentioned. The optimal substitution model was determined with the Akaike Information Criterion as implemented in PAUP* 4.0b10 (Swofford 2003) and MrModeltest v2.3 (Nylander 2004) (Additional file 3). Phylogenetic analysis was also performed with Bayesian inference using MRBAYES v.3.2.1 (Ronquist et al. 2012). BI analysis consisting of four simultaneous Markov chain Monte Carlo (MCMC) chains was run over 20 million generations with trees sampled every 100 generations. The sampling of the posterior distribution was considered to be adequate when the average standard deviation of split frequencies was lower than 0.01. Chain convergence was determined by checking the effective sampling size (ESS > 200) in Tracer 1.7 (Rambaut et al. 2018). By omitting the first 25% of trees as burn-ins using the “sump” and “sumt” commands, a majority rule consensus tree was generated.

Molecular phylogenetic species of the *P. ostreatus* species complex were delimited according to the genealogical concordance phylogenetic species recognition (GCPSR) and genealogical non-discordance criteria (Dettman et al. 2003, 2006). In brief, two GCPSR-based criteria must be fulfilled when a phylogenetic species is defined: (1) the clade is well supported in the majority (3/4) of the single-locus genealogies; (2) the clade is well supported by at least one single-locus genealogy, and is not contradicted in any other single-locus genealogy at the same level of support. Such clades were assessed using both ML bootstrap (MLB ≥ 70%) and Bayesian posterior probabilities (BPP ≥ 0.95). Each phylogenetic species had to be distinct and well differentiated from the other species, and all individuals had to be placed within the same phylogenetic species. For any clade which failed to obtain multi-gene sequences from at least two samples, it was also accepted as a potential independent phylogenetic lineage if it showed long branches as sequence variation from its sister groups and it possessed stable morphological difference.

Finally, five data matrices were compiled for different analyses. Dataset I (ITS) was used to identify most species of *Pleurotus* and *Hohenbuehelia* on the basis of molecular evidence and the previous phylogenetic research (Li et al. 2017; Zhao et al. 2016b). Dataset II (*RPB2*) was used to detect potential phylogenetic species. Dataset III (40 nuclear single-copy orthologous gene matrices and concatenated tree) was used to test the monophyly of the *P. ostreatus* species complex and for phylogenetic species recognition. Using TBLASTN from the DOE Joint Genome Institute (JGI, http://jgi.doe.gov/fungi), nucleotide sequences homologous to these single-copy genes were sought in whole genome shotgun sequencing datasets from 30 species in the Agaricales, Boletales, Polyporales, Russulales and other orders (Additional file 4). Dataset IV (40 single-copy orthologous genes) included an extensive sampling of non-*Pleurotus* outgroups from the JGI, and was used to estimate divergence time of the *P. ostreatus* species complex. Dataset V (40 single-copy orthologous genes) was subset of Dataset III, but was mostly reduced to one or two terminal per phylogenetic species from different geographical areas. It was used for diversification analyses and ancestral state reconstruction.

### Calibration procedure

A secondary calibration procedure from Renner (2005) has been used for fungi in several studies (Cai et al. 2014; Feng et al. 2012; Han et al. 2018; Wilson et al. 2012) and was employed in this study to estimate node ages. Dated phylogenies for both Datasets IV and V were reconstructed in BEAST v1.8.1 (Drummond et al. 2012). For Dataset IV, we used the calibration: the divergence between Ascomycota and Basidiomycota inferred from the fossil *Paleopyrenomycites devonicus* Taylor, Hass, Kerp, M. Krings & Hanlin. For the calibration, a normal distribution was applied by setting the mean and the standard deviation to 582.5 and 50.15, respectively (Berbee and Taylor 2010; Lücking et al. 2009). All introns within 40 nuclear single-copy orthologous genes were removed from Dataset IV because of the difficulty in alignment when large numbers of less closely related taxa are present. We selected the best partition schemes and evolutionary models for Dataset IV using MrModeltest v.3.2.1 (Ronquist et al. 2012) (Additional file 3). BEAUti v1.8.1 settings employed a Bayesian uncorrelated lognormal relaxed molecular clock and a Yule prior. Two independent MCMC runs were conducted for 100 million generations, sampling every 5000 generations. Log files of the two runs were combined using LogCombiner by setting the 25% logs as burn-ins and then analyzed in Tracer 1.7 (Rambaut et al. 2018) to confirm that the analysis reached a stationary distribution. The resulting trees were also interpreted in TreeAnnotator v1.8.1 to achieve a maximum clade credibility (MCC) tree. The estimated crown age of the *P. ostreatus* species complex by Dataset IV was used as the calibration point to date Dataset V by setting the prior to a normal distribution. The selection of evolutionary models of Dataset V, and the other protocols, followed the methods given for Dataset IV.

### Diversification analysis

We employed several packages in R 3.1.3 (R Development Core Team 2014) to explore changes in the diversification rate within the *P. ostreatus* species complex over time (Additional file 5). First, a maximum likelihood method implemented in the package “Laser” (Rabosky 2006) was used to determine if the diversification rate changed over time. Two rate-constant models (pure birth and birth–death) and three rate-variable diversification models (a logistic density-dependent, an exponential density-dependent and a yule2rate model) were fitted to the MCC tree (Akaike 1981). Second, we visually assessed the timing and tempo of diversification by constructing lineages through time (LTT) plots using “Ape” (Paradis et al. 2004). Third, we applied Bayesian analysis of macroevolutionary mixtures (BAMM 2.5.0) and “Bammtools” (Rabosky et al. 2014) to detect the location of diversification rate shift within the *P. ostreatus* species complex. Five species or varieties (*P. columbinus* Quél., *P. eous* (Berk.) Sacc., *P. subareolatus* Peck, *P. eryngii* var. *thapsiae* Venturella et al., *P. eryngii* var. *tingitanus* Lewinsohn) according to our phylogenetic tree of ITS and previous studies (Zervakis et al. 2014, 2019; Zervakis and Balis 1996) were not included in diversification analyses, so a command about “useGlobalSamplingProbability = 1; globalSamplingFraction = 0.80” was setting in controlfile to generate unbiased estimates of speciation and extinction.

### Reconstruction of ancestral state

Seven areas were delimited based on the tectonic history of continents and the distribution data of *Pleurotus*: A = East Asia; B = West Asia; C = North America; D = Europe; E = South America; F = Africa. Each sample was coded on the basis of its collection locality according to the field notes and references. Based on the MCC tree from BEAST analysis of Dataset V, RASP 3.2 (Yu et al. 2015) was used to reconstruct ancestral areas through Bayesian binary MCMC (BBM) analysis and dispersal-extinction-cladogenesis (DEC) analysis. MCMC chains in the BBM were run for 10 million generations with a sampling frequency of 100, excluding the first 1000 generations as burn-in.

## RESULTS

### Phylogenetic analysis

Of the 50 nuclear single-copy orthologous genes, forty were shared between at least 30 samples and were therefore used for reconstructing phylogenies. Thus, we got 40 single-copy nuclear sequence matrices rather than 50 for phylogenetic analysis. After removal of all sequences of poor quality, 1759 single-copy nucleotide sequences of 51 *Pleurotus* species and outgroup species were newly sequenced in this study; these sequences were deposited in GenBank (GenBank accession numbers: MN546051-MN546733, MN557857-MN558583, MN565733-MN565779, MN919217-MN919343, MN974593-MN974674, MT157415-MT157507). None of the ML trees from the individual gene analyses revealed any major conflicts among the datasets. Each nuclear single-copy orthologous gene analyzed separately resulted in fairly unresolved topologies, but they were all consistent with one another (Additional file 6). The concatenated dataset, after exclusion of the ambiguously aligned regions, had an alignment length of 12,995 bp. When all of nuclear single-copy orthologous genes were concatenated, they resulted in a single, completely resolved phylogeny.

**Fig. 1:**
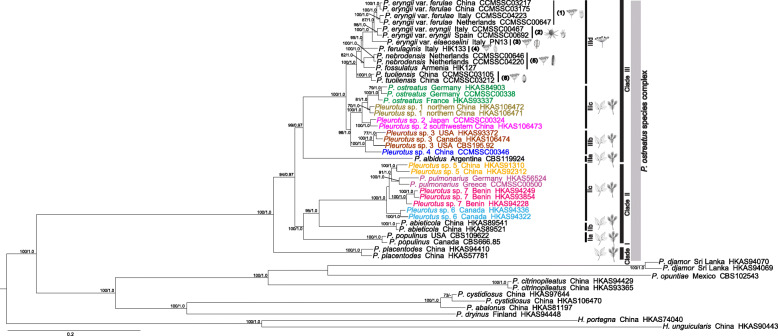
Phylogenetic tree generated from a forty-locus dataset. The branch support values are indicated by the numbers above the branches (MLB ≥ 70%, BPP ≥ 0.95). The *P. ostreatus* species complex is marked on the right of the phylogenetic tree, three major clades and seven subclades are in the black frames. Every phylogenetic species in Clades IIc, IIIb and IIIc is marked with different colors. The line-drawings of substrate plants and seeds are from Flora of China and Google. Table 1 includes the details of reference collections for all species

The phylogenetic analyses with a concatenated forty-gene dataset strongly supported the monophyly of the *P. ostreatus* species complex (100% MLB and 1.0 BPP value; Fig. 1). Within the *P. ostreatus* species complex, three distinct clades (Clades I, II and III) can be discerned, all of which are well-supported under both the maximum likelihood and Bayesian analyses. Some of these clades were further segregated into smaller groups with strong support (Fig. 1). Clade I was the basal most lineages in the *P. ostreatus* species complex and was sister to the Clades II and III collectively. Clade II was sister to Clade III, and consisted of three subclades of samples of North American, East Asian, European and African origin, respectively. The three subclades, named here Clades IIa, IIb, and IIc, could be inferred. Although characterized by short internal branches, the phylogenetic relationships within Clade IIc were resolved into four distinct lineages from East Asia, Europe, North America and Africa, respectively with 100% bootstrap support. Clade III was the largest of the three clades, and had four subclades (Clades IIIa, IIIb, IIIc, and IIId) with high statistical support. Clade IIIa, at the base of Clade III, formed a unique clade consisted of a single collection from South America, and its phylogenetic relationship to the taxa occurring in the Northern Hemisphere was highly supported (99% MLB; 0.97 BPP). Clades IIIb and IIIc were well supported as sisters to Clade IIId (100% MLB; 1.0 BPP). Clade IIIb (100% MLB; 1.0 BPP) included two lineages from North America and East Asia, respectively. In Clade IIIc, the collection from southwestern China clustered with Japanese accession with high support (100% MLB; 1.0 BPP), and were together a sister group to the north Chinese collections and European collections. Phylogenetic analyses also identified a relatively recent and rapid species-rich radiation within Clade IIId, which comprised close to two thirds of the phylogenetic species in Clade III. Furthermore, Clade IIId was a monophyletic group, and phylogenetic analysis placed *P. tuoliensis* as the most basal species of the subclade. Additionally, *P. eryngii* var. *ferulae* from Europe was clustered with a counterpart from northwestern China with of 100% MLB support.

### Phylogenetic species recognition

The maximum likelihood analysis was conducted using ITS sequences from 120 taxa, with *Hohenbuehelia* designated as outgroup. Sequences of ITS rDNA showed limited variation among the species within the *P. ostreatus* species complex. Most phylogenetic relationships among species of the complex were not well resolved (Additional file 7). The phylogenetic relationships of the Clades IIIb and IIIc remained uncertain. Within Clade IIIc, several samples were clustered with three specimens named ‘*P. spodoleucus* (Fr.) Quél.’ and ‘*P. columbinus*’, respectively, while the others were nested with the Clade IIIb and two specimens named ‘*P. eous*’. Similarly, the phylogenetic relationships between *P. eryngii* and its varieties were not clear. Some good ML bootstrapping support in the terminal clades in this dataset generally allowed us to partly delineate species boundaries. The preliminary phylogenetic analyses of ITS rDNA sequences and from the *P. ostreatus* species complex reference taxa distinguished 12 major clades.

As shown in Additional file 8, of the five species chosen as references for identifying the range of intra-specific variation of *RPB2* sequences, *P. abieticola* showed the highest value (0.93%), while the lowest (0) was observed in *P. tuoliensis* (C.J. Mou) M.R. Zhao & J.X. Zhang. Thus, 0.93% was chose as the cutoff value for the phylogenetic species identification using *RPB2* sequences. Taking this value as a threshold, *Pleurotus* sp. 3 and *Pleurotus* sp. 4 with a high inter-specific variation (1.74%) would be regard as independent species, respectively. Meanwhile, our provisionally adopted “pulmonarius-clade” and “ostreatus-clade” showed intraspecific divergence much higher than 0.93% (1.81% and 1.08%, respectively). Both the two temporarily accepted species consisted of more than one species based on the intra-specific variations and phylogenetic tree (Additional file 9), and therefore may represent more separate species, respectively. Other provisionally adopted species showed relatively high interspecific and low intraspecific variations and thus they were most likely valid phylogenetic species.

Among all twenty of the recognized phylogenetic species of the *P. ostreatus* species complex, fifteen of them fulfilled the GCPSR criterion. Among the fifteen phylogenetic species, three (*P. abieticola*, *P. placentodes* and *P. populinus* O. Hilber & O.K. Mill.) were strongly supported by more than thirty genealogies, four (*P. nebrodensis* (Inzenga) Quél., *P. tuoliensis*, *Pleurotus* sp. 6 and *Pleurotus* sp. 7) were strongly supported as monophyletic by MLB (≥ 70%) and BPP (≥ 0.95) in more than twenty individual gene trees, two (*P. ostreatus* and *Pleurotus* sp. 3) were resolved as monophyletic in more than ten individual gene trees, and the other six of them, namely *P. eryngii* var. *eryngii*, *P. eryngii* var. *ferulae*, *P. pulmonarius* (Fr.) Quél., *Pleurotus* sp. 1, *Pleurotus* sp. 2 and *Pleurotus* sp. 5, only formed monophyletic groups in less than ten gene trees (Additional files 6, and 10). *Gsh1* gene had the highest resolution and ten phylogenetic species (*P. abieticola*, *P. eryngii* var. *eryngii*, *P. eryngii* var. *ferulae*, *P. nebrodensis*, *P. placentode*, *P. pulmonarius*, *P. tuoliensis*, *Pleurotus* sp. 1, *Pleurotus* sp. 2 and *Pleurotus* sp. 5) formed monophyletic groups in the gene tree. Although one putative species lineage (*P. albidus* (Berk.) Pegler, *P. eryngii* var. *elaeoselini* Venturella et al., *P. ferulaginis* Venturella et al., *P. fossulatus* (Cooke) Sacc., *Pleurotus* sp. 4) was represented by single collection and therefore the monophyly could not be tested, they were considered to be phylogenetically distinct because they were genetically divergent from their sisters and could be clearly recognized by other means. We recognized a total of 20 phylogenetic species based on the criteria listed above (Fig. 1; Additional files 6, and 10): seven were revealed here for the first time, and 13 could be assigned to formally described species. Taxonomic treatment of the *P. ostreatus* species complex with seven species (*P. abieticola*, *P*. cf. *floridanus*, *P. eryngii*, *P. ostreatus*, *P. placentodes*, *P. pulmonarius* and *P. tuoliensis*) according to Li et al. (2017) was included with our results, which were strongly supported. Clade I comprised only *P. placentodes*, known from the subalpine habitat of the eastern Himalayan and Hengduan Mountains region, southwestern China (Liu et al. 2016). *Pleurotus abieticola*, *P. populinus*, *P*. *pulmonarius*, *Pleurotus* sp. 5, *Pleurotus* sp. 6 and *Pleurotus* sp. 7 were clustered together in Clade II. *Pleurotus albidus*, *P. ostreatus*, *Pleurotus* sp. 1, *Pleurotus* sp. 2, *Pleurotus* sp. 3 and *Pleurotus* sp. 4 were clustered with *P. eryngii* var. *elaeoselini*, *P. eryngii* var. *eryngii*, *P. eryngii* var. *ferulae*, *P. ferulaginis*, *P. fossulatus*, *P. nebrodensis*, and *P. tuoliensis* in Clade III. The phylogenetic tree also showed that *P. tuoliensis*, and *P. nebrodensis* were distinct species respectively, rather than varieties of *P. eryngii*.

### Bayesian estimation of divergence times of *P. ostreatus* species complex

**Fig. 2:**
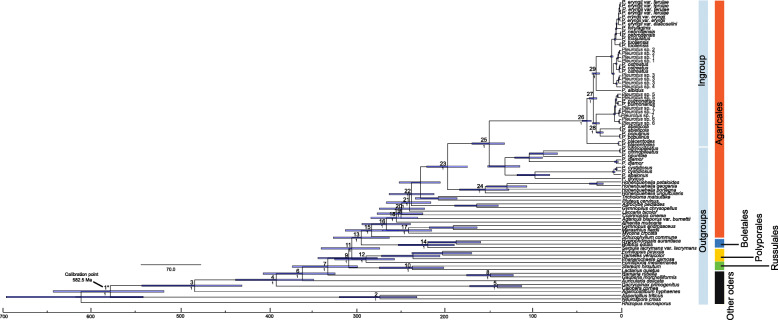
Maximum clade credible tree of *Pleurotus ostreatus* species complex based on Dataset IV. The chronogram was obtained using the Ascomycota-Basidiomycota divergence time of 582.5 Ma as the calibration point. Nodes are posterior mean ages (Ma) with blue node bars representing 95% highest posterior density intervals. The calibration point and object of this study are marked in the chronogram. The numbers at nodes indicate the important divergence events. Different orders of fungi included in the BEAST analysis are indicated by different colors used for the species names

The mean ages, 95% HPD intervals and Bayesian Posterior Probability values of all labelled nodes within *Pleurotus* are indicated in the chronogram (Fig. 2) and in Table 2. Topologies obtained for the Bayesian consensus tree (Fig. 1) and BEAST analysis (Fig. 2) based on the forty-gene dataset were almost identical. Our Bayesian dating, based on Dataset IV, suggested that the stem age of *Pleurotus* was estimated to be 153 Ma (135–171 Ma, 95% HPD; node 25) during the late Jurassic. The data suggest an ancient divergence of the major lineage *P. ostreatus* species complex from allied taxa during the late Jurassic, with the diversification of lineages within the complex having occurred mainly during the late Eocene (39 Ma, 33–44 Ma, 95% HPD; node 26). The earliest divergence formed Clade I, comprising collections from the eastern Himalayan and Hengduan Mountains region (HHM) (Fig. 2; Table 2). Clades II and III diverged at about 32 Ma (27–35 Ma, 95% HPD; node 27). The crown ages of Clades II and III were estimated at about 28 Ma (24–32 Ma, 95% HPD; node 28) and 29 Ma (24–32 Ma, 95% HPD; node 29), respectively.

**Table 2:** Estimated node ages and 95% HPD (Ma)

Clade lable for crown nodes	Node	Median age (Ma)	95% HPD (Ma)	Posterior Probability
Ascomcota/Basidiomycota	1	591	529–653	1
Ascomcota	2	280	237–325	1
Pucciniomycotina/Basidiomycota	3	492	437–546	1
Dacrymycetes/Agaricomycetes	4	398	357–442	1
Dacrymycetes	5	143	117–169	1
Auriculariales/Agaricomycetes	6	368	330–408	1
Gomphales-Hysterangiales/Agaricomycetes	7	340	305–376	1
Gomphales-Hysterangiales	8	152	128–178	1
Russulales-Hymenochaetales-Polyporales/Agaricales-Boletales	9	315	283–349	1
Russulales	10	241	209–275	1
Hymenochaetales-Polyporales	11	311	279–344	1
Hymenochaetales	12	296	264–329	1
Boletales/Agaricales	13	301	271–334	1
Boletales	14	224	196–253	1
Agaricales	15	289	259–320	1
	16	272	244–302	1
	17	261	234–290	1
	18	248	219–276	1
	19	256	230–284	1
	20	255	228–282	1
	21	246	220–273	1
	22	244	218–270	1
*Pleurotus*/*Hohenbuehelia*	23	201	179–224	1
*Hohenbuehelia*	24	158	132–185	1
*Pleurotus*	25	153	135–171	1
*Pleurotus ostreatus* complex	26	39	33–44	1
	27	32	27–35	1
	28	28	24–32	1
	29	29	24–32	1

### Diversification of the *P. ostreatus* species complex

**Fig. 3:**
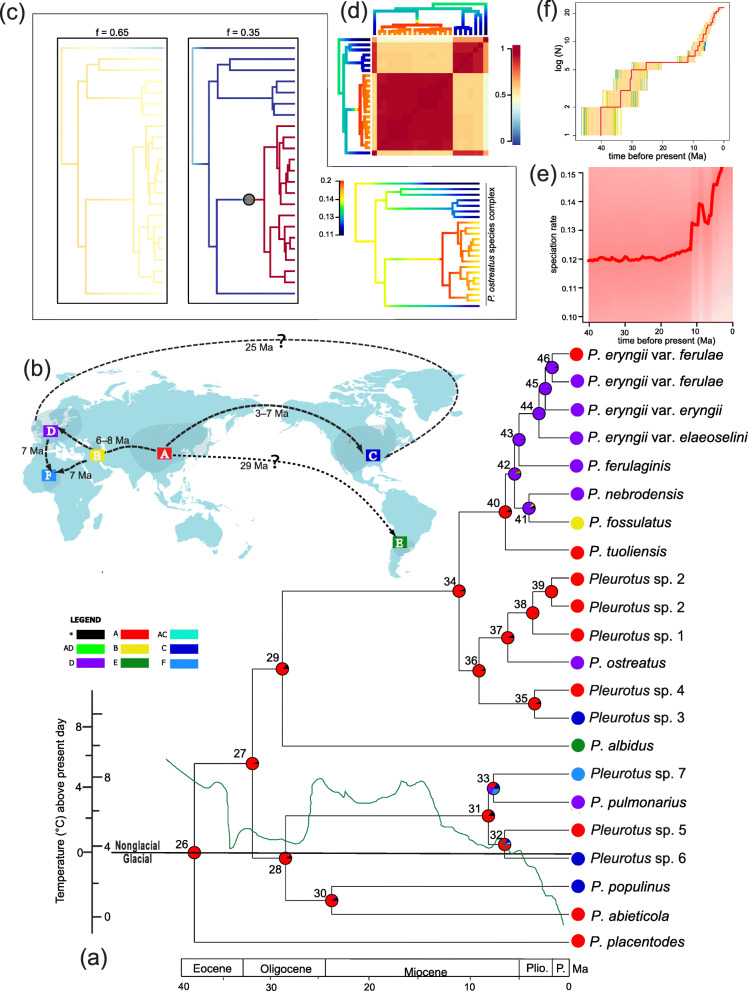
Chronogram of *Pleurotus ostreatus* species complex. The time-scale is set to the mean divergence dates produced in BEAST. Numbered nodes refer to mean divergence dates and their 95% HPD provide in Table 3. Small colored boxes indicate coded character states. **a** Ancestral area reconstruction with pie charts colored according to area. The green curve represents global temperature change in the geological history according to Zachos et al. (2001). **b** The area division and inferred dispersal route of *P. ostreatus* species complex. **c** A phylotate plot for distinct net diversification rates by mapping colors to rates on all branches, and 95% credible set of macroevolutionary shift configurations identified based on branch-specific marginal odds ratios. **d** Macroevolutionary cohort analysis with pairwise probabilities. **e** Plots of net diversification rates through time for lineages with 95% credible interval. **f** LTT plots from 95% HPD trees and a consensus tree annotated from the BEAST analysis

Our Laser results supported a pure birth model of diversification, with the diversification rate-constancy statistic ΔAIC_RC_ being − 0.69. Furthermore, the LTT plot suggested that lineages within the *P. ostreatus* species complex mainly accumulated during the late Eocene and the late Miocene, the diversification rate within the *P. ostreatus* species complex increased significantly in the late Miocene especially (Fig. 3f). One of the two tested prior (2) from the BAMM analyses identified only one rate shift at the stem of subclades IIIb, IIIc and IIId; however, the remaining prior (1) consistently showed no node as having potential shift (Fig. 3c). In the maximum a posteriori configuration, a rate shift at the stem of subclades IIIb, IIIc and IIId had a marginal shift probability (Fig. 3c). The rate-through-time plots suggested that the global speciation rate of the *P. ostreatus* species complex significantly accelerated from the late Miocene towards the present (Fig. 3e). The increases identified from the speciation rate-through-time curve were concordant with the positions of rate shifts along the phylogeny (e.g. stem of subclades IIIb, IIIc and IIId; Fig. 3c, e). In addition, the macroevolutionary cohort analysis for diversification revealed a certain level of heterogeneity in the diversification regimes within the *P. ostreatus* species complex (Fig. 3d).

### Ancestral area reconstruction of the *P. ostreatus* species complex

The *P. ostreatus* species complex was inferred to have a mean crown age of 39.05 Ma (33.84–44.11 Ma, 95% HPD) by Dataset IV, and this value was used as a calibration point for estimating the ages of major clades by setting the prior to a normal distribution, with the mean and standard deviation set to 39.05 and 2.62, respectively, which covers the 95% HPD of the crown age. The optimized ancestral areas inferred for internal nodes of Dataset V are mostly similar in the BBM and DEC analyses and supported East Asia as the center of origin for the *P. ostreatus* species complex: estimated ancestral distributions for root nodes of the complex and its major clades were all restricted to East Asia (Fig. 3a, Table 3). The East Asia clade (Clade I) became isolated from the ancestral *P. ostreatus* species complex lineage evolving in the remaining regions. Clades II and III were estimated to have the same ancestral area (East Asia), which therefore implies that several dispersal events to North America, Europe, and Africa occurred during the late Oligocene and late Miocene (nodes 30–33 for Clade II) and to South America, North America and Europe during the middle Oligocene and late Miocene to Pliocene (nodes 29, 35, 37, 40–41 and 46 in Clade III).

**Table 3:** Divergence time estimates of BEAST analyses for major nodes of *Pleurotus ostreatus* species complex

Node	Mean(95% HPD) Ma	Results of ancestral area reconstruction Area/RP	
		BBM	DEC
26	39 (33–44)	A/0.99	A/0.35
		NA/0.01	AC/0.16
			AD/0.15
			AE/0.13
			AF/0.11
			AB/0.10
27	32 (27–35)	A/0.97	A/0.67
		NA/0.03	AC/0.12
			AD/0.11
			AE/0.10
28	28 (24–32)	A/0.89	A/0.84
		AC/0.06	AC/0.16
		NA/0.05	
29	29 (24–32)	A/0.92	A/0.73
		NA/0.08	AE/0.17
			AD/0.10
30	25 (8–31)	A/0.96	A/0.60
		NA/0.04	AC/0.29
			C/0.11
31	8 (6–11)	A/0.88	AD/0.33
		NA/0.12	AF/0.31
			AC/0.13
			CD/0.10
			CF/0.09
			NA/0.04
32	7 (5–10)	A/0.81	AC/0.48
		C/0.12	A/0.40
		NA/0.07	C/0.12
33	7 (5–11)	A/0.32	D/0.32
		D/0.31	F/0.30
		F/0.23	DF/0.11
		C/0.05	A/0.08
		NA/0.09	AD/0.07
			AF/0.07
			C/0.05
34	11 (9–14)	A/0.96	AD/0.59
		NA/0.04	A/0.41
35	3 (2–4)	A/0.95	AC/1.0
		NA/0.05	
36	9 (8–11)	A/0.96	A/0.67
		NA/0.04	AD/0.22
			AC/0.11
37	6 (4–8)	A/0.93	AD/0.66
		NA/0.07	A/0.34
38	4 (2–6)	A/0.99	A/1.0
		NA/0.01	
39	2 (1–4)	A/0.99	A/1.0
		NA/0.01	
40	6 (5–8)	A/0.95	AD/1.0
		NA/0.05	
41	4 (2–7)	D/0.83	BD/0.63
		B/0.08	D/0.37
		NA/0.09	
42	5 (4–6)	D/0.83	D/1.0
		A/0.09	
		AD/0.06	
		NA/0.02	
43	5 (4–7)	D/0.99	D/1.0
		NA/0.01	
44	3 (2–4)	D/0.99	D/0.87
		NA/0.01	AD/0.13
45	2 (2–4)	D/0.99	D/0.78
		NA/0.01	AD/0.22
46	2 (1–3)	D/0.98	AD/0.81
		NA/0.02	D/0.19

RP represents relative probability

## DISCUSSION

### Phylogenetic reconstruction and rich diversity of the *P. ostreatus* species complex

The phylogenetic relationships of the *P. ostreatus* species complex were historically largely controversial, and were based only on phylogenetic comparisons of morphological characteristics and limited gene loci, such as the ITS, IGS, LSU, *TEF1α*, *RPB1*, and *RPB2* sequences (Avin et al. 2014; Estrada et al. 2010; Gao et al. 2008b; He et al. 2016; Li et al. 2017; Thorn et al. 2000; Zheng et al. 2006). The availability of whole-genome single-copy orthologous genes sequencing in the *P. ostreatus* species complex enables construction of a consensus phylogenetic gene tree, which offered an unprecedented opportunity to resolve this issue. Our results provide a solid basis for inferring the relationships among the taxa within the complex (Fig. 1). First, in our phylogenetic analysis, Clade IIIb was sister to Clade IIIc with high support (96% MLB; 1.0 BPP) (Fig. 1). Although the ML phylogenetic tree of the ITS dataset showed Clade IIIb clustered with *P. eous* (Additional file 7), the relationship was not strongly supported and *P. eous* has distinctive morphology (pileus color) and distribution (Pegler 1977). Second, we identified a single subclade marked as *P. albidus* (CBS119924) in Clade III, which occurs from Central America to Central Argentina on dead wood including *Salix* and *Ulmus* (Albertó et al. 2002). Third, *P. tuoliensis* was basal in Clade IIId, showing a close relationship with *P. nebrodensis* and *P. fossulatus* (Fig. 1). *Pleurotus eryngii* and its varieties, as well as *P. ferulaginis* formed a monophyletic group with high support (95% MLB; 1.0 BPP), which was consistent with Zhao et al. (2016b). In addition, although the northwestern Chinese population and the Mediterranean population of *P. eryngii* var. *ferulae* belong to the same genetic group according to phylogenetic analysis, they were highly differentiated based on genetic structure and pileus color (Lewinsohn et al. 2001; Urbanelli et al. 2003; Zhao et al. 2016a, 2016b). Our results also showed a more comprehensive molecular phylogenetic frame of *Pleurotus*, which was divided into three major clades (Fig. 1). The three major clades might represent three subgenera or three sections, and two clades (the *P. ostreatus* species complex and “cystidiosus-clade”) were consistent with Hilber (1982) (subgenera *Pleurotus*, *Coremioplaurotus*) respectively, the remaining one was unconformable.

Estimating worldwide species diversity for the *P. ostreatus* species complex has been difficult because of subtle morphological differences, and limited DNA sequence differences among different species (Gao et al. 2008a; Singer 1986; Zheng et al. 2006). Our forty-gene phylogenetic analysis of the global *P. ostreatus* species complex specimens based on high-throughput sequencing distinguished 20 phylogenetic species (Fig. 1), which is much more diverse than the previous studies (Li et al. 2017; Menolli Jr. et al. 2014; Zervakis and Labarère 1992). The number of phylogenetic species within Clade II increased from two (*P. abieticola*, *P. pulmonarius*) to six (*P. abieticola*, *P. populinus*, *P. pulmonarius*, *Pleurotus* sp. 5, *Pleurotus* sp. 6, and *Pleurotus* sp. 7). It was previously proposed that the North American population of *P. pulmonarius* is different from the European population, in morphology, mating behavior and other characters (Petersen and Hughes 1993; Vilgalys et al. 1993), and this was confirmed by our data. We recognized four phylogenetic species within Clade IIc following to GCPSR criterion, occurring in East Asia, Europe, North America, and Africa, respectively, although they are poorly differentiated morphologically (Fig. 1; Additional file 10). Similarly, taking account of genetic differences associated with geological origins, the terminal lineages of Clade IIIc were divided into three phylogenetic species (*P. ostreatus*, *Pleurotus* sp. 1 and *Pleurotus* sp. 2). Our results suggested that DNA sequences may have evolved faster than morphological characters in these phylogenetic species and these taxa might be relatively young, and have had insufficient time to accumulate morphological divergence. In Clade III, the total number of phylogenetic species increased significantly, with seven taxa belonging to Clade IIId. Of these, four are sympatric (*P. eryngii* var. *elaeoselini*, *P. eryngii* var. *eryngii*, *P. ferulaginis*, and *P. nebrodensis*), and are mostly restricted to Mediterranean Europe from sea level to 2000 m, one species is common to West and Central Asia (*P. fossulatus*), one to northwestern China and West Asia (*P. tuoliensis*), one to Mediterranean Europe and northwestern China (*P. eryngii* var. *ferulae*) (Fig. 1).

### Dating of the origin of the *P. ostreatus* species complex

To our knowledge, the dating analyses in our study have provided the first deep insight into the temporal and spatial evolution of the *P. ostreatus* species complex. Our data suggest that divergence of the genus *Pleurotus* probably occurred in the late Jurassic (153 Ma, node 25; Fig. 2; Table 2) as ancient as the previously suggested 200 Ma (Vilgalys and Sun 1994b). Considering the fossil record documented the existence of Pinaceae by the early Jurassic and Betulaceae, Fagaceae, and Salicaceae by the middle Cretaceous (Lin et al. 2010; Magallón et al. 2015), this estimation seems reasonable. However, the most recent common ancestor of the *P. ostreatus* species complex was inferred to have originated in East Asia during the late Eocene (39 Ma, node 26; Fig. 2; Table 2), with the most basal modern species, *P. placentodes*, known from the subalpine habitat of the Himalayan Mountains. The divergences likely arose from the uplifts of the Qinghai-Tibet Plateau (QTP) and climate changes. The orogenic uplifts of the QTP are likely to have contributed strong vicariance forces that shaped the diversification process after the tectonic collision of India plate with Eurasia 50 Ma (Yang et al. 2009). The subsequent pulse of rapid uplift of the QTP occurred between 25 and 30 Ma (Wang et al. 2012), and this generally coincides with our date for the major diversification in the *P. ostreatus* species complex (32 Ma, node 27; Fig. 2; Table 2). The unique geological history of the QTP is thought to have generated heterogeneity in the soils, climate, and elevation across the region (Yang 2005). These changes arising from the uplift of the QTP might be potential drivers of evolutionary radiations in the *P. ostreatus* species complex, as has been suggested for many other organisms (Ran et al. 2006). On the other hand, based on our estimated timing for the earliest divergence of the *P. ostreatus* species complex (39 Ma, node 26; Fig. 2; Table 2), the period coincided closely with the second Eocene cooling between 42 and 38 Ma (Wolfe 1978, 1997; Zachos et al. 2001). The significant global cooling during the Eocene-Oligocene transition (35 Ma; Zachos et al. 2001) resulted in some species adapting cold temperatures and forced others to migrate towards lower altitudes. Stochastic changes in temperature may have led to the expansion and contraction of species to small regions (refugia) in East Asia, especially in southwestern China (Harrison et al. 1992; Qiu et al. 2011).

### Historical diversification and migration of the *P. ostreatus* species complex

Our data indicated that, like EMF and other SPF (Han et al. 2018; Hibbett 2001; Qin et al. 2018; Sato et al. 2017), there are distinct biogeographical patterns in the distribution of the species complex. Biogeographic analyses in our study suggested that intensification of aridity caused by rapid uplift of the QTP and retreat of the Tethys Sea in the late Miocene, climate cooling events in Quaternary glacial cycling, and substrate transitions might contribute jointly to rapid diversification of the species complex since 10 Ma (Fig. 3a, c, e, f).

The East Asian - North American floristic disjunction pattern has been well documented in many angiosperm taxa (Tiffney 1985a; Wen 1999; Wen and Ickert-Bond 2009; Yi et al. 2015). Recently, the dispersal-vicariance theory was used to explain the biogeographic pattern of mushrooms (Cai et al. 2014; Geml et al. 2006, 2008; Hibbett 2001). Two land bridges potentially served as routes for organism exchanges between Eurasia and North America: the Bering Land Bridge (BLB, Beringia) and the North Atlantic Land Bridge (NALB) (Graham 1999; Grímsson and Denk 2005; Poore 2008; Thiede and Eldholm 1983; Tiffney 1985a, 1985b; Tiffney and Manchester 2001; Wen and Ickert-Bond 2009). There are three asynchronous East Asian - North American disjunctions inferred in the *P. ostreatus* species complex, one early (25 Ma, node 30; Fig. 3a, b; Table 3) and the other two more recently: the *P. pulmonarius* siblings (7 Ma, node 32; Fig. 3a, b; Table 3) and species in Clade IIIb (3 Ma, node 35; Fig. 3 a, b; Table 3), respectively. All three divergence dates were more recent than the last appearance of the North Atlantic Land Bridge, which was terminated in the Eocene (40 Ma) (Graham 1999; Lickey et al. 2002). However, the North Atlantic Land Bridge cannot be excluded for the earlier migration (25 Ma, node 30; Fig. 3a, b; Table 3), as there was a possibility of a persisting bridge or closely spaced island chain through the middle Miocene (Tiffney and Manchester 2001). Similar to the previous studies, we conclude that the earlier dispersal (25 Ma) between East Asia and North America via the Beringia (Cai et al. 2014; Du et al. 2012; Hibbett 2001; O’Donnell et al. 2011; Qin et al. 2018) or the North Atlantic Land Bridge (Du et al. 2012; Skrede et al. 2011) might both have occurred, whereas the most possible dispersal corridor for the exchanges of *P. pulmonarius* ancient populations and Clade IIIb ancient populations could be the Beringia during late Miocene and middle Pliocene (Chen et al. 2015; Feng et al. 2012; Han et al. 2018). Subsequent vicariance events, such as the opening of the Bering Strait, and the disappearance of the North Atlantic Land Bridge would have acted to restrict gene flow and consequently leading to genetic diversification of the *P. ostreatus* species complex in the Old World and the New World.

The intercontinental distribution of *P. pulmonarius* siblings from East Asia, Europe, Africa and North America were indicative of dispersal before the formation of geographical barriers. After originating in East Asia, the divergence of *P. pulmonarius* siblings occurred relatively recently, and the first migration event from East Asia into Europe through Central Asia may have occurred in the late Miocene (8 Ma, node 31; Fig. 3a, b; Table 3). Eurasia was inferred to be the ancestral area for the African *Pleurotus* sp. 7 in our study (7 Ma, node 33; Fig. 3a, b; Table 3). Although multiple hypotheses might account for the spread of ancestral fungal lineage between Africa and Eurasia (Conti et al. 2002; Kosuch et al. 2001; Schatz 1996; Yuan et al. 2005; Zhou et al. 2012), according to the detail of the relative dispersal probability in ancestor area analysis (node 33; Fig. 3a, b), a pathway into Africa from southern Europe or Ancient Mediterranean - Central Asia in the late Miocene, is both plausible and parsimonious (Carranza and Arnold 2004; Guillaumet et al. 2006; Rögl 1997; Wolfart 1987). Together with the dramatic weakening of the African summer monsoon by the Tethys Sea shrinkage during the Tortonian, the arid and desert conditions of the Sahara constituted a strong barrier to gene flow and ultimately, adaption to novel habitats and divergence in allopatry may have occurred from that time on in the *P. pulmonarius* siblings (Boratynski et al. 2012; Brito et al. 2014; Dobigny et al. 2005; Zhang et al. 2014). During the late Miocene (7 Ma, node 32; Fig. 3a, b; Table 3), a migration event from East Asia to North America might have occurred via Beringia as mentioned above, and formed an ancient range throughout the Northern Hemisphere. Subsequently, allopatric divergence may have arisen along with the intermittent disappearance of Beringia (Cai et al. 2014; Manos and Stanford 2001). Consequently, the distribution patterns of *P. pulmonarius* sibling species have gradually established in the Northern Hemisphere and Africa due to fragmentation of the ancient range.

Results from this study show strong patterns of geographical division within the saprotrophic *P. ostreatus* species complex, but occasional long-distance dispersal (LDD) events, shaping the geographical distribution of the extant *P. ostreatus* species complex between East Asia and South America also seemed to occur (node 29; Fig. 3a, b; Table 3). Although oceans are effective barriers to gene exchange, long distance dispersal of fungi prior to anthropogenic influences could potentially entail transoceanic dissemination of basidiospores by wind (Hibbett 2001; Hosaka et al. 2008; Ingold 1971; Liang et al. 2004; Matheny et al. 2009; Moncalvo and Buchanan 2008; Pady and Kapica 1955; Skrede et al. 2011). The role of long-distance dispersal has also been invoked to explain the current distributions of other groups of taxa in *Pleurotus* (Bresinsky et al. 1987; Zervakis et al. 2004; Zervakis and Venturella 1998). Meanwhile, spore-trapping has demonstrated that spores of certain species of the *P. ostreatus* complex may be ‘trapped’ even in areas outside of their known distributions (Vilgalys and Sun 1994a). In addition, a previous study confirmed that species of *Pleurotus* subgenus *Coremiopleurotus* produce basidiospores with a particular physiology of dormancy, which can withstand adverse environmental conditions over long distances and germinate only at relatively high temperatures and in the presence of suitable substrates (Lahouvaris et al. 1995; Zervakis et al. 2004). These suggest that the distribution of the *P. ostreatus* species complex is probably not limited by the ability to disperse over long distances. However, there was a possibility cannot be excluded, which the earlier migration from East Asia to North America via the North Atlantic Land Bridge or the Beringia as well as to South America until closing of the Isthmus of Panama during 3.5 Ma (29 Ma, node 29; Fig. 3a, b; Table 3).

### Evolution and speciation triggered by climatic change and substrate specificity

Speciation and development of species richness appear to have been facilitated by climatic factors. At the boundary of the Eocene and the Oligocene (about 33 Ma), the rapid drop in temperature may have stimulated the radiation of cold-adapted groups in fungi, such as *Amanita*, *Boletus* as well as *Cudonia* and *Spathularia* (Feng et al. 2012; Ge et al. 2014; Geml et al. 2006). Our estimation of the initial diversification of the *P. ostreatus* species complex was consistent with this climate change pattern (node 27; Fig. 3a; Table 3), and suggests that the global cooling may have triggered evolutionary radiations of the *P. ostreatus* species complex in the Northern Hemisphere. This may explain why some extant species of *P. ostreatus* complex, such as *P. abieticola*, *P. ostreatus*, *P. placentodes*, and *P. pulmonarius*, possess cold-adapted features (Bresinsky et al. 1987; Liu et al. 2015, 2016; Vilgalys et al. 1993). Cases of very recent radiation offer opportunities to examine the role that palaeoclimatic factors play in speciation (Du et al. 2016; Hewitt 1996, 2000; Klicka and Zink 1999). Examination of the speciation pattern of Clade IIId, suggested that this group was influenced by the Quaternary climatic oscillations and associated environmental changes in Europe between the glacial and interglacial phases (Gόmez and Lunt 2007; Hewitt 1996, 1999, 2000, 2004; Qiu et al. 2011; Zervakis et al. 2001, 2014). Therefore, the richness of cold-tolerant species and variety taxonomic levels of Clade IIId in Europe may have accumulated over several Ice Ages (Hewitt 1999; Svenning 2003). Likewise, the stronger cold tolerance of species in Clade IIId might have contributed to its survival during the Pliocene and the Quaternary Ice Age, which probably explains why many species need low temperatures for fruitbody formation (Hua et al. 2017, 2018; Lu et al. 2015).

A terrestrial shift towards dryer condition occurred concurrently along the northern rim of the Mediterranean and Central Asia, where the herbs better suited for drier conditions proliferated due to the uplift of the Tibetan Plateau, the retreat of the Tethys Sea and ongoing global cooling during the Late Miocene (Liu et al. 2014; Lu et al. 2010; van Dam 2006). In many extant plant species, adaptation to dry condition is known to be the main driving force of diversification and rapid radiations (Fiz et al. 2002; Ogburn and Edwards 2015; Verboom et al. 2004). Similarly, it is probable that the continued intensification of aridity in the Plio-Pleistocene had a significant influence on diversification within Clade IIId. In addition, ancestral area reconstruction analysis indicated that a single colonization event took place within *P. eryngii* var. *ferulae* from Europe back to northwestern China during the Quaternary (node 46; Fig. 3a, b). Distance isolation and allopatric divergence have been proposed to address the differentiation of the two populations in previous research (Zhao et al. 2016a). It is of note that the two populations from the northwestern China and Mediterranean regions inhabited distinct climatic environments, dominated by temperate steppe with a temperate grassy climate, and by garigue, wasteland and pasture with a subtropical Mediterranean climate, respectively (Chen 1986; Zervakis et al. 2014). The differences in climatic traits would be conducive to promote divergence in the individual populations, such as some candidate genes related to stress responses and DNA repair revealing adaptation to the different environments, and the long-term environmental heterogeneity could promote ongoing allopatric divergence in different populations (Dai et al. 2019; Pildain et al. 2009). However, the distribution pattern of *P. eryngii* var. *ferulae* cannot be excluded for insufficient samplings, which there remained a scattered distribution from Europe to East Asia since the quaternary glaciation.

The close association between fungi and plants is also likely to be an important factor influencing the distribution of fungi (Malloch et al. 1980). Well known as the white-rot SPF, the *P. ostreatus* species complex exhibits wood-decay properties that cause degradation of components of the substrate plant cell wall (PCW), including lignin, cellulose, and hemicelluloses (Sánchez 2009; Xie et al. 2016). Although belonging to the same complex, substrate specificity or preference for decayed wood is characterized by the *P. ostreatus* species complex taxa: most species of the *P. ostreatus* complex have been reported on deciduous and coniferous trees, such as Fagaceae, Betulaceae, Salicaceae and Pinaceae (Albertó et al. 2002; Liu et al. 2015, 2016; Petersen and Hughes 1997; Petersen and Krisai-Greilhuber 1996; Vilgalys et al. 1993; Vilgalys and Sun 1994b), whereas species in Clade IIId grow in association with plant roots or stems of Apiaceae (*Eryngium*, *Ferula*, *Ferulago*, *Cachrys*, *Laserpitium*, *Diplotaenia* and *Elaeoselinum*) (Boisselier-Dubayle 1983; Bresinsky et al. 1987; Hilber 1982; Joly et al. 1990; Mou et al. 1987; Venturella 2000, 2002; Venturella et al. 2016; Zervakis et al. 2014; Zervakis and Venturella 1998; Zhang et al. 2006; Zhao et al. 2016a, 2016b). Association with coniferous or deciduous logs appeared to be an ancestral character in the *P. ostreatus* species complex, followed by transitions to stems or roots of herbaceous Apiaceae. Recently, the genomic data provided insights into genomic basis of lignocellulose degradation mechanisms between *P. ostreatus* and *P. eryngii* (Xie et al. 2016; Yang et al. 2016; Zhang et al. 2018). Furthermore, it has been inferred that the substrate plants may have had an effect on the genetic differentiation among the species in Clade IIId due to competition for ecological niches (Zervakis et al. 2014).

## CONCLUSION

With samples of the *P. ostreatus* species complex from East Asia, Europe, North and South America, and Africa, a robust phylogeny of the *P. ostreatus* species complex was presented based on 1759 newly generated sequences of 40 nuclear single-copy orthologous genes, with a full resolution of the relationships of the major clades and the species. Here, The *P. ostreatus* species complex was strongly supported as monophyletic, including three major clades and seven subclades. Twenty phylogenetic species were recognized based on genealogical concordance phylogenetic species recognition (GCPSR), with seven putatively new species. The biogeographic analyses of the *P. ostreatus* species complex were conducted based on molecular clock estimation and ancestral area reconstruction for the first time. The data suggested that the most recent common ancestor of the *P. ostreatus* species complex diversified in the late Eocene (about 39 Ma) in East Asia, and diversification may have been triggered by the uplifts of the Qinghai-Tibet Plateau, the retreat of Tethys and intensification of aridity in the late Miocene, climate cooling events in Quaternary and substrate transitions to Apiaceae.

## Supplementary information

**Additional file 1:** Forward and reverse PCR primers for amplifying a short fragment of each single-copy gene.

**Additional file 2:** Reference of *Pleurotus* and *Hohenbuehelia* used in this study and their GenBank accession numbers.

**Additional file 3:** The best nucleotide substitution models used for dating analysis.

**Additional file 4:** Multiple alignment of nucleotide sequences of each single-copy gene in non-*Pleurotus* species for molecular clock analysis.

**Additional file 5:** R Commands to perform the analyses of diversification rates.

**Additional file 6:** Phylogenetic tree inferred from ML analysis based on each single-copy gene. Branch support values are indicated by numbers above branches (MLB ≥ 70%, BPP ≥ 0.95). Taxon labels are listed in Table 1.

**Additional file 7:** Phylogenetic relationships of *Pleurotus* inferred from ITS sequences using ML analysis. Branch support values are indicated by numbers above branches (MLB ≥ 70%). Accession numbers for sequences retrieved from GenBank database are listed in Additional file 2.

**Additional file 8:** Average evolutionary divergence over *RPB2* sequences pairs within and between groups (provincially adopted phylogenetic species) calculated by MEGA 5.

**Additional file 9:** Phylogenetic relationships of the *P. ostreatus* species complex inferred from *RPB2* sequences using ML analysis. Branch support values are indicated by numbers above branches (MLB ≥ 70%, BPP ≥ 0.95). Provisionally adopted names (based on tree topology) are listed. Accession numbers for sequences generated newly and retrieved from GenBank database are listed in Table 1.

**Additional file 10:** Criteria for phylogenetic species recognized by genealogical concordance in individual gene and in the combined dataset

## Data Availability

All data generated or analyzed during this study are included in this published article [and its supplementary information files].

## Abbreviations

EMF: Ectomycorrhizal fungi
SPF: Saprotrophic fungi
NGS: Next-generation sequencing
HKAS: Herbarium of Cryptogams, Kunming Institute of Botany of the Chinese Academy of Sciences
CCMSSC: China Center for Mushroom Spawn Standards and Control
ACAM: Herbarium of the Laboratory of the General and Agricultural University of Athens
CBS: Westerdijk Fungal Biodiversity Institute
CTAB: Cetyltrimethyl ammonium bromide
ITS: Internal transcribed spacer region
*RPB2*: The gene for RNA polymerase II second largest subunit
ML: Maximum likelihood
GCPSR: Genealogical concordance phylogenetic species recognition
MCC: Maximum clade credibility
LTT: Lineages through time
HHM: Himalayan and Hengduan Mountains region
QTP: Qinghai-Tibet Plateau
BLB (Beringia): Bering Land Bridge
NALB: North Atlantic Land Bridge
LDD: Long-distance dispersal events
PCW: Plant cell wall
