# Supplementary material for: The saprotrophic Pleurotus ostreatus species complex: late Eocene origin in East Asia, multiple dispersal, and complex speciation
Source: IMA Fungus. 2020 Jun 8;11:10. doi: 10.1186/s43008-020-00031-1 (PMC7325090; doi:10.1186/s43008-020-00031-1)
Supplement: Supplementary file 1 — Additional file 1: Forward and reverse PCR primers for amplifying a short fragment of each single-copy gene. [file 43008_2020_31_MOESM1_ESM.doc]

Additional file 1 Forward and reverse PCR primers for amplifying a short fragment of each single-copy gene

| Gene | Forword primer (5’- 3’) | Reverse primer (5’-3’) |
| --- | --- | --- |
| Atp2 (FG459)* | GTYCGYACYATTGCYATGGA | ACGTTGTTGATGAGYTCCTGRAT |
| Fba1 (FG507) | AGSTWCCTYGTCTTCCAYGGHGG | ACCTTCTTGTTRGGCTTRTC |
| Yhm2 (FG524)* | TTYACGACRTGYATGAARAC | ACYTCRATRGGYTGRTTCCA |
| Gus1 (FG525)* | ATGGAYTGGGGYAAYGCNATYGT | ARTATCCYTTCCTCTCRAAYTG |
| Elp3 (FG533)* | ATGACVGGNAAYATMTGYGT | TARTCYTCTGGCATRCTCATRAA |
| Hom3 (FG534) * | GAGGTGCAYGTBAGYATGGC | ATRACRCANGADATRTTGATCTC |
| Atp3 (FG543)* | AACATYGAGAAAATYACVAAG | ATACCRCCGCAVAGRCCYTTGTC |
| Pol30 (FG546) * | CARGCNATGGAYAACTCYCAYGT | TCRATRTCCATNAGYTTCAT |
| Gdi1 (FG576)* | AAGAAGGTSCTYCACATGGA | GCYTCCATYTCBGTRCTBGG |
| Sec17 (FG579) | GARGCTGCNAAYGCNTGGTGGAA | GCATCYTCYTGNGMRTACCA |
| Get3 (FG591)* | ATGRGYTTYGCDGARATYATGAA | GGRTCYTTGAAYTGNGTRTT |
| Ilv2 (FG595) * | GGNCARCAYCAGATGTGGGC | ACCATNCCYTGGAABTCGTTGTT |
| Cct2 (FG644) * | ATGGAYACYGAYAARATCAA | TCCTCGCCRATCATRATYTCYTC |
| Ade12 (FG673)* | AGCATCGGNACMACVAAGAA | CCRAARTCRATRTCVAGCAT |
| Hsp60 (FG691) * | AARGACAAGTTYGAGAAYCT | CCCTCCTTGCCRACYTTYTCCAT |
| Krr1 (FG695)* | AAAGCNCGNGAYATGATCAA | TATATNGGRTGRATRTTYTTCAT |
| Rfc2 (FG720)* | CCHCAYATGYTGTTCTAYGG | GTCATWGARTCKGCYTCRTC |
| Met6 (FG740)* | GTCGAYGAGCCNGCYATYCGTGA | TAGACACCVGGVCCRATCTGGTT |
| Qns1 (FG747)* | GCNTGYTGGCTBTGGGAYTA | CCCATRTARCANGTRTGGAA |
| Hem15 (FG756)* | CAGTAYCCBCARTAYAGYTGYAG | TCYCCRCGGTTBACVACYGACAT |
| Ygr207c (FG757)* | AARCARGCYATYGAYGAYGA | TTCTTYTTNGCYTTCATRATRTT |
| Rfc4 (FG761)* | AAYGTVTTCAAAGTBTGYGAYCA | CATRTGYGTRAABCCRATYTCCT |
| Arc40 (FG771) * | TGATCACNTCNATYGAYTGGGC | GTCGAYCKGATNGGYTTCTT |
| Tif5 (FG832) | GACAARTTYTAYCGCTAYCG | GTGGTSARCTTRTGSCKCATRTC |
| Uba3 (FG844)* | GARCAYTGYATHGARTGGGC | GCRATCTTGAANGCYTCRTTRCA |
| Uba1 (FG848)* | GARTTYGAGAAGGAYGAYGA | GGYTCNGAGAARCCRAAGAA |
| Tcp1 (FG850) * | TTYGTCGARGCTGGYGCNATGGC | ACCARTGTCGTNGCRAAGTTYTC |
| Pdb1 (FG855) * | GARTTCATGACNTTBAACTTYGC | AGGAAGACRACRGGRTTNGGRTC |
| Cct3 (FG861) * | AARGCCATGCTSAARATGAT | GARATGATSACGACNGGRTGRAT |
| Rrb1 (FG927) | GARGGBTTYGCNATGGAYTGGGC | TTCCARCTRATVACATTNACRTC |
| Sac6 (FG975) * | GAGCTBGAVGAYTGGGTHGAGGT | KRCABTCGTCRAAGAKYTGCAT |
| Nip1 (FG1010) | CCVTTYCAYATGCAYATCAACAC | ACTTGGCBARCATYTCYTTVAC |
| Mvd1 (FG1021) | TCVYRCAACAACTTCCCYAC | GTCTCGACGGTGCGYTGCATVCC |
| Gsh1 (MS320) * | AAYCCWCATGCNCGHTTYCCGT | CARCADCCCATBCCRAARCCCAT |
| Coq5 (MS348) | GTSCACMGGYTVTGGAARGA | CCRAAWGCRATGGTRTAVAGGTC |
| Trp2 (MS353) * | TACATGTTYTAYYTBGAYTGYGG | GTYARRTGRATGACRTGRCTGAA |
| Brx1 (MS361) | TGGGCTGCRAARACRCCNAAYGG | TGGAARTTNCGGAACCADATYTT |
| Ssl2 (MS413) | CAYGAVTCNAARAARATGATGGA | GGRCACCAHACYTCNGCRCACT |
| Ppt1 (MS417) * | AAYCAYGARGCRAARGABATGAA | GTRACRCCRTCYTTRCTGAA |
| Cct5 (MS422)* | GARAAGTTYGMGGAYATGATCAA | TCYTCRATDACVAGCATCTTRTC |
| Pre8 (MS429) * | GCAGCARGCMACDCARTCHGGGT | AGSGCKGTGTGRATNGCGTCYTC |
| Cap2 (MS437) | GCGAAYGAGGCBTTYGAYACNTA | TCRAADACRTGGATNGARTCCCA |
| Crm1 (MS442)* | ATGGTCAARCCNGARGAGGT | CGCTTYTCVGTYTCYTCRTCT |
| Ils1 (MS444) * | GAYGGMAAGAARATGAGCAARAG | AGRATCCAKCGRTCCATVACRTT |
| Cdc47 (MS456) * | TGCCTNATGGGTGAYCCHGGTGT | ATYGAGATYGTYTGYTGYTCCAT |
| Mcm2 (MS463) * | AARCGCATCRTCAARTCYATHGC | TCGTTCATYTTGTCRAAYTCRTC |
| Rio2 (MS481) * | TCNGCNTCRTGGATGTAYATGTC | ATGAGRATRTTGAAYTCRTT |
| Rpa135 (MS493) * | TATAYGGCAAYGARCCNATGTAYTC | GTHGARTARTCBGAGCARTTCAT |
| Frs2 (MS524)* | TTCMGMAAYGAGACNATGGAYGC | TCMARCATYTCDGGNCGGAACAT |
| Stt3 (MS561) * | ATCTTCCTBCTBATGTTCAC | CCRACRAARGGVACYTGCATRCT |

Gene markers used for further phylogenetic analyses and biogeographic analyses are labeled with *.
