# Supplementary material for: The saprotrophic Pleurotus ostreatus species complex: late Eocene origin in East Asia, multiple dispersal, and complex speciation
Source: IMA Fungus. 2020 Jun 8;11:10. doi: 10.1186/s43008-020-00031-1 (PMC7325090; doi:10.1186/s43008-020-00031-1)
Supplement: Supplementary file 2 — Additional file 2: Reference of Pleurotus and Hohenbuehelia used in this study and their GenBank accession numbers. [file 43008_2020_31_MOESM2_ESM.doc]

Additional file 2 Reference of *Pleurotus* and *Hohenbuehelia* used in this study and their GenBank accession numbers

| Taxon | Isolated ID | Location | ITS GenBank accession numbers |
| --- | --- | --- | --- |
| *Pleurotus abalonus* | CBS80391 | China | AY315806 |
|  | VT2476 | USA | AY315802 |
| *P. abieticola* | TENN52359 | Russia | AY450348 |
|  | TENN58284 | Russia | AY345656 |
|  | HKAS45720 | China | KP771696 |
|  | HKAS46100 | China | KP771695 |
| *P. agaves* | Colegio de Posgraduados CP-194 | Mexico | GU722262 |
|  | El Colegio de la Frontera Sur ECS-0165 | Mexico | GU722264 |
| *P. albidus* | BAFC50.261 | Argentina | AF345659 |
|  | Duke327 | Brazil | AF345658 |
| *P. australis* | PDD59215 | New Zealand | AY315761 |
|  | VT1953 | Australia | AY315758 |
|  | PDD87/021XP | New Zealand | AY315764 |
| *P. calyptratus* | CCRC36211 | — | AY265814 |
|  | C-1 | — | JQ837485 |
| *P. citrinopileatus* | HMAS63344 | China | AY696301 |
|  | TFM-M-E793 | — | AB115043 |
|  | HKAS85965 | China | KP867920 |
| *P. columbinus* | CBS281.32 | — | AY265815 |
| *P. cornucopiae* | H-14 | — | JQ837484 |
|  | TENN55191 | Austria | AY450341 |
| *P. cystidiosus* | IFO30607 | Japan | AY315778 |
|  | AG55 | USA | FJ608592 |
| *P. djamor* | SP445789 | Brazil | KF280329 |
| *P. dryinus* | CBS724.83 | Netherlands | EU424293 |
|  | 470 G III | — | KF932724 |
|  | 7239 | — | JF908617 |
| *P. eryngii* var. *eryngii* | HIK154 | China | HM998841 |
|  | HIK139 | Iran | HM998837 |
|  | HIK122 | Italy | HM998823 |
|  | UPA30 | Italy | HM998819 |
| *P. eous* | SP624-PE | India | KY214257 |
|  | P109 | South Korea | MG282448 |
| *P. euosmus* | CCRC36212 | — | AY265826 |
|  | CBS307.29 | UK | AY368659 |
| *P. ferulaginis* | PN15 | Italy | KF743833 |
|  | PN10 | Italy | KF743829 |
|  | LGMACC850404 | Hungary | HM998809 |
|  | HIK132 | Slovenia | HM998831 |
| *P. flabellatus* | ACCC51447 | — | EU424303 |
|  | P7 | India | KT970056 |
| *P. fossulatus* | ATCC52666 | India | AY265833 |
|  | ATCC90801 | — | FJ545251 |
| *P. giganteus* | MFLU14-0638 | — | KP135560 |
|  | HMAS P1 | China | KP793688 |
| *P. incarnatus* | CCRC36228 | — | AY265836 |
| *P. nebrodensis* | UPA6 | Italy | HM998816 |
|  | HIK125 | Greece | HM998826 |
|  | HIK137 | Iran | HM998835 |
| *P. ostreatus* | TENN53662 | Austria | AY854077 |
| *P. opuntiae* | ET3313 | Mexico | AY450339 |
|  | 6241 | Mexico | AY450340 |
| *P. placentodes* | HKAS57145 | China | KR827693 |
| *P. populinus* | TENN56749 | USA | AY450346 |
|  | ATCC90083 | — | AY368667 |
| *P. spodoleucus* | PHZAU7 | China | DQ077886 |
|  | ASI2012 | — | AY265848 |
| *P. subareolatus* | CCRC36226 | Canada | AY265849 |
| *P. tuberregium* | PTV2 | Ghana | AF109978 |
|  | PtWat | Cameroon | AF109988 |
|  | Pt1 | Nigeria | AF109983 |
| *P. tuoliensis* | CCMSSC02248 | China | KU612913 |
|  | CCMSSC02560 | China | KU612914 |
|  | CCMSSC01433 | China | KP867912 |
|  | HIK152 | China | HM998839 |
|  | HIK138 | Iran | HM998836 |
| *Pleurotus* sp. 2 | CCMSSC06141 | China | KP867915 |
| *Pleurotus* sp. 5 | HKAS73350 | China | KP867917 |
|  | HKAS76382 | China | KP867916 |
|  | HKAS76672 | China | AY696299 |
| *Pleurotus* sp. 6 | ECS-0158 | Mexico | GU722283 |
| *H. portegna* | HKAS82091 | China | KY426798 |
|  | A27 | — | KC414247 |
| *H. unguicularis* | CBS855.85 | Canada | MH861918 |
|  | DAOM155519 | Canada | KU355360 |
