# Supplementary material for: The saprotrophic Pleurotus ostreatus species complex: late Eocene origin in East Asia, multiple dispersal, and complex speciation
Source: IMA Fungus. 2020 Jun 8;11:10. doi: 10.1186/s43008-020-00031-1 (PMC7325090; doi:10.1186/s43008-020-00031-1)
Supplement: Supplementary file 3 — Additional file 3: The best nucleotide substitution models used for dating analysis. [file 43008_2020_31_MOESM3_ESM.doc]

Additional file 3 The best nucleotide substitution models used for dating analysis

| Gene matrix | Nucleotide substitution models | | |
| --- | --- | --- | --- |
|  | Dataset Ⅲ | Dataset Ⅳ | Dataset Ⅴ |
| Ade12 (FG673) | SYM+I+G | GTR+I+G | SYM+I+G |
| Arc40 (FG771) | HKY+G | GTR+I+G | SYM+G |
| Atp2 (FG459) | GTR+I+G | GTR+I+G | SYM+I+G |
| Atp3 (FG543) | HKY+I+G | GTR+G | HKY+I+G |
| Cct2 (FG644) | SYM+I+G | GTR+I+G | SYM+I+G |
| Cct3 (FG861) | GTR+I+G | HKY+I+G | SYM+G |
| Cct5 (MS422) | SYM+I+G | GTR+I+G | SYM+I+G |
| Elp3 (FG533) | SYM+I+G | GTR+I+G | SYM+G |
| Tcp1 (FG850) | K80+I+G | GTR+I+G | GTR+I+G |
| Frs2 (MS524) | HKY+I+G | GTR+I+G | SYM+I+G |
| Gdi1 (FG576) | GTR+I+G | GTR+I+G | SYM+I+G |
| Get3 (FG591) | SYM+I+G | GTR+I+G | K80+I |
| Gsh1 (MS320) | SYM+G | GTR+I+G | HKY+I |
| Gus1 (FG525) | GTR+I+G | GTR+I+G | GTR+I+G |
| Hem15 (FG756) | K80+I+G | GTR+I+G | K80+G |
| Hom3 (FG534) | SYM+I+G | GTR+I+G | SYM+I+G |
| Hsp60 (FG691) | HKY+I+G | GTR+I+G | HKY+G |
| Ils1 (MS444) | SYM+I+G | GTR+G | HKY+I+G |
| Ilv2 (FG595) | HKY+I+G | GTR+I+G | HKY+I+G |
| Krr1 (FG695) | K80+I+G | SYM+I+G | K80+G |
| Mcm2 (MS463) | HKY+I+G | GTR+I+G | GTR+I+G |
| Met6 (FG740) | HKY+I+G | GTR+I+G | HKY+I+G |
| Ppt1 (MS417) | HKY+G | GTR+I+G | HKY+I |
| Pre8 (MS429) | SYM+I+G | SYM+I+G | SYM+I+G |
| Crm1 (MS442) | SYM+I+G | GTR+I+G | K80+G |
| Cdc47 (MS456) | SYM+I+G | GTR+I+G | SYM+G |
| Pdb1 (FG855) | HKY+I+G | GTR+I+G | HKY+G |
| Pol30 (FG546) | SYM+I+G | GTR+G | SYM+I+G |
| Qns1 (FG747) | HKY+I+G | GTR+G | K80+I+G |
| Rfc2 (FG720) | SYM+I+G | GTR+I+G | SYM+G |
| Rfc4 (FG761) | SYM+G | HKY+I+G | SYM+G |
| Rio2 (MS481) | HKY+I+G | GTR+I+G | HKY+I+G |
| Rpa135 (MS493) | HKY+I+G | GTR+I+G | SYM+G |
| Sac6 (FG975) | GTR+G | GTR+G | GTR+G |
| Stt3 (MS561) | HKY+I+G | GTR+I+G | HKY+I+G |
| Trp2 (MS353) | HKY+I+G | GTR+I+G | HKY+I+G |
| Uba1 (FG848) | HKY+I+G | GTR+I+G | HKY+I |
| Uba3 (FG844) | HKY+I+G | HKY+I+G | K80+I+G |
| Ygr207c (FG757) | GTR+I+G | GTR+I+G | SYM+I+G |
| Yhm2 (FG524) | SYM+I+G | GTR+I+G | GTR+G |
