# Supplementary material for: The saprotrophic Pleurotus ostreatus species complex: late Eocene origin in East Asia, multiple dispersal, and complex speciation
Source: IMA Fungus. 2020 Jun 8;11:10. doi: 10.1186/s43008-020-00031-1 (PMC7325090; doi:10.1186/s43008-020-00031-1)
Supplement: Supplementary file 4 — Additional file 4: Multiple alignment of nucleotide sequences of each single-copy gene in non-Pleurotus species for molecular clock analysis. [file 43008_2020_31_MOESM4_ESM.pdf]

>Ade12 (FG673)_*Agaricus bisporus* var. *burnettii*
AGCATCGGAACCACGAAGAAAGGTATCGGTCCAGCCTATTCCGGCAAAGCTTCCCGTT
CCGGTCTTCGCGTCCACCACCTCTTCGACCATACCAACTTTGCAACCAAATTCCGTCAT
ATCGTCGAAGGTCGTTTCAAACGTTACGGTCATTTCGAATACGACACCGAAGGCGAAA
TCGAGAGATACAAACTCCTCGCAGAACGTTTGAAACCTTACGTTATTGATAGTGTGGTT
TATATTCATAAAGCTATCAGTCAAGGTAAACGTGTGTTGGTTGAAGGAGCGAATGCTTT
GATGCTGGATTTGGATTTTGG

>Ade12 (FG673)_*Schizophyllum commune*
AGCATCGGTACCACCAAGAAGGGCATCGGCCCAGCCTACTCTGGCAAGGCCTCCCGCT
CCGGCTTGCGCGTCCACCACCTTTTCGACCACGACACCTTCGCCGCCAAGTTCCGCCG
CCTCGTCGAAGGCCGCTTCAAGCGCTACGGCCACTTCGAGTACGACACCGAGGGCGA
AATCGAGCGCTACAAGCAGCTCGCGGAGCGCCTCCGGCCACACGTCATCGACAGCGT
CGCGTACATCCACAAGGCCATCTCGAGCGGGAAGCATGTGCTGGTCGAGGGCGCGAA
CGCGTTAATGCTGGATATCGACTTTGG

>Ade12 (FG673)_*Coprinopsis cinerea*
AGCATCGGAACAACCAAGAAGGGCATCGGCCCAGCCTACTCCGGCAAGGCCTCTCGC
TCCGGCCTCCGCGTGCACCACCTCTTCGAGCCCGAGACCTTCGCCGCCAAGTTCCGCA
AGGTCGTCGAGGGGCGGTTCAAGCGCTACGGGCACTTTGAGTACGACACCGAAGGCG
AGATCGAGCGGTACAGGAAGCTGGCGGAGAGGCTGCGCCCGTATGTCGTTGATAGCGT
TGCGTTCATTCATAAAGCCCTGGCGTCGGGGAAGAGGGTCCTGGTCGAGGGCGCGAAT
GCGTTGATGCTTGATATTGATTTTGG

>Ade12 (FG673)_*Fomitopsis pinicola*
AGTATCGGTACCACCAAGAGGGGTATTGGCCCAGCCTACTCCGGCAAGGCCTCGCGCT
CAGGCCTTCGCGTCCACCACCTCTTCGACCACGAGACTTTTGCAGCCAAGTTCCGCAA
AGTTGTTGAAGGCCGCTTCAAGCGCTACGGCCACTTCGAGTACGACACGGAGGGCGA
GATCGTCCGCTACAAGGTGCGTCGTCCCTGCCCCGTGTTCTTGCTCACTCGTCGCAATG
CAGGCCCTCGCTGAACGGTTACGTCCCTACGTGATCGACAGTGTCACGTACATACACC
GCGCGATCGCTGATGGCAAAAGGATTCTGGCCGAAGGCGCGAACGCGCTGATGCTCGA
TATTGACTTCGG

>Ade12 (FG673)_*Agrocybe pediades*
AGCATCGGAACCACAAGGAAGGGTATTGGCCCGGCCTACTCCGGAAAAGCCTCTCGTT
CGGGTCTCCGTGTTCACCACCTATTCGATCCCGAAACTTTCGCTGCCAAGTTTAGGACT
TTGGTGGAAGGTCGGTTCAAGCGATACGGGCACTTTGAGTATGACACCGAAGGAGAG
ATCAAGCGGTATAAGGTACGCCTCCTCTCTTACCCGCATATGTTGATTCTGTTTATTCTAG
GAACTCGCTGAGAGATTACGCCCCTACGTCGTCGACAGCGTCGTCTATATCCATAAAGC
CATCTCCTCTGGAAAGAGAGTTTTGGTCGAGGGTGCCAACGCGCTGATGCTCGACATT
GACTATGG
>Ade12 (FG673)_*Phanerochaete carnosa*
AGTATTGGAACAACCAAGAAGGGCATTGGCCCGGCCTACTCCGCCAAGGCGTCGCGCT
CTGGCCTCCGTGTTCACCATCTCTTCGATCATGAAACATTCGCCGCCAAATTCCGCAAG

ATCGTCGAGGGCAGATTTAAGCGCTATGGCTACTTCGAATATGACACCGAGGGCGAGAT
TGTCAGGTATAAGGTTCGTTTCCTTGCAAAGCTCTGACCACAACTTGTGCTGCAGGCA
CTTGCCGAACGGCTGAGGCCGTATGTTGTCGACAGCGTTATCTATATCCATGAGGCAAT
TGGCGAAGGCAAGAAGATTCTGGTCGAGGGTGCAAATGCTCTGATGCTCGACATCGAC
TTTGG

>Ade12 (FG673)_*Auricularia delicata*
AGTATCGGGACAACGAAGCGCGGCATCGGCCCCGCGTACTCCGCCAAGGCGTCGCGC
TCCGGCCTGCGCATCCACCACCTCTTCGACCACGACACCTTCGCTGCCAAGTTCCGCA
AGATCGTCGAGGGCCGGTTCAAGCGCTATGGGCACTTTGAGTATGATACTGAGGCTGA
GATCCTGCGGTACAAGGTACTACCGTGTCCCTGCTCCCTCCCCTCACGACGGCATGACG
CAGGAGCTGGCGGAGAGATTACGGCCGTTCGTCGTGGATAGCGTGTCGTATGTGCACG
AGGCGCTCGCCGCGGGAAAGCGCATCCTCGTCGAGGGCGGACAGGCGCTCATGCTCG
ACATCGACTTCGG

>Ade12 (FG673)_*Trametes versicolor*
AGCATCGGCACGACCAAGCGAGGCATCGGTCCTGCTTACTCGTCTAAGGCGTCTCGCT
CCGGTCTTCGCGTCCACCACCTCTTCGATCACGACACCTTCGCGCAGAAGTTCCGCAA
GCTCGTTGAGGGTCGTTTCAAGCGCTACGGCCACTTCGAGTATGACACCGAGGGAGAA
ATCCTCCGTTACAAGGTACAACATTGCCTCGCTTACCTATAACACACCTGGTTTACGCA
GGCCCTCGCGGAACGCCTGCGCCCCCACGTCATTGACTCCGTGGTTTACGTCCACAAA
GCGCTATCAGAGGGCAAGAAGCTTCTTGTTGAAGGCGCGAATGCGTTGATGCTGGATA
TCGACTTCGG

>Ade12 (FG673)_*Marasmius fiardii*
AGCATCGGGACCACCAAAAAAGGCATTGGTCCAGCATACTCGGGCAAAGCTTCACGAT
CGGGCCTCCGTGTTCACCATCTATACGACCACGACACCTTCGCCGGCAAGTTCAGAAA
GCTCGTCGAGGGTCGCTTCAAGCGCTACGGTCACTTCGACTACGACACAGAAGGTGA
AATATTACGTTACAAGGTTCATTTATCTCAATCTGGACTTAGTACTTCTGAACTGTCTTTA
GGACCTGGCTGAAAGACTCCGTCCATATGTCATTGACAGCGTTGTTTACATTCACCAAG
CCATCTCAAGTGGAAAGAACGTCCTGGTTGAAGGGGCAAACGCCCTGATGCTAGACAT
CGATTTCGG

>Ade12 (FG673)_*Laccaria bicolor*
AGCATTGGCACCACGAAGCGAGGTATCGGTCCAGCTTACTCCGGCAAAGCTTCACGGT
CAGGATTACGCGTTCACCACCTCTTCGACCACGAGACCTTTGCCGAGAAATTCCGAAA
GCTCGTAGAAGGCCGGTATAAGCGCTACGGCCACTTTGAGTACGATACAGAAGGAGAG
ATTGAACGATACAAGGTGATTCTGACCACCTACATATGCAAAGCCCGTCTGACATTTAC
AGAAACTCGCAGAACGACTGCGGCCATATGTCGTAGATAGTGTCGCCTACATCCACAA
AGCCATCACATCTGGGAAAAAGGTTCTTGTGGAAGGAGCCAACGCTTTGATGCTCGAC
CTTGACTTCGG
>Ade12 (FG673)_*Pluteus cervinus*
AGTATAGGTACAACGAAGAAAGGCATTGGTCCGGCTTACTCTGGCAAAGCTTCACGAT
CCGGCTTAAGGGTTCACCACCTTTTTGACCATGATACATTTGCGGAGAAGTTCAGGCG

GGTTGTAGAAGGACGCTTCAAGCGGTATGGCCATTTTGAGTATGATACCGAAGGAGAA
ATTGTCAGATACAAAGTAAGCACATACTTATGTCCTAGGTTTCTGTGGCGAACTTTCAA
AGGCATTGGCAGAGCGATTACGGCCGTACGTCGTTGATAGTGTGGCTTTCATCCACAAC
GCCATCGCGAATGGAAAGAAAGTCCTTGTCGAAGGAGCGAATGCTCTCATGCTGGACC
TTGATTATGG

>Ade12 (FG673)_*Gymnopilus chrysopellus*
AGCATCGGCACTACAAAGAAGGGTATTGGCCCGGCCTACTCTGGCAAAGCTTCCCGAT
CTGGGCTCAGGGTGCACCACCTCTTCGACCACGACACTTTTGCACAGAAATTCAGGAA
GCTGGTCGAAGGGAGGTTCAAACGCTACGGGCATTTTGAATACGACACTGAGGGTGA
GATCCAGCGATACAAGGTCAGTTTCTTCGCCTGCCTGTGTTTGATCGTCATGCCTGTATC
AGGAACTTGCTGAGCGACTACGCCCATATGTTATTGACAGCGTCGTATACATTCACCAA
GCCATTTCTTCAGGCAAGCGGGTCCTCGTCGAAGGTGCCAACGCCCTTATGCTCGACC
TCGACTTCGG

>Ade12 (FG673)_*Hygrophoropsis aurantiaca*
AGTATTGGAACGACGAAAAAAGGTATTGGCCCGGCTTACTCCGGCAAGGCATCTCGTT
CGGGACTTCGAGTCCATCATCTCTTTGATCATGATACCTTTGCTCAGAAGTTCCGCAAG
ATCGTCGAAGGTAGATTTAAGCGCTACGGACACTTCGAGTATGATACCGAAGGCGAAAT
AGAAAGATACAAGGTAAAGTCAGCGACTTTCATGTCACGTATTCTTATGATCTCGCTAG
GTTCTTGCACAACGCTTGAAACCGTTTGTTGTTGACAGCGTGGCGTATATCCATGGTTC
TCTCGCGGCACAGAAGAAGGTTCTCGTCGAAGGGGCCAATGCCCTCATGCTGGATTTA
GATTTTGG

>Ade12 (FG673)_*Serpula lacrymans* var. *lacrymans*
AGCATTGGTACCACGAAAAAGGGAATCGGTCCGGCCTACTCTGGAAAAGCATCGAGG
TCGGGTCTGCGGATACACCATCTTTTCGACCATGATACTTTTGCCCAAAAGTTCAGAAA
AATTGTAGAGGGCAGATTCAAACGCTATGGGCACTTTGAATACGACACCGAGGGAGAG
ATCGCTCGGTACAAGGTTGGTGACTGTCATTGCTATCATCGAAGTATAAACGTCATCGC
AGATTCTGGCAGATCGTTTGAGACCTTACGTTGTCGACAGTGTTGTCTATATCCATAAG
GCTCTGGCAGCAAAGAAGAAAGTTCTCGTTGAAGGGGCGAATGCACTCATGCTTGAC
TTGGATTTTGG

>Ade12 (FG673)_*Gymnopus androsaceus*
AGCATCGGTACCACAAAGAAGGGAATCGGTCCAGCTTACTCTGGCAAAGCTTCTCGCT
CGGGATTGCGCGTCCATCACCTTTTCGACCATGAAACGTTTGCCCAAAGGTTCCGCAA
ACTTGTAGAGGGCCGCTTCAAACGCTATGGCACTTTTGAATATGATACTGAAGGTGAAA
TCGAGCGGTACAAGGTATACAAGTCCCTTCTTACATCCTATATGAAACTTGGTACTTCAG
GCTCTAGCTGAGAGGTTACGCCCGTTTGTCATTGACAGCGTGGTATACATACACAAAGC
TCTATCCAGCGGAATGAAAGTCCTCGTCGAAGGGGCCAACGCACTCATGCTTGATCTT
GATTATGG
>Ade12 (FG673)_*Lactarius quietus*
AGCATTGGAACCACCAAGAAAGGCATCGGCCCCGCCTACTCCGCAAAAGCGTCGCGC
TCCGGCTTGCGCGTGCACCATCTCTTTGACCCCACGTTCGCGGAAAAGTTCCGTAAGA

TCGTCGAGGGTCGGTACAAGCGCTACGGGCATTTCGAGTACGACACGGAGGGGGAAA
TCGAGCGCTACAAGGTGCGGGGCTTCGAACGAGCGCCTGCTACGCTACCCGATCTTGC
AGGGATTGGCGGACAGGCTGCGCCCTTTCGTCATCGACAGCGTTGTGTATATACACGA
GGCACTCGCAACCGGAAAACGCATCCTCGTCGAAGGCGCAAATGCGCTGATGCTCGA
CATCGACTATGG

>Ade12 (FG673)_*Amanita muscaria*
AGCATCGGAACAACCAAGAAAGGAATTGGCCCAGCCTACTCTGGCAAAGCCTCCAGA
TCAGGCTTACGTGTCCACCATCTATTCGATCACGACACCTTTGCCGAAAAGTTTCGTAA
ACTCGTCGAAGGGAGGTTCAAACGATACGGATACTTTGACTATGACACAGAGGGCGAG
ATCGAGCGATATAAGGTCTGGGGTCGCTATTCCCTGCTTCATAGCCAAAAAACACATCC
AGACCCTCGCAACCAAACTAAAACCCTACGTGATCGACTCCGTATCATACATCCACAAC
GCCTTGTCGTCTGGTAAACGTATCCTAGTCGAAGGCGCCAACGCGCTGATGCTCGATAT
CGATTTCGG

>Ade12 (FG673)_*Mycena crocata*
AGTATCGGCACTACCAAGAAGGGAATCGGCCCAGCGTACTCGGGCAAGGCGTCCCGCT
CCGGCCTGCGTGTACATCATCTCTTCGATCCCGCCACATTTGCCGATAAGTTCCGCAAG
CTCGTCGAAGGCCGCTTTAAGCGCTATGGCCACTTTGAATACGATACAGAGGGGGAGA
TCAAACGTTATCAGGCAAGCCAGGCCCCTTTGGCTCCCGCAAACACAATGGGCTTCAC
AGGTCCTAGCTGCGCGACTCAAACCATACGTTATCGATAGCGTGGTTTACCTCCACCAG
GCCCTTGCCTTGGGCAAGCGCGTCCTTGTAGAAGGTGCCAACGCTGCGATGCTCGATC
TCGACATGGG

>Ade12 (FG673)_*Gautieria morchelliformis*
AGCATTGGGACAACAAAGAAGGGAATAGGTCCAGCGTATTCTGCCAAGGCTTCTCGCT
CGGGCCTTCGTGTCCACCACCTGTTCGATCACGATGTGTTCGCACAAAAGTTTCGAAA
GATCGTGGAAGGTCGTTTCAAGCGCTACGGTCACTTTGAGTATGACACAGAAGGAGAG
ATTTTGAGATATAAGGTATGCTCAAATCTGCCACGACGCTGTCATCCAGCGTCTCGTGT
AGGATCTAGCACAAAGACTGCAGCCATACGTCGTCGACAGTGTGACCTATCTCCACTC
TGCAATTGCTAGGGGCAAACAAGTCCTTGTAGAGGGTGCCAACGCTCTCATGCTTGATA
TTGACTATGG

>Ade12 (FG673)_*Ramaria acris*
AGCATTGGAACAACGAAGAAAGGAATAGGCCCAGCTTATTCGGCAAAAGCTTCCCGCT
CAGGTCTTCGCGTTCATCACTTATTCGACCACGACGTTTTTGCCCAGAAGTTTCGTAAA
ATTGTTGAAGGCCGTTTCAAACGCTACGGCCATTTCGAGTATGACACAGAAGGCGAAA
TTCTGAGATATAAGGTACGTCTATATCCTTGCCTACGACTCACAGCAGCGTCTTATGCAG
GAGCTTGCACTCCGACTACAACCGTACGTTATCGATAGTGTTACATATGTGCACGCCGC
CATCGCTACTGGCAAAAATATTCTCGTCGAGGGTGCCAATGCATTGATGCTTGATATTGA
TTATGG
>Ade12 (FG673)_*Stereum hirsutum*
AGTATCGGAACGACCAAGAAGGGTATCGGCCCCGCGTACTCTGCCAAGGCCTCCCGAT
CTGGCCTGCGCGTGCATCACCTTTTTGACCACAACACGTTCGCGGAGAAATTCCGGAA

GATAGTCGAGGGGAGGTTCAAGCGGTATGGGCATTTCGAGTACGATACCGAGGGGGAA
ATCGTGAGGTATAAGGTGCGTATCCATTCTTTCCTACGTCATCTCCTATTTGTGATTTTAG
GCTCTCGCCGAACGCCTCCGCCCCTACGTCGTCGACTCGGTAGTCTACCTCCACAAAG
CACTCGCGGAGAACAAACGCGTCCTCGTCGAAGGTGCCAACGCGCTCATGCTCGATCT
CGACTTCGG

>Ade12 (FG673)_*Tricholoma matsutake*
AGCATCGGAACAACTAAGAAAGGTATCGGCCCTGCCTACTCCGGGAAAGCCTCACGGT
CTGGCTTGAGGGTACACCATCTTTTCGACGATACCTTTGCGGTGAAGTTTAGAAAACTC
GTAGAAGGGCGGTTTAAGCGATACGGACATTTTGAATATGATACAGAGGGAGAGATAG
TGCGGTACAAGGTTTCTGAGTTCTTGAAGTGTTTGCTCCTGGCTTATTTGGTGATAGGC
TCTTGCCGATCGACTCCGCCCTTACGTGATAGACAGCCTGATCTACATCCACAATGCCA
TTTCCTCTGGGAAGCGTGTGCTGGTAGAGGGTGCAAACGCGCTTATGCTTGATATTGAC
TTTGG

>Ade12 (FG673)_*Fomitiporia mediterranea*
AGCATTGGTACGACGAAGAAGGGTATTGGCCCGGCGTACTCTGGCAAAGCTTCCCGCT
CGGGTTTGCGTGTGCACCATTTGTTCAATCATGATGCGTTTGCTGCCAAGTTCCGCAAG
ATCGTTGAAGGCCGATTCAAGCGATATGGGCATTTTGAATATGACACGGAAGGGGAGA
TCGTTCGGTACAAGGTAAACACCCCATTCATTGAATCTTATGGAGTTTTGACAACGACA
GAAACTCGCTGAACGTCTAAAGCCATACGTGCTCGACTCGCTTGCCTTCCTACACCTCC
AACTCACGCAAAACAAGCGCGTCCTAGTCGAAGGCGCGAACGCACTCATGCTAGACC
TGGACTTCGG

>Ade12 (FG673)_*Calocera cornea*
TCCATCGGCACGACGAGGAAGGGCATCGGCCCCGCGTACTCGAGCAAGGCGAGCCGC
AGCGGCCTGCGTGTGCACCACCTGCTCGCGAACGACAGCTTTGCCGACAAGTTTAGG
AAGCTCGTCGAGGGCCGGTTTAAGCGCTATGGCCACTTTGAGTACGACACCGAGGCGG
AGATTGCGCGGTACAAGGTGCGTGTACCCCTATCCCTATAGAGACTGGATTGCGCGCAT
ATAGGTCCTTGCCGAACGTCTCCGCCCATACGTGGTCGACGGCGTGACCTGGATCCAC
CAAGCGCTTTCGCAAGGCAAGCGCATCCTCGTCGAAGGCGCCAACGCGCTCATGCTCG
ACATCGATTTCGG

>Ade12 (FG673)_*Dacryopinax primogenitus*
TCAATCGGGACGACGAAGAAGGGGATCGGCCCCGCTTATTCGTCCAAGGCTAGCCGGA
GCGGGCTGCGTGTGCACCATTTGCTCGCGAATGACAGCTTTGCGGACAAGTTCAGGAA
ACTTGTTGAGGGGAGGTTCAAACGATATGGACACTTTGAATATGATACCGAGGCTGAA
ATCGCGCGGTACAAGGTATGCCAACGCGTTTTGTCCTGCACTCACTAGAGCACGCATGT
AGGTCCTTGCGGAACGTCTCCGGCCCTACGTTGTCGACGGCGTTACCTGGATCCACAA
AGCCCTTGCGGACGGGAAGCGTATCCTCGTTGAAGGGGCCAATGCCCTCATGCTGGAT
ATCGACTTTGG
>Ade12 (FG673)_*Agaricostilbum hyphaenes*
GCTGTCGGCACCACCAAGAAGGGTATCGGCCCAGCTTACTCTGGCAAGGCCTCGCGTT
CTGGCCTCAGAATCCACCATCTCTACGATGAGAAGCTCTTCGAGGAGAAGTTCAGGAC

CGTTGTCGAGAACCGCTTCAAGAGGTATGGCCACTTTGAGTACGACACCGAGGCAGA
GCTGAAGCGATATAAAGTGAGAGATGGCTACAAATACCTATTGAGATGACGTTTCTCTC
TAGGATCTGGCCAAGCGCCTCAAGCCTCATGTCGTGGACGGCTCCACCTGGCTCCACG
AGAAGCTCGAGCAGGGCAAGCCCGTCCTCGTCGAAGGCGCCAACGCTCTCATGCTCG
ACCTCGACTTTGG

>Ade12 (FG673)_*Rhizopus microsporus*
AGTATTGGCACTACCGGTAAAGGTATTGGTCCTACCTATTCCTCCAAGGCTTCTCGCTCT
GGTATCCGTGTTCACCACTTGTTCCAGTTTGAAGAATTTTCTGCTCGTTTCAGAAGCAT
GGTTGAAAACAAGCGTAAACGTTATGGTAACTTTGAATACGATGTTGAAGCCGAATTA
GAAAGATATAAGGAGTTGGCTGAACGTGTGAGGCCATACGTGATTGATACCATTCCTTA
TCTTCATGAACAAATCAAAGCTGGTAAGCGCATTTTGGTAGAAGGCGCCAACGCTTTG
ATGCTTGATATCGATTTTGG

>Ade12 (FG673)_*Asperigillus triticus*
AAGGTCGGTACCACGGGTAAGGGCATCGGTCCCTGCTACAGCGACAAGGCTGCCCGTC
GTGGCGTGCGTGTCGGCGAGATCTTGGATGAGGCGGTCTTTGAGCGCAAGCTGCGCA
ACCTGGATGCTGGGTACCGGGCGCGCTTTGGCGATCTGGAGTACAATGTGGAGGATGA
ACTTGCGTTGTTCAAGGTAACCCCCTAACCCCCTCTTTCTTGACGTCTGTTAATATTTGC
AGGACTTCCGTGCCCGTCTGGCCCCCCACATCGTCGACCAACTCGCCTTCCTCCAGGA
ATACAAAGACTCGCCCAACACCCTGGTCGAAGGCGCCAACGCGCTCATGCTCGACCTG
GACCACGG

>Ade12 (FG673)_*Neurospora crassa*
AAGATCGGTACCACCGGCCGTGGTATCGGTCCCTCTTACAGCACCAAGATGGCGAGAA
GCGGTGTCAGAATCCACGAGATCTTCAACGAGGAGATCTTTGAGCGCAAGCTCAGGC
AACTCGCTGCCGGCTACAAGAAGAGATTTGGTGACCTCAAGTACGATGTCGAGGAGG
AGATTGCCCGCTTCAAGGAGTACTGTCAAGCTCGCCCGCTATACTGTCGATGCCATCCA
GTACATGAAGGAGGCCCAGGACCGCGGCTACAAGATCCTCATCGAGGGTGCCAACGC
CCTCATGCTGGATATCGACTACGG

>Arc40 (FG771)_*Hygrophoropsis aurantiaca*
CCTAACTCCAACCGCATCGTCACTGCTTCTCAGGATCGTAATGCCTATGTGTGGCAGCA
GACCCTCGACCCGATCACTGGCCAGATGGCATGGAAACCCACATTGGTGTTGCTCAGA
ATCAATCGGGCTGCGACTTTTGTTCGATGGAGTCCTAATGAGGACAAATTCGCAGTAGC
TAGTGGTGCTAGGTGCGCTGTTATTGTTTTATGTAGGGTCCTACTGATGTAGCGGTTATA
GAGCAATCGCTATCTGTTCATTTGACTCCGAGAACAATTGGTGGGTTGCGAGGCAACT

>Arc40 (FG771)_*Marasmius fiardii*
CCTAATTCCAACAGAATTGTTACCGCATCTCAAGACAGAAATGCTTATGTTTGGCAGCA
AGCTCCTGATCCAGAAACCGGCAAGGTTATTTGGAAGCCGACGTTGGTGTTGTTGAGG
ATCAATAGGGCTGCTACGCATGTCCGATGGAGCCCGAACGAGGACAAATTTGCGGTTG
CGAGTGGTGCTAGGTGATTCCCTTTCGAATGACTTCTTGGGAAGCAGCTAAATATTACC
TAGAGCGATCGCTATTTGCTCTTTCGACCCAGAGAACAACTGGTGGGTCTCCAAATTGC

T

>Arc40 (FG771)_*Coprinopsis cinerea*
CCTAATTCCAACAGGATCGTCACCGCGTCTCAAGACCGAAATGCGTATGTCTGGCAAG
AGACTGCCGATCCCGAGACTGGCAAATTGGTGTGGAAACCTACACTCGTGTTGTTGCG
AATCAATCGCGCTGCGACGTATGTGCGATGGAGTCCCAAGGAGGACAAGTTTGCTGTT
GCCAGTGGTGCTAGGTGCGTTTGAAACCATCCCTTTCCTTGGCGGTCAACTGAATACC
ACCAGAGCTATCGCTATCTGTTCGTTTGACGCCGAGAACAATTGGTGGGTCGCGAAAC
AGCT

>Arc40 (FG771)_*Agaricus bisporus* var. *burnettii*
CCTGTATCTAACCGCATCGTCACCGCTTCTCAAGACAGGAATGCGTATGTTTGGACGGA
GACCACAGATGCAGAGACTGGCAAGTTGATTTGGAAGCCGACACTCGTATTGCTGAGA
ATCAATCGCGCTGCCACTCATGTTCGTTGGAGCCCCTTAGAAAATAAGTTCGCCGTTGC
CAGTGGTGCGAGGTGCGTATATATTTATCAATGTCTGGTATGCTCAAGCGAGGATATTCA
GAGCGATCGCTGTATGTTCTTTCGATGAAGAGAGCAATTGGTGGGTTGCCAAACACCT

>Arc40 (FG771)_*Schizophyllum commune*
CCCAACTCGAACCGCATCGTCACCGCGTCCCAGGACCGCAACGCCTACGTCTGGCAGG
AGACCCCCGACCCCGAAACAGGCCGCCTCGTCTGGAAGCCGACGCTCGTGCTCCTGC
GCATCAATCGCGCGGCGACGGTCGTGCGCTGGAGCCCCAAGGAGGACAAGTTCGCGG
TCGGCAGCGGCGCGCGCGCGATCGCGATCTGCTCCTTCGACCCCGAGAACAACTGGTG
GGTGTCGAAGCTGCT

>Arc40 (FG771)_*Auricularia delicata*
CCCAACTCGAACCGCATCGTCACCGCGTCGCAGGACCGCAACGCCTACGTCTGGACGC
AGACGCCCGACGCGTCCGGCAAGCTCATCTGGAAGCCCACGCTCGTGCTGCTGCGCAT
TAACCGCGCCGCGACCTATGTCCGCTGGAGCCCGAACGAAGATAAGTTCGCCGTCGCG
AGCGGCGCCAGGGCCATTGCCATCTGTTCGTTCGACGCCGATAACGACTGGTGGGTGT
CGCGCCTGCT

>Arc40 (FG771)_*Dacryopinax primogenitus*
CCTCACTCGAACCGCATCGTCACCTGCTCACAGGATCGAAATGCATATGTCTGGACACA
GGTGCAAGATGCACAGACCGGAGAGCTGATTTGGAAACCGACTCTTGTCTTGTTGCGC
ATCAACCGTGCGGCGACTTTCGTCAAGTGGAGTCCGAACGAGGACAAGTTCGCGGTT
GCGAGCGGTGCTCGGTAAGTGCTTCTCCCGCAACTAGCGGGAAGACGTTGAATTGGTT
GCAGCGCTATTGCGGTCTGTTCGTTCGATGAGGAAAACAACTGGTGGGTTTCCCGTCA
TTT

>Arc40 (FG771)_*Calocera cornea*
CCCAACTCGAACCGGATCGTCACCTGTTCGCAGGACCGCAATGCGTACGTGTGGACGC
AGGTGCCCGACCCGCAGACGGGCGAGCTCACTTGGAAGCCGACGCTCGTGCTGCTGC
GCATTAACCGCGCTGCTACGTTCGTCCGGTGGAGCCCGAATGAGGACAAGTTCGCGGT
TGCGAGCGGTGCTCGGTACGTTGCTGCGCCGCTTAAGGTTGCAGGGGCTAACTATCGG

TAGTGCCATCGCCATCTGCTCCTTCGACGAGGAGAACAACTGGTGGGTCTCTCGCCTG
CT

>Arc40 (FG771)_*Mycena crocata*
CCCAACTCCAACCGCATCGTTACGGCCTCGCAGGACAGGAATGCCTACGTTTGGCAGC
AGACCCCGGATCCCGAGACGGGCAAACTCATCTGGAAGCCGACCCTCGTCTTGCTGA
GAATCAACAGGGCGGCGACCCATGTCAGGTGGAGTCCCAAGGAAGACAAATTCGCAG
TGGCTAGCGGTGCGCGGTGAGCCTCATCGTATTCTTCTTGGTTCTCCTCCAGTGCGATC
GCCATCTGCTCCTTTGACCCCGAAAACAACTGGTGGGTCTCGAAGCTCCT

>Arc40 (FG771)_*Stereum hirsutum*
CCTAACTCCAACCGCATCGTGACCGCTTCTCAGGATCGCAACGCATACGTGTGGCAAC
AGACCCCCGACCCTCAGACCGGTCGTACCATCTGGAAGCCCACCCTCGTCCTACTGCG
GATCAATCGCGCCGCAACGTTCGTTCGGTGGAGCCCCAATGAAGACAAGTTCGCCGTT
GCGAGCGGTGCTAGGTACGTGCATGTTCGTGGTGGTTTTGCCAGGTGCTGAAGCAACT
CTTAGGGCTATCGCCATATGCTCGTTCGATCCGGACAACGATTGGTGGGTTTCGAGGCT
GCT

>Arc40 (FG771)_*Phanerochaete carnosa*
CCGAATTCCAATCGTATCGTCACTTCCGCCCAGGATAGAAACGCATATGTCTGGCAGCA
GACTCCTGACCCACAGACCGGACGCTCGATCTGGAAGCCTACGCTTGTGCTTCTCCGC
ATCAACCGCGCTGCGACGTATGTGAAGTGGAGTCCCAACGAGGATAAGTTTGCTGTGG
CCAGTGGTGCTCGGTGCGTACCCCTGGAGGCACAAGAGGCACACGAGCTAACAGGCC
TGTAGTGCTATCGCCATTTGCTCGTTTGATCCCGAGGGCGACTGGTGGGTTTCCCGTCT
CTT

>Arc40 (FG771)_*Trametes versicolor*
CCGAAGTCCAACCGCATCGTCACGTCCGCTCAGGATCGGAACGCGTACGTCTGGCAGC
AGAGCCCTGACCCGCAGACGGGGCATATGATTTGGAAGCCGACGCTCGTCTTGCTGCG
CGTGAACCGCGCAGCGACGTACGTGCGATGGAGCCCGAACGAGGACAAATTTGCGGT
GGCGAGCGGCGCTCGGTATGTCTCCTCGCCCTCGTATTTTATGCAGCTCCTAACCGCTG
CGCAGTGCGATTGCCATCTGCTCCTTTGATCCCGAGGGTGACTGGTGGGTGTCACGCC
TGCT

>Arc40 (FG771)_*Gymnopus androsaceus*
CCCAACTCGAACCAAATCGTAACGGCTTCTCAAGATCGGAATGCATACGTTTGGCAGC
AGACTCCCGATCCAGTGACCGGAAAGACTATCTGGAAACCAACCTTGGTTCTCCTTAG
GATCAACAGAGCGGCGACCCATGTACGATGGAGTCCTAAGGAAGACAAATTCGCTGTA
GCTAGTGGTGCTCGGTGCGTCTTCAACAATCTGCTGGTTTCACCGATATCATTTATTCTG
TAGTGCTATCGCTATATGTTCCTTCGATCCCGAAAACAACTGGTGGGTATCCAAATTGCT

>Arc40 (FG771)_*Laccaria bicolor*
CCGAATTCTAACCGCATCGTGACTTGTTCTCAAGATCGCAATGCGTACGTTTGGCAGGA
GACACCCGACCCGGATACGGGAAAACTTGTGTGGAAGCCAACCCTCGTTCTGCTCAG

AATTAACAGAGCAGCGACCCATGTCAAATGGAGCCCTCTGGAGGATAAATTCGCTGTT
GCGAGTGGCGCACGGTATGACGTCTTTTCTATCGAGACTAAGGGATTCACTTAACTACC
AGTGCGATAGCTATCTGCTCGTTCGATCCCGAGAACAACTGGTGGGTATCAAAGCTGTT

>Arc40 (FG771)_*Agrocybe pediades*
CCCAGCTCCAACCGTATTGTCACTTGCTCGCAGGACAGGAACGCATATGTCTGGCAAG
AGCAGCCAGACCCTGAGACGGGCAGGCTTGTCTGGAAGCCCACTCTTGTGTTGCTCA
GGATCAATAGGGCGGCGACTTATGTCAAGTGGAGCCCTAGGGAGGACAAGTTCGCTGT
GGCCAGCGGTGCTCGGTATGTTGCGTTTCTATGATAGATGGATTTGTTCTTTTTCGTGTA
ACAGAGCTATCGCCATTTGTTCGTTCGATCCCGAGAACAACTGGTGGGTCTCCAAGTT
GCT

>Arc40 (FG771)_*Gymnopilus chrysopellus*
CCTAATTCCAATCGTATCGTCACTTGCTCTCAAGACCGCAATGCATATGTATGGCAAGA
ACAGCCTGACCTGGAGACCGGCAAATTGATCTGGAAGCCGACTCTTGTGTTGCTGAGG
ATCAATAGGGCGGCGACTCATGTCAGATGGAGTCCCAAGGAGGATAAGTTTGCTGTAG
CCAGCGGCGCCAGGTGCGAAAACGATACAGCCCCTGACAGATGGCACTAACCTTTCCT
AAAGGGCTATCGCGATCTGTTCCTTTGATCCAGAAAACAACTGGTGGGTATCGAAATTG
CT

>Arc40 (FG771)_*Tricholoma matsutake*
CCAAATTCCAATCGCATTGTTACTGCGTCTCAAGATAGGAACGCATATGTCTGGCAAGA
GGCGCTAGATTCAGAGACGGGCAAAATTTCGTGGAAACCGACCCTCGTGCTACTAAGG
ATCAACAGAGCTGCCACTCATGTCAGATGGAGCCCCATGGAGGACAAGTTTGCTGTAG
CGAGTGGGGCGCGGTAATTATCATGCATATCATATCATTGAGGATACTGAACAAGAAAC
AGTGCGATCGCAATCTGTTCTTTCGACTCGGAAAATAATTGGTGGGTGTCAAAGCTCCT

>Arc40 (FG771)_*Gautieria morchelliformis*
CCAAATTCGAACCGCATTGTGACCGCCTCCCAGGACAGGAATGCCTACGTTTGGCAGC
AGGCCCCGGATCCGTCCACAGGCCAGACCATCTGGAAACCCACCCTCGTCTTACTTAG
GATCAACAGGGCCGCAACCTTCGTGAGGTGGAGTCCACTGGAAGACAAGTTTGCCGT
TGCCAGCGGTGCTCGGTGCATATTATTATACAGAACCCACAGTAATCCTTCTGATTCTTA
ATAGTGCCATCGCTATCTGCTCCTTTGACCCAGAGAATGATTGGTGGGTCTCAAGGCTG
CT

>Arc40 (FG771)_*Ramaria acris*
CCAAATTCCAATCGCATCGTCACCGCTTCTCAGGATAGGAATGCTTATGTCTGGCAACA
GACTCCTGACCCCTCTACTGGACAAACTATCTGGAAACCAACGCTGGTTTTGCTGAGG
ATAAACAGGGCGGCGACTGCTGTCAGATGGAGTCCACTAGAAGATAAGTTTGCCGTTG
CCAGCGGTGCTCGGTATGTATTCTTTATTCAAAACGTCGTATTTATTTGACTATGGCAGC
GCCATTGCCATTTGTTCCTTCGACCCAGAGAATGACTGGTGGGTGTCGAGACTGTT
>Arc40 (FG771)_*Boletus edulis*
CCGAACTCAAACCGCATCGTGACAGCATCGCAGGATCGCAACGCCTACGTTTGGTCCC
AATCGCCGGATCCACTCACGGGAAAGATGATGTGGAAGCCGACGCTCGTGCTGCTGCG

TATCAACAGAGCGGCCACGTTCGTTCGATGGAGCCCAAACGAAGATAAATTCGCAGTG
GCGAGTGGTGCCAGGTACGATTTACCATCGTGGGTGTCTAGAGGTCTGCTGACGTCAT
GGTAGAGCCATCGCCGTCTGTTCGTTCGACGCCGAGAACAATTGGTGGGTCGCAAAAC
AGTT

>Arc40 (FG771)_*Pluteus cervinus*
TCTTCTTCCAATCGCATCGTCACTGCATCGCAAGATCGTAACGCCTATGTCTGGCAGCA
GACAGCGGATGCCGACGGCAAGATAGTTTGGAAGCCAACCCTCGTTTTGCTTCGAATT
AACAGAGCAGCGACCCATGTTCGCTGGAGTCCCAAGGAAGACAAATTCGCAGTAGCT
AGTGGTGCCAGGTTCGTGTTTTTTGCCCCCTGGGATGTACCACCTCCTCATCAACAGGG
CCATTGCCGTCTGCTCGTTCGATCCTGAGAATAACTGGTGGGTTTCAAAACTTCT

>Arc40 (FG771)_*Fomitiporia mediterranea*
CCCAACTCTAACCGTATCGTAACTGCATCGCAAGACAGAAATGCATATGTGTGGACCCA
AATCCCCGATCCTCAGACGGGACATCTGATTTGGAAGCCGACGCTTGTGCTCCTGCGTA
TCAACCGTGCTGCAACTTACGTTCGGTGGAGCCCCTATGAAGATAAGTTCGCCGTCGC
AAGCGGTGCTCGGTAAGATACTCGACATTCCACCTGTCCTTGTCTCATGGTTCTTACAT
AGAGCTATTGCTGTCTGTTCTTTCGATCCAGAAAGTGACTGGTGGGTCTCTAAGCAACT

>Arc40 (FG771)_*Serpula lacrymans* var. *lacrymans*
CCGAACTCGAACCGTATCGTTACAGCATCGCAAGATCGCAATGCATATGTGTGGCAGCA
GAGCCCAGATCCTCAAACCGGTAAAATGGTCTGGAAGCCGACCTTGGTGCTGTTGCGG
ATTAACAGGGCTGCTACGTTCGTCCGTTGGAGTCCGAACGAAGATAAGTTTGCTGTTG
CCAGTGGTGCTCGGTAAGCTATTGTTTCTTAGTTTTGAGCGCAAGGTTATATTCCTCATT
AGGGCGATTGCAGTCTGCTCTTTTGATTCCGAGAATAACTGGTGGGTTGCTAGGCTCTT

>Arc40 (FG771)_*Amanita muscaria*
CCGACTACGAATCGCATCGTCACGGCATCTCAAGATAGGAATGCTTATGTATGGCAAGA
AACTCCGGATCCAGAGACAGGAAAGTTGATCTGGAAGCCAACTCTTGTTGTACTACGA
ATCAATCGCGCCGCTACTTATGTCCGGTGGAGTCCGAAGGAAGACAAGTTTGCAGTGG
CCAGTGGAGCACGGTAATGACGGTTAATACAACTGTGCGCATTATTGACTCTGACTTAG
GGCTATCTGTGTCTGCTCGTTTGATTCGGAAAGCAACTGGTGGGTTTCTAAGCAGCT

>Arc40 (FG771)_*Agaricostilbum hyphaenes*
CCTCGCACCAACCGCATCGTCACTTGCTCCCAAGACCGCAACGCTTACGTTTGGACGG
CACAAGACCAGCAGAACTCTACTTGGAAGCCTACGCTCGTCCTTTTGCGTCTCAATAG
ATCAGCTACTTGCGTCAAATGGTCGCCTACTGAATCCAAGTTCGCCGTAGGCTCTGGAG
CCAGGGCGATTGCCATCTGCACCTTTGATGAGGAGAGCGACTGGTGGGTTGCCAAGCA
CAT

>Arc40 (FG771)_*Rhizopus microsporus*
CCTAAAACGAATCGTATCGTTACCTGTTCACAAGATAGAAACGCTTACGTCTGGACGAA
TGAAGGAGGTGTTTGGAAGCCAGGACTTGTCTTGCTACGTATCAATCGTGCAGCTACTT

TCGTTCGTTGGTCACCCGATGAACAAAAGTTTGCTGTTGCCAGCGGTGCCCGCTGTATT
TCCGTCTGTTATTTTGAAGGAGATAATGATTGGTGGGCTAGTAAACACTT

>Arc40 (FG771)_*Neurospora crassa*
CCCAACACCGGGCGTATTGTGACCTGCTCTCAAGACCGCAACGCCCTCGTCTGGGAAC
CCTCCCCCACCGGCTACAAGCCCACCCTCGTGCTCCTCCGTATTTCCCGCGCCGCCACC
TTCGTCCGCTGGTCTCCCTCGGAAGCCAAGTTCGCCGTCGGCTCCGGCGACCGCGTCA
TCGCCATCTGCTACTTTGAGGAGGAAAACGACTGGTGGGTATCCAAGCACCT

>Arc40 (FG771)_*Asperigillus triticus*
CCTAACAGTGGCCGCATTGTCACCTGCTCTCAAGATCGCAACGCATACGTCTGGGAGC
GAACCCCCACTGGCTGGAAGCCTACCTTGGTTCTCCTGCGGATCAACAGGGCTGCTAC
CTTTGTGCGATGGTCTCCGTCGGAGCAGAAGTTTGCGGTTGGATCAGGGGCTCGCGTG
ATCGCCGTCTGCTACTTTGAGGAGGAGAACGACTGGTGGATCTCGAAGCATCT

>Atp2 (FG459)_*Gymnopilus chrysopellus*
GGTACCGAAGGTCTCGTACGTGGCACCAAGGTTGTTGACACCGGTGCCCCCATCATGG
TTCCCGTCGGAACTGCTACCCTCGGCCGTATCATGAACGTCATTGGTGAGCCCATTGAC
GAGCGTGGCCCCATCAAGGGTGTCAAGCTCTCCCCCATCCACGCCGACCCCCCGGCCT
TCGTTGACCAGTCCACCACTGCTGAGGTGTTGGAGACTGGTATCAAGGTCGTCGACCT
TCTTGCTCCCTACGCTCGTGGTGGAAAGATTGGTCTCTTCGGTGGTGCCGGTGTCGGA
AAGACTGTGTTG

>Atp2 (FG459)_*Coprinopsis cinerea*
GGTACTGAGGGTCTCGTCCGTGGTGCCAAGGTCGTCGACACCGGTGCTCCCATCATGG
TCCCTGTCGGCAAGGGCACTCTCGGTCGTATCATCAACGTCATTGGCGAGCCCATTGAC
GAGCGTGGCCCCATCAAGGGCGAGAAGATGTTGCCCATCCACGCCGACCCCCCTGCTT
TCGTCGACCAGTCCACGACCGCTGAAGTTTTGGAGACCGGTATCAAGGTCGTCGATCT
CCTCGCTCCTTACGCTCGTGGTGGAAAGATTGGTCTCTTCGGTGGTGCTGGTGTCGGA
AAGACTGTGCTC

>Atp2 (FG459)_*Hygrophoropsis aurantiaca*
GGTACCGAAGGTCTCGTTCGTGGACAAAAGGTTGTCGATACTGGTGCCCCCATCCAAA
TCCCCGTCGGTGTTGCTACTCTCGGCCGTATTATGAACGTCATTGGCGAGCCTATTGAC
GAGCGTGGTCCTATCAAGGGTGTCAAACTCTGTCCCATTCACGCTGACCCCCCGCCATT
TGTTGACCAGTCGACAACTGCCGAGGTTTTGGAGACCGGTATCAAGGTCGTCGACTTG
CTTGCTCCCTACGCCCGTGGTGGAAAAATTGGTCTTTTCGGAGGTGCTGGTGTCGGCA
AGACTGTGTTG

>Atp2 (FG459)_*Trametes versicolor*
GGTACCGAGGGTCTCGTCCGTGGGCAGAAGGTTGTCGACACGGGTTCGCCCATTCGGA
TCCCCGTCGGCGCTGCTACCCTTGGCCGTATCATGAACGTCATTGGCGAGCCCATTGAC
GAGCGTGGTCCCATCAACGGTGTCAAGCTCTCCCCCATTCACGCGGAGCCCCCTCCCT
TCGTCGACCAGTCGACGACTGCCGAGGTCCTGGAGACGGGTATCAAGGTTGTCGACCT

GCTCGCCCCCTACGCTCGTGGTGGAAAGATCGGTCTCTTCGGAGGTGCTGGTGTCGGC
AAGACTGTGTTG

>Atp2 (FG459)_*Fomitopsis pinicola*
GGTACCGAGGGTCTCGTCCGTGGGCAGAAGGTAGTCGACACGGGTTCACCTATCAAGA
TCCCCGTTGGCACTGCGACTCTTGGCCGTATCATGAACGTCATTGGCGAGCCCATCGAC
GAGCGGGGTCCCATCCAAGGTGTCAAGCTGTCCCCCATCCACGCTGACCCTCCGGCTT
TCGTGGAGCAGTCCACGACTGCTGAGGTCTTGGAGACCGGTATCAAGGTCGTCGACCT
GCTTGCTCCCTACGCTCGTGGTGGAAAGATTGGTCTTTTCGGCGGTGCCGGTGTCGGC
AAGACTGTGTTG

>Atp2 (FG459)_*Schizophyllum commune*
GGTACCGAGGGTCTCGTCCGTGGGCAGAAGGTCGTCGACACCGGTGCCCCCATCCAGA
TTCCTGTCGGCAAGGCCACCCTCGGCCGTATCATGAACGTCATCGGCGAGCCCATTGA
CGAGCGTGGTCCCATCAAGGGCGACAAGCTTCTCCCCATCCACGCCGACCCGCCCGCG
TTCGTCGACCAGTCGACCACCGCTGAGGTCCTGGAGACCGGTATCAAGGTCGTCGACC
TCCTCGCCCCGTACGCCCGTGGTGGCAAGATCGGTCTCTTCGGAGGTGCCGGTGTCGG
CAAGACGGTGTTG

>Atp2 (FG459)_*Phanerochaete carnosa*
GGTACCGAGGGTCTCGTCCGTGGTCAGAAGGTCGTCGATACCGGTGCTCCCATTCAGG
TCCCTGTTGGCAAGGGCACTCTCGGCCGTATCATGAACGTCATTGGTGAGCCCATTGAT
GAGCGTGGTCCAATCAAGGGCGACAAAGTCCTCCCCATTCACAATGACCCGCCTCCAT
TCGTCGAGCAGTCGACCACCGCTGAGGTCCTCGAAACCGGTATCAAGGTCGTTGACCT
TCTCGCCCCCTATGCCCGTGGTGGAAAGATTGGTCTCTTCGGTGGTGCCGGTGTCGGC
AAGACTGTACTT

>Atp2 (FG459)_*Auricularia delicata*
GGTACCGAGGGTCTCGTACGTGGCCAGAAGGTCGTCGACACCGGCGCTCCCATCAAGA
TCCCCGTCGGCCGCGGCACCCTTGGGCGTATCATGAACGTCATTGGCGAGCCCATCGA
CGAGAGGGGCCCCATCAAGGCCGAAAAGTTCCTCCCCATCCACGCGGACCCCCCGGC
CTTCGTCGACCAGTCTACAACGGCTGAGGTCCTCGAGACCGGCATTAAGGTCGTCGAC
CTCCTCGCCCCGTACGCTCGTGGTGGCAAGATCGGTCTCTTCGGTGGCGCCGGTGTCG
GCAAGACTGTGCTC

>Atp2 (FG459)_*Mycena crocata*
AGTACGGAGGGTCTCGTGCGCGGCCAAAAAGTCGTCGACACCGGTGCTCCCATTCTCG
TCCCTGTCGGCTCTGCTACCCTTGGTCGTATCATGAACGTCATTGGTGAGCCTATCGAC
GAGCGTGGACCCATCAAGGGCGTCAAGCTCAGCCCCATCCACGCCGACCCCCCGCCAT
TCGTCGACCAGTCTACCACCGCCGAGGTGCTTGAGACCGGTATTAAGGTTGTCGACCT
CCTCGCTCCCTACGCTCGTGGTGGAAAGATTGGTCTGCTTGGTGGCGCCGGTGTCGGG
AAGACCGTGTTG

>Atp2 (FG459)_*Laccaria bicolor*

GGTACCGAGGGTCTCGTCCGTGGAACAAAAGTGGTTGATACCGGCTCCCCCATCATGG
TTCCAGTTGGCACGGCTACGCTCGGACGTATCATGAACGTCATTGGAGAACCCATTGAT
GAGCGTGGCCCTATCAAGGGCGTCAAACTCTGCCCCATTCACGCTGACCCTCCCCCCT
TCGTCGACCAGTCGACGACTGCTGAGGTTTTGGAAACTGGTATCAAGGTCGTTGATCT
CCTCGCTCCTTATGCTCGTGGTGGAAAGATTGGTCTGTTTGGAGGTGCCGGTGTAGGA
AAGACTGTGTTG

>Atp2 (FG459)_*Agrocybe pediades*
GGTACAGAGGGTCTTGTTCGTGGAACCAAGGTTGTCGACACCGGCAACCCCATCATGG
TTCCCGTCGGAACTGCTACTCTCGGACGTATCATGAACGTCATCGGTGAGCCCATTGAT
GAGCGTGGTCCTATCAAGGGTGTTAAGCTCTCCCCCATCCACGCTGAGCCTCCTCCCTT
CGTCGACCAGTCCACTACCGCTGAGGTCCTCGAAACCGGTATTAAGGTCGTCGATCTC
CTCGCTCCTTACGCTCGTGGTGGAAAGATTGGTCTCTTCGGAGGTGCCGGTGTCGGAA
AGACTGTGTTG

>Atp2 (FG459)_*Agaricus bisporus* var. *burnettii*
GGTACCGAGGGTCTTGTCCGTGGTACAAAGGTCATTGATACCGGTGCCCCCATCCAAAT
TCCCGTCGGCAAGGCCACTTTGGGTCGCATTATGAACGTTATTGGTGAGCCCATTGACG
AACGTGGCCCCATCAAGGGTGTCAAGCTCTCTCCGATTCATGCCGAGCCTCCCGCTTTC
GTCGACCAAGCAACGACTGCCGAGGTCTTGGAGACTGGTATCAAGGTCGTCGATCTCC
TTGCTCCCTATGCTCGTGGTGGCAAGATTGGTCTCTTCGGCGGTGCTGGTGTCGGCAAA
ACTGTCTTG

>Atp2 (FG459)_*Stereum hirsutum*
GGTACCGAGGGTCTCGTCCGTGGCCAAAAGGTCATCGACACTGGTGCTCCTATCCAGA
TTCCCGTTGGCCGTGACACTCTTGGTCGTATCATGAACGTCATTGGTGAGCCTATCGAC
GAGAGGGGTCCTATCAAGGGTGTTACCCGCAAGCCCATCCACGCCGACCCTCCTGCTT
TCGTCGACCAGTCCACGACCGCTGAGGTGCTCGAGACCGGTATCAAGGTCGTCGATCT
TCTTGCCCCCTACGCCCGTGGTGGAAAGATTGGTCTCTTCGGAGGTGCTGGTGTCGGC
AAGACCGTGTTG

>Atp2 (FG459)_*Gymnopus androsaceus*
GGTACAGAGGGTCTCGTTCGTGGGCAAAAGGTTGTTGACACTGGTTCTCCCATTCTCG
TTCCCGTTGGAACTGGCACTCTCGGTCGTATCATGAACGTCATTGGTGAACCCATTGAC
GAGCGTGGTCCCATTAAGGGTGTTAAGCTCTGCCCCATCCATGCTGACCCTCCGCCATT
CGTCGAGCAATCCACGACTGCCGAGGTTCTTGAAACCGGTATCAAGGTTGTCGACTTG
CTTGCTCCCTACGCTCGTGGTGGAAAGATTGGTCTTTTCGGAGGTGCTGGTGTCGGAA
AGACTGTCTTG

>Atp2 (FG459)_*Lactarius quietus*
GGTACCGAAGGTCTTGTTCGTGGTCAAAAGGTCGTGGACACAGGGTCCCCCATTCAAA
TTCCCGTTGGCCGCGATACTCTCGGCCGCATTATGAACGTCATTGGCGAACCCATCGAC
GAGCGCGGTCCTATCAAGGGTGTTGTTCGCATGCCGATCCATGCCGACCCTCCTGCCTT
CGTAGAGCAGTCGACGACTGCTGAAGTGCTTGAGACCGGTATCAAAGTGGTTGACCTC

CTTGCTCCTTACGCTCGTGGTGGCAAGATTGGCCTTTTCGGTGGTGCTGGTGTCGGCAA
GACGGTTTTA

>Atp2 (FG459)_*Pluteus cervinus*
GGTACGGAAGGTTTGGTCCGTGGAACCAAGGTTGTGGACACTGGTTCTCCCATCCAGG
TCCCCGTCGGCAAGGCCACTCTCGGTCGTATCATGAATGTCATTGGTGAACCCATTGAC
GAGCGCGGTCCTATCATTGGTGTCAAGCTCTCTCCCATCCACGCTGACCCCCCAGCATT
CGTCGAGCAGTCCACTACTGCTGAGGTCCTTGAAACCGGTATCAAGGTTGTCGACTTG
CTTGCTCCCTACGCTCGTGGTGGAAAGATTGGTCTCTTCGGTGGTGCCGGTGTCGGAA
AGACTGTGTTG

>Atp2 (FG459)_*Serpula lacrymans* var. *lacrymans*
GGTACAGAAGGTCTCGTTCGTGGCCAAAAAGTGATTGATACCGGTGCACCCATTCAAA
TTCCTGTTGGCGTTGCTACTTTGGGCCGTATCATGAACGTTATTGGTGAGCCAATCGAC
GAACGTGGGCCTATCGTTGGTACCAAGTTGTCACCTATTCACGCCGACCCTCCTCCTTT
CGTTGACCAATCGACGACTGCTGAGGTATTGGAGACCGGTATCAAGGTTGTGGACTTG
CTCGCTCCTTACGCTCGTGGTGGAAAGATCGGTCTTTTCGGTGGTGCTGGTGTCGGCA
AGACTGTGTTA

>Atp2 (FG459)_*Boletus edulis*
GGTACGGAGGGTCTCGTCCGTGGACAGAAGGTCGTAGACACTGGTGCTCCCATTCAAA
TCCCTGTTGGCACTGCAACTCTGGGCCGAATCATGAATGTCATCGGAGAACCCATTGA
CGAACGTGGACCCATCAAGGGTGTCAAGCTTTGCCCCATTCATGCTGACCCACCCCCA
TTCGTTGACCAATCGACTACTGCTGAAGTCCTCGAAACGGGTATCAAAGTTGTCGACC
TTTTGGCTCCCTATGCACGTGGTGGAAAGATTGGTCTTTTCGGCGGTGCTGGTGTCGGC
AAAACTGTATTG

>Atp2 (FG459)_*Amanita muscaria*
GGTACAGAAGGTCTTGTCCGTGGTGCCAAAGTCGTTGACACTGGTGCTCCCATCATGG
TCCCCGTCGGAAAGGGTACACTTGGACGGATCATGAACGTCATTGGCGAGCCTATTGA
CGAACGTGGACCTATCAAGGGCACTAAGCTCAGCCCCATCCATGCTGACCCGCCTGCA
TTCGTGGAGCAATCCACGACTGCTGAGGTCCTTGAAACTGGTATCAAAGTCGTCGACC
TCTTAGCTCCCTACGCTCGTGGTGGTAAAATTGGTCTCTTCGGTGGTGCGGGTGTCGGC
AAGACCGTGTTG

>Atp2 (FG459)_*Fomitiporia mediterranea*
GGTACCGAAGGTCTTGTTCGCGGTCAGAAGGTCGTTGACACCGGAGCTCCCATCCGTA
TTCCTGTTGGAAAAGAGACCCTTGGCCGTATTATGAACGTCATCGGCGAACCTATTGAC
GAGCGTGGTCCGATCAAAGGCGTGAAATTGTCACCCATCCACGCGGAGCCGCCGCCAT
TCGTTGACCAGTCAACGACTGCTGAGGTACTTGAAACGGGTATCAAGGTTGTTGATCT
TCTAGCACCGTATGCCCGTGGTGGTAAGATTGGTCTTTTCGGTGGTGCTGGTGTCGGCA
AGACTGTGCTT

>Atp2 (FG459)_*Marasmius fiardii*

GGTACCGAGGGTTTGGTCCGAGGACAGAAAGTTGTTGACACTGGTTCTCCTATCATGG
TTCCTGTTGGGTCGGGTACTCTTGGACGTATCATGAACGTCATTGGTGAGCCCATTGAC
GAACGTGGTCCCATCAAGGGTGTGAAGCTCAGTCCCATTCACGCCGACCCTCCTCCAT
TCGTGGAGCAGTCTACCACCGCTGAAGTTCTTGAGACCGGCATCAAGGTTGTTGACTT
GCTCGCTCCTTACGCGCGAGGTGGAAAGATCGGTCTGTTTGGGGGTGCTGGTGTCGGA
AAGACCGTGTTG

>Atp2 (FG459)_*Tricholoma matsutake*
GGTACAGAAGGTCTCGTCCGAGGTACAAAGGTTGTGGACACCGGTGCTCCCATCATGG
TGCCCGTCGGCACTGCAACCCTCGGAAGAATCATGAACGTCATTGGCGAACCCATCGA
CGAACGTGGTCCCATCAAGGGTGTCAAACTTTCTCCAATTCACACGGACCCGCCTCCA
TTCGTTGACCAGTCTACCACCGCCGAGGTCTTGGAGACCGGTATCAAGGTTGTCGACC
TTCTGGCGCCTTATGCTCGTGGTGGAAAGATTGGTTTGTTCGGAGGTGCCGGTGTCGG
CAAGACCGTGTTA

>Atp2 (FG459)_*Ramaria acris*
GGTACTGAAGGTCTTGTCCGCGGCCAGAAGGTCATCGACACCGGGGCTCCAATTATGA
TTCCTGTTGGCAGTGGTACCCTCGGTCGTATTATGAATGTTATTGGTGAACCCATCGACG
AACGTGGTCCTATTAAAGGCGTCAAGAAATCTCCCATTCACGCCGATCCTCCGCCATTC
GTTGACCAGTCAACGACCGCGGAAGTGCTTGAGACTGGTATCAAGGTTGTGGACCTTC
TTGCTCCCTATGCTCGTGGTGGCAAAATCGGCTTGTTCGGAGGAGCTGGTGTCGGAAA
GACTGTGCTG

>Atp2 (FG459)_*Gautieria morchelliformis*
GGTACCGAAGGTCTCGTGCGGGGCCAGAAGGTCATCGACACCGGCGCGCCCATCATGA
TTCCTGTCGGCAGCGGGACACTTGGGCGCATCATGAATGTCATTGGAGAGCCCATTGA
CGAACGTGGACCCATCAAGGGAGTCAAAAAGTCCCCGATTCACGCCGACCCCCCGCC
CTTTGTCGACCAATCTACCACCGCCGAAGTGCTCGAGACGGGTATCAAGGTCGTGGAT
CTCCTGGCTCCGTATGCCCGTGGTGGTAAAATTGGTCTATTCGGAGGTGCCGGTGTCGG
CAAGACAGTACTC

>Atp2 (FG459)_*Calocera cornea*
GGAACGGAAGGCTTGGTTCGCGGTCAGAAGGTTGCGGATACCGGTTCGCCGATTAGG
GTTCCTGTTGGCAAGGGCACGCTTGGTCGCATCATGAACGTCATCGGTGAACCCATCG
ACGAACGCGGTCCTATTAAGGGAGACAAGCTCAACCCCATCCACGCGGAACCGCCTG
CGTTCGTGGAGCAGTCGACCACCGCCGAGGTCCTTGAGACCGGGATCAAGGTCGTGG
ACCTCCTGGCGCCTTACGCTCGTGGTGGGAAAATCGGTCTCTTCGGCGGTGCTGGTGT
TGGCAAGACCGTTCTC

>Atp2 (FG459)_*Dacryopinax primogenitus*
GGTACCGAAGGTTTGGTTCGTGGTCAGAAAGTTGCTGATACCGGCGCCCCGATTCGAA
TTCCCGTTGGCAAGGGAACCCTCGGCCGCATCATGAACGTCATTGGGGAACCAATTGA
TGAACGTGGTCCCATCAAGGGTGACAGGTTGAATCCCATCCACTCTGAGCCCCCGGCA
TTTGTCGATCAGTCGACCACCGCTGAAGTCCTTGAGACTGGAATCAAAGTTGTAGACC

TCCTTGCTCCTTACGCTCGCGGCGGGAAAATCGGCTTATTCGGTGGCGCTGGTGTAGGA
AAGACGGTGCTT

>Atp2 (FG459)_*Rhizopus microsporus*
GGTACTGAAGGTCTCGTCCGTGGTCAAAAGGTTGTTGACACTGGTGCTCCTATCACCA
TTCCTGTCGGTAAGGAAGTCCTCGGTCGTATCATCAACGTTATTGGTGAACCCATTGAT
GAACGTGGTCCCATCAACTCCAAGGCTCAACGTCCCATTCACGCTGATGCTCCCGAAT
TCGTTGACCAATCCCCCACTCCCGAAATTCTTGAAACTGGTATCAAGGTTGTCGACTTG
TTGGCTCCTTATGCTCGTGGTGGTAAGATTGGTCTTTTCGGTGGTGCTGGTGTCGGTAA
GACTGTGTTG

>Atp2 (FG459)_*Asperigillus triticus*
GGTACTGAGGGTCTGACCCGTGGTGCTGCCGCCCGTGACACTGGTGCTCCCATCACCA
TCCCCGTCGGTCCTGGCACTCTGGGCCGTATCGTCAACGTCACTGGTGACCCCATTGAC
GAGCGTGGCCCCGTCAAGGCCGTCAAGCACGCCCCCATCCACACGGAGGCTCCCCCC
TTCGTCGAGCAGTCCACCGCTGCCGAGATTCTCGTCACTGGTATCAAGGTCGTCGATCT
GCTTGCCCCCTACGCCCGTGGTGGTAAGATCGGTCTGTTCGGTGGTGCCGGTGTCGGT
AAGACCGTGTTC

>Atp2 (FG459)_*Neurospora crassa*
GGTACTGAGGGTCTCGTTCGTGGTGCCAAGGCCTCCGACAGTAAGCTTCTCTTTTGATC
CCCTGTCGGCCCTGCCACCCTTGGCCGTATCATCAACGTCACTGGTGACCCCATCGACG
AGCGCGGTCCCATCAAGACCGACAAGTTCCGCCCTATCCACGCCGAGGCTCCCGAGTT
CGTTGAGCAGTCCACCACTGCCGAGATTCTCGTCACTGGTATCAAGGTCGTCGATCTCC
TCGCCCCCTACGCTCGTGGTGGAAAGATTGGTCTCTTCGGTGGTGCTGGTGTCGGCAA
GACCGTCTTC

>Atp2 (FG459)_*Agaricostilbum hyphaenes*
GGTACCGAGGGTCTCGTCAGAGGCACCAAGGTCGTCGACACTGGCGCACCCATCACC
ATCGCTGTCGGCCCCGAGTGCCTTGGTCGTATCATCAACGTCATTGGTGAGCCCATTGA
CGAGCGTGGTCCCATCAACGCCAATGGACTACATAGTATTCACGCTGAGGCACCCGCC
TTCACCGACCAGTCAACAGCCGCCGAGATTCTCGAGACCGGCATCAAGGTTGTCGACT
TGCTCGCCCCTTACGCCCGTGGTGGTAAGATTGGCCTGTTCGGAGGTGCCGGTGTCGG
CAAGACTGTGCTC

>Atp3 (FG543)_*Schizophyllum commune*
ATTGCGTCTACCAAGTTGGCGAAGGCGCAGCGCGCCATGACCTCTGGGAAGGAGTAC
GGCGTCGCGAACACGGGTGCGTGCCCGCGTCAGTCGCGCATTAGACGTGCCTAACTCT
GCGCAGAGGTCTTCAACAACGCGAAGTCTGACCAGCCCCGGAGAACAAGATTGTTGT
CGTGATCTCCTCT

>Atp3 (FG543)_*Coprinopsis cinerea*
ATTGCGTCCACCAAGTTGAACAAGGCACAGCGAGCGATGCAGGCCGGAAAGGAATAC
GGTTTGGCCAACTCTGGTACGAAAACACGACATATACCCGACTAGCTATAACTAAACCA

TGAAACAGAGGTCTTCGAGCACGTCCCAACGGACAAGGCCGTGGCAAGAAGCTCTTC
ATCGTCATCTCGTCC

>Atp3 (FG543)_*Gymnopilus chrysopellus*
ATTGCGTCCACCAAGCTCGCCAAGGCTCAGCGAGCTATGGAGGCTGGAAAGCAATACG
GTATCGCCAACAAGGGTCAGTCCACCTCCTGTAACCACGTAAAATTTCGTATCATTACA
CCCGCAGAGGTCTTCGAACACGTCCCCTCCGACAAGCCCCACCCAACAAGCTCTTCAT
CGTTGTCTCCTCT

>Atp3 (FG543)_*Agrocybe pediades*
ATTGCCTCCACCAAGTTGGCAAAGGCCCAGAGGGCTATGGACGCCGGAAAGAAGTAC
GGTATTGCCAACAATGGTGCGTCGTCGCGCGGTTGATGTTTGGCCGTATGGTTAACGTG
GTTAATAGAGGTCTTCAACCACGTTCCTTCCGACAAGCCCCCCCAACAAGCTCTTCAT
CGTCATCTCTTCC

>Atp3 (FG543)_*Pluteus cervinus*
ATTGCCTCGACGAAACTTGCAAAAGCCCAGCGGGCCATGCAAGCAGGCAAGGAATAC
GGACTGGCTAACAAGGGTGCGTTTAGACGGCTTTCGGAATGGTTAAATATGACGCTTTC
ACTTGCAGAGGTATTCCAACATGCCAACGATAAGCCCGTGCACGCAAGCTCTTCCTTG
TCGTTTCGTCG

>Atp3 (FG543)_*Amanita muscaria*
ATCGCGTCGACCAAGCTTGCAAAAGCCCAGCGCGCAATGCAAGCAGGAAAGCAATAC
GGGCTCGCAAACGCTGGCATGTTGTTAGCTTGCTTACGTCCAAACACGCATTGACCCTT
TGGCAGAGGTTTTCCAACATGCCGCGGACAAACCACCGCCAACAAGCTCTTTGTCGTC
GTCTCTTCC

>Atp3 (FG543)_*Tricholoma matsutake*
ATCGCGTCAACCAAACTTTCGAAAGCTCAGCGAGCTATGCAAGCAGGCAAGCAGTATG
GACTGGCCAACGCTGGTAAGGAACTGATGATAAAACTCACAATACGTTTCTCACACCC
ACACCAGAGGTTTTTGCGAACTCACCAGCGGACACACCCCACCACGCAAGCTCTTCAT
TGTAGTCTCCTCC

>Atp3 (FG543)_*Gymnopus androsaceus*
ATTGCTTCGACCAAGCTCGCCAAGGCTCAGAGGGCCATGCAGGCCGGAAAGCTGTAC
GGTGCAGCCAACGCTGGTCAGTAATCCTAAATTTGGTGACGGCAACTCATTTCCTTCAC
AGAGGTCTTTGAAACTGCAAAAGAGGGCGCGACTGCAGTCGCAAGCTATTCCTTGTCA
TCTCCTCC

>Atp3 (FG543)_*Marasmius fiardii*
ATTGCCTCTACGAAACTAGCAAAGGCTCAGAGAGCCATGCAAGCAGGAAAGGCATATG
GTCTTGCTAATGCTGGTACGTGACCGTTCTGCTCACGTTGGTCCCTTATTTCCTACACCT
TTGTAGAGGTCTTCGATCACTCCAAGGATGACAAGCTCCAACTCGTCGACTTTTCATCG
TCATCTCCTCC

>Atp3 (FG543)_*Hygrophoropsis aurantiaca*
ATTGCGTCCACCAAGCTTGCAAAGGCACAGCGTGCCATGAACAGTGGCAAGCAATATG
GTCTTGCCAACTCCGGTGTGTCCCGACACCATTAGTCTCGGACATGCATCACCATATTA
CATCCAGAGATATTCGAACACACTCCTTCCGAAACCCCGCCAAACGCAAACTTTTCATC
GTCATTTCCTCC

>Atp3 (FG543)_*Boletus edulis*
ATTGCGTCCACCAAGCTTGCAAAAGCGCAGCGTGCCATGAATGCAGGCAAACAATACG
GTATCGCCAACTCTGGTGCGTCCCGTTCACGTCTGTGTGACCGTGTAGACCCTACCCGT
CACTCAGAAATTCTTGACCACACCCCCTCAGAGACGCCTCCAAGCGCCAACTCTTCAT
CGTCGTCTCCTCG

>Atp3 (FG543)_*Fomitopsis pinicola*
ATCGCCTCAACAAAACTCAACAAGGCTCAGCGTGCCATGCAAGCCGGCAAGCAGTAC
GGTATCGCGAACTCCGGTCCGTAGATTGATTCTATATTTCAATGACCTCTAACGCTGAGT
CCTATAGAGGTCTTCCAGCACGCCCCCGCAGAAGAGGCCGCAAGCGCAAGCTCTTCAT
CGTCATCTCTTCC

>Atp3 (FG543)_*Fomitiporia mediterranea*
ATTGCGTCGACGAAACTGGCGAAGGCGCAGCGCGCGATGCAAACTGGCAAACAATAC
GGTGCTGCTAATACCGGTGCGTGCATTTATTATTTGGCCGTGACACATTATACTCTCCTG
TGCGCAGAGATTTTCCAGAATGCTCCTCCCGAGGAAGGTCTAAACGCAAGCTCTATCT
TGTCATCTCCTCC

>Atp3 (FG543)_*Laccaria bicolor*
ATCGCTTCTACCAAGCTCGCAAAAGCCCAGAGGGCTATGCAAGCGGGTAAAGCGTATG
GACTTGCCAACTCTGGTTCGTCCTCTTTTTTTTCTCTCTCGTATATGTAACGCGGAAACG
AAACAGAGGTCTTCGCAAACGTCCCATCAGACAAACCACCCCCAACAAACTCTTCGT
CGTCATCTCTTCC

>Atp3 (FG543)_*Agaricus bisporus* var. *bisporus*
ATTGCTTCTACCAAACTCGCAAAGGCTCAGAGGGCAATGAATGCCGGAAAGCAGTATG
GTATCGCCAACGCGGGTATGTTTTTCACCGTTTTGACTTGGTACACTCTGTCTATCCTTT
CTACAGAGGTCTTTGCCAACACTCCTTCTGATAAACCACACCAACAAAACTCTTCATC
GTTGTATCGTCC

>Atp3 (FG543)_*Serpula lacrymans* var. *lacrymans*
ATTGCTTCGACCAAACTTGCCAAAGCACAACGCGCCATGCAAAGCGGCAAGCAGTAC
GGTCTCGCTAACTCTGGTGAGTTTGTCTTGATATTACACTTTGTAGATATATATTCTTTTT
CAGTGGTTTTCGAAAATGCAACCGCTGAGAACCCACCAAGCGCAAGCTCTTCATTGTG
GTTTCGTCC

>Atp3 (FG543)_*Phanerochaete carnosa*

ATCGCCTCGACAAAACTCAACAAGGCGCAGCGCGCTCCAGGCGGGCAAGGAGTAC
GGCATCGCGAACTCTGGTGCGCCCCACGCAACTCGCCCGAGAACCGACGCCATCTTCT
TCCCTGCAGAGGTCTTCCAGCACGCGCCCGCCGAAAACGTTGCAAGCGCAAGCTCTT
CATCGTCATCTCGTCC

>Atp3 (FG543)_*Trametes versicolor*
ATTGCGTCCACCAAAATGACCAAGGCGCAGCGTGCGATGCAGGCGGGTAAGGCGTAC
GGCCTTGCCAACTCAGGTACGTTATTACTTCGTTGGAACGTGTAACTCCTTCATAATGG
CGTGCAGAGCTCTTCCAGCACGCGACCGCTGAGGATGCTGCAAGCGCACCCTGTTCCT
CGTCATCTCGTCC

>Atp3 (FG543)_*Ramaria acris*
ATTGCCTCCACTAAGCTGAACAAGGCTCAGCGTGCCATGACAGTCGCAAAAGCATACG
GCTCTTCAAACAATGGTGTGTCGGGTTTGGTAGGAGATTGTCGTGTAGTTATTTATCTG
GTGCCAGAGCTGTTTAAACACGCGTCTGCGGATGGCTCGCTCAACGGAAACTCTTTGT
GGTCGTTTCTTCA

>Atp3 (FG543)_*Calocera cornea*
ATTGCCTCGACGAAACTGGCTCGTGCCCAGCGCGCCATGAACGCCGCCAAGATCTACG
GCATTGCCAACACCTCCGTGTACGAGCATGCTGAGGCAGAGAAGACCGAGTCTGAGG
GCAGACAAAGAAGCTGTTCATCGTCG
TCTCCTCC

>Atp3 (FG543)_*Dacryopinax primogenitus*
ATTGCCTCGACGAAGCTGGCTCGTGCGCAGCGCGCGATGACATTTGCCAAGTCGTACG
GCATTGCCAATACTTCAATCTATGAGCACTCCGAGGCCGAGAAGACCGAGACCGAGGG
AAGAAGAAGAAGCTCTTTATTGTGATATCATCC

>Atp3 (FG543)_*Auricularia delicata*
ATCGCGTCGACGAAGCTGAACCAGGCGCAGCGCGCAATGCTCGCGGCCAAGGAGTAC
GGCATCGCCAACGCCGAGGTCAGCCAGCACGCGACGGCGGATGAGGAGGCAAGCGC
CGGCTCTTCGTCGTCATCTCGTCC

>Atp3 (FG543)_*Mycena crocata*
ACAGCGAGCACGAAGTTGGCAAAGGCTCAGCGCGCCATGGCTGCTGGCAAGCAGTAT
GGAATTGCAAACTCCGGTGCGTTGGCTTAATTTTGAAAATCTCCAGTTCTTCACAAACG
ACGAACAGAGGTTTTCGCCCATTCCACCTCGGCTTCCTCCCCTCAAACAAGCTGTTCC
TTGTCATATCCTCC

>Atp3 (FG543)_*Stereum hirsutum*
ATTGCGTCCACGAAATTGGCCAAGGCCCAGCGCGCCATGCAGAACGGCAAGGAGTAC
GGCCGTGCCAACAACGGTCCGTTTCTCCTCAATCAGTGCACAAAATATATCCACACAA
CTCTATCAGAGGTCTTCGCTAACTCTACCTCCGAGGAAGTCCGAAGCGCAAGCTCTTCA
TCGTCATCTCCTCC

>Atp3 (FG543)_*Rhizopus microsporus*
ATTGCTTCCACCAAGGTCAACAAGGCTCAACGTAGTATGGAAGCTGCTCGTGCTTTCG
GTGCTGCTTCTACCTGTAGAGTATACAATTATAGAGGGGGAATTAACTGACACAAATTC
ATGATAGCTCTCTTTGAAAATGCTGAAACCAAGGCTGCTATGCCAAGGTTCTCTTCATT
GCCTCTTCCTCT

>Atp3 (FG543)_*Neurospora crassa*
GTTGCCTCGACCAAGCTCAACCGCGCCCAGCGTGCCATGACCGAGTCCCGCGGCTACG
GTGCCACCAGCAACGAGGTCTTCACCTCGGAGACTAAGCCTCTCGAGGCTGAGGCAA
GAAGAAGCTCGTCGTCGTCTGCTCCTCC

>Atp3 (FG543)_*Asperigillus triticus*
ATCGCCTCGACCCGTCTGACCCGTGCCCAGAAGGCCATGGACGACTCCCGTGCCTACG
GTCAGACTTCCAACACCGTCTTCAAGGAGGCCGAGACCAAGGCCCTTACAAGAAGAC
TCTGCTCGTTGTCGCCAGCTCC

>Cct2 (FG644)_*Tricholoma matsutake*
GTCTTTGGGGCCAGAGTCAAGGTAGACGGGACGGGGAAGCTTGCAGAACTTGAGCGC
GCAGAACGGGTGGGTGACCTGTGTTGTTGAAAGGACAACTAACTCCGTCGCAGGAAA
AAATGAAAGCCAAAGTAGATGCTATCGCTGCTCATGGAATTAACTGCTTCGTGAACCG
ACAGCTGATCTACAACTACCCTGAATCACTCCTGGCGGAGAGGGGCATCATGGTTATTG
AACATGCCGATTTTGAGGGTGTGGAGCGACTATCGCTTGTGACTGGCGGCGAAATTGC
CAGTACGTTTGATCGGCCGGACCTGTGAAACTCGGGCAATGTGAGTTGAT

>Cct2 (FG644)_*Phanerochaete carnosa*
GTCTTCGGCGCACGTCTGAAGGTTGACAGCACAGGCAAACTCGCCGAACTTGAGCGC
GCGGAGCGTGTACGTGTATTTTAAAACTGGCATGGTTGCTAACTGCTGTGAAGGAAAA
AATGAAGGCGAAGGTAGACGCCATTGCAGCACATGGAATCAACTGCTTCGTCAACCGG
CAGCTTATCTACAACTACCCTGAGTCCCTGCTCGCGGAAAGGGGCATTTTGGTTATTGA
ACATGCGGATTTCGAGGGCGTCGAACGGCTTTCGCTCGTGACCGGTGGCGAGATTGCC
AGTACCTTCGACAGACCAGATCTGTCAAGTTTGGACACTGCGAGCTCAT

>Cct2 (FG644)_*Schizophyllum commune*
ATCTTCGGCGCGCGCGTCAAGGTCGACGGCACGGGCAAGCTCGCAGAGCTAGAGCGT
GCAGAAAGGGTACGTCAAATAGCGAAGTGGTACAATGCGCCCACAGGAGAAGATGAA
GGCGAAGGTGGACGCGATCGCGGCGCACGGGATCAACTGCTTCGTCAACCGACAGCT
GATCTACAACTACCCCGAGTCCCTGCTCGCCGAGAAGGGCATTATGGTCATCGAGCAC
GCTGACTTCGAGGGCGTCGAGCGGTTATCACTCGTCACCGGCGGTGAGATTGCCAGTA
CGTTCGACAGCCCAGAGAAGTCAAGCTCGGCCACTGCGAGCTGAT

>Cct2 (FG644)_*Fomitopsis pinicola*
ATCTTCGGAGCGCGTGTGAAGGTCGACAGCACAGGGAAGCTCGCTGAACTCGAGCGT
GCGGAACGGGTACAGACTACACCGTATGCCGATGATTGCACACATGTGGGCAGGAAAA

AATGAAGGCGAAGGTTGAGTCGATCGCGGCGCACGGAATCAACTGCTTCGTCAACCG
GCAACTCATCTACAACTACCCAGAGTCACTATTGGCCGAGAAGGGTGTTCTGACCATC
GAGCATGCCGACTTCGAGGGCGTGGAGCGATTGTCGCTCGTCACTGGTGGCAAATTGT
AAGCACGTTTGACCGCCCTGATCTGTGAAGCTTGGCCGCTGCGAGCTCAT

>Cct2 (FG644)_*Gymnopilus chrysopellus*
ATCTTCGGTGCTCGCGTCAAGGTCGACAGCACAGGGAAGCTTGCTGAGTTGGAACGG
GCTGAACGAGTAAGTGATCATCCACCCATTGCTGTCCTTTTATCTTTGTTCAGGAAAAA
ATGAAGGCCAAAGTTGAGGCTATTGCTTCCCATGGCATCAATTGCTTCGTGAACAGGC
AGCTGATTTACAACTACCCGGAGTCGCTGCTATCTGAAAAGGGGATCTTGGTCATTGAG
CACGCCGACTTCGATGGTGTAGAAAGATTATCGCTGGTTACTGGCGGAGAAATTGCGA
GCACGTTCGATAGGCCAGATCTGTCAAACTCGGTCACTGCGAGCTCAT

>Cct2 (FG644)_*Hygrophoropsis aurantiaca*
ATCTTCGGTGCACGAGTGAAAGTTGATGGGACCGGTAAATTGGCAGAGCTTGAGCGAG
CAGAACGGGTACGTCTAAATATTCGATCGGTTCTCCGCCCAGAGCAGGAAAAAATGAA
AGCAAAAGTGGATGCCATTGCGTCACACGGAATCAACTGTTTTGTCAATCGGCAATTAA
TTTACAACTATCCCGAATCTCTCCTCGCAGAAAAGGGCGTCATGGTCATCGAGCACGCT
GATTTCGAGGGCGTGGAGCGCCTGTCCTTGGTGACTGGAGGTGAGATTGCGAGTACAT
TCGAGCGGCCGGACTTGTGAAGCTAGGCAAGTGCGAGCTGAT

>Cct2 (FG644)_*Agaricus bisporus* var. *bisporus*
GTATTCGGCGCCCGTTTCAAAGTTGATGGCACTAGCAAACTCGCTGAACTCGAACGTG
CAGAGCGTGTATGTTGCCATGCACCCTCATTCGATCCTCACCTTCAATACAGGAAAAAA
TGAAAGCCAAAGTCGAAGCAATAGCCTCACATGGCATCAATGTCTTCGTCAACCGCCA
GCTCATCTACAACTATCCAGAATCTCTCCTGTCAGAAAAAGGAATCATGGTCATCGAAC
ATGCAGATTTTGAAGGTGTAGAGAGACTATCATTAGTCACTGGCGGTGAGATTGCAAGT
ACATTCGATCATCCAGAACTGTCAAGTTGGGCCAATGTGACTTGAT

>Cct2 (FG644)_*Coprinopsis cinerea*
ATCTTTGGTGCAAGACTTAAAGTCGATGGGACTGGTAAACTCGCGGAACTTGAGCGTG
CAGAACGCGTACGTGCCTCTTCTCTGGCATCCGATCCATCTCAATCTTCCAGGAAAAAA
TGAAAGCCAAAGTCGACGCCATCGCTTCGCACGGCATCAACGTCTTCGTCAACCGACA
GCTCATCTACAACTACCCCGAATCCCTCCTCGCTGAGAAGGGCATCATGGTCATCGAAC
ACGCTGATTTCGAGGGTGTGGAGAGGTTGTCGCTTGTTACTGGCGGCGAGATCGCGAG
CACGTTCGATAGGCCTGACCTGTCAAGTTGGGACAATGCGAGTTGAT

>Cct2 (FG644)_*Stereum hirsutum*
ATCTTCGGGGCCCGTGTCAAAGTCGACGGTACTGGCAAACTCGCAGAACTCGAGCGC
GCAGAACGGGTAAGCAATTCCTTTCAAGAGTCCAACGCACTCACTTTAATCAGGAAAA
AATGAAGGCCAAAGTCGACTCCATCGCTTCGCACGGGATCAATGTCTTCGTCAACCGT
CAGCTCATCTACAACTACCCCGAATCTCTTCTCGCCGAGAAGGGCATCCTCACCATTGA
ACACGCCGATTTCGAGGGTGTCGAGCGGTTAAGTCTGGTCACCGGCGGTGAGATCGCG
AGTACATTCGATAGGCCAGATCTGTAAAGCTAGGCAAGTGCGAGTTGAT

>Cct2 (FG644)_*Laccaria bicolor*
ATCTTTGGTGCTCGCGTGAAGGTGGACAGCACAGGGAAGTTGGCAGAGTTGGAACGG
GCTGAAAGAGTACGTTCGACATCTTGACTTTGTATACCCCAGCCTCCTTGCAGGAAAA
AATGAAGGCCAAGGTCGACGCCATCGCAGCGCACGGAATCAACTGCTTCGTGAACAG
GCAGCTCATCTACAACTATCCCGAGTCCCTCCTCACCGAGAAAGGCATCATGGTCATCG
AACATGCCGACTTTGAGGGCGTGGAGAGGCTGTCGCTCGTCACTGGGGGTGAAATCG
CAAGCACCTTTGACCGACCGGATCTGTCAAGCTTGGGCAGTGCGAGTTGAT

>Cct2 (FG644)_*Trametes cinnabarina*
GTATTTGGTGCCCGCATCAAGGTTGACAGCACGGGCAAGCTCGCTGAGCTAGAGCGTG
CGGAGCGGGTAAGTCCCACTTTGTACGGATTTGGGCACCGTCCCCATTGCAGGAAAAA
ATGAAGGCCAAAGTGGATGCGATTGCAGCTCACGGAATTAATTGCTTTGTGAATCGCC
AACTTATCTACAACTACCCCGAGTCACTTCTCGCAGAGAAGGGCATCATGACGATCGA
GCATGCTGACTTTGAGGGTGTTGAGCGTCTCGCGCTTGTCACTGGGGGTGAGATCGCA
AGCACATTCGACCATCCGGAGTTGTGAAGCTTGGACGGTGTGAGCTCAT

>Cct2 (FG644)_*Auricularia delicata*
GTCTTCGGCGCGCGCATCAAGGTCGACTCGACGGGCAAGCTTGCAGAGCTCGAGAAG
GCCGAGCGGGTGAGCCGTTTCCGATATGAACAGGCGGCATTCGCACTGTTCAGGAAAA
AATGAAGGCAAAGGTTGCGGCCATCGCAGCGCACGGCATCAACTGCTTCGTCAACCG
GCAGCTCATCTACAACTTCCCCGAAAACCTGTTAGCGGAGCAGGGCATCATATCCATAG
AGCACGCCGACTTCGAGGGCGTCGAGCGGCTCTCGCTCGTCACGGGCGGTGAGATTG
CGAGCACGTTCGACCGCCCGGACCTGTCAAACTCGGCCACTGCGACCTCAT

>Cct2 (FG644)_*Agrocybe pediades*
ATCTTCGGTGCTCGCTTCAAGGTCGACAGTACCGGAAAACTGGCCGAACTCGAACGTG
CTGAAAGGGTAATTATTCACGCTTTCCTTCCATCCCTTTGCAGGAAAAAATGAAAGCCA
AGGTTGAAGACATCGCTGCGCATGGCATCAACGTCTTCGTCAACAGGCAACTCATCTA
CAACTACCCCGAGTCGTTATTGACGGAGAAGGGCATCATGGTCATCGAACATGCGGAC
TTCGAGGGTGTCGAGAGGTTGTCGTTGGTCACTGGAGGAGAGATTGCAAGCACGTTTG
AGAGGCCCGATCTGTGAAACTGGGACACTGTGAACTGAT

>Cct2 (FG644)_*Pluteus cervinus*
ATATTCGGTGCACGTCTAAAAGTTGATGGTACAGGCAAACTCGCCGAGTTCGAACGTG
CAGAGCGGGTAAGATTCAGATTACTGGTTTCTGCTATATATTCAGGAAAAAATGAAAGC
CAAAGTCGAAGCTATCGCTCAACACGGAATAAATTGTTTCGTGAATCGACAACTGATCT
ATAACTATCCCGAATCTCTGTTAGCAGAGAAGGGAATAATGGTAATCGAACATGCAGAC
TTTGAAGGCGTGGAACGATTGTCTTTGGTTACCGGTGGTGAGATCGCAAGTACGTTTG
AACGACCGGAACTGTGAGCTTGGGACACTGTGATTTGAT

>Cct2 (FG644)_*Ramaria acris*
ATATTTGGTGCCCGTGTCAAAGTAGATGGCACAGGGAAACTCGCTGAGCTCGAACGTG
CGGAGAGAGTTAGTGTTTTTCAAAGTTTACAATGTCATGCTCTAGGAAAAAATGAAAG

CTAAAGTTGCGCTGATTTCAGCGCACGGCATCAACTGTTTGTAAACCGACAGTTGATC
TATAACTACCCTGAAAATTTGCTTGCAGAGCAAGGCATTCTATCGATAGAGCATGCAGA
CTTCGAGGGCGTGGAGCGCCTTTCGCTCGTCACGGGCGGCGAGATTGCGAGTACTTTT
GACAGACCAGATCTGTAAAACTCGGACGGTGCGATTTGAT

>Cct2 (FG644)_*Gautieria morchelliformis*
ATTTTCGGTGCCAGGGTCAAGGTGGATGGCACAGGGAAGCTCGCTGAGCTCGAACGG
GCCGAGCGAGTGAGTGCACGTCGGACCTGACGCTGGGCAGCTTTTAGGAAAAAATGA
AAGCAAAAGTGGCGTCGATAGCGGCGCATGGCATCAATTGCTTTGTTAACCGCCAACT
GATCTACAATTACCCCGAAAATTTGCTCGCGGAGCAGGGTATCATGTCGATTGAACATG
CCGACTTTGAAGGTGTCGAGCGCTTATCGCTGGTAACGGGTGGGGAGATCGCTAGTAC
GTTCGAACGCCCAGACCTGTAAAGCTTGGGAGATGCGATCTGAT

>Cct2 (FG644)_*Marasmius fiardii*
ATCTTTGGCGCACGAGTGAAAGTAGACTCTACAGGAAAACTTGCTGAGCTTGAACGG
GCCGAGAAGGTGTTCTACTCGTCTCCTCTTCTGTCACCCTTTCCAGGAGAAAATGAAG
GCCAAGGTCGATGCAATTGCCGCTCATGGTGTCAACTGTTTCGTCAACCGTCAACTGAT
CTACAACTACCCTGAATCCCTCCTCGCCGAGAAAGGCATAACAGTCATAGAACATGCC
GATTTCGAAGGCGTCGAGCGACTTTCACTCGTAACAGGTGGCGAAATCGCTTCGACTT
TTGATCGACCCGATCTGTCAAGCTAGGACACTGTGATCTGAT

>Cct2 (FG644)_*Amanita muscaria*
GTTTTCGGAGCACGCATCAAAGTAGACAGTACAGGAAAGCTCGCTGAACTAGAGAGG
GCGGAACGGGTGAGTATATGCGCGTCAGTCTCATCCACTCATCATATGTCAAGGAAAAA
ATGAAAGCCAAAGTCGAAGCCATCGCTGCACACGGCATCAACTGCTTCGTAAACCGAC
AACTCATCTACAACTACCCTGAATCGATTCTTGCAGAAAAGGGAATCGTGGTTATCGAA
CATGCCGATTTTGAGGGTGTTGAACGTCTCTCGCTAGTTACAGGGGGAGAAATCGCGA
GCACGTTTGATCGACCTGAGCTGTCAAGCTAGGTCACTGTGATTTGAT

>Cct2 (FG644)_*Lactarius quietus*
ATTTTCGGTGCCCGCATGAAGGTCGACAGCACCGGGAAGCTTGCGGAGCTAGAACGC
GCAGAGCGGGTGCGTATATGCTACCCTTCAAGTCAGTCTGACTGTGGTAACAGGAAAA
AATGAAGGCCAAGGTGGAGGCCATCGCATTACACGGGATCAACGTCTTTGTTAATCGA
CAACTGATTTACAACTACCCTGAAAGTATCTTCGCAGAGAAGGGTATTATGTCGATCGA
GCACGCCGATTTCGAAGGTGTTGAACGTTTGTCACTGGTCACTGGTGGGGAGATCACG
AGCACGTTCGACCGGCCAGAACTGTCAAGCTCGGTCGATGCGACTTAAT

>Cct2 (FG644)_*Boletus edulis*
GTCTTTGGTGCTCGTGTAAAGGTAGACTCTGCGGGTAAACTTGCGGAACTTGAACGTG
CAGAACGAGAAAAGATGAAGGCAAAAGTGGAAGCGATTGCAGCCCACGGCATAAACT
GTTTCGTCAACCGGCAACTCATATACAACTACCCCGAATCCCTGCTTGCGGAGAAGGG
CATCATGGTCATCGAGCATGCAGATTTCGAGGGCGTTGAGCGTCTGTCACTCGTGACTG
GCGGTGAGATTGCAAGTACGTTCGATCGACCCGATCTGTGACACTTGGGAGATGTGAG
TTGAT

>Cct2 (FG644)_*Fomitiporia mediterranea*
ATATTCGGCGCACGAGTGAAGGTCGACAGCACTGGGAAGCTTGCCGAGTTAGAACGT
GCTGAACGTGAAAAAATGAAGGACAAAGTAAAAGCAATCGCTGCACATGGTATCAAC
TGTTTCATCAACCGACAGTTGATCTATAACTATCCTGAATCTCTTTTGGCTGAGCATGGC
ATTTTGACGATTGAACATGCGGACTTTGAAGGTGTTGAGAGGCTTTCGCTCGTAACTG
GTGGAGAAATTGCAAGTACATTTGAACAGCCGGATAAGTGAAGCTTGGTCGATGCGAA
CTCAT

>Cct2 (FG644)_*Agaricostilbum hyphaenes*
ATCTACGGTGCCAAGATGAAGGTGGAAAGCACAGGCAAGCTGGCTGAACTGGAGCGC
GCCGAGAAGGTGCGTGGCAGAGTCAGCTGGCCTGACCGTTTGTGATGTCACAGGAGA
AGATGAAAGCTAAGGTCTCCAAGATTGCATCACACGGCATCAACTGCTTTGTGAACCG
ACAGCTGGTCTACAACTACCCAGAGTCGCTCTTTGCTCAGCATGGCATCATGGTCATCG
AGCATGCCGACTTTGAAGGTGTTGAGCGACTGGCTCTGGTCACGGGCGGTCAGATCAC
TTCGACCTTCGAAAACCCAGAGTCGTCAAGATTGGTCAGTCGGCGCGCAT

>Cct2 (FG644)_*Calocera cornea*
ATCTTCGGTGCGCGGGTGAAGGTGGACTCGACGGGCAAGCTTGCCGAGCTGGAGCGC
GCGGAGCGCGAGAAGATGAAGGCCAAGGTCAAGTCGATTGCGGCGCTGGGGATAAAC
GTGTTTGTGAACCGGCAGCTGGTGTACAACTACCCCGAGAACCTGCTCGCAGAGCAC
GGCATCATGTGCATCGAGCACGCGGACTTTGAGGGCGTCGAGCGCCTCGCGCTTGTGA
CCGGCGGGGACGTCGTCAGCACGTTCGACGGCGCCGAGGCGTCAAGCTCGGGAAATG
CGAGGTCGT

>Cct2 (FG644)_*Dacryopinax primogenitus*
ATTTTCGGAGCGCGGGTAACGGTCGACAGCACTGGCAAGCTCGCCGAGCTCGAGCGG
GCCGAGCGGGCAAAGATGGCCGCCAAAGTCAAGTCCATCGCCGGCCTGGGGATCAAC
GTCTTCGTCAACCGGCAGTTGGTATACAACTACCCCGAGAACCTGCTTGCTGAGATGG
GAATCATGAGCATCGAGCATGCGGATTTTGAGGGTGTGGAGCGGCTTGCCCTTGTGAC
GGGGGGCGATGTCGTGAGCACGTTTGAGGGCGCGGAGGCGTCAGGCTGGGGAAATGT
GATCTCGT

>Cct2 (FG644)_*Asperigillus triticus*
ATCTTCGGTGCCCGGGTCAAGGTCGAGTCGACGGGCAAGCTGGCCGAGTTGGAGAAG
GCGGAGCGCGAGAAGATGAAGGCAAAGGTTGAGCGCATCAAGTCCCACGGCATCAAC
TGCTTCGTCAACCGACAGCTGATCTACAACTGGCCCGAGCAGCTGTTCACCGAGGCTG
GCATCATGTCCATCGAACACGCCGACTTCGACGGCATCGAGCGTCTGGCCCTGGTGAC
GGGCGGTGAGATCGCCTCGACTTTCGACGACCCGGAGCAGTCAAGCTGGGCAAGTGC
GATGTGAT

>Cct2 (FG644)_*Neurospora crassa*
ATCTTTGGTGCGCGCGTCAAGGTCAACTCTACCAGCAAGCTCGCCGAGCTTGAGAAGG
CCGAGAAGGAGAAGATGAAGGCCAAGGTTGAGAAGATCAAGGCGCACGGCATTAACT

GCTTCATCAACAGGCAGTTGA
TTTACAACTGGCCCGAGCAGCTGTTCACCGATGCCGGCATCATGTCGATCGAGCATGCC
GATTTCGATGGCATTGAGCGCCTTGCTCTCGTTACCGGCGGTGAAATCGCTTCTACCTT
CGACCACCCGGAGCAGTCAAGCTTGGATATGCCGACTTGAT

>Cct2 (FG644)_*Rhizopus microsporus*
ATTTTCGGCGCTCGTGTCAAGGTCGATGCCACAGGTAAATTAGCCGAATTAGAGCGTGC
AGAACGTGACAAGATGAAGGAAAAGGTTGAGAAAATCAAAGCTCACGGCATCAACTG
CTTTGTGAACCGTCAATTGATCTATAACTGGCCCGAACAACTCTTGGCTGATGCAGGCA
TTGCTAGCATTGAACACGCCGACTTTGAAGGTGTCGAAAGATTAGCCTTGGTCACTGG
TGGTGAAATTGCCTCTACCTTTGATCATCCAGAATTGTCAAGCTTGGACACTGTGATTT
GAT

>Cct3 (FG861)_*Agrocybe pediades*
CTGGACCCTATGGGAGGGATTCTGTATGCATGCTTGTGTCACTATCTGCTGTGATCTAAT
ATGCTTATACAGTCTTACGAACGATGGCAACGCTATCTTACGAGAAATTGACGTCGCCC
ACCCTGCTGCAAAAAACATGATCGAGCTCAGTCGTACTCAGGATGAGGAATGTGGCGA
TGGAACAACGAGTGTTATCATTCTT

>Cct3 (FG861)_*Gymnopilus chrysopellus*
CTCGATCCTATGGGTGGCATTCTGTGCGTTAATCGGACTTTTTTCTGCGTTTTCCTTCTTA
TTCTTGCACAGGCTGACGAATGACGGAAATGCGATTCTTCGAGAAATCGATGTCGCAC
ACCCTGCTGCGAAAAATATGATCGAGCTTAGTCGGACACAAGACGAGGAATGTGGAGA
TGGAACCACTAGTGTGATCATCCTC

>Cct3 (FG861)_*Schizophyllum commune*
CTGGATCCTATGGGTGGTATCCTGTACGTTTTCTCCAGCACTTGCAGACTTAAATTGAA
GATCCCTCGCAGACTTACCAACGACGGCAATGCGATCTTGCGAGAGATCGATGTCGCT
CATCCGGCTGCGAAGAACATGATTGAGTTGAGTCGCACACAAGACGAGGAGTGCGGA
GACGGAACGACGAGTGTGATTATCCTC

>Cct3 (FG861)_*Phanerochaete carnosa*
CTCGACCCGATGGGTGGTATCTTGTACGTCCGCGTCGCATGGATCATGCGCTGTGCTGA
CGGCATTCTCCAGACTGACCAACGATGGGAATGCGATTCTGCGAGAAATAGATGTTGC
ACATCCTGCAGCGAAGAACATGATTGAGTTGAGTCGGACACAAGACGAGGAGTGCGG
TGACGGTACGACGAGTGTCATCATCCTA

>Cct3 (FG861)_*Fomitopsis pinicola*
TTAGATCCTATGGGTGGTATCCTGTACGTCTGCCGTTTTGTTTGCTGGCTTTCTGTTGAC
TATACTGTCTAGGCTGACAAACGACGGAAATGCCATCTTGCGAGAGATCGACGTTGCA
CATCCAGCAGCAAAGAACATGATCGAGCTTAGTCGGACGCAAGATGAAGAGTGCGGT
GACGGCACTACCAGTGTCATTATCCTG

>Cct3 (FG861)_*Laccaria bicolor*

CTCGATCCGATGGGGGGTATCCTGTGCGTTAAGATTTTGACATCGAGAGGACTAATGCC
AGGTGTATCAAAGGCTAACGAACGACGGAAACGCGATCTTGCGTGAAATAGACGTGG
CTCACCCTGCAGCGAAGAATATGATTGAGTTGAGCCGAACACAGGATGAGGAGTGTGG
TGATGGAACAACCAGCGTCATTATTCTT

>Cct3 (FG861)_*Coprinopsis cinerea*
TTGGATCCTATGGGTGGTATCTTGTGGGTATTACTGGGTACCGAGGCAGGGAGGCTGAT
TATGGGGACACAGGTTGACGAACGACGGCAATGCGATCCTGCGCGAAATCGACGTTGC
GCATCCCGCTGCGAAGAACATGATCGAGTTGAGCAGGACACAGGACGAGGAGTGCGG
AGATGGAACGACGAGCGTTATCATCCTC

>Cct3 (FG861)_*Auricularia delicata*
CTGGATCCCATGGGTGGCATCGTGTGCGCGCTGTGGCTGCTGGTGTACCGTAGGCTGAT
CAGGCGTTATCAGCGTGACGAACGACGGCAACGCCATTTTGCGCGAGATCGACGTTGC
GCACCCAGCGGCGAAGAACATGATCGAGCTCGCACGGACGCAGGACGAAGAGGTCG
GCGACGGAACAACGTCCGTAATCGTCCTT

>Cct3 (FG861)_*Agaricus bisporus* var. *bisporus*
TTGGATCCGATGGGCGGTATACTGTGCGTGCTTGCAAAACCGAAGCACCATATCTGAAT
TTTTCGTTTTCAGACTTACCAATGACGGAAATGCCATTCTGCGTGAAATCGACGTGGCG
CACCCTGCTGCAAAAAACATGATTGAACTTTCCCGCACGCAGGATGAGGAGTGCGGTG
ACGGCACGACGAGCGTTATCATCCTT

>Cct3 (FG861)_*Boletus edulis*
TTAGATCCGATGGGCGGTATCTTGTACGACCTTCATCTCTCTCTCATTTTCCTCTCTTACT
TACGTCTCGAGACTGACGAATGACGGTAACGCAATCCTACGTGAGATCGACGTTGCTC
ATCCTGCTGCCAAGAACATGATTGAACTCAGTCGAACCCAGGATGAAGAATGCGGCGA
TGGAACGACAAGCGTGATCATCCTA

>Cct3 (FG861)_*Dacryopinax primogenitus*
CTCGATCCGATGGGCGGTATCCTGTGCGTCTAATTTGTCCCATACCTATACCGTCTAATAT
GGTGCCTGTAGGTTGACAAATGATGGGAATGCTATTTTACGAGAGATTGATGTTGCTCA
CCCGGCTGCGAAGAACATGATTGAGCTCAGCCGGACACAGGATGAGGAGGTTGGTGA
TGGAACCACAAGTGTCATTATCTTA

>Cct3 (FG861)_*Calocera cornea*
CTCGACCCCATGGGCGGCATCCTGTACGTCCACAACCGTGTGGCACACACTTGTCTTAT
CATGAATCGACAGGCTCACCAACGATGGGAATGCGATACTGCGCGAGATTGATGTTGC
ACATCCTGCGGCGAAAAACATGATTGAGCTTAGTCGGACGCAGGATGAGGAGGTTGGC
GATGGGACGACGAGTGTCATCATTCTT

>Cct3 (FG861)_*Tricholoma matsutake*
CTAGACCCTATGGGAGGCATCCTGTACGTCTGGAATCAGCAACATATATATACTTACGAT
CATATGTATCAGGCTAACTAACGATGGAAATGCTATTCTGCGGGAAATCGATGTAGCCCA

TCCAGCTGCGAAAAACATGATAGAGCTCAGCCGGACCCAGGATGAGGAATGTGGGGA
TGGAACGACAAGTGTTATTATCCTC

>Cct3 (FG861)_*Stereum hirsutum*
TTGGATCCTATGGGTGGCATTCTGTTCGTACCTTCCTTTGCCATTCTCTTCTCTTCTTGAC
GGCTTGAACAGTCTAACGAATGATGGAAATGCCATCCTTCGTGAGATTGATGTTGCGCA
CCCAGCTGCTAAAAACATGATCGAACTGTCTCGGACGCAGGATGAGGAGTGTGGCGAT
GGGACGACCAGTGTCATTGTGCTG

>Cct3 (FG861)_*Marasmius fiardii*
CTAGATCCAATGGGAGGAATTCTGTAAAATTACTATTTCACTTTCGAAGGTTTGCTGAC
GAAAAATCATCAGGTTGACAAACGATGGAAACGCAATCCTTCGAGAAATTGACGTAGC
TCATCCAGCAGCGAAGAACATGATCGAGCTTAGTCGGACCCAAGATGAGGAATGTGGA
GACGGAACTACAAGTGTGATTATTCTT

>Cct3 (FG861)_*Pluteus cervinus*
TTGGATCCCATGGGTGGTATATTGTTCGTAATTTCCCTCTCCTGCTCGATCAACCTCTGA
CCATCGATTTAGACTAACAAACGACGGTAACGCGATTCTACGTGAAATAGACGTAGCCC
ATCCCGCAGCCAAGAACATGATCGAACTTAGTCGGACACAAGACGAAGAGTGTGGAG
ATGGAACAACGAGCGTGATTATTCTT

>Cct3 (FG861)_*Fomitiporia mediterranea*
CTCGATCCTATGGGTGGTATTTTGTATGTTGCAGTTTCGTTTAACTTGCCGACCTATTAAT
CGCTGTTCAAGGCTTACGAATGACGGAAACGCAATTTTACGAGAGATAGAAGTGGCAC
ATCCCGCAGCGAAAAACATGATTGAACTAAGTCGGACGCAAGATGAGGAATGTGGAG
ATGGGACGACGAGTGTCATCATTCTT

>Cct3 (FG861)_*Trametes versicolor*
TTGGACCCGATGGGCGGTATTCTGTGAGCATTCATTCTCCTGCATAAAATACATTGCGTG
ACCTCTCCCTAGGTTGACAAACGATGGAAATGCTATCCTCCGTGAAATTGATGTCGCTC
ACCCGGCCGCGAAAAACATGATCGAGCTTAGCAGGACGCAAGACGAGGAGTGCGGAG
ATGGCACAACGAGTGTTATCATCCTT

>Cct3 (FG861)_*Lactarius quietus*
CTCGACCCGATGGGCGGCATAGTGTGAGCCTTTCTGCTGAATCTCCCTTTCCCTTAAGT
TGTTGTGGCCTAGCCTCACAAACGATGGAAACGCGATTCTTCGCGAGATAGACGTTGC
TCATCCCGCAGCCAAGAATATGATCGAGTTGAGCCGGACACAGGATGAGGAGTGCGGC
GACGGGACGACGAGTGTGATCGTCTTA

>Cct3 (FG861)_*Serpula lacrymans* var. *lacrymans*
TTGGATCCAATGGGAGGTATCTTGTAAGCCGATCGCGAGTAGACCATGTTTCTGTTAAA
TTCCGTCCAACAGACTCACCAATGATGGGAATGCCATTCTTCGCGAAATTGATGTAGCT
CATCCCGCCGCAAAGAACATGATCGAGCTCAGTCGGACCCAGGACGAGGAATGTGGG
GATGGGACGACAAGCGTCATCATTCTA

>Cct3 (FG861)_*Ramaria acris*
TTGGATCCAATGGGTGGAATTACGTGGGTTTACTCTTTATACTCTCATTTTCTGTTTTAA
GACACGACAAAGGCTCACCAATGATGGAAATGCTATTTTACGCGAGATTGATGTCGCC
CATCCTGCTGCAAAGAATATGATTGAGTTGAGTCGGACACAGGACGAGGAAGTCGGCG
ATGGAACAACATCGGTTATCATCCTT

>Cct3 (FG861)_*Gautieria morchelliformis*
TTGGATCCCATGGGCGGCATCCTGTGCGTTTCATCTTATTATCCATAGTAAGACACAGTG
AAGGCTTACTAACGATGGTAATGCCATTCTGCGCGAAATTGACGTTGCACATCCCGCTG
CGAAGAACATGATCGAGCTCAGTCGGACACAAGACGAAGAAGTTGGTGATGGGACGA
CATCAGTCATCATCCTT

>Cct3 (FG861)_*Hygrophoropsis aurantiaca*
CTTGATCCAATGGGGGGCATATTGTAAGCACAACATTCTGATATGATGTACCGGTTCAAT
ACGGCTGCACAGGCTAACAAACGATGGCAATGCCATTCTCCGAGAAATCGACGTCGCA
CACCCCGCTGCCAAAAACATGATCGAGCTCTCCAGAACCCAAGACGAAGAATGCGGT
GATGGGACTACGAGCGTTATCATCCTC

>Cct3 (FG861)_*Gymnopus androsaceus*
TTGGATCCGATGGGAGGGATATTGTAAGTCAACAAAAAAAAAGTGTACATTCACCAAC
TAATATTTCTCAGATTAACCAACGACGGAAATGCAATCCTCCGAGAAATCGACGTGGCG
CATCCCGCAGCGAAAAACATGATTGAACTCAGCCGAACGCAGGATGAAGAATGCGGA
GATGGTACAACAAGTGTAATTGTCCTT

>Cct3 (FG861)_*Asperigillus triticus*
TTGGACCCCATGGGCGGTATTGTTCTGACCAACGATGGTCATGCAATCCTGCGAGAGAT
TGAAGTGTCGCACCCCGCAGCAAAGAGCATGATCGAGCTTAGCCGCACGCAGGATGA
GGAGGTTGGAGACGGTACCACGACCGTCATCATTCTT

>Cct3 (FG861)_*Neurospora crassa*
CTCGATCCCATGGGCGGTATCGTCCTCACCAACGACGGCCATGCTATTCTCCGCGAGAT
CGAAGTGTCGCACCCCGCAGCCAAGAGCATGATCGAGCTCAGCCGGACACAAGACGA
AGAAGTTGGTGACGGTACCACAACAGTCATCATTCTT

>Cct3 (FG861)_*Rhizopus microsporus*
TTAGACCCCATGGGTGGCATCTTGTTAACAAATGATGGAAATGCCATTCTTCGTGAAAT
TGAAGTGGATCATCCAGCTGCTAAAAGTATGATTGAACTTTCTAGAACCCAAGATGAA
GAAGTAGGCGATGGAACAACCAGTGTCATCATTCTC

>Cct3 (FG861)_*Agaricostilbum hyphaenes*
TTGGATCCTATGGGCGGCATTCTCTTGACAAACGACGGGCATGCCATTCTCCAGGCGAG
AGCAGTCTTTGAGGAGAGCTGATCTCGCTTGTTCTTCCCACAGGAAATTGAAGTCGCT

CATCCAGCAGCAAAGAGCATGCTCGAGCTCAGCAGGACACAGGATGAAGAGTGCGGC
GATGGAACGACTTCAGTCATCATTCTA

>Cct5 (MS422)_*Mycena crocata*
CGTCAAGGATACCGGCGCAAATCTGGTCATCTGCCAATGGGGTTTCGACGACGAGGCC
AACCATCTGCTAATGCAGAACGAACTGCCGGCTGTGCGATGGGTTGGCGGACCTGAAA
TCGAGGTGAGTCGAAGTGAAATCCTGCGCTGACTTTTTCTTCTTTGTCAAGCTGATCGC
AATAGCGACCCAAGGGCGCATTGTGCCTCGATTTGAGGATCTCACGGCGGAGAAGCTG
GGCAAGGCTGGCATCGTGCGTGAGGTCTCGTTTGGGACAACCCG

>Cct5 (MS422)_*Agrocybe pediades*
TGTAAAGGACACTGGTGCAAACCTAGTTATTTGCCAGTGGGGTTTCGATGACGAAGCC
AACCATCTGCTCATGCAAAACGAACTCCCTGCAGTGCGGTGGGTTGGTGGTCCAGAAA
TCGAGGTTTGTGCTTTAAAATTTTGACCCATGCTCAGTTGCATTTCAGTTGGTTGCTATT
GCTACCCAAGGAAGAATCGTACCTAGGTTCGAGGATCTGACAGAAGCGAAACTCGGC
AAGGCCGGTATTGTGCGCGAGATTTCGTTTGGTACTACCCG

>Cct5 (MS422)_*Hygrophoropsis aurantiaca*
GGTGAAGGACACGGGCGCGAATCTGGTTATCTGTCAGTGGGGCTTTGATGATGAGGCG
AATCATCTGTTGATGCAAAATGAGCTGCCTGCTGTGCGGTGGGTCGGTGGACCAGAGA
TCGAGGTACGTTTGCCATAATGTTCCTCGTGTTGTTTACGTCGTGTTTTAGTTGATTGCA
ATAGCAACGCAAGGCCGAATCGTTCCCCGTTTCGAGGACCTCACTGCCGCAAAGCTCG
GAAAAGCAGGGATCGTACGTGAAGTATCCTTCGGTACCACACG

>Cct5 (MS422) _*Neurospora crassa*
AATAAAGAACACTGGTGCCAATCTGGCCATCTGCCAGTGGGGTTTCGACGACGAGGCG
AACCACTTGCTCCTTCAGAACAACCTTCCTGCTGTCCGATGGGTCGGTGGTCCCGAGA
TTGAGCTGATTGCGATTGCCACAAACGGTCGCATTGTTCCTCGCTTCGAGGATCTCAAG
CCCGAGAAGCTCGGTAGGGCTGGTGTTGTTCGCGAGATGACATTCGGTACGACAAG

>Cct5 (MS422)_*Schizophyllum commune*
CGTGAAGGACGCGGGCGCGAACCTGGTCATCTGCCAGTGGGGCTTCGACGACGAGGC
GAACCACTTGCTGATGCAGAACGACTTGCCCGCTGTGCGATGGGTGGGTGGCCCGGA
GATTGAGTTGATTGCGATTGCGACGCAGGGGCGGATTGTTCCTCGCTTCGAGGACCTTA
CTCCGGAGAAGCTCGGTCGGGCGGGAGTTGTGCGTGAGGTGACCTTCGGTACAACGC
G

>Cct5 (MS422) _*Auricularia delicata*
CGTCAAGGACGCCGGCGCGAACCTTGTCATGTGCCAGTGGGGCTTCGACGACGAGGC
GAACCACCTTCTCCTGCAGAACGACCTCCCCGCCGTGCGCTGGGTCGGTGGGCCCGA
GATCGAGCTCGTGGCCATCGCGACGCAGGGCCGCATCGTGCCGCGCTTCGAGGACCTC
GCGCCCGAGAAGCTCGGCCGCGCGGGCCTTGTGCGCGAGCTCACGTTCGGCACGACG
CG

>Cct5 (MS422) _*Trametes versicolor*
CGTCAAGGACACCGGCGCGAACCTTGTCATCTGCCAATGGGGCTTCGACGACGAGGC
GAACCACTTGCTCATGCAGAACGATCTCCCTGCTGTGCGGTGGGTCGGTGGACCCGAG
ATCGAGGTTGGTTCTTTGAGGTAAAGTTTGTCGGCTAACATACATCGCAGCTCATTGCG
ATTGCGACCAACGCTCGTATCGTTCCTCGGTTCGAGGACCTCACGAAGGAGAAGCTTG
GCCGTGCAGGTGTCGTGCGGGAAGTGACCTTCGGCACAACGCG

>Cct5 (MS422) _*Phanerochaete carnosa*
TGTCAAGGACACCGGTGCAAACCTTGTTATCTGCCAGTGGGGCTTCGACGATGAGGCA
AACCATTTGCTCATGCAAAACGATCTGCCCGCTGTGCGGTGGGTTGGTGGTCCTGAGA
TCGAGGTACGGGAATGATTGAAGCGTTCATAGTTTTTATACTTCACCCTAGCTTATTGCG
ATTGCCACCAATGGTCGGATTGTCCCTCGCTTCGAAGACTTGTCATCAGAGAAGCTTG
GTCATGCAGGTGTTGTTCGCGAAGTCACCTTCGGTACTACGCG

>Cct5 (MS422) _*Fomitopsis pinicola*
GGTGAAGGACACCGGCGCGAACCTGGTAATCTGCCAGTGGGGCTTCGACGACGAGGC
GAACCATCTGCTCATGCAGCACGACCTGCCTGCGGTGCGGTGGGTCGGCGGCCCCGA
GATCGAGGTGCGGAGGTTATTCTAGTGGATACGAACCCTAACGAACCACCTAGCTCATC
GCCATCGCAACAAATGGGCGTATCGTCCCGCGCTTCGAAGACCTGACGGCAGAGAAG
CTCGGCAAGGCAGGCATCGTTCGCGAAGTGTCTTTCGGGACCACAAG

>Cct5 (MS422) _*Dacryopinax primogenitus*
CGTGAAAGACGCGGGTGCAAACCTCGTCATATGCCAGTGGGGATTCGACGATGAGGCC
AACTCGCTTCTAATGGCAAACGACCTACCCTCGGTCAGATGGGTCGGAGGTCCCGAGA
TTGAGCTCATCGCTATTGCGACGCATGGGCGAATCGTGCCGAGGTTCGAGGACCTCAG
TGCTGAGAAGCTTGGAAAGGCTGGGTTGGTCAAGGAGATCGCCTTCGGTACGACAAG

>Cct5 (MS422) _*Calocera cornea*
CGTCAAGGACGCGGGCGCCAACCTCGTCATCTGCCAGTGGGGTTTCGACGACGAGGC
CAACTCACTGCTTATGGCGAACGACCTGCCGGCGGTGCGGTGGGTCGGGGGGCCGGA
GATCGAGCTTATCGCTATTGCGACCCAGGGGAGGATCGTGCCACGGTTCGAAGACCTT
AGCGCGGAGAAGCTCGGAAAGGCCGGCATCGTCAGGGAGATCTCGTTTGGGACCACA
AG

>Cct5 (MS422) _*Laccaria bicolor*
AGTGAAGGATACTGGCGCGAATTTAGTCATCTGTCAATGGGGCTTTGATGATGAGGCCA
ACCATTTGTTGATGCAGAACGAATTGCCCTCTGTGAGATGGGTTGGAGGCCCAGAAAT
CGAGGTTTGTAGTTTGATCTATTGATTGACGTCTTTTCTTTCCTTCCCAGCTCATCGCCA
TTGCGACACATGGGCGAATCGTTCCCCGCTTTGAAGACCTCACCAAAGAGAAGCTGG
GCAAAGCGGGCATTGTACGAGAAATTTCGTTCGGTACAACTCG

>Cct5 (MS422) _*Tricholoma matsutake*
GGTCAAGGACACGGGCGCAAACTTGGTCATCTGCCAATGGGGTTTCGATGATGAGGCA
AATCATCTGTTGATGCAAAACGAACTACCCGCTGTGCGATGGGTTGGCGGGCCTGAAA

TTGAGGTACGAGTTGCTATATTGATATCTATATTCCCTTTCCTGCAAGCTCATCGCAATAG
CAACACAAGGCCGAATTGTCCCACGTTTCGAAGACCTCACAAAGGAAAAGCTCGGAA
CAGCAGGAATCGTCCGTGAGATCTCATTTGGTACAACACG

>Cct5 (MS422) _*Serpula lacrymans* var. *lacrymans*
AGTAAAGGATACTGGCGCCAACTTGGTTATCTGTCAGTGGGGCTTTGACGATGAGGCT
AATCACTTGCTGATGCAAAATGAATTGCCCGCTGTCCGCTGGGTTGGTGGCCCAGAGA
TCGAGGTGCGCAATTGCTTATACTTATTGACGACCCAAACCTTCTATTCAGCTCATAGCA
ATAGCCACGCAGGGTCGTATCGTGCCGCGATTTGAGGATCTTACTGCAGAAAAGCTTG
GTAAAGCAGGCATAGTGCGGGAGGTCTCCTTTGGCACCACGCG

>Cct5 (MS422) _*Stereum hirsutum*
GGTGAAGGATAGTGGGGCCAACCTGGTCATCTGCCAATGGGGTTTCGACGACGAAGC
AAATCATTTACTGATGCAGAACGAGTTGCCGGCTGTACGGTGGGTTGGTGGACCGGAA
ATCGAGGTATGTGCTTTCAACCCCCAAATACACCCTAACGTGTCTTGCGTAGTTGATCG
CTATCGCCACCAATGGTCGCATCGTCCCCCGATTCGAGGATCTCACCCCAGAAAAGCTC
GGCAAAGCAGGGATTGTTCGCGAAGTGTCTTTTGGTACCACTCG

>Cct5 (MS422) _*Boletus edulis*
GGTGAAAAACACGGGTGCCAACCTGGTTATATGCCAGTGGGGCTTTGACGACGAGGC
AAATCATTTACTAATGCAAAACGAGTTGCCAGCTGTACGGTGGGTTGGCGGTCCCGAG
ATTGAGGTAACGTCCCTTTCATCACAATGTCCGGTTGTAACCTCCCTCTCAGCTAATTGC
TATAGCCACACACGGCCGCATCGTGCCCCGATTCGAGGAACTCACGGCAGAAAAACTA
GGAAAAGCAGGCATTGTACGCGAAATTACCTTTGGCACGACGCG

>Cct5 (MS422) _*Coprinopsis cinerea*
GGTGAAAGATACTGGAGCGAATTTGGTCATTTGCCAATGGGGCTTTGATGATGAAGCC
AATCATTTGCTTATGCAGAATGAGTTGCCGGCTGTGAGATGGGTTGGTGGGCCTGAGAT
TGAGGTGCGTTTTTTGTATTGTAGATCGATTGAACTGGAGGATGTAGTTGATCGCGCTT
GCGACTGAAGGAAGGATCGTACCCCGATTCGAGGACCTCAGCGCTAAAAAGCTCGGT
TCGGCTGGTATCGTTAGGGAACTCTCCTTTGGTACCACCCG

>Cct5 (MS422) _*Agaricus bisporus* var. *bisporus*
GGTAAAGGATACAGGTGCTAATCTGGTTATCTGTCAATGGGGTTTTGATGACGAAGCAA
ATCATCTCTTACTTCAAAATGAGCTTCCTGCCGTACGTTGGGTCGGCGGCCCCGAAATC
GAGGTATTTGCTTGATATCACAAGTCTACCGCTCATTCGGATTTAGTTGATTGCGATTGC
CACGCAAGGTCGCATCGTCCCTCGTTTCGAAGATCTCACAGCGGCAAAGCTTGGGACT
GCCGGTGTGGTGCGCGAGCTCACCTTTGGTACCACAAG

>Cct5 (MS422) _*Amanita muscaria*
GGTGAAAGATACAGGAGCGAATTTGGTTATTTGCCAGTGGGGTTTTGATGATGAGGCTA
ATCACCTTCTCATGCAAAACGATCTGCCTGCCGTCCGGTGGGTGGGCGGTCCCGAAAT
TGAGGTGCGTTCAGCATAACGATAACGAACTAAACGACGCATATACCTAGTTAATTGCC
ATTGCTACTCAAGGCCGCATTGTCCCCCGGTTTGAAGATCTGACCAGCGAAAAGTTGG

GCAAGGCTGGAATTGTTCGTGAGGTCACCTTCGGCACGACACG

>Cct5 (MS422) _*Marasmius fiardii*
GGTGAAGGACACGGGCGCAAATTTGGTTATATGTCAATGGGGATTTGACGATGAAGCA
AATCATCTGCTAATGCAGAACGATTTACCAGCCGTACGGTGGGTCGGTGGCCCGAAA
TTGAGGTATGGATTTTCAAGCATACCTATTGCTTGATACTCGATTCCCAGCTCATCGCAA
TTGCCACACAAGGTCGTATCGTACCCAGATTTGAAGATCTCACCGCTGCAAAACTTGGT
AAAGCCGGAATCGTAAGAGAAGTGACGTTTGGTACCACAAG

>Cct5 (MS422) _*Fomitiporia mediterranea*
AGTGAAAGATACTGGTGCTAATCTTGTCATTTGTCAATGGGGTTTCGACGATGAGGCGA
ATCATTTGCTTATGCAGAATGAATTGCCGGCTGTGAGATGGGTTGGTGGACCCGAGATT
GAGGTTTGCTTTTTCCCTTCAGCGACGTTTAATGTTAATTTGCAAGCTCATCGCCATTGC
TACAAACGGCCGAATCGTACCTCGATTTGAGGATCTGACATCAGAAAAACTTGGCAAG
GCAGGGATCGTACGTGAAATTACCTTTGGCACCACGAA

>Cct5 (MS422) _*Gymnopilus chrysopellus*
AGTGAAGGACACCGGCGCCAACTTGGTCATTTGTCAATGGGGTTTCGATGATGAGGCC
AACCATTTGTTGATGCAAAATGATCTTCCTGCTGTTCGATGGGTCGGAGGTCCTGAGAT
CGAGGTGAGTATTCTTGAGCCCTGCGCACTCACCGCAATCTTGAAAGTTAATCGCTATC
GCCACTCAAGGAAGGATTGTTCCTCGATTTGAAGATCTTTCCGCGGAGAAGCTTGGAA
GAGCTGGCGTCGTACGAGAAATTGGGTTCGGTACAACCCG

>Cct5 (MS422) _*Lactarius quietus*
CGTGCAAGATTCGGGCGCGAACCTAGTCATTTGCCAGTGGGGCTTCGACGACGAGGCG
AACCACCTATTAATGCAAAACGACCTTCCGGCCGTCCGATGGGTCGGCGGGCCTGAAA
TCGAGGCAAGTGGTTGTGTCAGTCGACTAGCGAACACCGTGCGATCACCAGCTCATTG
CCATCGCTACCGAGGGTCGCATCGTCCCTCGATTCGAGGACCTGACACCAGAGAAGCT
CGGAAAGGCGGGAGTTGTCAGAGAAGTCACGTTTGGGACCACGAG

>Cct5 (MS422) _*Gautieria morchelliformis*
AGTCAAAGACACAGGGGCCAATCTGGTCATCTGCCAGTGGGGCTTTGACGACGAAGC
AAACCACTTACTTCTACAAAATGAGCTCCCTGCTGTTCGTTGGGTCGGAGGCCCTGAA
ATTGAGGTGCGGTGTATTTCTGTTTTGCGACATCTGTCGGTTTTCATTGTAGCTTATCGC
GATTGCCACCCAGGGACGTATCGTCCCTCGGTTTGAGGATCTGACAGCTAAGAAATTA
GGCAAGGCGGGTATTGTCCGCGAAGTCTCCTTCGGGACAACACG

>Cct5 (MS422) _*Ramaria acris*
GGTCAAAGACACCGGTGCCAACCTCGTTATATGCCAGTGGGGGTTTGACGACGAAGCA
AATCACTTATTGTTACAAAATGAGCTTCCTGCCGTACGATGGGTTGGAGGCCCGGAGAT
TGAGGTGCGAATTGATTTCACTTGTTGAAAAACGCCAATATGTGGTGTAGCTCATTGCT
ATAGCCACACAAGGTCGCATTGTTCCTCGGTTCGAGGACCTTACTGCCAAAAAATTGG
GCAAGGCTGGTATTGTGCGAGAAGTTTCATTTGGTACAACAAG

>Cct5 (MS422) _*Pluteus cervinus*
AGTCAAGGATACCGGGGCAAACCTTGTTATCTGCCAGTGGGGATTCGATGACGAAGCA
AATCATTTGCTAATGCAAAACGACTTACCCGCAGTTCGATGGGTAGGGGGTCCAGAAA
TCGAGGTAGGTCAAGGGCCAAACTTCTAACGCGATAAACAGCTCATCGCCATTGCTAC
ACAAGGGCGCATCGTACCCAGATTTGAGGATCTGACTAAAGAGAAGCTCGGAAAGGC
TGGGCTCGTACGCGAAGTCACCTTTGGGACAACTCG

>Cct5 (MS422) _*Gymnopus androsaceus*
GGTCAAGGATACTGGCGCGAATTTGGTCATTTGTCAATGGGGTTTCGACGACGAAGCT
AACCATATGCTGATGCAAAACGATCTCCCTGCTGTGCGATGGGTTGGTGGTCCTGAGAT
CGAGGTGAAGCTGTTACAACAATACTGAGAATATCGCTAGCTTATCGCGATCGCTACCC
AAGGTCGCATCGTTCCCCGATTTGAAGACCTAACGGCGGCGAAACTTGGTAAAGCCGG
TATCGTCAGGGAAGTCTCCTTCGGTACCACCAG

>Cct5 (MS422)_*Rhizopus microsporus*
AGTTAAGGACTCTGGTGCCAACATGGTTGTCTGTCAGTGGGGTTTTGATGACGAAGCC
AACCACTTACTCTTACAGAACCAATTGCCTGCTGTTCGTTGGGTGGGTGGTCCCGAAAT
TGAGTTGATTGCAATCGCCACCAATGGTCGCATCGTACCCCGTTTTGAAGACCTTGCAC
CTGAAAAGCTAGGTCATGCTGGTGTTGTACGTGAATTGACATTTGGTACTACCAA

>Cct5 (MS422) _*Agaricostilbum hyphaenes*
TGTCAAGAACGCTGGAGCCAACCTCGTCATTTGCCAGTGGGGCTTTGACGACGAAGC
CAATCATCTGCTCATGACCAACAACTTGCCAGCTGTACGATGGGTGGGCGGCCCAGAA
TTGGAACTTATCGCCATTGCTACCAACGGCCGCATTGTGCCTCGATTTGAAGACCTCAC
TGAAGCCAAGCTCGGCAAGGCGGGTTCAGTCAAGGAGCTCGCATTTGGTACAACAAA

>Cct5 (MS422) _*Asperigillus triticus*
CCTGAAGAACTCCGGTGCCAACCTGGCGATCTGCCAGTGGGGCTTTGATGACGAAGCC
AACCACCTGCTGCTCCAGAACAAGCTTCCCGCTGTCCGTTGGGTCGGCGGTCCTGAGA
TCGAACTGATCGCCATTGCGACCAACGGCCGCATCGTCCCTCGGTTCGAGGATCTGAG
CGCGGACAAGCTAGGATCCGCCGGTAAAGTGCGAGAGATGACCTTTGGCACCACCCG

>Elp3 (FG533)_*Amanita muscaria*
ATCTGTGTGTATGTGGATCAATGACTATACTCAACACATTTTGACCGTTATTGCAGCTAC
TGTCCTGGAGGTCCAGATTCGGATTTTGATTACAGCACACAAAGCTATACGGGCTATGA
ACCAACAAGTATGCGAGCCATTCGCGCTCGCTACGACCCATACGAGCAGACACGAGGG
AGAGTCGAGCAGTTGAAAAGCTTGGGCCACAGTGTGGATAAGGTACGTTAGGTCTTGC
TTTTAAAGAGCTTCGATCTCATTCATACTAGGTCGAATTCATCATTATGGGAGGAACA

>Elp3 (FG533)_*Hygrophoropsis aurantiaca*
ATTTGTGTGTGAGCACAACCGACCATGGAGCGATCTTTTATAAGATTCGTAGATACTGC
CCGGGAGGTCCAGATTCAGACTTCGACTACAGTACACAGAGTTACACGGGCTACGAGC
CCACGAGTATGCGCGCGATCCGCGCACGTTATGACCCATACGAACAAACTCGAGGTCG
TGTCGAGCAGTTGAAAGCTCTCGGACATAGTGTTGACAAGGTAAGCCATATCAGCCTC

GAAAACGCTTTGTTTTCAAAAAAAGTGTGCCCCAGGTCGAGTTCATCATCATGGGCG
GGACT

>Elp3 (FG533)_*Mycena crocata*
ATATGCGTTTACTGCCCCGGTGGGCCCGACTCGGACTTTGACTATAGCACGCAGAGCTA
CACGGGGTACGAGCCGACGAGCATGCGCGCGATCCGCGCACGGTACGACCCGTATGA
GCAGACGCGGGGACGTGTGGAACAACTGAAAGCCCTAGGACATAATGTCGATAAGGT
GCGCGGAGCCATTGTTATTGAGACAAAAACTCATAATTGAGGCAGGTCGAGTTTATTGT
CATGGGCGGGACG

>Elp3 (FG533)_*Schizophyllum commune*
ATCTGCGTGTGCGTTCTACATACCTTGTCGAGAGATCGGAAAAATAGGTACTGCCCGGG
AGGACCGGACTCGGACTTCGACTACAGCACACAGAGCTATACTGGCTATGAGCCGACA
AGTATGCGAGCCATCCGCGCGCGGTACGACCCGTACGAGCAGACGCGAGGGCGAGTG
GAGCAGCTGAAGGCTTTGGGGCATAGTGTGGACAAGGTGCGAGGTCCGACTCACGGG
CAGAAAGTTTTGCTGACCATATACAGGTCGAGTTCATCATCATGGGTGGTACC

>Elp3 (FG533)_*Coprinopsis cinerea*
ATCTGTGTGTAAGTTGGACCTATTCGTTTTCCATGAGCAGCCTTTAACCTTTGACAGATA
TTGTCCTGGAGGACCCGATTCAGACTTCGATTACAGTACACAAAGTTATACCGGGTACG
AACCAACGAGCATGCGAGCTATTCGTGCTCGATACGATCCTTATGAGCAAACGCGAGG
GCGAGTGGAGCAGTTGAAGAGCCTGGGACACAGTGTCGATAAGGTGAGATCCAACGT
CGTGGTATCTGGGAAAGCAAACTCATGCAGTGGCGACACAGGTCGAGTTCATTGTCAT
GGGAGGCACC

>Elp3 (FG533)_*Gautieria morchelliformis*
ATATGCGTGTAGGTCTCAGTCCGCGTTTCAAATATCGAACCAATACAAAGATATTGTCCT
GGCGGACCGGACTCAGATTTCGACTACAGTACACAGAGTTATACCGGTTATGAGCCCA
CCAGCATGCGTGCCATCCGCGCACGGTACGATCCATATGAGCAGACACGGGGTCGCGT
CGAACAGCTTAAAAGTCTGGGTCACAGTGTCGATAAGGTTTTCTTTTTCTCCTGAAAAA
TCGCACAAGGCGTAATAATTATCGTGCTCAGGTAGAGTTTATTGTCATGGGCGG
CACT

>Elp3 (FG533)_*Ramaria acris*
ATATGCGTGTATGTCCGGACTACGCAGTTTCAAGCATTAAACCCATGTCAAGCTATTGTC
CCGGAGGACCGGATTCAGACTTCGATTATAGCACACAGAGCTATACCGGCTATGAGCCT
ACAAGTATGCGTGCCATTCGTGCTCGATACGATCCGTATGAACAGACACGGGGTCGGGT
AGAACAACTTAAAAGCTTAGGTCATAACGTCGACAAGGTAATTTATATTTTCGTAATCCT
CATATTATTCATGGTGACATATTTAGGTGGAGTTTATTATTATGGGCGGTACT

>Elp3 (FG533)_*Stereum hirsutum*
ATATGTGTGTATGTCGATCTCCCATCTATCCGTTTGTCCTATTCATTACGTATATAGCTATT
GTCCAGGTGGCCCCGACTCAGACTTCGACTACAGCACACAGAGTTATACAGGTTACGA

ACCAACCAGTATGCGTGCCATCCGAGCGCGTTACGATCCTTATGAACAGACGAGAGGA
CGGGTGGAGCAGCTGAAGGCGTTGGGCCACAGTGTTGACAAGGTGAGCAGATTCGTG
TCACGAGGAAGTTCGCGTTCTGAGGACTGTATTCAGGTCGAGTTCATCATCATGGGCG
GTACA

>Elp3 (FG533)_*Fomitiporia mediterranea*
ATTTGCGTGTATGCTCTCCATCTCTTCTACTTACTGCCCATCCACCATATTTTCAGTTATT
GTCCGGGTGGCCCAGACTCTGACTTCGATTACAGTACGCAGAGCTATACAGGTTATGAA
CCAACTAGTATGCGCGCGATCAGAGCGCGTTACGACCCCTACGAGCAGACTAGAGGAC
GGGTGGAACAATTGAAGAGTTTAGGTCATAGTGTCGATAAGGTAAGCTTGTGTGGCAT
CCGTGAGGCTTCATGGCTCTGACGTGATGGCATTCAGGTTGAATTTATCATCATGGGTG
GTACT

>Elp3 (FG533)_*Lactarius quietus*
ATATGCGTGTGCGCCGAGTCTATTCACCGTATCTTACCCACCCTTGCGGTAGATATTGCC
CAGGCGGCCCAGACTCCGACTTTGATTATAGCACGCAAAGCTACACCGGGTACGAGCC
CACGAGCATGCGCGCCATCCGTGCTCGGTATGATCCATATGAGCAGACGCGAGGGCGG
GTGGAACAGTTGAAGAGTTTGGGACACAGCGTTGATAAGGTGTGCTTTCCCCCACGAG
ACTGCTCTTACATGACCCATGTAGAAAAACAAAGGTCGAATTCATTATCATGGGCGGGA
CA

>Elp3 (FG533)_*Serpula lacrymans* var. *lacrymans*
ATATGTGTGTTAGTATGAATAGTGCGGATACTCACAACAATCTTACATTCTCTTCAGATA
CTGCCCAGGAGGACCAGATTCAGATTTTGATTATAGCACTCAGAGCTATACTGGCTATG
AGCCAACGAGCATGCGTGCTATTCGTGCGCGATATGACCCTTATGAGCAAACGCGTGGT
CGAGTAGAGCAGCTAAAGAGTTTAGGTCACAATGTAGACAAGGCAAGTCACGATGAC
ACTTTATGGCAGCTCCTATCTTGACGATTGTACTTAGGTCGAGTTTATCATCATGGGTGG
AACA

>Elp3 (FG533)_*Gymnopilus chrysopellus*
ATATGTGTGTGCGTAGTCTTCCTCGAGCTTTTTGGCTATTTCCTTGCACTTTAGATACTG
CCCCGGAGGTCCGGATTCCGATTTCGATTACAGCACACAGAGTTACACAGGATATGAG
CCGACAAGTATGCGTGCTATCAGAGCACGATACGACCCTTACGAGCAAACGAGAGGAC
GAGTGGAGCAGCTGAAGAGTTTAGGACACAGCGTTGACAAAGTATGATCTTAAAATAT
GGCATTGTTCATTTTACTGATCTTTTCTTTTTCTAGGTCGAGTTCATTGTCATGGGAGGC
ACC

>Elp3 (FG533)_*Fomitopsis pinicola*
ATCTGCGTGTGAGTAAGTGCGGTTACTCTACCGGATGCATGCCGACTATGTGCAGATAT
TGTCCAGGAGGTCCCGACTCGGACTTCGACTACAGCACGCAGAGTTACACGGGCTACG
AACCTACGAGTATGCGAGCGATCAGGGCGCGGTATGACCCTTACGAGCAGACGCGTGG
TCGAGTCGAGCAGCTCAAGAGCCTGGGACACAATGTGGATAAGGCACGTCTGCTACA
CGTAATACCTTCACGCTCTATTGACTTCCCGGGCAGGTCGAGTTCATCATCATGGGTGG
CACG

>Elp3 (FG533)_*Trametes versicolor*
ATCTGTGTGTGCGTAAAACACACGCCGTCCTGAACTGCTGATTTAATTTGCCTCTAGGT
ATTGTCCAGGAGGGCCCGACTCGGACTTTGACTACAGCACCCAGAGCTATACTGGATA
CGAGCCGACCAGTATGCGAGCTATCAGAGCGCGGTACGATCCGTATGAGCAGACACGA
GGTCGAGTAGAGCAACTCAAAAGCCTCGGGCATAACGTCGACAAGGTGGGATCTAATG
TCGCCCGTGTTGGCCTTTTTCACTTAATATATTGCGCAGGTCGAATTTATTGTCATGGGA
GGCACG

>Elp3 (FG533)_*Laccaria bicolor*
ATATGCGTGTAATTTTTTTTTCTGGGATGATATCGACGAGTTTATCGGCTAGGTACTGTCC
TGGAGGACCTGACTCTGATTTTGACTACAGCACGCAGAGCTATACTGGATACGAACCG
ACGAGTATGCGTGCCATTCGGGCGCGGTACGACCCCTACGAACAAACGCGAGGGAGG
GTTGAGCAGTTGAAAAGTCTGGGGCATAACGTTGACAAGGTATATGCTAGTCCACTCC
ATACCATTCACCTCACTGATTTGCCACAAGGTTGAGTTCATCGTCATGGGAGGGACA

>Elp3 (FG533)_*Agaricus bisporus* var. *burnettii*
ATATGTGTGTGCGCTCAAAAATTTCTTTCATCTTGGTTCTCGCGAACAACGACCAGATA
TTGTCCAGGAGGACCTGATTCGGACTTCGACTACAGTACGCAAAGCTACACCGGATAC
GAACCGACAAGTATGCGTGCCATTCGTGCTCGATATGACCCGTACGAACAGACGAGAG
GGCGAGTGGAACAGTTGAAGAGTCTGGGCCATAACGTTGATAAAGTAACGTCGGCTAC
CATCTTGGATACAATTTCAATCTGATGATTTCTAGGTTGAATTCATCATCATGGGTGGGA
CA

>Elp3 (FG533)_*Agrocybe pediades*
ATTTGCGTGTACGTGGGCACAAATGTTCTGAGGAGGGATGATTGACAATGTCCTAGGTA
TTGCCCAGGAGGACCCGACTCCGACTTTGATTACAGCACACAAAGTTACACAGGATAC
GAGCCAACGAGTATGCGAGCCATCCGTGCGCGATATGACCCTTATGAACAAACAAGAG
GAAGAGTGGAGCAGCTGAAGAGTCTGGGACATAGTGTGGACAAGGTAAGGACTTCAT
TCTCTTCTGCTTTTCATACCTTTAACGCTTGGAGTTAGGTCGAATTTATCGTTATGGGTG
GTACA

>Elp3 (FG533)_*Pluteus cervinus*
ATATGCGTGTAAGTTCTATCCTTTGACTGCGAAGCGCGTCTTGATTGTGGTTCTAGCTAT
TGCCCTGGCGGTCCGGATTCAGATTTCGACTACAGCACTCAAAGTTATACTGGCTACGA
ACCCACGAGTATGCGTGCGATACGTGCCAGATACGATCCTTATGAGCAGACACGGGGA
CGAGTTGAGCAATTGAAGAGTCTAGGACATAGTGTGGATAAGGTGCTTATGAACCAAA
CATTGACCGGCATACGCACTAATTCCTTGCCCTTTAGGTTGAGTTCATCATCATGGGTGG
TACA

>Elp3 (FG533)_*Boletus edulis*
ATCTGTGTGTGAGTGCGCTTGATGTTGAAAGTGACATATTCCTCGACGTCGCGTAGATA
TTGCCCAGGAGGACCTGACTCTGATTTTGATTACAGCACACAGAGCTACACTGGATAC

GAGCCTACGAGCATGCGTGCCATCAGGGCCCGTTACGACCCATACGAACAAACACGGG
GTCGCGTCGAGCAGTTGAAAGCCCTTGGCCATAGTGTCGACAAGGTCCGTCTCGTCAT
TCTTCTCCACTGTGGGTATCCATTCAAAGCATGCTACCAAGGTTGAATTCATCATAATGG
GCGGCACG

>Elp3 (FG533)_*Tricholoma matsutake*
ATATGCGTGTACGTGTTACGTGTTACGTGTAGGTCAGCTTGAGCGATTGTTGTTTAGATA
TTGCCCTGGAGGCCCAGACTCGGATTTCGACTATAGTACTCAGTCATATACGGGGTACG
AGCCCACGAGTATGCGTGCGATCCGTGCTCGATATGATCCGTATGAGCAAACTCGGGGG
CGTGTGGAACAGCTGAAGAGTTTAGGTCATAGTGTGGATAAGGTATGCTTCTTTTAATT
ATCGCACTCGTCTTTGAATAAAGGCCATTTTTAGGTCGAATTCATAGTGATGGGAGGGA
CA

>Elp3 (FG533)_*Auricularia delicata*
ATCTGCGTGTCAGTGAAGCCCGCATACCGAAAAACCCATAGAAGCATGACAGCTATTG
TCCCGGCGGCCCCGACTCAGACTTCGATTACAGCACACAAAGCTACACAGGCTACGAG
CCTACCAGTATGCGTGCCATCCGTGCACGCTACGACCCGTACGAACAAACGCGCGGCC
GCGTTGAGCAGCTAAAGTCGCTCGGCCATAGCGTAGACAAGGCGCGTGTATGCGCATA
CAGCCCGCTCCACCTCGCCTAACCTGCACTAGGTCGAGTTCATTGTGATGGGCGGGAC
G

>Elp3 (FG533)_*Calocera cornea*
ATTTGCGTGTACGCTCTCCCCCCCCCGCTGCCTTGCTCAGCAGGTCCCGCAACAGGTAT
TGTCCCGGTGGCCCGGACTCCGACTTCGACTACAGCACCCAGTCGTACACGGGCTACG
AGCCCACAAGTATGCGCGCCATCCGAGCGCGGTACGATCCCTACGAGCAGACACGAGG
TCGGGTGGACCAGCTTGCCAGTCTGGGGCACAGCGTCGACAAGGTCGAGTTCATCATC
ATGGGAGGGACG

>Elp3 (FG533)_*Dacryopinax primogenitus*
ATCTGTGTTTACTGTCCCGGTGGTCCCGACTCAGACTTTGACTACTCCACCCAGTCTTA
CACGGGGTATGAGCCCACCAGCATGCGTGCGATCCGAGCACGGTACGACCCATACGAA
CAAACCAGGGGAAGAGTCGAGCAGCTCGCGAGCTTGGGTCATAGCGTTGACAAGGTC
GAGTTCATTATAATGGGCGGGACG

>Elp3 (FG533)_*Gymnopus androsaceus*
ATCTGCGTGTGCGTGTATTATTTCTTTCATCGAGCATTGAAGTTATCCTCAGGTACTGCC
CTGGTGGTCCAGATTCGGATTTTGATTACAGTACGCAAAGTTACACTGGGTATGAGCCT
ACAAGTATGCGGGCTATCCGAGCTAGATACGATCCATATGAACAGACAAAAGGACGAG
TCGAGCAATTGAAAAGTCTTGGGCATAGCGTGGACAAAGTTCGCTCACTCTCTTTCCAT
TCGAAGCCCTAACTCAATCTATATACTTAGGTCGAATTCATCATCATGGGAGGCACG

>Elp3 (FG533)_*Neurospora crassa*
ATTTGCGTCTACTGCCCCGGTGGTCCAGACTCCGACTTCGAATACAGCACACAGTCATA
CACGGGATATGAGCCGACATCGATGAGAGCCATCCGGGCTCGTTACGACCCCTTCGAG

CAGGCGAGAGGACGAGTTGATCAGCTAAAGTCATTGGGCCACTCGGTCGACAAGGTC
GAATACATCATCATGGGCGGCACT

>Elp3 (FG533)_*Asperigillus triticus*
ATCTGCGTGTACTGCCCCGGCGGGCCCGACTCCGATTTCGAATACTCGACGCAATCCTA
CACCGGGTACGAGCCGACGTCGATGCGGGCCATCCGGGCGCGGTACGATCCGTTCGAG
CAGGCGCGCGGGCGGGTGGACCAGATCAAGTCGATGGGCCACAGCGTGGACAAGGTC
GAGTACATCATCATGGGCGGCACG

>Elp3 (FG533)_*Rhizopus microsporus*
ATTTGTGTCTACTGCCCTGGTGGCCCTGACTCTGATTTTGAATATTCAACACAGTCATAT
ACTGGATACGAGCCTACGTCCATGCGAGCCATTCGTGCTCGTTATGATCCCTATGAACA
AGCCCGTGGTCGTGTGGATCAACTTCGAAGCTTGGGTCACAATGTCGATAAGGTAGAA
TACATCATTATGGGTGGCACG

>Tcp1 (FG850)_*Laccaria bicolor*
GTACGTCGGTGCAGGAAAGAAGATCTGCGCCGGATTGCTAAAGCGACGGGTGGCCAA
CTTATATCCAGTCTCGCGAATCTGGAGGGAGAAGAAACCTTCGAAGCCAGCTATCTGG
GGACTGCCGACGAGGTGGTGCAGGAGCGCATCTCGGACGACGAACTGATTCTTATTAA
GGGGACGAAGGTCGTAAACTCAGCATCGATTGTCCTTCGGGGAGCAAAGATTACATGT
TGGATGAGATGGAACGAGCTTTGCATGACACACTGTCGATCATCAAGCGAACATTGGA
GAGTGGTTCTGTGGTACCTGGTGGCGGTGCTGTCGAATCGGCGTTGAGCATCTACCTG

>Tcp1 (FG850)_*Tricholoma matsutake*
GTGCGTCGGTGTAGAAAAGAGGATTTGCGTCGGATCGCGAAGGCGACCGGGGGTACA
CTGGTTTCCAGTCTTGCGAACCTGGAGGGTGAGGAGACATTTGAGGCGAGCTATCTGG
GAACTGCGGACGAGGTCGTCCAGGAGCGCATATCGGATGATGAGCTGATCTTGATCAA
GGGAACCAAGGTGGTCAACTCATCTTCGATTGTGCTGCGAGGAGCAAAGACTATATGC
TGGATGAGATGGAGCGAGCACTTCATGACACCCTGTCGGTCATCAAGCGGACCCTGGA
AAGCGGGTCTGTAGTCCCCGGCGGCGGAGCGGTGGAAACCGCTTTGAGTATTTATTTG

>Tcp1 (FG850)_*Mycena crocata*
GTTCGCCGTTGTAGGAAAGAGGACCTTCGTCGCATCGCCAAAGCCACTGGAGGCACG
CTAATTTCTAGTCTCGCGAATCTCGAAGGGGAAGAGACGTATGAAGCTAGCTATCTTGG
TTCTGCGGACGAAGTCATTCAAGAGCGCATCTCGGACGACGAACTCATCCTCATCAAG
GGCACCAAGGTCGTCAACTCCGCGTCTATTGTTCTACGAGGTGCGAAGACTATATGCTC
GACGAGATGGAGAGGGCGCTGCACGACACTCTATCGGTTATAAAGCGCACACTCGAAA
GTGGTTCGGTTGTGCCTGGCGGGGGTGCCGTTGAATCTGCACTGAGTATCTATTTG

>Tcp1 (FG850)_*Phanerochaete carnosa*
GTCCGCCGGTGTCGCAAGGAAGATCTCCGCCGCATCGCGAAGGCGACTGGTGGTACCC
TCGTATCGAGTCTTGCTAACCTCGAAGGGGAGGAGTCGTTCGAAGCTAGCTACCTTGG
TCATGCCGAGGAGGTCATCCAGGAGCGCATTTCCGATGATGAGCTAATCCTCATCAAAG
GCACGAAGGTGGTCAACTCGGCGTCGATAGTGCTTCGTGGCGCGAAGACTATATGCTG

GACGAGATGGAACGAGCTTTGCACGATACGCTTTCCATTATCAAGCGTACGCTTGAGA
GTGGCTCTGTAGTTCCAGGCGGAGGTGCTGTGGAGACCGCTCTGAGCATCTATCTT

>Tcp1 (FG850)_*Schizophyllum commune*
GTCCGCAGATGTCGCAAGGAGGACCTGCGCCGAATTGCGAAGGCCACTGGCGGTACC
TTGGTGTCGAGCTTGGCAAACCTCGAGGGCGAGGAGACCTTCGAGCCCAGTTACCTC
GGTACCGCGGACGAGGTCGTGCAAGAGCGCATCTCGGACGACGAGCTTATCTTAGTCA
AGGGCACAAAGGTCGTCAGCTCGTCCTCTATCATCCTCCGCGGCGCCAAGACTACATG
CTCGACGAAATGGAGCGTGCTCTGCATGACACTCTTTCCATCATCAAGCGGACGTTGG
AGAGCGGCGCCGTTGTCCCCGGTGGCGGAGCCGTCGAGTCTGCCCTCAGCATATACCT
C

>Tcp1 (FG850)_*Trametes versicolor*
GTCCGCAGGTGTAGGAAGGAGGACTTGCGCCGCATCGCGAAGGCGACCGGCGGTACT
CTGGTCTCCAGTCTTGCCAACCTTGAGGGCGACGAGTCTTTCGAGGCTAGCTTCCTCG
GCCACGCCGACGAGGTTGTTCAGGAGCGCATCTCTGACGACGAGCTCATCCTCATCAA
GGGCACGAAAGTCGTCAGCTCGGCTTCCATTGTGTTGCGCGGCGCGAAGACTACATGC
TCGACGAAATGGAGCGTGCGCTCCATGACACCTTGTCAATCATCAAACGGACCCTGGA
GAGTGGTTCCGTGGTTCCCGGCGGTGGCGCCGTCGAGACAGCCCTCAGCATCTACCTA

>Tcp1 (FG850)_*Stereum hirsutum*
GTTCGTCGGTGTAGAAAGGAGGACCTCCGTCGTATTGCGAAGGCAACCGGTGGTCAA
CTCGTTTCGAGTCTTGCAAACCTGAACGGCGACGAGACCTTCGAGGCGAGCTTCTTGG
GTACCGCGGAGGAAGTCGTGCAGGAGCGGATATCAGATGACGAGCTGATACTGGTCAA
GGGCACGAAGGTCGTCAGCTCTGCGTCGATCATCTTGCGTGGCGCGAAGATTATATGTT
AGACGAGATGGAGAGGGCTCTGCACGATACGCTTTCGATTATCAAGCGCACGCTCGAG
AGTGGCTCTGTAGTTCCTGGAGGAGGAGCGGTGGAGAGTGCGCTGAGCATCTACCTG

>Tcp1 (FG850)_*Gymnopus androsaceus*
GTTCGCAGATGTCGAAAGGAAGATCTACGAAGGATCGCAAAGGCTACAGGCGGTCAA
CTGATTTCTACTCTTGCCAACTTGGAGGGAGAGGAGAGTTTCGAAGCTAGTTATCTCGG
GACGGCGGAAGAAGTTGTTCAAGAGCGAATTTCGGACGACGAGCTCATCTTGATCAA
GGGCACGAAAGTCGTCAACTCCTCATCAATCGTCCTCCGAGGTGCAAAGACTACATGC
TCGATGAAATGGAGAGAGCGTTACACGATACCCTTTCCGTTATCAAGCGGACTTTGGA
GAGTGGTTCCGTGGTTCCTGGTGGTGGTGCTGTAGAATCCGCACTGAGCATCTACCTG

>Tcp1 (FG850)_*Lactarius quietus*
GTCCGCCGTTGCAGAAAAGAGGATCTTCGCCGCATTGCCAAGGCCACTGGTGGGCAG
CTTGTCTCGAGCCTGGCAAATATGGATGGAGAAGAGACATTTGAGCCGATCTATCTTGG
CACAGCGGAAGAGGTCGTGCAAGAGCGCATTTCGGACGATGAACTCATCTTAGTAAAG
GGAACGAAGGTGGTCAACGCGGCCTCTATCATTCTCCGCGGTGCCAAGATTACATGCT
CGACGAGATGGAACGCGCATTACACGACACATTATCTGTGATCAAGCGGACATTAGAG
AGCGGGACCGTTGTGCCTGGCGGAGGTGCCGTAGAGTCCGCACTAAGCATCTATCTG

>Tcp1 (FG850)_*Agaricus bisporus* var. *bisporus*
GTCCGCCGTTGTAGAAAGGAAGATCTTCGTCGGATTGCTAAAGCCACCGGTGGCCAAC
TCATCGCTAGTATGGCCAATCTGAACGGAGACGAGACCTTTGAAGCCAGCTATCTTGGC
ACTGCCGATGAAGTTGTGCAAGAACGCATTTCAGACGATGAACTCATATTAGTTAAAG
GGACAAAAGTGGTTAATTCCGCATCGATCGTACTTCGTGGAGCCAAGATTTCATGTTGG
ACGAGATGGAGAGGGCACTTCATGACGCATTATCTATTATCAAACGAACATTAGAGAGT
GGTGCCGTTGTTCCGGGCGGAGGCGCAGTTGAATCGGCCCTGAGCATCTACCTG

>Tcp1 (FG850)_*Hygrophoropsis aurantiaca*
GTCCGCCGCTGTCGCAAAGAGGATCTGCGCCGCATTGCAAAGGCTACTGGCGGAACTC
TGGTGTCCAGCCTTGCTAACCTTGAAGGCGATGAGACCTATGAAGCGAGTCTACTAGG
CACCGCCGATGAAGTCATCCAGGAACGCATCTCCGACGACGAATTGATACTGATCAAG
GGCACGAAAGTGGTCAACTCTGCATCCATTGTTTTGCGTGGTGCTAAGACTACATGCTC
GATGAAATGGAGAGAGCACTACATGATACGCTGTCTATTATCAAACGGACGTTGGAGA
GTGGCTCAGTTGTTCCTGGTGGAGGTGCAGTGGAATCTGCATTGAGTATCTACCTT

>Tcp1 (FG850)_*Serpula lacrymans* var. *lacrymans*
GTACGTCGTTGTCGGAAGGAAGATCTCCGCCGTATTGCGAAGGCAACAGGCGGAACCA
TGATTTCAAGTCTTGCCAATCTCGAAGGAGAGGAAACATACGAGTCGAGTTATCTAGG
TTATGCCGACGAAGTCATTCAAGAGCGTATCTCGGATGATGAACTCATTTTGATCAAGG
GAACAAAAGTTGTGAGCTCGGCGTCCATTGTCCTACGAGGTGCAAAGATTATATGCTG
GATGAGATGGAACGAGCATTGCACGATACGTTGTCTGTGATCAAGCGTACTTTAGAAA
GTGGCGCCGTTGTCCCTGGTGGAGGGGCAGTGGAGTCTGCTTTGAGCATTTACCTT

>Tcp1 (FG850)_*Boletus edulis*
GTGCGTCGTTGCCGCAAGGAGGATCTCAGGCGTATCGCAAGAGCCACAGGGGGAACC
CTGATTTCGAGTCTCGCAGATCTTGAAGGAAATGAGACTTATGAATCCAGCTACTTGGG
CGTAGCAGAGGAAGTCACACAGGAACGCGTTTCCGACGACGAGCTTATTCTCATCAAG
GGGACAAAAGTTGTCAACTCCGCGTCCATTGTTTTACGTGGCGCGAAGATTATATGCTA
GATGAAATGGAGCGGGCATTGCACGACACATTATCCATCATCAAGCGAACTCTTGAGA
GCGGGTCAGTCGTTCCGGGTGGAGGTGCGGTCGAATCTGCTCTGAGCATCTATCTC

>Tcp1 (FG850)_*Pluteus cervinus*
GTCCGGCGGTGTAGAAAGGAGGACTTGAGGCGGATCGCCAAAGCTACAGGAGGCACG
CTGGTGTCCAGTTTGGCCAACCTTGATGGTGAAGAAACATTCGAACCGAGCTACCTCG
GAACTGCAGATGAAGTCGTGCAAGAGCGGATATCGGATGACGAGCTAATCCTAATCAA
GGGGACCAAGGTCGTGAACTCTGCGAGTATAGTTCTCCGCGGGGCGAAGACTACATGC
TTGATGAAATGGAGCGCGCACTGCACGACACTTTATCCGTGATCAAACGGACACTGGA
GAGTGGTGCTGTGGTCCCAGGTGGGGGTGCTGTCGAATCTGCCTTGAGCATCTATTTG

>Tcp1 (FG850)_*Gymnopilus chrysopellus*
GTACGTCGGTGCAGGAAGGAGGACCTTCGCCGGATAGCCAAGGCTACCGGAGGCCAA
CTGGTTTCCAGTCTGGCAAACTTAGAGGGTGAAGAAACATTCGAAGCCAGCTACCTTG

GTACAGCGGAGGAAGTCGTCCAGGAGCGCATTTCTGACGACGAACTCATTTTGATCAA
AGGCACGAAAGTGGTCAACTCTGCCTCGGTTATTCTTCGAGGTGCAAAGACTACATGC
TGGATGAGATGGAGCGTGCCCTGCATGATACATTGTCGATCATTAAACGGACGCTTGAA
AGCGGGGCTGTTGTGCCTGGTGGCGGCGCCGTAGAATCGGCGCTCAGCATTTATTTG

>Tcp1 (FG850)_*Coprinopsis cinerea*
GTCCGTCGTTGTCGCAAGGAAGACCTCCGCCGTATCGCGAAAGCCACCGGTGGACAG
CTCATCTCCAGTCTTGCCAACTTGGAGGGTGAAGAAACGTTCGAGGCGAGCTATTTGG
GTACTGCCGACGAAGTGATTCAGGAGCGCATTGCGGACGACGAACTCATCCTGATCAA
GGGTACCAAAGTAGTCAGCTCGGCATCGATCATTCTCCGAGGTGCCAAGAATCGATGT
TGGATGAGATGGAAAGGTCGTTGCATGACACTCTTTCTATCATCAAGCGAACGCTCGA
AAGCGGCGCCGTGGTGCCCGGAGGTGGTGCGGTTGAGTCGGCTCTGAGTGTTTATCTT

>Tcp1 (FG850)_*Fomitopsis pinicola*
GTCCGCAGGTGTAGGAAGGAAGACCTGCGTAGGATAGCGAAGGCAACCGGCGGCACC
CTCGTCTCAAGTCTTGCAAACCTTGAAGGCGAAGAATCCTACGAAGCCAGCTACCTTG
GTACTGCTGAAGAAGTGGTTCAGGAGCGGATCTCCGATGATGAGCTCATCCTGGTCAA
GGGCACCAAGGTGGTCAGCTCAGCGTCCATCATCCTTCGAGGCGCGAAGACTACATGC
TTGATGAGATGGAGAGGTCCCTCCATGACGTTCTGTCTATCCTCAAGCAAACACTCGA
GAGCGGAGCTGTGGTTCCCGGGGGTGGTGCGGTCGAGAGCGCACTCTCCATCTATCTA

>Tcp1 (FG850)_*Gautieria morchelliformis*
GTTAGACGTTGCAAGAAGGAAGACCTAAGACGTATTGCCAAGGCAACGGGTGCAACA
CTGGTATCCAGTTTGGCTAATCTCGAGGGAGAAGAGACGTTTGAAGCGAGCAATTTGG
GAATCGCCGAGGAGGTCGCGCAGGAGCGCATATCGGACGACGAACTCATTTTAATTAG
AGGCACCAAAGTCATCAATTCCTCCTCGGTCGTATTGCGAGGTGCCAAGACTACATGCT
CGATGAAATGGAACGGGCATTGCATGACACTTTATCGATCATCAAGCGGACATTGGAG
AGTGGCGCTGTCGTTCCAGGCGGTGGCGCTGTCGAGTCAGCGCTGAGCATTTACCTG

>Tcp1 (FG850)_*Ramaria acris*
GTCAGGCGCTGCAAAAAAGAAGATCTCAGACGTATCGCGAAGGCAACAGGTGCCACA
TTGGTTTCCAGTCTTGCTAACCTTGAAGGGGAAGAAACGTTTGAAACGAGCAATTTGG
GTTCGGCCGAAGAAGTCGTACAGGAACGTATTTCGGACGATGAGCTCATTTTGATCAG
AGGCACTAAGGTCGTCAACTCGGCATCAGTGGTTTTGCGAGGTGCCAAGATTACATGC
TTGACGAAATGGAGCGGGCCTTGCACGATACATTATCCATTATCAAACGGACGTTGGAA
AGTGGCGCTGTAGTCCCTGGAGGTGGTGCCGTTGAGACCGCCCTCAGCATCTATCTT

>Tcp1 (FG850)_*Fomitiporia mediterranea*
GTCCGACGGTGTCGTAAGGAAGACCTTCGTCGTATTGCAAAGGCGACGGGAGCAACG
TTGGTTTCGAGTCTTGCCAATTTGGAAGGTGATGAGACCTTCGAAGCAAGCAATCTTG
GTTTCGCAGAGGAGGTCGTGCAAGAAAGGATATCAGACGATGAGTTGATCCTCGTCAA
GGGGACGAAGGTTGTCAACTCGTCCTCAATCGTGCTCCGCGGTGCGAAGATTACATGT
TGGATGAGATGGAGCGAGCAATACATGATTCATTATCCATCATTAAGCGCACACTGGAG
AGTGGGACTGTTGTTCCAGGTGGAGGTGCTGTCGAGACGGCACTGAGCATTTACCTC

>Tcp1 (FG850)_*Amanita muscaria*
GTCCGTCGGTGCAAAAAGGAAGATTTACGACGCATTGCCAAGGCCACAGGTGGCCAG
CTTGTTTCAAGCCTTGCAAACCTCGAGGGAGAGGAAACCTTCGAACCAAGCTATCTTG
GCATCGCTGACGAGGTTGTGCAAGAGAGGATCGCAGACGATGAACTCATACTTGTCAA
GGGCACCAAGGTGGTTAGCTCGGCGTCTATTGTCCTTCGAGGTGCAAAGACTACATGC
TGGACGAAATGGAAAGGGCAATACATGACACGTTGAGTGTGATTAAACGGACACTTGA
AAGTGGTGCAGTGGTCCCTGGAGGTGGCGCTGTGGAAACTGCGCTAAACATATACCTC

>Tcp1 (FG850)_*Marasmius fiardii*
GTTCGTAGGTGCCGCAAAGAAGACCTACGAAGGATAGCTAAAGCCACTGGGGGTCAA
CTTATCTCGAGTATGGCTAATCTCGAAGGGGAGGAAACCTTTGAAGCTAGTTATCTTGG
GTCGGCGGAGGAGGTTGTACAAGAGAGAATCTCGGATGATGAGCTGATTTTGATAAAG
GGGACAAAGACGGTTAGTTCTGCGTCGATTGTGTTGAGGGGGGCGAAGATTATATGCT
TGATGAGATGGAGAGAGCGATGCATGACACGCTCTCGATTATCAAGAGGACGTTGGAG
AGTGGGGCTGTGGTTCCTGGTGGGGGGGCGGTTGAGAGTGCGCTGAGTATATATTTG

>Tcp1 (FG850)_*Calocera cornea*
GTGCGAAGGTGTAGGAAGGAGGACCTCCGGCGGATAGCACGCGCGACTGGTGCGACT
CTCATCTCCGACATGGGCGATCTGGACGCAAATGAGACATTCGAGGCCTCCTACCTCG
GGACTGCTGAAGAAGTCGTGCAGGAACGCATCTCCGACGACGAGCTCATTCTCGTCA
AGGGTACAAAGGTCGTCAACTCCAGCTCGATCATCTTGCGTGGAGCGAAGACTATATG
CTGGACGAGATGGAGCGTGCATTGCATGACACCCTCTCGATCATCAAGCGGACGCTGG
AGAGCGGGCAGGTGGTGCCTGGTGGTGGTGCGGTGGAGACCGCTTTGAGCATCTATCT
G

>Tcp1 (FG850)_*Auricularia delicata*
GTCCGGCGGTGTAAGAAGGAGGACCTGCGCCGGCTTGCCAAGGCGACCGGCGCGACG
ATGGTCTCCGACCTGACGAGCGTCGAGGGCGAGGAGGTCTTCGACGCGTCCAACCTC
GGCACCGCGGACGAGGTCGTCCAGGAACGGATATCCGACGACGAGTGCATCCTCATCA
AGGGCACTAAGCTCGTTCAAACGTCGTCCATCATCCTGCGCGGAGCGAAGACTACATG
CTCGACGAGATGGAGCGCTCGGTGCACGACGTCTTGTCTGCGCTGAAGCAGGTATTGG
AGAGCGGGGCTGTCGTTGCGGGCGGCGGCGCTACAGAAGTTGCGCTCAGCATCTACTT
G

>Tcp1 (FG850)_*Rhizopus microsporus*
GTTCGTCGTTGTAAGAAGGAAGACTTGAAGCGTATTGCCAAAGCCACAGGTGCTACTT
TGATCTCTTCTTTGGCCAACTTGGAAGGCGAAGAAACATTCGAAGCTAGCTATCTCGGT
CATGCCGAAGAGGTTGTTCAAGAACGTATCTCTGACGATGAATGTATTTTAGTAAAGGG
CACAAAGATACAAAATTCTGCATCTATCATCTTACGTGGTGCTAAGACTATATGCTTGAC
GAAATGGAAAGATCACTCCATGACGCCCTTTGTGTTGTTAAAAGAACTTTAGAGAGCA
ACAGCGTCGTGCCTGGTGGTGGTGCCGTTGAGTCAGCCTTAAGCGTATACCTC

>Tcp1 (FG850)_*Asperigillus triticus*

GTTCGCCGCTGCAAGAAGGAGGACTTGAGACGCATTGCCAAGGCTACCGGAGCCACC
TTGGTCAGCACGCTCTCCGATCTCAACGGAGACGAGAAGTTCGAGGCTTCGAACTTGG
GACATGCGGAGGAAGTTGTTCAGGAGCGGGTATCCGACGATGAGTGTATCCTCGTGAA
AGGCACCAAGGTCCACACTGCGGCGTCCATCGTCCTCCGAGGACCCAAGATTTCAGCT
TAGACGAGATGGAGCGTTCAGTCCACGATTCGCTGTGCGCCGTCAAGCGAACCTTGGA
AAGCGGTAGCATCGTCCCCGGTGGTGGCGCCGTCGAGACGGCCCTTCACATCTACCTC

>Tcp1 (FG850)_*Neurospora crassa*
GTGCGCCGCTGCAAGAAGGAGGATCTTCGTCGCATCGCCCGTGCCACCGGCGCCACCC
TCCTCAGCACTCTTTCCGATCTCAATGGCGACGAGAAGTTCGAGCCCTCTTACCTGGGT
CACGCCGAGGAGGTTGTGCAGGAGCGCATTTCCGACGACGAGTGCATCTTGATCAAG
GGTACCAAGGTTCACTCTTCGGCTTCCATCATTCTCCGTGGCCCCAAGACTTCACCCTC
GACGAGATGGAGCGCTCGGTTCACGACAGCTTGTGCGCCGTCAAGCGTACCTTGGAG
AGCGGCAGCATTGTGCCCGGTGGTGGCGCGGTCGAGACGGCTCTGCACATCTACCTG

>Tcp1 (FG850)_*Agaricostilbum hyphaenes*
GTACGCAGATGCAAGAAGGAAGATCTCAGACGTATTGCAAAAGCAACAGGGGCGTCG
TTGGTCAGCTCTTTGGCGAATCTGGAAGGCGAAGAGACATTCGAGAGCTCCATGTTGG
GTCAGGCAGAAGAGGTCGTGCAGGAGAAGTTTGGTGACGATGAGTGCATTTTGGTCA
AGGGTACAAAGGTCGTTTCAAGCTCCAGCATCATCTTGAGAGGCGCGAAGACTATATG
CTCGACGAGATGGAGCGATCGCTACACGACGCTCTGTCCATCATCAAGCGTACCCTCG
AAAGCGGCTACGTCGTCCCAGGCGGTGGTGCAATCGAGACAGCTCTATCCATCTACCT
G

>Frs2 (MS524)_*Tricholoma matsutake*
ACGCACCTGGCTGAATTCCATCAGGTGGAAGGAGTTGTTGCTGATCGGGGACTGACGT
TAGCCGACCTCATCGGTGTGTTCCCTTTAGGAGGCTTATTTATGCACCAGGCTTTATGAG
GGTTTTCTTCGAAAAGATGGGCATTGACAGGGTGAGGTTCAAACCTGCCTACAACCCT
TACACCGAGGTCTGTTTATTGTCATCCACTGACCATCGCATCATAGCCCTCTCTCGAAAT
TTTTGCTTTCCATCCAATGCTCAACCGCTGGGTGGAGGTTGGAAACAGTGG

>Frs2 (MS524)_*Gymnopus androsaceus*
ACTCATTTAGCCGAATTCCATCAAGTCGAAGGTGTCGTTGCAGATAAGGGATTGACTCT
CGCCGACCTGATTGGTGAGATGACTTGAAGTTACGATTTTCTTATTTACTTCTATCTAGG
TTTCATGAGAGTGTTCTTCAAAAAGATGGGCATTGAAAAAGTCCGCTTCAAGCCAGCA
TATAACCCTTACACGGAGGTTAGAAATATTTCGTATCGAATCTTCTAACGCAGTTCATAG
CCTTCACTTGAGATCTTTGCTTTCCATCCCATGCTGGACAAATGGGTTGAGGTCGGTAA
CAGCGG

>Frs2 (MS524)_*Laccaria bicolor*
ACTCATTTGGCAGAGTTTCATCAAGTGGAGGGTGTTGTAGCTGACCGTGACCTGACAT
TGGCCGACCTTATTGGTGAGTTCAACTCAAGTCACCCTGGTCGTATATTGTGAACCTCG
TAGGGTTCATGAGAGTATTCTTTAAAAAAATGGGCATTGAGAAAGTGAGGTTCAAACC
AGCCTATAACCCCTACACTGAGGTAGGTAAATGCTAAGATCGAACTAGTGAAGGAGAT

GTCTAGCCTTCTCTGGAAATCTTCGCATTCCACCCCATGCTGAATCGGTGGGTAGAAGT
GGGAAATAGTGG

>Frs2 (MS524)_*Asperigillus triticus*
ACCCACCTGGCAGAGTTCCACCAGATCGAGGGTGTCATCGCCGACTTCGGGCTCACGC
TGGGCGGCCTGATCGGCTTCATGGAGGTCTTCTTCGCCAAGATGGGCATCCACCAGCT
GCGCTTCAAGCCCGCCTACAACCCTTACACCGAGCCCAGCATGGAGATCTTCGGCTAC
CACGAGGGTCTGGGCAAGTGGGTGGAGATCGGTAACAGCGG

>Frs2 (MS524)_*Schizophyllum commune*
ACGCATCTCGCGGAGTTCCACCAGGTCGAGGGCGTCGTCGCGGACCGCGGACTCACG
CTCGCCGACCTCATCGGCTTCATGCGCGTCTTCTTCGCGAAGATGAGCATCGAGCAGG
TGCGCTTCAAGCCCGCATACAACCCGTACACGGAGCCTTCCCTCGAGATCTTCGCCTTC
CACCCCATGCTGCAAAAGTGGGTTGAGGTCGGCAATAGTGG

>Frs2 (MS524)_*Mycena crocata*
ACGCACCTAGCCGAGTTCCACCAGGTAGAGGGTGTTGTCGCGGACCGCGGACTCACT
CTGGCCGACTTGATTGGCTTCATGAAAGTCTTCTTCAGTAAGATGGGTATTAAGCAGGT
CAGATTCAAGCCCGCGTACAACCCATACACGGAGGTCTGTCCCTGCTTCACGAGTGGT
GCCTGATGTGCAATACAGCCTTCCCTGGAAATTTTTGCATTCCACCCCATGCTAGACCG
GTGGGTTGAGGTTGGGAACAGCGG

>Frs2 (MS524)_*Coprinopsis cinerea*
ACCCACCTCGCCGAGTTCCATCAGGTGGAGGGCGTTGTAGCGGATCGCGATCTGACTC
TTGCAGATTTGATTGGTTTCATGAGGGTGTTCTTTAGCAAGATGGGGATCGAACAGGTC
CGATTCAAGCCTGCCTATAACCCCTACACCGAGGTATGTTGTAGCAATGGCTCACGCAC
CGGTTGGTTGGTGTAGCCGTCACTAGAGATTTTCGCCTTCCACCCACTGCTTAACAAAT
GGGTCGAGGTGGGCAACAGCGG

>Frs2 (MS524)_*Hygrophoropsis aurantiaca*
ACGCATCTCGCGGAGTTCCACCAGGTAGAGGGCGTCGTCGCCGACCGGGGACTGACC
CTCGCTGATTTGATCGGTACACCCCCTCCTTCCTCGTCGCCCCGCACCCCCACCCACTT
ACAGGTTTCATGCGCGTCTGGTTCAACAAGATGGGGCTCACCAACCTCCGCTTCAAGC
CCGCGTACAACCCGTACACCGAGCCCTCGCTCGAGATCTTCGCGTTCCACCCGATGCT
CCGCAAGTGGGTCGAGATCGGGAACAGCGG

>Frs2 (MS524)_*Auricularia delicata*
ACCCATCTCGCCGAGTTCAACCAGGTCGAGGGCGTCATCGCGGACCGTGGCCTCACTC
TGGGTGACCTCATCGGTGCGCGCTTCCGTTCACGCGCATCCAACGGGGTGGGTGGGAT
ATAGGCTTCATGGAGGTGTTCTTCGGCAAGATGGGACTATCGCAGTTGCGCTTCAAGCC
GGCGTACAACCCGTACACGGAGCCGTCGCTCGAAATCTTCGCCTTCCACCCGCAGCTG
AAGCGGTGGGTGGAGATTGGAAACAGCGG

>Frs2 (MS524)_*Lactarius quietus*

ACGCATCTGGCGGAGTTTCACCACGTCGAGGGCGTCGTTGCGGACCGCGGGCTGACG
CTCGCCGATCTGATAGGTCCGTGAGGCGGACCCCACGCGTGTCACTGATGGCGTTGTT
TCCAGGTTTCATGCGAGTATTCTTCAGCAAAATGGGGATCAAAAACGTCCGCTTCAAG
CCTGCATACAACCCCTACACAGAGGTGACTCAGTTTTTGAAGATAATGATCAATGTCGA
CTTGTAGCCGTCCCTCGAGATCTTCGCGTTCCACCCGATGCTGAACAAGTGGGTCGAG
GTGGGGAACAGCGG

>Frs2 (MS524)_*Fomitopsis pinicola*
ACGCACCTGGCCGAATTCCACCAGGTCGAGGGTGTCGTTGCAGACCGTGGGCTGACA
CTTGCAGACCTCATTGGTGAGCGTCTGCTACCTCGTGTCACTGTGACATGCGGTCATGC
GCAGGCTTCATGAGGGTCTTCTTCAACAAGATGGGCATCAAGAATATCCGCTTCAAGC
CCGCTTACAATCCGTACACGGAGGTTCGTTGGGCTATTGCTACATGGAACTAAATCTCG
ATTCAGCCGTCACTAGAGATCTTCGCCTTCCATCCCCAGCTCCGTAAATGGGTCGAAGT
AGGGAACAGCGG

>Frs2 (MS524)_*Trametes versicolor*
ACCCATTTGGCGGAGTTCCACCAGGTCGAGGGCGTCGTCGCGGACAAGAACTTGACC
CTTGCAGACCTCATCGGTGCGCCCTCAGCAATCTTATGCACGACTGACAGATAGTTTTT
GTAGGCTTCATGCGCGTGTTCTTCACGAAGATGGGCATCACGAACATCCGCTTCAAGC
CGGCATACAACCCGTACACCGAGGTCGGTCGTCTCGGCGCACTCCATTCCCCCGTCCC
TGCACAGCCCTCGCTCGAGATCTTCGCCTTCCACCCGCAGCTGAGCAAGTGGGTCGAG
GTCGGGAACAGCGG

>Frs2 (MS524)_*Phanerochaete carnosa*
ACACACTTGGCCGAATTCCATCAAGTGGAGGGTGTCGTTGCCGACCGTAACTTGACAC
TGGCGGATCTTATTGGTATGTCAACTTGCTTTTCACGCTGCCCCCGTCAGGCTTCATGC
GCGTCTTCTTCAAGAAAATGGGGATCGAGAACATCCGTTTCAAGCCTGCTTACAACCC
GTACACGGAGGTACATCTCGAAATGTTTCCGCATTCTGAAGTATTACCGTAGCCGTCAC
TAGAGATCTTTGCCTTCCATCCGCTACTTAAAAAGTGGGTCGAAGTCGGCAACAGTGG

>Frs2 (MS524)_*Gymnopilus chrysopellus*
ACACATTTGGCCGAGTTCCATCAAGTCGAGGGTGTCGTTGCTGATCGAAACTTAACGC
TGGCCGATCTAATTGGTTCGTTTAATGTCAAGCCACTCAACATCTTCCAGGTTTCATGA
GGGTCTTCTTCAGCAAGATGGGCATCGAGCGTGTTCGTTTCAAGCCGGCATATAATCCC
TACACGGAAGTGAGTTACCAAAGCCCGTCTTTCTGTCTCACATCCGTATAGCCCTCCTT
AGAGATTTTCGCTTTCCACCCGTTGCTGGATCGATGGGTAGAGGTTGGTAATAGCGG

>Frs2 (MS524)_*Marasmius fiardii*
ACGCATCTTGCAGAGTTTCATCAGGTCGAGGGCGTTGTTGCTGATCGTGGGCTTACTTT
GGCGGACTTGATAGGTATGTGTCCCGTGCCTTCCGTCTTTCCTTCGTTCCCTAAAAACT
AGGCTTTATGCGCGTGTTCTTCAAAAAAATGGGCCTAGAACAAGTCCGCTTCAAACCA
GCTTACAATCCATACACCGAACCTTCGCTCGAAATTTTCGCTTTCCACCCTCTTTTACAG
AAATGGGTTGAAGTTGGGAATTCAGG

>Frs2 (MS524)_*Agrocybe pediades*
ACACATCTTGCCGAGTTCCATCAAGTAGAGGGCGTCGTCGCTGACCGCAACCTGACAT
TAGCTGATTTAATCGGTTCGTTTGCGGGAACCTTTTGTTTCACTCACATTTTATATAACC
AGGATTCATGAGAGTTTTCTTCCAAAAGATGGGTATCGAGAAAGTCCGCTTCAAACCT
GCTTACAATCCTTATACGGAGGTGATTCACCCTTTTTTTGAATGTGTTAAGACGGTATTT
TAGCCTTCTCTTGAAATCTTTGCCTTCCACCCTATGTTGGACAGATGGGTGGAAGTCGG
CAACAGCGG

>Frs2 (MS524)_*Serpula lacrymans* var. *lacrymans*
ACGCATTTGGCTGAATTCCATCAGGTGGAGGGAGTCGTTGCCGACCGGAACCTTACAC
TAGCAGATCTGATAGGTGCGTTTCGTGTTATCAAGCATATGTATGATCTTGCTTGTTGTT
AGGGTTCATGAGAGTCTTCTTCAAGAAAATGGGAATTGAAAATCTAAGGTTCAAACCT
GCGTTCAACCCCTACACAGAGGTATGGCCTTCTTTCTCTATCTCAACTTACATCACATAA
AAGCCATCCTTGGAAATCTTTGCTTTCCATCCCTACCTGAACAAGTGGGTTGAAATAGG
CAACAGTGG

>Frs2 (MS524)_*Agaricus bisporus* var. *burnettii*
ACCCATCTAGCCGAGTTCCATCAAGTTGAGGGCGTGGTCGCGGACCGTGGCCTTACCT
TAGCTGATCTCATTGGCAAGCGATCTTGCTTTCATCGACTAGTTCAACTTACGGGAACA
CAGGCTTTATGCGCGTCTTTTTCAAAAAAATGGGCATAGAAAATGTAAGGTTCAAACC
AGCATACAACCCGTACACTGAGGTATGTACGATATTATCGACCACATCTTGATTTGTTTT
CTAGCCTTCCTTAGAGATATTCGCCTTTCACCCTATGCTTAACAAATGGGTTGAAGTAGG
AAATAGTGG

>Frs2 (MS524)_*Amanita muscaria*
ACGCATCTAGCAGAATTTCATCAAGTAGAAGGAGTCGTCGCGGATCGTGGACTCACTC
TTGCTGATCTTATCGGTATAATCAGTCACATCTCAATTGGAATGCTCTAGGGTTTATGCG
TGTCTTCTTCCGTAAGATGGGAATAGAAAAAGTCAGATTCAAACCAGCATACAACCCCT
ATACCGAGGTACATGGACTTTGGTCAACCTACTTCTGACCGACGTTCTAGCCTTCATTA
GAGATCTTTGCGTTCCATCCGCTCCTGGATAAATGGGTCGAGGTCGGAAATAGTGG

>Frs2 (MS524)_*Pluteus cervinus*
ACGCACCTTGCTGAATTCCATCAAGTAGAGGGTGTTGTAGCTGACCGTGGGTTGACTC
TGGCAGACTTACTTGGTACGAGCTGTTCTAGGTTATTGACTACCTGCAAGGCTTCATGC
GTGTCTTCTTCAATAAGATGGGAATCGAAAAGGTCAGGTTCAAACCTGCATACAACCC
ATATACGGAGGTACGAGTCCCTCCCATCTCATACATTAACATTATAAATTAGCCTTCCATG
GAAATTTTTGCATTCCATCCGCTGCTCGACAGATGGGTGGAGGTTGGTAACAGTGG

>Frs2 (MS524)_*Boletus edulis*
ACACACTTGGCTGAATTTCATCAAGTGGAGGGCGTCGTTGCCGATAGAAATCTAACGC
TGGCTGACCTCATCGGTAAGTTGCAATATGTACGTTTTGTTTATCACTGAACAAGACCCT
AGGATTTATGCGAGTGTTCTTCAAAAAAATGGGCATGACTGATATCCGTTTCAAACCCG
CATACAACCCCTACACAGAGGTCCGTATCAATGAATGCTTTGACCATATTGACATGGTG
CAGCCGTCACTTGAGATCTTTGCTTTCCACCCCATGCTTCAAAAGTGGGTTGAGATCGG

GAACAGTGG

>Frs2 (MS524)_*Gautieria morchelliformis*
ACCCATCTAGCAGAATTTCATCAAGTTGAGGGTGTTGTGGCAGATCGAGGACTAACATT
GGGCGACCTTATCGGTAATGGATGTTGTATTACTGTGCGGATTTTGCTTGGTGTTTTACA
GGTTTCATGCAGACGTTCTTCAGCAAAATGGGCATCACGAACCTACGGTTCAAGCCCG
CATACAACCCCTATACCGAGGTAACTTCCAAATTCTCATCCTCTGACGCAATTGTTATCC
AGCCTTCGCTAGAGATATTCGCCTACCATCCGGCATTGAATAAATGGGTAGAAATTGGG
AACAGCGG

>Frs2 (MS524)_*Ramaria acris*
ACGCATCTTGCAGAATTCCACCAAGTCGAGGGTGTTGTGGCAGATCGAGGCTTGACTT
TAGGCGACCTTATCGGTAATAAGATGTGTGCGGTACTATGAACAAATGATGTAATCTTCT
AGGCTTCATGCAGACCTTTTTCAGCAAAATGGGTATCGAAAACCTGAGATTCAAGCCA
GCATATAACCCATATACAGAGGTACTTTTCTCTTCTTTTTCATGTGCTGAGAGATTGTGAT
AGCCGTCGCTTGAAATTTTTGCTTTTCATCCAGCATTGGACAAGTGGGTAGAAATTGGT
AATAGTGG

>Frs2 (MS524)_*Fomitiporia mediterranea*
ACTCATCTCGCTGAGTTCCATCAAGTAGAGGGCGTTGTTGCTGATCATAACATTACTTT
GGGAGATCTCATTGGTGTGTTACTTCTTTTCGTAATAACGACTTATTCCGACTTGTGCAA
GGTTTCATGGCTACGTTCTTCAATAAAATGGGTATCCAAGACCTGAAATTCAAGCCCGC
GTACAATCCTTACACAGAAGTTCGTACCCAGATACAGATCCTTAATAGTTTCATTTATCA
GCCGTCACTAGAGATTTTTGCTTTCCATCCCACATTGAACAAGTGGGTAGAGATTGGGA
ACTCCGG

>Frs2 (MS524)_*Dacryopinax primogenitus*
ACCCATCTGGCAGAGTTCCACCAAGTTGAGGGTGTGGTAGCGGATAGGGGGCTCACGC
TTGGCGACCTGATTGGTATGCCCTTTTATTTTTTCGAGACAAATTGATATGCCACTTAGG
TTTCATGCAAACTTTCTTCGCGAAGATGGGCTTGCCAAATCTACGCTTCAAGCCGGCGT
ATAATCCATACACAGAGGTCAGTTTGATAGACGTATCACGTGATCATATTCCATCTAGCC
GTCCCTGGAGATTTTCTGCTGGCACCCTGGATTAGGCAAGTGGGTCGAGATTGGCAAT
AGCGG

>Frs2 (MS524)_*Calocera cornea*
ACCCACTTGGCTGAGTTCCATCAAGTAGAGGGAGTTGTAGCGGACAGGGGGCTGACAT
TGGGAGATCTTATCGGTACGTGTAGCTTTGTCTGCTTGTGGATTAGTGATAAGGCATGCC
AGGTTTCATGCAGACTTTCTTCGCGAAGATGGGTCTGCCAAACCTGCGCTTCAAGCCG
GCGTACAACCCGTATACGGAGGTACGAGGGATCCATGTATCACGTGATGATCTGTCGAT
TTAGCCATCTCTCGAGATCTTCTGCTGGCATCCTGGACTGGGCAAGTGGGTCGAGATTG
GCAACAGCGG
>Frs2 (MS524)_*Agaricostilbum hyphaenes*
ACACATTTGGCCGAGTTCCATCAAGTCGAGGGTGTGGTTGCAGACTACAACATCACGC
TCGGCGACTTGATTGGCTTCATGGACATCTTCTTCAGAAAGATGGGCATCACTCAGCTT

CGCTTCAAGCCCGCCTACAACCCTTACACAGAGGTACGTCTGACGGCTACAGAAGACT
GACGAGACTTGCTACAGCCCTCATTAGAGATCTTCTCATACCACCCAGGTCTCAAGAA
GTGGGTCGAGATTGGCAACTCGGG

>Frs2 (MS524)_*Rhizopus microsporus*
ACTCACTTGGCTGAATTCCATCAGATCGAAGGTGTCATTGCTGATAAAAACTTGACCTT
GGGTGATTTGATTGGTTTTATGGATGTCTTTTTCAAAAAGATGGGTATGGACAAGATTC
GTTTCAAGCCCACTTACAACCCTTATACCGAACCTTCTATGGAAATCTTTTCTTATCACG
AAGGATTAAAGACTTGGGTCGAAATCGGTAACTCGGG

>Frs2 (MS524)_*Neurospora crassa*
ACACATTTGGCCGAGTTCCACCAGGTCGAGGGTGTCATTGCCGACTATGGCCTGACCC
TCGGTGGTCTGATGGAGTTCATGGAGATCTTCTTCGGCAAGATGGGTCTGCATGATTTG
CAGTTCAAGCCCGCTTACAACCCTTATACCGAGCCTTCAATGGAGATTTCGTGAGTGCC
GCCATGTCGCCGTCGTTGAAGAAACCGAAACGCTACAAGGGCCTCGGCAAGCTCGTC
GAGATTGGAAACAGCGG

>Gdi1 (FG576)_*Phanerochaete carnosa*
CGCAACGACTACTACGGTGGCGAAAGTGCCAGTCTGAACCTGACCCAGGTACGTATCT
TGCCGTAGTATGGCATCACGCCGATATCTGTGACAGCTGTATCGCAAGTTCCGGCCAGA
TGAGACTCCTCCCTCTGAATTGGGACGCGACCGCGACTACGCCGTTGATCTGGTCCCC
AAGTTCATCATCGCGTCTGGCGAGCTCACCAAGATCCTCGTCCACACTGATGTCACCC
GCTATCTCGAATTCAAGCAGATTGCCGGCTCCTATGTGTACCGCGATGGCAAGATCTCC
AAGGTC

>Gdi1 (FG576)_*Marasmius fiardii*
CGCAATGACTACTATGGTGGCGACAGTGCCAGCTTAAATTTGACCCAGGTATTCATATC
GGTTTTTCATTCCAGTGTATTTAGCGAATCTCCAGCTTTATCGCAAGTTCCGCCCTGACC
AAGCCCCTCCCAGCGAACTGGGTCGCGACCGCGACTACGCCGTCGATCTCGTTCCCAA
ATTTATCATTGCCACCGGAGAACTCACCAAAATCTTAGTTCGCACAGAAGTCACCCGGT
ACCTTGAGTTCAAACAGATCGCAGGTAGCTTTGTCTATCGCGATGGCAAGATTTCAAA
AGTG

>Gdi1 (FG576)_*Coprinopsis cinerea*
CGCAATGACTACTACGGTGGTGACAGCGCCAGCTTGAACCTCACTCAGGTACGCAGTT
TCAAAGTCCAACCGACGGAAATGCCTGGCCCTCCAGCTTTATCGCAAGTTCCGCCCTG
ATCAGGCTCCACCCACCGAGCTCGGTCGCGATCGTGACTATGCCATCGACCTCGTCCCC
AAGTTCATCATCGCTTCAGGAGAACTCACAAAGATCTTGGTCCACACCGAGGTCACTC
GCTACCTCGAGTTCAAGCAGATTGCGGGAAGCTTTGTGTATCGCGATGGCAAGATCTC
CAAGGTA

>Gdi1 (FG576)_*Schizophyllum commune*
CGCAACGATTACTACGGCGGCGACAGCGCCTCTCTGAACCTCACCCAGGTGCGTGAGC
TAAAAGTCAGCAAACGCTGACTGCCTGCAGCTCTACCGCAAGTTCCGCCCTGATCAGC

AGCCGCCCGCCGAGCTGGGACGCGACCGCGACTACAACGTCGACCTCATCCCCAAGT
TCATCATCGCATCTGGAGAGCTCACCCGCATCCTCGTGCACACCGATGTTACCCGCTAC
CTCGAGTTCAAGCAGATCGCGGGCAGCTTCGTCTACCGCGAGGGCAAGATCTCGAAG
GTG

>Gdi1 (FG576)_*Fomitopsis pinicola*
AGGAATGACTACTACGGAGGAGACAGCGCGAGTCTGAACCTGACCCAGGTAGGCAGT
CCGACGCGGTGCAACGTCGCCGACTCCTGACACACAGCTCTACCGCAAGTTCAGGCC
CGACAAGACCCCGCCGACCGAGCTGGGGCGCGACCGCGACTACGCCGTCGACCTCAT
CCCCAAGTTCATCATCGCGTCCGGCGAGCTCACGCGCATCCTCGTGCACACGGACGTG
ACGCGCTACCTCGAGTTCAAGCAGATCGCGGGCAGCTTCGTCTACCGCGATGGCAAGA
TCTCCAAGGTA

>Gdi1 (FG576)_*Auricularia delicata*
CGCAACGACTACTACGGCGGCGACAGCGCCAGTCTGAACCTGACCCAGGCAAGCAGT
GCCTCTGCTGGCTTGCTAGACCCCTTGCGTCTGGCAGCTGTACCGCAAATTCCGCCCCG
ACCAGGCCGTGCCGACCGACCTTGGGCGGGACAGGGACTACGCCATCGATCTCATCCC
CAAGTTCATCATCGCGTCCGGCGAGCTCACCAAGATCCTCGTCCACACCGACGTGACC
CGCTACCTCGAGTTCAAGCAGATCGCCGGCTCCTTCGTCTTCCGCGATGGCAAGATCTC
CAAGGTC

>Gdi1 (FG576)_*Trametes versicolor*
CGGAACGACTACTACGGCGGTGACAGCGCCAGTCTGAACCTCACGCAGGTAATGCGG
TGGCCGATATCGCGCCTGATAACTCATGTTCCTCCAGCTCTACCGCAAGTTCAGACCCG
ACCAGGCCGTTCCTGCTGAGCTGGGCCGCGACCGCGACTACGCCGTTGATCTCATCCC
CAAGTTCATCATCGCGTCGGGAGAGCTAACGCGCATCCTCGTCCACACTGACGTGACG
CGCTACCTCGAGTTCAAGCAGATCGCTGGTAGCTATGTGTACCGTGATGGCAAGATCTC
CAAGGTC

>Gdi1 (FG576)_*Mycena crocata*
AGAAACGACTACTATGGTGGTGACAGCGCGAGTCTCAACCTGACCCAGGTGCATTGCT
GCATTTCTCGCTCACCAAAAGCTCACCTCTGCTTAGCTGTATCGCAAATTCCGCCCTGA
TCAGGAAGTTCCCACCGAACTTGGCCGCGACCGCGACTACGCCGTCGATCTCGTCCCC
AAGTTCATCATTGCCTCGGGCGAGCTCACAAAGATCCTCGTCCACACGGACGTCACCC
GATACCTCGAATTCAAGCAGATCGCCGGCAGCTTCGTTTATCGCGATGGCAGGATCTCC
AAGGTC

>Gdi1 (FG576)_*Serpula lacrymans* var. *lacrymans*
AGGAATGATTACTATGGCGGCGACAGTGCCAGTCTAAACTTGACCCAAGTAAGCTCAT
AGGTATCACAATCATGATTTCTGATCATCGTGCAGTTGTATCGCAAGTTCAGACCAGAT
CAAGCACCTCCTGCAGAACTTGGCCGTGACCGCGATTATGCAGTTGATCTGATCCCCA
AGTTCATCATTGCCTCTGGTGAGCTCACGAAGATCCTGGTTCACACCGACGTCACGCG
GTACCTGGATTTCAAGCAGATCGCGGGCAGCTTCGTGTACCGCGATGGCAGAATATCCA
AGGTT

>Gdi1 (FG576)_*Tricholoma matsutake*
CGAAACGATTACTACGGCGGGGACAGCGCCAGTCTTAATCTGACACAGGTCTGTATTTA
TAGAAGCCAGCGCTACCACACTTCCCTAGCTAGTTGTACCGCAAATTTCGACCTGATCA
ACCACCTCCTTCTGAGCTTGGTCGAGACCGTGACTATGCTGTTGATCTGATCCCGAAAT
TCATCATTGCTTCTGGAGAACTCACAAAGATCCTTGTCCACACCGATGTCACTCGATAC
ATGGAATTCAAGCAGATCGCGGGTAGCTTTGTCTACCGTGATGGGAAGATATCCAAAGT
A

>Gdi1 (FG576)_*Laccaria bicolor*
CGTAATGACTACTACGGTGGCGACAGCGCCAGTCTTAATCTGACCCAGGTGGCCCATAC
TGCTGTGGGCATGACCGAAACTCACCCATCCGCAGCTCTATCGCAAGTTCCGACCAGA
CCAAGCCCCACCAACAGCGCTAGGCCGTGATCGTGATTACGCCGTCGATCTTGTTCCC
AAGTTTATTATTGCGTCCGGGGAACTCACTAAAATCCTTGTGCACACCGACGTCACCCG
TTATCTTGAATTCAAGCAGATCGCTGGGAGTTTCGTTTATCGAGATGGCAGGATTTCTA
AAGTC

>Gdi1 (FG576)_*Gymnopilus chrysopellus*
AGGAACGATTACTACGGAGGTGACAGTGCCAGTCTGAATCTTACCCAGGTAATGCTTG
CCCCGATCTACCACTTCTCTGACAGCTCTGCGACAGCTCTATCGCAAGTTCCGCCCAGA
CCAAGAACCTCCGACAGAGCTCGGTCGCGACCGTGACTACGCAGTCGACCTTGTTCCC
AAATTTATCATCGCATCTGGAGAACTCACCAAGATCTTGGTCCACACCGATGTGACTCG
TTACCTCGAGTTCAAGCAGATCGCTGGTAGTTTCGTCTACCGAGATGGCAGGATTTCCA
AAGTC

>Gdi1 (FG576)_*Stereum hirsutum*
AGGAACGACTACTACGGAGGTGATAGTGCAAGTCTGAACTTGACTCAGGTATGTCGTT
ATCCTATCCATGAAGTGTTCGCTACCCGCGTACAGCTCTACCAGAAGTTCCGTGGTCAA
GCTCCACCAGCCGAGCTGGGGCGCGACAGGGATTACGCCGTCGATCTTGTTCCCAAGT
TCATCATTGCGTCTGGGGAGCTCACCAAGATCCTCGTCCACACCGATGTTACTCGCTAC
CTCGAGTTCAAACAGATCGCCGGTAGCTTTGTGTATCGCGATGGCAAGATCTCTAAGGT
C

>Gdi1 (FG576)_*Gymnopus androsaceus*
AGGAACGATTACTATGGTGGCGAAAGTGCTAGTTTGAATTTGACTCAGGTACTTCGTAC
ACTGTTGATCAGGGCTAACCCTTGCTTAGCTCTATCGCCAGTTCCGTCCTGGACAAGAG
CCACCTTCTGAACTCGGCCGTGACCGAGACTACGCTGTTGACCTTGTCCCAAAATTCAT
TATCGCATCCGGAGAGCTCACGCAGATCCTTGTCCACACCGATGTCACTCGCTATCTTG
AGTTCAAGCAGATCGCCGGAAGTTTCGTGTATCGCGATGGGAGAATCTCCAAGGTT

>Gdi1 (FG576)_*Hygrophoropsis aurantiaca*
AGGAATGACTACTATGGTGGTGAAAGCGCCAGCTTAAACCTTACCCAGGTAAAGTATTA
CATTCTTGATCCCGTCCCCGTTCACCATATCGCAGTTGTATCGCAAATTCAGACCTGGTC

AAGAGCCTCCCACTGAACTTGGGCGTGACCGCGATTACGCTATTGATCTCGTGCCCAA
GTTTATCATTGCCTCGGGAGAACTCACCAAGATTCTTGTGCACACTAATGTCACTCGCT
ATCTCGAATTCAAGCAGATCGCAGGAAGTTTCGTTTATCGCGACGGCAGGATTTCCAA
GGTC

>Gdi1 (FG576)_*Agaricus bisporus var. bisporus*
AGAAATGATTATTATGGTGGTGATAGTGCCAGTTTGAACTTGACACAGGTAAAAAAAA
GATGATATCTCATCTTTAAACTGACATCTTTCTAAGTTGTATCGCAAATTCCGTCCCGAC
CAATCTCCTCCTAGTGAACTCGGACGCGATAGAGACTACGCCGTGGACCTCGTCCCTA
AATTCATCATCTCCTCTGGTGAACTTACAGAGATCCTCGTACACACCGACGTCACTCGA
TACCTCGAATTCAAACAAATCGCTGGAAGTTTCGTTTATCGTGATGGTAGAATCTCAAA
GGTT

>Gdi1 (FG576)_*Agrocybe pediades*
AGGAATGACTACTATGGTGGTGACAGTGCCAGTTTGAACCTCACACAGGTGCTATATCT
TGTTTACCCCTTCATCGAGCTCATCCTCACCCAAGCTCTACCGCAAGTTCCGTCCAGAC
AAAGAAGTACCGACGGACCTTGGACGCGATCGTGACTACGCCGTTGATCTTGTCCCCA
AATTCATTATCGCATCTGGGGAGCTCACGAAGATTCTCGTTCACACCGACGTTACGCGT
TACCTCGAATTCAAGCAGATTGCTGGCAGTTTCGTCTATCGTGATGGAAAGATTTCCAA
GGTC

>Gdi1 (FG576)_*Pluteus cervinus*
CGTAACGACTATTATGGTGCAGACAGTGCTAGTTTGAACCTAACCCAGGTATCTCTATC
GCCGTTCATCATTCGCGGTCTCCCCCCTTCAGCTCTACCGCAAATTCCGTTCAGGCCAA
GAGCCCCCGGCCGACCTGGGTCGTGATCGTGACTATGCTGTCGATCTCATCCCCAAGTT
TATCATCGCATCAGGAGAACTCACAAAGATTCTGGTTCATACCGACGTCACCCGTTATC
TTGAATTCAAACAAATTGCCGGCAGCTTCGTGTACAGGGATGGCAGGATCTCTAAGGT
C

>Gdi1 (FG576)_*Gautieria morchelliformis*
CGTAACGACTATTATGGGGCCGAAAGTGCCAGTTTAAATCTGACTCAGGCGCGCTTGCA
TATCGCAGCTACTACAGACACTGATATATTTGTAGCTCTACCGCAAGTTCCGGCCGTCTC
AAGAACCCCCACAAGAACTTGGGCGGGATAGGGATTATGCCATCGACCTCATTCCCAA
ATTCATCATTGCCTCTGGCGAGCTCACTAAGATGCTTGTCCATACCGACGTCACCCGTT
ACCTTGAGTTCAAGCAGATTGCTGGGAGCTTCGTATACCGTGACGGCAAGATCTCGAA
AGTT

>Gdi1 (FG576)_*Ramaria acris*
CGCAATGACTATTACGGCGCAGAGAGTGCGAGTCTTAACTTGACCCAGGTGTGCTATCT
CCTGTAGCAATTCGCCTTCATTGACACATCACTAGCTCTACCGCAAGTTCCGGCCAAAC
CAAGAACCTCCGCAAGAGCTAGGGCGAGACAGGGACTATGCCATCGATCTTATTCCCA
AGTTCATCATTGCATCTGGCGAACTCACCAAGATGCTAGTTCACACTGATGTCACTCGT
TATCTTGAATTCAAGCAAATCGCGGGCAGTTTCGTATATCGTGATGGCAAGATCTCCAA
AGTG

>Gdi1 (FG576)_*Lactarius quietus*
AGGAATGACTATTATGGCGGCGACAGTGCCAGTCTGAACCTTACTCAGGTCTGCAGCT
GACATTATATGCTGCGGTTTCTGATCTGTCAATAGCTTTATCGAAAATTCCGCGGTGACC
AGGCTCCTCCCTCTGAGCTAGGTCGTGACAGGGATTATGCCGTCGATCTCGTCCCGAA
GTTTATCATAACATCCGGAGAACTCACGAAGATCCTCGTGCATACGGATGTCACACGTT
ATCTCGAGTTCAAGCAGATCGCGGGCAGCTTCGTCTATCGTGATGGCAAGATCTCTAAG
GTT

>Gdi1 (FG576)_*Boletus edulis*
AGAAATGACTATTACGGCGGCGATGGTGCCAGCTTGAACTTAACGCAGGCAAGTTACG
CGTCAACATTATGCATGGCCGCTCACGGGCATCTAGCTCTATCGCAAGTTCAGGTCGGA
TCAGCCCCCACCCACAGCACTCGGCCGCGATCGCGACTACGCCGTTGATCTGATACCC
AAGTTCATTATCACCTCCGGGGAACTCACCAAGATCCTCGTGCACACTGACGTGACGC
GCTATCTAGAGTTCAAGCAGATCGCAGGAAGCTTTGTTTATCGGGATGGAAAAATTTCC
AAAGTT

>Gdi1 (FG576)_*Amanita muscaria*
CGCAATGATTACTACGGTGGAGACAGCGCCAGTCTCAATCTAACACAGGTGCTGCCTT
CACCTCATCGTGCTCTGAACACGGTCTTTACTGCAGCTCTACCGCAAGTTTCGTCCAAA
GCAAGATCCTCCCAGTGAACTTGGTCGTGACAGAGACTATGCCGTGGATCTCGTACCC
AAGTTCATCATTGCATCGGGCGAACTGACTAAGATGCTAGTCCATACCGACGTAACTCG
TTACCTCGAATTTAAGCAAATCGCAGGCAGTTTTGTCTTCCGCGACGGCAGGATCTCCA
AAGTA

>Gdi1 (FG576)_*Fomitiporia mediterranea*
AGAAATGACTATTATGGCGGAGAAAGCGCCAGTCTGAACCTTACGCAGGTTAGTCCGA
TAGCTGAATGTGTGTTTATTCATATGCCACGTTCAGCTATACAGGAAGTTTAGACCGGG
CCAAGAACCACCGGCTGCGTTGGGTCGCGATCGAGACTATGCCATAGATCTTATTCCGA
AGTTTATCATTGCATCTGGCGAGCTGACAAAAATACTCGTCCATACGGATGTGACTCGT
TACCTTGAGTTCAAGCAGATCGCAGGAAGCTTCGTCTACCGTGATGGGAGGATCTCAA
AGGTC

>Gdi1 (FG576)_*Calocera cornea*
CGCAACGACTACTACGGCGGCGAGTCCGCCTCGCTCAACCTCACCCAGCTCTACCGCA
AGTTCCGCCCGGACGCCACGCCCCCGACCGACCTCGGCCGGGACCGCGACTACGCGC
TGGACAACATCCCCAAGTTCATCTTCGCCTCGGGCGGGATGACGGACCTGCTCGTGCA
CACGGACGTGACGCGGTACCTCGAGTTCAAGCAGATCGCGGGCTCGTACGTCTACCGC
GACGGAAAGGTCAACAAGGTC

>Gdi1 (FG576)_*Dacryopinax primogenitus*
CGGAACGACTACTATGGCGGCGAGTCAGCATCCCTCAACCTGACCCAACTTTATCGCA
AGTTCCGGCCAGACGCCACCGCACCCACCGACATAGGACGTGACAGGGACTACGCGC

TGGACCAGATTCCCAAGTTCATCTTCGCCTCTGGTGGGATGACGGACCTGCTCGTGCA
CACGGATGTGACTCGGTACCTCGAGTTCAAGCAGATCTCGGGCTCGTACGTCTACCGC
GATGGCAAGGTCAACAAGGTG

>Gdi1 (FG576)_*Agaricostilbum hyphaenes*
AGAAATGATTACTATGGTGGCGAGTCGGCCAGTCTCAACTTGACCCAGGTGGGCAGTT
GCAGAACGTCTCGCAATGAAGCTGAGACTCCTTCAGCTGTACCAGAAGTTCAGACCG
GGTCAGGAGGTGCCGACCAACCTGGGTCGCGACCGGGACTGCGCCGTGGATCTCATT
CCCAAGTTCATGATGGCCAACGGCGAGCTCACCCGTATGCTCATTCACACCGATGTCAC
CCGCTACATGGAGTTCAAGCAGATCTCTGGATCCTATGTCTTCAGAGATGGTCGCGTTT
CCAAAGTC

>Gdi1 (FG576)_*Rhizopus microsporus*
CGTAATGACTATTATGGTGCTGAATCAGCATCATTGAACTTGACTCAAGTAAGATGATAC
ATATTTAAACATTTATAAGTTTTACAGTAAATTTAGACCCGGAGAGAGTGTACCAGACCA
TCTCGGTCGTGATCGTGATTGGAACATTGACTTGATTCCTAAATTTATGATGGCTAATGG
TGAAATCGTTCGTTTCTTGACTCATACAGATGTTACTCGCTACTTGGAATTCAAGCAAAT
CGCGGGTTCTTATGTTTATAGTGGCAAGAAGGTCAGCAAAGTT

>Gdi1 (FG576)_*Asperigillus triticus*
CGTAACGACCACTACGGAAGCGAAGCTGCGTCGGTGAACATGACCTCAGTCTGATCTT
AGATCTGAATCCGTGCTAATGCTTTGCCTCTTCAAGAAGTACGGCAATGTCGCTCCCGG
CGAAGAACCCTGGAAGAAGTATGGGCGGGTCAACGACTGGAACATCGATCTGGTCCC
CAAGTTGCTCATGGCCAACGGCGAGTTGACGAATATTCTGGTTTCCACCGATGTCACAC
GGTACCTGGAGTTCAAGCAGATCGGGGGCAGCTATGTCCAGCAAGGGAAGGGCCCCA
AGGCG

>Gdi1 (FG576)_*Neurospora crassa*
CGCAACGACCACTACGGCAGTGAGGCGGCGTCGGTTAACCTCTCTTCAAGAAATATGG
CAACTTTGCCGCCGGTACCGAGCCTTGGAAGGAATACGGTCGCCCCAACGACTGGAA
CATCGACCTTGTTCCCAAGTTCCTCATGTCCTCGGGCGAGCTCACCAACATTCTTGTCT
CCACCGATGTTACCCGTTACCTCGAGTTCAAGCAGGTTGCCGGCAGCTACGTACAGCA
GGGTGCTGGTTCCAAGGCC

>Get3 (FG591)_*Gymnopilus chrysopellus*
GCATGTCAAATCCATGGAGTACTCAGTAATCGTTTTCGACACTGCACCGACTGGACAC
ACGCTGCGCTTCCTGTCCTTCCCAACAGTCCTCGAGAAAGCGTTGGGCAAGCTCAGCA
CCCTTGGTTCACGATTTGGTCCTATGATCAGCCAGGTATGCATCACATCCGTTTGTTCAA
AACGATCCTCTTGCTGCTGTGCAGATGTCAGCCATGATGGGTGGCGAAGCAGGTTCCC
AAGAGGACATGTTCGCGAAACTTGAGAACATGCGTGCTGTCATCACTGAGGTC

>Get3 (FG591)_*Gymnopus androsaceus*
ACACGTCAAATCCATGACCTACTCCGTCATAGTCTTCGATACTGCTCCGACCGGTCATA
CTTTAAGGTTTTTGAGCTTCCCAACGGTTTTGGAGAAGGCGTTGGGCAAGCTGAGTAC

GCTGGGGAGTAGGTTTGGCCCAATGATTGGTCAAATGTCCAGCATGATGGGAGGCGAA
GCGGCCAACCAAGAAGATATGTTTGCGAAGCTGGAGTCGATGAGGGCCGTTATTACAG
AGGTT

>Get3 (FG591)_*Coprinopsis cinerea*
GCATGTCAAGTCGATGCAGTACTCGGTCATCGTCTTCGATACCGCGCCTACTGGACACA
CCCTGCGCTTCCTTTCCTTCCCTACTGTCCTCGAGAAGGCTTTGGGCAAGCTCAGCACC
CTAGGCTCGAGGTTCGGCCCCATGATCAGCCAAATGTCCAGCATGATGGGCGGCGAGG
CGGGCTCGCAGGAGGATATGTTTGCAAAGCTCGAGTCGATGAGGGCTGTCATTACTGA
GGTC

>Get3 (FG591)_*Hygrophoropsis aurantiaca*
ACACGTCAAGTCGATGGAATACTCTGTGATTGTCTTCGACACCGCACCGACGGGACAC
ACCCTTCGTTTCCTTTCATTCCCGACTGTTTTGGAAAAGGCTCTAGGCAAGCTCAGCAC
TCTTAGTGGGCAATTTGGACCTATGATTCGACAGATGTCCAGCATGATGGGCGGTCAGC
AAGATTCCCAGGAGGATATGTTTGCTAAGCTCGAATCCATGCGAGCTGTGATCAATGAA
GTC

>Get3 (FG591)_*Trametes versicolor*
GCACGTCAAGTCCATGGAGTACTCTGTCATTGTCTTCGACACTGCGCCGACAGGACAC
ACCCTCCGTTTCCTGTCATTCCCCACTGTACTGGAGAAGGCTTTGGGCAAGCTGAGCT
CGCTTAGCGGCCGCCTCGGGCCTATGATCAGCCAGATGACCAGCCTTATGGGCGGCCA
GGCAGATGCGCCAGAGGACATGTTCGCGAAGCTGGAGTCAATGCGTTCGATCATCACG
GAGGTC

>Get3 (FG591)_*Phanerochaete carnosa*
GCATGTCAAGTCCATGGAATACTCCGTCATCGTCTTCGACACCGCCCCAACGGGACATA
CTCTACGTTTCTTGTCGTTCCCCACTGTCCTTGAGAAGGCCCTAGGCAAGCTTAGCACG
CTTAGCGGCCGCATAGGTCCCATGCTCAACCAGATGACTTCGCTCATGGGTGGTCAAG
GCGATCAAACAGAGGATATGTTCGCGAAGCTCGAGTCCATGCGCGCAGTGATTACGGA
GGTG

>Get3 (FG591)_*Boletus edulis*
GCACGTCAAATCGATGGAATACTCTGTCATCGTCTTTGATACCGCACCGACCGGTCACA
CACTTCGATTCCTCTCCTTCCCGACCGTCCTCGAGAAAGCACTGGGAAAGCTGAGCAC
CCTTAGTGGTCAATTCGGCCCCATGATCCGACAAATGTCATCCATGATGGGCGGGCAAC
AAGACTCTCAAGAGGACATGTTTGCCAAACTAGAGTCGATGCGGGCCGTCATCAATGA
GGTG

>Get3 (FG591)_*Dacryopinax primogenitus*
GCACGTCAAGAGCATGACGTACTCTGTCATCGTCTTCGACACTGCGCCTACAGGGCAC
ACACTGCGTTTCCTCTCCTTCCCGAGCGTGCTAGAGAAGGCACTGGGAAAGCTCAGTG
ATCTGAGCGGGAGGTTCGGGCCCATGCTCCAGCAGATGAGCGGCATGTTTGGCATGGG
ACAGCAGGAGGATATGTTCGGTCGCTTGGAGGAGATGAGGGGGACTATCACGGAAGT

C

>Get3 (FG591)_*Calocera cornea*
GCACGTCAAGTCGATGACGTACTCTGTCATCGTCTTCGACACGGCGCCCACGGGGCAC
ACACTGCGCTTCCTCTCCTTCCCCAGCGTGCTCGAGAAGGCGCTCGGCAAGCTCAGCA
CGCTCAGCGGGCGGTTCGGGCCGATGCTGCAGCAGATGAGCGGGATGTTTGGCATGGG
CCAGCAGGAGGATATGTTTGCCCGGTTGGAGGAGATGAGGGGGACTATCACCGAGGTC

>Get3 (FG591)_*Fomitopsis pinicola*
GCATGTCAAGTCGATGGAGTACTCAGTCATTGTGTTCGACACCGCTCCGACAGGACAC
ACACTCCGCTTCCTCTCGTTCCCCACCGTTCTTGAGAAGGCACTAGGCAAGCTTAGTG
CACTCAGTGGGCGTATCGGCCCTATGATCAATCAAGTACATTGCACAAAACTGCATTCA
TGTCATGGTTTACTGATGTGTGGCGCTGCAGATGACCAGTCTCATGGGTGGCCAGGCG
GACGCACCAGAAGACATGTTTGCCAAACTCGAGAGCATGCGCGCGGTGATCACGGAA
GTG

>Get3 (FG591)_*Pluteus cervinus*
ACATGTCAAATCTATGGAGTACTCTGTGATCGTTTTCGATACCGCACCTACAGGACATAC
CTTGCGTTTCCTATCCTTCCCTAGTGTACTGGAGAAGGCTCTTGGAAAGCTCAGCAGCC
TCGGGTCACGGTTCGCACCCATGATCAGTCAGGTACTTCCAACACAATTTCTTAGATTT
CGGACTTTATTTACTCACTGAATATCAGATGTCTAGTATGATGGGAGGCGAAGCAACTT
CTCAAGAAGACTTATTCGCCAAACTCGAGTCTATGCGGGCTGTGATTACCGAAGTC

>Get3 (FG591)_*Schizophyllum commune*
GCATGTCAAGTCCATGGAATTCTCGGTAATTGTGTTCGATACTGCACCAACTGGCCACA
CACTGCGCTTCCTCTCCTTCCCAAGTGTGCTCGAGAACGCACTCGGAAAGCTCAGCTC
CCTCGGCTCCCGATTTGGGCCCATGATCAACCAGGTGCGTCGGTTTCCCATGCATTTGC
TTTTGTACCAATGTCTTTCAGTTCTCCAGCATGATGGGTGGCGAGGCTGCGTCTCCTGA
GGACATGTTCGCGAAGCTCGATGAGATGAGGGCAACGATCACGGAGGTC

>Get3 (FG591)_*Tricholoma matsutake*
ACACGTCAAGTCGATGGAATACTCCGTTATTGTGTTTGACACGGCACCAACAGGCCAC
ACCTTGCGCTTTCTCTCCTTCCCCACTGTCCTCGAGAAAGCGCTCGGAAAACTCAGTT
CGCTCGGCTCGCGTTTTGGACCCATGATTAGCCAGGTATGCTGGAATTAATAGTGTTATC
CTCCATATTGACCGCCCCCCGAGATGTCCAGTATGATGGGCGGCGAAGCCGGCTCGCA
GGAAGACATGTTTGCGAAGCTAGATGCGATGCGCGCGGTTATCACGGAGGTT

>Get3 (FG591)_*Agrocybe pediades*
GCACGTTAAGTCTATGGAGTATTCCGTCATTGTCTTTGATACAGCACCAACGGGACATA
CTCTAAGATTTTTGTCCTTCCCAACTGTACTAGAGAAAGCGCTGGGGAAGTTGAGTTC
GCTCGGCTCTCGTTTTGGCCCCATGATAAGCCAGGTATGCTATGCTGTCAACTTTCTGCA
TCTTTGCAAACAAAATCAACTAGATGTCCGCTATGATGGGTGGTGAAGCCGGAACGCA
AGAAGACATGTTCGCGAAATTGGAGAACATGCGGGCAGTTATCACTGAAGTC

>Get3 (FG591)_*Mycena crocata*
ACACGTTAAATCAATGGAATACTCTGTTATCGTATTCGACACGGCACCAACCGGACACA
CGCTGCGGTTCTTGTCCTTCCCAACTGTGCTGGAGAAAGCGCTCGGTAAACTTAGCAC
GCTGGGCTCTCGCTTCGGGCCCATGATAAGTCAAGTATGTGACCATATTTCCACCCCAA
ATCAGGTTAAGACACATTAGATAGATGTCCAGTATGATGGGTGGTGAAGCCGGTTCACA
GGAAGACATGTTTGCGAAGCTCGACTCAATGCGTGCGGTGATCACGGAGGTG

>Get3 (FG591)_*Marasmius fiardii*
ACACGTCAAATCTATGGAATACTCTGTCATTGTATTTGACACGGCCCCTACTGGACACA
CCCTCCGTTTCTTGTCGTTCCCGACAGTTTTAGAGAAGGCATTAGGTAAATTGAGCACA
CTAGGTTCGCGGTTTGGCCCAATGATTAGCCAGGTCAGATCAAGCTTTGTTGAACCCA
GACGGTTCTTACGGCTGAAGACAGATGTCTGCGATGATGGGAGGCGAGGCAGGCTCA
CAAGAAGATATGTTTGCCAAATTAGAGTCTATGCGTGCTGTCATCACCGAAGTC

>Get3 (FG591)_*Laccaria bicolor*
ACATGTAAAATCCATGGAATACTCCGTGATCGTGTTTGACACTGCGCCCACGGGACATA
CCCTTCGCTTTTTGTCTTTCCCCACAGTATTGGAAAAGGCTCTTGGAAAATTAAGCTCC
CTTGGATCGCGGTTCGGGCCGATGATAAGTCAGGTACAGTGTTACTGTACATACTTACC
GTTCTCATCGTGACTTGATCTTAGATGTCGAGTATGATGGGAGGTGAAGCCGGTTCCCA
GGAGGATATGTTCGCGAAACTTGAGTCCATGCGCGGGGTCATCACTGAAGTC

>Get3 (FG591)_*Amanita muscaria*
ACACGTCAAATCTATGGAATACTCTGTCATCGTCTTTGACACTGCACCCACAGGACATA
CCCTTCGATTTCTTTCTTTTCCCACTGTTTTAGAGAAAGCTCTCGGGAAACTGAGCTCT
CTTGGGTCTCGGTTTGGACCTATGATTACCCAGGTATGATTCTTATCATGTCATTCATGTA
GTGACATATAATTACTAAATTATGGAGATGTCCAGTATGATGGGAGGTGAACCAGGGTC
GCAAGAAGACATGTTCGCTAAATTAGAATCAATGCGGGCTGTGATCACGGAGGTC

>Get3 (FG591)_*Serpula lacrymans* var. *lacrymans*
ACACGTCAAGTCAATGGAGTACTCGGTGATAGTGTTCGACACTGCACCAACAGGCCAC
ACCTTGCGGTTCTTGTCATTCCCGACTGTTTTAGAGAAGGCTTTGGGCAAGCTTAGTGC
CCTCAGCGGACAATTTGGGCCAATGATTCGTCAGGTACGTAATAGGTCCTATTTATTGAT
GCGTCACCAGGTAATAAACTATCTTTCAGATGTCAAGTATGATGGGAGGTCAGCAAGAT
TCTCAGGAAGACATGTTTGCTAAGCTGGAATCAATGCGTGCCGTCATCACTGAGGTC

>Get3 (FG591)_*Agaricus bisporus* var. *burnettii*
ACATGTGAAGTCGATGGAGTACGATGTCATTGTATTCGATACCGCACCCACTGGTCATA
CACTTCGTTTCCTATCCTTCCCTACCGTATTGGAAAAGGCTCTGGGAAAACTCAGTAGC
CTGGGCTCTCGTTTCGGTCCGATGATCAATCAAGTGCGTCCCGGTTTTAAATTCTTTTCA
TTTATTCTTTCTCGTCCTTTAGATGTCTTCCATAATGGGTGGGGATGCTGGTAGTCAGGA
GGATATGTTTGCTAAGCTAGAGTCGATGAGGGCTATCATCACAGAAGTG
>Get3 (FG591)_*Gautieria morchelliformis*
GCATGTAAAATCTATGGAATACTCTGTGATCGTGTTCGATACCGCACCAACGGGACATA
CACTACGATTCCTTTCGTTCCCGTCCGTATTGGAAAAGGCACTAGGGAAGTTGAGCGC

GCTTGGTGGACGATTTGGGCCAATGATTAACCAGGTTGAGCAACCCAGTCCTACTAGT
CATAACACATTGCCAGATGTCCGCCATGATGGGTGCCCCACAAGGCTCTCAGGAAGAC
ATGTTTGCGAAGCTCGAAGGGATGCGAGAAGTGATCACTGAAGTT

>Get3 (FG591)_*Ramaria acris*
GCATGTAAAGTCAATGCAATACTCGGTCATCGTATTCGACACCGCACCTACGGGTCACA
CTCTGCGTTTTCTTTCTTTCCCATCCGTCTTGGAAAAGGCATTAGGCAAACTGAGCGCG
CTCGGTGGACGATTTGGGCCTATGATCAATCAGGTTTCTAGAAGCGTGTTGTGTTTTCG
ATCTCTTCATGTTGTTGCTTCTGGTTTAGATGTCGGCGATGATGGGCGCACCACAGGGC
TCGCAGGAGGACATGTTCGCGAAGTTAGAGGGCATGCGGGAGGTTATCACAGAGGTT

>Get3 (FG591)_*Lactarius quietus*
GCATGTCAAATCCATGGAATATTCTGCGATCGTTTTCGACACGGCACCGACAGGCCACA
CTTTACGCTTCCTTTCCTTTCCCAGTGTGCTTGAGAAGGCGTTGGGGAAGCTATCCACT
CTGAGCGGACGCATCGGACCCATGATCAATCAGGTGGGCATCGTTTTGTATCTGCCTCA
GCGTTGCTCCACTTCCGAATAGATGTCTACGCTGATGGGCGGCCAAACGGACGCTACC
GAAGACATGTTCTCGAAGCTCGAATCAATGCGTGCGGTGATCACCGAAGTG

>Get3 (FG591)_*Stereum hirsutum*
GCACGTCAAGTCAATGGAATACTCGGTCATCGTGTTCGATACCGCACCTACAGGCCAC
ACTCTTCGTTTCCTCTCCTTCCCAACCGTCCTCGAGAAGGCACTCGGCAAGCTCTCTGC
ACTGAGTGGTCGCATTGGCCCTATGATTAACCAAGTAGGCGAATTCTACCAAGGTCATT
CGTTGGAAGATATGGTCGTGGCGAAATTAGATGTCGAGTCTGATGGGAGCCCAAGCTG
ACTCTACCGAAGATATGTTCTCGAAACTGGAGTCCATGCGGGCGGTCATCACGGAAGT
G

>Get3 (FG591)_*Auricularia delicata*
ATACGTCAAGTCGATGGAATACTCCGTCATCGTGTTCGACACCGCACCGACCGGCCAC
ACACTGCGCTTCCTGTCCTTCCCGTCCATACTTGAGAAGGCGCTGGGCAAGCTCAGCA
CGCTAGGCGGGCGTTTCGGACCGATGATCCAACAGGTAGCAGTATACCCCCTGGTTCTA
TACTTTGGCTCTGTCCACTCTACAGATGTCAGCCATGCTGGGTGGCGGTCAGACGGCG
CAGGAGGACGTGTTTGCACGACTCGAGAACATGCGGGAGGTGATCACCGAAGTA

>Get3 (FG591)_*Fomitiporia mediterranea*
ACACGTCAAATCAATGGAATTCTCAGTAATCGTCTTCGACACCGCACCAACAGGCCAT
ACACTCCGTTTCCTCTCCTTCCCGTCTGTGCTCGAGAAGGCTCTTGGAAAACTTAGTTC
TTTGGGGAGTCGGTTTGGGCCTATGATTAACCAGGTTCGTATCTTTCTTTCTTTCTTTTC
CAAGGCGAACGTGATCTTTTCTTTTGTAGATGTCCTCGATGATGGGTGGTCAACCTGGT
ACACAAGAAGACATGTTCGCGAAGCTTGACTCTATGCGCGAGGTCATCTCCGAAGTG

>Get3 (FG591)_*Rhizopus microsporus*
ACAAGTAAAGACGATGTCTTACTCAGTTGTTGTCTTTGATACTGCGCCTACTGGACACA
CGCTTCGTTTCTTATCGTTCCCTACTGTATTAGAAAAGGCACTTGCAAAGATTAGTGGAT

TAAGTAGTCGCTTTGGTCCAATGGTTCAGCAAGTATCAGGTATGATGGGTATGAATGCC
AATCAAGAGGATATGTTTTCAAAATTAGAAGAAATGAGATCGATTATTAACGAAGTC

>Get3 (FG591)_*Asperigillus triticus*
ACAGGTCAAGTCGCTTTCGTATGAGGTCATTGTCTTCGACACCGCGCCGACCGGTCATA
CTCTGCGTTTCCTCCAGTTCCCGACTGTGCTCGAGAAGGCCCTCGCGAAGCTCTCGCA
GCTGTCCTCGCAATTCGGGCCCATGCTGAACTCGATCCTCGGCGCCCGGGGTGGTCTG
CCCGGTGGTCAGGACGAGCTCCTGCAGAAGATGGAGTCCCTGAGGGAGACTATCAGC
GAAGTC

>Get3 (FG591)_*Neurospora crassa*
GCAAGTCAAGTCGCTCTCGTACGAGACCATCATCTTCGACACGGCACCGACCGGCCAC
ACGCTGCGCTTCCTGCAGTTTCCCTCGGTCCTCGAGAAGGCTCTGGCCAAGGTCTCGC
AGCTGTCTTCGCAATACGGCCCCTTGCTCAACGGCTTCCTTGGCTCCAACGGCACGCT
GCCCAACGGCCAGAACGAGATGATGGAGAAGCTCGAGACATTGCGGGCCACCATTTC
AGAGGTG

>Get3 (FG591)_*Agaricostilbum hyphaenes*
ACATGTCAACTCGATGCAATACTCGACCATCGTCTTTGATACCGCACCTACAGGTCATA
CATTGCGATTCCTCCAATTCCCTTCGATATTGGAGAAGGCGCTGGCCAAGTTGTCGGGA
TTAAGCGGGCGCTTTGGGCCCATGATGGGTCAGATGTCCAACATGATACGTTCCAGGTC
AGGCGCATGAAATCTCTGTTTGCGAAATGGACTGAGATTTTGGCGTCATTGAGAAAAT
GGAGTCTATGCGAGGCGTCATTACAGAAGTC

>Gsh1 (MS320)_*Pluteus cervinus*
TTCCCGTAATTTCAAGGGAAATAACCGACTCCCCCCACAGCACTTTGACCGCCAACATT
CGTACCCGACGCGGCTCTAAGGTTGCAATAAATTTACCACTGTTTTTGATGAGAATAC
TCCCCGCCCCTTCGTCGATCCAACTATTCCTTGGGATCGCGCACTCTACACCGAGGATG
ACGGTCCGTCAATTATCAATTAATCTTCCAACTAAATCTGAAATCTACAGAAGCGAAAA
ATGGAGCTGCACTACCTGATCATATTTACCTCGATGC

>Gsh1 (MS320)_*Marasmius fiardii*
TTTCCGTTAGTGGTGCTAAAAACAGTTTCAAATGCAACAGAACCTTGACTGCAAATATT
AGACGTAGGCGTGGCTCGAAGGTAGCCATCAATCTTCCGATATATTTCGACGAGAAAA
CCCCCAGACCATTCGTTGATCCAACAATACCTTGGGATCGCACTATTTACCCAGAGGAT
TCAGGTGAGGTTTGCATACACTCCGCTGAAAGCTCGGGCTTAATCTCTCAGAGGCTAA
AAATGGGGCTGCTCTCCCCGATCACATATATTTGGATGC

>Gsh1 (MS320)_*Agaricus bisporus* var. *bisporus*
TTCCCGTACGCTAAGTATGACCGAATTAATCTCCAGATAGAACTTTGACTGCGAACATC
CGAACACGCCGTGGCTCCAAAGTTGCTATCAATCTACCAATTTTTTCGACGAGAAAA
CACCTCGTCCATTCATAGACCCTACAATCCCCTGGGATCGTGATATCTATCCCGAAGACT
CTGGTGCGTTTAGTGTGTTATTCGGTTATGATCCTCAGCTTGTCCTGCAGAGGCCAAGA
GTGGTGCGGCCCTTCCAGATCATGTATATATGGATGC

>Gsh1 (MS320)_*Fomitiporia mediterranea*
TTCCCGTCAGTCATTAGTAGCTTACACCTTTCCCCTCTAGCACCCTTACTGCAAACATCC
GGTCTCGGCGAGGCTCTAAAGTAGCAATAAATTTACCCCTGTTCTTCGATGAGAAGAC
ACCAAGGCCGTTCATAGACCCAACGATCCCTTGGGACCGTAATATTTATCCCGAGGATC
CTGGTGAGTTTAAGTTGTACCGAAGATTTTCGGCTTTCCGCTGCTTTCAGAGGCAAAG
GACGGCGCGGCTCTCCGTGATCATATTTATATGGACGC

>Gsh1 (MS320)_*Laccaria bicolor*
TTCCCGTACGTGTGCCTACTTTCCTAACCCTTGCTTTCAGCACCCTTACGGCCAATATCA
GGAGTCGAAGAGGTTCCAAGGTTGCAATCAATTTACCAATTTTTTTCGACGAGAAGAC
CCCTCGTCCATTTGTCGACCCAACGATTCCCTGGGACCGCAACATTTACAAAGAAGATT
CAGGTTTGTATTGCTTTATGTCATTATTTTACTCAAAACCCGCAGATGCGAAGAATGGTG
CAGCCCTCCCCGATCACATTTATCTTGACGC

>Gsh1 (MS320)_*Gautieria morchelliformis*
TTCCCGTATGTCCACCAGAGATAGATTTGATTTTCATCAGCACCTTGACTGGCAACATC
CGCGCGAGGCGAGGTTCAAAGGTCGCGATCAACTTACCCATATTTATTGATAATATGAC
ACCACGGCCATTTGTGGATCCCACCATCCCTTGGGACCGTGACGTGTATCCCGAAGATT
CAGGTTAGATGCACTCCTTACGCTCATGACATCTTATGCAATTCGTACAGAGGCTAGAA
ACGGTGCCGCGCTCCAAGATCACATCTACCTGGATGC

>Gsh1 (MS320)_*Ramaria acris*
TTTCCGTGTGTACACTCTTAATTGTTAATGTTAATTCAAGCACTCTGACCGCCAATATTC
GGATGAGACGGGGTTCCAAGGTTGCTATCAACTTGCCCATTTTTTTTGACAGCAAGAC
ACCACGTCCATTTGTTGATCCTACAATTCCATGGGATCGGGACATATACCCAGAGGATTC
AGGTTCGTCCGATTCGACGTATATGTGTAAGGTTGCCAACTTCACATAGAGGCGAAGAA
TGGCGCTGCACTCCAGGATCATATATATTTGGACGC

>Gsh1 (MS320)_*Coprinopsis cinerea*
TTTCCGTACGCCCTTTTTGCAAATTTGACGTCACTTTCAGGACGTTGACTGCCAACATT
CGGAGCAGAAGAGGCTCCAAGGTAGCCATCAACCTCCCGATCTTCTTTGACAAAAATA
CGCCTCGCCCATTCATTGATCCAACGATTCCGTGGGACAGGAACATCTATCCGGAAGAC
CACGGTGAGTAGAAGATATTATTAGCCGGACTTTGTACTCATTGGTCAGAGGCGAAGGA
TGGGGCAGCCTTGCCTGACCACATCTATATGGACGC

>Gsh1 (MS320)_*Gymnopus androsaceus*
TTCCCGTCAGTACAATTCATTCAGCTGAAAGCTTTCACAGCACTCTAACCGCCAATATT
CGTAGTAGACGAGGTTCCAAAGTAGCGATCAACTTGCCAATATTCCTCGATGAGAAGA
CACCTCGCCCTTTCATTGATCCAACGATTCCATGGGATCGTTCGATCTACCCAGAGGAT
CCAGGTTGGTTCTGCTGTATTCACTGTTATAGCACGTCTCATCTTGTATAGAGGCTAAGA
ACGGAGCAGCGTTGCCCGACCACATATACATGGACGC

>Gsh1 (MS320)_*Auricularia delicata*

TTTCCGTACGCCCGGAGACCCGGTGGCTGACAGCAAGCAGCACACTGACCGCGAGCA
TTCGCAAGAGGAGAGGATCCAAGGTTGCCATAAACCTCCCGATCTTCGTCGACGAGAA
GACCCCACGCCCGTTCATAGATCCTACCATTCCATGGGATAGAAATGTCCACCCGGAGG
ACCCGGGTGAGCGACCATCGCTTAGCCACAGGGTTGCTCGCTGCGCGCAGAGGCAAA
GGCGGGAGCCGCGCTCACGGATCACATCTATATGGATGC

>Gsh1 (MS320)_*Agrocybe pediades*
TTTCCGTAAGTTTTTTGATTCATTTGCTTGCCTTTATTAGTACGCTTACTGCTAATATTAG
ACGGAGAAGAGGATCGAAAGTTGCGATCAATCTCCCCATCTTCGTAGACGAAAAGACC
CCTCGTCCTTTCATAGACCCTACTATTCCATGGGATAGGAACATTTACCCCGAAGATTCA
GGTAAGAGAATCTATCATCCTTTTCCTCATAAAGCTTATGGTTTTTAGAGGCCAAATCTG
GAGCCGCTTTACCAGACCACATCTACTTAGATGC

>Gsh1 (MS320)_*Gymnopilus chrysopellus*
TTTCCGTCAGTAACGATTCTTGTATTCATGCTCCCGCCAGGACTCTCACTGCTAATATTA
GACGTCGTAGAGGGTCAAAGGTCGCTATCAATCTTCCTATTTTCTTAGACGAAAACACC
CCGCGACCCTTTATTGACCCCACAATACCTTGGGATCGTAACTTATACCCAGAGGATCC
AGGTATGTTCTGATGACTTCTTTGCTATTCTTCTGACCTAGAACCTCAGAGGCCAAGTT
GGGAGCAGCGTTGCCTGATCATATTTATATGGATGC

>Gsh1 (MS320)_*Schizophyllum commune*
TTCCCGTAAGTGCAGTTGGGCGCGGTCTCACGATGATCAGGACATTGACTGCCAACAT
CCGGAGGCGGCGAGGCTCTAAAGTAGCCATTAACTTGCCTATCTACTCCGATGAGAAC
ACGCCAAAGCCTTTTGTCGACCCGACGATCCCCTGGGACAGGCAACTATTCCCAGAAG
ACTCCAGTGAGTTCGCATCACTTTACATCCGCCATTCTCATCACTCGTGCCAGATGCCA
AGGATGGTGCAGCCCTGGTCGACCACATCTACCTTGACGC

>Gsh1 (MS320)_*Hygrophoropsis aurantiaca*
TTTCCGTCAGTCTCCGCCATTTGAATTCATGTGACTCTAGTACACTTACAGCCAATATAA
GGACTAGGAGAGGGGCAAAGGTTGCGATCAACCTTCCCCTGTTCATTGATGAGAGGAC
TCCGCGACCTTTTGTTGACCCCACGATACCATGGCAGCGCGCAATATACCCGGAAGATC
CCGGTGAGTCTACATCTCACTACGATAGGAAGATCTTGTAATGTCCATAGAGGCTAAGC
ATGGTGCTGCCCTAATTGATCACATTTACATGGATGC

>Gsh1 (MS320)_*Serpula lacrymans* var. *lacrymans*
TTTCCGTCAGTGATTTAACTCGCAATTGACTTGCTTGCAGCACATTGACAGCCAATATC
AGAAGCAGAAGAGGATCAAAAGTAGCCATTAATCTCCCTATTTTCATAGACGACAAAA
CACCTCGCCCATTCGTTGACCCCACAATTCCATGGCAGCGCTCGATATACCCTGGGGAT
TCTGGTGAGGAAATTGATCATCAATATATGCATTTCATGAGATAACAACAGAGGCTAAG
CGTGGGGCTGCACTCAATGATCACATTTACATGGATGC

>Gsh1 (MS320)_*Boletus edulis*
TTCCCGTCGGTCCCTCCGCTTGTTTTCTAACTTTGTACAGCACCCTTACTGCCAACATTA
GAAGCAGGAGAGGTTCCAAAGTCGCCATCAATCTTCCTATTTTTATTGACGAGAAGAC

GCCAAGACCGTTCGTCGATCCTACTATACCGTGGCAGCGTTCTTTGTACCCAGAAGATC
CTGGTAAGGCTTTTCTTGAAAGCGCTCACAATGTTGATGTCTCGCCACAGAGGCCAAG
AATGGCGCAGCTTTAATAGATCATATTTACATGGATGC

>Gsh1 (MS320)_*Fomitopsis pinicola*
TTCCCGTCGGTGTTCTTGCATTGAGCCCTTTGATCTGCAGTACACTAACGGCAAATATTC
GGCGGCGACGCGGTTCGAAAGTCGCCATTAATCTTCCGTTGTTCATCGACTCGCGCACT
CCACGGCCGTTCGTGGATCCATCAATACCATGGCAGCGGAACATATACTCGGAAGATCC
CGGTATGTATGACAGCTTTCCGTGTGTATGTTTAGTGAGAGGTGTCCAGAGGCGAAGC
GGGGGGCCGCGCTGCTTGATCACATCTACATGGACGC

>Gsh1 (MS320)_*Phanerochaete carnosa*
TTCCCGTTCGTTTTGCGATCTGACCCTTACTTCTTGTCAGAACCCTGACGGCAAATATC
CGGCGGAGAAGGGGTTCGAAAGTCGCCATTAACCTTCCTTTGTTCATTGACGAAAACA
CTCCACGGCCATTTGTCGACCCGACTATCCCATGGCAGCGGAACATCTACCCGGAGGA
CCCAGGTGCGCACTGCGGCGCTCAATTCGCATCACATGCTGACCGAATCTAGAGGCAA
AACAGGGTGCGGCAAAGATAGATCACATCTACATGGATGC

>Gsh1 (MS320)_*Trametes versicolor*
TTCCCGTCGGTGTCCCAGGCTCAACCATCGCGTCGCATAGGACACTTACCGCTAACATC
CGGCGGCGGCGCGGATCGAAAGTCGCTATCAACCTCCCGCTATACATAGACACCCACA
CCCCTCGCCCGTTCGTAGACCCCACAATCCCATGGCAGAGGAATATATATCCGGAGGAC
TCGGGTGTGTACTATCGAATTGACGGTCAGGACAACGATGCTTTCCTCCAGAGGCCAA
GAAGGGAGCAGCATTTCTCGATCACATTTACATGGACGC

>Gsh1 (MS320)_*Lactarius quietus*
TTTCCGTACGTAGTAGATTCAGACGTGTTGACGATTCCAGAACTCTCACTGCGAACATC
AGAAGGCGGCGCAGGTCAAAGGTGGCCATCAACCTTCCCATATTCATCGATGACCGCA
CCCCGCGCCCCTTCGTAGACCCCACCATCCCGTGGCATCGGAATGTCTTTCCAGAAGAT
GCAGGTGGGGCTTCTCGGAAATGCAACCGCAGACGATCTAATTACTCTCAGAGGCAAA
AAACGGCGCCGCACTCATCGATCACATCTACATGGACGC

>Gsh1 (MS320)_*Stereum hirsutum*
TTCCCGTCAGTTCACGTGTGGCCCACTTCACACCCCTTAGAACGTTAACGGCAAACAT
CCGGACGCGAAGAGGATCAAAGGTTGCTATAAACCTTCCGTTATTCATAGATGAGCGTA
CACCCCGACCATTCGTGGACCCCACTATTCCGTGGCAGCGTAATATATATCCCGAAGATC
CCGGTATGCCTTGTCGCCTCTTCGTTCATGCTATTGCTGATAGCGTTCAGAGGCAAGAA
ATGGCGCTGCTTTGAACGACCATATATACATGGATGC

>Gsh1 (MS320)_*Amanita muscaria*
TTCCCGTCAGTATACCTGTTGCTGATGAACCACAACACAGCACGCTTACTGCGAATATC
AGACGTCGTCGAGGTTCAAAAGTGGCAATTAATTTGCCCCTTTTCTTCGATAAAAACAC
TCCGCGACCGTTCATCGATCCTACTGTACCCTGGGATAGATCAATCTACCCAGAAGACT
CCGGTAAGGAGTGCCGAGACCGTATTGTGTAATCTGCTGACTTACCTCAGAGGCGAAG

AATGGTGCCGCTCTCCCGGACCACATCTATATGGATGC

>Gsh1 (MS320)_*Tricholoma matsutake*
TTTCCGTCAGTTTTCATAATTTACTCAATATATCATGCAGGACCCTAACGGCAAACATCA
GATCCAGGAGGGGCTCCAAAGTAGCGATCAACCTGCCTCTTTTCATCGACTCTGAAAC
ACCCCGACCATTCATTGACCCTTCAATCCCTTGGGACCGTTCACTGTACCCCGAGGATC
CACGTAACTGGTCTGCGTTTCCCTTTATATCTTGTGCTCATCGTGATTAGAGGCCAAGAA
TGGTGCAGCACTTCCGGACCATATATACCTCGATGC

>Gsh1 (MS320)_*Mycena crocata*
TTCCCGTCAGTGAACTGACTCACCGCTTATCAGCACTCTGACCGCAAATATCAGACAG
AGGAGAGGGTCCAAAGTTGCAATCAACCTCCCACTGTACTTTGATGCACAAACTCCTC
GTCCATTCATCGATCCCACCATTCCATGGGAACGCTCTATATACGCTGAGGATCCTGGTT
AGATACAGCATTTTTCTCCCTGCCAAGATCCTGCTCTCAACCAGAGGCGCAAGCGGGA
GCTGCGCTTCCTAATCACATCTATATGGACGC

>Gsh1 (MS320)_*Agaricostilbum hyphaenes*
TTTCCGTGAGTTGGATTACGAACCAACTTCAGGACGCTGGCTGCGCACATTCGAGAGC
GAAGAGGTTCCAAGGTCGCCATGAATGTGCCAATCTTGATAGATAAGAATACTCCGCGT
CCCTTCATTGACCCCTCCATCCCATGGGACCGCCAGCTGTTCCCTGGCGACTCCGAGGC
CAAGGATGGTGCCGCGCTGCCAGATCACATCTATATGGATGC
>Gsh1 (MS320)_*Calocera cornea*
TTCCCGTACGTGCGGTCGCTGACCTGGCCCGGGGTAACAGCACCCTCACAGCCAACAT
CCGCCGCCGCCGCGGACACAAGGTCATCATCAATCTGCCCATATTCGAGGACACCCAC
ACGCCCAAGCCGTTCATCGACCCGACTATTCCCTGGGACCGCGACTGGTTCCCTGGGG
ATAACGAGGCGAAGAACGGAGCCGCGCTCCCGGACCACATATATATGGACGC

>Gsh1 (MS320)_*Rhizopus microsporus*
TTCCCGTAAGTTGATTTTCTTTTTTTTTCTTTTCTGATAGTACGCTGACAGCCAACATTC
GTCGTCGAAGAGGATCCAAAGTAAAGATCAATATGCCAATCTTTCATGACAAAAATACA
CCCAGGCCATTTATTGACCCTACCATTCCATGGGACCGTGACTTGTTCGATCATGATAAG
GAAGCAAAGGAAGGTGCGGCATTACCAGACCATATCTATATGGATGC

>Gsh1 (MS320)_*Asperigillus triticus*
TTTCCGACTTTGGCGGCTAATATACGATCGCGCCGAGGTCGGAAGGTCGAGTTGAATGT
GCCTGTGTTCAAGGACAAGAACACCCCTCGGCCGTTCAAGGATCCCTCTGTCAACTAC
GACCTCCACAAGTGGCCCGAGGATGATGACGTGCGGAACGGTGCCGCCAAGGACGAT
CATGTTTACATGGATGC

>Gsh1 (MS320)_*Neurospora crassa*
TTCCCTACCCTTGCGGCCAACATCCGTTCGCGCCGCGGCCGCAAAGTCCAGGTCAATG
TTCCAGTGTTCAGAGACGAGAACACCCCTTGGCCATGGAAGGACCCAACCGTCAACT
ACGACCTACACAACTGGCCCGAGGACGATGATGTTCGTAATGGTGCCGCTCCCGACAA
CTTTATTCACATGGATGC

>Gus1 (FG525)_*Phanerochaete chrysosporium*
ATGGATTGGGGTAACGCCATTGTTCGCTCCAAGACCACTGATGGATCCGGCAAAATCA
CCGGTGTGACTATGGAATTACACCTCGAGGGTGACTTCCGCAAGACCAAGAAGAAGAT
CACCTGGCTCTCTGCTCCCGTGAGAACCTCGTGGAGTCGACTCTGCTCGACTACGACT
ACCTCATCACGAAGAAGAAGCTCGAGGAGGACGACGATGTCAAGGACTTTGTCACCC
CCGTCTCCGAGTTCCGCGAGGAGGCTGTTGCCGATGCAAACGTGAAGGAGCTTAAGA
AGGGCGACATCATCCAGTTCGAGCGCAAGGGATA

>Gus1 (FG525)_*Trametes cinnabarina*
ATGGACTGGGGCAACGCCATCGTTCGCTCGAAATCGGTGAACGAATCCGGCGTAGTCA
CCGGCATCGAGATGGAGCTCCACCTCGACGGCGACTTCCGCAAGACCAAGAAGAAGA
TCACCTGGCTCGCACAGCCCACGCAGAACACCACCTCGTCGAGGCGACGTTGCTCGA
CCACGACTACCTCATCACGAAGAAGAAGCTTGAGGAGAACGACGACGTCAAGGACTT
CGTGACGCCCGTCACCGAGTTCCGCGAGGAGGCGCTCGCGGACGCCAACGTGCGCGA
GCTCAAGAAGGGCGACATCATCCAGTTCGAGCGCAAGGGTTA

>Gus1 (FG525)_*Fomitopsis pinicola*
ATGGACTGGGGCAACGCCATCGTACGCTCCAAGGAGACGAATGCCGACGGTGTGATCA
CACACATCACGCTGGACCTCCACCTCGCGGGCGACTTCCGCAAGACCAAGAAGAAGA
TTACCTGGCTGACCGACCCGGCGCGGACCACCCGCTCGTCGAGACGACGCTGCTCGA
CTACGACTACCTGATCACGAAGAAGAAGCTCGAGGAAGAGGACGACGTGAAGAACTT
CGTCACGCCGCAGAGCGAGTTCCGCGACGACGCGCTCGCGGACGCGAACGTGCTCGC
GCTGAGCAAGGGCGACATCATCCAGTTCGAGCGCAAGGGCTA

>Gus1 (FG525)_*Mycena crocata*
ATGGACTGGGGCAACGCCATCGTCCGTTCGAAGACCACCAATGCCGCCGGCGAAATCA
CCGCAATCGAAATGGAGCTCCATCTCGAGGGCGACTTCCGCAAGACGAAGAAGAAGA
TCACCTGGCTATCACAGTCGACGCGTCTCACCCACTCAGCCCCGTCGTGCTTCTCGACT
ACGACTACCTCATCACGAAGAAGAAACTGGAGGAGAACGACGAGGTCGCGAACTTTG
TGACGCCAGTGACCGAGTTCCGACAGGACGCACTGGCGGACGCGAACGTCGGTGACC
TGACCAAGGGCGACATCATGCAGTTTGAGCGCAAGGGGTA

>Gus1 (FG525)_*Lactarius quietus*
ATGGACTGGGGCAACGCCATCGTGCGTTCTAAAACGGCCGATAGCTCTGGCACGATCA
CGGCACTCACCATGGAGCTCAATCTCGAAGGCGACTTCCGCAAAACGAAGAAGATCA
CCTGGCTAGCACAGCCATCGCCGCGCACCCGCTAATCGACGCGACACTGATCGACTAC
GACTACATCATCACCAAGAAGAAGCTGGAGGAAAACGACGACGTGAAGGACTTCGTC
ACACCCGTGACGGAGTTCCGCGAGGACGCGCTCGCAGACGCGAACGTGCGCGAGCTC
AAGCGCGGCGACATCATCCAGTTCGAGCGTCGTGGGTA

>Gus1 (FG525)_*Serpula lacrymans* var. *lacrymans*
ATGGACTGGGGTAATGCTATTGTCCGCTCGAAAACTACTGGACCTTCGGGAGAAATCA
CTTCTCTCACCATGGACCTCAACCTCGAAGGTGACTTCCGCAAAACGAAGAAGAAAAT

AACCTGGTTAGCCCAACCCAGGAATCCTACCCATTGATCGATGTCACGCTCCTCGACTA
TGATTATCTTATCACAAAGAAGAAGTTGGAGGAGAACGACAACGTAGCCGACTTTGTG
ACTCCTATGACTGAGTTCAAAGAGGAGGCATTGGCTGATGCCAACGTAAACGACCTGA
AAAAGGGGGATATCATTCAGTTCGAGAGGAAAGGCTA

>Gus1 (FG525)_*Pluteus cervinus*
ATGGACTGGGGCAATGCTATTGTCCGTTCCAAGACAGTCGACGCTTCAGGTCATATTAC
CGCCATTGAGATGGACCTCCATTTAGAGGGCGACTTCCGTAAAACCAAAAAGAAGATT
ACATGGCTAGCTCAGCCGACGACGAACACCCCCTGTTTACTGTCACACTCTTGGACTA
CGACTACTTGATCACCAAGAAAAAACTCGAGGAAGGGGATAACCTGACGGACTTTGT
CACTCCTGTCACCGAGTTCAGAGAGCAAGCGTTAGCGGATGCTAATGTTGCCGATCTG
AAGAAGAGTGACATCCTGCAGTTTGAGCGGAAGGGATA

>Gus1 (FG525)_*Hygrophoropsis aurantiaca*
ATGGACTGGGGCAACGCCATTGTGCGCTCCAAAACGACTGGTCCATCTGGAGAAATTA
CCTCGGTTACCATGGATCTACATCTCGAAGGTGATTTCCGAAAAACGTCGAAGAAGAT
CACCTGGCTCGCACAGCCAATGACACTCACACCCTGATCGACATCACGCTCGTCGACT
ACGACTACATGATCACCAAGAAAAAATTGGAGGAGAATGATGACGTCAAGGACTTCGT
GACCCCTACGAGCGAGTTCCGCGAAGAGGCCGTTGCGGATCCTAATGTGCGCAGTCTC
ACGAAGGGGAACATCATCCAGTTTGAGCGAAAAGGCTA

>Gus1 (FG525)_*Tricholoma matsutake*
ATGGACTGGGGCAATGCTATCGTTCGCTCCAAGACAGTTGATGCAGCAGGCCAGATAA
CTGCAATCCAGATGGACCTCAATCTCGATGGAGATTTCCGGAAGACGAAGAAGAAGAT
AACGTGGCTTGGTGCACCAACTCTGCGCATCCTCTTATACCTGTCACACTCCTCGACTA
CGATTATTTGATCACAAAGAAGAAGTTGGAAGAGAACGACGATGTTGCTGATTTCGTT
ACCTCTGTTACGGAGTTCAGAGAGGCGGCGTTTGCAGACGCAAACGTCAGTGATCTGA
ACAAGGGGGATATCCTGCAGTTCGAGAGGAAAGGATA

>Gus1 (FG525)_*Agrocybe pediades*
ATGGACTGGGGTAATGCCATTGTCAAGTCCAAGGAGGTCGATGCTTCTGGAGCTGTCA
CTTCAGTGACGATGGACCTCAACCTTGAGGGTGACTTCCGCAAGACCAAGAAAAAGA
TCACATGGCTTGCCCAACCCACCCCGAACATCCTCTACCATCTGCTACTCTCGTTGACT
TCGACTACCTCATCACAAAGAAGAAACTCGAGGAAAACGACAACGTCGCTGACTTCG
TTACACACATCACCGAGTTCAAGGAGTATGCATACGCTGATGCCAACATCCTCGATCTC
CAGAAAGGAGACATCATTCAGTTCGAAAGAAAGGGATA

>Gus1 (FG525)_*Laccaria bicolor*
ATGGACTGGGGGAATGCAATTGTTCGGTCCAAGGTGGTGGACACATCGGGACTCGTGA
CATCCATTGAAATGGAGCTCAACCTCGAAGGCGATTTCCGGAAAACGAGGAAAAAGA
TCACTTGGCTTGCCAGTCCTACCAGAACCATCCACTTCCAACGGTGTCGCTTGTCGACT
TCGATTACATTATCACTAAAAAGAAACTCGAAGAAAACGACGACGTCGCGGATTTTGT
GACACCAGTGAGCGAATTCAAGGAGGAGGCCTATGCGGACGCCAACACGCTCGAACT
GGCTAAGGGCGAGATCATGCAATTTGAAAGAAAGGGATA

>Gus1 (FG525)_*Gymnopilus chrysopellus*
ATGGACTGGGGCAATGCCATTGTACGCTCAAAGCAGGTTGATGCCTCGGGCATCGTCA
CGTCTATTACCATGGACCTGAATCTCGAAGGCGACTTCCGAAAGACAAAGAAGAAGAT
CACCTGGCTCGCTCAACCTACGCCGATCACCCTCTCCCGTCCGTAACCCTCGTTGATTT
CGACTACTTGATCACAAAGAAGAAACTCGAAGAAAACGATAACGTTGCGGACTTCGT
CACGCCCGTCACCGAGTTCAAAGAGTTTGCGTACGCTGATGCGAACGTCCTTGAACTC
AAGCCAGGCGACATCATCCAATTTGAGAGGAAGGGGTA

>Gus1 (FG525)_*Agaricus subrufescens*
ATGGATTGGGGCAATGCAATCGTCCGCCAGAAACAGATCGACGCCTCGGGCAAGGTCC
TGTCGCTTGCTATGGAACTCCACCTCGAAGGGGATTTCCGGAAGACGAAAAAGAAAAT
CACTTGGCTCGCAGAATCTACCCGGCGCACCCTCTCCCCACAGTGACGCTCACAGATT
TCGATTATATCATCACAAAGAAGAAGCTTGAGGAGGAAGATAACGTCGCAGACTTCGT
GACTCCGGTAACCGAATTCAAGGAGATCGCGTATGCAGATGCAAATGTCCTTGAGCTG
AAGAAGGGTGACATCATTCAATTTGAGAGGAAGGGATA

>Gus1 (FG525)_*Coprinopsis cinerea*
ATGGACTGGGGTAATGCTATCGTCCGAACTAAGTCTATCGACGCAGCCGGCACCGTCA
CAGAGATCGTCGTCGATCTCCACCTCGAAGGTGACGTGAAGAAGACCAAAAAGAAGA
TCACCTGGCTCGCTGCCCCCAAGACAACCACCCCCTCCCTACCGTCTCTCTCGTAGACT
TCGACTACCTCATCACGAAGAAGAAGTTGGAGGAGAACGACGAGTTTGCCGACTTCG
TCACCCCAGTTACCGAGTTTAAGGAAACCGCTCTCGCCGATGCTAACGTACTTGACCTC
CCCAAGGGCGAGATTATCCAGTTCGAGAGGAAGGGATA

>Gus1 (FG525)_*Boletus edulis*
ATGGACTGGGGCAACGCAATCGTGCGTTCAAAAGTGGTCGGAAATAACGGCGAAGTA
ACCTCCCTCGTTATGGAGCTCAACCTGGATGGTGACTTCCGAAAAACGAAAAAGAAAA
TAACTTGGCTTGCCCAACCCACGATTCCCACCCAGTAATCGAGGTCACCCTTCTGGATT
ATGACTACCTGATTACAAAAAAGAAGCTCGAGGAAGAAGATGACGTGAAGGACTTCG
TCCCACCAGTAAGCGAATTTCGCGAAGAGGCACTGGCGGATGCGAACGTGAAAACGC
CTAAAGTCGGCGATATCATCCAATTCGAGAGAAAAGGATA

>Gus1 (FG525)_*Gymnopus androsaceus*
ATGGATTGGGGTAACGCGATCGTTCGATCCAAGTCGGTTGATTCATCCGGTAATGTCAC
CTCTATCGAGATGGACCTTCACTTGCAGGGAGATTTCAAGAAAACTAAGAAGAAGATA
ACCTGGTTGGCTCAACCTTCTCCGAGCATCCTTTGATTCCTGTCACCCTCCTCGACTAC
GACTACCTCATCACCAAGAAGAAGCTTGAGGAGAACGACAGTATCAAGGATTTCATCA
CCCCCGTCACCGAATTCAAAGAGCTCGCGCTGGCAGATGCCAATGTCAAGGATCTCAA
AAAGGGCGATTATTTGCAGTTTGAGCGCAAAGGCTA

>Gus1 (FG525)_*Marasmius fiardii*
ATGGATTGGGGCAACGCTTTCATCCGTTCCAAAGTCATCGATGTCTCTGGTCAAGTAAC
CTCTATCGAAATGGAACTACATCTCGCAGGAGACTTTAAAAAGACCAAAAAGAAGATC

ACTTGGCTTGCGGAATCTACGCTGAACATCCTCTCATCCCTGTGACCCTCCTCGACTAC
GACTATTTGATCACCAAAAAGAAGTTGGAGGAGAACGATAACGTGAAGGATTTCATTA
CACCTGTCACGGAGTTCAGGGAGGCTGCGTTGGCGGATGCGAATGTGAGGGATTTGA
AGAAGGGGGATTATCTTCAGTTTGAGAGAAGGGGGTA

>Gus1 (FG525)_*Amanita muscaria*
ATGGATTGGGGCAATGCAATTGTCCGTTCCAAGAAAACTGATGCTTCCGGATCAGTTGT
ATCAGTAGAGATGGATCTTTACCTCGAGGGAGATTTTCGCAAAACCAAAAAGAAGATT
ACTTGGTTAGCTGCACCAAAGCCAATCATACCCTCGTCAGCGTCGTTCTATTGGATTAT
GATTACCTCATCACGAAGAAAAAATTGGAAGAGGAAGACGACGTTGCTGATTTCGTGA
CCCCTGTTACGGAGTTCAAAGACAGTGCCATGGCAGATGCCAACGTACGCGATCTCAA
AAAAGGAGACATCATACAATTTGAGAGGAAGGGTTA

>Gus1 (FG525)_*Stereum hirsutum*
ATGGACTGGGGCAACGCCATCGTGCGCTCCAAGACCACCGACGCCTCCGGTGCCGTTA
CATCCCTCACCATGGACCTCAACCTCGCCGGCGACTTCCGCAAAACCAAAAAGAAAAT
CACCTGGCTCTCCCAACCCACCCCGAGCACCAGCTCACCCCAGCCGTTCTCATCGATT
TCGACTACATCATCACGAAGAAGAAGCTTGAGGACGGAGACGAGGTCAAGGATTTCG
TCACGCCTGTCACTGAGTTCCGCGAGGAGGCGTGGGCGGATCCGAATGTGAGGTTGGT
GAAGAAGGGAGAGGTGATCCAATTCGAGAGGAAGGGGTA

>Gus1 (FG525)_*Dacryopinax primogenitus*
ATGGACTGGGGCAACGCGATCGTGCGCTCAAAAGAGACCGGACCGAACGGCGTCATC
ACGCATATCACGATGGATCTGCACTTAGAGGGCGATTTCAAGAAGACCGAGAAGAAGA
TCACGTGGCTCACCTCTCCGACGCGGCCCACCCGTTGGTCAACGTCGTACTGAAGGAC
TACGACTACCTTATTACGAAAAAGAAACTCGAGGAGGAGGACAAAGTGCAGGACTTT
GTTACCCCCGTCACGGAGTTCAAGGTCGCTGCCTGGTCGGACGCGAACGTGCTCGATC
TCGCTAAAGGGGATATCATCCAGTTCGAGCGGAAGGGGTA

>Gus1 (FG525)_*Calocera cornea*
ATGGACTGGGGCAACGCGTTCGTCCGCTCAAAGGAGAAGGACGCGAACGGCGTCATC
ACGCATATCGCGATGGAGTTGCACCTCGCAGGCGACTTCAAGAAGACGAAGAAGAAG
GTCACGTGGCTCGCCTGCCCCACCCCGCCCACCCGCTCGTCAACGTCGTGCTCAAGGA
CTACGATTATCTCATCACGAAGAAGAAGCTCGAGGAGGAGGACAAGGTGGAGGATTTT
GTCACGCCCGTGACGGAGTTCAAGGTCGCCGCATGGTCCGACGCGAACGTACTCGAC
CTCGCCCAGGGCGACATCATCCAGTTCGAGCGGAAGGGGTA

>Gus1 (FG525)_*Auricularia delicata*
ATGGACTGGGGCAACGCGTTCGTGCGCAAGAAGACGGGCGGCACGAACGGACTCGTG
GCGGCGCTGGAGATGGAGCTCTTCCTCGAGGGCGACTTCAAGAAGACGAAGAAGAAG
ATCACGTGGCTCGCCGACGGCGCGGCCGAGCGCGTGAGGCTGCTCGACTACGACTTCC
TCATCACGAAGCGCGCGCTCGAGGAGAACGACGAGCTCACGGACTTCGTCACGCCGC
AGACTGAGTTTGTCGTCGACGCCGTGACCGACGCAAATGTCCGCGCGCTCAAGAAGG
GCGACATCATCCAGTTCGAGCGCAAGGGGTA

>Gus1 (FG525)_*Gautieria morchelliformis*
ATGGATTGGGGCAACGCTATCCTCCGCTCAAAAACTCTCTCCCCCTCCGGTGCTGTCGA
GTCGCTGACAGCAGACTTGCACCTCGAAGGCGATTTCAGAAAGACTGAGAAAAAGAT
CACATGGCTTGCGGACTCTGCTCACCGAACCCGCTCGTCAGCGTGACACTTGTTGATTA
TGACTACCTGATCACAAAGAAAAAGCTAGAAGAGGACGATGATGTCGCAGACTTTGTT
ACTCCCGTTTCCGAGTTCCGCGTGGAAGCTTGGGCGGACGCGGGTGTGAAAGAATTAC
GAGTTGGCGACACCATGCAGTTCGAGCGTAAGGGCTA

>Gus1 (FG525)_*Ramaria acris*
ATGGACTGGGGGAACGCCATTATCCGTTCCAAAACACTCTCTCCCTCCGGCACTATCGA
GTCTCTCACCGCAGACCTTCACCTCGCCGGCGACTTCCGTAAAACAGAGAAGAAGATT
ACATGGCTTGCCGACTCGTCCCGTCACACTCCTTCGTGAGCGTTACGCTCCTCGATTAT
GACTACCTCATCACGAAGAAGAAGCTTGAGGAGGAGGATGATGTCGTGGACTTTGTGA
CGCCAGTCACGGAGTTCCGCGTTGAGGCGTGGGCGGACGCAGGCGTAAGGAGTCTGA
GCCCGGGGGATATTCTGCAGTTTGAACGTAAGGGTTA

>Gus1 (FG525)_*Asperigillus triticus*
ATGAGCTGGGGCAATGCCATTGTGCGCAAGATCACGGCGGACGCCTCGGGAGTGGTCC
AGGAGCTGGAGCTGGAGCTCCACCTGGAGGGCGACTTCAAGAAGACGGAGAAGAAG
GTGACCTGGCTGTCGCGGACCAGGAACTGGTGCCGGTGGAGCTGGTGGACTTTGACT
ACCTCCTCAAGAAGGACACCCTGCAGGAGGAGGACGCTCTCGAGGACGTCCTGAACA
AGAACACCGAGTTCCGCGAGGACGCCGTGGCCGACTGCAACGTGGCCGACCTGAAGC
AGGGCGAGATCATCCAGTTCGAGCGCAAGGGATA

>Gus1 (FG525)_*Neurospora crassa*
ATGGGTTGGGGCAACGGCTTTGTCCGCAACATTGACACCTCTGCCGAGGTCATCCCCG
CCTTTGAGATCGATCTCAACCTTGCCGGCGATGTCAAGTCTACCGAGAAGAAGGTTAC
CTGGCTCGCGTCAAGGGCCAGACGCTGGTCCCTGCCGAGCTCTGGGACTTTGACTACC
TCATCACCAAGGACGTTCTCCAGGAGGAGGACAACATGGAGGACTTCCTCAACCCTGT
CACCGAGACCATGGAGGAGGCCTGGTGCGACGAGGCGGCGGCCTCCCTCAAGAAGG
ACGACATCATTCAGCTCGAGAGACGCGGTTA

>Gus1 (FG525)_*Agaricostilbum hyphaenes*
ATGGACTGGGGCAATGCCTACATGCGCACCAAGCACACCGAGGGCGACGCCGTGACA
TCTCTCGACGCAGTGCTGCACCTCGAGGGTGACTTCAAAAAGACTGAAAAGAAAGTG
CATTGGCTGGCGACATGGATTCGCTCGTGCCTGTCACCTTGATCGACTACGACTACCTC
ATCACCAAGAAGCGTATCGAAGACACAGACGAGTGGACCGATTACATCAACAAGCAG
ACCGAATTCCGCACTGAGGCTTTTGCAGACCCCAACGTCGCTTTGCTCAAGCAGGGCG
ACATTATCCAGTTTGAGCGCAAGGGCTT

>Gus1 (FG525)_*Rhizopus microsporus*
ATGGATTGGGGAAATGTCTTTGTTCGCAAGGTAACCAAGAACGACCAAGGTCTGGCCA
CAGATGTTGAGTTGGAACTTCACCTTGAAGGTGATTTCAAAAAGACCAAGAAGAAGC

TCACTTGGTTGGCGCCGATGAAGACGCCACAGATGCTCTCTTGGTTGATTACGACTATT
TGATTACAAAGAAGAAGGTTGAAGAAGGCGATGATGTCAAGGATCTTGTTACACCTCA
AAGTGAATTTAAATACCCTGCCTTAGCAGATGGAAACATTAAACAATTAAAGAAGGGT
GATATTATCCAATTCGAACGTAAGGGATA

>Gus1 (FG525)_*Fomitiporia mediterranea*
ATGGACTGGGGCAACGCGATTGTGCGAAACATTTCGCGATCCGCGTCGGGGGATGTTA
CGCACCTCGATCTGGACCTACACCTCGCTGGAGACTTCAAAGCAACCGAGAAGAAGA
TCACCTGGCTCGCTGTGCAAGCCCATCACTGCCGCTTGTACCCGTCACCCTACTCGACT
ACGACTACATGGTAACGAAACGCAAACTCGAGAAAGATGATACTGTAGAGTCGGTTGC
AACGCCACAGACGGAGTTCAAAGTCGAGGCGCTCGCGGATGCGAATGTCCTTACGCT
GAAAGAACGTGATACCATTCAATTCGAGCGGAAAGGTTA

>Gus1 (FG525)_*Schizophyllum commune*
ATGGACTGGGGCAACGCGATCGTGCGCAAGAAGGCGCTCGAGGGCGACAAGGTCACC
TCCCTTGAGCTCGAGCTGCACCTGGAGGGCGACGTGAAGAAGACGAAGTTGAAGGTG
GGTGGTCGGGCTTGGCTGTGGCATCGCTCGCGCCCGTGCCGGTCACGCTCCTCGACTA
CGACTACCTGATCACGAAGAAGAAGCTGGAGGAGGGGGATGATGTGAAGGATTTTGT
CACCCCCCAGACCGAGTTCCGCACCTCAGCGATCGCGGACGGCAACGTGCGCACGCT
CAAGAAGGGCGACATCATCCAGTTCGAGCGCAAGGGGTA

>Hem15 (FG756)_*Amanita muscaria*
ACGACAGGAAGTAGCCTGAACGATCTATACAGACGAGGGAAATCTGGTCAATTTGGCT
CTGATGTCGAATGGAGCGTGATTGACCGATGGGGAACACATTCAGGCTTTATTGAGGTC
AGTTGCAATTTATTTATACCTGATGCGACCAAGAACAGGCAGTTACACAGAGAGTGGC
AGCAGCTCTCGCCAAGTTTCCTGCATCCACCAGAGCAGATACCGTAATTTTGTTCTCAG
CACACTCGTTGCCA

>Hem15 (FG756)_*Fomitopsis pinicola*
ACGACGGGGAGCAGCCTGAACGAGCTTTACAGGCGCGGCAAGGCGGGCGAGGTAGG
GGACGTGGAGTGGAGCGTCATTGACCGCTGGGGCACGCACCCGGGCTTCATCGAGGT
GCGTTCTGCTTCACTGTTTTTGACGTGACCGTCTGCTGCTGTACAGGCTGTCGCGCAGA
ACATCGAGCGGGCGCTGGCAAAATTCCCTGAGGAGACGCGCTCGGACGTTGTGCTGC
TCTTCTCGGCGCACTCACTTCCC

>Hem15 (FG756)_*Phanerochaete carnosa*
ACGACGGGCAGCAGTCTCAACGAGCTGTACAGAAAGGGCAAGACTGGAGAAATCGG
GGACGGCGTCGAATGGAGCGTGATTGATCGCTGGGGGACCCACCCAGGCTTTGTTGAG
GTGCGCGCTTTGGGATAGAGTTGCCGCCGAATGCGTGCCGTGGGACAGGCCATGGCAC
AGAACATCGAGCGTGCGCTCGCAAAGTTCTCCGAGGAAGAACGCTCGGACGTCGTCC
TTCTCTTCTCGGCGCACTCGCTACCC
>Hem15 (FG756)_*Trametes versicolor*
ACCACAGGGAGCAGTCTGAACGAGCTGTATCGGAAGGGCAAGACGAGCGACTTCGGG
GACGGGGTAGAATGGAGTGTGATTGACCGGTGGGGTACTCATCCCGGTTTTATCGAGG

TATGTCCGTCGATGTGTTCTCGAAGCTGCTTAACGTGCTGCATACAGGCGGTCGCGCAG
AACATCGAGCGTGCGCTGGCGAAGTTCCCTGCCGAGAAGCGCTCAGAGACGGTCCTG
CTCTTCTCCGCACATTCGCTCCCG

>Hem15 (FG756)_*Agaricus bisporus* var. *burnettii*
ACGACGGGAAGCAGCCTCAATGAACTGTTCAGAAGAGGAAAGGCTGGCGAAATGGGT
GACATAGAATGGAGCGTCATTGACCGTTGGGGAACTCATCCGGGATTTATTGAAGTGTG
CCACGTGAAATTCATTTTTCGACTAACCCTTGCATCTCTTCAGGCTGTGGCGCAGAATA
TCGAAGCCGCCCTCGCAAAGTTTCCCGAAGCAACGCGGTCGGAAACCGTGCTCTTATT
TTCGGCGCATTCTTTGCCC

>Hem15 (FG756)_*Marasmius fiardii*
ACTACAGGGAGCAGTTTGAATGAATTATACAGAAGAGGAAAAGCTGGAGAGTTTGGT
GACGTTGAATGGAGCGTGATTGATCGTTGGGGAACACATCCAGGTTTCATCGAGGTCC
GTCAGCTTTAACCGACTCGAGAAATTTACGTTCGCCGAATATAGGCTGTGGCTCAGAAT
ATCGAAGCGTCTCTTGCTAAATTTTCTCAATCCGAAAGATCAGAAGTCGTTCTCTTATTT
TCTGCCCATTCCTTGCCT

>Hem15 (FG756)_*Mycena crocata*
ACGACGGGAAGTAGTTTGAACGAGCTATTCAGGCGAGGAAAGGCAGGCGAACTGGAC
TCGGAAATTGAATGGAGTGTTATCGATCGTTGGGGAACACATCCTGGCTTTATAGAAGT
CCGTTTGTGTGCAATTATTGTTCACTCATTTTTACCCTTCCTTTAGGCTGTTGCGCAGAA
TATAGAAAAGACCCTCGCCAAATTCCCTGTTGCGACGCGCGATGAGACCGTCATTTTAT
TCTCAGCCCATTCCTTGCCC

>Hem15 (FG756)_*Gymnopus androsaceus*
ACGACTGGCAGTAGTTTGAATGAGATTTACCGACGGGGAAAGTCTGGTGAATTTGGAC
AAGATATTTCTTGGAGCGTTATTGACCGATGGGGAACGCATCCGGGGTTTATTGAGGTA
CGTGCAACCTCATTCATCTCGGGTCTAATTAATCTCTTCGTGCAGGCCGTTGCGCAAAA
CATAGAGGCTGCTCTGGCCAAGTTCCCTGCATCAACACGTTCAGAAACTGTCATCTTAT
TTTCGGCTCACTCCTTACCC

>Hem15 (FG756)_*Pluteus cervinus*
ACGACTGGAAGCAGTCTCAACGAGCTGTTCAAACGAGGGAAAGCCGGTGAACTAGGC
CAACTAGAGTGGAGCGTCATTGACAGATGGGGTACCCACCCTGGGTTTATCGAGGTGT
GTTTGGTGTTGTTCTTGCAAGGCGTCAAACGCAGCTCTTCTCAGGCTGTCGCGCAAAA
CATCGAAGCAGCACTTTCCAAATTCCCGGAGTCAACGCGTTCAGAAACAGTCTTGCTT
TTTTCAGCGCATTCGCTGCCC

>Hem15 (FG756)_*Tricholoma matsutake*
ACCACCGGTAGTAGTCTTAACGAGTTGTTCAGACGTGGGAAAGGGGGTGACATTGGTC
AGGATGTTGAGTGGAGTGTGATTGACCGCTGGGGCACACATCCCGGGTTCGTCGAGGT
GCGTCACATAACGCACACTTGATAATGGCTGCATGTTTTGTCCAGGCTGTCGCACAAAA
CATCGAAGCAGCTCTAGCGAAATTCTCTCCATCAACCCGGAAGGACACCGTACTGCTAT

TTTCGGCCCATTCATTACCT

>Hem15 (FG756)_*Hygrophoropsis aurantiaca*
ACTACTGGAAGTAGTTTGAATGAGCTCTTCAGAAAAGGAAGGGCGGGTGAAATCGGA
GACATTGAATGGAGCGTCATTGATCGGTGGGGTACCCACCCAGGATTCATTGAGGTCG
GCATTCATTTCAATACATCGAATGTTCAAAAATAATGTTACAGTCAGTTGCACAAAATAT
CGAAGCAGCGTTGTCGAAATTCCAAGAATCAGATCGCTCAGATGTGGTCTTGTTATTCT
CTGCACATTCCTTGCCC

>Hem15 (FG756)_*Boletus edulis*
ACGACAGGAAGCAGCTTGAACGAACTCTTCAGACGAGGAAGGGCGGGTGAGATGGG
AGATGTCGAATGGAGTGTGATTGACCGCTGGGGTACACATCCTGGCTTCGTCGAGGTG
AGTACCACTGTCTTTTGCCGGACACCCATGGCTATCCAGGCAGTGGCACAAAACATCG
AGGCCGCGTTACAACAATTCCAACCGTCCGATCGCTCTGACGTGGTGCTGCTATTCTCG
GCACATTCGCTACCC

>Hem15 (FG756)_*Gymnopilus chrysopellus*
ACGACTGGAAGCAGCCTCAATGAACTCTACCGACACCGAAACAACAGTGATATGCAA
GGCATTGAGTGGAGCGTTATAGACCGATGGGGTACGCATTCTGGCTTCGTGGAGGTAAT
TTTCATCTGATTTACTTTCTATTCAACGGCGTAATTATCTAGGCAGTCGCCCAGAACATT
GAGGCTGCTCTTGCTAAGTTTCCAGCTGATAAACGCTCGGAGACTGTCCTATTATTCTC
GGCTCACTCACTGCCC

>Hem15 (FG756)_*Agrocybe pediades*
ACAACAGGAAGTAGTCTGAATGAGCTCTACCGTAAACGAAAGGAAACGGGTTTGGAC
TCTATTGAATGGAGCGTCATAGACCGTTGGGGTACTCATCCGGGCTTCATTGAGGTGAG
TCGTAAAACATACAACTTTCCTTCATCTAAGGACCCTGTATAGGCTGTTGCCCAAAATAT
CGAAGCTGCACTCGCGAAGTTCCCGGCCGACAAGCGCTCGGAGACTGTCCTTTTATTT
TCAGCCCACTCTCTCCCC

>Hem15 (FG756)_*Ramaria acris*
ACGACTGGGAGCAGTTTGAATGAGGTGTGGAGGCGGGCTAAGGGCGGTGCGTGGGGA
GACGTGGAGTGGAGCGTGATTGATCGGTGGGGCACACATCCTGGGTTTGTCGATGTGC
GCCTCCCTCCAGTTTTGTCTTAGTGTGTTCTGATGCACCTCTAGGCCGTCGCACGAAAC
ATCGAAGCAGCACTCGCCAAATTCCCGCCAGACCGCCGCGCAAACGCCGTCCTCCTAT
TCTCTGCGCATTCCCTCCCC

>Hem15 (FG756)_*Gautieria morchelliformis*
ACCACCGGCAGCAGTTTAAATGAGATGTTTAAGCGAGGAAGCAAGGGGGAGCTGGGC
GGTATAGAATGGAGTGTCATTGATCGGTGGGGCACACATCCCGGCTTTGTTGAAGTGA
GCCTCTCGTCGCCCACAACTTAACCCGATCCTTTAATGATCAGGCAGTTGCACAAAATA
TCGAGAAAGCTCTCGAAAAGTTCCCTCCTGAACGCCGTTCCTCTGCCGTTCTTCTCTTC
TCGGCCCATTCTCTCCCC

>Hem15 (FG756)_*Coprinopsis cinerea*
ACCACGGGCAGCAGCCTTAACGAGCTATACCGACGCGGGAAGGCCGGTGAAATGGGT
GACATCAAATGGAGCGTAATAGACCGATGGGGAACACACCCTGGATTCATCGAGGTAG
GGTTTTTAAACTTTGCGAAGAAACAGCTCATCTCCTGGCTGTAGGCTGTCGCCCAAAA
CATTGAAGGAGCATTAGCCAAGTTCCCACCTGAAGTCCGGTCAGATGTTGTATTGCTAT
TCTCAGCGCACTCCCTACCG

>Hem15 (FG756)_*Serpula lacrymans* var. *lacrymans*
ACAACTGGAAGTAGCTTGAATGAACTTTTTCGTCAAGGCAAGGCCGGTGAAATGGGTG
ACATTGAATGGAGTGTGATCGACCGATGGGGGACACACCCTGGGTTTATCGAGGTACG
TTATTCACACTCCTTAGAACTACAGCTTTGTTTTTTCCCCTAGGCAGTATCCCAACACAT
AGAAGCAGCGCTAGCAAAATTCGATCCCGCGGTCCGCTCAGATGTGGTACTTCTCTTCT
CAGCGCATTCTTTGCCA

>Hem15 (FG756)_*Laccaria bicolor*
ACAACAGGAAGCAGTTTGAACGAGCTATATCGTCGTGGAAAGGGTGGAGAGATGGGT
GATGTCCAATGGAGCGTGATAGATCGCTGGGGAACTCATTCTGGTTTCGTCGAGGTACT
TGCCGCCTCACATACACATCTTACATCCACACCACAGGCAGTTGCGCAAAATGTTGAG
GCGGCTTTGGAGAAATTTTCACCCCACACACGCGCCAACGCCGTTTTACTCTTTTCCGC
ACATTCTTTACCA

>Hem15 (FG756)_*Schizophyllum commune*
ACGACGGGCAGCAGCCTGAACGAGCTCTACCGCAAGGGCCGGAGTAGAGAGTTTGGG
AATGAGGTCGAGTGGAGTGTGATCGACCGGTGGGGGACGCATCCCGGGTTCATTGAG
GTGCGTTGAGTGGACTTACCCTGTCGCGCAGGCCGTAGCGCAAAACGTAGAGTCGGC
GCTGGCCAAGTTCCACCCGTCGGACCGCAGCGACGCAGTGATCCTCTTCTCCGCGCAC
TCGTTGCCG

>Hem15 (FG756)_*Fomitiporia mediterranea*
ACGACGGGGAGCAGCCTTAATGAGATTTACCGGCTCGGGAGGTCAGGTGTGGCTGGCT
CTGGTGTGGAATGGAGCGTTATAGACCGATGGGGAACGCATCCTGGGTTTGTCGACGC
AGTTGCGCAACGCGTTGAAGCAGCACTTCAACGGTTCCCTCCTGACAGGCGGAAAGA
TGCAGTTCTTCTCTTCTCCGCACATTCTCTTCCC

>Hem15 (FG756)_*Stereum hirsutum*
ACGACGGGAAGTAGCTTGAACGAGATATTCAGGAAGGGAGCACCGAGTGGGATGGAA
GGGATCGAATGGAGTGTCATCGACCGGTGGGGAACGCATCCTGGGTTCATCGAGGTCC
GTGTCTTTTACTACCTCCGATTCCATTGTAACTTGTTGTCACAGGCCGTAGCTCAAAATA
TCGAAGCAGCACTACAGAAATTTCCCGAAGCCACTCGGTCGGACACCATTCTCCTCTT
CTCAGCACATTCCCTTCCC

>Hem15 (FG756)_*Calocera cornea*
ACTACTGGGAGCAGCTTGAACGAGATGTGGCGCTGGGCGACGGGGATCAGGATGGAA
GGGGTGGAGTGGAGCGCCATCGACCGGTGGTTCTCCCATCCGGGTCTGGTAGAGGTGC

GCGCCCTATGGCCTTCCCAACAGGAGCTAACACCACCCAGGCCTTCGCCCGCAACATC
GAAGCCGCGCTGGAGCACTACGAGCCGTCTGTGCGCTCGTCCGTCGTCCTCCTCTTCT
CCGCCCACTCCCTCCCC

>Hem15 (FG756)_*Dacryopinax primogenitus*
ACTACTGGGAGCAGCCTGAATGAGGTCTGGCGCTGGGCGACGGGGGTGAAGATGCCC
GGTGTGGAGTGGAGCGTTATCGATCGTTGGTTCGCCCACCCCGGTTTGGTAGAAGTAC
GTTTCCCCTCGCCTACACTAGCTGACGAAAGCAGGCATTCGCACGCAACATCGAAACT
GCGTTGGAACACTACGATCCCTCAACGCGTGACTCGGTCGTGCTCCTCTTCTCTGCCCA
CTCCCTCCCC

>Hem15 (FG756)_*Auricularia delicata*
ACGACCGGCAGCAGCCTGAACGAGGTCTTCCGCTCATGTGGCATCGATAGAGAGGCTG
GCGACGTCGAGTGGAGCGTCGTCGACCGCTGGGGCACCCATCCAGGTCTCGTAGAGG
TGCGTCTAGCGGTACATACTCCGCATTCGTGCTGACCGCCCACGCAGGCCTTTGCCCAA
ATTATCGAAACCTCGCTCGCAGAGTGGTCAGCGGAGGAACGCAAGGACGTAGTCCTCC
TCTTCAGCGCGCATAGCTTACCA

>Hem15 (FG756)_*Rhizopus microsporus*
ACCACAGGCAGCAGCCTGAATGAGCTTCATCGCCGTATTAAAGAATTGGGAATGGATA
CAGGTATCCAATGGAGCATCATTGACAGATGGCCAACACACCCTGGGTTTATTGATGCT
ACTGTTCACAAGATTGAACAAAAATTGGCAGAGTATGGACCAGAGGAACGAAAGGAC
GCGGTCATCATGTTCTCTGCTCACTCACTTCCA

>Hem15 (FG756)_*Agaricostilbum hyphaenes*
ACCACTGGCTCCAGTCTGAACGAGATGGCCAGGCAGATTAAGCAGCGACCGGGGCTC
AAGGATGTAGAGTGGACCGTGTTGGACAGATGGGCGACGCATCCTGGTTTCGTTGAGG
TACGCTGTTCTGCAGTTTCTAGACTTGCCGGCACAGCTGTACCCCAGGCCATGGCTCAA
GTCATCGAGCGCGGTCTGGAGTCTTATGACCCTGAAGTCCGCAAGGACGTCATCATTG
TGTATTCGGCACACAGTCTGCCC

>Hem15 (FG756)_*Asperigillus triticus*
ACGACGGGGAGCTCCCTCAATGAGCTGTGGAAATGGCGGACGCGGCTGGAGGCGGCG
GGCTCGATCCAGTGGAGTGTCATCGATCGGTGGCCGACCCATCCCGGCCTGGTGGAGG
CGTTTGCGCGGAACATCGAGGACCAGCTCAAGACGTACCCCGAGGACCGCCGGAGCG
GCGTGGTCCTGCTCTTCTCCGCCCACAGTCTCCCC

>Hem15 (FG756)_*Neurospora crassa*
ACCACGGGCAGCAGTTTGAACGAGCTATGGAAGTGGAGGCAAAGACTGGAGGAGGA
CGGTACCATTCGGTGGAGTGTGATTGACAGGTGGCCTACTCACCCTGGCCTTGTCGAG
GCCTTTGCGAGGAATATCGAGGAGAAGCTGGCCGAGTACCCCGAGGAGCGCAGAAAA
GATGTTGTGTTGCTGTTCTCGGCTCACAGCTTGCCC

>Hom3 (FG534)_*Fomitopsis pinicola*

ATTGAGGACAGCCTCGAAAAGAAGCTGCTCGACCGCGTCGTGCGTGAGCTGAAGAAG
AGCGGGACGGTCGGTGCCTGTCACCAATATTGTTGACGTCCGGTGCTGCAGGTGACTG
TCAGCAGAGACATGGCCATCTTGTCTCTGGTCGGCAAGAGGATGCGGAATATGGTCGG
CATAGCCGGCCGGATGTTCACGACCCTGGGCGACGGGAAGGTGAACATTGAGATGATC
AGCCAGGGTGCGAAC

>Hom3 (FG534)_*Schizophyllum commune*
ATTGAGGACTCGCTCGCGCCGAAGCTGCTCGACCGGCTCGTCACCGAGCTCAGGAAG
AGCGGGACGGTGTCGGCGCACCGCGATATGGCCATCCTGTCCCTCGTCGGGAAGCAGA
TGCGCAATATGGTCGGCATTGCGGGGCGGATGTTCACGACTTTGGCGCAGGGCAATGT
GAACATCGAGATGATCAGCCAGGGCGCGAGC

>Hom3 (FG534)_*Auricularia delicata*
ATTGAGGACCGCGGCGAACGCAAGAGCGTCGACCGCCTCGTGCGCGAGCTGCAGAAG
CTCGGCTCTGTCTCGGTGCATCGCGATATGGCGATCCTCTCGCTCGTCGGCAAGTCGAT
GCACAAGATGGTCGGCATCGCCGGCCGCATGTTCAGCACGCTCGCCGAAGGCGGCGT
CAACATAGAGATGATCAGCCAGGGCGCGAGC

>Hom3 (FG534)_*Mycena crocata*
ATTGAAGATGGGTTGGCGAAAAAGATGCTCGAGCGGCTTCTGCGGGAGCTGCAGAAG
AGCGGGACGGTATGCATCGACTGGTCTCATTGTTAAAACGCACCTACGCAGGTCTCGG
TGCACCACAACATGGCGATCCTGTCGCTGGTGGGAAACATATGTGCAATATGATCGGC
ATATCAGGGCGAATGTTCACGACACTTGCGCAGGGCAAGGTTAACATAGAGATGATCA
GCCAAGGCGCGAGC

>Hom3 (FG534)_*Gymnopilus chrysopellus*
ATTGGGGATAATCTGCCAAGGAAGATGCTGGACAGGCTGGTGAAGGAATTGAAGACC
AACGGCTCCGTATGTCATTCTCCCGTAGGCATTCTTCCTCTCTTCTCATAGGTATCGGTA
CATCATGACATGGCCATCCTCTCCCTCGTCGGGAAGCAGATGCGCAATATGGTCGGTAT
TGCAGGGCTCATGTTCACCACCCTCGCCCAAGGCGGCGTCAATATCGAGATGATCTCGC
AAGGAGCCAGC

>Hom3 (FG534)_*Trametes versicolor*
ATCGAGGAGAACTACGACAAAAAGGTGCTCGACCGCGTCGTGCGCGAGCTGAGGAAG
AGCGGCACCGTACGTCTTCACATCAGTACTCCTTTGCTGACCGTCCCGCAGGTCCAGG
TCATCCGGGATATGGCCATCCTCTCCCTGGTCGGGAAGTCGATGCGGAACATGATCGGT
ATTTCGGGCCGCATGTTCACCACGCTCGGCCAGGGCAACGTGAACATTGAGATGATCA
GCCAGGGCGCGAAC

>Hom3 (FG534)_*Amanita muscaria*
TTTGAAGACACGCTAGGCAAGAAACTTTTAGAAAGGCTTCTGAATGAACTACGGAAG
AGTGGGACGGTATGCGTTCTTTCTCTGTTCTCGGATACTTTTGTTCGTCAGGTGTCAGT
CCACCGCGACATGACCATCCTCTCGCTTGTCGGGAAACAGATGAGGAACATGGTCGGT
ATCGCTGGGCGAATGTTCACAACGCTCGCGCAAGGCGACATCAACATCGAGATGATCA

GCCAAGGCGCAAAT

>Hom3 (FG534)_*Laccaria bicolor*
ATCGAGGACGGGTTAGGGAAAAAAGTGCTTGATCAGTTGGTTGCGGAACTAAGGAAG
AGCGGCACGGTGAGTTTTAGCCTTGTTTCGAAATAGATAAGAATGAATCAGGTCTCCAT
CCACCGTGACATGGCTATCCTGTCCCTAGTAGGCAAACAGATGCCGAACATGGTGGGTA
TCGCAGGGAGGATGTTTCAGACCCTTGCACAAGGGAATGTCAACATCGAAATGATCAG
CCAGGGTGCAAGT

>Hom3 (FG534)_*Pluteus cervinus*
ATCGAGGACGGTCTTGCCAGGAAGTTCTTGGAGAGATTAGTCGGCGAGCTAAAAAAA
AGTGGAACAGTAAGGCGGATCGTGGCGATAGAAAGGCTGACGAGGTCCTAGGTCACG
CTCCACAAGGACATGGCCATCCTTTCCCTCGTTGGAAAACAAATGCGGAACATGGTCG
GTATTGCAGGACGCATGTTCACGACTCTTGCACAAGGAAATGTGAATATCGAGATGATT
AGCCAAGGGGCTAGT

>Hom3 (FG534)_*Stereum hirsutum*
ATTGAGGACGTGTTGGAGAAGAGGGTCGTGCAGAGGTTGGTTCGGGAGTTGGAGAAG
AGTGGGAGTGTGAGTCGTCTTCCCTTCCCTTTATTATTTGTTGTCGTACAGGTCTCCCTC
CACCGCGATATGGCCATCCTCTCCCTCGTCGGCAAACAGATGCGCCACTCGATCGGAAT
TGCGGGCAGGATGTTCACTACCCTCGCCCAGGGCAACATCAACATCGAGATGATCTCC
CAAGGTGCAAAC

>Hom3 (FG534)_*Coprinopsis cinerea*
ATTGCGGATGGGATGGGGAAGCGGGTGGTGGATAAGTTTGTGAATGAGCTGAAGAGGT
TCGGGACGGTATGTTGTTTTTACTGTTTTTGCATTACACCTATGGAACAGGTCTCGGTG
GGCAGGGATATGGCCATTCTCTCCCTCGTTGGCAAGCAGATGCGGAACATGGTCGGTAT
TGCCGGCAAGATGTTCACGACGCTTGCGGATGGCAATGTCAACATTGAGATGATCTCC
CAAGGCGCGAGT

>Hom3 (FG534)_*Phanerochaete chrysosporium*
ATCGAGCAGGGCGTCGAGCGCCGCTCGCTTGACAAGGTCGTGCGCGAGCTGAACAAG
TACGGCACTGTGAGTGGACATGGTTGTGATGCGAACGTTCGCGATCGCCAGGTGACGG
TCGCGCGCGACATGGCGATCCTGTCGCTCGTCGGGAAGCAGATGCGGCACCTCGTCGG
TATTGCAGGGCGCATGTTCACGACCCTCGGGCAGGGCAACGTGAACATCGAGATGATC
AGCCAGGGCGCGAAC

>Hom3 (FG534)_*Serpula lacrymans* var. *lacrymans*
ATCGAAGATAACCTGTCAAAAAAAATCATGGACCGTCTAGTCGCTGAGCTGAAGAAAA
GTGGAACGGTACGCGTGGTTTTAACGTCTTGCTTACCTTGACCTTTACAGGTCTCCGTC
CATCGCGAAATGGCTATTTTGTCACTTGTGGGAAAGCAAATGAGGAACATGGTTGGTAT
TGCCGGACGCATGTTCACGACACTCGCGCAGGGCAATGTGAATCTTGAGATGATCAGT
CAGGGGGCCAGC

>Hom3 (FG534)_*Hygrophoropsis aurantiaca*
ATTGAAGATAGTTTGTCCAACAAAATAATGGAGCGACTTGTCTCTGAGCTGAAAAAAA
GCGGAACGGTGGGTCATTATTTGGACATGCAGTCCTTACCTGCTCTCTAGGTGTCTGTC
CATCGGGAAATGGCCATTCTCTCGCTCGTCGGGAAACAGATGCGCAATATGGTTGGTAT
ATCCGGCCGCATGTTCACTACCCTGGCACAGGGAAATGTGAACATCGAAATGATTAGC
CAAGGGGCTAGC

>Hom3 (FG534)_*Gymnopus androsaceus*
ATTGAAGACGCGCTGGCGCAAAAGTTATTGGATAGGTTGATTTCGGAGCTGAAGAAGA
GTGGTACGGTGAGTTGTTTTCCTTCTTTTTCCTCTCACACATACTTCCAGGTCTCTGTCC
ACAAAAACATGGCCATTCTCTCCCTCGTCGGTCGAGAAATGCGTCACCTCGTTGGAAT
CGCCGGACGAATGTTCACCACGCTTGGAAACGGTAACGTCAATATCGAGATGATCAGC
CAAGGAGCGAGC

>Hom3 (FG534)_*Tricholoma matsutake*
ATCGAAGAGGGGTTATCTAAGAAAATTGAGGACCGGCTTATTAAAGATCTCAAGAAAA
CGGGAACGGTAAACGCAACTTTTGCATCTACAGTTTCTTCCCCTTCTTAGGTATCTGTA
CACCGTGACATGGCCATTTTGTCTTTGGTTGGAAAAGAGATGCGCAATATGGTCGGCAT
AGCTGGGAGAATGTTCACGGCGCTCGCACAGGGAAACGTCAACATTGAGATGATTAGT
CAGGGCGCGAGC

>Hom3 (FG534)_*Agrocybe pediades*
ATTGGCGATAGTCTTCCTAGAAAGGTTCTAGATAGACTGGTGAAAGATTTGAAAAACA
ACGGATCGGTACTATCATCTCCTTTTCCCCTGTTCACATTTATTCATCAGGTATCCGTGCA
CCATGACATGACCATCCTATCTCTCGTCGGAAAGCAAATGCGCAACTCGGTCGGTATTG
CGGGCTTGATGTTCACCACGCTCGCTCAGGGCAACGTGAACATCGAGATGATCTCGCA
AGGTGCTAGC

>Hom3 (FG534)_*Boletus edulis*
ATCGAGGACAATCTTCCCCCGAAGATCATGGACCGTTTAGTCTCCGAACTCCAAAAAA
GTGGATCGGTGAGCCTGCTCTGCCTCCTCGTGTACGCTATTTCCCAACAGGTCTCTGTC
CACACAGACATGGCTATTCTCTCGCTCGTTGGAAAACAGATGCGCAATTTGGTGGGCAT
GTCGGGACGGATGTTTCAATCACTCGCACAGGGCAACGTCAACATCGAGATGATCAGT
CAAGGCGCGAGC

>Hom3 (FG534)_*Agaricus bisporus* var. *burnettii*
ATAGAAGATGGATTGGCCAAGAAAGTGTTGGATAGACTGGTCAAGGAGCTCAGGAAA
AACGGAAGTGTGCGTATTTGTTCTATTTGACTTTGCTGAAAACGGAAACAGGTGTCGG
TGCACAAGGAGATGGCGATACTGTCGCTGGTGGGGAAGCACATGCGGAACATGGTTG
GGATTGCGGGAAGGATGTTTACGACACTTGCGGAGGGGAGTGTGAATATCGAGATGAT
CAGCCAGGGTGCGAGT
>Hom3 (FG534)_*Marasmius fiardii*
ATTGAAGGTAGTCTAGCCCCCAAATTACTCGATCGGCTTGTGCGGGAGTTGAAGAAGA
ATGGGATGGTACGACGGTTTCTCCCTTTCAGAAGTTTACTTTTCGCTCAGGTCACGGTA

CATCACAACATGGCAATTCTGTCCCTGGTCGGCAAAAATATGCGTCATCTCGTCGGCGT
AGCTGGGCGCATGTTCACGACTCTCGGTCAAGGGAACGTTAATATCGAGATGATTAGCC
AGGGTGCTAGT

>Hom3 (FG534)_*Lactarius quietus*
ATCGAGGACGTGCTCGATCGAAAGATGGAGGCCCGTCTCGTGAGGGATCTCGAGAAG
ATCGGTATCGTGCGTCTACCTCCCCCCTTCGCTTTTTCTCGTCCTTTTTAGGTGTCTGTG
TATCGCGACATGGCGATATTGTCCATGGTGGGCAAGCGCATGCGCAACACGGTCGGCAT
CTCCGGGCGCATGTTCACGACGCTCGCGCAGGGTAACGTGAACATAGAGATGATCAGC
CAGGGCGCGAAC

>Hom3 (FG534)_*Gautieria morchelliformis*
CTGGAGGAGGGGATGAGCAAGAAGGTGTTGAAGAAGCTGGTGTCGGAACTGGAGAA
AACGGGCAACGTGCGTGTTCCCCCTGCCGCGTACGCTCACGCGTGGGGGCAGGTCTC
GCTGCACGGCGACATGGCGATTCTGTCCCTGGTGGGACACCAGATGCGCCACATGGTA
GGGATTGCCGGGAGAATGTTCGCGACACTGGCAGAGGGGAACATCAACATCGAAATG
ATCAGCCAGGGGGCGTCC

>Hom3 (FG534)_*Ramaria acris*
ATAGAGGAGGGATTGAGTAAGAAGACACTGACCAGGCTTGTAAGAGAGCTGGAGAAA
GCCGGCTCTGTGAGTCTTTTTCTTATACACGTTTCTTATAGTGACATTCAGGTATCCCTT
CATCCTGACATGGCAATACTATCGCTCGTCGGTCACCAAATGCGGAATATGGTGGGAAT
TGCAGGGAGGATGTTCGCTACTCTTGCGGAGGGCAATGTCAATATCGAGATGATCAGTC
AGGGAGCATCG

>Hom3 (FG534)_*Fomitiporia mediterranea*
ATCGAGAATAACTTGGGCAAACGTATGATGGATCGACTGGTAAGGGAACTGGAAAAGA
GTGGGTCTGTGAGTCGAATTTCCTTTCGTGTTAAGGTGAAACTTGTGTAGGTCTCTGTG
ACACCTGACATGGCTATTCTTTCGCTCGTCGGGAAACAGCAACGTAATTTAGTCGGTAT
TTCTGGTCGTATGTTCTCATCCCTCGCCGAGGGCAATATCAACATTGAGATGATCAGCC
AAGGCGCGTCT

>Hom3 (FG534)_*Calocera cornea*
CTGGAGGACTCTGGCGGCCGGCGGATGCGGGACCGGCTCGTTGCCGAGCTGAGCAAG
ATCGGCCAGGTCACGCTCAGCCCGGAGATGGCGATCCTGTCGCTGGTGGGCAGCCAGA
TGCGCCACATGGTCGGCATCTCCGGCCGCATGTTCACCACGCTCGCCGAGGGCAACAT
CAACATCGAGATGATCAGCCAGGGCGCGAAC

>Hom3 (FG534)_*Dacryopinax primogenitus*
CTGGAGGACTCTGGGGGAAAACGCATGCGCGATCGACTGGTGCACGAACTGAGCAAG
ATAGGCCACGTCACCCTCTCCCCAGGGATGGCGATCCTCTCGCTCGTGGGGAGCCAAA
TGCGGCACATGGTCGGTATCTCCGGGCGGATGTTCACCACTCTCGCCGAGGGGAATATT
AACATTGAGATGATCAGTCAGGGCGCGAAC

>Hom3 (FG534)_*Rhizopus microsporus*
CTATCCATAGATGTCACTGAGAATAGTTTAAGTAGAGCTCTAGTGGAATTAGAAAGATT
AGGAACAGTAAGTGTTCACATGAAACCCATCCTGATGTCTTGGTTTTAGGTCGATGTTA
TCCGAGATATGGCCATTGTGTCTCTTGTAGGCAAACAGATGAAGAATATGATCGGTATA
GCCGGTGAAATGTTCTCTTCAGTGGCGGAAGCTGGAGTTAGTTTAGAAATGATTTCAC
AAGGTGCATCA

>Hom3 (FG534)_*Agaricostilbum hyphaenes*
ATGCACGGTGTTTTCCAGAGACACTTGCTGGATCGAGTCGTCAAAGACCTCAGCGTAG
TCGGCGAAGTGTCCATGACCAAGGACATGACCATCCTGTCCCTGGTGGGCAAATTGAA
GAACATGGTCGGCATCGCTGGCAAGATGTTCACGGTACTGGCTAATGGCGGCATCAAC
ATCGATGTCATCTCTCAAGGAGCCTCA

>Hom3 (FG534)_*Asperigillus triticus*
CTTCACTCGGTAATCATTGACGAGGATCTCAAGGGCGCCTTGCACGACCTCCAGAGAT
ACGGTACCGTTGACATCATCCCGGAAATGGCCATCCTCAGCCTTGTTGGGAAGCAGAT
GAAGAATATGATTGGGGTCGCCGGCAAAATGTTTTCGACCCTCGGGGAGAATAACGTT
AATATAGAGATGATTTCTCAAGGCGCGAGT

>Hom3 (FG534)_*Neurospora crassa*
GTGCACATCAATGCCGAGATGACCAACTTTGACGCGGCCGTCAAGGACCTGGGCGACT
GCGGCGATGTGAGCGTCCTCCATGGTCTGGCCATCCTCAGTCTGGTGGGCGCCGAGAT
GAAGAACATGATTGGTATTTCGGGAAAGATGTTTTCGACGTTGGGTGATCACAGAATC
AATATTGAGATGATTTCTCAGGGTGCCAGC

>Hsp60 (FG691)_*Boletus edulis*
GGTGCCCGGTAAGTTTCCCTCTCTTTGCCCATGTCGCTCACACGGACAGTCTCATTCAA
GACGTCGCCCAAAAGACGAACGAAATTGCGGGCGATGGGACAACGACCGCGACTGTC
TTGGCTCGCGCCATTTACGCCGAGGGTGTCAAGAACGTTGCTGCGGGATGTAACCCTAT
GGACCTTCGTCGTGGGTCGCAAGCCGCTGTTGACCGTGTCGTCGAGTTCCTGTCTGTC
AATACCAAGAAGGTGACGACGACTGCCGAGATTGCTCAAGTCGCGACCATCTCTGCCA
ATGGTGACACGCACGTCGGCAACTTGATTGCCCAGGCC

>Hsp60 (FG691)_*Gymnopilus chrysopellus*
GGTGCTCGGTAAGCCTTTCGTGCTCGTTCTTCAACTCATTACCATACAGTCTGGTCCAG
GATGTCGCCCAGAAGACCAACGAGATTGCTGGTGACGGAACCACCACCGCGACCGTT
CTAGCTCGCGCCATCTACTCAGAAGGTGTCAAGAACGTCGCTGCTGGCTGCAACCCCA
TGGACCTCCGCCGAGGCTCCCAGGCTGCCGTCGACCGTGTTGTAGAGTACCTCTCTGC
CAACACCAAGACCATCACCACCACGGCCGAAATTGCCCAGGTCGCCACCATTTCTGCC
AACGGTGACACCCACGTTGGCAACCTCATTGCCCAGGCT

>Hsp60 (FG691)_*Serpula lacrymans* var. *lacrymans*
GGTGCTCGGTAAGCTTTGGAGTTGTTGCGTTTACGCTAACGTCTTTCAGTCTTGTCCAA
GATGTAGCTCAAAAGACCAACGAGATTGCCGGTGATGGTACCACCACCGCCACTGTCC

TCGCTCGTGCTATCTACTCTGAGGGTGTTAAGAATGTCGCCGCAGGGTGCAACCCCATG
GATCTTCGTCGTGGTTCCCAGGCTGCTGTTGACCGCGTCGTTGAGTTCCTCTCTGCCAA
CACCAAGACTATCACAACCACCGCCGAGATTGCCCAGGTCGCGACTATCTCTGCCAAT
GGCGACATCCACGTCGGCAACCTCATTGCGCAGGCC

>Hsp60 (FG691)_*Laccaria bicolor*
GGTGCTCGGTAACTACCCCTTCCCCTCGTCCTCTTTCTCACATTTCCAGCCTTGTCCAA
GACGTTGCACAAAAGACAAACGAAATCGCAGGTGATGGAACAACAACCGCCACCGTC
CTCGCTCGCGCCATCTACTCCGAGGGCGTCAAGAACGTCGCGGCAGGCTGCAACCCCA
TGGACCTCCGCCGCGGTTCCCAAGCAGCCGTCGACCGCGTCGTCGAGTTCCTCTCCGC
CAACACAAAGACCATCACCACCACCGCAGAGATTGCTCAAGTCGCCACCATCTCCGCA
AATGGCGACACCCACGTCGGCAACCTCATTGCGCAGGCC

>Hsp60 (FG691)_*Phanerochaete carnosa*
GGTGCCCGGTGAGTAACTACTCTATCCCACGGATTACTGACTTTTGCAGTCTTGTCCAG
GATGTCGCGCAAAAGACTAACGAGATTGCTGGTGATGGCACTACCACGGCCACTGTCC
TCGCTCGTGCAATTTATTCGGAGGGTGTAAAGAACGTTGCTGCTGGCTGCAACCCAAT
GGACCTCCGCCGTGGCTCGCAGGCTGCTGTAGACCGTGTTGTCGAATTCCTCTCGTCG
CAAGCCAAGACAATTACCACCACCGCTGAGATTGCTCAGGTCGCCACTATCTCTGCTAA
TGGTGATACCCACATCGGCAACCTCATTGCCCAAGCC

>Hsp60 (FG691)_*Trametes versicolor*
GGTGCTCGGTCAGTAAAACCCCGCGTTCGTTCGCCACTCATCCATTTAGTCTCGTCCAG
GATGTTGCGCAAAAGACGAACGAGATTGCCGGTGACGGCACGACCACCGCGACCGTC
CTCGCGCGTGCCATCTACTCGGAGGGTGTCAAGAACGTCGCTGCTGGCTGCAACCCCA
TGGACCTCCGCCGTGGTTCCCAGGCCGCCGTCGAGCGTGTCGTCGACTTCCTCTCGAA
GCAGGCAAAGACGATCACCACGACCGCCGAGATTGCCCAGGTCGCAACCATCTCCGC
GAACGGCGACGCGCACGTCGGCAACCTCATTGCGCAGGCC

>Hsp60 (FG691)_*Schizophyllum commune*
GGTGCCCGGTGCGTTCCCCTCCGCGTTTTCTCCTCTACTTACACCTCAGCCTCGTTCAG
GATGTCGCGTCGAAGACTAACGAGATTGCCGGTGACGGTACGACGACCGCGACGGTC
CTCGCACGCGCCATTTACTCCGAGGGTGTCAAGAACGTCGCTGCTGGCTGCAACCCCA
TGGACTTGCGCCGTGGCTCGCAGGCCGCCGTCGACCGCGTTGTCGAGTTCCTCTCCTC
GCAGGCCAAGACGGTCACCACCACCGCCGAGATTGCGCAGGTCGCGACCATCTCCGC
GAACGGCGACACCCACGTCGGCAACCTCATTGCCCAGGCC

>Hsp60 (FG691)_*Coprinopsis cinerea*
GGTGCTCGGTACGTGTTCCCGCATTCTCCCGCCATAATTGACGCCCCAGTCTCGTCCAG
GATGTCGCGTCAAAAACGAACGAAGTCGCTGGTGACGGTACCACCACGGCCACCGTC
CTCGCCCGTGCCATCTACTCGGAGGGTGTGAAGAACGTTGCTGCTGGCTGCAACCCCA
TGGACCTCCGCCGAGGTTCCCAAGCCGCCGTCGACCGCGTCGTCAACTTCCTTTCCCA
ACACGCCAAGACTGTCACCACCACCGCTGAAATCGCCCAAGTCGCCACCATTTCTGCC
AACGGCGATACCCACGTTGGTAACCTCATCGCCCAGGCC

>Hsp60 (FG691)_*Pluteus cervinus*
GGTACCCGGTAAGCTTTGTCATTTTTTTTTTTGTCCGCTAACGCCCTTAGACTCATTCAAG
ACGTTGCTTCGAAGACAAACGAAATTGCTGGTGACGGAACAACCACTGCGACCGTCT
TGGCTCGTGCCATCTACTCCGAAGGTGTCAAGAATGTCGCAGCTGGTTGCAACCCCAT
GGACCTCCGTCGCGGTTCCCAAGCGGCTGTCGACCGTGTCGTGGAATTCCTTACGGCC
AACGCAAAAACTGTGACCACCACTGCTGAGATCGCACAAGTCGCTACCATCTCTGCGA
ACGGTGACACTCACGTCGGTAACCTCATTGCTCAGGCT

>Hsp60 (FG691)_*Mycena crocata*
GGCGCTCGGTATGTTTTTTTCTTTCTTTTTCTTTCTTACTTGAATACAGTCTCGTCCAGGA
TGTGGCTCAAAAGACAAACGAGATTGCTGGAGATGGGACAACCACGGCCACGGTCCT
TGCGCGCGCCATCTACTCTGAGGGTGTCAAAAACGTCGCTGCAGGCTGCAACCCCATG
GACTTGCGCCGCGGTTCGCAGGCTGCTGTCGAGCGCGTCGTGCAGTTCCTCTCCTCGC
ATACGAAGACGATCACCACCACCGCAGAGATTGCCCAGGTCGCCACCATCTCCGCCAA
CGGCGACTCGCACGTCGGTAACCTCATTGCCCAGGCC

>Hsp60 (FG691)_*Hygrophoropsis aurantiaca*
GGCGCCCGGTAGGCCCCTCTCACCCCCTCTGCATTCCTGAGCCTCTTAGTCTCGTCCAA
GATGTTGCGCAAAAAACTAACGAGATAGCTGGTGATGGTACTACCACCGCAACTGTTC
TCGCACGTGCTATCTACTCCGAAGGTGTCAAGAACGTCGCAGCAGGATGCAATCCTAT
GGATCTCCGTCGCGGTTCACAAGCCGCTGTTGACCGTGTAGTTGAATTCCTTGCAGCC
AACACGAAAACCATCACCACTACGGCAGAGATAGCACAGGTCGCCACCATTTCTGCAA
ATGGTGACACCCATGTCGGAAACCTCATTGCTCAGGCT

>Hsp60 (FG691)_*Agaricus bisporus* var. *bisporus*
GGGGCTCGGTAGGTTTTCCCCATTTTTTTAAAAATTCATAATATTTTAGTCTCGTGCAGG
ATGTCGCTTCCAAGACCAACGAGATTGCAGGTGACGGCACGACAACGGCCACCGTTTT
GGCTCGTGCTATTTATTCAGAGGGTGTGAAGAACGTCGCAGCTGGCTGCAATCCTATGG
ACCTCCGTCGAGGTTCCCAAGCGGCTGTCGACCGTGTCGTAGAGTTCCTTTCTCAGAA
CGCAAAAACAATCACCACTACTTCTGAAATTGCTCAGGTTGCCACCATCTCAGCGAAC
GGTGACACTCACGTGGGCAACCTCATCGCCCAGGCT

>Hsp60 (FG691)_*Stereum hirsutum*
GGTGCAAGGTATGCCGTCGTTCGTTGGGATTTACTATTATGAATCCTAGGTTGGTCCAA
GACGTCGCTCAAAAGACGAACGAAATTGCCGGTGACGGTACTACGACCGCCACCGTG
CTTGCTCGTGCCATTTACGCCGAGGGTGTGAAGAACGTCGCAGCCGGTTGCAATCCCA
TGGATCTTCGTCGTGGCTCTCAGGCTGCCGTCGAGCGGGTTGTCAGCTTCCTTTCTGCC
AACACCAAGACCATCACTACCACCGCTGAGATTGCTCAGGTCGCAACCATCTCAGCAA
ACGGCGATACTCACGTCGGTAACCTCATTGCGCAGGCG

>Hsp60 (FG691)_*Agrocybe pediades*
GGTGCTCGGTATGTATACCTACTTGTGCCAGTCATACTAATGATCTCAGTCTCATCCAAG
ACGTTGCTGCTAAGACCAACGAAATCGCCGGAGACGGCACGACCACCGCCACCGTTC

TCGCCCGCGCCATTTACTCGGAGGGTGTCAAGAACGTCGCTGCTGGATGCAATCCAAT
GGATCTCCGCCGTGGATCCCAAGCTGCCGTCGACCGTGTCGTTGAGTTCTTGTCCGCC
AACACCAAAACTATCACCACCACCGCGGAGATTGCCCAAGTCGCTACCATCTCAGCCA
ACGGCGACACCCACGTCGGTAACTTGATTGCTCAGGCT

>Hsp60 (FG691)_*Gymnopus androsaceus*
GGTGCACGGTAAGTATTTTGACACGTAAGGTCTGAGACTAACTTTTCAGTCTTGTCCAA
GACGTGGCTCAAAAGACCAATGAAATAGCTGGTGACGGCACAACTACTGCTACTGTCC
TTGCACGCGCCATTTATTCCGAAGGTGTCAAAAATGTTGCAGCAGGATGCAATCCCATG
GACCTTCGTCGCGGTTCTCAGGCTGCTGTCGAACGTGTCGTTGAATTCCTAGCTTCGCA
GACCAAAACGATCACAACCACTGCCGAGATTGCCCAGGTCGCAACCATTTCAGCAAA
CGGTGATACTCACGTCGGTAACCTCATTGCCCAGGCC

>Hsp60 (FG691)_*Marasmius fiardii*
GGTGCACGGTAAGTAAATATGAACTTGCGGTAGTTCTAACTTTTCTTAGTCTCGTGCAA
GACGTCGCACAAAAAACGAACGAGATTGCAGGTGACGGTACCACCACTGCAACTGTT
CTTGCACGCGCCATCTACTCCGAGGGTGTCAAGAACGTTGCAGCCGGCTGCAATCCCA
TGGATCTTCGTCGCGGTGCACAAGCGGCCGTTGAACGTGTCGTCGAGTTCCTTGCGAC
ACAAACCAAGACTATCACCACCACCGCGGAAATTGCCCAGGTCGCCACCATTTCTGCT
AACGGCGATACCCACGTCGGAAACCTGATCGCTCAGGCT

>Hsp60 (FG691)_*Amanita muscaria*
GGCGCACGGTAATTTTCTCTCATTTCCCTCACGACCTAATAAATAATAGCCTCATCCAAG
ATGTCGCACAAAAGACAAACGAGATTGCTGGTGACGGTACAACCACCGCAACCGTTC
TCGCACGTGCCATCTACTCCGAGGGTGTCAAGAACGTCGCTGCCGGGTGCAACCCCAT
GGATCTCCGACGTGGTTCACAAGCTGCTGTCGAGCGTGTCGTTGACTACCTCGCCAAG
CACGCCAAGACAGTGACGACGACTGCTGAAATTGCACAGGTCGCCACCATTTCAGCC
AACGGCGATACTCACGTTGGTAACCTCATTGCCCAAGCA

>Hsp60 (FG691)_*Lactarius quietus*
GGCGCACGGTATGTTTTTCTATTTCGTCTTAATGCACATTGACTTGTAGGCTAGTGCAGG
ACGTTGCACAAAAAACAAACGAGATTGCCGGCGATGGCACGACCACGGCAACAGTTC
TCGCGCGCGCTATCTATGCTGAGGGTGTTAAGAACGTCGCTGCTGGCTGCAACCCGAT
GGACCTTCGTCGTGGTTCCCAGGCTGCAGTCGAGCGTGTTGTCGAGTTCCTTGCGAAG
AACACTAAGACAATAACGACTACCGCCGAGATTGCTCAGGTGGCAACCATTTCCGCCA
ACGGTGACACTCACGTCGGTAACCTCATTGCGCAAGCC

>Hsp60 (FG691)_*Fomitopsis pinicola*
GGTGCTCGGTGAGTGTTTTTAACGAGTCTGTAGTTGCTTACGCGTTCAGCCTCGTCCAG
GACGTTGCGTCCAAGACAAACGAGACTGCGGGTGACGGTACGACGACAGCGACCGTA
CTCGCGCGTGCCATCTACTCCGAGGGTGTCAAGAACGTTGCTGCGGGCTGCAACCCCA
TGGACCTCCGCCGAGGCTCGCAGGCAGCCGTCGAGCGCGTCGTCGAGTTCCTCTCCGC
ACACGCAAAGACGATCACGACCACGGCAGAGATTGCGCAAGTAGCCACTATCTCCGCT
AACGGCGACGCCCACGTCGGCAACCTCATCGCGCAGGCT

>Hsp60 (FG691)_*Tricholoma matsutake*
GGTGCTCGTCTTGTCCAAGACGTCGCACAGAAGACTAACGAGATGGCTGGAGACGGC
ACAACCACCGCCACAGTCCTGGCAAGGGCCATATATTCCGAAGGTGTAAAGAACGTAG
CAGCCGGGTGTAATCCCATGGACCTTCGTCGCGGCTCTCAAGCAGCTGTTGACCGCGT
TGTTGAGTACCTTTCTGCACATACTAAAACCATCACCACCACCGCTGAGATCGCACAAG
TCGCTACCATCTCGGCCAATGGTGACCTCCACGTCGGCAACCTCATTGCCCAAGCC

>Hsp60 (FG691)_*Fomitiporia mediterranea*
GGTGCGCGGTAAGCCATGGCTCTTGGTACATGCGCTCATTGTAATGTAGTCTCGTACAA
GACGTCGCGCAGAAGACGAACGAGACTGCTGGTGACGGAACGACAACCGCAACCGT
CCTTGCTCGTGCGATCTATGCAGAAGGCGTGAAGAACGTCGCTGCTGGCTGCAATCCG
ATGGATCTTCGTCGAGGTGCTCAGGCGGCTGTTGACCGCGTAGTTGACTTCCTGTCAA
AGAACACGAAAGACATTACGACGACAGAGGAAATTGCACAAGTTGCAACTATTTCTGC
GAATGGTGATACGCATATTGGCAATCTCATCGCGACAGCC

>Hsp60 (FG691)_*Auricularia delicata*
GGTGCACGGTACGCGGTTTCTTCCCGGCACCCTGCCATGATTGGCTCAGGTTGGTGCA
GGACGTCGCGTCCAAGACAAACGAAATCGCTGGCGACGGCACGACTACCGCGACGGT
GCTCGCGCGCGCCATCTACGCGGAGGGCGTCAAGAACGTCGCCGCGGGCTGCAACCC
GATGGACCTGCGGCGCGGCGCCCAAAAGGCCGTCGACAAGGTCATCGCGTTCCTCGA
ACAGAACAAGCGCGAAATCACAACGTCCGAGGAGATCGCCCAGGTCGCGACCATCTC
CGCGAACGGCGACACGCACGTCGGCCAGCTCATCGCGACGGCG

>Hsp60 (FG691)_*Calocera cornea*
GGTGCTCGGTATGTCTGTCCTTACTCGGCTGTCGGCTGACGTGTCGTAGTCTCGTTACC
GATGTCGCGAACAAGACCAACGAGATCGCTGGTGACGGCACAACCACCGCAACTGTC
CTCGCCCGCGCTATCTACGCCGAGGGTGTCAAGAACGTCGCAGCCGGCTGCAACCCCA
TGGACCTCCGCAAAGGTAGTCAGAAGGCGGTCGAGAAGGTGATCGAGTACCTCGAGA
AGCACAAGCGGGTGATCACAACCTCGGAGGAGATCGCCCAGGTCGCCACCATCTCTG
CGAACGGCGATGTCCACGTCGGCCAGCTTATCGCCACCGCG

>Hsp60 (FG691)_*Dacryopinax primogenitus*
GGAGCTCGGTATGGACCATTTTTATCGTGTTGTTAATTGACTCTTGCAGTCTTGTTACCG
ATGTTGCTAACAAGACCAACGAAGTGGCCGGTGACGGGACAACAACCGCGACCGTCC
TTGCCCGCGCTATCTACTCCGAGGGCGTCAAGAATGTCGCTGCTGGTTGCAACCCTATG
GACCTCCGTCGAGGCAGCCAAAAGGCCGTCGAGAAGGTGATCGAGTACCTCGAGAAG
AACAAGCGAGTTATCACTACTTCCGAGGAGATCGCCCAGGTCGCGACTATCTCCGCCA
ATGGCGATACCCATGTTGGCCAGCTAATTGCTACCGCT

>Hsp60 (FG691)_*Asperigillus triticus*
GGTGCCCGTCTCCTGCAGGATGTGGCCTCCAAGACCAACGAGATCGCCGGTGACGGTA
CCACCACCGCCACCGTCCTTGCCCGTGCCATCTTCTCCGAGACCGTCAAGAACGTTGC

TGCCGGCTGCAACCCCATGGACCTGCGCCGTGGTATCCAGGCCGCCGTCGATGCTGTC
GTCGACTACCTCCAGCAGAACAAGCGTGACATCACCACCGGTGAGGAGATCGCCCAG
GTCGCGACCATCTCCGCCAACGGTGACACCCACATCGGTAAGCTCATCTCCACCGCT

>Hsp60 (FG691)_*Neurospora crassa*
GGTGCCCGCCTCATCCAGGAGGTCGCCGGCAAGACCAACGAGGTCGCCGGTGACGGT
ACCACCTCCGCCACCGTCCTCGCCCGCGCCATCTTCTCCGAGACCGTCAAGAACGTCG
CTGCCGGCTGCAACCCCATGGATCTCCGCCGCGGTATCCAGGCTGCCGTCGAGGCCGT
CGTCGAGTACCTCCAGGCCAACAAGCGCGATGTCACCACCTCCGAGGAGGTTGCCCA
GGTCGCCACCATCTCCGCCAACGGCGACAAGCACATTGGTGAGCTGATTGCCTCGGCC

>Hsp60 (FG691)_*Gautieria morchelliformis*
GGTGCCCGGTTAGTGCAGGATGTCGCGCAGAAGACCAATGAGACTGCTGGAGATGGA
ACTACGACCGCGACTGTTCTCGCGCGTGCTATCTATGCCGAGGGAGTGAAGAATGTCG
CTGCAGGGTGCAATCCTATGGACCTCCGTCGTGGCTGTCAGGCTGCTGTGGACCGCGT
TATAGCCTTCCTTGAAAGTAACAAACGCAGTATTACTACTTCGGCCGAGATAGCCCAAG
TCGCCACAATCTCTGCTAATGGCGACGCGCATGTAGGCGAGCTTATTGCGCAGGCT

>Hsp60 (FG691)_*Ramaria acris*
GGTGCACGTTTAGTTCAAGATGTAGCTCAGAAAACGAACGAAGTTGCCGGAGACGGC
ACGACTACAGCAACTGTGCTTGCGCGCGCTATTTACGCTGAAGGTGTAAAGAACGTCG
CTGCAGGTTGTAATCCCATGGATCTTCGTCGGGGTTGTCAGGCTGCTGTAGAACGCGTT
ATTGCCTTCCTCGAGAGCAACAAGCGTAGCATCACTACCTCGGCCGAAATTGCACAAG
TAGCTACGATTTCCGCCAATGGTGACGCCCATATCGGTGAACTTATTGCTCAAGCC

>Hsp60 (FG691)_*Rhizopus microsporus*
GGTGCTAGGTAAGTGATGCTCATTGTCAATCCATGGTACTTAGAAATAGACTTGTTCAA
GACGTTGCAAGCAAGACAAACGAAATTGCCGGTGATGGTACTACCACTGCTACCGTCT
TGACTCGTGCCATCTTTACTGAAGGTGTCAAGAATGTTGCTGCTGGCTGCAATCCTATG
GATCTCCGTCGCGGTGCTCAAATGGCTGTTGATGCCGTTGTTGACTTCTTGAAGGCTCA
CACCAAGGTTATCACAACTCCTCAAGAAGTTGCTCAGGTTGCTACCATCTCTGCTAACG
GTGATAAGCATGTCGGTAACATGATTGCTCAAGCT

>Hsp60 (FG691)_*Agaricostilbum hyphaenes*
GGCGCTCGGTGAGGCTCTGTCTTACGACGGTGAGGGATGTGTTCGGCAGTCTTGTGCA
GGACGTTGCCAACAAAACCAACGAAGTTGCTGGTGACGGTACCACCACAGCTACTGT
TTTGGCCAGAGCCATCTACGCAGAGGGTGTCAAGAACGTCGCAGCTGGCTGCAACCCT
ATGGACTTGCGAAGAGGGTCACAGCAAGCTGTTGAGTGGGTCGTTGCCTTCCTCGAG
AGCAATAAGAGAGACATCACCACATCAGCAGAGATTGCTCAGGTTGCTACCATCTCAG
CAAATGGTGACTCACACGTTGGAGCGCTCATTGCTCAGGCC

>Ils1 (MS444)_*Agrocybe pediades*
CTCAAAAACTATCCCGATCCTAACCTCATCATTGACAAATATGGCGCCGATGCAACAAG
GTGGGTTCTCTTTCCCTATCATCATTGACTATCTGTGTACAGAATGTTCCTTGTCAACTC

GCCGATCGTGAGGGGAGACAATCTCCGTTTCCGTGAGGAGGGTGTCCGAGAAGTCATC
TCGCGCGTATTGCTGCCATGGTTGAACTCGTTCCGCTTCTTCATGGGTCATGTCGCCTTG
CTGAAGAAGACCACCGGCTTCGACTTCAAGTATAATGCACATGCGCCATTGCCAAAC

>Ils1 (MS444)_*Pluteus cervinus*
TTGAGAAATTATCCAGATCCCAATATCATCATTGATAAGTACGGTGCTGATGCCACAAG
GTAAGATCGATAGCTTCGTCGGGTATTTGACCTGTGACATAGGATGTTCTTGGTAAATTC
CCCCATTGTGCGTGGAGACAACCTACGATTCCGAGAAGAGGGTGTGCGCGAGGTTGTA
TCGCGGGTCCTCCTGCCCTGGCTCAACTCCTTCCGGTTCTTCTTGGGACATGCGGCTAT
CCTGAAGAAGGGCACAGGACACGACTTCAGATATAACGCTCATGCACCACTTCCGAAT

>Ils1 (MS444)_*Agaricus bisporus* var. *bisporus*
CTGAAAAACTATCCTGATCCTAATATAATTATTGAGAAGTATGGCGCCGATGCGACTAGG
TATGCCGCATGCTTCTGCCTTCGATATTTGGATACAGGATGTTCCTTGTCAATTCGCCCA
TTGTCCGTGGCGACAACCTGCGATTCCGTGAAGAGGGCGTGCGCGAAGTCATATCCCG
AGTTCTCCTTCCTTGGCTCAACTCTTTCCGCTTTTTCTTGGGGCACGTTGCTCTGTTGA
AAAAAACTAGCGGTGTTGATTTCAAATATAACCCCCATGCGCCCCATCCGACG

>Ils1 (MS444)_*Laccaria bicolor*
CTGCGAAATTATCCTGATCCAAACATCATCATCGACAAGTATGGTGCTGACGCGACAAG
GTGGGAGAACTAGTGAATTTTTCGGAAGCTCATTATGCCCAGAATGTTCCTCGTCAATT
CGCCTATTGTACGTGGTGACAATCTGCGGTTCCGTGAGGATGGTGTCCGCGAAGTCATC
GCCCGCGTTCTTTTACCCTGGCTCAACTCCTTCCGATTTTTCTTGGGGCACGTTGCGCT
GTTCAAGAAGGTAAATGGCATCGATTTCACCTACAACGCACATGCCCCACTGCCAAAC

> Ils1 (MS444)_*Tricholoma matsutake*
TTAAGGAATTATCCCGATCCAAATTTAATCATTGATCAATATGGTGCCGATGCGACAAGG
TAAAGTGTGTAATCAGTATGATGTCTCTCACGCGGACGCAGAATGTTCCTCGTCAACTC
ACCCATTGTTCGCGGGGATAATCTTCGTTTCCGTGAAGAGGGTGTTCGCGAGGTGATCT
CACGGGTTCTTCTGCCATGGCTTAACTCTTTCCGGTTCTTCTTGGGCCATGTCTCTTTGT
TTAAGAAAGTCAGTGGCATGGATTTCAAGTACGATCTACATGCGCCAGTGCCCAGC

>Ils1 (MS444)_*Fomitopsis pinicola*
CTGAAGAACTACCCCGACCCCAATCTCATCATCAACCAGTACGGCGCTGATGCCACGC
GGTATGCCTTCCATCGCCCCCTGCCTGGCTCACTCTCCCTCAGGATGTTCCTCGTTAACT
CGCCCGTTGTCCGTGGTGACAACCTGCGCTTCCGTGAAGAGGGCGTTCGCGAGGTTGT
CTCCCGTGTGCTCCTCCCCTGGCTCAACTCGCAGCGCTTCTTCCTTGGTCAGGCCGCCC
TGCTCAAGAAGAACACGGGCCGCGACTTCCAATACGACGCACACGCGCCCGTATCTTC
C

>Ils1 (MS444)_*Stereum hirsutum*
CTGCGTAATTACCCGGACCCGAACTTGATCATCAACCAGTATGGTGCTGATGCTACCAG
GTACGCCACATTCTTCTTTCTTTAGCGACTAACCGTAAACAGAATGTTCCTCGTCAACT

CCCCCATCGTCCGAGGAGACAACCTCCGCTTCCGCGAAGAAGGCGTCCGCGAAGTCG
TCTCCCGCGTTCTCCTACCCTGGCTCAACTCCTTCCGGTTCTTCCTCGGCCAAGTCGCA
CTGCTCAAAAAATCCACAGGCCACTCCTTCAAATACGACGCCCACGCGCCCGTATCGA
AC

>Ils1 (MS444)_*Lactarius quietus*
CTAAAGAACTACCCTGACCCCAACTTGATTCTCGATCGGTACGGGGCTGATGCTACAA
GGTCTGTTTTGTGCTACCGTTGAGAGATAATATCCATCGGCAGGATGTTCCTCGTCAAC
TCACCCATTGTCCGTGGTGACAATCTCCGCTTCCGGGAAGAGGGCGTGCGAGAGGTCA
TCTCGCGCGTCCTTCTTCCTTGGCTCAACTCTTTCCGTTTCTTCATTGGCCAGGTCGCG
CTGTTCAAGAAGGCTACAGGGCACGAGTTCCGGTATGACGCCCATGCACCTTTGTCGG
GC

>Ils1 (MS444)_*Trametes versicolor*
CTGAAGAACTACCCCGACCCCAACCTCATCATCGACCAGTACGGCGCCGATGCGACAA
GGTAGGCAGCGTGCACAGATATCGTGGTCACTAACGTAAACAGGATGTTCCTCGTCAA
CTCGCCTATCGTCCGCGGCGACAACCTGCGTTTCCGGGAAGCTGGCGTCCGCGAGGTC
GTCTCACGTGTACTCCTCCCGTGGCTCAACTCCTTCCGCTTCTTCCTGGGGCAGGCTGC
GCTCTTGCAGAAGACAACCGGGCGCGCATTTGTATACCACGCGCATGCGCCCGTGTCG
CAG

>Ils1 (MS444)_*Hygrophoropsis aurantiaca*
TTGCGGAACTTTCCTGATCCGAACTTGATATTCGATCTCTACGGCGCTGACGCAACAAG
GTGCGGACTCACACATACTCCTAAACTGACTTCCTCGCACAGAACGTTCCTCGTCAAC
TCCCCCATCGTGCGCGGCGAGAACCTCCGATTCCGCGAGGAAGGCGTCCGCGAAGTC
GTATCGCGCGCGCTTCTCCCGTGGCTCAACTCCTTCCGCTTCTTCCTCGGTCAAGTCTC
CCTCTTGCGCAAAACGACCGGCGTCGCGTTTGTCTACAACCCGCACGCCCCGCTATCA
AAC

>Ils1 (MS444)_*Gymnopus androsaceus*
CTTAAAAACTACCCTGATCCTACTCTTATCATCGACAAGTATGGAGCCGACGCTACCAG
GTAAATTATTTCATGATATAGTAGATTGACGAGTCTGTGTAGGATGTATCTCGTCAACTC
CCCGATTGTTCGCGGAGACAACTTGAGATTCCGAGAAGAGGGTGTCAAGGACGTTATC
TCCCGAGTCCTCCTTCCATGGCTCAACTCTTTCCGCTTCTTCCTCGGTCACGTCGCCAT
CTTGAAGAAGGCGACAGGTATCGACTTCAAGTATGACCCTCATGCTCCTCTTCCCAAC

>Ils1 (MS444)_*Coprinopsis cinerea*
TTGAAGAACTACCCTGATCCCAATCTGATCATCGACAAATATGGTGCTGATGCTACCCG
GTAAGGCATTCCCGCCGAGACTGATGGTCTTATTCCCCATAGAATGTTCTTGGTCAACT
CGCCAATCGTTCGCGGCGACAACTTGCGCTTCCGCGAGGAAGGTGTCCGGGATGTCAT
TTCCCGTGTGCTTCTCCCGTGGCTCAACTCCTTCAGGTTCTTCCTCGGCCACGCTGCGC
TTTACAAGAAAGCATTCAAGGAAGATTTCGTCTACAATGCCCATGCACCCCTCCCCAAC

>Ils1 (MS444)_*Gymnopilus chrysopellus*

CTGAAGAATTATCCAGATCCCAATCTCATCATTAATCAATATGGCGCTGATGCAACAAGG
TGAGGAGTGGTCGAACCTTGGAATCTCATGGATCTTCTTAGAATGTTCCTCGTGAACTC
GCCTATCGTACGTGGCGACAACCTTCGCTTCCGTGAAGAGGGTGTCCGAGAGGTCATC
TCTCGTGTCCTGCTTCCGTGGTTGAATTCCTTCAGATTCTTTTTGGGCCACGCTGCCCT
GCTGAAGAAGACTACAGGGACCGACTTCAAGTATAATGCTCATGCTCCGTTACCCAGC

>Ils1 (MS444)_*Serpula lacrymans* var. *lacrymans*
AAGAAGAATTACCCAGATCCCAATCTTATTCTTGACCAGTACGGCGCCGATGCCACAAG
GTTTGTCCTACCTCTGCCTGATTGGTTGATTTCATCCCAGAATGTTCCTCGTTAACTCG
CCCATCGTTCGTGGGGACAACCTTCGTTTCCGCGAAGAAGGTGTGCGAGAAGTGGTG
ACCCGCGTGCTCCTCCCGTGGCTCAATTCCTTCCGTTTCTTCCTCGGCCAAGTCGCGCT
TTTACAGAAAACGACGGGTGTTTCTTTCACGTACAACCCACATGCGCCGCTCTCGAAT

>Ils1 (MS444)_*Marasmius fiardii*
CTCAGAAACTTCCCCGATCCTAACCTTATCATCAACCAGTACGGTGCTGACGCAACCAG
AATGTATCTCGTCAATTCTCCAATCGTTCGTGGAGACAACCTTCGTTTCCGTGAAGAGG
GTGTCAAAGATGTCATTTCCCGAGTTCTTCTGCCATGGCTGAATTCTTTCCGATTCTTCC
TTGCCCAAGTCGCGCTTCTTAAGAAGACCACTGGGCATGAATTCAAGTACAATCCGCA
CGCGGGTCTTCCTCAG

>Ils1 (MS444)_*Phanerochaete carnosa*
CTGAAGAACTACCCGGACCCAAATCTCATCATAAATGAGTACGGTGCGGACGCGACGC
GCATGTTCCTTGTCAATTCCCCCATCGTGCGCGGCGACAACCTCCGTTTCCGCGAGGA
AGGCGTCCGGGAGGTTATCTCGCGCGTGCTCCTACCATGGCTGAACGCATTCCGATTCT
TCCTTGGGCAAGTGCAGCTGCACCGCAAGACGGGCTCGTTTGACTTCCAGTACAACGC
GCACGCGCCGGTGTCGAAT

>Ils1 (MS444)_*Amanita muscaria*
CTCAAAAACTACCCTGATCCCACCCTGATCATCGACAAGTATGGCGCAGATGCGACAA
GGTGAGGTCTTATGACCGTTTCTGGTACTGAAGGTTCGAAGAATGTTCCTTGTCAATTC
ACCAATTGTCCGCGGTGACAATCTAAGGTTCCGCGAAGAAGGCGTCCGAGAAGTCATA
TCCCGCGTCTTACTCCCATGGCTCAACTCGTTCCGCTTTTTCCTGGGGCACGCGGCGTT
GTTGAAGAAATCCACCGGACACGAATTTGTGTATAATAAGGATGCGCCTCTACCAAGC

>Ils1 (MS444)_*Mycena crocata*
GCCCGGAATTACCCTGATCCGACTCTCGTCATTGATAAAATGGGTGCAGATGCTACGAG
GTTGGTGTTTTCCTGTCGGTTAGAGTTAATAGTGCGCTCTAGAATGTTTTTGGTGAACT
CGCCCATTGTCCGCGGTGATAATCTGCGTTTCCGCGAGGAAGGTGTCCGCGAGGTCAT
CTCCAGAGTACTTTTGCCTTGGCTGAACTCTTTCCGGTTCTTCCTGGGACATGCTGCTC
TGTTCAAGAAGGTCACGGGCACCGACTTTGTATACAATGCCCACGCCACTGTCCCAAA
T
>Ils1 (MS444)_*Calocera cornea*
AAGAAGAACTACCCGGATCCTACCATCCTCGTGGATAAGCTGGGCGCCGATGCGCTGA
GGTATGTGGAGTTCTGGCTGGGAGGGTGCTCATTTGTCACAGAATGTTCCTCGTCAACT

CTCCCATCACCCGTGGCGAGAACCTGCGCTTCCGTGAAGACGGTGTGCGCGAGGTCGT
CTCCCGCGTCCTCCTTCCGTGGCTCAACTCCTTCCGCTTCTTCCTCGCCCAAGTGGCCC
TGCTCAAGAAGACGACCAACGTCGACTTCATGTGGGACCCACATGCAAAGCGCTCGC
CT

>Ils1 (MS444)_*Dacryopinax primogenitus*
AAGAAGAACTATCCGGACCCGAATCTGGTTGTAAACAAGTACGGTGCGGATGCGCTAC
GGTATGGGGTTGTCCCAATCTTCCAAACTCATATGGCGTTTAGAATGTTCCTCGTCAATT
CCCCCATTACCCGTGGTGAGAACTTGCGGTTCCGTGAGGATGGTGTCCGCGAGGTTATT
TCACGGGTGCTTCTACCCTGGGTCAACTCTTTCCGCTTCTTCCTGGCCCAAGTCGCGCT
GCTCAAGAAGACGACGAATGTGGATTTCATGTGGGAGCCACACGCCAAACGCTCCGC
C

>Ils1 (MS444)_*Auricularia delicata*
CTCAAAAACTACCCCGATCCGAACCTGGTCATTGAGCGCTACGGCGCGGATGCCACGC
GGCATGTCACTTGCCTCGACGAGCCCACTAAACTGTGTCTAGGATGTTCCTCGTCAATT
CGCCCATCGTGCGCGGCGACAACCTGCGCTTCCGCGAGGAGGGCGTGCGCGAGGTCG
TCGCGCGCGTGCTGCTCCCGTGGCTCAACTCGTTCCGCTTCCTGTTGGGCCACGCAGC
GCTGCTCGAGAAGGCACACGGCGTACGCTTCCAGTACCAGGCGCACCTCGCGCAGTC
CGGC

>Ils1 (MS444)_*Ramaria acris*
CTGAAAAATTATCCAGATCCCAATTTAATCATCAACCAATATGGCGCGGATGCTACACG
GTCCGTTGCAGTCTCCCGTTTTATCCTAATATGGGTGGTCAGCATGTTCCTCGTTAATTC
CCCCATTGTTAGGGGTGACAATCTTCGATTTAGAGAAGAAGGCGTTCGCGAAGTGATC
TCTCGGGTCCTGTTACCCTGGCTTAATTCTTTCCGGTTCTTCCTTGGACAAGTCAACCT
GCTGCGTAAAGAGGAAGGGCATTCGTTTGTGTACGATGCGCATGCACCACGATCAAAC

>Ils1 (MS444)_*Fomitiporia mediterranea*
TTGAAGAACTATCCTGACCCTACGGATATCATTGATCGTTATGGTGCGGACGCGACGAG
GTGCGTGCAAAAACCCGCCAAGAAGAAAACTTATGGTTTTAGGATGTACCTCGTAAAC
TCTCCGGTCACCCGCGCGGAGAACCTACGCTTCCGCGAAGCAGGCGTCCGCGAAGTC
ATCTCGCGTGTTTTCCTTCCCTGGCTAAATGCCTTCCGATTCTTCCTCGGGCAGGTCGC
ACTACACAAGAAAACAAGTGGATTGGAATTCAAGTATGATCCGCATGCGCCGGTGTCT
GCG

>Ils1 (MS444)_*Boletus edulis*
CTTAGAAATTACCCCGATCCGAACTTGATGTTTGAAAAGTATGGTGCTGATGCGGTCAG
GTTCGTGTCTCATGATCGAATGAACCAAAGGCTACTTTGCAGGATGTTCTTGGTGAACT
CGCCGATCGTACGTGGTGAGAACCTTCGGTTCCGCGAGGAGGGTGTGCACGACGTCGT
CTCTCGCGTCATGCTCCCCTGGGTTAACGCCTTCCGTTTCTTCCTCGGCCAAGCGAGTC
TTTTCCGAAAGACGACTGGGATCGAGTTCAAGTACAACCCTCACGCACCACTCTCGAG
C

>Ils1 (MS444)_*Schizophyllum commune*
TTGCGCAACTACCCGGACCCTAACCACATTCTCGACACATACGGAGCGGATGCCACCC
GGTAAACGCACAATCCGCTTCGTCTCCTAACACACCGAGTCAGAATGTACCTCGTCAA
CTCCCCCGTCGTCCGCGGCGACAACCTGCGCTTCCGCGAAGACGGCGTGCGCGAGGT
CGTCTCCAAGGTCCTCCTTCCCTGGCTCAACTCCTTCCGCTTCTTCCTCGGGCACTCCG
CCCTGCTCACCAAAACTACTGGCGCCGAGTTCAAGTACAACGCGCATGCGCCGCTGCC
GAAC

>Ils1 (MS444)_*Agaricostilbum hyphaenes*
CTCAAGAACTACCCTGACCCCAACCTAATCCTCGACCAGTATGGCGCAGACGCTCTCA
GACTCTTCCTGATCAACTCACCAGTTGTCCGAGGTGATAACTTGCGCTTCAGAGAAGC
AGGTGTCCGGGAAGTCGTCAGCAGAGTCCTCTTACCCTGGCTCAACTCTTTCCGCTTC
TTCCTCGGCCAGGCTGCCCTGCTCAAGAAGGAGTCTGGCCAAGAGTTCTACTACGATG
GTACAGCGGCTCAGTCAAGC

>Ils1 (MS444)_*Neurospora crassa*
CTCAAGAACTACCCCGACCCTACTCTTGTCATGAACAAGTATGGTTCGGATGCCCTCCG
TCTGTACCTTATCAACTCTCCTGTTGTGCGTGCCGAGCCGCTCCGCTTCAAGGAGGCTG
GTGTCAAGGAGGTCGTTGCCAAGGTTCTCCTTCCTCTGTGGAACTCGTACAAGTTCTTT
GAGGGCCAGGTTGCTCTCCTCAAGAAGGTGGAGAACGTCGACTATGTGTTTGACCCCA
AGATGGAGTCTTCCAAC

>Ils1 (MS444)_*Asperigillus triticus*
CTGAAGAACTACCCCGATCCCTCTCTCATCATGGACCGCTACGGCTCGGACGCTCTTCG
TCTGTACCTGATCAACTCGCCCGTCGTCCGCGCGGAGCCGCTGCGGTTCAAGGAAACC
GGTGTTAAGGAGATCGTTGCCAAGGTTCTCCTGCCCCTGTGGAACAGTTACAAGTTCT
TCGAGGGCCAGGCGGCTCTCTTCAAGAAGACCAACGGCATTGACTACGTCTTCAACCC
GGAGGCTGAAGAGACCAAC

>Ils1 (MS444)_*Rhizopus microsporus*
CTCCGTAACTACCCTGATCCCAATCTTGTCATTGACAAGTTCGGTTCTGATGCTCTCAG
ACTGTACCTCATCAACTCCCCTGTTGTTCGTGGTGAAACATTAAAGTTCAGAGAAGATG
GTGTCAAAGATGTAGTCAGCAAGGTCTTCTTGCCTTGGTACAATGCCTACAAGTTCTTC
CTTACTCAAACTGCTGTCTTGAAGAAGGAATTCGACTATGACTTCCAATATGATGCTCAT
ATCAAAAAGTCTGGC

>Ilv2 (FG595)_*Agaricus bisporus* var. *burnettii*
GCGCAACACTATCGATGGAGACATCCCCGATCACTTGTCTCTTCCGGTGGTTTAGGTGT
ACGTTTCCTCTCTCACTTGTCGACGTGTTCTTGACTCAGTTTACCTTTACAGACTATGGG
CTTTGGTCTGCCGGCAGCTATCGGTGCCAAGGTTGCTGCTCCTAATAAGACTGTTATCG
ACGTCGATGGCGACGCTTCGTTTAGTATGACTGCTATGGAACTCGCTACCGCTTCTCAA
TACGGTATCGGTGTCAAAGTTCTCATCCT

>Ilv2 (FG595)_*Gymnopilus chrysopellus*

GCTCAATTTTTCCGATGGAGACACCCTCGATCAATGGTAACTTCTGGCGGTCTAGGGGT
ACGTCCTTGCTCCAGATCTTCTTCATGTTCCTTGCTGATTTTCCTCGATTAGACCATGGG
CTTCGGTCTGCCATCTGCTATCGGAGCCAAGGTTGCTGCTCCTCAGAAGACTGTTGTCG
ACATCGATGGTGATGCATCATTTAGCATGACTGCGATGGAACTTGCTACTGCGTCACAA
TACAATATCGGTGTCAAAGTCATCGTCTT

>Ilv2 (FG595)_*Agrocybe pediades*
GCTCAGCACTTCAGGTGGAAGCACCCCAGATCGATGGTTACATCGGGTGGTCTTGGTG
TATGTTTCTTTTCTATTCTATTTCTTTTTCGTTTTTCTCTCTTTTCTCTGTAGACTATGGGC
TTCGGTCTACCTGCAGCTATTGGTGCCAAGGTCGCCGCACCTCAAAAGATTGTCGTTG
ACATTGACGGTGACGCCTCCTTCAGCATGACCGCTATGGAGCTTGCCACCGCCTCTCAA
TACAACATCGGTGTCAAGGTCCTCGTTTT

>Ilv2 (FG595)_*Serpula lacrymans* var. *lacrymans*
GCACAGCATTTCAGATGGAAGTACCCCCGATCGATGGTGACTTCTGGTGGTCTGGGTGT
ATGTTTTACTACTCATCGCATTACCTGATCAGTTGACTGATACGTTTTGTAGACGATGGG
CTTCGGGCTTCCATCAGCTATTGGTGCCAAAGTCGCTGCTCCACAGAAGACTGTCGTT
GACATTGACGGCGATGCTTCGTTCAGTATGACCGCGATGGAACTTGCCACCGCTTCTCA
ATACAATATCGGTGTCAAGGTTCTTGTGTT

>Ilv2 (FG595)_*Mycena crocata*
GCGCAGCACTACCGGTGGACGCACCCACAGTCGATGGTCACCTCGGGTGGACTAGGG
GTCAGTTTTATTTTCTGAATGAAAACGGGTCATGACTGACGCGCGTGTACACAGACGAT
GGGCTTCGGACTGCCTGCTGCGATTGGCGCCAAAGTCGCCGCGCCGCACAAGACGGT
TGTGGACATTGATGGCGATGCGTCGTTCAGCATGACCGCGATGGAGCTCGCGACCGCC
GCACAGTACGGCATCGGCGTCAAGATCCTCGTTCT

>Ilv2 (FG595)_*Schizophyllum commune*
GCGCAGCACTTCAGGTGGACGAACCCGCAGCAGATGGTCACTTCCGGCGGGCTGGGC
ACGATGGGCTTCGGCTTGCCCGCGTCGATTGGCGCGAAGGTCGCGGCGCCGGAGAAG
ACGGTTATTGATATCGACGGCGACGCGTCGTTCAGCATGACGGCGATGGAGCTCGCCA
CCGCGTCGCAGTTTGGCATCGGCGTCAAGGTTCTCGTGCT

>Ilv2 (FG595)_*Auricularia delicata*
GCGCAGTTCTTCCGCTGGAGACATCCGCACACGATGGTCACGTCTGGCGGTGCTGGGA
CCATGGGCTTTGGCCTGCCTTCGGCGATTGGCGCTAAGGTGGCCGCACCGGAGAAGAC
TGTTGTCGACATCGACGGCGATGCCTCGTTCAGCATGACCGCAATGGAGCTTGCGACC
GCGTCGCAGTACAACATCGGCGTCAAGGTGCTCGTGCT

>Ilv2 (FG595)_*Calocera cornea*
GCACAGTTCTTCCGTTGGCGGCATCCGCGAACTATGATCACATCCGGGGGCCTTGGCGT
AAGTGTTCTGTTCTTCTTCTGGCCCCCCGGCTGACCGTGCCGTGTTTTCCAGACCATGG
GCTTTGGCCTGCCCTCTGCCATTGGATGCAAGGTCGCCGCACCGGAGAAGATTGTCGT
CGACATCGACGGTGATGCCTCATTCAGCATGACGGCAATGGAGCTCGCCACCGCCGCA

CAATACAGCATTGGCGTGAAGGTGCTGGTGCT

>Ilv2 (FG595)_*Phanerochaete carnosa*
GCGCAGCACTACCGGTGGAGGTATCCACGCACGATGGTCACTTCCGGTGGCTTGGGTG
TAAGTCTATTCGCGGCGCTTTTACCCATGAATGTGCTGATTCTATGTTTGCAGACCATGG
GCTTCGGCCTGCCAGCTGCTATCGGCGCTAAAGTCGGCGCGCCAGAGAAGACCGTCGT
TGATATCGATGGTGATGCTTCGTTCAGCATGACTGCGATGGAGCTTCAGACTGCATCGC
AGCACAACATCGGCGTTAAGGTCATCGTCCT

>Ilv2 (FG595)_*Fomitopsis pinicola*
GCGCAACATTACCGGTGGCGGCAGCCGCGGAGCATAGTCACCTCCGGTGGTCTTGGGG
TCAGTGTCTGCGTTCCATTCCGACCTGCATGGTTGACTGATACCGTTATGCAGACGATG
GGCTTCGGTCTTCCCGCAGCCATCGGTGCCAAGGTTGGCGCGCCGAACAAGATCGTCG
TGGACATCGACGGTGATGCGTCGTTCAGCATGACTGCGATGGAGCTGCAGACCGCATC
ACAGTACAACATCGGTGTCAAGGTCATGGTGCT

>Ilv2 (FG595)_*Trametes versicolor*
GCGCAGCACTTCCGGTGGAGGCACCCGCGCTCGATGGTGACGTCGGGTGGTCTTGGT
GTAAGTTTGACACGTTGTATTCCTTGCTGTGCTAACGCTTTTCGCAGACGATGGGCTTC
GGTCTTCCGTCGGCCGTTGGCGCGAAGGTCGGCGCGCCTCACAAGACCGTCGTTGACA
TCGACGGCGACGCGTCGTTCAGCATGACCGCCATGGAGCTGCAGACCGCATCGCAGTT
CAGCATCGGTGTCAAGGTCTTGGTACT

>Ilv2 (FG595)_*Lactarius quietus*
GCGCAACACTTCCGATGGCGGCATCCGCGCACGATGATCACTTCGGGAGGGCTTGGTG
TACGTTTTGTCAATGATCCCCATAGCTCCCGCCTTGACCGTTCGATTAGACAATGGGCTT
TGGTCTCCCATCAGCGGTAGGCGCAAAAGTCGCTGCACCAAACAAGACTGTCGTCGA
CATCGATGGTGATGCTTCATTTAGCATGACTGCCATGGAACTTGCAACTGCTTCCCAATT
TGGCATCGGCGTTAAGGTGCTGATTTT

>Ilv2 (FG595)_*Tricholoma matsutake*
GCGCAACACTTCAGATGGCGGTATCCCGGGTCGATGGTCACTTCTGGTGGACTGGGGG
TATGCATTCGCGTTCAGTTTGTTTGTTGCCAGTCTTTTTTCTCTCAAAAAAAGACAATG
GGCTTCGGACTGCCGGCGGCTATTGGCGCCAAGGTGGCTGCGCCGCAGAAGACCGTC
ATTGACATCGACGGCGATGCCTCATTTAGCATGACCGCAATGGAGCTCGCCACTGCCTC
TCAATTCAACATTGGCGTCAAGGTCCTCATTCT

>Ilv2 (FG595)_*Dacryopinax primogenitus*
GCTCAGTTCTACCGCTGGCGGCATCCTCGGACCATGATCACCTCGGGCGGACTGGGTG
TAAGTAGTTTCCTGGTTATGTAGCTTACTAGGCCTAACAGACTATGGGCTTCGGCTTACC
TGCTGCTATAGGTTGCAAAGTTGCTGCTCCGGAGAAAGTCGTCGTCGACATTGACGGA
GATGCCTCTTTCAGCATGACTGCGATGGAATTGGCCACTGCTGCGCAATACAACATCGG
TGTTAAGGTCCTTGTGCT

>Ilv2 (FG595)_*Laccaria bicolor*
GCCCAATTTTTCCGCTGGAAACACCCTAGAACAATGGTAACGTCTGGCGGTTTGGGGG
TATGTGGTTTAGTCTGACGCCTATCATCGCGTAATCGCCCTTCCCTTTTCTAGACGATGG
GCTTTGGTTTGCCAGCATCTATTGGTGCTAAAGTGGCCGCGCCACATAAGACCGTCATC
GATATTGACGGCGACGCCTCATTTAGCATGACTGCTATGGAGCTCGCAACTGCCTCACA
GTATGGCATTGGTGTCAAAGTTCTTATCCT

>Ilv2 (FG595)_*Boletus edulis*
GCTCAGTATTATAGATGGAGAACACCACGGTCAATGGTTACCTCCGGTGGGCTCGGCGT
ACGTATCATCTTCTGTCTCCACGCTTTCGATCGCTGAGTTTGGTCTAGACGATGGGCTTT
GGTCTGCCCTCGGCCATCGGTGCCAAAGTCGCTGCACCCAACAAAACTGTGGTTGATA
TCGATGGCGATGCTTCGTTCAGCATGACTGCAATGGAACTTGCCACTGCTTCCCAGTAT
GACATCGGCGTGAAAGTCCTCATAAT

>Ilv2 (FG595)_*Hygrophoropsis aurantiaca*
GCTCAGCACTTTAGATGGAAAACGCCCCGTTCGATGGTGACATCCGGCGGCTTGGGGG
TAAGATTCCATTGGCTATGCCTGTGAAATATTGACTCGCCCTTTTCATCATAGACAATGG
GTTTCGGTCTTCCTTCAGCTATTGGAGCTAAAGTCGCTGCTCCTAACAAAACTGTCATC
GATATCGACGGCGATGCCTCATTTAGCATGACGGCTATGGAGCTTGCGACAGCGTCGCA
ATACGACATTGGCGTCAAAGTCCTCGTTCT

>Ilv2 (FG595)_*Gymnopus androsaceus*
GCTCAGTTCTATCGCTGGACACATCCAAGACAGATGGTCACTTCTGGGGGTCTTGGTGT
AAGCTCTTTCCTTTCCGTATTTATACATTGAGCTCATTTATGCAGACCATGGGTTTCGGT
CTACCTGCCTCCATTGGTGCCAAGGTCGCATCACCTCAAAAAATCGTTGTCGACATTGA
TGGCGACGCTTCATTCAGTATGACAGCTATGGAACTGCAGACAGCATCACAATACAACA
TTGGTGTGAAGGTTCTTGTGCT

>Ilv2 (FG595)_*Amanita muscaria*
GCGCAACATTTTAGGTGGAGATATCCCAGGTCAATGGTTACTAGTGGTGGTCTTGGGGT
AAGTGCGAGCGTTTTGTCTTCTGTCCTTTATTAACATTTGGGCGCACAGACAATGGGAT
TCGGCCTCCCAGCTGCAATAGGCGCCAAAGTTGCCGCACCCGAAAAAATTGTCGTAGA
CGTTGACGGTGACGCGTCATTTAGCATGACGGCTATGGAGCTCGCTACGGCTTCTCAGT
TTGGGATCGGAGTCAAAGTTTTGGTGTT

>Ilv2 (FG595)_*Pluteus cervinus*
GCTCAGTTTTTTAGGTGGACGAGGCCGAGGTCGATGGTTACTAGTGGTGGATTGGGGG
TCAGTGTCTCTTTCTCTCTCGTTTTTTGGGCTAATGCGTTGATGGTTCTTTAGACAATGG
GTTTCGGTTTACCAGCAGCTATTGGTGCTAAAGTAGCTGCACCGGAGAAGATTGTGGTT
GATATCGACGGTGATGCTTCGTTTAGCATGACTGCGATGGAACTTGCTACTGCTTCGCA
GTATAACATCGGCGTTAAAGTTCTTGTGAT
>Ilv2 (FG595)_*Stereum hirsutum*
GCTCAGCACTATCGGTGGACGGAGCCCAGGTCAATGGTCACGTCGGGTGGTTTGGGAG
TAAGTTCTTCTTCCTTCGTCTCGTCCCTCGTTTTTTTTCCGTTTTCTCTCTAGACCATGG

GCTTCGGTCTTCCGTCGGCTGTGGGCGCTAAGGTGGCAGCACCGAACAAGATCGTGGT
TGACATTGATGGTGATGCCTCGTTCAGCATGACTGCGATGGAGCTCGCCACCGCGTCG
CAATACAACATCGGTGTCAAGGTCATGGTTCT

>Ilv2 (FG595)_*Fomitiporia mediterranea*
GCACAGTTCTTCCGGTGGAGGACACCAAGATCCTTGGTGTCCTCCGGTGGTCTGGGCG
TAAGTTGAGAACACTTTGAAAAGATTTTCCATCTAAGTTGCTTCTGTTTTCAGACCATG
GGTTTCGGTCTTCCTGCGGGTATCGGTGCCAAAGTCGCAGCTCCCGAAAAAATCGTCG
TCGACATCGACGGCGACGCCTCCTTCAGCATGACAGCAATGGAGCTCCAAACCGCTTC
ACAATACAACATCGGTATCAAAGTCATCATCCT

>Ilv2 (FG595)_*Marasmius fiardii*
GCACAGTTTTATAGGTGGACGGAACCCAGACACTTGGTTACCAGTGGCGGATTGGGGG
TGAGTCGCACTTGCTTTTTGTTCCTTTTCCTGATTACCGATATCTTTTAGACTATGGGATA
CGGTCTCCCTGCCGCTATCGGTGCCAAAGTCGCTCTTCCTCACAAGACGGTTGTTGACA
TTGACGGTGACGCTAGTCTGAGTATGACAGCTATGGAGATGCAAACCGCGTCGCAGTT
TGGAATTGGAGTCAAAATTCTCGTTTT

>Ilv2 (FG595)_*Ramaria acris*
GCACAGTTTTATAGGTGGACTCATCCACGGACCATGATCTCCTCTGGTGGACTTGGGGT
AAGTCGCACTCTCTTGGCATTTTGTCTAACCTAACCTTCCCTCAAGACGATGGGCTTTG
GTTTACCAGCTGCCATTGGAGCCAAGGTTGGCGCTCCAGACAAGATTGTCGTCAACAT
TGACGGTGATGCATCTCTGAGTATGACTGCCATGGAACTTGCCACCGCTCACCAATACA
ATGTTGGCGTAAAATGTATGGTTTT

>Ilv2 (FG595)_*Gautieria morchelliformis*
GCACAGTTTTATCGATGGACTCATCCACGGACTATGATCTCTTCAGGTGGACTGGGGGT
GTGTTATCGCCATTTTGATAATGTTCTGGCGATTGTGCTCATTCTCTGACAGACCATGGG
TTTCGGCCTCCCAGCTGCGATTGGATGCAAGGTCGGCGCACCTGACAAAATTGTTATAG
ATATTGATGGTGATGCATCCTTGAGTATGACTGCTATGGAATTGGCTACCGCAGCGCAAT
ACAAGGTTGGCGTGAAATGCTTGGTGTT

>Ilv2 (FG595)_*Asperigillus triticus*
GCTCAGCACTTCCGCTGGCGCCATCCCCGCACCATGATCACCTCCGGTGGTCTGGGAA
CCATGGGCTACGGTCTCCCCGCCGCCATCGGCGCCAAGGTCGCGCGCCCGGATGCCCT
GGTTATCGACATCGACGGCGACGCGTCCTTCAACATGACCCTGACCGAGCTGTCGACG
GCGGCCCAGTTCAACATCGGCGTCAAGGTGCTGCTGCT

>Ilv2 (FG595)_*Neurospora crassa*
GCTCAGCACTTCCGCTGGCGCCACCCCCGTACCATGATCACCTCCGGTGGTCTCGGTAC
CATGGGCTTCGGTCTCCCCGCCGCCATCGGCGCCAAGGTTGCCAAGCCCGATGCTCTT
GTCATTGACATCGACGGCGACGCCTCCTTCGGCATGACCCTCACCGAGCTCTCCACCG
CTGCTCAGTTCAACATTGGCGTCAAGGTCATCGTCCT

>Ilv2 (FG595)_*Agaricostilbum hyphaenes*
GCCCAGCATTACAGATGGACATCACCTCGCTCGTGGGTGTCTTCCGGTGGTCTTGGCGT
AAGCACAATTGAATGATCCGTGAACTCCACTGATGTTTCCTCATGCTTCCAGACTATGG
GTTACGGGTTGCCATCATGCATTGGCGCCAAGGTGGCTGCACCCGAGAAATACGTCGT
CGACATTGACGGTGATGCTTCATTCAGCATGACGGCTATGGAGATGGCTACTGCTTCGC
AGTATGGCATCGGTGTTAAGCTGCTCGTCTT

>Ilv2 (FG595)_*Rhizopus microsporus*
TGTCAGTACTACCGTTGGACACATCCTCGTACTTTCATTTCCTCAGGCGGTTTAGGAAC
CATGGGTTATGGCCTTCCTTCTGCTATTGGTGCCAAGGTTGCAAAGCCCGACCATATCG
TCGTGGACATTGATGGTGATGCCTCCTTTTCGATGACTGCCATGGAACTTGCCACCGCT
GCTGAATTCAACATTGGTGTCAAGGTCTTGTTACT

>Krr1 (FG695)_*Pluteus cervinus*
TTGCTGGCTCGAGGCGTCGCTATTAACCAAGCCGTCAAAATCCTGGATGACACCATGG
CTTGTGACATCATCAAAATCGGAAATTTGGTTAGCAATAAGGAGCGGTTCGTAAAGCG
GAGACAACGTATCATAGGACCAGATGGAAGTACACTCAAAGTGCGTGCAGATACTCTC
TTTACTCCCTTTTCTCACTCAATTGCAGGCAATCGAATTGCTCACACAATGTTATGTCCT
TGTACAAGGAAGTACCGTCAGCGTTATGGGTCCCTACAAATCCTTGAAAGAGGTTCGA
AGAATTGTATTGGACTGC

>Krr1 (FG695)_*Gautieria morchelliformis*
CTTCTAGCCAGAGGTGTTGCTGTTGCCCAAGCGGTCAAGATTTTAGATGATGGTGTTGC
TTGCGACATAATAAAAATCGGAAACTTGGTACGGAACAAGGAGAGATTTGTGAAACGA
CGGCAGAGGATCATAGGGCCTGATGGGAGCACACTGAAGGTGAGGGCCACGAAGCCT
CAAACCTTCGTACTTATCACTGTGATAGGCAATCGAATTGTTAACTTCGTGCTACGTGCT
TGTGCAAGGCAGTACAGTAAGCGTCATGGGGCCTTACAAATCCCTAAAAGAAGTACGG
CGAATAGTACTAGATTGC

>Krr1 (FG695)_*Fomitopsis pinicola*
TTGCTTGCCCGCGGCGTAGCGATGGGGCAAGCTGCTAAGATCCTGGATGACGCCGTTG
CATGCGACATCATAAAAATCGGCAACATTGTACGGAATAAGGAGCGTTTTGTGAAGCG
GAGACAGAGGATCATTGGGCCAGACGGTAGCACGCTCAAAGTGAGTCTCATACTTGGT
CATGGGGCCTATCTGACATGTGGTGCAGGCCATCGAGTTGTTGACGCAGTGCTACGTAC
TCGTTCAAGGAAGCACCGTTAGCGTAATGGGACCATATAGGGGCCTGAAGGAAGTCCG
CCGGATAGTCCTCGACTGC

>Krr1 (FG695)_*Schizophyllum commune*
CTACTAGCTCGTGGTGTCGCCGTCGCTCAAGCAGTCAAGATCCTCGACGACAACATGG
CATGCGACATCATCAAGATCGGCAGCTTGGTACGGAATAAGGAGCGCTTTGTGAAGCG
GCGGCAGAGGATTATCGGGCCGGATGGGAGTACGCTGAAGGTGCGTGGTGAACTTTCT
TGACTTCCTGGAGTTTAACGCGCTGCAGGCAATCGAGTTGTTGACCCAGTGCTACGTC
CTCGTGCAAGGTAGCACCGTCAGTGTCATGGGTCCTTACAAGTCCTTGAAGGAGGTCC
GCCGCATCGTCCTCGACTGC

>Krr1 (FG695)_*Tricholoma matsutake*
CTGTTGGCAAGAGGTGTTGCCATTACCCAAGCAGTGAAGATACTAGACGATAACATGG
CGTGCGACATCATCAAGATCGGAAACCTCGTGCGCAATAAAGAACGGTTTGTGAAGAG
GCGACAGAGGATCATTGGTCCGGATGGGAGCACTCTCAAGGTACGTTTTCTACGCAAT
CGTTCCTCCTAATTTGTTTGTGGGACAGGCTATTGAACTGTTGACGCAATGTTATGTGCT
GGTGCAGGGGAACACTGTCAGTGTCATGGGGCCATACAAGTCCCTGAAGGAAGTCCG
TCGGATTGTACTAGATTGC

>Krr1 (FG695)_*Gymnopilus chrysopellus*
CTCCTCGCACGCGGCGTCGCGGTGAACCAGGCCGTGAAGATCCTCCAGGACGATGTG
GCGTGCGATATCATCAAGATTGGGAATCTGGTGCGGAATAAGGAGAGGTTTGTGAAGA
GGAGACAGAGGATTATCGGCCCGGATGGGAGTACGTTGAAGGTGTGTGTGTGTTCTTA
TGGAGGGGTGGTGACCTTTTATTTTCAAGGCTATTGAGCTGTTGACGAACTGTTATGTC
CTCGTGCAAGGGAACACCGTCGCTGCTATGGGCCCATACAAGTCCTTAAAAGAAGTAC
GCCGAATAGTCCTCGACTGC

>Krr1 (FG695)_*Agaricus bisporus* var. *burnettii*
CTTCTTGCTCGCGGTGTGGCCGTTAATCAGGCCGTTAAAATCCTTGAGGACGACATGGC
GTGTGATATCATAAAAATTGGAAATTTGGTACGAAATAAAGAAAGGTTTGTGAAGCGG
CGGCAAAGGATCATTGGACCAGACGGCAGTACACTGAAGGTTCGCCTGGCCCCTAACC
CCCTGTCGAAGCTATCTCCGTTCATAGGCAATTGAGCTTCTAACTCAGTGCTATGTGCT
GGTGCAAGGAAGCACTGTTAGTGCCATGGGACCCTACAAATCTCTGAAAGAAGTCAG
GCGGATCGTCCTCGATTGC

>Krr1 (FG695)_*Trametes versicolor*
CTGCTTGCACGAGGTGTCGCGATCGGGCAGGCGGTCAAAATCATGAGCGACGACATTG
CGTGTGACATCATCAAGATTGGGAATGTTGTGCGGAATAAGGAGCGGTTCATCAAGCG
GCGGCAGAGAATCATTGGGCCGGACGGCAGCACGCTGAAGGTACGCTTTACGGATAAT
GCACGCAGATAGATTAACAATATATCAGGCTATCGAATTGCTCACGCAGTGTTACGTCCT
TGTACAAGGAAACACTGTCAGCGTCATGGGTCCGTACAAGGGGCTTAAGGAGGTACG
GCGGATCGTGCTCGACTGC

>Krr1 (FG695)_*Dacryopinax primogenitus*
CTCCTCGCGCGTGGAGTTGCGGTCGGGCAGGCGGTGAAGATCCTGGACGACGCTGTG
GCGTGCGATATTATCAAGATTGGGAATATCGTTAGGAATAAGGAGCGGTTCGTCAAGCG
GAGACAGAGGATCATTGGCCCGGATGGGAGTACGCTTAAGGTGCGTTTCGCGTCTGTT
AATCTTATTGGCTACAGGCCATCGAACTCTTAACACAATGTTACGTCCTCGTGCAAGGA
AACACCGTATCCGTGATGGGCCCGTACAAGTCGCTTAAGGAAGTCCGACGTATCGTCAT
CGACTGT

>Krr1 (FG695)_*Agrocybe pediades*
CTCATGGCCCGTGGTGTAGCAGTGAACCAAGCCGTTAAGGTCTTGCAAGACGATATTG
CTTGTGACATTATCAAAATCGGCAACCTGGTGCGGAATAAGGAGAGATTTGTGAAGAG

ACGGCAGAGGATTATCGGACCTGATGGAAGTACACTGAAGGTGAGTCTGAGTTTTCTC
TGAGAGGTGAGAATGAATGTGTATACAGGCTATCGAACTCCTCACAAATTGCTACGTCC
TCGTACAAGGAAACACAGTCAGCGTAATGGGTCCTTTCAAATCACTAAAAGAGGTCCG
ACGCATAGTCCTCGATTGC

>Krr1 (FG695)_*Coprinopsis cinerea*
CTCATGGCCCGTGGTGTTGCTCTGAACCAGGCGGTGAAAATCCTTCAAGACGACATGG
CCTGCGATATTATCAAGATTGGGAACCTCGTACGGAATAAGGAGCGCTTTGTTAAGCGG
AGACAACGTATCATTGGACCTGACGGGAGCACGCTGAAGGTGTGTTTGTTGGCCACAA
ATATGTTGTATTCTAGGCCATCGAGCTGCTCACCAACTGCTACGTCCTGGTCCAGGGAA
GCACAGTGAGCGTGATGGGTCCCTTTAAATCTCTAAAAGAAGTGCGGAGAATAGTTCT
CGATTGC

>Krr1 (FG695)_*Laccaria bicolor*
CTTTTGGCGCGTGGTGTGGCTGTCTCGCAAGCGGTAAAGATTTTGGACGACGCGGTTG
CGTGTGATATCATCAAGATTGGGACGTTGGTGCGGAACAAGGAGAGGTTTGTGAAGCG
GAGGCAGAGGATTATTGGACCGGATGGGAGTACGCTCAAGGTGAGTTGGGCTTCTTCT
GAAGTAACAGAAACCTAATGGAGAGTAGGCCATTGAACTATTGACGAATTGTTATGTGC
TGGTGCAAGGAAGCACAGTGAGCGTGATGGGTCCTTACAAGTCGCTCAAGGAGGTTC
GACGAATAGTTCTCGATTGT

>Krr1 (FG695)_*Amanita muscaria*
CTGCTCGCACGAGGTGTAGCTATCAACCAAGCAGTCAAGATATTAGAAGACAACATTG
CGTGCGATATCATCAAAATCGGGAGCCTGGTACGGAACAGGGAGAGATATGTCAAGCG
CAGGCAGAGGATCATCGGACCTGATGGAAGCACGCTCAAGGTGAAGCCGAAAACCGA
AACCCGATTGTCGTTGATCGGAATGTCAGGCCATTGAATTGTTAACGGGATGCTATGTG
CTGGTGCAGGGCAGCACTGTAAGTGTGATGGGACCGTACAAGGCGCTCAAAGAAGTT
CGACGAATAGTCCTCGATTGC

>Krr1 (FG695)_*Gymnopus androsaceus*
CTCCTTGCGCGAGGTGTTGCAATGACACAGGCCGTAAAGATCCTCAACGATGAGATTT
CGTGTGATATTATCAAGATTGGGAATCTAGTAAGGAACAAGGAACGGTTTGTGAAGAG
ACGACAGAGGATTATTGGACCGGACGGAAGTACGTTGAAGGTGTGTGTGGTGTGTGAT
TTCTTCCTGTTGATCGACGTGTACGCAGGCTATTGAGTTGTTAACGCAATGTTATGTGCT
TGTACAAGGAAGCACGGTCAGCGTTATGGGACCTCATAAATCCCTCAAGGAAGTTCGC
CGGATAATTCTCGATTGC

>Krr1 (FG695)_*Phanerochaete carnosa*
TTACTTGCTCGTGGCGTCAACATCGGTCAGGCCGTGAAGATACTTGATGACGCTATTGC
CTGCGACATTATCAAGATCGGGAACATTGTTCGGAATAAGGAACGATTTGTGAAACGG
CGGCAGAGGATAATTGGTCCGGACGGCAGCACGCTGAAGGTACGTTCACTTGAGTGTA
GCCGTGGTCAGGTTTCATGTACGCCAGGCTATCGAGCTCTTGACCCAATGCTACGTCTT
GGTGCAAGGAAGCACTGTCAGCGTAATGGGCCCATATAAGGGCTTGAAGGAAGTCCG
GAGAATTGTGCTTGATTGC

>Krr1 (FG695)_*Ramaria acris*
CTTTTAGCTAGAGGTGTGGCTATTGGACAAGCTGTCAAGATCTTAGATGACAGTGTCGC
GTGCGACATCATCAAAATTGGGAATTTAGTACGAAACAAAGAGAGATATGTAAAGAGG
AGACAAAGGATCATAGGACCTGACGGGAGTACATTGAAGGTGAGTAACTCGGTGTTGG
GATCCTCGGGGCTTAATCCTTGTTAAGGCGATCGAACTGTTAACTTCGTGCTACGTACT
GGTTCAGGGCAGCACTGTGAGCGTCATGGGCCCCTACAAGTCTCTCAAGGAAGTACG
AAAAATTGTGTTAGATTGC

>Krr1 (FG695)_*Lactarius quietus*
CTTCTCGCGCGAGGTGTCGCAGTCGGACAGGCCGTCAAAATCTTGGATGATGGCGTTG
CATGCGACATCATCAAGATTGGAAATCTTGTTCGGAACAAGGAGCGTCTCGCAAAACG
GAGACAGCGCATCATCGGACCAGATGGCAGTACCCTCAAGGTTTGTTACTCTAGTCTAC
ACCTCGGAATGACTCGATCACCACTAGGCTATCGAGCTGCTCACTCAGTGCTACGTCTT
GGTGCAAGGGAGCACCGTTAGCGTCATGGGTCCCTACAAATCCCTCAAGGAAGTCCGT
CGAATTGTGCTAGACTGT

>Krr1 (FG695)_*Hygrophoropsis aurantiaca*
CTCATGGCACGAGGTGTTGCAGTTACGCAAGCTATGAAAGTTCTGCAAGACGATGTAG
CATGCGATATCATCAAGATTGGTAGCTTGGTTCGTAACAAAGAACGTTTCGTTAAACGG
AGACAAAGGATCATTGGACCAGATGGGAGCACTCTCAAGGTGGGCACGCCAAAGATT
TGCATGATGCTTATACCGGATACGCTAGGCGATTGAGCTTTTAACTCAATGTTATGTACT
TGTACAAGGCAGTACGGTTAGCGTTATGGGCCCATACAAATCCCTCAAAGAGGTCCGG
CGAATAGTAATAGACTGC

>Krr1 (FG695)_*Serpula lacrymans* var. *lacrymans*
CTATTGGCGAGAGGTGTAGCAATATCACAAGCAGTTAAGGTTCTCCAGGACGACGTTG
CATGTGATATAATCAAAATTGGCAGTCTTGTACGAAACAAAGAGCGGTTCGTGAAGCG
AAGACAGAGAATCATCGGACCAGATGGGAGTACTCTCAAGGTATGTTTTGTATCAGATT
TTTGAAGTGTTGTATTCGTTCTCCTAGGCCATCGAGCTCTTGACCCAGTGTTATGTATTG
GTCCAAGGAAGCACTGTAAGCGTCATGGGGCCTTACAAGTCACTAAAGGAAGTCAGG
AGAATAGTTTTGGACTGC

>Krr1 (FG695)_*Marasmius fiardii*
CTCCTCGCTCGTGGTGTTGCAGTTCAGCAGGCAGTCAAGATTCTTGAAGACAACATCT
CTTGTGACATTATCAAAATTGGCAACCTGGTTCGGAACAAGGAACGATTTGTGAAGCG
AAGGCAGCGTATAATAGGTCCGGATGGTAGTACACTGAAGGCACGTTTCATCAGCTCTT
GCCGATTTGTTCTACTGCTGTGTGTAGGCCATCGAACTGTTAACGCAGTGCTATGTCTT
GGTTCAGGGGAATACTGTCAGTGTAATGGGGCCTCACAAGTCACTGAAGGAGGTGCG
GCGTATAGTCTTGGATTGT

>Krr1 (FG695)_*Auricularia delicata*
CTGCTTGCGCGCGGCGTGGCGATAAATCAAGCGCTCCGGGTGCTTGAGGACGGGGTG
GCGTGCGACATTATCAAGATTGGTGGCATGGTGCGGAATAAAGAACGCTTCGTCAAGC

GGAGACAGCGTATCATTGGACCGGATGGGAGCACACTGAAGGTCCGTGGTCTTCTGAT
GATCGAAAGACTGACGCTTCGGTTGACAGGCTATCGAGTTGCTCACTGAGTGCTACGT
CCTTGTACAAGGAAACACTGTCAGCGTACTCGGTCCCCATAAGAAGCTCAAAGAGGTC
CGACGCATCGTCGAGGACTGC

>Krr1 (FG695)_*Calocera cornea*
CTGCTCGCGCGCGGGGTCGCGCTCGGGCAGGCGGTGAAGATCCTCGACGACGCGGTG
GCGTGCGATATTATCAAGATTGGGAATATTGTGCGGAATAAAGAGCGGTTCGTGAAGAG
GAGGCAGAGGATTATCGGCCCGGACGGGAGCACGCTCAAGGTGCGTCGTATTGGCCC
GTGGCATATGTCAATGGATTGATCGTCAGGCCATCGAGCTCCTCACAGGCTGCTACGTT
CTCGTGCAAGGCAACACGGTGTCCGTGATGGGCCCGTACAAGTCGCTCAAAGAGGTG
CGGCGCATCGTCCTGGACTGC

>Krr1 (FG695)_*Stereum hirsutum*
CTTCTCGCTCGTGGTGTGGCCGTGACTCAGGCCGTGAAGATTCTGGACGACAACGTTG
CATGCGACATCATCAAGATTGGAAACCTTGTGAGAAACAAAGAGAGATTTGTGAAGA
GGAGACAAAGAATTATTGGGCCGGATGGGAGTACGTTGAAGGTATGCACTGATTTACT
GTTCCGATCAGTATCTCATATCTTCACAGGCCATTGAATTGTTGACCCAGTGTTACGTTT
TGGTACAAGGAAGCACTGTTAGCGTCATGGGTCCTTACAAGTCTCTCAAGGAGGTTCG
GCGGATAGTCCTGGATTGT

>Krr1 (FG695)_*Fomitiporia mediterranea*
CTCCTCGCGCGTGGAGTCGCCCTTGGCCAAGCCGTGAAGATCCTCAACGACGACATGG
CTTGCGACATCATCAAAATTGGCGGTTTAGTCAGAAATAAGGAACGATTCGTTAAGAG
ACGGCAGAGGATTATTGGACCTGATGGGAGTACGTTGAAGGTACGTATTTTAGTTTTTG
TCCTGACGATTTTGTTGGTACATTTAGGCAATTGAACTTTTAACCCAGTGCTATGTCCTT
GTACAAGGCAACACAGTTAGCGCCATGGGCCCATACAAATCGCTCAAAGAAGTCCGTC
GAATCGTACTCGACTGC

>Krr1 (FG695)_*Boletus edulis*
CTCCTTGCTCGAGGCGTTGCAGTCACCCAAGCGGCCAGGATCCTCCAAGATGATATCG
CATGTGACATCATTAAAATCGGAAATCTTGTGCGGCACAAAGAGCGCTTCGTTAAGAG
GAGGCAGCGTATCATTGGTCCTGACGGTAGCACTCTCAAGGTGACTTTGGTGCCATCC
CTTCAAGGTCTCACAAAGGAAGGATTAGGCAATTGAGCTTTTGACGCAGTGCTATGTT
TTAGTCCAGGGCAATACGGTGAGCGTTTTGGGCCCGCACAAATCCCTGAAGGAAGTAC
GACGCATCGTTTTGGATTCC

>Krr1 (FG695)_*Mycena crocata*
CTACTTGCGCGAGGCGTTCCAGTGAGCCAGGCCGTCAAGATACTCGATGATTCTGTTG
CGTGCGACATCATAAAAATCGGCACCTTGGTGCGGAATAAAGAAAGATTCGTAAAGCG
GAGACAGAGGATAATTGGTCCTGATGGAAGTACTCTGAAGGTAATGTGTTGGTACTTCC
TTCGAGTAGCTGATTATGGCAGGCTATCGAACTATTAACGCAATGCTACGTCCTGGTGC
AAGGAAATACTGTCAGTGTTATGGGTCCGTATAAATCTCTTAAGGAGGTTAGACGGATA
GTTCTCGACTGC

>Krr1 (FG695)_*Rhizopus microsporus*
TTATTAGCAAGAAGTGTACCTTTTATATAGGCAATCAAAATTATGGAAGATGGCATTGCT
TGTGATATCATAAAGATTGGAAATATCACTCGTAACAAGGAAAGATTTGTAAAGCGTAG
ACAAAGATTGATTGGACCTAATGGTTCAACTTTAAAAGTAAAAAATAATCAAAATAAGG
TTATGATGATTGTGGATTTCTTAGGCAATTGAACTCTTGACAAAATGTTATATGATGGTT
CAAGGTAATACGGTTGCTGCTATGGGTCCATATAAGGGACTTAAGGATCTTCGTCGTATT
GTGATTGATTGT

>Krr1 (FG695)_*Neurospora crassa*
TTGCTCGCCCGTTCAGTACCAGCCCCACAAGCGATCAAAATTCTCGAAGATGGCATGG
CCTGCGACATCATCAAGATCCGCAGCATGGTACGGAACAAGGAGCGTTTTGTTAAGAG
GCGCCAAAGAATTCTCGGACAGAACGGCACAACGCTCAAGGCTCTCGAGCTTCTCAC
ACAAACATATATCCTCGTGCACGGAAACACAGTGTCGGTAATGGGTCCTTTCAAGGGT
CTGAAGGAGGTGAGGAGGGTTGTTGAGGACACC

>Krr1 (FG695)_*Asperigillus triticus*
TTGCTGTCGAGAAGTGTGCCTGTGTGACAGGCACTGAAAATCCTCGAGGACGACATTG
CATGCGACATCATTAAAATCCGCAACCAGGTGCGCAACAAAGAACGCTTCGTCAAGCG
ACGACAACGTATCCTCGGTCCCAACGGCTCGACGTTGAAGGCGCTCGAACTCCTGACG
AGCACGTACATCCTCGTGCAAGGCAACACCGTGGCAGCGATGGGACCGTTCAAGGGG
CTGAAGGAAGTGCGCAAGATCATCAACGACTGC

>Mcm2 (MS463)_*Gymnopus androsaceus*
AAGCGTATCATCAAGTCCATCGCTCCCTCCATCTACGGTCACGAAGATATCAAAACGGC
GATTGCGTTGTCTCTCTTTGGTGGTGTCTCCAAAGACATCAACCACAAGCATCGTATTC
GTGGTGATATTAATGTTCTCCTCCTCGGTGACCCCGGTACAGCCAAATCCCAATTCCTC
AAATATGTCGAAAAGACAGCCCATCGATCTGTCTTTGCTACCGGTCAAGGTGCCTCTGC
GGTTGGTTTGACAGCTAGTGTGCGCAAAGACCCGGTTACGCGTGAATGGACGCTGGA
GGGAGGCGCGTTGGTGCTCGCCGACAAGGGTACTTGTCTTATTGACGAATTCGACAAG
ATGAACGA

> Mcm2 (MS463)_*Fomitopsis pinicola*
AAGCGCATCATCAAGTCTATTGCCCCTTCGATCTACGGGCATGAAGACATCAAGACAGC
CCTCGCCCTGTCATTATTTGGTGGCGTGCCCAAGGACATCAACCGCAAGCACCGTATCC
GCGGAGACATTAACGTCCTCCTTCTCGGCGACCCGGGTACAGCAAAGTCGCAGTTCCT
GAAGTACGTGGAGAAAACAGCGCACCGGTCCGTGTTCGCGACCGGGCAGGGCGCGTC
CGCAGTCGGTCTGACGGCGAGCGTGCGCAAGGACCCGGTGACGCGCGAGTGGACGCT
CGAGGGCGGCGCGCTCGTGCTCGCGGACAAGGGGACGTGCCTGATCGACGAGTTCGA
CAAGATGAACGA

> Mcm2 (MS463)_*Phanerochaete carnosa*
AAACGCATCATCAAGTCCATTGCCCCTTCCATCTACGGACACGAGGACATCAAAACCG
CCATCGCGTTGTCTTTGTTCAGTGGTGTCTCCAAAAATATCAACCGGAAGCACCCTCTT

CGTGGTGATATTAACGTACTCTTGTTGGGAGATCCCGGTACTGCCAAGTCGCAATTCCT
CAAGTACGTCGAGAAAACAGCGCACCGTTCCGTCTTCGCCACTGGCCAGGGTGCTTCC
GCCGTCGGTCTTACCGCGAGTGTGCGCAGAGACCCTGTGACCTGCGAATGGACGCTCG
AGGGAGGTGCACTCGTTCTTGCAGACAAGGGCACCTGTCTGATCGATGAGTTTGACAA
AATGAACGA

> Mcm2 (MS463)_*Trametes versicolor*
AAACGCATCATCAAGTCTATCGCCCCCTCCATCTACGGCCATGAAGACATCAAGACAGC
CTTGGCCCTATCGCTTTTCTCCGGTGTTTCCAAGGATATTAACCGGAAGCATCGTATTCG
TGGTGATATCAACGTCCTTCTGCTCGGTGACCCAGGAACGGCCAAGTCCCAATTCTTG
AAGTATGTGGAGAAGACGGCCCATCGTTCTGTGTTCGCCACGGGTCAGGGCGCGTCTG
CCGTCGGTCTTACGGCGAGCGTGCGCAAGGATCCTATCACGCGCGAGTGGACTCTGGA
GGGTGGCGCACTCGTGCTCGCCGACAAGGGAACTTGTCTCATCGACGAGTTCGACAA
GATGAACGA

> Mcm2 (MS463)_*Stereum hirsutum*
AAACGCATCATCAAATCTATCGCGCCCTCCATCTACGGTCACGAAGACATCAAAACTGC
TCTCGCCCTCTCCCTTTTCGGTGGATGTTCGAAGGACATCAAGCGCAAGCACCGAATC
CGTGGCGACATCAACGTACTGCTGCTTGGTGATCCTGGAACGGCCAAATCTCAGTTCC
TGAAGTACGTTGAGAAGACGGCTCATCGGTCGGTGTTCGCTACGGGCCAAGGCGCATC
TGCGGTCGGTCTTACGGCCAGCGTTCGCAAGGATCCCGTTACTCGCGAATGGACTCTC
GAAGGTGGCGCGCTCGTTCTCGCCGACAAGGGCACCTGTCTCATCGACGAGTTTGACA
AGATGAACGA

> Mcm2 (MS463)_*Pluteus cervinus*
AAGCGAATCATCAAGTCCATTGCGCCCTCTATCTACGGCCACGAAGACATTAAAACTGC
CATTGCACTTTCTCTCTTCGGCGGTGTACCCAAGGATATCAAACACAAACATCGAATCC
GTGGTGATATCAACGTCCTGCTCTTGGGTGACCCAGGAACAGCAAAATCTCAATTCCT
GAAATACGTAGAAAAGACGGCACATCGGTCGGTCTTTGCGACGGGTCAAGGTGCATCT
GCTGTCGGTCTTACTGCGAGTGTCCGCAAAGATCCTATAACGCGAGAATGGACGCTCG
AGGGTGGTGCTTTGGTGTTGGCGGACAAGGGTATCTGCCTTATCGACGAGTTCGACAA
GATGAACGA

> Mcm2 (MS463)_*Laccaria bicolor*
AAAAGGATCATCAAATCCATCGCCCCATCCATCTACGGCCACGAAGATATCAAGACCGC
CATTGCCCTTTCACTCTTTGGTGGAGTGTCCAAGGACATCAATCACAAGCATCGCATTC
GTGGAGATATCAACGTCCTTCTTCTAGGAGACCCTGGAACAGCCAAATCTCAATTTCTC
AAATATGTTGAAAAGACTGCTCACAGATCGGTCTTCGCAACTGGGCAAGGTGCATCAG
CGGTCGGTCTCACTGCCAGCGTCCGCAAGGATCCCATCACGCGAGAGTGGACGCTAGA
GGGTGGCGCACTAGTTCTCGCAGATAAAGGCACCTGCCTTATCGACGAATTCGACAAG
ATGAACGA
> Mcm2 (MS463)_*Schizophyllum commune*
AAACGCATCATCAAGTCCATCGCACCGTCCATCTATGGCCATGAGGACATCAAGACCGC
CATTGCCTTGTCATTATTTGGTGGGGTACCCAAGGACCCGAACCACAAGCATCGTATTC

GTGGCGATATCAACGTGCTCCTTCTCGGCGACCCCGGTACCGCCAAATCGCAGTTCCTA
AAGTATGTCGAGAAGACCGCCCATCGTAGTGTCTTCGCCACTGGTCAGGGGGCCTCCG
CCGTCGGTCTCACCGCCAGCGTCCGCAAGGACCCCATCACGCGCGAGTGGACGCTCG
AAGGCGGCGCGCTCGTCCTCGCCGACAAGGGCACCTGCCTCATCGACGAGTTCGACA
AGATGAACGA

> Mcm2 (MS463)_*Coprinopsis cinerea*
AAACGAATTATCAAGTCTATTGCTCCTTCTATCTACGGCCACGAGGACATCAAGACCGC
CATCGCTCTCTCCTTGTTCGGAGGTGTACCCAAGGATATCAACCATAAGCACCGTATCC
GTGGAGACATCAACGTACTTTTACTCGGTGACCCTGGTACGGCCAAATCCCAATTTCTC
AAATATGTTGAGAAAACCGCCCACCGATCCGTATTTGCGACGGGCCAGGGAGCTTCCG
CAGTCGGTCTCACAGCCAGTGTGCGCAAGGACCCAGTGACCCGAGAGTGGACGTTGG
AAGGAGGAGCATTGGTACTAGCCGATAAAGGAACCTGCCTCATCGACGAATTCGATAA
GATGAACGA

> Mcm2 (MS463)_*Agrocybe pediades*
AAGAGGATTATCAAGTCTATCGCACCGTCCATTTACGGCCACGAGGATATCAAAACTGC
TATTGCTCTCTCTCTGTTTGGAGGTGTACCTAAAGACATCAACCATAAGCATCGTATTCG
AGGCGACATCAACGTCCTTCTACTCGGAGATCCCGGAACAGCGAAGTCGCAATTCCTG
AAATATGTTGAAAAGACAGCCCATAGATCAGTCTTCGCCACTGGTCAAGGTGCTTCCG
CTGTCGGTTTGACTGCGAGCGTGCGGAAAGACCCTATAACTCAGGAATGGACATTGGA
AGGAGGTGCCTTGGTACTTGCGGATAAAGGGACATGCTTAATCGATGAGTTCGACAAG
ATGAACGA

> Mcm2 (MS463)_*Gymnopilus chrysopellus*
AAAAGGATCATCAAGTCGATCGCTCCATCCATTTATGGCCATGAAGATATCAAGACTGC
CATCGCCCTATCTCTCTTTGGTGGTGTACCCAAAGATATTGACCACAAACTACGGATTC
GAGGCGATATCAATGTCTTACTTCTCGGTGACCCCGGTACAGCCAAATCACAATTCCTG
AAATACGTTGAGAAGACTGCCCACCGGTCTGTCTTCGCCACGGGTCAAGGAGCCTCCG
CTGTAGGTCTTACAGCCAGCGTGCGCAAGGACCCTGTCACTCGAGAATGGACGCTCGA
AGGAGGTGCTCTGGTGCTCGCAGACAAGGGCCACTGCCTCATCGACGAGTTCGACAA
GATGAACGA

> Mcm2 (MS463)_*Lactarius quietus*
AAGCGTATCATCAAGTCCATCGCGCCTTCGATCTACGGGCACGAGGACATTAAGACCG
CCATCGCGCTCTCTCTCTTCGGCGGCGTCTCGAAGGACGTGAAGCGTAAGCTGCGCAT
CCGTGGCGACATTAACGTGCTACTACTTGGCGACCCCGGAACCGCCAAATCGCAGTTC
CTCAAGTACGTCGAGAAGACCTCGCATCGGTCGGTCTTCGCGACGGGCCAGGGCGCG
TCGGCGGTCGGTCTGACGGCAAGCGTGCGCAAGGACCCTGTGACGCGCGAGTGGACG
CTCGAGGGCGGCGCGTTGGTGCTTGCTGATAAGGGCCACTGTTTGATAGACGAATTCG
ACAAGATGAACGA
> Mcm2 (MS463)_*Auricularia delicata*
AAGCGCATCGTCAAGAGTATCGCGCCTAGCATCTACGGGCACGAGAATATCAAGACCG
CGCTCGCTCTGTCGCTCTTTGGCGGTGTTCCGAAGAACGTCAACAACAAGATGAACAT

CCGTGGCGACATCAACGTTCTCCTGCTCGGCGACCCTGGCACGGCCAAGTCGCAGTTC
CTCAAGTACGTTGAGAAAACCGCGCACAGAGCCGTCTTCGCGACAGGCCAGGGCGCG
AGCGCCGTCGGTCTGACGGCGAGCGTGCGGAAGGACCCCGTGACGCGCGAGTGGAC
GCTGGAGGGCGGCGCGCTGGTGCTCGCGGACAAGGGCGTGTGTCTGATCGACGAGTT
CGACAAGATGAACGA

> Mcm2 (MS463)_*Agaricus bisporus* var. *burnettii*
AAGCGCATCATCAAATCCATCGCTCCGTCTATCTATGGACATGATGATATCAAGACCGCT
ATAGCTTTGTCCCTCTTTGGTGGCGTGTCGAAGGATATCAATCACAAACATCGCATTCG
TGGAGATATCAATGTCCTTCTCCTAGGCGATCCCGGCACTGCGAAATCCCAATTCTTAA
AATACGTGGAAAAGACTGCTCATCGATCTGTTTTCGCAACGGGCCAAGGTGCTTCGGC
TGTGGGTTTGACTGCTAGCGTTCGCAAAGACCCAGTCACACGTGAATGGACTCTCGAA
GGTGGAGCTTTGGTATTGGCTGATAAAGGAACCTGCTTAATCGACGAGTTTGACAAAA
TGAATGA

> Mcm2 (MS463)_*Fomitiporia mediterranea*
AAGCGTATTATCAAGTCCATCGCGCCATCGATTTATGGACATGAAGATATCAAGACAGC
GATAGCACTGTCACTCTTTGGTGGTGTGTCGAAGGATATCAACCGCAAGCATCGTATTC
GTGGGGATATCAACGTGCTTATGCTCGGTGACCCAGGTACTGCGAAGTCGCAGTTCCT
GAAGTACGTTGAGAAAACTGCACATCGTGCTGTTTTCACGACAGGTCAAGGTGCTTCT
GCAGTCGGTTTGACTGCTAGCGTGCGCAAGGACCCAGTAACGCGGGAGTGGACTCTT
GAAGGGGGCGCCCTTGTTCTTGCAGATAAGGGAACTTGTCTCATCGATGAGTTCGACA
AGATGAACGA

> Mcm2 (MS463)_*Tricholoma matsutake*
AAACGCATCATTAAATCCATTGCGCCCTCCATCTACGGCCACGAAGGCATCAAAACAG
CGATTGCACTTTCTCTATTTGGCGGCGTTCCAAAAGATATCAACCACAAGCATCGCATC
CGGGGCGACATCAACGTTCTCCTCCTCGGCGACCCAGGAACGGCCAAATCTCAATTCC
TCAAGTACGTCGAAAAGACCGCCCACCGATCTGTCTTCGCAACTGGCCAAGGTGCTTC
TGCTGTAGGTCTGACTGCAAGTGTTCGCAAAGACCCTATCACACGCGAGTGGACACTA
GAAGGAGGGGCCCTTGTTCTCGCAGACAAGGGAACTTGCCTTATCGATGAATTTGACA
AGATGAACGA

> Mcm2 (MS463)_*Hygrophoropsis aurantiaca*
AAACGCATCATCAAATCCATCGCGCCGTCTATATACGGCCACGAAGACATCAAAACTGC
CATCGCGCTCTCGCTCTTCGGTGGCGTTCCCAAGGACATCAACGGCAAACACAGAATT
CGTGGAGATATCAACGTCCTTCTCTTGGGCGATCCCGGAACAGCCAAGTCGCAATTTTT
GAAATATGCAGAAAAGACAGCCCACCGGTCGGTATTTGCTACAGGCCAGGGAGCCTCC
GCTGTGGGTCTCACTGCTAGCGTCCGTAAGGACCCTGTTACAAGGGAGTGGACACTCG
AAGGAGGCGCACTAGTCCTTGCTGACAAGGGGACGTGCCTGATAGACGAATTCGACA
AAATGAACGA
> Mcm2 (MS463)_*Serpula lacrymans* var. *lacrymans*
AAACGGATAATTAAATCCATAGCACCTTCTATCTACGGTCATGAAGATATCAAAACCGCT
ATCGCACTCTCTCTGTTCGGAGGAGTTCCCAAGGATGTTAACAGGAAACACAGAATTC

GTGGCGACATCAACGTCCTCTTACTCGGTGACCCTGGAACAGCGAAGTCTCAGTTCTT
GAAATATGCAGAAAAAACGGCACATCGCTCCGTTTTCGCCACAGGTCAGGGCGCATCT
GCTGTTGGTCTGACTGCCAGTGTACGAAAAGATCCGATAACAAGAGAATGGACGCTCG
AAGGCGGGGCATTGGTTTTGGCTGACAAAGGGACATGTCTAATTGATGAGTTCGACAA
GATGAATGA

> Mcm2 (MS463)_*Marasmius fiardii*
AAACGGATCATCAAGTCGATCGCACCATCCATATACGGACACGAAGACATAAAGACCG
CCATTGCGCTCTCACTCTTTGGAGGGGTGGCTAAAGATATCAATCACAAACACCGCATT
CGTGGCGATATAAATGTTCTTCTTCTCGGCGACCCGGGTACAGCCAAATCGCAGTTCCT
CAAGTATGTTGAGAAAACAGCACACCGATCGGTGTTCGCTACGGGTCAAGGTGCATCC
GCGGTCGGTCTGACGGCCAGTGTTCGCAAAGATCCTATAACCAGGGAGTGGACACTGG
AAGGAGGAGCCTTGGTTCTTGCCGACAAGGGAACCTGTCTCATTGACGAGTTTGACA
AAATGAACGA

> Mcm2 (MS463)_*Mycena crocata*
AAGCGGATCATTAAATCGATAGCGCCATCCATTTACGGTCACGAGGATATTAAGACTGC
CATCGCGCTCTCGCTCTTTGGCGGTGTTCCAAAAGACATTGACCACAAGCATCGTATTC
GAGGCGACATTAACGTTCTCCTTCTCGGCGATCCTGGAACGGCCAAATCGCAGTTTTTG
AAATACGCGGAAAAGACTGCACACAGATCTGTGTTCGCCACTGGACAGGGCGCATCA
GCCGTTGGTCTCACAGCTAGTGTGCGTAAGGACCCGATCACTCGTGAATGGACGCTCG
AAGGCGGAGCCCTGGTGCTTGCCGACAAGGGCACCTGTCTTATCGACGAGTTCGACA
AGATGAACGA

> Mcm2 (MS463)_*Boletus edulis*
AAGCGCATCATCAAATCTATTGCCCCGTCAATATATGGCCATGAAGACATTAGGACTGC
CCTTGCCCTCTCCCTCTTTGGTGGCATCCCAAAAGATGTCAAACGAAAGCATCCCATCC
GAGGTGACATTAACGTTCTCCTCCTTGGTGACCCTGGTACAGCAAAGTCTCAGTTCTTG
AAATACGTGGAAAAAACTGCGCATCGGTCTGTCTTCGCCACTGGGCAGGGGGCATCTG
CCGTGGGTCTGACAGCCAGTGTTCGCAAAGATCCCGTCACCCGTGAGTGGACACTCG
AAGGCGGAGCGCTCGTCCTGGCCGACAAAGGAACATGCCTTATTGATGAGTTCGATAA
AATGAATGA

> Mcm2 (MS463)_*Amanita muscaria*
AAGCGCATCGTTAAGTCTATCGCACCTTCGATCTTTGGCCATGAAGATATCAAGACTGC
TATCGCCCTTTCGTTGTTTGGGGGCGTATCAAAAGATATTAATCACAAGCATAGAATCCG
TGGCGACATTAATGTTTTGTTGCTAGGTGATCCTGGAACTGCTAAATCTCAGTTCTTAA
AATATGTAGAGAAGACCGCGCACCGTTCCGTGTTTGCAACAGGTCAGGGTGCTTCAGC
AGTCGGTCTTACAGCCAGCGTTCGCAAGGACCCGGTTACTCGTGAGTGGACCCTTGAA
GGCGGTGCACTCGTTTTGGCAGACAAGGGCGTCTGTCTAATTGATGAGTTCGACAAAA
TGAACGA
> Mcm2 (MS463)_*Gautieria morchelliformis*
AAGCGAATCATCAAGTCTATAGCTCCCTCCATCTATGGACACGAAAACATTAAGACAGC
CATCGCACTCTCCCTCTTTGGAGGTGTCGCTAAGGACGTGAATCGCAAGCACCGAATC

CGGGGGGATATTAACATCTTACTCCTGGGGGACCCTGGTACCGCCAAATCACAGTTTCT
CAGATACGTAGAGAAGACGGCCCACAGGGCAGTATTCACCACGGGTCAGGGTGCCTC
CGCTGTCGGTTTGACGGCAAGTGTGCGAAAGGACCCCGTTACCAGGGAGTGGACCCT
TGAGGGAGGCGCACTAGTTCTCGCGGACAAAGGAACTTGCATGATTGACGAATTTGAC
AAGATGAATGA

> Mcm2 (MS463)_*Ramaria acris*
AAACGAATTATCAAGTCCATTGCTCCTTCCATCTATGGGCACGAGAACATCAAGACAGC
CATAGCACTTTCGCTTTTCGGAGGCGTTGCAAAGGACGTAAACCGCAAACACAGGATT
CGAGGTGACATCAACGTGCTCTTGCTGGGTGATCCCGGTACAGCTAAATCACAATTTCT
CAAATACGTGGAGAAGACCGCACATCGCGCAGTATTCACAACGGGTCAAGGCGCATCC
GCTGTCGGTTTGACAGCAAGTGTACGGAAAGACCCGGTTACTCGTGAGTGGACACTC
GAGGGAGGCGCACTAGTTCTCGCGGACAAAGGAACCTGCATGATCGATGAATTTGATA
AGATGAACGA

> Mcm2 (MS463)_*Calocera cornea*
AAACGCATCATCAAGTCCATCGCGCCCTCCATCTTCGGACACGACGACATCAAGGCAG
CCATCGCGTTGTCGCTCTTCAGCGGCGTGCCCAAGGACGTCAAGGGCAAGCATCGCGT
GCGTGGAGATATCAACATCCTCTTGCTCGGTGACCCGGGCACGGCCAAGTCCCAATTC
CTCAAGTACGTGGAGAAGACGGCGCACAGGGCGGTGTTCACGACTGGCCAGGGCGCT
TCCGCCGTCGGTCTGACCGCCAGCGTGCGCAAGGACGCAGCCACGAGGGAGTGGACA
CTCGAGGGCGGCGCGCTCGTCCTGGCGGACAAGGGCGTGTGCCTGATCGACGAGTTC
GACAAGATGAACGA

> Mcm2 (MS463)_*Dacryopinax primogenitus*
AAGCGTATCATCAAGTCCATTGCGCCCTCCATCTTCGGACACGACGACATCAAGGCCG
CCATCGCGCTGTCCCTTTTCAGTGGGGTACCCAAGGACGTCAAAGGCAAGCATCGTAT
TCGTGGAGACATCAACGTGCTTCTGCTGGGTGACCCGGGTACCGCCAAGTCCCAGTTC
CTCAAATACGTGGAGAAGACCGCGTATCGTGCGGTGTTCACCACCGGCCAGGGCGCTT
CTGCAGTCGGTCTCACTGCAAGCGTACGCAAAGACCCAGCAACGCGAGAATGGACAC
TTGAAGGCGGCGCCCTCGTGCTCGCTGATAAGGGGGTGTGTCTGATCGACGAGTTTGA
CAAGATGAACGA

> Mcm2 (MS463)_*Agaricostilbum hyphaenes*
AAGCGCATCATCAAGTCTATTGCGCCTTCCATCTATGGTCATGAAGACATCCGAACGGC
TCTCGCGCTATCGCTCTTTGGTGGTGTGGCCAAAGACATCAACCGCAAGCACCGTATC
AGAGGCGACATCAATGTCCTGCTGCTCGGTGACCCTGGTACCGCCAAGTCACAATTCC
TCAAATATGTCGAAAAGACTTCCAACCGAGCTGTCTTTGCAACAGGTCAAGGTGCTTC
TGCCGTTGGTCTTACGGCCAGCGTGAGGAAAGACCCAGTCACCAGAGAGTGGACGTT
GGAAGGTGGTGCTCTGGTGTTGGCCGACAAGGGCGTCTGCCTCATTGATGAGTTTGAC
AAGATGAACGA
> Mcm2 (MS463)_*Asperigillus triticus*
GACAAGATCGTGCGGTCCGTGGCGCCCAGCATCTACGGACACGAGGACGTCAAGACG
GCCGTCGCGCTGTCGCTCTTCGGCGGCGTGCCCAAAGAAGCCCAGGGCAAGATGTCC

ATCCGCGGCGACATCAACGTGCTCCTGCTGGGTGACCCCGGTACGGCCAAGTCCCAGA
TCCTCAAGTACGTCGAGAAGACGGCCCACCGCGCCGTCTTCGCCACGGGCCAGGGCG
CCTCAGCCGTGGGTCTGACGGCCAGCGTGCGGCGCGACCCGCTGACCAGCGAGTGGA
CGCTCGAGGGCGGCGCCCTGGTGCTGGCCGACCGCGGCACGTGCCTGATCGACGAGT
TCGACAAGATGAACGA

> Mcm2 (MS463)_*Neurospora crassa*
GACAAGATCATCAACTCCGTCGCCCCTTCCATCTACGGACACACCGACATCAAGACCG
CCGTCGCCCTCTCCCTCTTCGGCGGCGTCGCCAAGCAAGTCGGCGCCCACCACATCCG
CGGTGACATCAACGTGCTGCTCCTCGGCGACCCCGGTACCGCCAAATCACAAGTACTC
AAGTACGCCGAAAAGACGGCCCACCGCGCCGTCTTCGCCACCGGCCAGGGTGCTTCC
GCCGTCGGTCTGACGGCTTCCGTCCGGCGCGACCCGCTCACGTCCGAATGGACTCTCG
AGGGCGGCGCCCTCGTCCTCGCCGACAAGGGCACCTGCCTGATCGACGAGTTCGACA
AGATGAACGA

> Mcm2 (MS463)_*Rhizopus microsporus*
AAACGTATTATGAAGAGTATTGCACCTTCCATTTATGGTCATGAAGACATCAAGAGAGC
CATTGCTTTGGCTATGTTTGGTGGTGTGCCAAAGAATATCAAAGGAAAACACATGATCC
GTGGTGATATCAACGTGCTCATGCTTGGTGATCCTGGTACAGCCAAGTCTCAATTCCTC
AAGTATGTTGAAAAGACCGCCCATCGTTCGGTCTATACGACCGGTCAAGGTGCCAGTG
CTGTCGGTTTAACAGCTTCTGTTCATAAGGACCCTGTGACGCGTGAGTGGACCTTGGA
AGGTGGTGCACTCGTGTTGGCTGATCGTGGTGTTTGTCTGATTGATGAATTTGACAAGA
TGAACGA

>Met6 (FG740)_*Agrocybe pediades*
GGCGTAAGTCCCTGTCAAACTCCTTGTGGCATCTCTTGCTCATTCTTTTTAGTTGCCCC
TCCGTCGCTCTGATTGGGACGCTTACCTCAAGTGGGCCGTCGACTCTTTCAAGCTCGCT
ACTGCCGGTGTTACCGATCAACTCCAGACCCACTCCCACTTCTGCTACTCCGACTTCGA
CGACATCTTCCCATCCATCCAACGTCTCGATGCTGACGTTATCTCCATCGAAGCTTCGA
AGAGCGACATGAAACTCCTCAACACCTTCAAGCAATACGGCTACTCT

> Met6 (FG740)_*Mycena crocata*
GGTGTACGTTCCATCTTTTATCGTTGCGATTGTGGTGATTGATTATTTTGCAGCTGCCTCT
TCGTCGCTCGGACTGGGACGCCTACCTCAAGTGGGCCGTCGACTCGTTCAAGCTCTCC
ACTGCTGGTGTGACCGACAAACTCCAGACCCACTCTCACTTCTGCTACTCCGACTTTG
ACGACATCTTCCCCTCCATCCAGCGTCTCGATGCCGACGTCATCTCCATCGAGGCGTCC
AAGGCCGACATGAAGCTGCTGAACACCTTCAAGCAGTACGGCTACTCC

> Met6 (FG740)_*Stereum hirsutum*
GGTCTCCCCCTTCGTCGCTCTGACTGGGACAGCTACCTCAAGTGGGCTGTCGATTCCTT
CAAGCTCTCTACTGCTGGCGTTACCGATGCTCTCCAGACTCACTCTCACTTCTGCTACT
CCGACTTCGACGACATCTTCCCCTCCATTCAGCGCCTCGACGCTGATGTCATCTCCATC
GAGGCATCGAAGAGCGACATGAAGCTCTTGAACACCTTCAAGCAGTACGGCTACTCC

> Met6 (FG740)_*Coprinopsis cinerea*
GGAGTACGTTTTTGACCTTTGTTATGACTTCAATTCCTGACAACATGGCGCAGTTACCT
CTCCGTCGCGGGGACTGGGACGCCTACTTGAAGTGGGCTGTCGATTCATTCAAGCTCG
CCACCGCCGGCGTCACCGACAAGCTCCAGACCCACTCTCACTTCTGCTACTCCGACTT
CGACGACATCTTCCCCTCCATCCAACGCCTTGACGCTGACGTCATCTCCATCGAGGCCT
CCAAGAGCGACATGAAGCTTCTCAACACCTTCAAGCAGTATGGATATTCC

> Met6 (FG740)_*Dacryopinax primogenitus*
GGTCTTCCTCTTCGCCGTGTTGACTGGGATGGCTACCTCAAGTGGGCTGTGGACTCCTT
CCGCCTGTCGACGGCCGGTCTTACCGACAAGACCCAGGCTCACTCCCACTTTTGCTAC
TCCGACTTCAACGATATCTTCCCCTCCATCCTCGCCCTCGATGCTGATGTCATCTCCATC
GAGGCGTCGAAGAGCGACATGAAGCTGCTCAACGCCTTCAAGGAGTTCAGCTACTCC

> Met6 (FG740)_*Calocera cornea*
GGTCTCCCCCTCCGCCGCGTCGACTGGGATGCCTACCTCACCTGGGCCGTCGACTCGT
TCCGCCTCTCGACCGCTGGTCTTACGGACAAGACCCAGGCCCACTCCCACTTCTGCTA
CTCTGACTTCAACGACATCTTCCCCTCCATCCTGGCGCTGGACGCCGATGTCATCTCCA
TTGAGGCGTCGAAGAGCGACATGAAGCTGCTCAACGCCTTCAAGGAGTTCAAGTACT
CC

> Met6 (FG740)_*Fomitopsis pinicola*
GGTCTTCCGCTCCGCAAGGCTGACTGGGACGCGTACCTCAAGTGGGCGCTGCCCTCGT
TCAAGCTCTCGACTGCCGGCGTCTCCGATAGCCTGCAGACGCACTCGCACTTCTGCTA
CTCCGACTTCGACGACATCTTCCCGTCGATTCAGGCCCTCGACGCCGACGTCATCTCCA
TCGAGGCGTCGAAGAGCGACATGAAGCTGCTCAAGACCTTCAAGCAGTACGGATACT
CG

> Met6 (FG740)_*Trametes versicolor*
GGTCTGCCTCTGCGCAAGGCCGACTGGAACGCGTACCTGGCGTGGGCCCTGCCCTCGT
TCAAGATCGCGACCGCCGGTGTCACCGACGCGCTCCAGACGCACTCGCACTTCTGCTA
CTCCGACTTCGACGACATCTTCCCGTCCATCCAGGCTCTCGATGCGGACGTCATCTCGA
TCGAGGCGTCGAAGAGCGACCTGAAGCTGCTCAACACCTTCAAGCACTACGGCTACT
CG

> Met6 (FG740)_*Laccaria bicolor*
GGGGTCGGTAATCCAAAATTTCTCTTTCTGCCGCTAACTTCTGTTTTTCCTAGTTGCCTC
TTCGTCGTTCTGACTGGGACGCGTACCTCAAGTGGGCTGTTGATTCCTTCAAACTTTCC
ACTGCTGGGGTCACCGATCAACTCCAGACCCACTCTCACTTCTGCTACTCTGACTTCGA
TGATATCTTCCCCTCCATCCAGCGCCTTGACGCCGATGTCATTTCGATCGAAGCCTCTAA
AGCTGATATGAAACTGCTCAATACGTTCAAACATTACGGCTATTCC

> Met6 (FG740)_*Phanerochaete carnosa*
GGTGTAAGTTTCATTCACCCTGATATGGGCCTTGACTGATTACTTCTGTATAGCTTCCTC
TTCGCAGGGTTGACTGGGATGCGTATCTCGGTTGGGCTCTTCCTTCCTTCAAGCTCGCC

ACCGCCGGCGTCACCGATGCTCTGCAGACCCACTCCCACTTCTGCTATTCCGACTTCGA
CGACATCTTCAGCTCCATTCAGGACCTTGACGCCGATGTCATCTCTATCGAGGCCTCGA
AGAGCGACTTGAAGCTGCTCAACACCTTCAAGCGGTACGGCTACTCC

> Met6 (FG740)_*Serpula lacrymans* var. *lacrymans*
GGTGTGAGTAACTCCCATTCTACTTTATGACAGTAATTGAATATTTGCTACAGCTTCCTC
TCCGCCGATCTGACTGGGACGAATATCTCGAGTGGGCAGTCGACACCTTCAAGCTTTC
CACTGCGGGTGTCACCGATGCTACCCAGACGCACTCACACTTCTGTTACTCTGACTTTG
ACGATATCTTCCCTTCCATCCAGCGCCTTGACGCTGATGTGATTTCCATCGAGTTCTCCA
AGAGTGATATGAAGTTGTTGCACACCTTCAAACAGTACGGTTACTCC

> Met6 (FG740)_*Hygrophoropsis aurantiaca*
GGCGTGAGTAGACTTTATCTGAAACACGTGATCCTAAGATGCATTTCCTCCAGCTGCCC
CTCCGTCGCTCTGATTGGGACGAGTATTTGGAATGGGCTGTTGACACGTTCAAGCTGTC
CACTGCTGGTGTCACCGATGCTACCCAGACTCACTCCCACTTCTGCTACTCCGACTTCG
GCGACATCTTCCCTTCGATCCAACGTCTTGATGCTGATGTCATCTCCATCGAGTTCTCTA
AGAGCGACATGAAGCTCTTGCAAACCTTCCAGCAATATGGATACTCG

> Met6 (FG740)_*Agaricus bisporus* var. *bisporus*
GGTGTAAGTTGTTTTACCTGTTTTGAAGGATTATAGTGATTGATACTCTGTAGCTTCCCC
TCCGCCGGGCTGACTGGGATGGCTATCTTGAGTGGGCTGTAGACTCTTTCAAGCTTGC
AACAGCTGGTGTCACTGATCAACTTCAGACTCACTCTCACTTTTGCTACTCTGACTTTG
ACGATATTTTCCCGTCTATTCAACGCCTTGACGCTGATGTTATTTCGATCGAGGCTTCCA
AGAGTGACATGAAACTGCTCAATACCTTTAAGCACTATGGCTATTCC

> Met6 (FG740)_*Pluteus cervinus*
GGTGTAAGCTCTCATTGTAATGCATACTTCATAATTTCTGATGTCACTTTCAGCTTCCTCT
TCGACGTGCCGATTGGGATGCATACCTCAAGTGGGCCGTGGACTCCTTCAAACTCGCC
ACCGCTGGTGTTGGCGACACTCTTCAAACCCACTCCCACTTCTGCTACTCTGATTTCGA
TGATATCTTCCCATCGATTCAACGACTTGATGCTGATGTTATCTCCATCGAGGCGTCAAA
GAGTGACATGAAGTTGCTTACCACCTTCAAGCAATACGGTTACTCC

> Met6 (FG740)_*Tricholoma matsutake*
GGCGTAAGCGTTTTTAAAGCACTATCTTTCGTGTTAAGGCTAATTGTGTCTAGTTGCCCC
TTCGTCGTGCTGACTGGGACGCCTATCTCAAATGGGCGGTTGACTCGTTCAAGCTTGCC
ACAGCCGGTGTTACTGACCAGTTGCAGACCCACTCTCACTTCTGCTACTCCGACTTTG
GAGACATTTTCCCTTCCATCCAGCGTCTTGACGCTGATGTCATCTCGATAGAAGCTTCC
AAGAGTGACATGAAGCTCATCCAAACTTTCAAGCAGTACGGTTACTCT

> Met6 (FG740)_*Gymnopilus chrysopellus*
GGCGTATGTACATCCTCACTCTGTCTTAAACATCTGCTCAATCATCTACTTAGCTACCCC
TTCGCCGCTCTGACTGGGACAACTATTTGAAATGGGCGGTCGACTCCTTCAAGCTCGC
AACTGCTGGCGTCAGCGACCAACTTCAAACCCATTCCCACTTCTGTTACTCTGATTTCG
ACGATATCTTCCCTTCGATTCAGCGTTTGGATGCAGATGTGATTTCCATCGAGGCGTCCA

AGAGCGACATGAAACTCCTCTCGACCTTCAAGCAGTATGGATACTCG

> Met6 (FG740)_*Gymnopus androsaceus*
GGTGTAGGTGTCATCTTACAGCTTTTAATTTTATTCATGGATTTGCTCTTTAGCTTCCCCT
TCGTCGCGCAGACTGGGACTCCTACCTGAACTGGGCCGGAAACACCTTCAAGCTCTCG
ACTGCCGGTGTCGGTGACCACACCCAGACCCACTCTCACTTCTGCTACTCCGACTTTG
ACGACATCTTCCCTCAAATCCAGCGTCTCGACGCTGATGTCATCTCTATCGAAGCATCG
AAGAGCGATATGAAGCTTATCAACACCTTCAAGCACTACGGATACTCC

> Met6 (FG740)_*Auricularia delicata*
GGTGTAAGTTTCCATCTATTTCCATAACGTTTAGTTGCTGACGGCGTCCGCAGCTTCCTC
TCCGCCGCGTTGACTGGGACGACTACCTCACTTGGGCTGTCGACTCTTTCCGCCTCGC
CACTGCCGGTGTTGAGGACAGCACGCAGACGCACTCGCACTTCTGCTACTCGGACTTC
AACGACATCTTCCCGTCCATCCAGCGCCTCGACGCGGACGTCATCTCGATCGAGGCGT
CGAAGAGCGATCTGAAGCTGCTCGACGCGTTCAAGCAGTACGGCTACTCG

> Met6 (FG740)_*Fomitiporia mediterranea*
GGTGTATGTATTAAGCACTTATATACATAGCATATTGACTTCGCTCTTCCCAGCTCCCACT
CCGTCGCGCTGACTGGGATGACTACCTCCGCTGGGCCGTGGACACTTTCAAGCTCGCA
ACAGCTGGTGTCTCCGATGCCACGCAGACACACTCTCACTTCTGCTACTCAGACTTCA
ACGACATCTTCCCTTCCATTCAGCGCCTTGACGCGGATGTCATCTCCATTGAGGCGTCC
AAGAGCGACATGAAGTTGCTTGCTGCCTTCAAGGCATACGGTTACTCG

> Met6 (FG740)_*Marasmius fiardii*
GGTGTAAGTTTTTCGTCATGTGGTAATGTTCTGATTGTTCTGATCGTGTATAGCTTCCTC
TTCGTCGCGCGGACTGGGACAACTACCTTGGATGGGCCGGGAATACCTTCAAGCTCTC
CACCGCTGGTGTCAAGGATGAGACCCAAACCCACTCTCACTTCTGCTACTCCGACTTC
GACGACATCTTCCCCCAGATCCAGCGCCTCGATGCTGATGTTATCTCCATTGAAGCGTC
CAAGAGTGACTTGAAGCTTATCAAAACCTTCAAGCACTACGGGTACTCC

> Met6 (FG740)_*Boletus edulis*
GGCGTAAGTTGATTCGAAAGATAAACGCGTGAGCTCTCTGACATCCATGCTAGTTGCCT
CTTCGTAGATCTGACTGGAATGAGTACCTCGGATGGGCTGTCGACACTTTCAAGCTTGC
TACGGCAGGTGTCGAAGATGGAACTCAGACTCATTCCCATTTCTGCTACTCCGACTTCG
GCGATATCTTTACGTCCATTCAACGTCTTGACGCTGATGTCATTTCCATCGAGTTCTCAA
AGAGCGACATGAAATTGTTGCATACCTTCCAGCAGTACGGCTATTCC

> Met6 (FG740)_*Amanita muscaria*
GGTGTAAGTGATAGCGTTTCAGGCACCGTACGCTTGCTAACAACTCTTCAGCTTCCTCT
CCGCCGCGTAGACTGGGATGCATACCTTAAATGGGCTGTTGACTCCTTCAAGCTTTCCA
CTGCAGGTGTCACCGACAAATTGCAAACGCATTCGCACTTCTGCTACTCGGACTTTGA
CGATATCTTCCCATCGATCCAGCGGCTCGATGCTGATGTCATCTCCATCGAAGCTTCCAA
GAGTGACATGAAACTCCTCAACACTTTCAAGGCTTACGGCTATTCA

> Met6 (FG740)_*Ramaria acris*
GGAGTAAGCGTTTGTCAACTCATCCTATTGTATTTACGTTTACTTTTCTTCAGCTACCCC
TTCGCCGCGTCGACTGGGATAACTACCTCACCTGGGCCGTCGACTCCTTCAAGCTGTC
CACAGCAGGTGTCACCGATGCATTGCAAACCCACTCTCACTTTTGCTACTCAGACTTTA
ACGATATCTTCCCGTCAATCCAACGTCTCGATGCTGATGTTATTTCCATTGAGGCTTCGA
AGAGTGATCTTAAACTCCTCAGTGCCTTCAAACAGTTTGGTTACTCT

> Met6 (FG740)_*Gautieria morchelliformis*
GGGGTGCGTGTCTTGGCCTGATTAGCAATACTCACGTTATTCTCCGTTTACAGTTGCCG
CTTCGTCGCGTAGATTGGGACAACTACCTCACCTGGGCTGTCGACTCATTTAAACTTGC
CACTGCTGGTGTCACCGACTCTTTGCAGACTCACTCTCACTTCTGTTATTCGGATTTCA
ATGATATCTTCCCCTCGATACAGCGTCTTGATGCCGACGTCATCTCTATCGAAGCTTCTA
AGAGTGACCTTAAGCTCCTTAGCGCCTTTAAGGAGTTCGGCTATTCG

> Met6 (FG740)_*Lactarius quietus*
GGTTTGCCTCTCCGTCGCGCAGACTGGGATAACTACCTCAAGTGGGCCGTCGACTCGT
TCAAGTTGTCGACCGCGGGTGTAAGCGATGCCTTACAGACCCACTCACACTTCTGCTA
CTCGGACTTCGGCGATATCTTCCCTTCGATCCAGCGATTGGATGCAGATGTCATCTCGAT
TGAGGCTTCTAAAAGTGACATGAAGCTAATCACCACGTTTAAACACTACGGATACTCG

> Met6 (FG740)_*Asperigillus triticus*
GGTCTGCCCCTGCGCGGCACCGAGCGCGAGAAGTACCTGCAGTGGGCCGTCAAGGCC
TTCCGCCTGGCCACCGCCGGCGTCACCGACGGCACCCAGATCCACTCCCACTTCTGCT
ACTCCGAGTTCCAGGACTTCTTCCACGCCATCGCGGCCCTGGACGCTGACGTGCTCTC
CATCGAGAACAGCAAGTCGGACGCCAAGCTGCTCAAGGTCTTCATCGACGAGGCCTA
CCCC

> Met6 (FG740)_*Neurospora crassa*
GGTCTCCCTCTCCGTGGTCAGGAGCGTGAAGCCTACCTCAAGTGGGCTGTCGACTCCT
TCAAGCTCGCCACCGCTGGCGTCGAGAACTCCACTCAGATTCACTCTCACTTCTGCTA
CTCTGAGTTCCAGGACTTCTTCCACGCCATCGCTGCCCTTGATGCCGATGTCCTCTCCA
TCGAGAACTCCAAGTCTGATGCCAAGCTCCTCAAGGTCTTCATTGACGAGGAGTACCC
C

> Met6 (FG740)_*Agaricostilbum hyphaenes*
GGTCTGCCTCTCAGACGCTCCGACTGGGACGCCTACTTGACCTGGGCCGTCGACTCGT
TCCGTCTGTCCACCGCTGGTGTGCGCGATGACACTCAGACTCACTCGCACTTCTGCTAC
TCTGACTTCAACGCCATCTTCTCGCACATTGCCCGCCTTGACGCCGATGTCATCTCCAT
CGAGGCCTCAAAGAGCTCCCTCAAGCTGCTCGATGTCTTCACATCTAACAAGTACTCT

> Met6 (FG740)_*Rhizopus microsporus*
GGTTTGCCCATCCGTCGTGCTGATTGGGAAGCTTATCTCGAATGGTCTGTTGCCTGTTT
CCGTTTGTCTACTGCTGGTGTCCGTGACGAAACTCAAATCCACACTCACATGTGTTACT

CTGACTTTAACGATATCTTTGGTGCCATTACTGCTTTGGATGCTGATGTTATCACTATTGA
AAACTCCAAGTCTGATGAACGTTTGCTTCAAGTCTTCCAAACTCAAGGATATCCT

>Ppt1 (MS417) _*Agrocybe pediades*
CGTACTTATGGCTTTGAAGGAGAGGCAAAGCATAAACACGGAGAGCAGGCATATAAGG
TTCTTCGAGCTCATTTATCGTCTTTTCATTGTTGAATAACTGGACAAACAGCTTTTCGCC
CATGTCTTCACATGCTGTGAGTAATCTTTTGACCGATCAAGGGAATGGTGCTTAAAACG
ATTTCTAGTGCCGCTGGCCACACTTGTCAGTGCGACGAAGCCTCCAGCGACCAAGGAC
AATTCTATTCTTTCTCCAGAAGGCCGTAGACGTTTCTTTGTCGTTCACGGTGGCTTG

> Ppt1 (MS417) _*Serpula lacrymans* var. *lacrymans*
AGGACGTATGGCTTCGAAGGTGAAGCGAAGCACAAGCATGGAGAACAAACCTACAAG
GTTCGTCCTACTTAATATAGAGCACAGACACTATTTGACATTTTAATCAAGCTCTTTGCA
TACGTTTTTACGACTTGTAAGATACCAGTGTTCATATGGAATGCTAATTAAGGACCTAAT
TTGCCCTCTAGTACCGCTAGCTACACTGGTTAGCGCCACCAAGCTGCCTGCGACGCCTG
ACAAGGCCGCCATTCTATCACCAGAAGGGCGAAAACGTTATTTTGTCGTTCATGGTGGT
CTC

> Ppt1 (MS417) _*Hygrophoropsis aurantiaca*
CGCACATATGGCTTTGAAGGTGAAGCGAAGCACAAGCACGGAGAACAAACATACAAG
GTATACCTCTCCAATATTCATCGCCTATAGTCAAAATATCTCACTGATCATTCAACCTTCA
GCTGTTCGCGCATGTTTTCACGACGTGTATGCTCATTTGATCCATTCACAGCTTATTCCA
TTCTCAATTACATTCACATTTCCTCACTAGTACCACTTGCAACCTTGGTCTCTGCTACGC
AGCCTCCTTTGACTCGAAGCCCAAATACGATTCTCTCGCCTGAAGAACGAAAGCGATA
TTTTGTCGTGCACGGTGGCCTG

> Ppt1 (MS417) _*Gymnopilus chrysopellus*
CGCACATATGGCTTTGAAGGCGAGGCAAAACACAAACATGGAGAGCAATCGTACAAG
GTAGGCGTCAAGTTTTTTTTTCCGTAATTCGAATTTATCTTCGTTCAGCTATTCGCCCAT
GTCTTCACTACCAGTAAGTCACCGTTGTGTTTCATTTGATCAAACAACAGGGCATATTG
AAGATTGCTACAGTGCCATTGGCCACTCTTATCAGTGCAACTCAATCCCCTTCTTCTAA
AGACGATGCTATCCTTTCGCCTCAAGGCTTCAAGCGCTTCTTCGTGGTTCACGGAGGCT
TG

> Ppt1 (MS417) _*Phanerochaete carnosa*
CGGACGTACGGCTTTGAGGGTGAGGCGAAGCACAAGCATGGCGAGCAAACGTACAAG
GTACGTTAGATGCTATATCGTATCTGGGAGTTCTCACTAACCTGTCTCCTAGTTGTTCGC
TCATGTCTTCACAGCCAGCAAGTCGCACTCCTTGTGAATTAGATCACGACTCTGACATT
CCAGTTCTACAGTGCCACTGGCAACCCTGATCTGTGCGACTAAACCTCCGTCATCACCT
TCGAAGAGCGCGATCCTGTCACCTGAGGGCAAGAAGCGGTTCTTCGTTGTGCACGGC
GGCCTA
> Ppt1 (MS417) _*Trametes versicolor*
CGCACGTACGGCTTTGAGGGCGAGGCGAAACACAAGTTTGGCGAGCAATCATACAAG
GTCAGTGCACCATCTTCTCGACAGATAGGAATTTCGCTGACACGCCCAACAGCTCTTC

GCGCACGTATTCACAGCCAGTAAGCAGCTTGCGCTAGTCTCGCGCGGAATGAATGGGT
ACTGAGCTCGTCTTGTAGTGCCCTTGTCCACCCTCGTTTCCGCAACCCAGCCGCCAGTA
GCGTCCGCAAAGAAAGCCATCTTGTCGCCCGAGGGTCGCAAGCGGTATTTCGTCGTGC
ACGGCGGACTG

> Ppt1 (MS417) _*Fomitopsis pinicola*
CGTACGTATGGCTTCGAAGGTGAAGCGAAACACAAGCATGGCGAGCAGACGTATAAG
GTGCGACCTCCGCTTTGGCAATGCACGTGCTACTATCTGACTCCATCTTCGTGCTTCGA
GCTATTCGCACATGTCTTCACCGTCAGTGAGTATTCATTGGTGTATACTCACTTGGAAGC
CTCTGACCACGCTACCAGTGCCCCTTGCGACCTTGATCTGCGCGGAGAAGCCGCCGGA
AGGGCCTTCACCTGGCGCTATTTTGTCGCCAGAAGGCCGTAAACGCTATTTTGTTGTAC
ATGGCGGTCTA

> Ppt1 (MS417) _*Schizophyllum commune*
CGCTCCTACGGCTTCGAGGGCGAAGCGAAGACAAAGCACGGCGAGCAGAGCTACAA
GCTGTTTGCGCATGTCTTCACTACATGTGAGTTCTACCTTTGAACGCCGACATGGGGCT
CTACTGACAATAATATCAGTACCTCTCGCTACCCTAATATGCGCCTCGAAGCCGCCACC
GAGCCCTGGGAAGGCTATTCTATCATCAGACGGCAAAAAGCGGTACTTTGTCGTGCAC
GGGGGCCTC

> Ppt1 (MS417) _*Mycena crocata*
CGATCTTATGGGTTTGAAGGCGAGGCGAAACACAAACACGGCGAACAGACCTACAAG
GCGCGTTTGTTCTGTTTTTTATTCCACCTTGCAACTGATTTAATCAACGCAGCTCTTCGC
ACACGTTTTCACGACATGTGAGTCGT
ACTTGCATTCTCCACCCTGGGATATGAACCTGAGTCATATCTAGTGCCGCTGTCCACGC
TTCTGAGCGCAACAAAACCACCGCCGAATCAAGCCGGCAGCCCTATCTTATCGCCGCA
GGGATTAAAGCGGTACTTCGTCGTTCATGGAGGGCTC

> Ppt1 (MS417) _*Stereum hirsutum*
CGGACGTACGGGTTCGAAGGCGAAGCGAAACACAAGCATGGCGACCAAACTTACAAG
GTAAAGACTTTCGCTGTCGTGACTGTCGCGGTACTTCACTGAGAATTTTTACTACAGCT
CTTTGCCCATGTCTTCACTGCCAGTTCGTTTTTCCATTCCATTGATGCTGCATAAACGTC
AGACTCATCATGCATTGCTGCAGTGCCTTTGGCATCTCTTATAAGCGCAACCAAGCCAC
CAATCTCGTCGCAGGGTGCTATCCTATCTCCAGACGGCCTCAAACGGTACTTCGTCGTG
CATGGAGGTCTC

> Ppt1 (MS417) _*Lactarius quietus*
CGTACATATGGGTTCGAGGGCGAGGCGAAACACAAGCACGGAGAACAAACATACAAG
GCAAGTCCCATCGCATCGCTTGATCGAGCATCCCATTAACAGTATTCTTCCTCAAGCTCT
TCGCTTATGCATTTACCGCCCGTCTGTTCCTTGCCGTCTCTCCGTCTGGGATTCCTGCTG
ACAGACCTTAGTGCCTCTCGCCACCCTTGTCAGCGCAACGCTACCTCCTGCTAAGAAA
CAGGATGTGATTCTTTCTCCCGATGGCTTTAAGCGGTACTTCGTCACGCATGGAGGCCT
C

> Ppt1 (MS417) _*Laccaria bicolor*
AGAACATATGGCTTTGAAGGCGAGGCAAAACACAAACACGGAGAACAGTCATACAAG
GCCAGTCTCCTACTCCATCCACACTTCTTGGTAGACTTACCGTGAAACGCACAGCTTTT
TGCTCATGTCTTTACGACCTGTCAGTTCGAGTTGCAGTCGAAAATTATCCCCACATTTT
GGCTTATATGTTCTAGTGCCGCTCGCTACCCTTGTGTCCGCAACGAAACCACCGCCCCC
GAAGGAAAGGATAAACGCTATTCTTTCGCCAGATGGTCTAAAAAGGTTCTTTGTTGTGC
ACGGTGGACTA

> Ppt1 (MS417) _*Gymnopus androsaceus*
AGCACATATGGCTTTGAAGGTGAAGCGAAACATAAACACGGCGACCAGTCATACAAGG
TACGCGTTATTGTTCTCTTGGAAACCTACGTCTCATCAACGTACATTTCCAGCTGTTCGC
CCACGTCTTTACCACATGTGAGTCTACTTATCCCCGACGTTTAGTTACATTGTTTAGCTC
CATCTATAGTACCTTTGGCCACACTCATCTGCCCCACCCAACCTCCTCGCATTAAGAAA
GCAAATGATATTCTCTCACCAGAAGGAAAGAGACGCTTCTTTGTCGTACACGGAGGTT
TG

> Ppt1 (MS417) _*Tricholoma matsutake*
CGGTCCTATGGATTTGAAGGGGAAGCCAAGCATAAGCACGGAGAACAATCATACAAGG
TTTGCACCACGTCGACCGCTCTCTGATTAACTATTAATACCATTTAAGCTTTTTGCCTAT
GTTTTTACTACATGTGAGCCTCTTTATAGAAGATATATGCTCTCTGGCACACACTAACGT
TTTTCCCTTTAGTACCACTCGCAACCCTCATCAATGCCACCCAACTGCCTCTCGCGAAG
AAAAGTCCTAACGCCATCCTTTCACCGCAAGGATATAAACGATACTTGGTCGTTCACGG
AGGGCTG

> Ppt1 (MS417) _*Agaricus bisporus* var. *bisporus*
CGGACATACGGGTTCGAAGGCGAAGCCAAGCAAAAACACGGTGACCAGAGTTACAAG
GTGAGTCGAAAATCAATGTACATCGCGCCACGCTCATGACATTATAAGTTATTTGCGCA
CATATTCACCACCTGTATGTCGACTCATTTTCTTCTCTTACTTTATACTCATCGTTCGACA
GTGCCTTTGGCAACTCTGGTGAGCGGTACCAAGCCTCCAACGACCAAGGAAGCCGCC
GGTGCCATACTCTCACCTGATGGATTCAAACGGTATTTCGTTGTTCATGGTGGTTTG

> Ppt1 (MS417) _*Boletus edulis*
CGGACGTATGGCTTCGAAGGAGAAGCCAAGCATAAGCATGGGGAACAGACGTACAAG
GTGAGGCGATTTAAAGCATTCTTTTCAGAGACTGAGTTGCCAAACATAGCTTTTTGCCC
ATGTATTTACGGAGAGTGCGTGGCTCGATCGAAGGCTAGATATCATATGAGAATCTGAA
CCTTCACACAGTGCCACTGGCAACTCTTATCAGCGCAACCACCCCATCCAAGGACGTC
ACTTCGAAAACAATATTATCTCCGGAAAAGCTCAAACGTTATTTTGTTGTCCATGGCGG
GTTG

> Ppt1 (MS417) _*Marasmius fiardii*
AGCACGTATGGATTCGAGGGTGAAGTCAAGCATAAACATGGGGACCAGACCTACAAG
GTCAGTTCAGTCTTTCGTTTCTGTCAATGAACCACAACGCTGACACTTCGAAAGCTCTT
CGCATACGTATTCACAACGCGTAAGTGGATTTGTTGAGCATATTAGAGCCCCCTTTGAA
CAGACCATGCGCAACTCAGTACCTTTGGCAACCCTCCTTAGTGCAGCAAAACCACCAA

TTTCAAAGACGCCTCAAACAATCCTGTCACCTCAGGGTACCAAACGATACTTCGTCGT
GCATGGTGGCCTC

> Ppt1 (MS417) _*Pluteus cervinus*
CGCTCCTATGGCTTCGAGGGCGAAGCGAAGCACAAACATGGGGAACAGACATTCAAG
GCATGTTTTTCCGTTACATGTGTTCACTCAGTCCCAGTGACTCACATAAACTAACACATA
TAGTTATTCTCTCATGTGTTCACAGCAAGTGAGTCCCATCAAGTCAAGTCCGGGTGTCG
TTCTAAGATTGGCAAGTACCCCTGGCCACCCTTTTGAGCGCCACCAAAGCACCATCAG
AAAGTGAAGCAAAGAAGTCGATCCTGTCACCAGAAGGCTTTAAACGGTATTTCGTGGT
TCACGGTGGATTG

> Ppt1 (MS417) _*Auricularia delicata*
CAGATGTACGGCTTCCAGGGCGAATGCGAGAAGAAGAGTGGAGAGCTTTCGTACAAG
GTGAGCGCGCATTGCTGCGCTTCATGAACAAAACTGAAAGCAGATCTAGCTCTTTACG
CATGTGTTCACCTCATGTAAGTTCCATG
AACGCCCAATGCAGCATTGGCTCAATCTATCTGATAGTGCCGCTTGCAACGTTAATCTC
TGCAACAAAGCCGGCGACGGGGGCGGCGCTGAACAGCAACCCGATTCTGTCGTCGGA
TGGGCGGAAACGGTTCTTCGTTGTGCACGGCGGCCTA

> Ppt1 (MS417) _*Gautieria morchelliformis*
TCGGTCTACGTCAGTCCGTCCGTTGTTTCCTTATATATCTCCAATCCATTCATGAGTGCTT
CAGGGTTTTGAGGGTGAGGCGAAGCACAAGCACGGCGAACTGACATACAAGGTGTGT
CGTCTCATCTAACAATGAATCTTGGGCATTGCTGATGACGTTGTCTAGTTGTTTGCTCAC
GTATTCACGGCCCTGCCGTTGGCGACCCTGGTCTCCGCATCACTTCCGCCTACTCCTTC
AGAGAAACCAACTATTCTCTCTCCTGAGGGTCGGAAGCGCTATTTTGTTGTACATGGCG
GGCTG

> Ppt1 (MS417) _*Ramaria acris*
TCAGTTTATGTCTGTGTTCATCTTATTGATAGAGCTCAATCCATTCATACATTCTCCAGGG
CTTTGAGGGCGAAGCAAAACACAAACATGGAGAACTGACATACAAGGTATGCGTTCCT
CTACCCAGGCTGTCAGTGTCTGATAGAAGAAATACTACATCTAGTTATTCGCTCATGTAT
TCACAGCTCTACCATTAGCGACTTTGGTTTCGGCGTCTTTACCATCCACGTCCTCAAAG
ACGCCGATTATTTTATCCCCAGAAGGCCGAAAGCGGTATTTTGTAGTACACGGCGGCCT
A

> Ppt1 (MS417) _*Neurospora crassa*
AGGGTGTATGGCTTCGAGGGCGAGTGCAAGCACAAGTACAATGAGAGGTAAGCGAGT
CGAACTCTTTGCGCCCATTATGCGACATCCTGAATTTTCGAGCACACACCAGCTGACAC
ATCTCACCTTCTTCCCAGAACCTACAAGCTCTTTTCGGAAAGTTTCTCGGCCCTCCCGC
TCGCCACGTTGATTGGCAAGAAGTTCCTCGTCCTCCACGGCGGTCTC

> Ppt1 (MS417) _*Asperigillus triticus*
AAGGTGTATGGATTCGAAGGCGAGTGCCGGGCCAAATACAACGAAACGGTCTTCAAG
GTCTTCTCCGAGTCCTTCTCCGCCCTGCCCCTGGCTACGTTGATCGGCAACAAGTATCT

CGTCCTCCACGGCGGTCTC

> Ppt1 (MS417) _*Calocera cornea*
AAGGTGTACGGGTTTGAGGGGGAGTGCAAGCATAAACTCGGGGAGATGACTTACAGC
GTGCGCATGTCTGGATGGAGTTCGTGGTGCCCTGGCTGACCATCACGCAGCTCTTCGA
GGACGTCTTCACCGCACTGCCGCTAGCCACGCTCATCTCTTGCACGCTCCCTCCCACAC
CCGAGCCCTCAAAGCCAATCCTCTCAGCAGAAGGAAAGAAGCGGTTCTTCGTAGTAC
ACGGTGGCCTC

> Ppt1 (MS417) _*Rhizopus microsporus*
AAAGTATATGGTTTTGAAGGCGAAGTAAAGGCAAAGTTTAGTGAAATGATGTTTAAATT
ATTTTCAGAAACATTCAATGCCTTACCACTGTCTCATGTTGTTGAAAACAAGATATTCGT
GACCCATGGTGGCTTA

>Pre8 (MS429)_*Agaricus bisporus* var. *burnettii*
ACGCAATCTGGGTAGGACGAATGAGTTTTTTGAGTTTCACTCTTTCGCTATCCCAGCGG
TGTCCGACCTTTCGGTGTCTCCTTATTAGTCGCCGGTTGGGACATTAACCGTGGCCCAT
CATTATATCAGGTAGACCCGTCAGGATCATTCTGGGCGTGGAAGGCCAGCGCGATTGGT
AAAAATATGATCAATGCAAAGACGTTCTTGGAGAAA

> Pre8 (MS429)_*Schizophyllum commune*
ACGCAGTCAGGGTGCGTAGCCCTAATGATAACTGTGTGAATGTGACCTAACCTATAGAG
GTGTCCGACCATACGGTGTCTCCTTGCTCGTCGCAGGCTGGGACAGCAATCGCGGCCC
TTCCCTGTACCAGGTTGACCCGTCAGGGTCGTTCTGGGCATGGAAAGCGAGCGCGATA
GGGAAGAACATGGTCAATGCGAAGACGTTCTTGGAGAAG

> Pre8 (MS429)_*Laccaria bicolor*
ACGCAATCCGGGTGAGACCCTTGGAAAATTAACTTTCCGCATGCTGATGTGCAGTTTAG
AGGAGTAAGGCCATATGGTGTTTCATTACTAGTCGCAGGCTGGGACAGTCATCGTGGCC
CGACGCTATATCAGGTGGACCCCTCAGGATCTTATTGGGCATGGAAGGCAAGCGCTATA
GGGAAAAACATGTTGAACGCAAAGACATTTTTGGAAAAA

> Pre8 (MS429)_*Coprinopsis cinerea*
ACCCAATCCGGGTGAGGACCCTTGTGACATTTGATTAACTTCTGTCAGCGCTGCCCTTA
GAGGTGTACGCCCTTACGGTGTTTCATTGTTAGTGGCAGGATGGGATAGTCATCGAGGG
CCAACATTGTATCAGGTTGATCCATCAGGGTCATTCTGGGCATGGAAGGCCAGTGCGAT
AGGGAAGAATATGGTGAACGCGAAGACTTTTTTGGAAAAA

> Pre8 (MS429)_*Agrocybe pediades*
ACCCAATCAGGGTACGTGTAAGCGCTGATTACAAATGATATTGCTGATACGTGACTCAG
AGGAGTGAGGCCCTATGGTGTCTCACTGCTTGTGGCAGGTTGGGATACTCACCGCGGT
CCTACGCTATATCAGGTTGATCCTTCTGGATCGTTTTGGGCCTGGAAAGCCAGTGCTATT
GGTAAGAATATGGTCAATGCTAAGACATTCCTGGAGAAA

> Pre8 (MS429)_*Gymnopilus chrysopellus*
ACTCAGTCAGGGTAAGACTCAAAGCGAATCCATGGGATCGTCATTGATTCGCGGTGCA
GAGGTGTGCGGCCATACGGCGTGTCGTTATTGGTGGCTGGTTGGGACACTCATCGAGG
ACCGCAACTCTATCAAGTTGATCCTTCCGGGTCTTTCTGGGCATGGAAAGCGAGCGCTA
TTGGTAAAAATATGGTAAACGCTAAGACATTCTTGGAGAAA

> Pre8 (MS429)_*Tricholoma matsutake*
ACACAATCTGGGTATGGCTTTCCTTTATTCATCGGACTTCATTCATTTGGAGAGTAGAGG
AGTGCGGCCGTATGGAGTGTCATTACTTGTCGCAGGATGGGATATCAACCGCGGCCCA
ACGTTGTATCAGGTAGATCCGTCAGGGTCCTTTTGGGCGTGGAAAGCCAGTGCCATCG
GGAAGAATATGGTCAATGCGAAAACCTTTCTAGAAAAA

> Pre8 (MS429)_*Hygrophoropsis aurantiaca*
ACGCAATCTGGGTAGCACTGCTCCTTAACCCTGACAAATTTGACTCACAATTTCATCAA
GGGGTGTACGACCATATGGTGTATCTCTCCTTGTTGCAGGCTGGGACTCACACCGGGGT
CCAAGTCTATTTCAAGTCGATCCTTCAGGTTCGTTCTGGGCGTGGAAAGCCAGCGCCAT
TGGAAAAAACATGGTCAACGCTAAAACGTTCCTGGAAAAA

> Pre8 (MS429)_*Gautieria morchelliformis*
ACTCAATCAGGGTTAGTCCCTCCCCCACGCATGAAAGATCATCTAAGTGATACCTTAGA
GGTGTACGACCGTATGGCGTGTCGTTACTCGTGGCAGGTTGGGATGCAAATCGTGGAC
CGAGTTTGTACCAAGTTGACCCCTCTGGATCATTCTGGGCCTGGAAAGCCAGCGCAAT
AGGAAAGAACATGGTCAACGCTAAAACTTTCCTCGAGAAG

> Pre8 (MS429)_*Ramaria acris*
ACACAGTCTGGGTCTGTGCATCCCATCATTCTACTTTCGCATACACATCATTTCAGAGGT
GTCCGACCTTATGGTGTATCGCTACTGGTCGCCGGCTGGGATGCAAACCGCGGTCCGA
GCTTATACCAAGTTGATCCATCCGGCTCGTTCTGGGCATGGAAAGCTAGTGCCATAGGC
AAAAACATGGTCAATGCCAAGACCTTCCTTGAGAAA

> Pre8 (MS429)_*Lactarius quietus*
ACTCAATCTGGGTACGCTTTCGACGTGACCCTCATAGCGATTGAATGGAATATTTGGCA
GTGGCGTACGGCCCTATGGTGTTTCACTGCTCGTAGCGGGATACGACCAAAACCGGGG
ACCGACGCTCTACCAAGTCGACCCCTCTGGCTCATTTTGGGCGTGGAAGGCTAGCGCG
ATAGGGAAAAATATGACCAACGCAAAGACATTTCTTGAGAAA

> Pre8 (MS429)_*Calocera cornea*
ACGCAGTCTGGGTACGTGACGTGGTAATGCAGGTTTAAATGGCTTCGTAGTGGTGTGA
GGCCTTTCGGTGTATCGCTGCTGGTTGCTGGGTGGGACGATCATCGTGGACCCTCGCTC
TACCAGATCGATCCTTCCGGTTCGTTCTGGGCGTGGAAGGCGAGCGCAATAGGGAAGA
ACATGGTCAACGCGAAGACGTTCCTGGAAAAG
> Pre8 (MS429)_*Amanita muscaria*
ACACAATCCGGGTAGGCTTGCCCTCTAATTCCAACTAATTTTATTAGTGGGGTCCGGCC
ATATGGTGTATCATTACTTGTCGCAGGCTGGGATATCACCCGCGGACCAACCCTTCACC

AGGTAGATCCTTCTGGATCCTTCTGGGCATGGAAAGCGAGCGCAATAGGCAAGAACAT
GGTGAATGCCAAAACCTTTCTTGAGAAG

> Pre8 (MS429)_*Phanerochaete carnosa*
ACACACTCAGGGTACGCTGTGTGATTGTCGTGACTCATCAAGCTATTTAGAGGTGTGAG
GCCATATGGCGTCTCACTGCTCGTGGCAGGCTGGGACTTCAACCGAGGACCCACGTTG
TATCAGGTTGACCCATCGGGATCCTTCTGGGCGTGGAAAGCGAGTGCGATTGGCAAGA
ACATGGTCAATGCAAAGACGTTCCTGGAGAAG

> Pre8 (MS429)_*Auricularia delicata*
ACACAGTCCGGGTAAATCACCATTATTACCGTTCCGCGCACGATAAAACGCGGGTTGC
AGCGGTGTTCGGCCATTCGGCGTCTCGCTGCTGGTCGCGGGCTGGGACGCACACCGG
GGCCCGAGCCTTTACCAGGTGGACCCGTCCGGCTCCTTCTGGGCGTGGAAGGCCAGC
GCTATCGGCAAGAACATGGTGAACGCGAAGACGTTCTTGGAGAAG

> Pre8 (MS429)_*Stereum hirsutum*
ACGCAATCAGGGTACCCACTCTCCGAGTCTTTGAGGTGGTGTAATGGTGCTGACTCGC
AGTGGTGTCCGGCCTTATGGGGTTTCTTTGCTCGTTGCCGGTTTCGATACGAACCGAGG
TCCAACTCTATACCAGGTTGATCCATCAGGATCCTTTTGGGCCTGGAAGGCCAGCGCGA
TAGGAAAGAATATGACCAACGCAAAGACGTTTTTGGAGAAG

> Pre8 (MS429)_*Trametes versicolor*
ACACATTCCGGGTACGTCTTCTGTGTTGCCTTTCACAGGCGGTCTGATAATTTGAAACA
GCGGAGTGCGGCCATACGGCGTATCCCTCTTGGTTGCGGGATGGGACATAACCCGAGG
GCCGTGCTTGTACCAGGTTGACCCTTCAGGTTCGTATTGGGCGTGGAAGGCGAGCGCG
ATCGGAAAGAACATGGTAAACGCGAAGACGTTCTTGGAGAAG

> Pre8 (MS429)_*Serpula lacrymans* var. *lacrymans*
ACGCAATCCGGGTGTGTTTTGACCTTGATCGCTTTAATGTTGACCGAGTGTGTGATTTA
GAGGTGTGCGACCGTTTGGTGTATCGCTGTTGGTAGCAGGATGGGATAACAACCGGGG
GCCTAGTCTGTACCAAGTAGATCCATCTGGCTCATTCTGGGCCTGGAAAGCCAGTGCTA
TTGGCAAGAACATGACAAACGCGAAGACATTCTTAGAGAAA

> Pre8 (MS429)_*Rhizopus microsporus*
ACTCAAAGCGGGTAAGCGAGAAAGGAAAAAAGAATTTTGACCACTTAATCATTCATAT
AGTGGTGTTCGTCCATTTGGTGTTTCTATTTTGATTGCAGGATATGATGAGGTGAACGG
ACCAGCACTCTATCAGGTCGATCCCACTGGATCTTACTTTGCATGGAAGGCCAGTGCTA
TTGGTAAGAACATGATTAATGCAAAGACGTTCTTGGAAAAG

> Pre8 (MS429)_*Neurospora crassa*
ACGCAGTCCGGAGGTGTCCGCCCCTATGGTGTCTCGCTGCTGATCGCCGGCTGGGACG
TGAAGGGCGGCCCCATGCTGTACCAGGTCGACCCCTCGGGCAGTTATTTCCCTTGGAA
GGCGACGGCCATTGGCAAGAACGCCACGACGGCCAAGACCTTTTTGGAGAAG

> Pre8 (MS429)_*Asperigillus triticus*
ACCCAATCCGGTGGTGTGCGGCCGTACGGTGTCAGTTTGCTGATCGCGGGGTGGGACG
TCAAGGGCGGCCCGAGTCTCTACCAGGTTGACCCCAGCGGCAGTTACTACCCGTGGAA
GGCGACGGCCATCGGCAGACACGCGACGAGCGCCAAGACGTTCCTGGAGAAG

>Crm1 (MS442)_*Amanita muscaria*
ATGGTCAAGCCAGAAGAGGTGAGCGGAAATTCATATGGTGCACGATCAAAGGTTCTTA
TTGTGGAAAACGATGAAGGAGAAATTGTCAGAGAATTCATGAAAGAAAGCGACACCA
TTGTTCTATACAAGTCCATGCGACAGTTACTCGTTTATCTAACTCATCTGGATGTCCAAG
ATACCGAGAACATCTTGACCGAGAAGCTCGCCAAACAAGTTGACGGTACAGAGTGGT
CTTGGAACAATCTGAACACACTGTGTTGGGCTATAGGATCGATTTCTGGAGCAATGA

> Crm1 (MS442)_*Agrocybe pediades*
ATGGTGAAGCCGGAGGAGGTTAGTTGTCTTCTCTATCCTTTTTCGTGTCAGGTTCTAATT
GTTGAGAATGACGAAGGTGAAATCGTTCGTGAATTCATGAAGGAGAGCGACACCATCG
TCTTGTACAAGTCGATGCGCGAACTACTAGTTTACCTTACCCATCTTGACGTGGGTGAC
ACTGAAACCATCCTTACGGAGAAGTTGGCCAAGCAAGTGGATGGGTCCGAGTGGTCG
TGGAACAACTTAAACACTCTTTGCTGGGCTATCGGTTCAATTTCTGGAGCTATGA

> Crm1 (MS442)_*Gymnopilus chrysopellus*
ATGGTGAAGCCTGAGGAAGTAAGTTTCTTTGAACTTGACGCGCCGGTCTAGGTTCTTAT
CGTGGAGAACGACGAGGGTGAAATCGTCCGTGAATTCATGAAGGAGATCGATACAATC
GTTTTGTACAAGTCTATGCGCGAATTACTCGTGTACCTCACGCATCTTGACGTGCAGGA
TACCGAGACAATTCTCACAGAGAAGTTGGCAAAGCAGGTCGATGGATCAGAATGGTCA
TGGAACAATCTCAATACCCTTTGCTGGGCCATTGGCTCTATCTCCGGTGCTATGA

> Crm1 (MS442)_*Laccaria bicolor*
ATGGTCAAGCCAGAGGAGGTTAGTCGTTTCAATATTGGTTTTCGACTTCAGGTCCTGAT
TGTTGAGAATGACGAGGGGGAGATCGTCCGAGAGTTCATGAAGGAGATCGATACCATT
GTACTGTATAAGTCAATGCGTGAACTTCTGGTCTACCTCACCCATCTCGACGTCTCAGA
CACAGAAACTATCCTCACAGAGAAACTTGCGAAGCAGGTCGATGGGTCTGAATGGTCG
TGGAACAACCTCAATACCCTTTGCTGGGCTATCGGCTCTATCTCGGGTGCTATGA

> Crm1 (MS442)_*Coprinopsis cinerea*
ATGGTGAAGCCCGAGGAGGTGTGTCACAAAGGGAACTATGCATCATTCTAGGTCTTGA
TCGTTGAGAACGACGAAGGTGAAATTGTTCGCGAGTTCATGAAGGAGAGTGACACCA
TCGTGCTCTACAAGGCCATGAGGGAGCTCCTTGTTTATCTTACCCACTTGGACGTCAAC
GATACCGAAACCATCTTGACGGAAAAGCTGGCCAAGCAAGTGGACGGATCTGAATGG
TCATGGAACAACTTGAACACCCTGTGTTGGGCGATTGGTTCCATTTCCGGCGCCATGA

> Crm1 (MS442)_*Phanerochaete carnosa*
ATGGTGAAGCCTGAGGAGGTGCGCCATCCGCCACAGACTATTTGGACAAAGGTTCTCA
TCGTCGAGAACGAGGAGGGAGAGATTGTACGTGAGTTCATGAAGGAGAGTGACACCA

TTGTCCTCTACAAGCAGATGCGCGAACTACTTGTGTACCTCACCCACCTGGATGTTCAA
GACACCGAGACCATTCTCACGGAGAAGCTTGCCAAGCAAGTGGACGGCTCCGAATGG
TCGTGGCAGAACCTCAATACTCTGTGCTGGGCTATTGGATCCATATCCGGCGCCATGA

> Crm1 (MS442)_*Fomitopsis pinicola*
ATGGTCAAACCCGAGGAGGTAGGTGGCCGGTCGTTCTATACGACAGCGTAGGTGCTCA
TCGTCGAGAACGAGGAGGGCGAGATCGTCCGCGAGTTCATGAAGGAGAGCGACACCA
TCGTTCTCTACAAGTCAATGCGCGAACTGCTTGTCTACCTCACCCATCTCGACGTCAAC
GACACGGAGAACATCCTTACGGAGAAGCTTGCTAAGCAAGTTGATGGGTCGGAGTGG
TCCTGGCAGAACCTCAACACGCTATGCTGGGCTATAGGCTCGATTTCTGGTGCCATGA

> Crm1 (MS442)_*Schizophyllum commune*
ATGGTCAAGCCCGAAGAGGTTCAGCCACCATCCTGCTGCTTTTCATGCCAGGTTCTCGT
TGTCGAGAACGACGAGGGTGAGATCGTTCGTGAGTTCATGAAGGAGAGCGACACCAT
CGTGCTATACAAGTCTATGCGCGAGTTGCTGGTTTACCTTACTCACTTGGACGTCAATG
ACACGGAGACCATCCTCACGGACAAGCTCGCAAAGCAGGTCGACGGCTCAGAATGGT
CATGGAACAACCTCAACACGCTATGCTGGGCTATTGGCTCGATATCGGGCGCCATGA

> Crm1 (MS442)_*Auricularia delicata*
ATGGTCAAGCCCGAGGAGGTGCTCGTCGTCGAGAACGACGAGGGCGAGATCGTGCGC
GAGTTCATGCGCGAGAGCGACACGATCATGTTGTACAAGTCCCTGCGCGAGTGCCTTG
TCTACCTCACACACTTGGACGTCCAGGACACCGAGGCTATCCTCACGGAAAAGCTCTC
AAAGCAGATTGACGGTACCGAGTGGTCCTGGAACAACATCAACAGACTCTGCTGGGC
GATTGGCAGCATCTCCGGCGCGATGA

> Crm1 (MS442)_*Stereum hirsutum*
ATGGTCAAGCCTGAAGAGGTGAGTGTTTAATATGTCTCCTCGTGATTCTAGGTTCTGGT
CGTTGAGAACGAAGAAGGAGAGATTGTTCGAGAGTTCATGAAGGAGTCGGACACCAT
CGTGCTCTACAAGTCTATGCGCGAACTTCTCGTTTACCTCACCCATCTGGACGTCTCGG
ATACGGAGACGATCCTCACAAGTAAGCTGGCGAAACAAGTCGACGGCGCGGAGTGGT
CGTGGGGAAACCTTAACACTCTTTGCTGGGCCATTGGTTCGATATCTGGTGCCATGA

> Crm1 (MS442)_*Trametes versicolor*
ATGGTCAAGCCCGAAGAGGTGCGTTCAATCCCGCTGAGTTTGTGACTACAGGTCCTCA
TTGTCGAGAATGAGGAGGGCGAGATCGTCCGTGAGTTCATGAAGGAGAGCGATACCAT
CGTGCTGTACAAGTCGATGCGTGAGCTCCTCGTGTACCTCACTCACCTGGACGTCAAC
GACACCGAGAACATTCTTACCGAGAAGCTGCAGAAGCAGGTCGACGGCTCCGAGTGG
TCGTGGCAGAACCTCAACACGCTGTGCTGGGCAATCGGTTCTATCTCGGGAGCGATGA

> Crm1 (MS442)_*Lactarius quietus*
ATGGTGAAGCCTGAAGAGGTTCGCATTCTTTCCCTTCATTCATTGCTGCAGGTCCTCAT
TGTCGAGAACGATGAGGGTGAAATTGTTCGTGAATTCATGAAAGAGTCGGACACCATC
GTCCTTTACAAATCAATGCGCGAGCTTTTGGTCTATCTCACCCATCTAGACGTCTCCGAT
ACCGAAAACATTCTTACGGAGAAGCTTGCGAAACAAGTCGACGGCTCAGAATGGTCG

TGGCAAAATTTAAACACATTGTGTTGGGCCATTGGGTCAATTTCAGGGGCGATGA

> Crm1 (MS442)_*Pluteus cervinus*
ATGGTGAAGCCTGAAGAGGTATGGTTTCCACGTACTCATCCAGGTACTCATCGTCGAAA
ACGATGAGGGTGAAATTGTTCGCGATTTCCTGAAGGAAATCGATACAATCGTCCTCTAC
AAATCCATGCGCGAACTTTTGGTTTATCTTACCCATCTTGACGTTAATGACACGGAAAC
CATCCTAAACGAGAAGCTTGCGAAACAAGTAGACGGTTCTGAATGGTCGTGGAATAAC
TTGAACACCCTCTGCTGGGCTATTGGTTCGATCTCTGGCGCCATGA

> Crm1 (MS442)_*Mycena crocata*
ATGGTAAAACCTGAGGAGGTGCGTTTCTATGTTCTGTGTTCATTAGACAAGGTTTTGAT
CGTGGAAAATGACGAGGGTGAAATCGTGCGCGAATTCATGAAGGAAAGCGACACTAT
CGTCTTGTACAAGTCGATGCGAGAGCTGCTTGTATATCTTACCCATCTGGACGTGTCAG
ATACAGAAAACATACTTACAGAGAAGCTTGCGAAACAAGTGGACGGGTCGGAATGGT
CATGGAACAATCTGAACACGCTATGCTGGGCTATCGGTTCGATATCAGGAGCCATGA

> Crm1 (MS442)_*Tricholoma matsutake*
ACGGTTAAGCCCGAAGAGGTGCGTGCTGGTTCTATGTGGAGATGAATATAGGTGCTCAT
TGTAGAGAACGAGGAAGGAGAGATCGTCCGAGAATTCATGAAAGAGAGCGACACAAT
AGTGCTATACAAGTCTATGCGCGAGCTGCTTGTCTATCTTACGCATCTTGACGTGGTAG
ACACGGAGACGATACTAACGGAGAAGCTCGCGAACCAGGTCGACGGCTCAGAGTGGT
CATGGAACAACGTGAACACGCTCTGCTGGGCTATCGGCTCCATATCTGGCGCTATGA

> Crm1 (MS442)_*Gymnopus androsaceus*
ATGGTGAAGCCGGAGGAGGTGACTATGATTCGATTCTATTCAGGTGCTCATTGTCGAGA
ACGAAGAAGGAGAAATCGTTCGAGAGTTCATGAAAGAGAGTGATACTATTGTCCTCTA
CAAGTCCATGCGCGAACTTTTGGTCTATCTTACGCATCTGGACGTGGCTGATACTGAGA
CTATCCTCACCGAAAAGCTCGCAAAACAAGTCGATGGCTCCGAATGGTCATGGAACAA
CCTCAATACCCTTTGTTGGGCAATTGGTTCCATATCTGGCGCCATGA

> Crm1 (MS442)_*Marasmius fiardii*
ATGGTGAAGCCAGAAGAGGTGAGATTCGGAATAGCTTTTATTTCATTTCAGGTTTTGAT
TGTCGAAAACGAGGAAGGAGAAATTGTCCGCGAATACCTGAAGGAGACCGATACTATC
GTTCTGTACAAGTCTATGCGTGAATTGCTTGTGTACCTTACCCACTTGGATGTTTCCGAT
ACCGAGAACATCCTTACCGAAAAGCTGTCCAAACAGGTCGATGGATCTGAATGGTCTT
GGCAGAATCTCAACACTCTTTGCTGGGCAATAGGTTCAATATCCGGTGCAATGA

> Crm1 (MS442)_*Boletus edulis*
ATGGTCAAACCAGAAGAGGTGAGGGCCTCCGTTTATTCGACAGGTTCTCATCGTTGAA
AATGATGAAGGCGAAATTGTCCGGGAGTTCATGAAGGAGTCCGATACCATCGTTCTCTA
CAAGCAAATGCGTGAACTTCTGGTATATCTCACCCACCTAGACGTCGTTGACACAGAA
AACATCCTGACGGAAAAGTTGGCCAAGCAAGTTGACGGCTCGGAATGGAGCTGGCAA
AACTTGAACACACTCTGCTGGGCCATTGGTAGTATCTCCGGAGCCATGA

> Crm1 (MS442)_*Hygrophoropsis aurantiaca*
ATGGTGAAACCTGAGGAAGTGAGTGTCATAAATCATTGGAAATAAATCTAGGTCCTCAT
TGTCGAAAATGACGAAGGCGAGATTGTCCGCGAGTTCATGAAGGAGTCGGATACTATC
GTGCTCTACAAGCAAATGCGTGAACTTCTGGTTTACCTCACACATCTGGACGTCGTGGA
TACGGAAAATATCCTTACTGAAAAACTCGCAAAGCAAGTCGATGGTTCCGAATGGAGT
TGGCAAAATCTCAACACTCTTTGCTGGGCTATCGGCAGTATTTCTGGCGCTATGA

> Crm1 (MS442)_*Fomitiporia mediterranea*
ATGGTCAAGCCGGAAGAGGTAGTCCAGTCTTTCGCCTGTAGAAGACTCCAGGTCCTCG
TCGTGGAGAATGACGAAGGTGAAGTTGTCCGCGAGTTCATGAAGGAGAGCGATACCAT
TGTCCTCTATAAGTCTATGCGTGAACTTCTCGTGTATCTGACACATCTGGACGTTCTCGA
CACGGAAAATATCCTGACGGAGAAGCTTGCAAAGCAAGTCGACGGTTCCGAATGGTC
GTGGAATAACCTCAATACACTGTGCTGGGCCATCGGTTCTATTTCTGGAGCTATGA

> Crm1 (MS442)_*Agaricus bisporus* var. *burnettii*
ATGGTGAAACCAGAGGAGGTGCGTACTTATCGACAGCACTTGTTTCATCAGGTCTTAAT
CGTCGAAAATGAAGAGGGAGAGATTGTGCGTGAATTCATGAAGGAGGGCGATACAATC
TCTCTTTACAAGTCCATGAGGGAGTTACTCGTTTACCTGACGCACTTGGACGTTACGGA
CACAGAGACTATTCTCACTGAAAAACTTGCCAGACAGGTTGATGGATCTGAATGGTCT
TGGAATAACCTCAATACTCTCTGTTGGGCCATCGGTTCGATTTCAGGTGCCATGA

> Crm1 (MS442)_*Serpula lacrymans* var.*lacrymans*
ATGGTCAAACCAGAGGAGGTATGTGTGTTCATATTTAATTTTTTGTTCTAGGTTTTGATT
GTCGAAAACGAAGAAGGAGAAATCGTGCGTGAATTCATGAAAGAAAGCGATACCATT
GTCCTTTACAAGTCGATGCGCGAACTTTTGGTGTACCTTACACATTTGGACGTCGTTGA
TACTGAGAATATTCTCACCGAAAAGCTGGCGAAGCAAGTCGATGGTTCAGAGTGGTCT
TGGCAGAATCTAAACACACTATGCTGGGCTATTGGTTCTATCTCTGGAGCGATGA

> Crm1 (MS442)_*Dacryopinax primogenitus*
ATGGTCAAGCCGGAGGAAGTTCTCATCGTAGAGAACGAAGAAGGCGAAATCGTCCGC
GAAGTCATGAAGGAGACGGACACCATTGCACTGTATAAATCAATTCGAGAGGTGCTCA
TCTACCTCACTCACTTGGACGTCATGGATACCGAGAATGTCCTGACCGAGAAGCTCGC
GAAGCAGGTAGATGGTAGCGAGTGGTCGTGGAACAATCTGAATACACTCTGCTGGGCT
ATAGGCTCTATCTCTGGATCGATGG

> Crm1 (MS442)_*Calocera cornea*
ATGGTCAAGCCCGAAGAGGTTCTCATTGTTGAGAACGAGGAGGGAGAGATCGTCCGC
GAGGTTATGAAGGAGACGGACACGATCGTCCTCTACAAGTCGATTCGGGAGGTGCTCA
TCTACCTCACGCACCTGGACGTTATGGACACCGAGAATGTCCTCACCGAAAAACTTGC
AAAGCAGGTGGATGGCAGCGAGTGGTCGTGGAACAACTTGAACACGCTCTGCTGGGC
TATCGGTTCCATCTCTGGGTCCATGG
> Crm1 (MS442)_*Agaricostilbum hyphaenes*
ATGGTCAAGCCTGAAGAAGTGCTGGTCGTGGAGAATGACGAGGGCGAGATTGTGCGA
GAATTCATGAAAGAGATTGACACCATTGTCCTGTACAAGCAGATGCGTGAAGTGCTGG

TCTACCTCACCCACTTGGACGTGCAAGACACGGAGATTATCATGACGGATAAGCTGGC
CAAGCAAGTCGATGGCAGTGAATGGTCATGGGCC
AACTGCAACACTCTATGCTGGGCCATTGGGTCCATCTCGGGTGCTATGA

> Crm1 (MS442)_*Neurospora crassa*
ATGGTGCGCCCCGAAGAAGTCCTCATTGTTGAGAACGACGAGGGCGAGATTGTCCGC
GAGTTTGTCAAGGAAACCGACACTGTCCAGCTTTACAAGACCATCCGCGAGTGTCTCG
TTTATCTCACCCATTTGGATGTGGTCGACACCGAGCAGATTATGACCGACAAGCTCGCA
CGCCAAGTCGATGGATCCGAATGGTCATGGCACAACTGCAACGTTCTCTGCTGGGCTAT
TGGTTCTATTTCTCTCGCCATGA

> Crm1 (MS442)_*Asperigillus triticus*
ATGGTACGCCCGGAAGAGGTCTTGATCGTCGAGAATGACGAGGGAGAGATCGTTCGCG
AGTTCGTCAAAGAAAGCGATACTATCCAGCTGTATAAGACGATACGCGAATGTTTGGTT
TATCTTACCCATCTCGATGTTGTTGACACGGAGAACATCATGATCGAGAAGCTAGCCAA
GCAGGTTGACGGATCCGAATGGTCGTGGGTTAATTGCAACACACTTTGTTGGGCAATC
GGATCGATCTCGGGTGCCATGA

> Crm1 (MS442)_*Rhizopus microsporus*
ATGGTTAAGCCAGAAGAAGTAAAGAGATTTATATAGAACACATATAATTAGGTATTGATT
GTCGAAAATGATGAAGGTGAAATTGTCAGAGAATTTGTAAAGGAAAGTGATACTATTG
TGTTATACAAGAGTATGAAGCAAGTCTTGGTGTACTTGACTCATCTTAACGTTGAAGAT
ACGGTGAACATCATGACAGTTAAATTGACAAAAAAGATGGATGGAACTGAATGGTCTT
GGAACAATTTGAATAAATTGTGTTGGGCAATTGGTTCGATCTCTGGTGCAATGG

>Cdc47 (MS456)_*Agaricus bisporus* var. *bisporus*
GCCAAGTCCCAACTTCTCAAATTTATGTCAAAAATCGCTCCTCGAGGGGTTTACACTAC
TGGGAAAGGATCATCCGGAGTTGGCCTTACGGCCGCTGTCATGCGAGATCCGGTCACC
GATGAGATGGTTTTAGGTACGTTTGTCAAGATTATGCCGCAAGTTCATTTAGCTAAAATT
AGGACAGAGGGTGGCGCTCTTGTCCTTGCTGACAATGGCATCTGTTGTATTGACGAATT
TGACAAGA

> Cdc47 (MS456)_*Schizophyllum commune*
GCCAAGTCGCAGCTGCTGAAGTATATCTCGAAGGTCGCGCCTCGTGGTGTATACACCA
CGGGCAAGGGTTCGTCAGGCGTTGGTCTCACTGCTGCAGTTATGCGCGACCCGGTAAC
GGACGAGATGGTGTTGGGTATGTGCTTCTCACCTTTGCCTTTACCACTGACGTCGCTGA
CATTGTCGTAGAGGGTGGCGCGCTCGTGCTCGCGGACAATGGCATCTGCTGCATTGATG
AATTCGACAAAA

> Cdc47 (MS456)_*Fomitopsis pinicola*
GCAAAATCTCAGTTGCTCAAGTATATCTCCAAGGTCGCGCCGCGTGGCGTGTATACAAC
CGGCAAAGGTTCATCAGGCGTTGGTCTCACTGCAGCAGTGATGCGAGACCCTGTTACG
GATGAGATGGTACTCGGTAAGGACTGCGCTGCGTTTTCTGTGATCATGGCTGACTTCAT
GGCAGAGGGTGGCGCACTCGTGCTTGCTGACAACGGCATTTGCTGCATTGATGAGTTC

GATAAGA

> Cdc47 (MS456)_*Coprinopsis cinerea*
GCCAAATCGCAGTTACTCAAGTATATCTCCAAGATTGCTCCTCGAGGAGTCTACACCAC
AGGCAAGGGCTCCTCGGGCGTTGGACTCACTGCTGCTGTCATGCGGGATCCTGTGACG
GATGAGATGGTCCTCGGTAAGCATTTCCGATTGTTACACTGACTGCTTCCAACTTACTC
CTGTCAACAGAGGGTGGTGCTCTCGTCCTCGCAGACAACGGCATTTGCTGCATTGACG
AATTTGACAAAA

> Cdc47 (MS456)_*Auricularia delicata*
GCTAAGTCGCAGTTGCTCAAGTACATCTCCAAGGTCGCTCCCCGTGGCGTGTATACGAC
GGGCAAGGGTTCTTCGGGCGTCGGTCTTACCGCTGCTGTCATGAAGGACCCAGTCACG
GAGGAGATGGTGCTTGGTGAGTTGTTTCAATTCAACTGCTTTGCACCTGCTCATTGTTG
GTAGAGGGTGGTGCGCTTGTTCTGGCCGACAACGGTATCTGCTGCATCGATGAATTCG
ACAAGA

> Cdc47 (MS456)_*Lactarius quietus*
GCGAAATCGCAGTTGCTGAAGTATATATCAAAAGTGGCTCCTCGAGGTGTCTACACAA
CCGGAAAGGGTTCATCCGGCGTTGGTCTAACAGCCGCTGTCATGCGGGACCCGGTGAC
AGATGAGATGGTTCTCGGTGCGTTTCTCATTGTCAGATATCTTATTCAGTTGCTGACAAT
GTTGCAGAGGGCGGCGCGCTCGTCTTGGCAGACAATGGTATATGCTGTATCGATGAGTT
CGACAAGA

> Cdc47 (MS456)_*Phanerochaete carnosa*
GCAAAGTCTCAGCTACTGAAGTACATCTCAAAGGTCGCCCCGAGAGGTATTTACACCA
CTGGAAAAGGCTCTTCCGGCGTTGGTCTCACCGCAGCCGTCATGCGGGATCCCGTCAC
GGATGAGATGGTCCTAGGTAAGCATCTTCATTCTTCAGCTTAAACGTCTACTCACATGG
ACATGCAGAGGGAGGTGCACTTGTTCTGGCGGACAACGGAATTTGCTGCATTGACGAG
TTCGACAAGA

> Cdc47 (MS456)_*Calocera cornea*
GCCAAGTCCCAGCTGCTCAAGTACATTTCGAAAGTCGCCCCAAGAGGCATCTATACCA
CCGGCAAGGGTTCTTCCGGTGTCGGTCTTACTGCTGCGGTGATGCGTGATCCTGTTACG
GATGAGATGGTCCTTGGTACGTACTCCGTCCAGCTTCATGTAGCAGCGTGCTCAAGGAT
CATCAAGAGGGCGGTGCACTCGTCCTTGCGGACAACGGTATTTGCTGCATCGACGAGT
TCGACAAGA

> Cdc47 (MS456)_*Dacryopinax primogenitus*
GCCAAGTCGCAGCTCTTGAAATATATCTCGAAGGTGGCACCGAGAGGTGTCTACACCA
CCGGGAAAGGTTCTTCTGGTGTTGGTCTTACTGCAGCAGTAATGCGTGATCCGGTGAC
TGACGAAATGGTCCTTGGTAAGCCCCACACATCTGTAGGGGATCAATGTCCTCATTTCC
ACCTCAGAGGGCGGTGCCCTTGTTCTCGCCGATAATGGCATTTGTTGCATCGATGAATT
CGACAAGA

> Cdc47 (MS456)_*Tricholoma matsutake*
GCCAAGTCCCAACTGCTCAAGTACATTTCGAAGATTGCTCCTCGAGGGGTTTATACAAC
TGGAAAGGGGTCTTCTGGTGTGGGTCTTACCGCCGCTGTCATGAGGGATCCTGTCACC
GATGAGATGGTTTTGGGTCAGTCCTTCTGTCACGTTCCAGTTGATACATTTGTGATTGTG
AAACTCGACAGAGGGAGGAGCGCTTGTTCTTGCTGACAATGGCATATGTTGCATCGAC
GAATTCGACAAGA

> Cdc47 (MS456)_*Stereum hirsutum*
GCCAAATCTCAGTTGTTGAAGTATATTTCGAAGGTTGCTCCTCGAGGGGTGTACACGAC
GGGCAAGGGTTCGTCGGGAGTCGGTCTGACTGCCGCTGTCATGCGCGATCCAGTAACA
GACGAGATGGTTCTTGGTGCGTTTTCTTACTGGACTTAATCGCGACTGATGGCTAACGA
AGGGATTTTCAGAGGGAGGCGCACTTGTTCTCGCAGATAATGGTATTTGCTGTATAGAC
GAGTTTGACAAGA

> Cdc47 (MS456)_*Trametes versicolor*
GCCAAGTCGCAGCTCCTCAAGTACATCACCAAGGTCGCTCCTCGCGGAGTGTATACCA
CCGGTCGTGGTTCGTCGGGCGTCGGCTTGACTGCTGCCGTCATGCGCGACCCAGTTAC
CGACGAGATGGTTCTTGGTACGTTCGTCTTCACGACACATTTGTAGATGGCCACTGACG
TCTTTGGGGCAAAAGAGGGCGGCGCTCTCGTTCTTGCTGACAACGGCATCTGTTGTAT
CGATGAGTTCGACAAGA

> Cdc47 (MS456)_*Mycena crocata*
GCTAAATCTCAATTACTCAAATATATCTCCAAAATTGCGCCTCGCGGTGTTTACACTACT
GGAAAAGGTTCTTCGGGAGTCGGTCTTACAGCCGCCGTGATGAGAGATCCCGTCACGG
ACGAGATGGTGCTGGGTACGTTTCTCTTTTCGGTAACCTCGAACAGCAACTGAATATTG
GATACAGAGGGCGGAGCCCTCGTTCTCGCTGACAATGGTATCTGCTGTATCGACGAGTT
CGATAAAA

> Cdc47 (MS456)_*Laccaria bicolor*
GCGAAATCGCAACTGCTGAAATATATATCCAAAATCGCTCCTCGAGGTGTTTATACCAC
CGGAAAGGGATCGTCAGGAGTCGGTCTTACCGCTGCCGTTATGCGGGATCCCGTCACG
GACGAGATGGTGCTAGGTACGTGAATCTATTTCAATCATTCCAACAATCTAATATCTTTT
GAACCTCAGAGGGTGGAGCATTGGTCCTTGCCGATAACGGAATTTGCTGCATTGATGA
ATTCGACAAGA

> Cdc47 (MS456)_*Pluteus cervinus*
GCCAAGTCGCAGTTGTTGAAGTATATTTCGAAAATTGCACCGAGAGGCGTCTACACCA
CTGGAAAGGGTTCATCAGGCGTTGGTTTGACGGCCGCCGTCATGCGAGACCCTGTCAC
GGATGAGATGGTCCTCGGTATGTTTCATCAGGAACTAAAGCTCGACTTATTTCTGACGA
CACATCTAGAGGGTGGTGCTCTCGTCCTTGCGGATAACGGCATATGCTGTATCGATGAG
TTCGACAAGA
> Cdc47 (MS456)_*Serpula lacrymans* var. *lacrymans*
GCCAAATCTCAATTGCTCAAATACATCTCGAAAATCGCACCCAGAGGAGTATATACCAC
CGGTAAAGGTTCTTCAGGGGTTGGTTTGACCGCAGCAGTCATGCGTGATCCTGTCACG

GACGAGATGGTATTAGGTATGGCATTTTCTATCAGCAACCAGTTCATCATACTTATTAAA
TTTGTTAGAGGGAGGTGCTCTCGTCCTGGCCGATAACGGAATCTGTTGTATTGATGAAT
TCGATAAAA

> Cdc47 (MS456)_*Gymnopilus chrysopellus*
GCGAAATCTCAATTATTGAAATATATCTCGAAAATTGCACCCAGAGGTGTCTACACAAC
TGGCAAAGGCTCGTCGGGTGTCGGTTTGACGGCCGCTGTGATGCGCGACCCTGTTACA
GATGAAATGGTGCTAGGTTTGTAGCCCCCTCTAAGACTTCTGATCATACACTAATTCTAC
TTCCAGAGGGTGGTGCTCTTGTTCTTGCTGACAATGGTATCTGTTGTATCGACGAGTTC
GACAAAA

> Cdc47 (MS456)_*Gymnopus androsaceus*
GCTAAATCGCAGTTGCTGAAGTATATCTCGAAGATTGCCCCTCGCGGAGTCTACACTAC
CGGAAAAGGTTCTTCTGGTGTTGGGCTGACCGCAGCGGTTATGCGAGACCCTGTAACC
GACGAGATGGTCTTGGGTTTGTGCTTACCAGTCGTCTGGCTCTCGGATAATACTGATGT
TCGATTGCAGAGGGTGGTGCACTCGTACTAGCCGATAACGGTATCTGCTGCATCGATGA
ATTCGATAAGA

> Cdc47 (MS456)_*Agrocybe pediades*
GCCAAGTCCCAGCTACTGAAATATATCTCAAAAGTTGCACCGAGAGGCGTTTATACCAC
TGGAAAAGGTTCCTCAGGCGTCGGTTTGACAGCTGCCGTCATGCGAGATCCAGTCACG
GACGAGATGGTATTAGGTGAGTTCGACGGAGGGTCATACTATAATAACATGTTTGACAT
TTTCTTTTAGAGGGAGGAGCCCTCGTACTTGCAGACAACGGTATTTGCTGCATCGACGA
ATTTGACAAAA

> Cdc47 (MS456)_*Hygrophoropsis aurantiaca*
GCTAAATCACAGCTTCTGAAGTATATATCCAAAATCGCACCACGAGGCGTGTACACCAC
TGGAAAAGGTTCTTCGGGCGTAGGTTTAACTGCCGCTGTCATGCGAGATCCCGTGACG
GACGAGATGGTACTAGGTGCGTCCGCGTCCGCGATCGATGCCCTGAACCTCCACTCCT
AACCATGCATCTGTTGCATTCCTCAGAGGGCGGCGCACTAGTCCTAGCTGACAACGGC
ATATGCTGTATCGACGAGTTCGACAAGA

> Cdc47 (MS456)_*Boletus edulis*
GCCAAGTCACAACTGCTCAAGTATATCTCCAAGGTTGCCCCGCGTGGTGTGTACACCA
CGGGTAAGGGTTCCTCCGGCGTTGGTCTCACAGCTGCTGTCATGCGAGATCCGGTCAC
GGACGAAATGGTTCTTGGTAGGTAATTTGGCCTATGAAGTATACATATTGACGTGAATG
AGTTCAGAGGGTGGCGCTCTTGTGTTGGCTGACAATGGCATCTGTTGCATTGATGAGTT
CGACAAAA

> Cdc47 (MS456)_*Gautieria morchelliformis*
GCAAAGTCGCAGCTGCTCAAGTATATTTCCAAGGTGGCTCCTCGTGGAGTTTACACGA
CAGGAAAAGGCTCGTCTGGCGTTGGTCTGACGGCCGCTGTCATGCGAGATCCTGTGAC
GGATGAAATGGTCTTGGGTACGTTCTCAGCTATTGCCCGACTCTTGAATTGACCTCTCG
ACAGAGGGCGGAGCACTGGTTCTGGCCGATAACGGAATATGTTGCATTGATGAGTTCG

ATAAGA

> Cdc47 (MS456)_*Ramaria acris*
GCTAAGTCTCAGCTGCTGAAGTATATTTCAAAAGTGGCACCCCGTGGAGTATATACTAC
AGGCAAGGGCTCATCTGGCGTTGGTTTGACGGCTGCAGTGATGCGGGACCCGGTGAC
GGACGAAATGGTTTTGGGTGCGTCCCTAGAGCGGTACGGATTGTCTCACTGATCAACA
TTCTGGTAGAGGGTGGAGCACTGGTCCTTGCCGATAATGGAATCTGCTGCATTGATGAG
TTTGACAAAA

> Cdc47 (MS456)_*Amanita muscaria*
GCAAAGTCGCAGTTGCTCAAGTATATTTCCAAGGTTGCTCCACGTGGTATTTACACGAC
AGGAAAGGGTTCATCTGGCGTAGGATTGACAGCCGCTGTCATGCGCGATCCTGTCACG
GACGAGATGGTCCTAGGTTAGCTTCGCACTATTCCATGGCTAACTGCAAGGCTGACAA
GGCTTAGAGGGAGGCGCATTGGTTCTGGCTGACAACGGAATATGTTGTATAGACGAATT
CGACAAGA

> Cdc47 (MS456)_*Marasmius fiardii*
GCCAAGTCTCAACTTCTTAAGTATATATCGAAAATCGCACCTCGTGGAGTGTACACCAC
AGGAAAGGGTTCGTCTGGGGTCGGTTTGACGGCTGCGGTTATGAGAGACCCGGTTACA
GATGAGATGGTGCTCGGTGAGTACTATATCCAGTTATTGGAAAACCACTTCGGCTTACA
AGCAACCTAGAGGGCGGCGCCCTCGTACTCGCCGACAACGGAATCTGTTGCATCGATG
AGTTCGATAAGA

> Cdc47 (MS456)_*Rhizopus microsporus*
GCCAAATCCCAGTTATTAAAGTTTATTGCCAAGGTAGCACCCAGAGGTGTATACACGAC
CGGTAAAGGTTCATCTGGTGTAGGTTTAACAGCTGCTGTGATGAAAGACCCTGTGACA
GATGAGATGGTGTTGGAAGGAGGTGCTCTTGTCTTGGCTGATAATGGTGTTTGCTGTAT
TGATGAATTTGACAAGA

>Pdb1 (FG855)_*Agrocybe pediades*
CAAGCCATTGATCAAATTGTAAACTCTGCTGGAAAGACATATTACATGTCCGGTGGAAA
CGTTCCTTGCCCAGTTGTGTTCCGTGGTCCCAACGGCGCCGCCGCAGGTGTTGCTGCT
CAGCATTCTCAAGACTACGCTGCATGGTACGGTTCAGTTCCTGGTCTCAAGGTTGTCAG
CCCGTGGAGCGCAGAAGACTGCAAAGGTCTTCTGAAGGTACGTTGACGCTGTCCTTAC
TGCATTTATTGACTCATCATCTAAACTAGTCTGCCATCCGC

> Pdb1 (FG855)_*Schizophyllum commune*
CAGGCCATTGACCAGATCGTCAACTCCGCCGGCAAGACGTACTACATGTCTGGTGGCA
ACGTGCCCTGCCCGGTCGTCTTCCGTGGCCCCAACGGTGCCGCTGCAGGTGTCGCCGC
GCAGCACTCGCAGGACTACGCATCGTGGTACGGCCAGGTGCCTGGCTTGAAGGTCGTT
TCCCCGTGGAGTGCGGAGGACTGCAAGGGCTTGTTGAAGGTAGGCATCACCTTCCATG
TTGTACCACTTCTGCATGGCACCTCTGTTAGTCTGCCATCCGT

> Pdb1 (FG855)_*Mycena crocata*

CAGGCGATTGACCAAATCGTCAACTCGGCCGGCAAGACGTACTACATGTCGGGCGGAA
ATGTACCCTGCCCAGTCGTTTTCCGCGGTCCTAACGGTGCCGCCGCTGGTGTCGCGGC
ACAGCATTCGCAGGATTACGCTGCTTGGTACGGGTCCGTTCCCGGGTTGAAGGTCGTT
AGTCCGTGGAGCGCAGAGGACTGCAAGGGCCTGCTCAAGGTACGTATCCCTTCGAAA
GTGTTTCCTTCAACATGTTCCCTATCCCGCAGTCCGCTATCCGC

> Pdb1 (FG855)_*Stereum hirsutum*
CAGGCCATTGACCAGATCGTGAACTCGGCCGGAAAGACATACTACATGTCCGGCGGGA
ACGTCCCCTGCCCTGTCGTCTTCCGTGGTCCGAACGGTGCCGCCGCTGGTGTCGGCGC
TCAGCACTCGCAGGACTACGCGTCGTGGTATGGTCAGATTCCCGGTTTGAAAGTCGTC
AGCCCGTGGAGTGCGGAGGATTGTAAAGGTCTGCTCAAGGTATGTGGTCACTACTCTT
CCTCGTCCGTACTGCGGCGCGTATCGGACAGACCGCCATTCGT

> Pdb1 (FG855)_*Agaricus bisporus* var. *bisporus*
CAGGGAATTGATCACATCGTCAATTCTGCTGGCAAAACATATTACATGTCAGGTGGTAA
TGTCCCTTGTCCCGTTGTGTTCCGAGGTCCCAACGGCGCTGCTTCTGGTGTGGCTGCA
CAGCACTCGCAGGATTATGCGGCCTGGTACGGCTCTATCCCAGGACTCAAGGTTATCAG
CCCCTGGAGTGCTGAGGATTGCAAAGGTCTCCTCAAGGTCTGTGTATTTATTTCGTCCA
GATGCATGTGTCTCAATTCCGTTCTAGTCCGCTATTCGG

> Pdb1 (FG855)_*Laccaria bicolor*
CAAGCAATTGATCAAATTGTCAACTCGGCCGGTAAAACATACTACATGTCTGGTGGCAA
TGTCCCATGCCCAGTCGTGTTCCGCGGTCCCAATGGTGCTGCTGCCGGTGTTGGTGCA
CAACACTCCCAGGATTACGCAGCTTGGTATGGTTCAATTCCCGGATTGAAGGTTGTCAG
TCCCTGGAGTTCTGAAGATTGCAAAGGACTACTCAAGGCAGGAAACTGACCCACTCTT
CTTCCGTATCATTTACCTGTTCTAGTCGGCAATTCGA

> Pdb1 (FG855)_*Lactarius quietus*
CAAGCGATCGACCAAATCGTCAACTCGGGCGGGAAGACATACTACATGTCTGGTGGGA
ATGTCCCATGCCCTGTTGTTTTCCGCGGTCCGAACGGTGCTGCTGCTGGCGTTGGTGCC
CAACACTCCCAGGATTATGCGTCTTGGTACGGACAGATCCCAGGACTTAAAGTTGTCA
GTCCATGGAGCGCCGAGGATTGCAAAGGTCTGCTCAAAGTGCGTAATTTCTTCGTGTA
AGATGCTAACTGGCGGCCATATTCAGTCCGCCATTCGC

> Pdb1 (FG855)_*Coprinopsis cinerea*
CAGGCCATTGATCAGATTGTCAACTCGGCAGGCAAGACCTATTACATGTCTGGCGGCA
ACGTTCCCTGTCCCGTCGTATTCCGAGGCCCCAACGGTGCTGCTCTCGGTGTAGCTGCC
CAGCATTCCCAGGACTATGCCGCTTGGTATGGTTCTATCCCTGGATTGAAGGTCGTCAG
CCCATGGAGTGCCGAAGACTGCAAGGGTCTGCTCAAGGTAAATGATTCATATTCCGCTC
CCCCGAGTCAATCTTAAATACATCACAGTCCGCCATCCGG

> Pdb1 (FG855)_*Boletus edulis*
CAAGCCATCGACCACATCGTCAATTCTGCTGGTAAAACGTACTACATGTCCGGCGGAA
ACGTCCCATGCCCAGTTGTTTTTCGTGGTCCCAACGGTGCCGCCTCTGGTGTTGCTGCA

CAACATTCACAGGACTATGCTGCATGGTATGGCTCAATTCCTGGTCTCAAGGTCGTCAG
CCCATGGAGTGCCGAGGACTGCAAGGGTCTCCTCAAGGTGTGTGCGTCGTCCGATTCC
TCGTACATTCCTCATACACATGCAGTCTGCCATCCGG

> Pdb1 (FG855)_*Phanerochaete carnosa*
CAAGCCATCGACCAGATCGTCAACTCTGCGGGAAAGACCTATTACATGTCCGGCGGAA
ACGTCCCATGTCCAGTCGTCTTCCGTGGACCCAACGGTGCAGCTGCTGGTGTCGCCGC
ACAACACTCGCAAGATTACGCGGCATGGTATGGCAGCGTTCCAGGCCTAAAGGTCATC
AGCCCGTGGAGTGCCGAGGACTGCAAGGGTCTGCTCAAGGTGCGTACCCGTCGCGGG
GATCCGCCATATGTATCATTGCTCATAGGCTGCCATCCGT

> Pdb1 (FG855)_*Gymnopus androsaceus*
CAGGCCATTGACCAGATAGTCAACTCGGCTGGGAAAACATACTACATGTCGGGAGGAA
ATGTGCCTTGCCCAGTAGTGTTCCGTGGTCCTAACGGTGCTGCTGCTGGTGTTGCTGCG
CAACATTCGCAGGACTATGCTTCTTGGTATGGTCAGGTTCCTGGTCTCAAGGTTGTCAG
TCCGTGGAGTGCAGAAGACTGCAAGGGTCTTCTCAAGGTGAGTTGAGCTTCGCGTTTT
TGTATTCAACAACTTGTTATTCTAGTCGGCTATCCGT

> Pdb1 (FG855)_*Trametes versicolor*
CAGGCTATTGACCAGATCGTGAACTCTGCTGGCAAGACCCACTACATGTCGGGTGGTG
TTCTCCCTTGTCCTATCGTGTTCCGTGGTCCCAACGGTGCTGCCGCCGGTGTCGCGGCC
CAGCACTCACAAGACTACGCCTCGTGGTACGGTCAGGTCCCTGGTCTCAAGGTCGTCA
GCCCTTGGAACGCGGAGGACTGCAAGGGTCTTCTCAAGGTACGTTGGAGTGGAACGA
CCTATACATGGAAAGACTGACCCGATTGAAGTCTGCCATCCGG

> Pdb1 (FG855)_*Hygrophoropsis aurantiaca*
CAAGCCATTGACCAAATCGTTAACTCGGCCGGAAAGACGTATTATATGTCGGGCGGCAA
TGTCCCATGCCCTGTAGTCTTCCGTGGTCCCAATGGTGCAGCTGCCGGAGTTGCCGCTC
AACATTCACAAGATTATGCTGCGTGGTACGGTTCAGTTCCCGGCCTCAAAGTTGTCAGT
CCTTGGAGTGCTGAGGATTGCAAGGGTCTTCTGAAGGTAAACTTCCTTTCCATTTCTGC
TCATGTATTGCGTTTTTCCAAATAGGCGGCTATCCGC

> Pdb1 (FG855)_*Fomitiporia mediterranea*
CAGGCCATTGATCAGATTGTGAACTCTGCTGGCAAGACGTACTACATGTCTGGTGGGA
ACGTTCCTTGTCCTGTCGTTTTCCGTGGACCCAACGGCGCGGCCGCTGGTGTAGCAGC
GCAGCACTCCCAAGACTATGCGTCGTGGTATGGTCAAATCCCCGGTCTTAAGGTCCTCA
GTCCCTGGTCTGCGGAGGATTGCAAAGGTCTATTGAAAGTACGCGTTCTCTCTTACGTG
TTGTCATTTTCGTAATTTCTCTGATAGGCTGCTATTCGC

> Pdb1 (FG855)_*Pluteus cervinus*
CAAGCTATCGACCAAATAGTGAACTCAGCCGGAAAAACTTATTACATGTCTGGCGGCA
ATGTTCCTTGTCCCGTTGTGTTCCGTGGACCCAACGGTGCCGCAGCTGGTGTTGGCGC
ACAACATTCACAAGACTACGCTGCGTGGTATGGTTCGATCCCTGGACTTAAAGTTGTGA
GTCCATGGAGCGCAGAGGACTGCAAGGGTTTACTCAAGGTATGATCATTAGCACCCTG

ACCGCACCAAATTCCACTCCTTACAGTCCGCCATCCGA

> Pdb1 (FG855)_*Tricholoma matsutake*
CAGGCTATTGACCAAATTGTCAATTCGGCGGGAAAAACATACTATATGTCCGGTGGCAA
CGTTCCATGTCCGGTTGTGTTCCGCGGTCCTAATGGCGCTGCGGCTGGTGTTGCAGCCC
AACATTCGCAAGACTATGCTGCTTGGTATGGTTCCGTACCTGGCCTAAAGGTCGTCAGT
CCATGGAGTTCGGAGGATTGCAAAGGTCTACTCAAGGTATGTCGACTTAGTGTCATAAA
CATCAACTAATTGAACTCAGTCTGCTATCCGA

> Pdb1 (FG855)_*Auricularia delicata*
CAGGCCATCGACCAGATCGTCAACTCGGCAGGCAAGACTTACTACATGTCGGGTGGAA
ACGTCCCCTGCCCCGTAGTGTTCCGTGGCCCCAACGGTGCCGCGTCCGGTGTCGCCGC
CCAGCACTCCCAGGACTACGCGGCATGGTACGGCCAAATCCCCGGTCTCAAGGTGCTC
TCGCCCTGGAGTGCTGAGGACTGCCGCGGCCTGCTCAAGGTCACACGCCCATACACCG
CACCCCCTCTACTGCTCGCACAGGCAGCAATCCGC

> Pdb1 (FG855)_*Fomitopsis pinicola*
CAGGCCATCGACCAGATTGTGAACTCGGCCGCAAAGACACTCTACATGTCTGGTGGTT
TCCTCACCTGCCCGATCGTCTTCCGTGGTCCCAACGGTGCCGCTTCCGGTGTCGGAGC
CCAGCATTCGCAGGACTACGCTGCATGGTACGGCTCTATCCCCGGTCTCAAGGTAGTCA
GTCCCTGGAGTGCTGAGGACTGCAAGGGCCTGCTCAAGGTTTGCTCCGCCCATAGTGA
TGTGCCCACTTAAATCTTCGTGTTACAGGCCGCCATCCGT

> Pdb1 (FG855)_*Amanita muscaria*
CAAGCAATAGACCAGATTGTGAACTCTGCTGGAAAAACACATTACATGTCCGGCGGCA
ACGTCTCATGTCCGATAGTTTTCAGAGGACCCAACGGTGCGGCCGCTGGAGTCGGTGC
CCAGCATTCACAAGATTATGCAGCCTGGTATGGGTCTATTCCAGGACTTAAAGTTGTCA
GTCCGTGGAGTGCGGAGGATTGCAAAGGTCTGCTCAAGGTATGTTCATCAATGCATTAT
CTCAATTCTCGTGATAGGCAGCAATACGG

> Pdb1 (FG855)_*Marasmius fiardii*
CAAGCCATTGATCAAATCGTCAACTCTGCCGGAAAGACCTACTACATGTCCGGAGGGA
ACGTTCCTTGTCCTGTTGTTTTCCGTGGCCCGAATGGTGCGGCATCAGGTGTAGCAGCT
CAGCATTCACAAGATTACGCCGCTTGGTATGGTCAGATTCCCGGTCTGAAAGTTGTCAG
TCCATGGAACGCTGAGGATTGCAAGGGCCTTTTGAAAGTGAGTTACGGAAGGCTCAG
GCTCTATCTAAATAAAATAGTCTGCTATCCGT

> Pdb1 (FG855)_*Dacryopinax primogenitus*
CAGGCGATTGACCAGATCGTTAACTCGGCTGGAAAGACACATTACATGTCTGGCGGCG
AGGTCCATTGCCCAGTTGTCTTCCGCGGGCCAAATGGTGCCGCGTCCGGTGTTGCTGC
CCAACACTCGCAGGACTACTCTGCTTGGTACGGCTCTGTCCCTGGCCTGAAGGTGGTC
AGCCCTTATAGTGCTGAAGACTGCAAGGGTCTCTTGAAGGTAGGATTAAGTCTTCATAG
GCTTGCCCTGACATGTGGAAAGGCGGCTATCCGC

> Pdb1 (FG855)_*Calocera cornea*
TAGGCTATTGACCAAATCGTGAACTCTGCCGGGAAGACATATTACATGTCTGGCGGCAA
TGTTCCCTGCCCAGTCGTCTTCCGTGGTCCCAACGGTGCTGCTGCTGGTGTTGCTGCCC
AGCATTCGCAAGATTATGCTGCTTGGTACGGCTCCATCCCGGGCCTGAAGGTTGTCAGC
CCTTGGAGCGCTGAGGACTGCAAGGGTCTGTTGAAGGTACGACCATGCTGCCGTAATG
CTCATCTAATATATGACAAGGCCGCGATCCGT

> Pdb1 (FG855)_*Ramaria acris*
CAGGCCATTGATCAAATTGTGAACTCGGGTGCGAAGACGTATTACATGTCAGGAGGAA
ATGTCCCATGTCCTGTTGTTTTCCGTGGTCCTAACGGTGCAGCCGCTGGTGTCGCCGCG
CAACACTCTCAAGACTATGCTGCTTGGTATGGTCAGATACCTGGCCTTAAAGTCGTTAG
TCCATGGAGCTCGGAAGATTGCAAAGGCCTCCTAAAGGTATGTTGCCATTCTACGCGGT
CGTATCTGATAATTTCCTCCTCGCCTAGGCCGCAATCCGT

> Pdb1 (FG855)_*Gautieria morchelliformis*
CAGGCCATCGACCAGATTGTTAACTCGGGAGGGAAAACATATTACATGTCAGGCGGCA
ATGTCCCTTGTCCTGTTGTTTTCCGTGGTCCCAATGGTGCAGCCGCAGGCGTCGCTGCG
CAGCATTCTCAAGATTATGCCGCATGGTACGGTCAAGTGCCAGGTCTTAAAGTCATAAG
CCCGTGGAGCTCTGAGGACTGCAAAGGTCTCCTAAAAGTGAGGCGAATCCCTTCGCCT
ACCTGAATCTAAGACGTTTCCCCTCACAGGCTGCCATTCGC

> Pdb1 (FG855)_*Rhizopus microsporus*
TAGGCTATTGATCAAATCGTCAACTCTGCTGCAAAGACCCTCTACATGTCTGGTGGTAT
CGTCAAGTGCCCCATTGTCTTCCGTGGTCCTAACGGTGCTGCTGCTGGTGTCGGTGCTC
AACACTCTCAAGACTTTGCTGCTTGGTACGGTTCCGTCCCTGGTCTTAAGGTCGTCTCT
CCTTGGAACTCTGAAGATGCCAAGGGTTTGTTGAAGGCTGCCATTCGT

> Pdb1 (FG855)_*Neurospora crassa*
CAGTCCATCGACCACATCGTCAACTCTGCCGCCAAGACGCTCTACATGTCCGGCGGTAT
CCAGCCCTGCAACATCACCTTCCGCGGTCCCAACGGTTTTGCCGCCGGTGTCGCCGCC
CAGCACTCGCAAGACTACAGCGCCTGGTACGGTTCCGTCCCTGGTCTCAAGGTCGTGT
CCCCCTGGTCTGCTGAGGACGCCAAGGGTCTGCTTAAGGCGGCTATCCGC

> Pdb1 (FG855)_*Asperigillus triticus*
CAGGCCATCGATCAGATCATCAACTCGGCCGCTAAAACCCACTACATGTCTGGAGGTAT
CCAACCTTGCAACATCACTTTCCGTGGCCCCAACGGGTTCGCCGCCGGTGTCGCCGCG
CAGCACTCTCAGGATTACTCCGCCTGGTACGGCAGTATCCCTGGCCTGAAGGTTGTCTC
GCCCTGGAGCTCGGAGGATGCCAAGGGTTTGCTGAAGGCCGCCATCCGC

> Pdb1 (FG855)_*Agaricostilbum hyphaenes*
CAAGCAATCGACCAAATCGTCAACTCTGCAGCCAAGACCTACTACATGTCTGGCGGTA
ACGTGCCGTGCTCAGTTGTGTTCCGTGGGCCAAACGGTGCAGCAGCCGGTGTCGCCG
CTCAACACTCACAGGATTACGCCTCATGGTACGGTCAGATTCCAGGTCTCAAGGTCGT
GTCACCCTACAGCGCAGAGGACCACAAGGGTCTCCTCAAGGCAGCAGTTCGC

>Pol30 (FG546)_*Agrocybe pediades*
GCCCTGGTCTCCGTCAAGTTCGGTGCCCCCGCTTTCAAGCGCTATCGCTGTGATCGCCC
CATGCCACTTGGCGTGAACCTCACCAGCCTGACTAAAGTCCTCAAGTGTGCCAAAGAC
GATGATATTTGCACGTTGAAGGCCGCGGATGAGGCGGATGTGTTGAATTTGGTGTATGA
GGC

> Pol30 (FG546)_*Boletus edulis*
GCCCTTGTCGCCGTTACCCTCGAGGCATCTGGTTTCAAAAAGTTCCGCTGCGACCGTC
CCATGCCCCTAGGCGTCAACCTGAACTCCCTGACCAAAGTGCTGAAATGCGCGAAAGA
CGACGACATATGTACAATCAAAGCTACTGACGATGTCGATGTATTGAACCTCGTCTACG
AAGC

> Pol30 (FG546)_*Fomitopsis pinicola*
GCCCTCGTCGCGGTCAGCCTAAAGGCCTCCGGCTTCAAGCGCTACCGCTGCGATCGCC
CCATCCCCCTCGGCGTCAACGTCTCCTCCTTCACAAAGGTCCTCAAGTGCGCAAAGGA
CGATGACGTCTGCATTCTCAAGGCCAACGACGACGCCGACATCCTCAGCCTCACCTAC
GAGGC

> Pol30 (FG546)_*Trametes versicolor*
GCCCTCGTCGCCGTCAAGCTCAAGGCAGACGGCTTCCAAAAGTACCGCTGCGACCGC
CCCATCCCGCTCGGCGTGAACGTCGGGTCGCTCACAAAGGTCCTCAAGTGCGCCAAG
GACGACGACATCTGCACGCTCAAGGCCGCCGACGACGCCGACATCCTCTCCCTCACCT
ACCAAGC

> Pol30 (FG546)_*Phanerochaete carnosa*
GCGCTCGTGGCTGTCAAACTCAACGCGACTGGCTTTAAGCGCTACCGCTGCGACAGGC
CTATGCCACTCGGTGTGAATCTCACATCGCTGACAAAGGTCGTGAAGTGCGCAAAGGA
CGACGACCTCTGCACGCTTAGCGCGGCAGACGACGCCGATGTCCTCAGCTTGACTTAC
GAGGC

> Pol30 (FG546)_*Gymnopilus chrysopellus*
GCGCTCGTCTCGGTCAAGTTCAGTGCTGCTGCCTTCAAGCGGTACCGCTGCGACAGAC
CCATGCCGCTGGGCGTGAACCTCACCAGTCTCAACAAGGTGCTCAAGTGCGCCAAGG
ACGACGATATCTGCACGCTCAAGGCCGCTGATGAAGCTGACGTGCTCAACCTCGTGTA
TGAGGC

> Pol30 (FG546)_*Laccaria bicolor*
GCTCTCGTAGCAGTCCATTTGGAAGCTGCGGGGTTTAAGCGTTACCGTTGTGATCGCCC
TATGCCCTTGGGCGTGAACCTGACCTCCCTCACAAAGGTTCTCAAGTGTGCCAAGGAC
GATGACATTACCACCCTCAAGGCTGCAGACGAGGCCGATGTGCTTAATCTTGTCTATGA
GGC

> Pol30 (FG546)_*Hygrophoropsis aurantiaca*

GCCCTGGTTGCAGTGAAGTTGGCGGCTACGGGTTTCAAGAAATACAGATGTGATAGAC
CAATGCCTTTGGGTGTCAACCTTAATTCCCTCACCAAAGTTCTCAAATGTGCCAAAGAT
GATGATATCTGTATCCTCAAAGCTACGGATGATGCCGACGTTTTGAACCTTACATACGAA
GC

> Pol30 (FG546)_*Serpula lacrymans* var. *lacrymans*
GCTTTAGTGGCGGTGAAGCTGGACGCTGTTGGCTTCAAGAGATATCGCTGTGACCGCC
CAATGCCTCTCGGTGTCAACCTCAATTCCCTTACCAAAGTCCTCAAGTGTGCAAAAGA
CGACGATATATGTGTCATAAAGGCCACTGATGACGCTGACGTGCTTAATCTCGTATACG
AAGC

> Pol30 (FG546)_*Lactarius quietus*
GCGCTTGTATCGGTCAAGTTACACTCCGATGGGTTCGCGTTGTACCGCTGCGACCGTCC
GATCCCCCTTGGCGTCAACCTCGGTTCACTTACCAAGGTGCTCAAGTGTGCTAAGGAC
GACGATAAGTGTACACTGAAAGCGACTGACGACGGTGATGTTCTCAGTCTCAAGTACG
AGGC

> Pol30 (FG546)_*Mycena crocata*
GCGCTCGTGGCCGTCTTTCTCGAAGCCGATGGCTTCAAGCGATATCGCTGCGACCGAC
CGATGCCCCTCGGTGTCAATCTGTCGAGTCTGACCAAAGTCCTCAAGTGCGCAAAGGA
CGACGACGAGTGCACGCTCAAGGCCGCCGACGACGCCGACGTCCTCAATCTCGTGTA
CGAGGC

> Pol30 (FG546)_*Gautieria morchelliformis*
GCTCTCGTTGCTGTCAAGCTTGACGCGGACGGTTTCGACTTGTATCGATGTGACCGACC
GATGCCGCTTGGCGTTAACCTTGGATCTTTAACAAAAGTCCTCAAGTGTGCGAAGGAC
GACGACATAGTCACACTGAAAGCGACCGACAATGGCGATATTTTGAACTTGATTTATGA
AGC

> Pol30 (FG546)_*Ramaria acris*
GCACTGGTCGCCGTTAACCTCGAAGCCGAGGGTTTCAAGTCTTATCGATGTGACAGGC
CTATGCCCCTTGGTGTCAACCTGGGATCCTTGACGAAGGTGCTCAAATGCGCCAAGGA
CGACGACATTGTCACACTCAAAGCAACCGACAATGGTGATATCCTGAACTTGATTTACG
AAGC

> Pol30 (FG546)_*Tricholoma matsutake*
GCGCTTGTTGCTGTACTCCTTGAGGCCACTGGCTTTAAACGCTATCGCTGTGACCGCCC
CATGCCCTTAGGTGTCAATCTTACCAGCCTAACCAAAGTCCTCAAGTGTGCGAAGGAC
GATGATGAGTGCACTCTTAAAGCCGCAGACGAGGCTGATGTCCTTAACTTGGTTTACG
AGGC

> Pol30 (FG546)_*Agaricus bisporus* var. *burnettii*
GCGCTGGTCATTATCAACTTGGACCACAAACTCTTCCTCAGTTATAGATGCGATAGGCC
CATGCCCTTGGGAGTCAATCTCAATAGTTTGGCCAAGGTCCTAAAATGTGCAAAGGAC

GACGACGTTTGCACGTTGAAAGCAAGCGACGATGCTGACCTCTTAAATCTGGTCTACG
AGGC

> Pol30 (FG546)_*Stereum hirsutum*
GCACTCTGCTCCGCCAAGCTCGACGCGACTGGATTCACACGCTATCGCTGCGATCGTC
CTCTCCCGCTCGGCGTCAACCTCAGCAGCTTGACAAAGGTTTTGAAGTGTGCAAAGGA
TGATGATACATGTACGCTGAAGGCGAGCGATGACGGTGACATCTTGAGTTTGACTTACG
AGGC

> Pol30 (FG546)_*Marasmius fiardii*
GCCTTAGTGGCCGTAAAGCTACAACATCAGGGCTTCAAAAAGTACCGTTGCGATAGGC
CAATGCCTCTCGGCGTGAACATTGCTAGCTTGACAAAGGTTCTCAAGTGCGCGAAGGA
TGATGACTTGTGCACTCTCAAGGCAGCGGATGAAGCAGACGTGCTTAACCTGGTATAT
GAAGC

> Pol30 (FG546)_*Amanita muscaria*
GCGCTCGTCTCTGTGCTCATCAAAGCAAAAGGCTTTGAGAGGTTCAGATGTGACCGCC
CAATGCCACTGGGTGTCAATCTCGGCAGTCTGTCCAAAGTCCTCAAGTGTGCGAAAGA
CGATGATACGTGCACGCTCAAGGCTCTAGACGACGCAGATACTTTAAATCTCATCTATG
AGGC

> Pol30 (FG546)_*Auricularia delicata*
GCCCTTGTCAGCGTCAACATGGACGTGGCTGGCTTCCAGATGTACCGCTGTGACCGCC
CGATGCCGCTGGGCGTGAACCTGGCATCGCTGACCAAGGTGCTCAAGTGCGCCCAGG
ACAACGATCAGGTCACGCTCAAGGCGGCGGACTCGGCGGACGTCCTTCAACTGCAAT
TCAACTC

> Pol30 (FG546)_*Gymnopus androsaceus*
GCCCTCGTCGCGGTAAAATTGGTCCAGAGTGGCTTCAAGAAATATCGCTGTGACCGGC
CGATGCCTCTGGGCGTCAACGTCGGAAGCTTGACAAAAGTATTGAGATGCGCCAAGGA
CGACGATACATGTACTCTCCGTGCAGCTGACGAGGCGGACGTGTTGAATTTGACTTATG
AACC

> Pol30 (FG546)_*Pluteus cervinus*
GCACTCGTTTCGGTGCATCTTATGGCTTCGGGCTTCAAACGATACCGTTGTGACAGGCC
AATGCCATTGGGCGTCAACCTGAACACGTTGACCAAGGTGTTGAAGTGTGCGAAGGAT
GATGATATCTGTATATTGAAGGCGGCGGATGAAGCGGATTTGTTGAACCTTATATATGAA
GC

> Pol30 (FG546)_*Schizophyllum commune*
GCGCTCGTCGCGGTGAAGATCCTGGTCTCTGGCTTCAAGCGCTATCGATGTGACCGAC
CCATGCCTTTGGGCGTCAACCTGGGCAGCCTGACCAAAGTCTTGAAGTGCGCAAAGG
TGTGTGTCTTGTCCAACCGCCCGAAAGGGCAACCGCGCTAAGATGATCCTAGGACGAC
GATATCTGCACATTGAAAGCGGCAGACGAAGCGGACGTCCTAAACCTCATCTACGAAG

C

> Pol30 (FG546)_*Coprinopsis cinerea*
GCGCTCGTCGCGGTGAAGATCCTGGTCTCTGGCTTCAAGCGCTATCGATGTGACCGAC
CCATGCCTTTGGGCGTCAACCTGGGCAGCCTGACCAAAGTCTTGAAGTGCGCAAAGG
TGTGTGTCTTGTCCAACCGCCCGAAAGGGCAACCGCGCTAAGATGATCCTAGGACGAC
GATATCTGCACATTGAAAGCGGCAGACGAAGCGGACGTCCTAAACCTCATCTACGAAG
C

> Pol30 (FG546)_*Fomitiporia mediterranea*
GCACTCGTCGCCGTTTCCCTGAAGGCGGATGGTTTCGTCAAGTACCGTTGCGACAGAC
CGATGCCGCTGGGTGTCAACCTTGGATCTCTCACAAAAGTGCTCAAGTGCGCAAAGGA
CGACGATGTCTGCACGCTCAAGGCCTCCGATGATGCGGACGTACTGAATCTTACGTACG
AAGG

> Pol30 (FG546)_*Calocera cornea*
GCGCTCGTTGCCGTCGAGCTCCAGGCGGATGGCTTCACCGAGTACCGCTGTGATCGGC
CGATGCCCCTCGGCGTTAACCTCGCGAGCTTGACGAAGGTCATGAAGTGCGCACGGGA
CGATGACGAGGTTCAGCTCAAGGCGAGCGATAATGCCGACTCTCTGCATTTGCTCTTCG
AGAA

> Pol30 (FG546)_*Dacryopinax primogenitus*
GCTCTCGTAGCGGTTGAGCTCCAGGCAGATGGTTTCACAGAATACCGCTGTGACCGGC
CGATGCCCCTTGGTGTGAATTTGGCCAGCTTGACCAAGGTCATGAAGTGCGCGCGTGA
TGATGATCAGGTGCAGCTCAAAGCCAACGACAATGCTGACTCGTTGCATCTCCTGTTC
GAAAA

> Pol30 (FG546)_*Neurospora crassa*
GCGCTCGTGTCCATGATGCTCAAGACGGAGACCTTCTCGCCCTTCCGGTGCGACCGCA
ACATTGCGCTCGGCGTCAACCTGACGTCGCTGACCAAGGTGCTCCGCGCCGCCCAGA
ACGAGGACATCCTGACGCTCAAGGCCGAGGACGCGCCCGACGTGCTCAACCTCGTCT
TCGAATC

> Pol30 (FG546)_*Asperigillus triticus*
GCGCTGGTGTCGATGCTGTTGAAGGCGGAGGGCTTCTCGCCCTACCGGTGCGACCGCA
ACATCGCCCTGGGTATCAACCTGGTCTCCCTGACCAAGGTGCTGCGCGCCGCCCAGAA
CGAAGACATCCTGACGCTCAAGGCGGACGACTCGCCCGACGCCGTCAACCTGATGTTC
GAGAG

> Pol30 (FG546)_*Rhizopus microsporus*
GCCTTGGTGGCCATGATGCTTCGCTCTGATGGTTTTGATCCTTACCGCTGTGATCGTAAC
TTGCCTTTGGGTATCAACTTGACCAACTTGGGTAAGATTTTAAAGTGTGCTCGTAACGA
TGATATCGTGACACTCAAGGCTGATGATGATGGTGATGCTTTAAGTTTGGTATTTGAAA
G

> Pol30 (FG546)_*Agaricostilbum hyphaenes*
GCTTTGGTCTCCCTAGATCTTCAACCTGAATCCTTCATGGATGACGCCTACCGCTGTGA
CCGCAACATGTCCTTGGGCATGAACTTGAGCAGCTTGCAGAAGATCATCAAGTGCGCC
GGCAACGATGATGAAGTGACTTTGCGCGCCGACGATGACTCAGACAACCTTTCACTTA
CATTTCAGAA

>Qns1 (FG747)_*Boletus edulis*
CTCCGAAGATCCCGGACTCAGGGATACTTTGTGCCTCTTAGCGGTGGAGTCGACAGCT
GCGCCACGGCAGTTATCGTGTATTCCATGTGCCGCTTGGTCGCCGAAGCTGCCACTCGA
GCGAGTAAGTACATCCGGTCATCGCTCTTTAACTCACTAGACAAGCAGGTCATTGCTGA
TGCCCGTCGCATTGCGGGAGAGCCTGACGACTCGAGCTACATCCCTTCCGACCCCAAG
GAGTTTTGCAATCGCATA

> Qns1 (FG747)_*Stereum hirsutum*
CTGCGACGCTCACGGACGCAGGGATATTTTGTCCCGCTGAGCGGCGGGATTGATAGCT
GTGCCACCGCCGTCATTGTCTATTCGATGTGTCGGCTGGTCGCTGAAGCTGCGCGTCGA
GCAGGTCCGTGTCTCCCTAGTTTGTTATTGACACGAACAGACAAACAGGTCATCGAGG
ATGCGCGCCGTATCGTCGGAGAACCAGCGGACTCTGGCTACATACCCTCAGATCCGAG
AGAGTTTTGCGGTCGTATA

> Qns1 (FG747)_*Coprinopsis cinerea*
TTGCGAAGGTCGAGAACCCAGGGCTACTTTGTTCCTCTGAGCGGTGGAATCGATAGCT
GTGCAACCGCCGTGATCGTGTTCTCCATGTGCCGCTTGGTAACTGAAGCATCGGCTCGT
GGAGGTAAACGAGTTTCACGCTACTGACCTAACCAGAGCAACATGTCATCGCCGACGC
AAGGAGAATGACCGGAGAACCTGTGGACTCTACCTATATTCCGACCGACCCCAAGGAA
TTCTGCAACAGGATT

> Qns1 (FG747)_*Agrocybe pediades*
TTGCGACGCTCACGGACTCAGGGCTATTTTATCCCTCTCAGCGGTGGCATCGACAGCTG
CGCTACTGCCGTGATTGTTTACTCCATGTGTCGTCTAGTCGCCCAAGCTGCGTCCAGGG
GAGGTATGTGTCTTTTGATTTTAATGCTAATCAGAGGTGCAAGTCATTGCGGATGCCAG
ACGGATGACTGGAGAACCCGAGGATTCCGACTACCTGCCTACTGATCCTCGAGAATTT
GCCAACAGGATA

> Qns1 (FG747)_*Laccaria bicolor*
CTTCGACGATCCCGAACTCAGGGATATTTTATACCATTAAGCGGTGGCATTGACAGCTG
TGCAACTGCGGTCATCGTATATTCCATGTGCCGCCTTGTTTCAGAGGCCGCGCTTCGGG
GAGGTCAGCCTATTTTTCTACGTCTTGACTAGAGCAGCAAGTCATCGCTGATGCGAGGC
GAATGACTGGGGAGCCGGATTCCTCCTACATTCCTTCCGATCCCAGGGAATTTGCGAAC
AGGATT
> Qns1 (FG747)_*Gymnopilus chrysopellus*
CTTCGCCGTTCAAGAACGCAAGGATATTTCGTACCCTTAAGCGGCGGGATTGATAGTTG
CGCCACTGCTGTGATTGTTTATTCTATGTGTCGTCTGGTTGCGCAGGCTGCTGCAAGAG

GGCGTAAGTTGCTTTTTTTGGCTCAGAGAAGCAAGTAATTGCCGACGCCAGACGTATG
GCTGGCGAACCTGAGGGTTCAGAATACTTGCCGACCGATCCAAAGGAATTCTGTAACA
GGATT

> Qns1 (FG747)_*Trametes versicolor*
CTGCGCAGATCGCGCACGCAAGGGTATTTTATTCCTCTCAGTGGGGGCATCGACAGTTG
CGCCACGTCTGTGATAGTCTTTTCCATGTGCCGTCTAGTTGCCGAGGCAGCGCGGAAC
GGAGGCAAGTGGGCTTCAACACACCCCGCTCTGACCAGATGGACAGGTCATTGAGGA
CGCGCGACGCATCGCGGGCGAAGAGCCGGGGTCCACATACGTGCCAGACGATCCCCG
CGAGTTCGCGAACCGCATC

> Qns1 (FG747) _*Gautieria morchelliformis*
CTCCGTCGGTCGCGCACACAGGGGTATTTCATTCCTCTTAGTGGCGGAATCGACAGCTG
TGCTACTTCCGTTATTGTGTATTCTATGTGCCGTCTCGTTGCAGAAGCTGCCACTAGGGC
GGGTAAGAATTTCTTTTGTTGACTCGGTCAGATAAACAGGTCATCGAGGATGCTCGAA
GGATGACAGGCGAGCCCCCGGATTCTAGTTATATCCCTTCCGATCCTAGGGAGTTTGCA
AACCGCATA

> Qns1 (FG747)_*Ramaria acris*
CTTCGCCGGTCGCATACCCAAGGCTATTTCATTCCCCTCAGCGGAGGGATTGATAGTTG
TGCCACCTCGGTCATTGTCTATTCCATGTGTCGCCTCGTAGCCGAGGCTGCCACTAGGG
CCGGTAAAGATGTTAACAATGGCCCACTCATCTGGTTAGATAAACAGGTAATTCAAGAT
GCTCGAAGGGTAACTGGAGAGCCTCCAGACTCCAGTTACATACCTTCCGACCCTCGCG
AGTTTGCTGGCCGCATA

> Qns1 (FG747)_*Agaricus bisporus* var. *burnettii*
CTAAGGCGTTCTCGAACCCAAGGCTACTTTCTTCCTCTCAGTGGGGGTATAGACAGCTG
TGCAACAGCAGTGATTGTACATTCTATGTGCCGCCTCGTCGCGGAGGCATCTAACCGTG
GCGGTGAGTGGGATTTTTTAACAAATCACCTTACCAGACAAGTCGGTTATCGCTGATGC
GAGAAGAATGACTGGTGAACCGGAAGACTCCTCCTATATTCCTTCGGACCCTGTGGAA
TTTGCTGGTCGCATA

> Qns1 (FG747)_*Hygrophoropsis aurantiaca*
CTCAGGCGTTCACACACACAAGGATATTTTATTCCTCTGAGCGGAGGTATCGACAGCTG
TGCAACGGCAGTTATTGTCTATTCTATGTGCCGTTTAGTTGCGGAAGCAGCTACTAGAG
CAGGCGAGTCAAACAAATCCTTGACTAAGAACTCACTAGACAAGCAGGTCATTTCTGA
TGCTCGCCGCATCGTCGGAGAACCGGAAGAGTCGAGCTATATTCCCTCTGATCCTCGG
GAGTTTTCAAGCAGGATC

> Qns1 (FG747)_*Phanerochaete carnosa*
CTTCGTCGCTCGCGGACCCAAGGTTACTTTGTTCCTTTGAGTGGAGGCATTGATAGCTG
CGCAACAGCCGTCATCGTGTACTCCATGTGTCGTTTAGTCGCAGAAGCTGCTCAAAGA
AGAGGTATGGCTCGAGCGCGCGCATACGCTGATTAGACCAGCAAGTCATCTCAGATGC
GCGTCGCATCGCTGGCTTGCCAGAAGATTCCGATTATGTCCCTCAAGATGCACAAGAAT

ATTGTAACCGTATC

> Qns1 (FG747)_*Fomitopsis pinicola*
TTGCGCCGGTCGAGGACACAGGGGTACTTTGTTCCGCTGAGTGGTGGTATCGACAGCT
GCGCGACCTCGGTCATCGTTTATTCGATGTGTCGCCTCGTCGCCGATGCTGCCAGCAAC
GGAGGTACGCATGCGGCCTTTGCCAGCCTAATCAGATGAGCTGGTCATCACCGACGCG
CGCCGCATCGCGGGCGAGCCCGAGGACTCGTCTTACATCCCAACGGACGCACGCGAAT
TTTGCAACAGGATC

> Qns1 (FG747)_*Pluteus cervinus*
CTGAGGCGGTCCCGAACCCAAGGATATTTCGTTCCACTGAGTGGCGGTATAGACAGTT
GTGCAACAGCTGTCATCGTTTTCTCTATGTGCCGCCTTGTGGCAGAGGCAGCCAGACG
AGGGGGTATGTCGAGGTGGATTACATAGATCCCCAAGTAATTTCAGACGCTCGGCGCAT
GGTCGGTGAACCACCGGATTCAGATTACGTCCCGACAGACGCTCGCGAATTTTGCAAT
CGAGTT

> Qns1 (FG747)_*Tricholoma matsutake*
TTGAGACGATCGCGCTCGCAGGGCTATTTCATTCCCCTGAGCGGTGGGATTGACAGCT
GTGCGACAGCCGTCATCGTGTTCTCCATGTGCAGGCTTGTGGCTGAAGCTACCCGAAG
AGGAGGTTTAAAAAAGCTCTGTTTGTTTCTTACATGGTCAGACAAGCAAGTGATCGCA
GATGCAAGACGCATGACTGGAGAAGCAGAAGACTCTACATATATGGCTTCTGATCCAC
GCGAATTTTGCAATCGGATA

> Qns1 (FG747)_*Calocera cornea*
CTTCGTCGTTCCCGCACTCAAGGTTACTTCGTGCCGCTCAGTGGTGGTATCGACAGCTG
CGCAACATCAGTCATAGTGTACAGCATGTGTCGGCTTGTCGCACAGGCTGCAACCAAC
GCCGACAAGCAAGTAATTGCCGATGCACGACGCATTGTTGGGGAGCCAGATGACTCGA
GCTACATCCCTTCGGACCCAAGAGAATTTTGCAAGCGCATC

> Qns1 (FG747)_*Dacryopinax primogenitus*
CTTCGTCGCTCCCGAACGCAAGGGTATTTTGTACCCCTCAGTGGCGGTATTGACAGCTG
CGCAACCTCCGTCATCGTGTACAGTATGTGCCGTCTCGTGGCTCAATCAGCTGCAAAA
GGCGACAAGCAAGTGATTGCCGATGCACGGCGCATCGCTGGCGAGCCCGAAGATTCTA
GCTACATACCCTCGGATCCCAGAGAATTCTGCAAGCGCATC

> Qns1 (FG747)_*Gymnopus androsaceus*
CTTCGGCGATCTCGCACCCAAGGCTACTTCCTTCCTCTAAGTGGTGGTATAGACAGCTG
TGCGACCGCAGTCATCGTGTACTCGATGTGTCGCTTGGTCGCTGAAGCCGCAGGCAAA
GGCGACAAACAAGTTATTGCAGATGCTCGTCGGATTGCTGGCGAGAAGGAGAACTCTG
CATATGTTCCCACTGACCCACGAGAGTTTGCTGGGAGGATT

> Qns1 (FG747)_*Auricularia delicata*
CTGCGGCGCTCGCGCACGCAAGGCTTCTTCCTGCCGCTTAGCGGAGGGATAGACAGCT
GCGCGACCGCCGTCATCGTCCATTCGATGTGTAGACTAGTCGCGGCGGCGGGGGCGCA

GGGCAATGCGCAGGTCATCTCAGACGCGCGGCGGATGACGGGCGAACCGGAAGGCTC
CAGCTATCTGCCAACGGACCCGCATGAATTCGCAAGGCGAATC

> Qns1 (FG747)_*Schizophyllum commune*
TTGCGCCGTTCGCGCGCGCAGGGCTTCTTCCTCCCTCTCAGTGGCGGCATCGACAGCT
GTGCCACCGCGGTCATCGTCTACTCTATGTGCAGACTCGTCGTCGAGAAAGCCAAGGA
AGGCGGTACGTCACCTCCTTTCCCATCGCTCACCTCACCAGACCCCCAAGTCATCGCC
GACGCCCGGCGCATCTCCGGCGAGCCCGAGTCCTCCACCTACATCCCTACCTCCCCGC
ACGAGTTCGCCAACCGCGTC

> Qns1 (FG747)_*Lactarius quietus*
CTCCGCCGCTCGCGCACCCAGGGCTACTTCCTCCCGCTCAGTGGCGGAATCGACAGCT
GCGCGACGGCCGTGATCGCCTATTCGATGTGCCGGCTTGCTGTCGAAGCAGCGCGCAC
GGGGAGTACGTCCGACCTGTGCCGTCTGACGCTTCCCCCAGATGCACAGGTGATCGCG
GATGTACGGCGGATCGTCGGGGAGCCAGAAGGCTCTGAGTACGTCCCTACGGACGCGC
GCGAGCTCTGCGGCCGGATC

> Qns1 (FG747)_*Amanita muscaria*
TTACGTCGCTCACGTACCCAGGGATTTTTCTTGCCTTTAAGTGGTGGACTCGATAGCTG
TTCGACTGCTGTTATCGTACATTCTATGTGTCGTCTTGTGACTGAAAAGGCGCAAGCAG
GAGGTACGTCGGATCACCCTTCAACTGGTAAGACGCTTTGGTCCTTGCCGATGCCAGG
CGCATAGTCGGCGAGCCAGAAACCTCAATTTATGTTCCAACAAACCCGCAGGAATTTG
CTAACAGAATT

> Qns1 (FG747)_*Mycena crocata*
CTCAGGAGATCGCGTACACAGGGCTACTTTGTACCGCTGAGTGGCGGTATCGATAGTTG
CGCCACTGCCGTCATCGTCTTTTCCATGGCCCGGCTTGTGGCCGAAGCCGCTCGCAGA
GGCGGTCCGTCGTCCCTTCCCATTTTTTTCACCAGATGTACAAGTCATCGCAGATGCCC
GACGAATTTCGGGTGAACCCGACGGCTCGGAATACATCCCTTCTGACCCTCGTGAATTC
ACGAACCGCATA

> Qns1 (FG747)_*Fomitiporia mediterranea*
CTTAGGAGATCAAGGACGCAAGGGTACTTCCTACCTTTGAGCGGTGGGATTGACAGCT
GTGCGACTGCTGTCATTGTTTACTCAATGTGTAGACTTGTTTCAGAAGCTGCCCATCGT
GGAGGAAAGTTTAGTCCATCACGCTTAATATCCTTACCAGACGCTCAAGTCATTGCTGA
CGCGCGTCGAATCGCCGGCGCGCCCGAGGATTCATCTTACGTACCGACTGACCCGAAC
CAGTTCTGCCAGCGCATA

> Qns1 (FG747)_*Agaricostilbum hyphaenes*
CTTCGGCGCTCAAGAACAGCCGGCTACTTTGTGCCTCTCAGTGGCGGCATCGACAGTT
GTGCCACGGCAGTCATCACCTATTCCATGTGCAGACAGGTCGTCAAGGCTTGTGCCGA
AGGAAGTCAGTATATAGTTGCCGCGGTCGTGCTGATCAGACGCACAAGTCATCGAAGA
CGCTCGGCGCATTGGCGGCGAACCAGAAGACTCCGACTACATTCCTACAGATCCAAAA
GAGTTCTGCAACAGAATC

> Qns1 (FG747)_*Rhizopus microsporus*
CTTCGTCGCTCTAAAACAGCCGGGTACTTCTTACCTCTCAGCGGTGGTATCGATAGCTG
TGCTACTGCTGTCATTGTCGCCTCCATGTGTAGATTGGTGGTCAAAGAAGCTGCAAAG
GGTAACAAGGAAGTCATTGAGGATGCTCGTCGTTTAGCCTGTCAAGGTGACAATTACA
TCCCCACTGATCCTCGTGAATTCGCAAGTCATATC

> Qns1 (FG747)_*Asperigillus triticus*
CTCCGAAGATGCGGGGCTGTCGGATACTTCCTTCCGCTCAGTGGTGGGATCGACAGCT
GCGCCACTGCTGTGATTGTCCATTCAATGTGCAGGGAGGTAGTCAAGGCTGTCCAGGA
GGGAAATGAGCAAGTTATCGCGGATGTGCGCAAGCTCTGCGCCGAGCCCGAGGGCTC
AGACTGGCTTCCCAGCACAAGCCAAGAATGTTTTAGTCGTATC

> Qns1 (FG747)_*Neurospora crassa*
CTCCGAAGGTCTGGCACAGCGGGTTACTTGGTGCCCCTGAGCGGAGGCATCGACTCTT
GCGCCACGGCTACTCTCGTATTCTCCATGTGCCGTATCGTTATTCAAGCCATCGAAGAC
GGGAACCAGCAAGTCATTGATGATGTCAGGTGTATCTGCAAGTATGGCAAGGAAGGCG
AGCTTCCCAAGACGCCCCAAGAGCTTTGCAATCAGGTC

>Rfc2 (FG720)_*Pluteus cervinus*
CCCCCAGGGACAGGGAAAACGTCCACCATCCTTGCATTGGCCCGCCAACTCTTTGGGT
CCGTTGCAAAGTTTCAATTGACCCACAATCTCACACCTTGATAGGCCAGATAATTTCCG
AAATCGCGTCCTCGAGTTGAACGCGTCGGACGAACGTGGTATTGCCATTGTTCGTGAA
AAGGTCAAGAACTTTGCTCGACAGACGCCTCGAGCCAGGTTGTGGGCTCTGACGGGA
AGACTTACCCTTGTCCACCATATAAAATCATCATACT

> Rfc2 (FG720)_*Fomitiporia mediterranea*
CCTCCTGGGACAGGGAAGACCTCGACCATCCTTGCACTATCTCGTCAACTCTTCGGGTA
TGTCGCCTGCTATACGGAAGCCATCAGGCTTACACTTTTTATAGTCCAGACAACTTCAA
AAACCGCGTACTCGAGCTAAACGCATCCGACGAACGTGGCATAACGATCGTACGAGAG
AAAATCAAGAACTTTGCTCGTCAAACTCCTCGTGCCAAGCCGTCGCATCAGACGGCAA
AACCTATCCTTGTCCACCATACAAAATCATCATACT

> Rfc2 (FG720)_*Serpula lacrymans* var. *lacrymans*
CCACCGGGGACAGGGAAAACATCTACTATCCTCGCGTTGTCTAGACAGCTATTCGGGT
GTGTGCTTTTTGTACTTGTGAATCGTGATTTTTTAAACTTCTACGATAGACCAGATAACT
TTCGGAACAGGGTACTCGAGCTTAATGCATCTGACGAGCGTGGAATCAGTATCGTAAG
GGAAAAGATAAAAAACTTCGCTCGCCAAACGCCCCGAGCCAAATGGTCGCGTCCGAC
GGCAAAACATACCCTTGTCCACCATACAAAATCATCATCTT

> Rfc2 (FG720)_*Laccaria bicolor*
CCTCCGGGTACAGGCAAAACATCAACTATCCTTGCTTTGGCTCGACAACTTTTTGGGTA
AGAACGCGCTCTAGGTGACAATCGTTCTTTTTTCTGATAACTATATAGCCCAGACAATTT

TAGAAACAGGGTTCTTGAGTTAAACGCGTCGGATGAACGTGGAATCAGCATCGTTAGA
GACAAGATCAAGAACTTCGCCCGCCAGACACCGCGAGCCAAGCGGTGGCTTCTGATG
GAAAATCATACCCATGTCCTCCCTACAAAATTATCATCCT

> Rfc2 (FG720)_*Schizophyllum commune*
CCTCCTGGGACTGGAAAGACATCTACCATTCTCGCTCTGTCACGACAACTGTTCGGGT
GCGTATCCCAGTCATATTCGAGCTTCTTAGTACCCATTCTTTTGCAGTCCAGACAACTTC
CGTAGCCGCGTCCTCGAGCTCAACGCTTCAGATGAACGCGGTATCAGCATCGTTCGAG
AGAAGGTCAAGAACTTTGCCCGTCAAACACCAAGAGCCAAGCGGTCGCCTCAGATGG
GAAGGAATACCCATGTCCCCCATACAAGATCATCATCCT

> Rfc2 (FG720)_*Hygrophoropsis aurantiaca*
CCGCCTGGAACAGGAAAGACATCTACAATCCTTGCATTATCTCGGCAGTTGTTCGGGTA
CGTTTTAACTAACCTTCTTAGATGCAGAACTCCAACTTGACTTTAGCCCAGACAACTTC
CGCAACCGAGTGCTCGAGCTCAACGCCTCTGATGAGCGAGGCATATCCATTGTTAGAG
AGAAGATCAAAAACTTTGCTCGCCAAACACCAAGAGCCAAATGGTGGCTTCGGATGG
GAATACCTATCCATGTCCACCCTATAAAATCATCATTCT

> Rfc2 (FG720)_*Phanerochaete carnosa*
CCACCCGGAACAGGAAAGACTTCAACTATCCTCGCTCTGGCGCGACAACTGTTCGGGT
ATGTAACGCCTCTATGTCCAAGACGGAAGCCTCATTCTTCCCTCGGAAGGCCAGACAA
CTTCAGGTCGCGTGTTCTTGAGCTCAACGCTTCCGACGAACGAGGTATAAGCATCGTC
CGAGAGAAGATCAAGGACTTCGCTCGCCAGACCCCTCGCGCCAAGCCGCGTCTTCGG
ACGACAAAACGTACCCATGTCCGCCATACAAAATTATAATCCT

> Rfc2 (FG720)_*Agrocybe pediades*
CCCCCCGGCACAGGAAAGACATCGACAATATTGGCTTTGGCTAGACAACTCTTTGGGT
AAGCCTTCTTGAATTAATCCACTCTCAGGTATATAACCGTATACTCAGACCAGATAACTT
TAAAAACCGAGTTCTCGAACTGAACGCGTCCGACGAACGTGGTATCTCAATCGTCCGC
GAGAAAATCAAAAACTTTGCGCGTCAGACACCCAGAGCCAAGCCGTTGCTTCAGATG
GAAAGACATATCCCTGCCCTCCTTACAAGATCATCATTCT

> Rfc2 (FG720)_*Agaricus bisporus* var. *bisporus*
CCTCCTGGAACGGGCAAGACTTCGACCATCTTAGCCTTGGCGAGACAACTCTTCGGGT
AACTCACCCATCCTCAATCATCATTCAACTCATGCACTTGGGCAACCAGACCCGACAAT
TTCAAAAACAGGGTACTCGAACTAAATGCTTCAGACGAACGAGGTATCAGCATCGTTC
GAGAGAAAATCAAAAACTTTGCACGTCAAACTCCTCGAGCCAAGCCGTCGCTTCAGA
TGGCAAAACATATCCATGCCCTCCCTACAAGATTATTATTCT

> Rfc2 (FG720)_*Gymnopilus chrysopellus*
CCTCCTGGGACTGGCAAGACCTCGACGATTCTGGCTTTGGCACGACAACTCTTTGGGT
ATGTTTTCAGCTCGGCGCATACATATACTGATATAACATGCGATGGCAGGCCAGACAAC

TTCCGAAATCGCGTACTTGAATTAAACGCCTCTGATGAACGTGGTATTGCAATTGTTCG
CGACAAAATTAAAAACTTTGCTCGCCAAACACCTCGAGCCAAACTGTCGCCTCAGACG
GTAAAACATACCCATGCCCGCCTTATAAAATTATCATTCT

> Rfc2 (FG720)_*Mycena crocata*
CCACCAGGGACCGGAAAAACATCAACCATTCTCGCGCTTTCAAGGCAGCTCTTCGGGT
TCGACGTCTGCATAGGCGAACGCTCCGACTGACACCTTTCTCAGTCCTGATAACTTTCG
TAACCGAGTTCTTGAGCTGAACGCGTCAGATGAGCGCGGAATTTCCATCGTTCGCGAG
AAAATAAAGAACTTCGCTCGTCAAACACCCCGTGCCAAGCTATTGCTTCCGATGGCAA
AGAATATCCGTGTCCACCTTACAAAATAATTATTCT

> Rfc2 (FG720)_*Trametes versicolor*
CCACCAGGGACCGGAAAGACATCCACCATCCTCGCCCTTGCCAGACAACTCTTCGGGT
ACGTTACCCCACTATGCTCCAGAACGCTCGCAACTCACGCTCTCTCAGCCCTGAGAAC
TTTCGATCCCGCGTGCTCGAGCTGAACGCGTCTGACGAGCGCGGTATCTCCATCGTCCG
CGAGAAAATCAAGAACTTCGCGCGCCAGACGCCCCGCGCCAGGCCGTTTCAGCAGAC
GGCACCGTCTATCCATGTCCTCCGTACAAAATCATCATCCT

> Rfc2 (FG720)_*Coprinopsis cinerea*
CCTCCAGGAACAGGAAAGACATCCACAATCCTCGCTCTCGCCCGTGAACTCTTCGGGT
GCGCCTACCTCCCAACCTATCTCCATCCCCATCCTCCCCACAAAAACAGCCCAGACAA
CTTCCGCAACCGCGTTCTCGAACTCAACGCCTCAGACGAACGCGGTATCTCCATCGTC
CGCGACAAAATCAAAAACTTTGCCCGCCAAACACCCCGCGCCAGGCCGTCTCGTCAG
ACGGCAAAACCTACCCCTGCCCACCGTACAAAATCATCATTCT

> Rfc2 (FG720)_*Gymnopus androsaceus*
CCACCGGGCACTGGAAAGACTTCGACTATTCTAGCTCTAGCAAGACAGCTTTTCGGGT
GCGTGTCGGTCTCTAGACCTTGCTCTATGCTCATTTACTTTAGGCCAGACAACTTTCGC
AGTCGTGTTCTCGAACTTAATGCTTCTGACGAACGAGGCATATCCATTGTACGCGATAA
AATCAAGAATTTTGCTCGGCAAACTCCTCGTGCCAAGCAGTGTCTTCCGATGACAAAG
TTTATCCTTGTCCGCCCTACAAGATCATTATTCT

> Rfc2 (FG720)_*Stereum hirsutum*
CCTCCTGGAACGGGGAAGACATCCACTATTCTAGCATTGTCTCGTCAACTCTTTGGGTA
TGCAGACTGTTTTACTAGGACTTTGTGGACACTTACGTTACTCTCAAGACCAGATAATT
TCCGTTCTCGAGTCTTGGAACTGAACGCCTCAGACGAACGAGGAATCGCCATCGTACG
TGAAAAGATCAAGAACTTCGCCCGACAAACTCCACGCGCCAAGCGGTCTCGTCAGAC
GGCAAGGCATACCCATGTCCGCCCTACAAAATTATTATTCT

> Rfc2 (FG720)_*Fomitopsis pinicola*
CCCCCTGGTACCGGCAAAACGTCTACCATCCTTGCATTGTCAAGACAGTTGTTTGGGTG
CGCAATGATGTTTGACCTCCTAGGCTGAACTTAGACACGTCTTGCAGCCCAGATAACTT
CCGTTCAAGAGTACTTGAACTCAATGCGTCTGACGAACGTGGTATCGCCATTGTACGTG
ACAAGATCAAGAATTTCGCGCGACAAACACCACGCGCCAGGCTGTTTCTTCTGACGGA

AAGACATACCCTTGTCCGCCGTACAAGATCATCATCCT

> Rfc2 (FG720)_*Amanita muscaria*
CCGCCTGGAACGGGCAAAACATCTACAATTCTGGCATTAGCTCGTCAACTGTTTGGGT
GCGCGTTGTCCAGTAGTAAAGTGAAGTAAACTGATACCGTTAAAAGTCCAGAGAATTT
TCGGAGTCGTGTCCTTGAATTGAACGCGTCAGATGAACGTGGTATCAGCATCGTCCGC
GAGAAGATCAAAAATTTTGCGCGACAGACGCCTCGCGCCAGGTTGTCTCCTCGGACG
GCAAGGTATATCCCTGTCCCCCTTATAAAATCATCATACT

> Rfc2 (FG720)_*Lactarius quietus*
CCTCCCGGGACAGGCAAAACCTCCACCATCCTCGCCCTCGCACGTCAGCTGTTCGGGT
GGGTCTCCACTCGAACTACACGGCGTTCGTACTTGACGTCCACCAGGCCTGATAACTTT
CGATCGCGCGTCCTAGAATTAAACGCGTCTGACGAGCGCGGCATATCCATCGTACGCGA
AAAGGTCAAGAACTTTGCTCGTCAGACACCGCGCGCGAGGCTGTGGCATCAGACGGA
AAGAAGTACCCATGTCCGCCCTACAAGATTATTATCTT

> Rfc2 (FG720)_*Auricularia delicata*
CCTCCAGGAACGGGCAAAACAAGCACAATCCTTGCACTTGCTCGACAACTATACGGGT
GCGCGTCTTCGCGTACAAATTGACTGGCTCATACGCCCTCTCGCTCTAGGCCCGACAAC
TTTCGTACGCGCGTGCTCGAGCTCAACGCCTCCGACGAGCGTGGAATCACCATCGTCC
GCGAGAAAATCAAGGACTTTGCACGCCAAACGCCGCGCGCAACGTTGCCTCGAGCGA
CGGACAAACATATCCTTGTCCACCATACAAGATTATCATCCT

> Rfc2 (FG720)_*Marasmius fiardii*
CCTCCCGGAACAGGGAAGACGTCGACAATACTTGCGCTGGCAAGGCAGCTTTTCGGG
TCCGTGGCTCTTGACTTTTGGGTACGTATTTGCTCATCTCTTTTCTCAGACCAGATAACT
TTCGAACACGCGTTCTTGAACTCAACGCATCGGATGAGCGTGGCATATCTATCGTTCGG
GAGAAGATAAAGAATTTCGCGCGTCAAACGCCTAGAGCAAGACGGAGTCCTCCGATG
GAAAGGAGTATCCCTGTCCGCCATATAAAATTGTTATTCT

> Rfc2 (FG720)_*Tricholoma matsutake*
CCCCCAGGGACAGGCAAGACTTCCACCATCCTTGCCTTGGCACGACAACTCTTTGGGT
ATGCTCCACATTACCTATTTTAGGATTACTGTTGAATGGACATACAGTCCGGACAATTTC
CGCGAAAGAGTTCTAGAATTAAATGCATCAGATGAGCGTGGAATTGGCATCGTCCGGA
ACAAAATCAAGGATTTTGCACGCCAAACGCCCCGCGCCAAAAAGCCTCTGATGGCAA
ATCATACCCATGCCCCCCTTACAAGATCATCATTCT

> Rfc2 (FG720)_*Boletus edulis*
CCCCCAGGAACAGGCAAGACATCCACGATTCTTGCGTTGTCGCGACAGCTATTTGGGT
GCGTAATCTGTTGGCTCCTATCGAGTGTTTCATTTTGGTTGTTTTCCAGTCCTGACAACT
TCCGCGAGCGGGTACTTGAACTGAATGCATCAGACGAGCGGGGTATTTCCATTGTCAG
AGAGAAAATAAAGGTATTCGCTCGACAGACACCGAGGGCCAAAAAGTTGCTTCGGAT
GGCAACCCCTATCCATGCCCACCCTACAAGATTGTCATTCT

> Rfc2 (FG720)_*Dacryopinax primogenitus*
CCACCGGGGACAGGCAAGACGAGCACCATCCTGGCCCTCGCTCGTCAACTATTCGGGT
ACGCCGTTCATGTTCCCCACCATGTATGCTCACTTCCCATAGTCCTGAGCTCTTCCGTTC
CCGCGTGCTGGAACTGAACGCCTCCGACGAACGAGGCATCACCGTCGTCCGAGAGAA
GATCAAGAACTTTGCACGTCAGACCCCACGCGCAGCGGACGATGAGGCGTCTAAGGG
ATACCCCTGCCCTCCGTACAAGATTATCATACT

> Rfc2 (FG720)_*Calocera cornea*
CCTCCCGGGACGGGCAAGACCAGCACCATCCTCGCGCTCTCACGAGAGCTGTTCGGGT
ACGCCCAATCTCACCCGTACTGTGCTGCCACTGACCGAAGCAGACCCGAGCTCTTCCG
CTCACGCGTGCTCGAGCTCAATGCATCCGACGAGCGGGGAATTTCCGTCGTCCGCGAG
AAGATCAAGAACTTTGCGCGCCAGACGCCGCGCGCGGCGGACGCCGAGGCTGCCCGG
GGTTACCCCTGCCCCCCGTACAAGATCATCATCCT

> Rfc2 (FG720)_*Ramaria acris*
CCTCCCGGGACGGGGAAGACTTCTACTATACTGGCTTTGGCTCGACAACTGTTTGGGT
GAGCATCCATTGGGATTAACAATATTGTTTGATATTGAATAAGTCCCGACAACTTTCGCA
ATCGCGTACTGGAGCTAAATGCGTCAGATGAACGGGGTATCACGGTTGTCCGCGAAAA
AATCAAAACATTCGCCAAGCAAACCCCAAGAGCTGCCGTCAATACGGACGAGGGGCG
ATACCCGTGTCCACCTTATAAAATCATTATTCT

> Rfc2 (FG720)_*Gautieria morchelliformis*
CCACCCGGCACAGGGAAAACGTCTACGATCCTTGCCTTGTCTCGACAACTTTTTGGGT
GAGCAGTGATGCATGTGGAACCGATAACATCATGCTTCTGATTGTTAGTCCGGACAACT
TCCGTAATCGAGTTTTAGAGCTCAATGCATCAGATGAGCGGGGCATCACCGTCGTCCGC
GAGAAAATCAAAACGTTCGCGAAACAGACTCCCAGGGCTGCCGTTTCTTCGGAAGGA
AAAACATTTCCATGCCCACCCTACAAAATCATTATCTT

> Rfc2 (FG720)_*Agaricostilbum hyphaenes*
CCGCCCGGCACAGGCAAGACCTCTACCATCCTCGCTCTAGCCAAACAGCTCTTTGGCC
CTACCGTCTTCAGATCACGCGTGCTCGAACTCAACGCATCAGATGAAAGAGGTATTTCT
GTCGTGAGAGACAAGATCAAGAATTTTGCCAAGATTGCACTCAGCACCCGCCGGCTAG
TGAGAAGGACTATCCTTGCCCACCTTACAAGATCATCATTCT

> Rfc2 (FG720)_*Asperigillus triticus*
CCTCCCGGTACGGGAAAAACCTCCACGATCCTCGCCCTCGCCAAATCCCTCTTTGGAC
CGGCCCTCTACCGCTCCCGCATCCTCGAGTTGAACGCTTCCGACGAACGAGGAATTGG
TATCGTTCGCGAAAAAGTCAAGGGCTTCGCCCGAACGCAATTGAGCCAGGGGTGGGC
AAAGAATACTTCGAGCAGTACCCGTGTCCTCCGTTCAAGATCATCATCTT

> Rfc2 (FG720)_*Neurospora crassa*
CCTCCAGGAACGGGCAAGACCTCGACCATCCTTGCTCTCGCCAAGGAGCTCTACGGCC
CCGAACTCATCAAGTCCCGCGTCCTAGAGCTCAACGCCTCCGATGAGCGTGGTATCTC

CATCGTCCGCGAAAAGGTCAAAGACTTTGCTCGCATGCAGCTTACCAATCCCTCCGCG
GCTTACAAGGCGCGCTACCCATGTCCGCCCTTCAAGCTTATCATCCT

> Rfc2 (FG720)_*Rhizopus microsporus*
CCTCCTGGTACTGGTAAAACTTCAACCATCTTGGCATTAGCACATGAACTCTATGGACC
TCAGCTGATGAAATCTCGTGTACTTGAATTAAACGCTTCGGACGAAAGAGGTATCGCA
GTTATTCGTGAAAAAGTAAAGGATTTCTCAAGAACGACAGTAACAAGAAAGTGAGGT
AATGACACTAAAAAGGATATCCTTGCCCTCCATACAAGATTGTTATTTT

> Rfc4 (FG761)_*Boletus edulis*
AACGTATTCAAAGTTTGTGACCAACCCCATCCGGTAATAGTCCAAAGTATCATCCGCGC
GTGCATCAAGGGCGACATCAATAGTGCAATGGAAAAGTTAAACGAGCTGTGGGAACA
AGGGTACAGTGCAGTGGACATCGTCGTCACAATCTTCAGGGTGACAAAGACTTTTGAT
GAGTATGTCCAATGAACGATTGGCGGAACTATGGTTGATTCTTTTCATTAGACTACCGG
AATATACTAAACTGGAGTATATCAAAGTACGAATAATTTCTTTGGTTTTGGTCATAAGAC
TCACTTTGTGCAGGAAATTGGATTTACACATATG

> Rfc4 (FG761)_*Trametes versicolor*
AATGTGTTCAAAGTCTGCGACCAACCACATCCAATCACCGTGCAAGCCATGATACGCG
CCTGCCTGAAAGGTGACATCGAAGGCGCGATGGATAAGCTGGACGAGCTTTGGGACC
AAGGATATAGTGCGGTCGACATTGTGGTCACGGTCTTCAGGGTGGTCAAGACGTTTGA
CGAGTATGTCTACCTGCGAACAACGCGACCACTAGCTGACACTGGGCCCTATAGGATTC
CGGAGTACACAAAACTTGAGTACATCAAGGTAAGGATTTCGGAAACCTGGACGACGTT
GCAAGCTAATGGTCATTTCAGGAGATTGGCTGGACGCACATG

> Rfc4 (FG761)_*Laccaria bicolor*
AATGTATTCAAAGTGTGCGACCAACCGCATCCTATCATCGTACAAGCTACGATTCGGGC
TTGTTTAAAAGGTGACATTGACGGTGCCATCGAAAAAGTGAATCAACTTTGGGAGCAA
GGGTACAGTGCAGTGGATATTGTGGTGACCATCTTCAGGGTTGTGAAGATCTTTGACG
AGTAAGTGGCTGCGTTAGTCTCTTTTCCTGTGCTGACGGTGATAGAATGCCGGAGTACA
CCAAACTGGAATACATCAAGGTTCGTGAGAATCCATCCCTATCGGTTGAGTTAGATTTG
TGATATGCTTCTAGGAAATTGGGTTCACACATATG

> Rfc4 (FG761)_*Gautieria morchelliformis*
AATGTATTCAAGGTTTGCGACCAACCGCATCCAATCGTCGTGCAAGCCATGTTACGCTC
GTGTCTCAAGGGCGAAATTAGCCCTGCTATGGAAAGGCTTGATGAATTATGGAGCCAA
GGGTATAGCGCTGTTGACATCGTGGTAACCATGTTCAGGGTAGTGAAGATCTTTGACGA
GTGAGTGTTACCTTGCAACGTGTTGCAAAGGACGCCGCGTTCTACACGTTGCATAAAT
GGGAGCTGACCTTACCTCCAGGTTGCCAGAATATACAAAGCTAGAATATATCAAAGTTA
GTGGGCTTTTAGAGATTTCCTGATATTTGCTTTTAAACCTTTCCCCCAGGAGATCGGATG
GACACACATG
> Rfc4 (FG761)_*Ramaria acris*
AACGTGTTCAAAGTGTGCGACCAACCGCATCCAATTGTTGTTCAAGGTATGTTGCGGG
CGTGTCTAAAGGGTGAAATCAACCCAGCTATGGAAAAGCTCGATGAGCTTTGGGGCCA

AGGTTACAGTGCCGTGGATATTGTGGTGACAATGTTTAGAGTGGTGAAGGTGTTTGAC

> Rfc4 (FG761)_*Gymnopilus chrysopellus*
AATGTTTTTAAAGTCTGCGACCAACCACATCCGATCATAATACAAGCAACAATACGTGC
TTGCTTGAAGGGCGACGTTGATACTGCAGTCGAAAAATTAAATGACCTGTGGGCTCAA
GGGTATAGTGCTGTTGATATTGTTGTGACTCTCTTCCGTGTTGTCAAGACATTTGAC

> Rfc4 (FG761)_*Agrocybe pediades*
AATGTATTCAAGGTTTGCGACCAGCCGCATCCCATCATCATTCAGGCGACCATCCGTGC
ATGCTTAAAGGGCGACGTCGACACGGCGGTGGAAAAATTAAACGATCTATGGGCACAA
GGTTATAGTGCCGTTGATATCGTTGTCACTATATTCCGGGTGGTGAAGACCTTCGATGAG
TAAGTGAATCAACAAATGGCAGTTGCAGTACCTCATGATCCTCTAGAATGCCGGAGTAT
ACGAAACTTGAATATATCAAGGTTGGTCTAGGGTTGACTTGCAGCAGTCGACGTAATAA
AGACTGATCAACTTCTCTACAAGGAAATTGGTTTTACGCACATG

> Rfc4 (FG761)_*Mycena crocata*
AATGTCTTCAAAGTCTGCGACCAACCCCACCCCATCATCGTCCAGGCTATCATCCGCGC
CTGCGTAAAAGGGGATATCAACACTGCTATGGAGAAGCTGAATGAGCTTTGGGGGCAG
GGATACAGTGCTGTCGACATCGTAGTGACGATATTCCGGGTTGTTAAAACTTTTGAC

> Rfc4 (FG761)_*Tricholoma matsutake*
AACGTTTTCAAGGTGTGCGACCAACCACACCCTATCGTTGTACAGGCCACAATCCGGT
CATGTATGAAAGGAGATATCGACGGTGCAATGGAGAAACTGAACGAGCTTTGGGAGCA
AGGTTACAGTGCCATTGATATCGTTGTGACTGTTTTTCGTGTTGTGAAGACTTTTGAC

> Rfc4 (FG761)_*Pluteus cervinus*
AATGTGTTCAAGGTTTGTGATCAGCCACACCCCATACTGATACAAGCCACCGTCCGAA
GCTGTATGAAAGGTGACATCGAAGGCGCGATGGAAAGGTTAAACGAGCTGTGGGACC
AAGGGTATAGCGCAGTCGATATTGTGGTGACCTTGTTCAGAGTGGTGAAGACGTTTGAT

> Rfc4 (FG761)_*Amanita muscaria*
AACGTATTCAAAGTTTGCGACCAGCCGCATCCTTTGACTGTGCAAGATGCTATCCGTAG
TTGCATGAAGGGGGACATTGAGGGCGCGATGTCAAAGCTCGATCTACTGTGGGATCAG
GGCTATAGCGCCGTGGACATTGTTGTCACCTTCTTCAGAGTCGTCAAGACATTTGATGA
GTGAGTGCCATGGATTGACACATTCTTGCGACTCTAACGTGAGATTCAGGATTCCCGAG
TACACTAAACTAGAATACATCAAGGTTTCGAGAATTCGTCTGTGAGAGTTTTTTGCACT
ATTAAAAGCTTAACAGGAAATCGGGTTCACACATATG

> Rfc4 (FG761)_*Marasmius fiardii*
AACGTCTTCAAAGTGTGCGACCAGCCACATCCAATTATCGTACAGGGGCTCGTCAGGC
AATGCATCAAGGGTGATGTCGATAAAGCTATGGATAGGCTCAATGAGCTGTGGGATCAA
GGATATAGTGCTGTGGATATCGTGGTGACGATATTCAGAGTGGTCAAGACTTTTGATGA
GTGAGCGTTATGTGGTTACTCCCGCTATAAATGCACTTACTTTCGCTCTCTTTTAGGATA
CCGGAATACACGAAACTAGAGTTCATAAAGGTTGGCATCGACAGCGGCGAATGCGTAT

CATGGACTTATTGCATATCTATAGGAGGTTGGATTCACGCATATG

> Rfc4 (FG761)_*Fomitopsis pinicola*
AATGTCTTCAAAGTTTGCGACCAGCCGCACCCCATTGTCGTTCAAAGCATCATCCGGG
CATGCCTTCAGAGCGACATTGATGGGGCGATGGAGAAGCTCAATGAGCTCTGGGATCA
AGGCTATAGTGCGGTTGACATTGTAGTCACAGTATTCCGGGTCGTGAAGACCTTTGAC

> Rfc4 (FG761)_*Calocera cornea*
AACGTGTTCAAAGTGTGCGACCAGCCGCATCCCGTGCTCATCCAGGGGATGCTGCGCA
GCTGCCACAAAGGCGACATCGGGAGCGCAATGGAGAAGCTGGACGAGCTGTGGGATC
AAGGGTATAGCGCGGTGGATATTGTGGTGACGCTGTTTAGGGTGACCAAGACGTTCAA
TGAGTGAGCACTGCGTTACCAATATCATGCGCTTTGACTGAGTGGGTATCAGCTTGCCG
GAATACACAAAACTGGAGTACATCCGGGTACGCTTTGTGCCACAAAATTTGGCGATAA
CACGGACTGACTGGATTCACAGGAGATTGGATGGACCCATATG

> Rfc4 (FG761)_*Dacryopinax primogenitus*
AATGTCTTCAAGGTGTGCGATCAACCGCATCCTGTGCTCATACAAGCGATGCTCCGCAG
TTGCCACAAGGGCGATGTTCAGGGCACGATGGACAAGCTAGATGAGCTATGGGGTCAG
GGTTACAGCGCGGTGGATATTGTGGTGACTCTGTTCAGGGTTACTAAGACGTTCAAT

> Rfc4 (FG761)_*Gymnopus androsaceus*
AATGTATTCAAAGTCTGCGATCAGCCTCATCCCATAGTAGTTCAAGAAATGATCAAGCT
GTGCTCGGAGGGAAAGGTGGACGCCGCGATGGACAAGCTCACTGACTTGTGGGAGAG
TGGTTACAGTGCAGTCGACATTGTCGTGACGATTTTCCGTGTAGTAAAGACAGCGGAC
AAGTAAGTTTTGCGCGATCCCTCTTTATTTTCTTGTTTATTCAGGACGTCCACATAGTATT
CCCGAGTATACGAAGCTGGAATACATCAAGGTAATTCCCGTGTTACTAACAGTCCCGTA
TTTACTCACAATATCTGTAGGAAATCGGCTTCACACACATG

> Rfc4 (FG761)_*Fomitiporia mediterranea*
AACGTCTTCAAAGTCTGCGACCAACCACATCCTATCTTAGTACAAGCTATGATAAGGGC
TTGCATGAAAGGCGATATAAATACTACGATGGAGAAACTTACTGAGCTTTGGGATCAAG
GGTATAGTGCTGTGGATATAGTTGTGACTATCTTTCGCGTGGTAAAGACCTTTGAT

> Rfc4 (FG761)_*Lactarius quietus*
AATGTCTTCAAAGTTTGCGACCAACCCCATCCCATCGTCGTCCAGAGTATGATCCGTGC
ATGCTCCAAAGGCGACGTCGAAAGTTCCATAATTAAGCTTCGAGAGCTCTGGGACCAA
GGTTACAGCGCCGTGGATATCATTGTAACCATATTTCGCGTCGTGAAGACCTTCGATGA
GTACGGCGCCTCCACCTTCGGCTCCCCACTCACACACTTACCGCATTTACGTTAGGCTG
CCGGAATACACAAGATTGGAATATATAAAGGTAGCGATCGGCCAACTGATTCCCTTACG
TAGGATGCTTATTTTTGGCCCAGGAAATCGGCTTCACACACATG

> Rfc4 (FG761)_*Schizophyllum commune*
AACGTCTTCAAAGTTTGCGATCAGCCGCACCCCATCACCATTCAGACCATCATCCGATG
TTGCTTGAAGGGCGATATTGACGGTGCGATGGTGAAGCTGACGGAGCTGTGGGATCAG

GGGTACAGTGCGGTGGATATTGTAGTGACGTTGTTCAGGGTGGTGAAGAACTTTGACG
AGTGCGTAACCTGCCCGAATACGAAGGGGGCTTCTGCTGACACGATACAGGATTCCGG
AGTACACAAAACTGGAATATATCAAGGTACGTGATGACCAGCACGCATCTCGCAGTTCT
CGTGCTGACGTACGATAGGAAATCGGCTTCACGCATATG

> Rfc4 (FG761)_*Agaricus bisporus* var. *burnettii*
AATGTATTCAAAATTTGTGATCAACCCCACCCGATTGTCGTACAAGCTGCCATTCGGTC
ATGTCTAAAAGGAGACGTGGATGAGGCTCTAGGAAAAATAAAGGAACTCTGGGACCA
AGGGTATAGTGCTGTGGATATTGTGGTAACCTTGTTCAGAGTGGTGAAGACGTTTGATG
AGTGCGCATCGTTTCTGAACGCGATTCGGACTATTCGCTGACAATGTTGTAGGGTACCA
GAGTACACCAAACTGGAATTTATCAAGGTGGGTAGTGTGTTGGCGAGGACGAATTGAT
AATTAAATGTTTGCATTGTAGGAAATTGGGTTTACGCATATG

> Rfc4 (FG761)_*Phanerochaete carnosa*
AACGTTTTCAAGGTCTGCGACCAGCCACACCCTGTGGTGGTACAAGCCATGATTCGGT
CGTGTTTGAAGGGAGACGTTGAAAATGCGATGGAGAAGCTTAACGAACTCTGGAATCA
TGGCTATAGTGCCGTGGACATTGTGGTCACGATTTTCCGCGTCGTGAAGACGTTCGACG
AGTACGTCTCCGTTCGTCAGCAGATACTTTATGCATTGACGACGTATCTAGGATACCAG
AATACACGAAACTTGAATATATCAAGGTCCGTGCTTCCATTCCGGAGATGGACCCCAAT
TGACATCTGATCTTCTTGCATAGGAGATCGGCTTCACGCATATG

> Rfc4 (FG761)_*Serpula lacrymans* var. *lacrymans*
AATGTCTTTAAGGTGTGCGACCAACCACATCCAGTAATCGTGCAGAGCATTATCCGCGC
CTGCCTGAAGGGTGATATCGATGGGGCTATGGGGAAGCTGAATGAACTCTGGGAGCAA
GGTTACAGCGCAGTTGATATTGTAGTGACGGTGTTCAGAGTCGTGAAGACATTTGACG
AGTAAGTGCAAAGTACATCACGATGGCCGACAACCACGTACCAACTAATATCTTTCAGA
TTACCTGAGTATACCAAATTGGAGTACATCAAGGTAGATTAACTCTTTTTGTAGTGATAG
TTCATCATCCTGACCCCCCAGCATCAAGGAAATTGGGTTCACGCACATG

> Rfc4 (FG761)_*Stereum hirsutum*
AATGTCTTCAAAGTTTGCGATCAGCCACATCCGATCATCGTCCAGACCATCATTCGCGC
TTGTATGAAGTCAAATGTTGACCTCGCGATGGAGAAGTTGTCGGAGCTCTGGGGACAA
GGTTATAGCGCAGTAGACATTATTGTCACGATTTTCCGTGTCGTCAAGACATTTGATGA
GTGCGTCTCCATGTCGTTCATCCGAGCTCGTTTGGCTGATTAGCACTGCGTAGGCTCCC
TGAATATACTAAGCTCGAATATATCAAGGTTCGCCTCCCCCTATTCTGCGACTGAGCATA
GACTGATGCGATCTTATTAGGAGATCGGCTTTACCCACATG

> Rfc4 (FG761)_*Coprinopsis cinerea*
AATGTGTTCAAAGTCTGCGACCAGCCACACCCTATTGTTGTGCAATCCATGATTCGAGC
TTGTCTAAAAGGAGAGATCGACCCTGCACTCGAAAAACTACACGAACTGTGGGATCA
GGGGTACAGTGCTGTGGACATTGTCGTTACTCTTTTCAGAGTGGTCAAGACCTTTGAC
GAGTTGAGTTGTTTCAAGGTTTTCCATTTTCGTCCTAAAGCCGTTAATTAATTATCCTTG
CAGCATGCCCGAGTATACGAAACTGGAGTATATCAAGGTCAGTTCCAGCGCCGCTCAA
AGATAGTTAATTATGCTGAGTGAGGTTTGGCAGGAAATTGGGTTTACGCACATG

> Rfc4 (FG761)_*Auricularia delicata*
AATGTGTTCAAAGTCTGCGACCAGCCACATCCAACGATGGTGCAGAAGATGATGAGGT
CCTGCAAAGAGGGAAACATCAACGACGCCATGGACCACTTGACGGCACTGTGGAGGC
AAGGGTACAGCGCCGTCGACATTGTCGTGACAATTTTCCGAGTGGTCAAGGTTTTCGA
CGAGTGCGTTCTCCGCATGCGGGTTCTCCTTAAGCTGACCCCTTGCTAGGCTACCGGAA
TATCTGAAGCTCGAATTCATCAAGGTGCATTTATCATAAAGCGATAATGGCTATGACCGG
ACTGACCGACGTGCAGGAAATTGGCTTTACTCACATG

> Rfc4 (FG761)_*Agaricostilbum hyphaenes*
GCCGTCTTCAAGGTCTGCGATCAACCGCACCCGACTGTCGTGCAAGATATGCTGCTCG
CTTGCTACAAAGGAGACATCACACTTGCTATGGATCGACTGGATGGATTGTGGAAGCA
AGGGTACGCGGCGGTGGATATCGTCAGCACCATCTTTAGAGTGGCCAAGACCTTGCCA
GATCTACCGGAGTACATGAAGTTGGAGTTCATCAGGGTATGTGTTGAGTCTCAGTGTCG
GTACGAAAGAGACTGACGGCTTGAGGTCCAACAGGAGATTGGGTTTGCACATATG

> Rfc4 (FG761)_*Asperigillus triticus*
AACGTGTTTCGAGTTGTCGATAGCCCTCATCCCATCAAAGTGCAAGCTATGATCAAGGC
TTGCTGGGAAGGCAAGGTCGATGCTGCGCTGGAGACGCTTAATGAGCTATGGTATGTG
ATGTTTTTTATTGCAAGCTTCGAGGTCATTTGTCACTGACATGTGCAGGAACCTGGGGT
ACTCCTCGCACGATATCATCAGCACGATGTTCCGAGTCACCAAGACCATTCCGACATTG
TCCGAGCATTCTAAATTGGAGTTCATCCGGGAGATTGGCTTCACGCACATG

> Rfc4 (FG761)_*Neurospora crassa*
AATGTCTTTAAAGTTGTGGATAGCCCGCACCCGATCAAGGTGCAGGCGATGCTCAAGG
CCTGCTACGAGGGTAATGTTGATGCGGCTCTGGACGGGCTGCGGGAGCTGTGGGACCT
GGGGTATTCCAGTCATGATATTATCAGCACCATGTTCAAGGTCACAAAGACGATTCCGA
CGCTGAGCGAGCACGCTAAGCTCGAGTTTATCAAGGAGATTGGGTTTACGCATATG

> Rfc4 (FG761)_*Rhizopus microsporus*
AATGTCTTCAAGATTTGTGATCAACCTCATCCTATTGTCATTCAGCATATTCTCAAATGC
TGCTCTGAAGGAAACTTGAATGAATCTGTCAAGTGCATGGAAGAATTATACTATTTAGG
ATATTCTTCACTTGATATCATCACCACAATCTTTCGTGTAGTGCGAAGCTATGATGAACT
AGATGAAGGATTGAGATTGGACTATTTGAAAGAGATTGGTATGACACATATG

>Rio2 (MS481)_*Agrocybe pediades*
CGGCTAGCAGCAGAAAAAGAGTATGCTTTTATGAAGGTAAGGCAGTAACATATATTCTG
ACGCCTTGTCGCTACGTTTAGATTCTACATGAGCATAATTTCCCTGTACCTCGACCTATC
GACCAAGCACGCCACACCATTCTCATGGAATTCATTGATGCCTACCCATTGTGAGAATC
TTGCTTTCTTGCACGTCTTACGGGAATAGACGCCAAGTTTCAGAAGTAGCAAATCCCG
GAAAGCTTTACTCTGAATTAATGGACGTCATCGTTCGTTTTGCTCAAGCAGGACTTATC
CATGGCGACTAC

> Rio2 (MS481)_*Trametes versicolor*

CGTCTCGCTGCCGAGAAGGAATACGCCTTCATGAAGGTAGATTATCACCGCTGCCGCA
CCTTCGTTACCAAGCCCGCTAGGTGCTCTACCAACACGATTTCCCTGTCCCACGCCCCG
TCGACCAGGCGCGGCATTGCATCCTAATGGGATTCATCGATGCATACCCTCTGTAAGCT
TTCGGTTCTATCCAGCTTCTTTCCAATACAGTCGGCAGGTTGCGGACATCCCGTCTCCA
GGGAAACTCTACTCGGAGCTTATGGACCTTATTGTCCGGTTTGCGCGCGCAGGGCTCAT
TCACGGCGACTTC

> Rio2 (MS481)_*Coprinopsis cinerea*
CGTCTCTCCGCGCAGAAAGAATATGCCTTCATGAAAGTATATACGTCTCTCTTCTCGTCC
TTGAGAATTCACTCGATTAGGTGCTTTATGAACATGGTTTCCCCATACCAAAGCCCATC
GACCAAGCCAGACATTGCGTACTGATGGAATTCATAAATGCCTACCCGTTGTAAGATTA
TTTTCCCTATGAGATTCACATGACAATAGGCGACAGATATCAGAAGTGCCGTCTCCAGG
AAAGCTCTATTCCACGTTGATGGACCTCATCGTCCGCTTTGCACAAGCAGGCCTTATCC
ACGGCGATTTC

> Rio2 (MS481)_*Tricholoma matsutake*
CGTTTAGCAGCACAAAAGGAGTGGGCTTTCATGAAGGTAGCTCTGATTTATGAATATGG
TATCGGCACTTACATTTGTAGGTGTTGCATGAACATGGGTTTCCTGTTCCTCAGCCGATT
GACCAAGCAAGGCATTGTATTTTGATGGAATTCATCGATGCCTATCCTCTGTAGGCCCTT
TTGGCCTCGCTTTTCATCACGTAAACAGCCGTCAGATCTCCGAAATACCTTCCCCGGGG
AAGCTCTACTCAACGCTCATGGATTTGATCGTGCGATTTGCCCATGCAGGACTAATCCA
TGGTGACTTT

> Rio2 (MS481)_*Schizophyllum commune*
CGTCTTGCTGCCCAGAAGGAATGGGCTTTCATGAAGGTCGGTCGATTGACGGCGCTCG
GGCTCCCTGACAGTGCTCGAAGGTCCTCCATGAGCACGACTTCCCAGTACCCAAGCCC
ATCGACCAAGCGAGACATTGCATCCTGATGGAGAAAATTGACGCCTACCCATTGTACGT
CCATCTCTCGTCGTTCGGTCAAGCGCCCGCAGACGACAGGTTTCCGAAGTACCGAACC
CAGGAAAGCTATACTCGACTCTCATGGACCTCATCGTGCGCTTCGCTCAAGCAGGCCT
GATCCACGGTGACTTT

> Rio2 (MS481)_*Auricularia delicata*
CGTCTAGCCGCGCAAAAGGAGTGGGCGTTCATGAAGGTACGCTCGTTCCATGTCAATG
GATCCTTCGCTGAATGCGAAAGGTCCTGCATGAACACGGCTTCCCCGTCCCTACACCA
ATCGACCAAGCGCGGCATTGCATCCTTATGGAACTCATTGACGCGTATCCTCTGTAAGT
CGTCCCACCAATCCTCACCACAGTCCTTGTAGACGACAAATCGACGAGCACCCGAACC
CTGGCCAGCTCTACTCGACACTCATGGACCTCATCGTGCGCTTCGCTCAGGCGGGGCT
GATCCATGGCGACTTC

> Rio2 (MS481)_*Mycena crocata*
CGTTTGGCGGCGCAAAAGGAATGGGCTTTTATGAAAGTAAGGAGCTCCTTGCTAATAA
TCCCCAATCATAATATTCCTAGGTGCTTCATGAACATGACTTTCCAGTTCCCCGACCCAT

CGATCAAGCAAGGCATTGCATACTGATGGAGTTCATCGATGCGTACCCTCTGTAAGTTT
TCAGTATTTTACTGGATCAAACTCTACTAGCCGGCAGATAGCAGAAGTCCCCTCCCCAG
GAAAACTATATTCTACGTTAATGGATCTAATTGTGCGGTTTGCACACGCAGGACTCATCC
ACGGTGACTTT

> Rio2 (MS481)_*Pluteus cervinus*
CGTCTGGCAGCACAGAAGGAGTGGGCCTTCATGAAAGTATGGGACTTTGTGGTATCTT
TGCCAGCTTCTGATCAAAATAGGTGCTACATGAGCATGACTTTCCCGTGCCCAGACCAG
TGGACCAGGCGCGTCATTGTATACTCATGGAGTTCATTGACTCATATCCGCTGTAAGGA
AATCACTTGCCTTTTGATTGACCATCTGCAGGCGTCAGATCGCGGACATACCATCACCG
GGGAAGTTGTATTCAACCCTTATGGACCTGATCGTCCGGTTTGCTCAAGCGGGGCTGAT
ACACGGCGACTTC

> Rio2 (MS481)_*Laccaria bicolor*
AGATTAGCAGCTCAAAAGGAGTGGGCTTTCATGAAGGTTTGAGATGCAAGTTTGAAGC
GACGAATATCTAAGGCCTCTAGATCTTGCACGAGCATAATTTTCCGGTACCCAGGCCGA
TCGACCAAGCAAGGCATTGTATTTTGATGGAATTTATAGATGCATATCCCTTGTACGAAT
TTGACAATTCTTTCAATAACAGACAATTAGACGACAGGTTTCGGATGTAACCTCACCGG
GGAAGCTGTATTCGTCCCTGATGGACATAATCATCCGATTCGCCCATGCAGGCTTGATTC
ACGGCGACTTC

> Rio2 (MS481)_*Gymnopilus chrysopellus*
CGATTATCTGCCCAAAAGGAATGGGCGTTCATGAAGGTTGGTTTCCTTTTCCTAAAACG
AGCCTTTGCTGAATCTGTCAGATTCTCCATGAACATGGGTTTCCTGTGCCTCGTCCAAT
TGATCAAGCACGCCACTGTGTCTTGATGGAATTCATTGACGCATATCCTTTGTACGGGC
ACCTTTGTTCTCATATATGACAACATTCAGACGTCAAGTATCGGAAGTGGAAAATCCAG
GCAAGCTGTACTCGAGTCTCATGGATGTCATCGTTCGATTTGCCCAAGCTGGCCTGATT
CATGGAGATTAC

> Rio2 (MS481)_*Gymnopus androsaceus*
AGATTAGCTGCACAAAAAGAGTGGGCTTTTATGAAGGTAAGGATAACCCTCTAATCATC
TGGTGGCTTCATATGACATAGGTTCTACACGAGCATGGCTTCCCTGTTCCGACACCCAT
TGATCAAGCGAGACATTGTATTCTTATGGAATTCATCGATGCATACCCTCTGTCAGTGCC
CAAGCTGCTCTCAGTCCCCGGTCCCTTAGTCGTCAAATAGCTGACGTCCCTTCTCCCGG
CAAGTTATATTCCTCACTCATGGATCTTATCGTGCGGTTTGCACGGGCGGGTTTGATACA
CGGCGATTTC

> Rio2 (MS481)_*Marasmius fiardii*
AGGCTAGCAGCCCAGAAAGAATGGGCGTTTATGAAGGTGACGGATATGTTTCCGTTGC
GGCCCATTGACACGACGATCAGGTGTTATATGAGCACAACTTCCCAGTCCCAAAGCCC
ATTGATCAAGCCAGACACTGTATATTAATGGAATTCATCGACGCATATCCTCTGTAAGTA
TCAAGTTTTTATTTTTCCTGAATGGGAATAGCCGCCAAATAGACGATGTTCCGTCTCCG
GGGAAGCTATACTCCTCCCTCATGGATCTCATTGTGAAATTTGCTCGAGCTGGACTCAT
TCATGGAGACTAC

> Rio2 (MS481)_*Serpula lacrymans* var. *lacrymans*
CGTCTGGCTGCACAAAAGGAATGGGCCTTCATGAAAGTTCGACCCGTTCCTTTCACTT
CATTTCTGACTTAAAAAAATAGGTGTTATACGATCATGACTTCCCCGTCCCACGTCCCAT
AGACCAAGCAAGACATTGCATTCTCATGAGCCACATCGACGCATACCCATTGTAATGCT
CCTTACCCCTCTCAGTCCCTACCCCCACAGACGCCAAATAGCTGAGGTTCCCAATGCCG
GAAAACTTTACTCCACACTGATGGACCTCATTGTCCGGTTTGCCCGCGTAGGGCTAATA
CACGGCGACTTC

> Rio2 (MS481)_*Agaricus bisporus* var. *burnettii*
AGACTAGCCGCTCAGAAGGAATGGGCTTTTATGAAGGCCGGTGAACTAGACACTCCCA
ATCAAGCTGACCCGATCGACAGGTGTTGTACGACAATGGCTTCCCGGTCCCTAAGCCC
ATTGACCAAGCACGGCATTGCATTCTCATGGGATATATCGATGCTTATCCGCTGTGAGTT
CGCTATCCCAGAGCAGGCGCTTCTTGGTCAGGCGTCAGGTCGCTGAGCTACCCTCTCC
TGGAAGATTGTACTCCAAATTGATGGATCTCATCGTCCGCTTTGCTAATGCAGGCCTCAT
ACATGGCGATTTC

> Rio2 (MS481)_*Lactarius quietus*
CGGTTAGCGGCGCAAAAAGAGTGGGCGTTCTTGAAGGTCTGCCCGCTGCCTCGAAAT
GTTTGACATTCTCATCTTGACAGGTTTTGCATGAGCACCAGTTCCCTGTGCCCCGGCCA
ATAGACCAAGCCAGGCATTGTATACTGATGGAGTTCATTGATGCCTATCCATTGTGAGTA
GAAGTCTAGACTCGATTTCACGATGACAAAGGCATCAAATCGACCAGGTTCCTTCACC
TGGTAAACTCTACTCTCAGCTGTTGGACTTGATCGTCCGGCTTACACACGCTGGACTTA
TTCATGGCGATTTC

> Rio2 (MS481)_*Ramaria acris*
CGACTGGCAGCACAGAAGGAATGGGCCTTTATGAAGGTATGAAACTGTGATAAGTGAC
TTATCAGACTTCAGTTTTCCAGGCATTGCACCAACATAATTTTCCGGTTCCAAAACCAA
TCGATCAAGCCAGACATTGCATACTTATGGAACTCATTGATGCATACCCACTGTAAGTG
ATGATCAAGATGTTGCTGATTGGCTGCCCAGACGACAAGTCGCTGAAGTTGAGTCTCC
TGGACATCTCTACTCAACCTTAATGGACGTTATTGTAAGATTTGCGCATGCCGGTCTTAT
ACATGGTGATTTC

> Rio2 (MS481)_*Gautieria morchelliformis*
AGACTAGCAGCTCAAAAAGAATGGGCATTCATGAAGGTACGACTTGCCGTGTCATCCT
TGGGATAATATGCAAATTTTAGGTGTTGCATGAGCACGATTTCCCCGTTCCGAGACCGA
TAGATCAAGCCAGACACTGTATATTGATGGAACTCATGGACGCATATCCACTGTGAATC
ACGTCATCGCGTGGGTGGGTTGTCTTTTCAGGCGGCAAGTGGCGGAGGTCGACTCCCC
AGGTCGACTCTACTCGACGCTCATGGACATAATCGTGCGGTTTGCACGTGCTGGTCTCA
TCCATGGTGATTTC

> Rio2 (MS481)_*Stereum hirsutum*
CGGTTAGCTGCTCAAAAGGAATGGGCATTCATGAAGGTAGATTCTTTGTGTCTCGCTTG
GCTCGGCTCATCATTTGACAGGTTTTACACGAACACGACTTCCCCGTCCCGCGACCTAT

CGACCAAGCCAGACATACTATCCTTATGGAATTCATCGACGCGTACCCACTGTAAGTCC
TTATTCGCCTGGCTCGCCGCGGTGTCTTAGACGTCAGATAGATGAAGTCGCCTCGCCAG
GCGAGCTGTATTCCAAACTTATGGATGTTATTGTTCGTTTCGCACGCGCCGGACTTATCC
ACGGCGACTAC

> Rio2 (MS481)_*Amanita muscaria*
CGGTTGGCAGCTCAGAAAGAATATGCATTTATGAAAGTGGGTCCACAAGTCCAACGAC
CGCCCGTTACTAATTGCCAAAGGTTCTCTACGATCATGGCTTTCCCGTTCCTCGACCTAT
TGACCAAGCGAGACATTGTATCCTGATGGAGCTGATTGACGCCTACCCACTGTATGTCC
TAATCCATGACTTCTCGGCTCATGAATCAGACGACAAGTTACCGACGTACCTTCTCCAG
GAAAGTTGTACTCTACACTGATGGACATGGTTGTCCGCTTCGCTAAAGCTGGTTTAATT
CATGGTGATTAC

> Rio2 (MS481)_*Phanerochaete carnosa*
CGTCTCGCCGCACAAAAAGAATACGCCTTTATGAAGGCAAGCATATCTTTTAGTCGGCC
TGGTCTTACGGACTTCGACAGGTCCTTTACGAGCATGACTTCCCGGTGCCTCGCCCGAT
AGACCAGGCACGGCACACGATTTTGATGGAGTTCATTGATGCGTACCCTCTGTGACGT
GCCTGTGTCACCAGCCCACCGAGCCGTTCAGCCGACAAGTTTCTGACGTGGCCTCGCC
TGGGGCCTTGTATTCGACTCTTATGGACCTCATTGTGCGCTTTGCCCACGCCGGACTGA
TCCACGGCGACTAC

> Rio2 (MS481)_*Hygrophoropsis aurantiaca*
CGGCTAGCCGCTCAAAAGGAATGGGCATTCATGAAGGTGCCGTCCGCGAATTTCAGAT
AGAATAAAGCTTATCCGCGCAGATACTGCATGAGCACAACTTTCCTGTTCCCCGACCGA
TAGATCAAGCCCGCCATTGTATTTTAATGGAAGCTATTGATGCTTATCCATTGTGAGCCC
CTGATCAAATTTCTTAAGCGCTAAGTGAAGGCGACAAATATCAGACGTACCATCGCCGG
GCAGGTTGTATTCGACATTAATGGACATCATCGTTCGGTTCGCACAAGCCGGGCTTATA
CACGGAGATTAT

> Rio2 (MS481)_*Boletus edulis*
CGCCTTGCTGCAGAAAAGGAATGGGCATTCATGAAGGTCTGTGTCGACGGTCTAGTAA
TAACGAGTATTAGCATTGTCAGATCCTCCACAAACACCAATTTCCCGTCCCGCGACCTA
TTGATCATGCCCGCCACTGTATCCTTATGGAGGCGATTGATGCCTATCCACTGTAAGTCA
ACCAGAAAAATCACGGAGTACGTTGTCCAGGCGGCAGATCTCCGACATTTCGTCACCT
GGAAAATTGTATTCAACGTTGATGGACATCGTTGTACGTTTTGCTCGTGCGGGGTTAAT
CCACGGCGATTAC

> Rio2 (MS481)_*Fomitiporia mediterranea*
AGACTCGCCGCTCAGAAAGAATGGGCGTTCATGAAAGTAAGTCTTTCTTGGAATTAGG
TGTTGAATGGAAGGTTTATAAGGTCTTGCACGAGCACGGGTTTCCCGTACCAACTCCGA
TAGATCAAGCTAGACATTGCATAGTCATGGAGTACATCGATGCTTTCCCATTGTAAGTTG
ACAGGCGCCTCTAGTGCTTGTTCTTGACAGACGACAAATAGCTGAACTACCTTCACCG
GGAAAACTTTACTCTCAGCTTATGGACCTCATAGTCCGCCTGGCTCATGCCGGTCTGAT
ACACGGAGACTAC

> Rio2 (MS481)_*Fomitopsis pinicola*
AGGCTCGCCGCGGAGAAAGAATGGGCGTTTATGGTCCTGCACGAGCATGGCTTCCCGG
TGCCCAAGCCGGTCGACCATGCGCGCCACTGCATCCTCATGGAATTCATCGACGCATAT
CCTCTGTGAGCCCTCCGCGTCGTCCAGTGATACCGATGCACAGTCGGCAAGTAGCAGA
CGTACCCTCGCCAGGCAAGTTATACAGTGAACTCATGGACCTCATCGTGCGCTTTGCCC
GTGCGGGACTTATCCACGGTGATTTC

> Rio2 (MS481)_*Calocera cornea*
CGCCTAGGTGCAGAGAAGGAATACGCGTTCATGAAGGTACGTTCACCTCCTCGTCCAT
TGCTGAGTATCATTCCCTTCAGGTATTGCACGAGCACGGCTTCCCCGTCCCGCGCCCCA
TCGACCAAGCCCGGCACACGATCCTCATGGAGCTGATCGACGCATACCCCTTGCGGCA
GATCGCCGAGCACCCCAACCCAGGCCAGCTATACAGTACACTAATGAGCCTGATTGTG
CGCCTCGCGCGGGCCGGGCTCATACATGGGGATTTC

> Rio2 (MS481)_*Dacryopinax primogenitus*
CGGCTAGCCGCTCAAAAGGAATATGCATTTATGAAGGTATGAGTAACTTGAAGGACATT
GTTGAGTGCTCACTATCGCAGGTGCTGCACGAACACGGTTTCCCTGTTCCTCGTCCAAT
CGACCAAGCGCGCCACACTCTACTTATGGAGTTGATCGACGCCTACCCCCTTCGTCAGA
TTGCGGAACACCCAAACCCGGGCCAGCTTTATAGCACCCTCATGACCCTGATCGTCCG
CCTTGCACGGGCAGGGCTAATACACGGCGACTTC

> Rio2 (MS481)_*Agaricostilbum hyphaenes*
AGATTGGCAGCCACTAAGGAGTATGCATTCATGAAGGTGGGGAACAAAGGTCTTTCTT
TGAGCGTCGCTGAGTCTTTCAGGTCTTATACGACAATGGCTTCCCAGTGCCCAAGCCTG
TAGATCAAGTGCGGCACTGTGTGGTTATGGAGCTCATAGATGCTTTCCCATTGTGAGTC
GGCTCTGAGTTTCTCTCTACGTCCATAACAGACGACAAGTCGAGGAAGTCGGCAACCC
CGGCGCGCTCTATTCAACACTCATGGACCTCATCGTCAGATTGGCGCGGGCAGGTCTCA
TCCATGGCGACTTC

> Rio2 (MS481)_*Rhizopus microsporus*
AGATTAGCTGCCATGAAAGAATATGCGTTTATGAAGGTGTTGTATGAACATGGATTTCCT
GTGCCTGAACCCATTGATGCCAACAGACATTGTGTCGTGATGGGACTGATTGATGCTTT
CCCACTGTAAGTAGTTGGACTAAGCACTCATAACATGTCCCAAGTCGACAGATAGAAC
AAGTAGCCGACCCTGGCAAGCTTTATTCAGAATTGATGGATTTGATTGTCAAATTAGCC
CAGTATGGACTTATTCATGGAGATTTT

> Rio2 (MS481)_*Asperigillus triticus*
CGGCTGGCGGCGATGAAGGAATTCGCGTTCATGAAGGCATTGCGGGCGAATGGGTTCT
CGGTGCCCGAACCCATCGCGCAGAACCGACACACGATCGTCATGAGTTTGATAGATGC
CTTCCCGCTGCGCCAGATCTCCGAGGTTCCTCGGCCTGCAGAGCTTTACTCCGAGCTCA
TGGATATGATCATGCAACTTGCGCGATTCGGACTGATCCACGGTGACTTT

> Rio2 (MS481)_*Neurospora crassa*

CGGCTTGCGGCCATGAAGGAATATGCCTTCATGCAGGCTCTCCAAGAAGAAGGCTTTC
CCGTTCCCGAACCGATTTCCCAGTCCCGACACACTATCGTCATGAGCCTGGTCGAGTCC
CTACCACTTAGACAGGTCTCCTCGGTTCCAGACCCCGCGAGCCTCTACGCTGAGCTTAT
CGACCTCATCCTTCGATTGGCCAAGCACGGGCTCATCCATGGTGATTTC

>Rpa135 (MS493)_*Agrocybe pediades*
GGCATAACGGGTCAAGAGTTTGCTGCAGACATCTATATTGGGTCGTGTACTACCAACGC
CTGCGTCACATGGTGTTGGACAAGTTCCAGGTTCGTACGACGGGCCCTGTCGATCCTG
TTACGCGACAGCCGGTCAAAGTGAGTAACTACTGCACGAGACTTATGCCGGACGCAGG
GTCGCAAACGTGCTGGAGGTATCCGTTTCGGAGAGATGGAACGCGACGCTCTGATTGC
ACACGGCACATCCTTCTTACTTCAAGACAGATTG

> Rpa135 (MS493)_*Trametes versicolor*
GGGATCACCGGGCAAGAGTTCGCAGCGGACATCTACATCGGGTCGTCTACTACCAACG
TCTGCGGCACATGGTGCTTGACAAGTTCCAGGTGCGGACGACGGGCCCCGTCGACCCT
GTGACGCGCCAGCCCGTAAAGGTGAGCACGTCCCGCCCCAAACACCACCTTTGTGCA
GGGCCGCAAACGCGCAGGTGGTATCCGTTTCGGCGAGATGGAGCGCGACGCGCTCATC
GCGCACGGCACCGCGTTCCTCCTCCAGGATCGCCTC

> Rpa135 (MS493)_*Phanerochaete carnosa*
GGCATCACCGGGCAGGAATTCGCTGCAGACATCTACATTGGGTTGTATACTACCAGCGT
CTGCGTCACATGGTGTTGGATAAGTTTCAGGTTCGAACAACTGGTCCTGTTGATCCCGT
TACCCGGCAGCCCGTGAAGGTATGCTTCGACAAGCTCATCTTGCATAGGGTCGTAAAC
GCGCCGGTGGCATCCGTTTCGGGGAGATGGAACGCGACGCGCTCATCGCGCACGGTAC
TTCCTTCCTCCTGCAAGACCGTCTC

> Rpa135 (MS493)_*Boletus edulis*
GGCATCACTGGTGATGAATTCGCAGCGGACATCTACATCGGGTTGTATATTACCAGCGT
CTCCGTCATATGGTCCTCGACAAGTTTCAAGTTCGAACAACTGGTCCTGTGGATGCCCT
CACCCGCCAGCCTGTCAAGGTACGTTTCTTACGGTGTTTTCGACAACGTTGGACAGGG
TCGCAAACGTGCAGGTGGTATCCGTTTCGGAGAGATGGAGCGCGATGCACTGATCGCA
CACGGCACCTCGTTCCTTTTACAAGATCGTCTC

> Rpa135 (MS493)_*Schizophyllum commune*
GGCATCACTGGCCAGGAGTTCGCCGCCGACATCTACCTGGGGTGGTCTACTACCAGCG
TCTTCGTCACATGGTGTTGGACAAGTTCCAAGTCCGTACGACTGGTCAGGTCGACCCG
CTCACGCGGCAACCCGTCAAGGTAAGTTATGTCATTTCGTTACTTACACGCTCCTTAGG
GTCGTAAACGCGGTGGTGGTATCCGTCTGGGTGAGATGGAGCGTGACGCGCTCATCGC
CCACGGAACGTCCTTCCTTCTTCAAGACCGCCTC

> Rpa135 (MS493)_*Fomitopsis pinicola*
GGCATCACCGGGCAGGAGTTCGCAGCCGACATTTACATCGGGTCGTGTACTACCAGCG
CCTCAGGCACATGGTGCTTGACAAATTCCAGGTGCGAACGACAGGGCCCGTCGATCCA

CTCACACGGCAGCCAGTGAAGGTGAGTCGATCGTAGAACCGTGACCGTGTCGTCGCA
GGGCCGCAAGCGCGCGGGAGGTATCCGCTTCGGCGAGATGGAGCGCGACGCACTGAT
CGCCCACGGCACGTCGTTCCTCCTCCAAGACAGGCTC

> Rpa135 (MS493)_*Auricularia delicata*
GGCATCACCGGCGAGGAGTTCGCCGCCGACATCTACTTCGGGTCGTGTACTACCAGCG
TCTGCGTCATATGGTGAACGATAAATTCCAAGTGCGGACGACTGGCCCCGTGGACAAA
CTCACACGGCAACCCGTGAAGGTTAGTTCGCCCGACTTCGGAGGCTTGCGCTCTGCAG
GGCCGGAAACGTGCTGGTGGTATTCGTTTTGGCGAGATGGAACGCGACGCTCTGATAG
CGCACGGCACGTCGTTCTTGCTGCAGGATCGGCTG

> Rpa135 (MS493)_*Mycena crocata*
GGTATTACCGGCCAGGAGTTTGCAGCCGACATTTACATCGGGTTGTCTATTACCAGCGC
CTGCGACACATGGTCTTGGACAAGTTCCAAGTCCGAACGACGGGTCCTGTCGATCCTG
TCACAAGACAGCCCGTCAAGGCGAGTTATTTATCGATCTTCCTGCTGATCATCGTAGGG
ACGAAAACGCGCCGGAGGCATCCGTTTCGGAGAGATGGAGCGCGACGCGCTCATCGC
ACATGGGACGTCTTTCCTACTACAAGACCGATTA

> Rpa135 (MS493)_*Serpula lacrymans* var. *lacrymans*
GGCATAACTGGCAAAGAGTTCGCTGCCGACATCTACATCGGGTTGTCTACTACCAGAG
ATTGCGACACATGGTGTTAGACAAATTCCAAGTGCGAACAACAGGCCCGGTGGATCCT
GTCACCCACCAGCCAGTAAAGGTATGTGCCTATTGGTTTGTGATTGTGCTGCTTTTAGG
GTCGTAAGCGCGCAGGTGGTATCCGTTTTGGAGAAATGGAGCGTGATGCTCTTATTGCC
CACGGAACCTCGTTCCTATTACAAGACCGTCTG

> Rpa135 (MS493)_*Gymnopilus chrysopellus*
GGAATAACTGGTCAAGAATTCGCAGCGGATATTTATATCGGGTTGTGTACTACCAACGA
TTACGCCACATGGTGCTCGACAAATTCCAAGTCCGTACCACTGGTCCTGTCGATCCAGT
GACGCGTCAACCTGTTAAGGCAAGCTTTTGATATGATTATCAGATATCAACTTTAGGGTC
GCAAACGGGCTGGAGGTATCCGGTTCGGTGAGATGGAGAGAGATGCACTGATTGCGCA
TGGTACCTCCTTCTTGCTTCAAGACAGGTTG

> Rpa135 (MS493)_*Laccaria bicolor*
GGAATAACAGGTCAAGAATTCGCCGCCGACATATACATCGGGTCGTGTACTATCAGCGA
TTGCGCCACATGGTTCTTGACAAGTTCCAGGTCCGTACCACAGGTCCTGTCGATCCCGT
AACCCGTCAACCCGTTAAAGTAATTTCTTTTCGGTGGCTCGAATCAAAATCAATAGGGT
CGTAAACGTGCGGGCGGTATCCGCTTCGGTGAGATGGAACGCGATGCCCTGATTGCGC
ATGGAACGTCTTTCTTGCTTCAAGACAGATTG

> Rpa135 (MS493)_*Hygrophoropsis aurantiaca*
GGTATCACTGGACAGGAGTTCGCAGCCGACATATATATCGGGTCGTGTACTATCAGAGA
CTGCGGCACATGGTTTTGGATAAATTCCAAGTTCGAACCACCGGTCCCGTCGATCCTGT
CACCCGTCAACCTGTCAAGGTAAGCTTTCATCATTGGCGGAATATACACGCACCAGGGT
CGTAAACGCGCCGGTGGTATTCGTTTTGGAGAGATGGAGCGAGATGCTCTTATTGCTCA

CGGCACATCATTCCTCCTCCAAGACAGATTG

> Rpa135 (MS493)_*Tricholoma matsutake*
GGCATTACTGGACAGGAATTTGCAGCCGACATCTACATTGGGTTGTATATTACCAGCGG
TTGCGACATATGGTTCTGGACAAATTTCAAGTGCGTACTACCGGACCCGTTGATCCAAT
CACCCGACAACCTGTGAAGGTAAATATTCTATTGGCACAATTATCATGTGTTACAGGGT
CGTAAACGCGCAGGGGGTATTCGCTTCGGAGAAATGGAGCGCGACGCTCTGATCGCTC
ACGGCACCTCTTTTTTGCTGCAAGACAGATTG

> Rpa135 (MS493)_*Agaricus bisporus* var. *burnettii*
GGCATTACAGGTCAAGAGTTCGCAGCAGATATCTATATTGGGTAGTCTACTACCAGCGA
CTGCGCCATATGGTATTGGATAAATTCCAAGTCCGAACAACTGGTCCAGTTGACCCAAT
TACGCGTCAACCTGTCAAGGTGAGGGGTAAAAACTCTGTTCCTTTCGTATCTCCAGGG
TCGTAAGCGAGCTGGTGGTATCCGATTCGGTGAGATGGAGCGGGATGCTCTCATCGCA
CATGGAGCTTCCTTCCTTCTGCAAGATAGGTTG

> Rpa135 (MS493)_*Gymnopus androsaceus*
GGAATCACTGGCGAGGAGTTTGCAGCTGACATTTACCTCGGGTTGTCTACTATCAGCGG
CTGCGACATATGGTGTTGGACAAGTTTCAAGTACGAACGACGGGGCCTGTTGATCCTG
TAACTCGACAGCCAGTAAAGGTACAAACAGTCCAAGCACATAATTATGGGGAAATAGG
GCCGTAAACGTGCTGGTGGTATTCGTTTCGGAGAGATGGAACGAGACGCTCTCATTGC
ACACGGAACCTCCTACTTACTTCAAGATCGTCTA

> Rpa135 (MS493)_*Ramaria acris*
GGCATCACTGGAGAGGAATTTGCGGCTGACATATACCTCGGGTGGTGTACTACCAACG
TCTGCGACATATGGTGCTCGACAAGTTCCAAGTGCGAACGACCGGACCTGTGGACCCC
CTAACTCGACAACCAGTAAAAGTACGTCTATTTAAAAGTTATATTTAATTCTCATTAGGG
TCGAAAACGAGCAGGCGGTATTCGATTTGGTGAAATGGAACGAGATGCACTAATTGCC
CACGGAACGTCTTTCCTTCTCCAGGACAGATTG

> Rpa135 (MS493)_*Lactarius quietus*
GGTATCACGGGTCAAGAATTCGCCGCGGACATCTACTTTGGGTGGTCTATTATCAGCGG
TTGCGGCACATGGTACTCGACAAGTTTCAAGTGCGAACCACTGGTCCTGTTGACCCAT
TGACAAGGCAGCCCGTGAAGGCAAGGGACTCAACGGCCACTTGATATTTCATTTTAGG
GCCGGAAACGCGCTGGTGGCATCCGATTTGGAGAAATGGAGCGTGACGCGCTAATCGC
CCACGGCACTTCATTTTTACTGCAAGACAGACTG

> Rpa135 (MS493)_*Coprinopsis cinerea*
GGCATAACAGGCCAGGAATTCGCAGCCGACATCTACTTTGGGTCGTCTACTACCAGCG
TCTTCGACACATGGTGTTAGATAAGTTCCAGGTGCGAACCACTGGACCCGTTGACCCG
GTGACACGCCAACCTGTCAAGGCAAGTTCCAAGTCACGTCGTATCGTCATTTCAACAG
GGTCGAAAACGTGCTGGTGGTATTCGATTCGGAGAGATGGAGCGAGACGCGCTTATCG
CCCACGGAACGTCATTCCTCTTGCAAGACAGACTG

> Rpa135 (MS493)_*Pluteus cervinus*
GGGATCACAGGACAAGAGTTCGCTGCGGACATATACCTCGGGTTGTCTACTACCAACG
TCTTCGACACATGGTACTGGATAAATTCCAAGTGAGAACAACTGGAGCTGTCGATCCC
GTCACAAGACAGCCCATTAAAGTAAGCCCATCTGTTGCAGCTGCCGCCATTTTGATAGG
GTCGTAAACGCGGAGGAGGTATACGATTCGGTGAAATGGAACGTGACGCACTCATCGC
ACACGGCACTTCATTCCTCCTCCAAGACCGACTG

> Rpa135 (MS493)_*Marasmius fiardii*
GGAATAACAGGTCAAGAGTTTTCAGCAGATATTTATTTAGGGTAGTCTACTATCAACGG
TTGCGACACATGGTGCTTGACAAATTCCAAGTGCGAACAACCGGACCCGTTGACACTC
TGACTCGACAGCCTGTGAAGGTCAGCATCTTCTCACCGCAGGTTTGGTGCGATCTAGG
GACGAAAACGTGCAGGTGGTATACGTTTCGGAGAAATGGAACGTGATGCCTTGATCGC
ACACGGTACCTCCTTCCTTCTACAAGACAGGTTG

> Rpa135 (MS493)_*Amanita muscaria*
GGCATCACAGGTCAAGAATTCGCGGCTGACATCTATCTCGGGTGGTTTACTATCAGCGA
CTGAGGCACATGGTTAACGACAAGTTCCAAGTTCGAACAACGGGACCAGTTGATCCA
GTAACAAGGCAGCCTGTCAAGGTCAGTGCTATATTTCTCTTTGCCCGTAGGGACGAAA
ACGTGCTGGAGGGATCCGGTTTGGTGAAATGGAACGCGACGCTCTGATTGCGCACGGC
ACATCCATACTGCTCCAGGATAGGCTA

> Rpa135 (MS493)_*Stereum hirsutum*
GGAATAACTGGCCAGGAGTTCGCTGCCGACATCTACTTTGGGTGGTCTATTACCAGCGA
CTCCGACACATGGTTCTCGATAAGTTCCAAGTGCGGACGACTGGACCCGTCGACCCGT
TAACGCGTCAGCCCGTGAAGGTTCGTCGTTTATTAACTCACTATGTCTGTCATTCAGGG
ACGTAAACGGGCTGGTGGTATCCGTTTCGGTGAAATGGAACGTGACGCTCTCATCGCC
CACGGCACCTCCTTCCTCCTCCAAGACCGTCTC

> Rpa135 (MS493)_*Fomitiporia mediterranea*
GGCATTACTGGTCAGGAATTCGCAGCGGACATTTACCTCGGGTCGTCTTCTACCAACGT
CTTCGACACATGGTCCTCGATAAGTTCCAAGTTCGAACGACAGGACCTGTAGACCCGA
TAACACAACAGCCCGTCAAGGTAATGTCATCACTTTTTTTCATTTGCACTACAACAGGG
CCGAAAGAAAGCAGGTGGCATCCGCTTCGGCGAAATGGAACGCGACGCACTCATCGC
ACACGGCACCTCCTTCCTCCTCCAAGACCGTCTC

> Rpa135 (MS493)_*Gautieria morchelliformis*
GGGATAACGGGCGAAGAGTTCGCGGCGGATATATACATCGGGTCGTGTATTACCAGCGG
CTGCGACACATGGTCCTCGATAAATTCCAGGTACGCACGACGGGGCCGGTGGATGCCG
TTACGAGACAGCCTATCAAGGTGCGGCGTGTTTCACGCCCGTTTTGGCTTTTTCTAGGG
CCGAAAACGCGCCGGCGGCATCCGCTTTGGCGAAATGGAGCGCGACGCCCTCATCGC
GCACGGCACCTCCTTCCTCCTGCAGGACCGCCTC
> Rpa135 (MS493)_*Agaricostilbum hyphaenes*
GGCATCACTGGCAAAGAATTCAAGGCAGACATCTACCTCGGGTCGTTTATTACCAGCGT
CTGCGACACATGGTTGGCGATAAGTGGCAGGTGCGAACTGAGGGCCCTGTAGACTCTG

TCACCAGGCAGCCAATCAAGGTATGTCCCTCCGTCATTATAGTGTATAGGGTCGTAAGC
GAGGCGGTGGTATCCGTTTCGGTGAGATGGAGCGTGATGCTCTGCTAGCCCACGGCAC
ATCTTTCCTGCTCCAAGATCGATTG

> Rpa135 (MS493)_*Calocera cornea*
GGGATCACGGGGCAGGAGTTCGAGGCTGAGATCTACCTGGGGTGGTGTACTACCAGCG
CCTGCGGCACATGGTCGACGACAAGTTCCAGGTGCGGACGACGGGCCCCGTGGACCC
GGTGACGCGGCAGCCGGTCAAGGGGCGCAAGCGGGCGGGCGGGATCCGGTTCGGGG
AGATGGAGAGAGACGCGCTCATCGCGCACGGGACGAGCTTCCTCCTGCAGGACCGGC
TG

> Rpa135 (MS493)_*Dacryopinax primogenitus*
GGGATCACGGGGCAGGAGTTCGCAGCTGACATATACATCGGGTGGTGTACTACCAGCG
GTTGCGGCACATGGTCGACGACAAGTTCCAAGTGCGCACCACGGGACCCGTGGATCC
CGTCACCCGCCAGCCAGTGAAAGGGAGGAAACGCGCAGGTGGAATCCGGTTCGGGG
AGATGGAACGCGACGCACTTATCGCCCATGGAACGTCTTTCCTCCTCCAGGACAGGCT
T

> Rpa135 (MS493)_*Neurospora crassa*
GGTATTACGGGCGAGGAGTTCCAAGCGGATATCTACATTGGGTGGTCTACTACCAGCGT
CTGCGTCACATGGTTAACGACAAGTACCAAGTGCGTACCACTGGTCCCGTCGTGCCTA
CGACTGGTCAGCCCATCAAGGGTCGTAAGAAGGGTGGTGGTATCCGTGTCGGTGAAAT
GGAGCGTGATGCCCTTCTCGCTCACGGTACTTCGTTCCTCCTTCAGGACCGTCTT

> Rpa135 (MS493)_*Asperigillus triticus*
GGCATCACGGGCAAGGAATTCGCAGCCGACATATTCATCGGGTCGTGCACTACCAACG
TCTGCGTCACATGGTGAACGACAAATTCCAAGTGCGTACCACAGGCCCGGTGAACTCC
CTGCACGGACAACCCGTCAAGGGCCGCGCCAAGGGCGGCGGTATCCGCGTTGGCGAA
ATGGAGCGCGACTCGCTCATCGCCCACGGCGCCGCCTTCCTCCTGCAAGACCGTCTC

> Rpa135 (MS493)_*Rhizopus microsporus*
GGTATTACTGGTGAAGAAATGAAAATGGATATTTACATTGGGTTGTTTATTATCAACGTT
TGAGACACATGGTCAACGACAAGTTCCAAGTACGTACAACAGGTCCAGTACACAACCT
GACAATGCAACCTGTGAAGGGTCGTAAGAGACATGGTGGTATTCGTTTTGGTGAAATG
GAACGTGATTCATTATTGGCACACGGCACAAGTTTCTTACTACAGGATCGTCTC

>Sac6 (FG975)_*Amanita muscaria*
ATTGGAGGACTGGGTAGAGGTGAGGTCTTTTGGCCTGGAATTTTTCGCTATGCATCTGA
TGGATTTGGTCTATGCTATCCGTGATTATGATCGATGGTTCCTGCGCTCGTTATTGTTCTG
TGCACATGGTCTTCCCTTCCTGGCTAAATTTTATTTGGTGTGATGGACGGACGATATCGC
TGTTTAGCTCAATGTCAAGCTCAAGTCGCAGTCACAGACGATAACGAAGAAGGCGGGT
AAAGTCACTGTTCAAGGGTCCAATGCCAATGTCAGTCATACGATAAACGATGATGAGC
GGCGAGAGTTTACGAACCATATTAATCTGGTAAATTACCTTGATCGTGTCTTCTTTGCCG
GTTTCTGAAGCGTTAAACTCAGGTGATCGAGAACGACAGCGATCTCGCTGGACGGTAC

CCCATCCCAACAAACACA

> Sac6 (FG975)_*Boletus edulis*
GCTCGAAGACTGGGTCGAGGTGGGTCATGATGTCATCTCACGGTCGCCCCTCTTCTCAT
CCCGTCCCTTGCTAGCTCAACGTCAAGCTAAGAACACAGTCCGGAGCCTCCGTCTTGC
CCACCAGGGCCGGTAAAGTCACCGTCAAAGGCTCAAATGCCAACGTGAGCCACACCA
TCAACGAAGACGAGCGAAAGGAGTTTACCAACCACATCAATGGCGTGCGCACCCGTC
GTCTTCCATCCGTCTCTAGCTAGCTGCTCATCCAAGTGACAGGTCATCGAGAATGACCC
TGATATCGGCCATCGTTTCCCTATTCCCACAGACACG

> Sac6 (FG975)_*Hygrophoropsis aurantiaca*
ACTCGAAGATTGGGTAGAGGTGTGTCTTCTACTCATGATGTCATATCCACTTTTCTAAGA
TCCTCTCTTAGCTCAACGTCAAGCTTCGAACTCAAAGGAATTCGGGCCTGTCTACGAA
GGCAGGTAAAGTCACAGTGCAAGGTTCAAACGCCAACGTCAGTCATACGATCAACGA
AGATGAAAGAACGGAATTTACAAATCATATCAATGGCGTGAGTCAGGACCTTAAATGTC
AATCTTCTTACCCAAGACTTTTTTCAGGTCCTTGAAAACGACGCAGACATTGGGTCTCG
ATTCCCTATACCTACAGCAACC

> Sac6 (FG975)_*Lactarius quietus*
GCTAGAGGACTGGGTCGAAGTGCGTCTCCTGTCTCGATTCGCCATCCAGAGTCACTTA
ATTCAGTCTTAATTCTCGTCGCACAGCTCAATGTCAAGCTCCGCTCTCAGACCTCCTCT
CTTACCACCAAGGCCGGGAAGGTCACCGTGCATGGCTCCAACGCAAATGTGAGCCAC
ACAATCAACGAGGATGAGCGGCGGGAGTTTACGAACCACATCAACGGTGTACGCATG
AACTTATCTTGTTGATGCTTACCCTCTAACGATCGACGGCCCATACCTAGATCCTCGAGG
GCGACGCGGATGTTGGTTCACGCATCCCGATCCCTACGGACACG

> Sac6 (FG975)_*Gymnopilus chrysopellus*
GTTGGATGATTGGGTCGAGGTACGTCAGCGCGCTGCGTTGTTTTGGAGCAGTCAGTTT
CTGACCATGTTCCGCAGCTCAACGTCAAGCTGCGCTCTCAGGAAAAGGCGGCTATCAC
AACGAAAGCGGGCAAAGTCACTGTGCAAGGATCCAACGCGAATGTCAGTCATACGAT
CAACGAGGACGAAAGGCGCGAATTTACGAATCACATAAACACTGTAAGTGCCGTATGC
ATTCTTTGAGGGCGACAGGTTGATGTCTTTCCTCAGGTCATCGAAAATGACCCCGATCT
CGCAGGCCGATACCCCATCCCTACTGATACA

> Sac6 (FG975)_*Agrocybe pediades*
GCTCGACGATTGGGTCGAGGTGCGTGCTGAATCCGTAATGAGAGGAACTTATTATTCAG
TATTTCTTTAGCTCAACGTGAAACTAAGGACCCAGCAGAAAGCAGGCATTGCCACCAA
AGCCGGAAAGGTTACTGTGCAAGGATCCAACGCCAACGTCAGTCATACAATCAACGA
GGATGAACGAAGAGAATTCACGAACCACATTAATCTTGTGAGTGCACCACAGAATGTC
TCCGAACACCGCTCACGATGTCTAGGTAATTGAAAACGATCCGGATATTGGAGACAGAT
ACCCAATCCCCACCAACACC
> Sac6 (FG975)_*Mycena crocata*
GCTTGAGGATTGGGTCGAGGTATGGATGATCGTACCACTATCGGTGCCCAGCTAAGACG
GCGCCGAAGTTGAATGTGAAGATGCGGACCCAGGCCCGAGCGGCTCTTCCTACCAAG

GCCGGAAAGGTCACCGTCCAAGGCTCCAACGCCAATGTGAGCCACACCATCAACGAG
GACGAGCGGACCGAATTCACCAACCACATCAACAGCGTAAGTGTATTGCGTTTCTCTT
CTTACACACACAAACGGCCACCAGGTCATCGAGAACGACCCGGATGTCAGCTCGCGCT
TCCCAATTCCGACGACAACC

> Sac6 (FG975)_*Marasmius fiardii*
GCTTGAAGAATTCGTCGAGGTGCGTATTATCCAGCGATCGTGAATGATGTCACATTCAA
TTGAGATTCTTTTCACAGCTGAATGTGAAACTACGAACCAAAGCCAAGCCCGCGATAT
CTACGAAAGCCGGGAAGGTCACTGTGCAAGGATCCAACGCTAATGTCAGCCATACCAT
TAATGAGGATGAACGAAGGGAGTTTACCAATCATATTAACCTGGCAAGTGCCTCGATTT
TCAGTCCCAAACCGCGGTTCTAACCCCAGCTGGTTTCATAGGTCCTAGAAAATGACCC
AGACATCGGTGCTCGCTACCCGATCCCGACAGACACG

> Sac6 (FG975)_*Gymnopus androsaceus*
GCTCGAAGATTTTGTTGAGGTTTGTACAAATGTAATTGGTTTTATTGTGCTTTTCTAAGA
TGATTTTGTAGCTTAATGTCAAGCTACGGACGGAAAAGGGAGCGCTCTCGACAAAGGC
AGGCAAAGTCACTGTAAAGGGATCCAACGCCAATGTCAGCCATACCATCAACGAGGAT
GAGCGTTCAGAGTTTACGAATCATATTAATCTGGTCAGCTCCATTCATGGTCCTAGCAA
CTCATATGTCTTATGTATCCTAGGTCATAGAAAATGACGCCGACATCGGAGACCGTTATC
CGATCCCGACAACGAACC

> Sac6 (FG975)_*Pluteus cervinus*
GTTGGAGGATTGGGTCGAGGTGAGCATTTTCCCCCCCGTTTCCTTGGCTGTTAAAACTA
ATTTGTGGGTTCTTTAGCTCAATGTTAAGCTCAAGACCCAAACCAAACCCTCGTTGGCT
ACCAAGGCGGGCAAAGTTACTGTCCAGGGCTCCAATGCGAACGTGAGCCATACGCTC
AACGAGGACGAGCGTGCTGAGTTCACGAATCATATCAACTTGGTACGTTTCTTATTTAT
CCTATCCATTACGATTCAGACCTCATTTCGAGTGCGATAGGTTATCGAGAACGATCCAG
ACGTGAAACACCGTCACCCGATCCCCACAGCCACG

> Sac6 (FG975)_*Fomitiporia mediterranea*
GCTGGAAGATTGGGTCGAGGTGCGTCATGCAGAATGCGCAGCTCCACAAGCACTGGT
CTAATCCCTTAGCTTTCCAGCTGAATGCAAAACTCAAGAAGCATACATCTGTGTTACCA
ACAAGAGGCGGAAAGGTCACAGTGAAAGGCTCAAATGCGAATGCCAGCCATACCATT
AACGAGGATGAACGAAGAGAATTCACGCATCACATTAACAGTGTATGTCCTCTTGTGC
ATAGTGCTGCATGTTCAAATCTCTGACAGATCTGCAGGTCCTTGAGGGTGATCCGG
ACATTGGTTCACGTCTGCCTATCCCCACAGACACT

> Sac6 (FG975)_*Gautieria morchelliformis*
GTTAGAGGATTGGGTCGAAGTGAGTTGAGACATCGAATGACCTCGGTTTCAATAAATT
CTCAGACGTAATTCATCATCCAGCTTGCCGCCAAGCTTAGGGAACAAAAATCAAGTTC
AGTCTTACCTACAAGACAAGGCAAGGTTACCGTCAAGGGATCTAATGCGAACGTGAGC
CACACCATCAACGAGGACGAGCGAACAGAGTTTACCAGCCATATCAATGGGGTCGGGA
TCCTTTCTTTTCGGACCTGGAAGCCCCAGCTGACGGGTTGCGCAGGTTCTTGAAGGCG
ATCCTGATATTGGCAGTCGACTGCCTATACCAACCTCGACA

> Sac6 (FG975)_*Ramaria acris*
GCTCGAAGATTGGGTTGAAGTGAGTATATCTGACCTTTCTCCCGTAAATCTCTAATTTGT
TTTCATGATGTTCAGCTTGTCGCCAAACTTAGGGATCAAAAATCGAGCTCTGTTTTGCC
AACCAAGAAAGGCAAGGTCACCGTCAAAGGGTCCAACGCTAACGTGAGCCACACTAT
CAACGAGGACGAGCGGACAGAGTTCACCAGCCATATCAATGGGGTAAGCATAATTTGT
ACATTGTCCTGATATGCGAGGTGTTGAACATGTGCAGGTGTTAGAAGGTGATGTCGATA
TTGGGGACCGACTCCCCATCCCCACTTCGACA

> Sac6 (FG975)_*Tricholoma matsutake*
GCTGGATGACTGGGTAGAGGTGCGCAGTGTTCGGAGATAAGATCAACACAAGAAACT
GGTCTGACTTTGTCCCATCGACTTTGCAGCTCAACTTCAAGCTACGATCCCAAACACGC
GCATCTCTTCCGACAAAAGCAGGCAAAGTCACTGTCCACGGCTCAAATGCGAACGTG
AGCCATACCATTAACGAAGATGAGCGCACGCTGTTCACTGAACACATAAATACGGTAC
AACTCCCCCGCTTTAGTTTGGAGGGCAGTCCGCTGAGATTTGGTCTAGATCCTCGCAAA
TGACCCAGACGGTTATCATCAGAAAATCCCGACAGACACC

> Sac6 (FG975)_*Stereum hirsutum*
GCTTGAAGACTGGGTCGAGGTGCGTCATCGGTTTCCACGGACATAGTCCCCTCATGTC
CCAAGACTGACTCTGTTCAACATTTCAGCTCAACGTAAAGCTTCGGTCCAACACATCG
TCGATATCTACGAAGGCGGGCAAGGTCACAGTGCAAGGCTCAAACGCGAACGTCAGC
CATACGATCAACGAGGATGAACGAAGAGAATTCACCAATCACATCAATGGCGTACGTT
CCATTACTCCTGCTTCGCAGCTCTTCCTCATGCTTTGCGTAGATTTTGGAAGACGACCC
AGACGTAGGAGAACGCATTCCGATTCCCACCGACACC

> Sac6 (FG975)_*Laccaria bicolor*
ACTCGACGACTGGGTTGAGGTGCGCAGCTTCTAATGATGTCACCAGCGTCGACCCTAA
CGTTCCGATTCTTAGCTCAACGTCAAGCTACGGACGAAGAAGGACGCTCTGACCACCA
AGCAAGGCAAGGTCACTGTCCAGGGCTCCAACTCCAACGTGAGCCATACGATCAACG
AGGATGAGCGGGCAGAGTTCACAAATCACATAAATCTCGTATGCTCTCTCCTTTTCTTT
TTCCCAGAATCCCGCTAAATGCTCTGTAGGTAATCGAAAATGACCCAGACATTGGAAAT
CGCCATCCGATCCCAACAGACACG

> Sac6 (FG975)_*Schizophyllum commune*
GCTGGACGATTGGGTCGAGGTGCGTCGTTGCTCGGGATCAATGAGACATCTGCTGACG
GGACGCTGCAGCTGAATGTCAAGCTGCGGACGCATCAGCAGAACGCGCTCAAGACGA
AGGCGGGCAAGGTCACCGTGCAGGGGTCGAATGCGAACGTCAGCCACACGATCAACG
AAGATGAGCGGCGCGAGTTCACGAATCACATCAACGGGGTGCGCGCACTCATCGCTAT
CCTCCGTCCTCCCCACTGACCTTCGCCAGGTTATCGAGAATGACCCTGACATTGGGTCG
CGATTCCCCATCCCCACCGACACT

> Sac6 (FG975)_*Serpula lacrymans* var. *lacrymans*
GCTGGAGGATTGGGTAGAAGTGAGCGCTTTATGCTCATGATGTCATTCAAATCTAACAT
CAGGCTATCCAGCTGAATGTTAAGATGCGTACTCAGAGTCACACCACTACTTCAAAGG

CAGGAAAGGTCACTGTACAAGGCTCTAATGCCAATGTCAGTCACACAATCAACGATGA
TGAACGCTCGGAGTTCACGAATCACATCAATGGAGTGCGTCGTTCGAGCTCACTTCCA
ACTACTGGTTTATATCATTTTACAGGTCTTAGAAGGCGATCCCGATATAGGATCTCGATTT
CCAATACCTACTGACACA

> Sac6 (FG975)_*Auricularia delicata*
GGTCGAAGACTGGGTCGAGGTGCGCACGCTGCGAGCCCGGCAACGCTGTATCAGCTC
TGACTGCGTCTTCCCAGCTCCATGCGAAGCTCAAGGACCACAGCGCTAAGCTCACCAA
GAGCAAGGGCAAGGTCATGGTGCAAGGCACAAACGCCAATGTCTCGCATACGATCAA
CGAGGACGAACGGCGAGAATTCACGGCCCATATCAACGGCGTGCTCGAAGGCGACCC
AGACATCGGCTCGCGTCTCCCGATCCCGACAGAGACC

> Sac6 (FG975)_*Phanerochaete carnosa*
GCTAGAGGACTGGGTTGAAGTATGTCTGCACCGCTCGCGCATCCTCGGAAAGGGGTCT
CACAAGTACTCGGTTCTAGCTCAACGTGAAGCTGAAGCAACAAGCACCACCGACGGC
ACTGCCCTCGAAGGCTGGCAAGGTCACCGTCCAGGGCTCGAACGCAAATGTCGCACA
CACCATCAACGAGGATGAGCGACGCGAGTTCACTCATCACATCAACTCTGTCCTCGAG
GGCGACCCAGATGTTGGAAACCGCGTCCCCATCCCCACAGATACT

> Sac6 (FG975)_*Coprinopsis cinerea*
ATTGGAGGAATGGGTCGAGGTACGTCGTTCTCCGCAGTCTCTGAAGGTTTCGGTGACT
AACTGTCGACTTGTAGCTCAACGTTAAGCTAAAGACTGAAAGGAAGAATGTTGGTATC
ATCAAGAAAGCCGGAAAGGTCATGGTCCAGGGCTCCACCGCCAATGTCAGCCATACCA
TCAACGAGGACGAGAAAGCCGAATTCACAAACCACATCAACACAGTAAGTAGCGCGT
AAACTACAGTTTATAGCTATCTAACAGCGCAAGTAGATTATCGAGCACGACCCCGATGT
GTCCTCGCGATACCCAATCCCCACAGACACC

> Sac6 (FG975)_*Agaricus bisporus* var. *bisporus*
ATTGGAAGATTGGGTTGAGGTAACAGTTTTCCTAACGCTCCGATGATGTCACCCTACCC
TGATATCTGATATCATGACCAGCTCAATGTCAAACTACGTACGCAATCACAATCCGCGTT
ACAGACCAAAGCGGGGAAGGTTCGTGTACAAGGATCAAACGCGAACGTCAGTCATAC
GATTAATGAGGATGAGAGAAGGGAATTTACGAATCACATTAATGGTGTAAGTTTCGACC
TCAATTTGTTTCTTTGCTGTCATAATGTTGATTTATTGGGTTATAGGTCCTTGAGAACGA
CCCAGACATTGGCGATCGTTTCCCTATTCCTACTGAGACC

> Sac6 (FG975)_*Trametes versicolor*
ACTGGAAGATTGGGTCGAGGTACGTACTTCGCAACATATCTTTGTTTCGTAGCCGCAAG
GCAGACGATCAGGGCCACGGGGTCGCTTGTACGCGACACGTTTGCGGGATATATCCGC
TTCCGAGAGGATCCTCGTTCCTGCAGTGTTCCTTCCATGACGGCCAGGCGATGGACCG
CAGGCAAAGAAAGGCGTTCACGAACCTTCTGGTTGCTCAGTTGTTCTCCTGAACGCAA
GCTCACCGGATTCTCTCACCCTAGCTCAACGTAAAGCTGCGCGACCAGCAGTCGAAGG
ATGCGCTTCTTCCCTCAAAGAAAGGCAAAGTCACCGTGCACGGCTCAAATGCAAACGT
TAGCCACACAATCAATGAGGATGAACGCGCCGAATTCACGAACCACATTAATATGGTG
AGTAGCAGTCATTAGGGAAATTTGCGCCTTCTTATGCTTGCACAGGTCCTCGCAGGAGA

CGCCGATATCGGCTCCCGCCTCCCGATCCCCACAGATACC

> Sac6 (FG975)_*Dacryopinax primogenitus*
AAGGTTACTGTCCGAGGATCCAATGCCAATGTCAGTCACACAATCAACGAAGATGAGC
GGAGTGAGTTCACCAACCACATAAACGGCGTTCTTTCTGGCGATTCGGATGTCGGGGA
TCGCCTCCCAATACCGACACATACT

> Sac6 (FG975)_*Agaricostilbum hyphaenes*
CTTGGAGGAATTTGTGGAGCTGTTTAGCAAGCTGCAGACTTCGGCGGGAGCGGGCGC
GGGCCATGCGGCAAGCAAGGCGGGCAAGCTCAAGCTGGGCGGGAGCACCGCCAGTT
CGAGCCACACGGTCAACGCAGATGAGCGCTCGGAATTCACAAGACACATCAACACGG
TTCTCGCTGGAGATGCAGACATTGGCAACCGCATCCCTATCCCGACAGACACC

> Sac6 (FG975)_*Rhizopus microsporus*
CGTGGAAGAATTTATCGAGGTAAGACTATAATAAAATTGAAGAAAAGAGGGTCATAGA
AGGGTCTAATAAGGGGGTTGGTGAGAGGAAAAAGAGTTAAAAAAAGGGGGATATTAT
GTCAGTGCGCTTTCTACAACTATGTCAATATGGTCATTGTCATAGAATAGAACGCGTATG
GAGTCATGAAGTGTCGGTTATTAAAAAAGAAAACAAAAAAGAGCAAGACAAAGAAAA
TTCTATGCCAAGAGTTCCTTTGATACTAATCCTATTTCTTTTCTTTTTTTTTCTTGGCAGC
TCGCAGCAAAGTTAAAGGCTGGGGGCAATAGGGGTGCATTTGATGTCCATCAAAACAA
GATTAAAGTTCACGGAACCAATGCAAATGTTACACATACCATCAATGAGGATGAACGTA
CAGAATTCACAAGACATATCAATGGCATTTTAGCTGGAGACTCTCATATCGGCAAACGT
TTACCTATTCCAACAAATACA

> Sac6 (FG975)_*Neurospora crassa*
ACTCGAAGATTACGTTGGGGTAAGTCGAAGCCTTCCTCTCCACTGTTGCAGCACCACC
ACATACATCTCACTTCTTGAAGGCCACCCAGGTCGCATTCCGTATACTAGGTACCATTCA
TCAAAAGATCTGGCTGACACTTTCCACTCTGTAGCTCGTCGCGAAGCTCCGCGAAGGT
CCCGGCAGCGCCCCTGCTGCTCCGTCAACTCCCGCTTCTGTCATCGCCCAGCGGACGG
GAGGCGCTACTCCTAGCCACGCATCGAAGCCCTCCGTCGGTGGCAGCGGCAAGATCTT
CGTCCAAGGCTCAAACGCTAACATCACCCACACCATCAACGAAGATGAACGTACCGAA
TTCACGCGCCACATCAACGCTGTCCTTGCGGGCGACCGCGACATCGGCAGCCGCCTGC
CTTTCCCGACCGACACC

> Sac6 (FG975)_*Asperigillus triticus*
ACTCGAAGATTATGTTGATGTTAGTGAAATATTCCCTGTTAGTACATATTTTGTAAGAGTT
ACTAATGGAGCAATATGACAGCTTATCTCTAAACTTCGTTTCACTTCAGGAAATAGCAA
AACTGCTCGTGACCCGAGCTCTCCTGGACCGGTCCCTGGAGGTGGAGCTGGCACGTCT
CGACATGTCTCCAAAGGCAGCATCGGAGGGAAAATCCAAGTGCAGGGTTCTTCTGCA
AACGTAACCCACACCATTAACGAAGATGAGAGAACTGAGTTCACAAGACACATAAATG
CTATCCTTGCTGGCGACCCGGATATCGGTGACCTTCTTCCATTTCCCACAGACACG
>Stt3 (MS561) _*Marasmius fiardii*
ATTTTCCTGCTCATGTTCACCTTTTACCTCTGGATCAAAGCGCTGAAATTGGGAAGTGC
TTTGTACGGTACCCTCGCCGCTATCTTTTACTTCTACATGGTGGCAGCTTGGGGTGGTTA

TGCGTTCATTACGAACATGATACCCATTCACGCCCTTGTTTTGATGCTCATGGGACGTTA
CAGTAGTCGACTCTACGTTGGTTACTGTTCGTGGTATGTCATCGGAACTCTCGCGTCGA
TGCAAGT

> Stt3 (MS561) _*Laccaria bicolor*
ATCTTCCTCCTCATGTTCACCTTCTTCTGCTGGATTAAAGCTCTGAAGCAAGGGAGCGC
ATTGTTCGGTACCATCGCTGCAGTGTTCTACTTCTACATGGTCGCGGCTTGGGGCGGAT
ACGTGTTCATTACGAACATGATACCCCTGCACGCTCTCGTCCTCATACTCATGGGCCGAT
TCTCTCACCGCCTCTACGTAGGGTACTCTTCCTGGTACGTTGTTGGGACTCTTGCGAGT
ATGCAAGT

> Stt3 (MS561) _*Agrocybe pediades*
ATCTTCCTTCTCATGTTCACCTTCTACTGCTGGATCAAGGCCCTGAAGCAAGGCAGCGC
GCTCTTTGGTACCATCGCTGCCGTCTTTTACTTTTACATGGTTGCAGCATGGGGTTAGTG
TTACATACCTCGAATTAGACCAGACCTTTGATTCTTCAGGTGGTTATGTTTTTATTACGA
ACATGATTCCTCTGCATGCGCTAGTCCTCATCCTTATGGGCCGCTTCTCTGACCGCCTAT
ATGTTGCGTACTCGTCATGGTATGCCATTGGTACATTATCTAGCATGCAAGT

> Stt3 (MS561) _*Auricularia delicata*
ATCTTCCTGCTCATGTTCACGTTCTACGCGTGGATCAAGGCCCTCAAGGTCGGCTCTGC
ATTCTTTGGCACCATTGCTGCTATCTTCTACTTCTACATGGTGGCTGCATGGGGTACGTC
CCACCTATGTCCACCCTGCCTTTGACTATCCGCGCTTAGGTGGATACGCCTTCATTACCA
ACATGATACCCCTCCACGCGCTCACGCTCATTCTTATGGGCCGCTTCAGCAACAGGCTC
TATGTCGCCTATTCCTCGTGGTATGCCATCGGCACTCTCGCTAGCATGCAGGT

> Stt3 (MS561) _*Pluteus cervinus*
ATTTTCTTGCTTATGTTCACGTTCTTTTGCTGGATTAAAGCCTTGAAGCTAGGAAGCGC
ACTCTTTGGCACCGTTGCAGCTGTTTTCTATTTCTACATGGTTGCTGCGTGGGGTACGTA
CCATCAACCCTCTCTTATTTACGTGCCTAGGTGGATATGCCTTCATCACCAACATGATCC
CTCTTCATGCCCTCGTACTGATCCTCATGGGCCGTTTCACAAGTCGCCTCTATGTAGCCT
ACTCTTCCTGGTATGCCATTGGTACGCTTGCTAGCATGCAGGT

> Stt3 (MS561) _*Mycena crocata*
ATCTTCCTGCTCATGTTTACCTTCTACTGCTGGATCAAGGCCCTCAAACTTGGCAGTGC
TCTATTCGGAACCCTCGCAGCCGTGTTTTACTTTTACATGGTGGCCGCCTGGGGTATGG
TCTCCGTGAACCGCTTACTAAATCATCGCAGGTGGATATGTCTTCATTACCAACATGATC
CCTCTACATGGACTGGTGTTGATCCTTATGGGCCGCTTCACCAGCCGCCTCTACGTTGC
ATACTCGTCGTGGTATGTCATCGGCACGTTGTCCAGCATGCAAGT

> Stt3 (MS561) _*Trametes versicolor*
ATATTTTTGCTCATGTTCACCTTCTACTGCTGGATTAAAGCTCTGAAGACCGGAAGCGC
CTTCTTCGGCACGCTAACCGCGCTGTTCTACTTTTACATGGTCGCCGCTTGGGGTGCGT
GCGGCTTAATATCTGCATCAGATATAACATACTCTTACAGGTGGTTATGCATTCATCACG
AACATGATCCCCCTACACGCCTTGACGCTGATCCTCATGGGGCGATTCAGCAGCCGCCT

GTACGTCGCATACTCGTCTTGGTACGCGATCGGGACCTTGTCGAGTATGCAAGT

> Stt3 (MS561) _*Gymnopilus chrysopellus*
ATCTTCCTTCTCATGTTCACGTTCTACTGCTGGATCAAAGCCTTGAAGCAAGGCAGTGC
TCTTTTTGGAACCTTGGCCGCCCTGTTCTACTTTTACATGGTCGCTGCATGGGGTATATT
TCTTGCAATACGTTGGCTTCGTTGACTATGTCTTGTCAGGTGGTTACGCTTTCATCACGA
ACATGATCCCTCTTCATGCTCTTGCTCTCCTCTTAATGGGACGGTTCTCTAGTCGTCTATA
TGTTGCGTATACCTCTTGGTATGCGATTGGAACCCTGGCGAGTATGCAGGT

> Stt3 (MS561) _*Serpula lacrymans* var. *lacrymans*
ATCTTCCTCTTGATGTTCACGTTCTTCAGCTGGATCAAGGCCTTGAAGCAGGGAAGCG
CGTTCTTCGGGACTATAGCTGCCGTTTTCTACTTTTACATGGTGGCCGCGTGGGGTAAG
CGGATATTATATCGTCCGTAGTCGGTGTTAATTTACACTAGGTGGTTATGTGTTCATCACG
AACATGATTCCTCTCCACGCTTTGGTTCTGTTACTCATGGGCCGCTTCACGAGCCGCTT
ATATGTCGCGTACTCGTCGTGGTATGCCATCGGTACCCTCGCGAGTATGCAGGT

> Stt3 (MS561) _*Coprinopsis cinerea*
ATCTTCCTGCTCATGTTCACCTTCTTCCTCTGGATCAAGGCTCTCAAGACTGGAAGCGC
GCTCTACGGAACCATTGCTGCTATCTTCTACTTCTACATGGTGTCGGCTTGGGGTGCGTT
CATCCCTCCTCTCGGACCGGTGGCTGACGAATGTGATAGGTGGATACGTTTTCATCACC
AACATGATTCCTCTTCACGCTCTGGTTCTCATGCTCATGGGTCGTTTCTCATCGCGTCTA
TACGCCGCCTACTGCTCCTGGTACGCCATTGGCACTCTCGCAAGTATGCAAGT

> Stt3 (MS561) _*Hygrophoropsis aurantiaca*
ATATTCCTGCTGATGTTCACTTTCTTTACCTGGATCAAAGCTCTCAAGCAAGGAAGCGC
ATTTTTTGGAACTATTGCTGCTGTATTCTACTTCTACATGGTCGCCGCATGGGGTTAGTT
GCAATGTTTCATGCATACTTGATAACCCATCTCCATCAGGTGGATACGTGTTTATCACCA
ATATGATTCCTCTTCATGCACTGGTTCTGTTGCTCATGGGACGGTTCACCAGCCGTCTCT
ATGTCGCCTACTCATCTTGGTACGCGATTGGTACATTGGCGAGCATGCAAGT

> Stt3 (MS561) _*Agaricus bisporus* var. *bisporus*
ATCTTCCTTCTTATGTTCACTTTTTACCTCTGGATCAAAGCCATCAAGATCGGCAGTGCG
CTTTATGGTACCCTAACCGCTGTTTTCTACTTCTACATGGTTGCTGCCTGGGGTGAGTCT
CTCTCTCCACACCTGTTACTAGACCGAAAATAACTCAGGTGGTTATGCCTTCATCACAA
ACATGATTCCCTTACATGCACTCGTCCTTATCCTCATGGGCAGGTTCTCCAACCGTCTCT
ATGTCGGGTATTCTTCGTGGTATGCTATTGGAACACTTGCTAGCATGCAAGT

> Stt3 (MS561) _*Lactarius quietus*
ATTTTTCTCCTCATGTTCACCTTCTTCCTCTGGATCAAAGCTATGAAGCAAGGCAGTGC
CTTGTATGGCACTCTCGCAGCCATTTTCTACTTCTACATGGTTGCAGCTTGGGGTGAGT
CTCTAATTTGGTGCGGATGACCATCATGTATGTTTCGCAGGTGGCTATGCATTCATCACG
AACATGATCCCGTTGCACGCTCTCGCCCTCACACTGATGGGTCGTTACACAAACCGCCT
TTATGTGGGCTACTCGTCATGGTATGCGATCGGTACATTGGCGAGCATGCAGGT

> Stt3 (MS561) _*Boletus edulis*
ATCTTCCTTCTCATGTTCACGTTTTTCTGCTGGATCAAAGCACTCAAGCAGGGTAGTGC
ATTTTTCGGCGCCGTTGCCGCCGTCTTTTACTTTTACATGGTTGCCGCATGGGGTTCGTG
TGCATTCTTCTGATACTCCCGGTCCATCATCCTTCCCAGGCGGTTATGTGTTCATCACTA
ACATGATCCCTCTACACGCACTTGTCCTCCTTCTTATGGGTCGATACTCCAGTCGCCTGT
ACGTTGCATACTCCACCTGGTACTCTATCGGCACACTCGCGAGTATGCAAGT

> Stt3 (MS561) _*Phanerochaete carnosa*
ATTTTCCTCCTCATGTTCACCTTCTTCTGTTGGATCAAGGCGTTGAAGCTTGGGAGCGC
TTTCTTTGGTACCTTAGCGGCGCTATTCTATTTTTACATGGTGGCGGCTTGGGGTGAGAG
TCCGTCTACTATGTATGCTCTGGAGATTGGACTTCGCAGGCGGTTACGCTTTCATCACG
AACATGATACCGCTCCATGCGCTAACGCTCATTCTGATGGGCAGGTTCAGTAGCAGACT
TTATGTTGCGTATTCGTCATGGTATGCTATCGGAACGCTGGCCAGTATGCAGGT

> Stt3 (MS561) _*Fomitopsis pinicola*
ATCTTCTTGCTCATGTTCACGTTCTTCCTATGGATCAAGGCACTCAAGACGGGCAGTGC
CTTGTTTGGCACTCTGGCTGCGCTGTTCTACTTCTACATGGTAGCCGCTTGGGGTAAGA
TTTGATCGTTCCTCGCTACGATGCTGACCCTGTGGTCTAGGTGGCTACGCCTTCATTACA
AACATGATTCCGCTGCATGCCTTCGCGCTGATACTCATGGGACGCTTCAGCAGCCGCCT
GTATGTCGCGTACTCGTCGTGGTACGCGATTGGCACGCTGGCCAGCATGCAGGT

> Stt3 (MS561) _*Fomitiporia mediterranea*
ATCTTTCTCCTCATGTTCACCTTCTTCCTCTGGATAAAGGCACTCAAGACTGGTAGCGC
GCTTTTCGGAGTTGGTGCAGCCCTATTCTACTTTTACATGGTGGCAGCTTGGGGGGTTA
CGCCTTCATCACAAACATGATTCCGCTGCACTCTCTTGTCCTGCTCCTTATGGGTCGGTA
CTCAAGCCGTCTGTATGTTGCATATTCGTCTTGGTACGCCATCGGTACACTTGCAAGTAT
GCAAGT

> Stt3 (MS561) _*Ramaria acris*
ATCTTTCTTCTTATGTTCACATTTTACTTGTGGATCAAAGCGCTCAAAGACGGTAGTGCC
TTCTTTGGTACAGGGGCTGCAGTGTTCTACTTCTACATGGTGGCCGCTTGGGGTTAGAC
TTTTTCTAGGCAATCTGAGTGGTGAGCTCTGTATTTCAGGTGGTTATGCATTCATCACCA
ACATGATACCCCTTCACGCCCTAACACTCATTCTGATGGGCCGTTTCACCAACCGTTTAT
ACATCGCCTATTCTTCATGGTATGCTGTAGGCACGCTTGCAAGTATGCAGGT

> Stt3 (MS561) _*Gautieria morchelliformis*
ATTTTCCTTCTAATGTTCACCTTCTATCTCTGGATCAAAGCATTAAAGCAAGGCAGCGCA
TTCTTCGGCACTGGTGCTGCAGTATTCTACTTCTACATGGTGGCTGCTTGGGGTCAGAG
ATCCTTCTAGATTATGTTGCTGTGAACTCCATTTTATAGGTGGTTATGCTTTTATAACCAA
CATGATCCCACTTCATGCCTTGGCACTTATTTTGATGGGGCGCTTCACCAACCGCTTGTA
CATTGCCTACTCTTCGTGGTACGCTGTGGGTACTCTGGCGAGTATGCAAGT
> Stt3 (MS561) _*Amanita muscaria*
ATCTTCTTGCTCATGTTCACGTTCTTTTGCTGGATCAAGGCATTGAAGAGCGGAAGTGT
ACTGTTTAGCACAGTAGCAGGGGTGTTTTACTTCTACATGGTTGCTGCCTGGGGTACGT

GTCCATTTTCCTTTTCTCTACTTCTGACGTTCTGTGGTAGGTGGCTACGTGTTTATTACC
AATATGATCCCTCTTCATGCCCTAGTTCTTTTGCTTATGGGTCGCTTCTCAAGTCGACTAT
ACGTCGCTTACTCTTCCTGGTATGCCATAGGAACACTAGCAAGCATGCAAGT

> Stt3 (MS561) _*Gymnopus androsaceus*
ATCTTCCTGCTGATGTTCACATTTTATTGCTGGATCAAAGCGCTCAAGCTAGGTAGTGC
GTTCTTCGGTACGCTTGCTGCAGTCTTCTACTTTTACATGGTGGCCGCTTGGGGGTTAT
GCGTTCATCACCAACATGATTCCAGTACACGCACTCGTTCTTCTTCTCATGGGTCGTTTC
AGCGGTAGACTCTATGTCGCATACAGCTCCTGGTACGCTATCGGTACACTGTCCTCCAT
GCAGGT

> Stt3 (MS561) _*Calocera cornea*
ATCTTCTTGCTAATGATCACGTTCTACATCTGGATCCGAGCACTAAAGGATGGTAGTGTC
CTGTACGGGACATTGGCTGCCGTATTTTATTTCTACATGGTTGCTGCATGGGGTGAGCG
CTACTGCGGCTGCCGTGTATGCTCCTAACTGAAATGCAGGTGGATACGCCTTCATCACC
AACATGATCCCTCTGCATGCTTTCGTGTTGCTACTGATGGGTCGATATTCCGATCGCTTG
TATTCGGCATATTCGGCATGGTACGCAGTGGGGACGCTCGCTAGTATGCAAGT

> Stt3 (MS561) _*Dacryopinax primogenitus*
ATCTTCCTGCTCATGATCACCTTCTATATTTGGATCAGAGCGCTGAAAGATGGCAGTGTC
CTGTATGGGACCCTTGCCGCTGTATTCTACTTTTACATGGTTGCAGCTTGGGGTAAGCTT
AATCATTGCTATTATATGACCTTATTCATGCACACTAGGTGGATACGCGTTCATTACGAAT
ATGATTCCTCTGCATGCTTTCGTGCTTCTTTTGATGGGCCGCTTCTCCCATCGCTTATACT
CCGCTTATTCGGCTTGGTATGCCGTTGGCACACTTGCCAGCATGCAAGT

> Stt3 (MS561) _*Rhizopus microsporus*
ATCTTTTTGCTCATGTTTACGTTTTACTTGTGGATCAAGGCGCTCAAATTAGGATCCGCC
CTTTGGGCCTCTGCATCCGCCTTCTTTTACTTTTATATGGTAGCTGCTTGGGGCGGTTAT
GTGTTCATCATCAATTTGATTCCTCTTCATGTCTTTGTGCTTATGCTCATGGGTCGTTTCT
CTAACCGTGTCTACGTGTCTTACTCGACCTTTTATGTCCTGGGTACTTTGATGTCAATGC
AGAT

> Stt3 (MS561) _*Asperigillus triticus*
ATTTTCCTGCTCGTCTTTACCTTTTTCCTGTGGATCAAGGCTGTCAAGAATGGCTCGATC
ATGTGGGGCGCGCTCACCGCGCTGTTCTACGGCTACATGGTGTCAGCCTGGGGTGGTT
ACGTCTTCATCACCAACCTGATTCCCCTGCACGTCTTCGTCCTTCTCTGCATGGGGAGA
TACAGCTCCCGTCTGTACATCAGCTACACGACCTGGTATGCTTTGGGAACGTTGGCTAG
CATGCAGAT

> Stt3 (MS561) _*Neurospora crassa*
ATTTTTCTGCTGGTCTTCACCTTCTACCTGTGGATCAAGGCTCTCAAGCTCGGATCCATG
CTTTGGGGAGCTCTTTGCGCCCTCTTCTACGGTTACATGGTTGCTTCCTGGGGTGGTTAT
GCCTTCATCACCTGTCTCCTCCCCCTCCACGCATTTGTTCTTATCTGCATGGGTCGCTAC
AGCACTAGGCTTTATGTTGCGTACACCACTTGGTATGCATTGGGAACATTGGCCAGCAT

GCAGAT

> Stt3 (MS561) _*Agaricostilbum hyphaenes*
ATATTTATCCTGACATTCACCTTTTACCTTTGGCTCAAGGCTCTGCGAGAGGGCTCGGC
ACTGTACGGCACGCTAACAGCTCTCTTTTATTTCTACATGGTCGGCGCATGGGGTAAGC
GATCCTGCTGATGGGTCGATATCGATGAACCCACGTACAGGTGGTTTTGCCTTCATCAC
CAACATGCTCCCTCTGCACGTCTTTGTCCTCTTGCTGATGGGCCGCTTCACCACCAAGA
TGTATGTCGCCTACTCGACCTACTACGTCATCGGCACACTAGCAGCCATGTCCGT

>Trp2 (MS353)_*Amanita muscaria*
GGTTTGCAGATTGTGGGTGCCAGCCCGGAAACATTGTGCAAAGTCGAGAGGAATGTTG
TCTACAATCATGCTATCGCTGGTACTACCCGGCGCGGCAAGACTCCTGAAGGTTTTCTC
CGTTTAGTGTGTGTGTTAATGTCGTCTAATGCATTGGTGCAGAGGATGAGAAATTGGGA
GCTGCCTTGCTCGAATCTGAAAAGGATCGAGCAGAACATATTATGCTTGTTGATCTGGC
TCGCAACGATGTCAATCGCGTCTGTCAGCCCAAAACAGTCAAGGTTGACCATCTTATG
AAACTAGAAAAG

> Trp2 (MS353)_*Laccaria bicolor*
GATCTGCAAATCGTTGGCGCAAGCCCGGAAACCTTATGCAAGGTTGAAAAGAATGTTG
TTTACAACCACGCAATCGCTGGCACGATAAAACGAGGAAAGACACCAGAAGGTTCGT
TGTTTGGGTTTTGCCACCAACATTTGCTCACTTTAAGTAGAGGATGAAAAACTCGGGG
CCACTCTGTTAGCTTCAGAAAAAGATCGTGCTGAACATATCATGCTGGTTGATCTCGCG
CGGAACGATGTCAACCGAGTGTGCCAGCCTAAAACAGTCAAGGTTGACCACCTGATG
AAGGTTGAGAAA

> Trp2 (MS353)_*Tricholoma matsutake*
GACATCCAAATCGTTGGTGCCAGCCCTGAAACATTATGCAAAGTAGAAAGGAATGTCG
TGTACAACCATGCGATTGCGGGAACTACAAAGCGGGGAAAGACGCCGGAAGGTACGT
GGCTTATTCACTGCACCTTTAATTGGAATGCCATTGCAGAGGACGAGAAGTTGGGTGCT
GCACTGCTTGCTTCAGAGAAGGATCGAGCAGAGCATATCATGCTTGTCGATCTGGCAC
GAAACGATGTGAATCGTGTCTGTCAGCCGAAAACAGTCAAGGTAGACCATCTGATGAG
AGTTGAAAAA

> Trp2 (MS353)_*Pluteus cervinus*
GATCTACAAATCGTTGGTGCAAGCCCGGAAACTCTTTGCAAGGTGCAGAAGAATGTGG
TATTCAACCACGCCATCGCGGGCACCACCAAGAGGGGAAAGACGCCTGAAGGTGATTA
TATCCAACTGTAGCCAAGTTCTGATACACTTCGTTGATAGAGGACGAGAAACTTGGTGC
TGAGCTATTGGCGTCCGAGAAGGATCGTGCAGAACACATCATGTTGGTTGATCTAGCA
CGGAACGATGTCAATCGCGTTTGCGATCCAAAGACCGTCAAGGTTGACCATCTCATGA
GGGTAGAAAAG

> Trp2 (MS353)_*Agaricus bisporus* var. *bisporus*
GACTTCCAAATCGTCGGTGCAAGCCCAGAAACCATGTGTAAAGTCGAGAAGAACGTA
GTTTTCAACCATGCTATCGCTGGAACTGTAAAACGAGGCAGAACACCTGAAGGTATCC

CTACTCAGGATTGCACGATGGATTTGACTGACATGTTATTGTAGAGGACGAGAGATTGG
GGGCCGAGCTCCTAGCCTCAGAAAAGGATCGGGCAGAACACATCATGCTTGTCGATCT
TGCACGAAATGATGTCAATCGCGTCTGTCAGCCCAAGACCGTCAAGGTTGATCACTTA
ATGCAAGTTCAAAAG

> Trp2 (MS353)_*Coprinopsis cinerea*
GATATGCAGATCGTCGGCGCCAGCCCCGAAACTCTTTGCAAAGTCGAGAAGAACGTGG
TCTATAACCATGCCATCGCTGGAACCATCAAGAGGGGCAAGACCCCCGAAGGTCAGTT
AATCCATTTCAGGCTTGAAATCGTCGCTCATACTCGGTTTCAGAGGACGCCAAACTCGG
TGCCATCCTTCTCAATTCTGAGAAAGACCGCGCGGAGCACATCATGTTGGTTGACTTG
GCCCGGAACGACGTCAATCGTGTCTGTCAGCCCAAAACGGTCAAAGTGGACCACCTC
ATGCAGGTGGAGAAG

> Trp2 (MS353)_*Gymnopilus chrysopellus*
GACCTCCAGCTCGTTGGAGCGAGCCCGGAGACCCTGTGCAAGGTAGAGAAGAACGTC
GTATACAACCATGCCATCGCAGGTACCATCAAGAGGGGAAAGACACCAGAAGGTCAG
GGACAGGTCTCCAACTGCGTAACTTCTGCTAATAGTATTTCAGAGGATGAGAAATTGGG
TGCTATACTCCTTGCATCGGAGAAAGACCGAGCGGAGCACATCATGCTTGTAGACTTAG
CTAGGAACGACGTGAATCGCGTCTGCCAACCCAAGACCGTCAAAGTCGATCATTTGAT
GAAGGTCGAGAAG

> Trp2 (MS353)_*Agrocybe pediades*
GGATTACAAATTGTCGGTGCAAGCCCGGAAACTTTATGCAAGGTTTCAAAAAACGTTG
TCTACAATCACGCAATCGCGGGGACCATCAAGAGGGGAAGAACCCCGGAAGGTGAGT
AATTTACCCAAATGATGTTTGCATATGTTAAACTTCGTGATCAGAGGATGAGAAGCTGG
GTGCTATTCTCCTTGCTTCTGAGAAGGATCGAGCAGAGCATATCATGCTTGTCGATCTT
GCTCGCAACGATGTGAACCGTGTCTGCCAGCCCAAGACAGTGAAGGTAGATCATTTGA
TGAAGCTGGAGAAA

> Trp2 (MS353)_*Gymnopus androsaceus*
GACCTACAAATTGTCGGTGCTAGCCCAGAGACTCTGTGCAAGGTGGAAAAGAACACT
GTTTTCAATCATGCCATTGCAGGAACCACGAAGCGAGGGAAGACGCCAGAGGGTGAG
TTCATCGTCGTTATTATTAACACAATATCTGAATCCTTTATCCAGAGGACGAACAGCTCG
GTGCGACTCTACTCAACTCTGAGAAGGATCGCGCAGAGCATATCATGCTGGTCGACCT
GGCTCGCAATGATGTAAATCGCGTGTGCGACCCGAAGACGGTAAAGGTTGATCACCTC
ATGAGGCTTGAAAAG

> Trp2 (MS353)_*Schizophyllum commune*
GACGAGCAGATTGTTGGTTCGAGTCCCGAGACGCTCTGCAAGGTGGAGGCAAACACT
GTCTTCAACCACGCGATCGCAGGCACTACACATCGCGGGCGGACACCCGAGGAGGAC
GAGGAGCTTGGGAAGACGCTGCTCGCCTCGGAGAAGGACCGCGCGGAGCACATCATG
CTGGTCGACCTCGCACGAAACGACGTCAACCGCGTGTGCGACCCGAAGACCGTGAAG
GTCGACGAGCTCATGAAGCTCGAGAAG

> Trp2 (MS353)_*Auricularia delicata*
GACCTCCAGATTGTCGGCGCGAGCCCGGAAACGCTGTGCCGCGTCGAACGCAACAAA
GTTTACAACCACGCCATTGCGGGCACAACGCACCGCGGAGAGACGGCCGAAGGTGCG
CGCTAGCGTCGTTCACTTTTAGCCTCGGCCGCTGGCGCGCCGCAGAGGACGAGCAGCT
GGGCAAGGAGCTGCTCGCATCCGAGAAGGACCGCGCGGAGCACATCATGCTCGTCGA
CCTCGCGCGCAACGACGTGAACCGGGTGTGCGACCCGGCGACGGTGAAGGTGGACG
ACCTGATGCGGCTCGAGCGC

> Trp2 (MS353)_*Mycena crocata*
GGCGTTCAGATTGTCGGAGCTAGTCCGGAAACGCTCTGCAAGATTCAAAAGAACGTCG
TATACAATCACGCAATTGCAGGGACCACCAAGCGGGGCAAAACCCCAGAGGGTCAGT
TCACTTGCACAAACATCCTGATCGTCTAAGCGTCGTCCCCAGAGGATGAAAAATTGGG
CGCAGCTCTTCTCGCTTCGGAAAAAGACCGCTCGGAGCATATCATGCTGCTGGATCTTG
CCAGAAATGACGTGAATCGTGTCTGCAAACCAAAAACGGTCAAGGTCGACCATTTGAT
GAAGCTAGAAAAA

> Trp2 (MS353)_*Hygrophoropsis aurantiaca*
GAACTTCAAATCGTGGGGGCTAGTCCTGAAACGCTGTGTAAAGTGGAAGCTAATAAGG
TGTTCAACCATGCTATAGCTGGAACTGTCAAGAGAGGGAAAAGCCCGGAAGGTTGGTT
AGACATTCCAAGATTGTTTCAATATGCTGACCGATTATCGCAGAGGATCAGAAACTCGG
AGAACAACTCCTCGCTTCTGAAAAGGATAGAGCAGAGCATATCATGCTTGTTGACCTT
GCGCGCAATGATGTCAACCGAGTTTGCAAACCTGAAACAGTCAAAGTAGACCATCTCA
TGCAGGTCCAAAAG

> Trp2 (MS353)_*Serpula lacrymans* var. *lacrymans*
GAACTTCAGATCGTTGGTGCCAGTCCGGAGACCCTGTGCAAAGTGGAAAGCAACAAA
GTATTCAACCACGCTATTGCAGGCACCATCAAGAGAGGGAAGAGTCCAGAAGGTTTGC
ATTGTTTTCCAAGAGTATTGTTAGAACTTATCGTTGATCTGTAGAGGATGTAAAGTTGGG
AGAAGAGCTACTGTCTTCTGAGAAGGACCGTGCCGAACATATTATGCTTGTTGACCTG
GCTCGGAACGATGTGAACCGTGTGTGCCGACCAGAAACCGTCAAGGTTGACCACCTC
ATGCAGGTGCAAAAA

> Trp2 (MS353)_*Phanerochaete carnosa*
GGCCTCAAAATAGTAGGTGCAAGTCCGGAGACACTTTGCAAGGTGGAGAAGAACAAA
GTGTTCAACCACGCCATTGCGGGGACTGTCAAGCGTGGGAAGACTCCAGAAGGTATGT
ATTTGAGCTTCCATCATACGCTATGTGTTGAAATCGCCGCGCAGAGGACGAGAGGCTTG
GTCAAGAGCTTCTGAACTCTGAGAAGGATCGTGCAGAGCATATTATGCTCGTGGATCTG
GCTCGGAACGATGTCAACAGGGTCTGCCAACCGAAGACAGTCAAGGTAGACCACCTG
ATGCGCCTAGAGCGC

> Trp2 (MS353)_*Boletus edulis*
GACCTGCAGATTGTTGGCGCCAGCCCGGAGACACTCTGTAAAGTGGAGGCGAACAAG
GTATACAATCATGCTATAGCAGGTACCACGAAGAGAGGCCAAACACTGGATGGTTCGTT

CCATTAGTCCTCATTTGCCGTTTGACATGATAGTATAGAGGACAACTTACTAGCGGCAC
AATTAACTGCATCGGAGAAGGACAGAGCAGAACATATCATGCTTGTCGATCTTGCACG
AAACGATGTTAATCGCGTGTGCAAACCAGAGACTGTTCAAGTCGACCAGCTCATGCAG
GTTCAAAAA

> Trp2 (MS353)_*Lactarius quietus*
GAGCTGCAGATCGTCGGCGCTAGCCCAGAAACGCTGTGTAAAGTAGAGGCGAACAAG
GTCTACAACCACGCCATCGCTGGAACGACTAAACGCGGTGAAACTGCCGAAGGTGCG
CTATCTACCACTCATTGATGTGGACCCCTAAAATTTGTGAACAGAGGATCAACGGCTGG
GTGAGGAGCTGCTTAATTCGGAGAAAGATCGTGCCGAGCACATCATGCTGGTTGACCT
CGCGCGCAACGATGTCAATCGTGTCTGCCAGCCCAAGACGGTCAAAGTGGATCATCTG
ATGAGGTTAGAAAGG

> Trp2 (MS353)_*Stereum hirsutum*
GGGTTGCAGGTCGTCGGGGCTAGCCCAGAGACGCTGTGTAAAGTGGAGAGCCGAAAG
GTTTACAACCATGCCATTGCGGGTACGACAAAACGCGGAAAGACACCAGAAGGTGAG
ATTGATCTCTTTCTTGATTCGACGTGGGCTAATCTGTCTGTTTAGAGGATAGGTTACTGG
GAGAAGAGCTTCAGAAGTCCGAGAAGGACCGAGCGGAACACATCATGCTCGTCGACC
TCGCACGGAATGACGTTAACCGAGTCTGCCAACCGAAGACGGTCAAAGTCGACGAGC
TAATGAAGCTGGAAAAG

> Trp2 (MS353)_*Fomitopsis pinicola*
GAACTGCAGTTGGTCGGAGCTAGCCCGGAGACACTCTGCAAGGTGGAGAAAAACAAA
GTGTTCAACCACGCCATTGCGGGGACCGTTCGGAGGGGCCTCACAGTGGAAGGTTGG
TATATATACCTAACCCCCTGTCGCGACATTCCCGTAGAGGACGAGATGCTAGGACAGCA
GCTCCTGAACTCAGAGAAAGACAGGGCGGAGCACGTCATGCTCGTAGACCTTGCGCG
TAACGACGTCAACCGTGTCTGCCAACCGAAGACCGTGAAAGTCGATCATCTCATGCGG
CTAGAGAAA

> Trp2 (MS353)_*Marasmius fiardii*
GATCTTCAGATTGTTGGTGCCAGTCCAGAGACGATGTGCAGAGTTTCCAAGAACGTCG
TTTTCAATCATGCTATCGCAGGAACAACAAAGCGAGGAAAGACGCCTGAAGGTAACA
CATTTCTCTCTGTCACAACAACGACGGGTAATACTTCTCTGCAGAGGATGAAGCCCGT
GGAGCAGAACTTCTTGCCTCAGAAAAGGATAGAGCAGAACACATCATGTTGGTCGACT
TGGCTCGAAACGATGTCAACCGAGTCTGTGACCCTAAAACCGTCAAGGTTGACCACTT
GATGACGCTTGAGAAG

> Trp2 (MS353)_*Fomitiporia mediterranea*
GATGTACAAGTCGTAGGCGCGAGTCCAGAGACACTCTGTAAGGTGGAACGGAACAAG
GTTTACAATCATGCTATCGCTGGCACGACGAAACGAGGTAAAACTCCTGAAGGCAAGT
AGAGTTGGCTTCCCTTGTAGTTTCCCGTTATCTATTTGATGCAGAGGACGAGAGGCTCG
GGAACGAGCTTCAATCGTCTGAGAAGGACCGCTCCGAGCATATCATGCTTGTTGACCT
TGCGCGGAACGATGTAAATCGCGTATGCCAAGCGAAGACCGTGAAAGTGGACCATCTC
ATGCGCTTGGAAAAG

> Trp2 (MS353)_*Trametes versicolor*
CACTTGCAGATCGTCGGCGCGAGTCCGGAGACGCTTTGCAAGGTCGAGAAGAACAAG
GTGTACAACCACGCTATCGCGGGTACAATCCGAAGAGGACAAACTGTTGAAGGTCTGT
CCCTTGTCCTGTTTGTTGTTACTGAAACAAATTGCATTCTGCAGAGGACGAGAAGCTC
GGTGCCGAACTGCTCGCATCAGAGAAAGACAGAGCAGAACATATCATGCTGGTCGATC
TTGCACGAAACGACGTCAACAGGGTAGCCAGGCCTGAAACCGTGCACGTCGACCACC
TTCTACGACTGGAGAAG

> Trp2 (MS353)_*Ramaria acris*
GACCTGCAGATTGTCGGTGCCAGCCCAGAAACTCTCTGCAAGGTTGAACGCAACAAA
GTGTACAATCACGCCATCGCTGGGACGGTGCGGAGAGGAAAAACACTGGAAGGTAAG
ATGATCCCCCTCCCCCTCTTGCGGACGTTTATAACCGTGATACAGAGGATGAAAAGCTC
AGCGATCAATTGGCGAATTCTACCAAAGACCGTGCAGAGCACATTATGCTTGTGGATTT
GGCGCGAAACGATGTCAACCGCACGTGCCAACCAAAGACAGTGAAAGTCGAGAAATT
GATGGAAGTGGAGAAG

> Trp2 (MS353)_*Gautieria morchelliformis*
GAGGTGCAAATTGTAGGTGCTAGCCCGGAAACACTCTGCAAAGTCGAGCGAAGAAAA
GTATTCAACCATGCAATTGCAGGGACCGTGCGCAGAGGAAAGACACCAGAGGGTACG
GAGATCCTGGCTCTATCTTCTTGTCTTTGTGACATTATATCACAGAGGATGAGAAGCTCA
GTGCGCAATTGTCCAATTCTAACAAAGATCGCGCCGAGCATATCATGCTTGTCGACCTG
GCCAGGAATGACGTTAACCGCGTCTGTCAGCCCAGCACCGTCAAAGTTGAGAAACTG
ATGGAGGTGGAGAAG

> Trp2 (MS353)_*Calocera cornea*
GATGTGCAGCTTGTAGGTGCAAGCCCAGAAACTCTCTGCAAGGTTTCGAAGAACAAG
GTGGAGAACCATGCTATCGCAGGGACTGTCAGGAGGGGGAAGTCAGCGAAAGGTCCG
TCATGCGTTATTGATGCCTTACAGGCTCTTAACCATATTTGATAGAGGATGAGGAGCTTG
GTGCTCAACTACTTGCCTCGGAGAAAGATCGCGCAGAGCACATCATGCTTGTCGACCT
TGCACGAAACGATGTCAACCGAGTCTGCAAGCCCGAGACTGTCAAGGTGGACGAGCT
TATGAAACTGAAGCTC

> Trp2 (MS353)_*Dacryopinax primogenitus*
GATATACAACTGGTCGGTGCCAGCCCGGAGACGCTTTGCAAGGTTACGCGAAACAAAG
TGGAGAACCACGCTCTCGCCGGAACGGTGCGCAGGGGCAAGTCCCCAAAAGGTAAGC
TTCGCATCCCCGTGGCGATATGGGCATATGCAAACGCTCCGCAGAGGATGAAGAACTT
GGTGCGCAACTGCAGGCGTCGGAGAAGGACCGCGCGGAGCATATCATGCTGGTCGATC
TTGCCCGCAATGACGTAAATCGGGTGTGCAAGCCCGAGACGGTCAAAGTGGAAGAGC
TTATGAAGCTCAAAATG

> Trp2 (MS353)_*Asperigillus triticus*
GACTTCCAGCTGGTGGGAGCCAGCCCCGAGCTGCTGGCCAAGGAGGAGAAGGGGCG
GATGATCACGCACCCGATCGCGGGCACGGTGAAGCGGGGCAAGACAGCGGAGGAGG

ACGACGCGCTGGCGGCGGAGCTGCGCAGCAGCCTCAAGGACCGGGCGGAGCACGTG
ATGCTGGTGGACCTGGCGCGCAACGACGTGAACCGGGTGTGCGACCCGACGACGACG
CAGGTGGACCGACTGATGGTGGTGGAGAAG

> Trp2 (MS353)_*Agaricostilbum hyphaenes*
GGCTTGTGTCTGGTGGGCGCATCGCCCGAGTGCTTGTGTGCCGTCAACAAGAATGTCG
TGACGAACCACGCTATCGCCGGGACAGTCAAGCGAGGTGCTACAGAAGAAGGTCGGT
GGTCCCTCAGCTTGCTCTTGGCGTTGTTGAAAGGCCGTTGGCAGAGGATGAGATTTTA
GGCGCAGAACTCAGCGCATCCGAAAAAGATCGTGCAGAACACATCATGTTGGTCGATC
TGGCCCGCAACGATGTCAACAGAGTCTGCGAGCCGGCCAGCGTCAAAGTCGACTCAC
TCATGCAAGTCGAGAAA

> Trp2 (MS353)_*Neurospora crassa*
GACTTCCACATTGTTGGCGCCTCGCCCGAATGCCTGATGAAGACCGACGGCTATGCCG
TCGTCAACCACGCCATTGCCGGTACCATCAAGCGTGGCCTCAACTCGGAGGAGGACGA
TGAGCTTGCGGCCGTGCTGCTGGCCTCAACCAAGGACCGCGCCGAGCACGTCATGTTG
GTCGATTTGGCCCGCAACGATGTCAACCGTGTCTGCCACCCGTCTACCGTCAAGGTCG
ACCGCCTCATGCGCATCGACCGT

> Trp2 (MS353)_*Rhizopus microsporus*
GAATTCACTCTTGTAGGTGCTTCACCCGAGATGCTGACTAAGGTCCAGGATCGTATTGC
CTTCACTCATCCTATTGCCGGCACACGAAAGCGAGGAAAGACCCCAGAAGAGGATAAA
TCCTTGGCAGAAGATCTCTTGAAGGATCCCAAGGAAATTGCTGAGCATGTCATGTTGG
TCGATCTCGGACGTAACGATATCAATCGTATCTGTAAGCCAGAGACAGTCAAGGTCGAT
AAGCTGATGCAAATCGAGTAT

>Uba1 (FG848)_*Agrocybe pediades*
ACCAACCACCACATTGATTTCATCACCGCTGCTTCCAACCTTCGTGCCATGAACTACAA
CATTAATCCGGCCGATAGACATACCACGAAGCAAATCGCGGGCAAGATTATACCTGCTA
TTGCTACTACCACCTCTCTCGTTACTGGATTGGTATGTTTGGAGCTCTACAAGGTATATA
TTAACACAATACTTCACGTGCCCTAACATCATTCTAGATCATTGACGGAAAGAAGAAGC
TCGAAGATTACAAGAACGGCTTCGTCAACATCGCTCTGCCC

> Uba1 (FG848)_*Fomitiporia mediterranea*
ACTAACCATCACATCGATTTCATCACCGCCGCTTCTAACCTTCGGGCTATGAACTACAGT
ATTTCTATCGCGTCTCGCCACCAGACGAAGCAGATTGCGGGAAAAATCATCCCTGCAAT
TGCGACAACGACTTCATTAGTGACTGGGCTCGTGTGTCTGGAGCTTTACAAGATTATCG
ATGGGAAGAGCAAGCTCGAAGATTACAAGAATGGATTTGTCAATCTAGCTTTGCCA

> Uba1 (FG848)_*Stereum hirsutum*
ACGAACCACCATATCGACTTCATCACCGCCGCCTCCAACCTCCGCGCGATGAACTACG
GCATCAACCCCGCCGACCGTCACACGACGAAGCAGATCGCGGGCAAGATCATCCCCG
CGATCGCCACGACGACGTCGCTCGTCGTTGGCCTCGTCTGTCTCGAGCTGTACAAGGT
CATCGACGGCAAGAACAAGCTCGAGGACTACAAGAACGGGTTCGTCAATCTCGCGTT

GCCG

> Uba1 (FG848)_*Trametes versicolor*
ACGAACCACCACATTGACTTCATTACCGCATCCTCGAACCTGCGCGCTCTCAACTACAG
CATCACGCCCGCGGACCGCCACACGACAAAGCAGATTGCGGGCAAGATCATCCCCGC
GATCGCGACGACTACGTCGCTGGTGACCGGCCTCGTATGTCTTGAGCTGTACAAGCTCA
TCGACGGCAAGAAGAACATTGAGTCGTATAAGAACGGCTTCGTGAACCTCGCCCTGCC
G

> Uba1 (FG848)_*Phanerochaete carnosa*
ACCAACCACCACATTGACTTCATCACCGCTGCGTCCAACTTGCGTGCTATGAACTACAG
CATCAACCCCGCCGACAGGCACTCGACCAAACAGATTGCAGGCAAGATCATCCCCGCT
ATCGCCACCACTACCTCGCTTGTCGTCGGTTTGGTCTGCTTGGAGCTGTACAAGGTGCG
TGTTGCGCGTCTTGACGAACCTTGCTCAATGTATCCCAGATTATCGACGGCAAGAACAA
GCTCGAGCAGTATAAGAACGGCTTCGTCAACCTCGCGCTACCC

> Uba1 (FG848)_*Auricularia delicata*
ACGAACTTCCACATCGACTTTGTGACCGCTGCGTCGAACCTCCGTGCGACGAACTACA
GCATCCCAATCGCCGATCGTCATACGACGAAGCAGATTGCGGGCAAGATCATCCCGGC
CATCGCGACGACGACCGCGCTCGTCACTGGCCTGGTCTGCCTCGAACTCTACAAGGTA
CTTCATCCCTATGAAACATATTCAACGCGCAGATCATCGACGGCAAGAACAAGCTCGAT
GAGTACAAGAACGGCTTCGTCAACATCGCGCTGCCG

> Uba1 (FG848)_*Lactarius quietus*
ACCAATCACCACATCGACTTTATCACCGCCGCTTCCAACCTTCGCGCGATGAATTACAG
CATCACGCCCGCTGATCGCCACACCACCAAACAAATCGCCGGGAAGATCATTCCCGCA
ATCGCCACGACGACGTCCCTTGTTGTGGGTTTGGTCTGCCTAGACCTTTTAAAGGTAAG
AAGTTGCCAATCCGTTATCGCCTTCCGCGCTGTTATTAGATCATTGACGGCAAGAACAA
GCTCGAAGACTACAAGAACGGTTTCGTCAACTTAGCCCTCCCG

> Uba1 (FG848)_*Coprinopsis cinerea*
ACGAACCATCACATCGACTTCATTACTGCCGCTTCTAATCTGCGAGCCATGAACTATGG
CATCAACCCCGCAGACAAACATACCACGAAGCAGATTGCCGGAAAGATCATCCCTGCA
ATCGCCACCACTACCTCGCTGGTGACAGGTCTGGTCTGCCTCGAATTGTACAAGGTATG
AATTATGGAATGTTCGAAAACATGCTGACGACTCATTAGATCATCGACGGCAAGAAGA
ACATCGAGTCGTACAAGAATGGATTCGTTAACCTCGCACTGCCA

> Uba1 (FG848)_*Schizophyllum commune*
ACCAACCACCACATCGACTTCATCACGGCGGCGTCCAACCTTCGTGCCACCAACTACA
GCATCCAGCCCGCTGATCGCCATACCACGAAGCAAATTGCGGGCAAGATTATCCCTGCC
ATCGCGACCACGACATCGCTCGTGACGGGCTTGGTCTGCTTGGAGCTGTACAAGGTGC
GTGTCGTGTGACTCGGCATATGTTGCTGAACGGCTTGTAGATCATTGATGGCAAGCGGA
AGCTCGAGGCGTACAAGAACGGTTTCGTCAACCTCGCCTTGCCC

> Uba1 (FG848)_*Agaricus bisporus* var. *burnettii*
ACTAATCATCATATCGACTTCATCACTGCTGCCTCTAATCTGCGTGCCATGAACTATGGC
ATCAACCCAGCGGATCGCCATAACACCAAACAAATTGCTGGTAAGATTATTCCTGCAAT
CGCAACCACTACCTCCCTTGTTACCGGCCTCGTTTGTCTTGAGCTTTGTCTTGAGTTTT
CAAGATTATCGATGGCAAAACGAAGTTAGATGATTATAAAAATGGCTTTGTTAACCTTG
CATTACCT

> Uba1 (FG848)_*Laccaria bicolor*
AGCAACCATCACATTGACTTTATCACTGCTGCATCCAATCTTCGTGCCATGAACTATGGT
ATCAACATCGCGGACCGTCATACAACGAAACAGATTGCTGGAAAAATTATACCTGCCAT
TGCTACTACCACGTCTCTCGTGACAGGTTTGGTGTGCCTCGAGTTGTACAAGGTTTGTC
CCATTAGCGTATTTTATTTGTCCTGATCTTGCAACAGATAATTGACGGCAAGAAAAAGC
TGGAGGATTATAAGAACGGATTCGTCAATCTTGCTTTGCCT

> Uba1 (FG848)_*Pluteus cervinus*
ACCAATCATCACATCGACTTCATCACTGCCGCCTCCAACCTGCGCGCGTCGAACTATGG
AATCACCATCGCGGATCGTCATACAACGAAGCAAATCGCTGGGAAAATCATCCCTGCA
ATCGCCACCACCACATCCCTCGTAACAGGACTAGTGTGCCTGGAGCTATACAAGGTAA
GTTGGAAACTGAATTCTGATTACTGTTGACTTTGTTGTAGATCATCGATGGCAAGAAGA
AACTAGAGGACTACAAGAACGGATTCGTCAACCTTGCCTTACCC

> Uba1 (FG848)_*Fomitopsis pinicola*
ACGAACCACCATATCGATTTTATCACTGCTGCTTCTAACTTGCGTGCGATGAATTACAAC
ATCAAGCCCGCTGACCGCCACACAACGAAGCAGATCGCCGGCAAGATCATTCCCGCCA
TTGCCACCACCACGTCCCTCATCACGGGTCTGGTGTGCTTGGAACTCTACAAGGTCGG
CGACATCGCGGCTTGCGTCACGTGACTGCCTTCTAGCTCATCGACCATAAGTCGGAGC
GCGAAGATTACAAGAACGGCTTCGTCAACCTGGCTCTGCCC

> Uba1 (FG848)_*Mycena crocata*
ACTAACTACCACATTGATTTCATTACCGCCGCGTCCAACCTTCGAGCGACGAACTACAA
TATTACCCCTGCTGATCGACACACCACTAAGCAGATCGCCGGCAAGATTATTCCTGCTAT
TGCGACGACCACATCACTTGTGACCGGCTTAGTTTGCCTTGAGCTGTACAAGGTAGGAT
ATATTCCTTGTATGGCACTTTTTCTAACGATAATCAGATCATTGATGGCAAGGCCAAGCT
TGAGGAGTATAAGAATGGATTCGTCAACCTTGCCCTGCCA

> Uba1 (FG848)_*Gymnopus androsaceus*
ACCAACCACCATATCGACTTTATCACTGCCGCCTCCAATCTCCGTGCTACGAACTACAG
CATCACTCCTGCTGACCGACACACGACCAAGCAGATTGCCGGAAAGATCATTCCTGCC
ATTGCTACAACCACATCGTTGGTGACTGGTTTGGCTTGCCTGGAGCTCTACAAAGTGTG
TAGTTTTCTTCATTCTGCTTGTGCCCTAACGTCTACCAGATCATCGACGGCAAGAGAAA
GCTTGAAGACTACAAGAACGGATTCGTGAATATCGCGTTGCCA
> Uba1 (FG848)_*Serpula lacrymans* var. *lacrymans*
ACCAATCACCATATCGACTTCATCACTGCAGCTTCCAACCTTCGCGCCATGAATTATAAT
ATTCCAATTGCGGACCGACACACGACTAAGCAAATTGCAGGCAAGATCATTCCAGCCA

TTGCCACTACAACAGCTCTCGTTACTGGCCTGGTTTGCTTGGAACTTTACAAAGTAGGT
TATGTATTTTTTTTTGGTAAAAACTTCGTTTTCAACAGATCATAGATGGCAAGAATAAAC
TAGAGGACTATAAGAATGGTTTCGTAAACTTAGCCTTACCG

> Uba1 (FG848)_*Ramaria acris*
AGCAATTTCCATATCGATTTTATCACTGCTGCCTCCAATCTTCGCGCGATGAACTATAATA
TTCCAATCGCCGACCGGCACACCACCAAGCAAATTGCAGGAAAGATCATACCCGCTAT
CGCCACTACCACGGCCCTCGTCACAGGTTTAGTCTGCTTAGAGCTGTACAAGGTTTTCT
CTTTTGTTTGTTCAGCCTGTTCTTTCACCAGCTATAGGTCATAGACAACAAGCAAAAGT
TAGAAGACTACAAGAATGGTTTCATCAACCTCGCCCTTCCA

> Uba1 (FG848)_*Tricholoma matsutake*
ACCAATCATCATATTGACTTTATCACTGCTGCTTCTAATCTTCGGGCCATGAACTACAGC
ATTAATATCGCAGATCGTCATACTACAAAGCAGATAGCTGGGAAGATTATACCCGCTATT
GCTACAACTACCTCACTCGTGACTGGCCTGGTATGCTTAGAGCTTTATAAGGTATGAAC
TCATGCGTACGGTGGCATGGTGATTAGATTATCGACGGCAAACAGAAGCTCGAGGACT
ACAAGAATGGATTCGTAAATCTAGCTCTTCCA

> Uba1 (FG848)_*Amanita muscaria*
ACCAACCATCACATTGACTTTATTACGGCAGCTTCGAATCTGCGTGCTATGAATTACAG
CATCACTATTGCGGATCGCCACACGACGAAGCAGATTGCAGGGAAAATCATTCCTGCC
ATTGCAACAACTACTTCCCTTGTGGTGGGTTTGGTTTGCCTAGAATTATACAAGGTATG
ACGTGCAAGTATCAATACCCAAAATTCACTTTTCCCCAGGTTGTGGATGGCAAGAAGA
AGGTTGACAGTTATAAAAATGGATTCATCAACTTAGCTTTACCG

> Uba1 (FG848)_*Hygrophoropsis aurantiaca*
ACGAACCACCACATCGACTTCATCACTGCCGCTTCAAACCTTCGCGCAATGAACTACA
CTATCCCCATTGCTGATCGCCACGCGACCAAGCAGATTGCGGGAAAGATTATACCAGCA
ATTGCGACGACCACAGCTCTTGTTGTTGGTTTGGTTAACTTAGAACTTTACAAGGTATT
ATAATCGATTCCTTGAACCATCAATGCCCCTGTGCTTAGATCATCGACGGGAAAAACAC
AATTGAAGAATACAAGAATGGATTCATCAATCTTGCTCTTCCG

> Uba1 (FG848)_*Gymnopilus chrysopellus*
ACGAACTTCCATATTGACTTCATCACTGCGGCGAGCAATCTTCGCGCCATGAATTACTC
CATTAACCCAGCAGACAGACACACCACGAAGCAAATCGCTGGCAAAATCATTCCCGCT
ATCGCTACTACGACCTCACTCGTCACTGGGTTGGTTTGCTTGGAGCTCTACAAGGTACC
TGAATTTAGAAACTTTCCTTCTAACTAAACTATCCACAGGTCATCGATGGAAAGAAGAA
GCTCGAAGATTACAAGAACGGATTTGTGAATCTGGCTCTGCCT

> Uba1 (FG848)_*Marasmius fiardii*
ACGAATCATCACATAGACTTCATCACCGCTGCTTCGAACCTTCGGGCAACTAATTATAA
CATCACACTAGCCGACCGTCATACAACTAAACAGATTGCAGGGAAGATTATTCCTGCAA
TTGCAACCACAACCTCCTTGGTGACCGGTCTGGTCTGTCTAGAGCTTTTGAAGGCACG
TTGCGCTGGCTCGTATAACGTCAATTCATCTGTTTATAGATCATAGACGGCAAAGACAA

GTTGGAGGAGTACAAGAACGGTTTCGTCAACTTGGCGCTCCCG

> Uba1 (FG848)_*Boletus edulis*
GCAAACCATCACATAGACTTTATCACTGCTGCTTCTAATCTACGCGCAATGAACTACAA
CATTCCCATCGCCGATCGCCACGCCACAAAACAGATTGCCGGAAAGATCATTCCCGCTA
TTGCTACGACTACTGCCCTTGTTGTTGGCTTGGTGTGCTTAGAGCTGTATAAAGTGCGT
GCTTCTCATGTCATGATGGATCATTCACCGGGAATTAGATTATTGATGGGAAGAACAAA
CTCGAGGAATACAAGAATGGATTCGTGAACCTCGCTCTTCCT

> Uba1 (FG848)_*Gautieria morchelliformis*
ACTAATTTTCACATTGACTTCATCACCGCTGCATCGAACCTTCGCGCCATGAACTACAA
CATTGCTATTGCCGATCGACACACCACCAAGCAAATTGCAGGCAAGATTATACCCGCGA
TCGCCACTACTACAGCGCTTGTGACAGGTCTGGTTTGCTTAGAGCTTTATAAGGTTCCT
TCAGCCCTTCCCTTCTTTTTTCGTTTAAAGCATAACAGGTAATAGACAATAAGCGAAAG
CTCGAGGATTACAAGAACGGGTTTGTGAATCTTGCACTTCCT

> Uba1 (FG848)_*Calocera cornea*
ACAAACTTCCACATCGACTTCATCACGGCTGCGTCAAACCTTCGGGCTACCAACTATG
GCATTACCGTCGCTGACCGACACCACACCAAGCAGATCGCTGGCAAGATCATTCCGGC
CATTGCTACCACAACGGCTGTGGTGTCGGGACTCGTTTGTCTGGAGCTGTACAAGTGA
TCGATGGCAAGAAGAAGCTGGAGGCATACAAGAATGGCTTCGTTAACCTGGCCCTTCC
T

> Uba1 (FG848)_*Dacryopinax primogenitus*
ACCAACTTCCACATCGATTTCATCACTGCTGCATCTAATCTCCGTGCAACGAACTATGG
CATTGCCGTCGCTACCCGTCATCACACGAAGCAGATTGCTGGCAAGATCATTCCTGCTA
TTGCTACCACAACTGCTGTGGTGTCAGGGCTCGTGTGCTTGGAGCTTTACAAGTGATT
GATGGGAAGAAGAGGCTCGAGGCTTACAAGAATGGATTTGTCAACCTCGCCCTCCCG

> Uba1 (FG848)_*Rhizopus microsporus*
AGCAACCATCATATTGACTTCATCACTGCCGCTTCTAATTTGCGTGCAATGAACTATGGT
ATTACCATTGCCGACCGTCACCGAACCAAATTCATTGCAGGCAAGATCATTCCTGCTAT
TGCTACTACCACAGCCTTGGTTACTGGTTTAGTTTGTCTTGAATTGTACAAAATCATTGA
TGGTAAGAAGGACTTGGAACAGTACAAGAATGGTTTTGTCAACCTCGCTTTACCA

> Uba1 (FG848)_*Asperigillus triticus*
ACCAACCACCACATCGACTTCATCACGGCCGCCAGCAATCTCCGTGCCGACAACTACG
ACATTCCCCAGGCCGATCGCCACCGGACCAAGTTCATCGCCGGCAAGATCATCCCCGC
TATCGCCACGGCCACTGCCTTGGTGACCGGGTTGGTTGCATTGGAGTTGTACAAGGTC
ATCGATGGCAAGGATGACATTGAACAGTACAAGAACGGGTTCGTCAACCTCGCCCTCC
CC
> Uba1 (FG848)_*Neurospora crassa*
ACCAACTATCACATCGACTTCATCACGGCCGCCAGCAACCTGCGCGCCGAGAACTACA
AGATTGAGCCTGCCGACCGTCACAAGACCAAGTTCATCGCCGGCAAGATCATTCCCGC

CATCGCCACCACCACGGCCCTGGTTACCGGCCTCGTCGTCCTCGAGCTGTACAAGATTA
TCGACGGCAAGACCGACATTGAGCAGTACAAGAACGGCTTCATCAACCTGGCCCTGCC
C

> Uba1 (FG848)_*Agaricostilbum hyphaenes*
ACGAATCACCACATCGACTTCATCACGGCTGCTTCGAACCTGAGAGCGCACAACTACG
CTATTGCTCCTGCCAACCGCCATCAGACCAAGCAGATTGCAGGCAAGATCATCCCTGCC
ATTGCAACAACTACTTCACTGGCCACTGGACTGGTGTGCCTCGAGCTCTACAAGGTAT
GCGATTCTGCGAGGTGCCATTGACGCTCACGGTCTCGCAGATCCTAGATGGCAAGACG
GACATTGAAGCCTACAAGAACGGCTTTGTGAACCTTGCTTTGCCC

>Uba3 (FG844)_*Boletus edulis*
TCCGTACTCGAGTGGCCTAGAGTCCATGGAGGTTAGCTTCTCTGACATCGTGCATTTCT
CTGCAGATAAGAAGCTTGACACTGATGACCCCGATCACATCAATTGGTTGTACACTGTT
GCATCTGCACGCGCCAAGGTGTTCAAGATTGAGGGGATGACATGGTCACTTACTCAAG
GTGTTGTCAAGAACGTGATACCTGCCATAGCATCGACAAACGCCATCATTGC

> Uba3 (FG844)_*Hygrophoropsis aurantiaca*
TCCGTCCTAGAATGGCCTCGTGTACACGGAGGTAAGATTTTTTTCTGGCCCATATCCAC
GCTAGACAAGAAATTGGATACAGATGATCCCGAACATATAGGGTGGCTTTTGAGCGTTG
CCTCCGCGCGAGCCAAAGAGTTCAAGATTGAAGGCGTGACTTGGTCTCTTACCCAGGG
TGTTGTCAAGAATGTCATCCCTGCTATCGCTTCTACAAACGCCATTGTCGC

> Uba3 (FG844)_*Gymnopilus chrysopellus*
TCAGTGCTTGAGTGGCCTCGTGTTCATGGAGGTATCGTTATTTTTTCCTTCTTGTGATTG
GGTCGCAGACAAGAAGTTGGACACAGATGACCCTGACCATATTGGCTGGCTATACCGG
GTTGCGGCTGCAAGAGCAGCAGAATTCAAGATTGAAGGTGTAACTTGGTCTCTGACAC
AGGGTGTGGTAAAGAATATCATTCCGGCGATCGCTTCCACCAATGCAATCATAGC

> Uba3 (FG844)_*Mycena crocata*
TCAGTTTTAGAATGGCCCCGTGTGCAAGGAGGTAAGGCTCGGACACGTCTTACTTAGT
TCATAGACAAGAAAATGGACACCGATGACCCAGATCACATTGGCTGGCTTTACAGTATT
GCATTGGCCCGAGCAAAGGAATTCAAAATTGAAGGTGTTACGTGGTCACTTACACAAG
GGGTCGTGAAGAACATCATTCCGGCAATCGCATCCACGAACGCCATTATTGC

> Uba3 (FG844)_*Tricholoma matsutake*
TCTGTCCTGGAATGGCCCCGCATACACGGCGGTCGGTACCCCTTGTCGCGGTGGAATCT
GATAGACAAGAAAATGGACACAGATGATCCAGAACATATCGTATGGCTTTACACTGTTG
CAGCAGCTCGGGCGAAGGAGTTCAATATCGAGGGTGTGACATGGTCACTGACGCAAG
GTGTCGTGAAGAACATCATTCCCGCCATTGCTTCCACAAATGCCATCATCGC

> Uba3 (FG844)_*Pluteus cervinus*
TCTGTCCTTGAATGGCCCAAGGTACAAGGAGGTCAGCGGGCTAACTTCATATGAAACA
GATAAAAAGCTTGACACCGACGATCCTGAACACATTGGTTGGCTTTATCAAGTTGCTTC

TGCCCGCGCCAAGGAGTTCAAGATCGAGGGTGTGACCTGGTCTTTGACCCAAGGGGT
TGTGAAGAATATCATCCCTGCTATCGCTTCGACAAATGCTATCATCGC

> Uba3 (FG844)_*Gymnopus androsaceus*
TCGGTGCTAGAATGGCCTCGAGTTCATGGAGGTACGAAGACGTTCTACGTTCCTTCTCA
CTGTAGACAAGAAAATGGACACTGATAATCCGGATGACATTAGTTGGCTCTACTCTGTC
GCGGCAGCACGAGCCAAGGAGTTCAAAATCGAGGGTGTTACCTGGTCGTTGACTCAG
GGTGTTGTTAAGAACATTATTCCGGCTATTGCTTCCACAAACGCTATCATTGC

> Uba3 (FG844)_*Marasmius fiardii*
TCCGTTCTAGAATGGCCGCGCGTTCACGGAGGTGAGAGAAAGACTTCTCGATTTCGGT
TGACAGACAAGAAGATGGATACAGACGATCCGGAACATATCGGCTGGTTGTATAATACT
GCTGCCGCGCGCGCGAAGGAGTTCAAGATAGAAGGAGTTACTTGGTCTCTGACGCAG
GGTGTGGTGAAGAACATTATACCAGCTATCGCCTCAACAAATGCGATAATAGC

> Uba3 (FG844)_*Agrocybe pediades*
TCTGTTTTAGAGTGGCCAAGGGTATACGGAGGTCAGTCATATTCGCTTACTTTTGTAGAT
GCCAGACAAGAAGATGGACACGGATGACCCAGAACACATTGGGTGGCTCTACAAAGT
TGCTGCAGCTCGCGCGGCTGAGTTCAAGATCGAAGGCGTCACTTGGTCCTTGACTCAG
GGAGTCGTAAAGAACATTATTCCTGCCATTGCATCTACGAACGCTATAATTGC

> Uba3 (FG844)_*Fomitopsis pinicola*
TCAGTTCTAGAGTGGCCAAAAGTCTTCAAAGGCGGGCTCCATCAGCCCAACGATTGTT
CAGGCTATAGATAAGAAGCTGGATACGGATGACCCAGAACACATCGGATGGCTATTCAA
GACTGCATCGGCGCGAGCGAAGGAATTCAAGATTGAGGGCGTCACATGGTCCCTCACG
CAAGGTGTTGTCAAGAACATCATTCCTGCCATTGCATCCACGAACGCCATCATCGC

> Uba3 (FG844)_*Calocera cornea*
AGTGTGCTGGAGTGGCCGCGTGTCTTCCCTGGTGTGTATCGCTGACTCTATGCCTCGTA
TTATGACAGACAAGAAGATGGACACAGACGACCCGGATCACATCCAGTGGCTCTACGA
GCGCGCGTCTGCCCGCGCACAAGAGTTCAAGATCGAAGGCGTGACGTGGTCGCTCAC
GCAAGGCGTCGTCAAGAATATCATCCCTGCGATCGCGAGTACGAACGCTATCATCGC

> Uba3 (FG844)_*Dacryopinax primogenitus*
AGCGTGCTAGAGTGGCCCAAAGTCTTCCCAGGTGCGTTTCCCCCTGAGTTTACACAGA
TAAGAAACTCGACACAGACGACCCGGAGCACATCCAGTGGCTCTTCACGCACGCCTCT
ACCCGCGCGCGCGAATTCAAGATCGAAGGCGTGACCTGGTCTCTCACCCAAGGCGTC
GTCAAGAACATCATCCCCGCGATCGCGAGTACGAACGCGGTGATCGC

> Uba3 (FG844)_*Neurospora crassa*
CACGTCATCGCTTGGGACAAGGAGAAGCGTTCCCGCAGCTCGACAAGGACGACCCGG
AGCACATCACCTGGCTCTACCAGAAAGCCCTCGAGCGGGCAAAGGAATTCAACATATC
GGGCGTCACGTACTCTCTGACGCAAGGCGTGGTGAAGAACATCATCCCGGCCATCGCG
GCCACGAACTCGGTCATCGC

> Uba3 (FG844)_*Coprinopsis cinerea*
TCCGTACTCGAGTGGCCCAAAGTGCATGGAGATAAGAAGATGGATACTGACGATCCAG
AGCATATCAGTTGGCTTTACAGCGTGGCTCTTAAGCGTGCCCAAGAATTCAATATCGAA
GGCGTCACCTGGTCGCTGACACAAGGCGTCGTGAAGAACATCATCCCAGCTATCGCAT
CCACCAACGCCATCATAGC

> Uba3 (FG844)_*Gautieria morchelliformis*
TCAGTACTCGAATGGCCAAGGGTTCACAAAGGTCAGTATTTCAATGGGCTCGGTTTCTC
TTCTGTAAGACGAGAAAATGGACACTGATAATCCAGAACATATTGGATGGCTATATAGT
GTGGCAGCCGCGCGTGCAAAAGAGTTTAACATTGAAGGTGTCACATGGACTCTCACCC
AAGGTGTTGTGAAGAACATTATCCCCGCCATCGCATCGACCAATGCCATTATCGC

> Uba3 (FG844)_*Amanita muscaria*
TCTGTGCTCGAATGGCCCCGTGTTCACGGAGGTATGGATGGTTTAACGCCCAAGATTTC
TCACTACAGAGAAGAAGATGGACACGGATGATCCAGAACATATAACCTGGCTTTACCA
GACAGCTTCTAAGCGCGCAAAGGAGTTCAATATTGAAGGAGTGACTTGGTCGCTGACA
CAAGGTGTTGTGAAAAACATTATTCCTGCCATTGCCTCCACCAATGCCATCATTGC

> Uba3 (FG844)_*Schizophyllum commune*
TCCGTACTCGAGTGGCCTCGCGTGCATGGGGGTGAGTGCGCCTGTTCAGGCTCGGTCG
GCTTGACAGACAAGAAGCTCGACACGGACGATCCTGAGCACATTGGCTGGCTGTACTC
CGTCGCTGCAGCGCGCGCGAAGGAGTTCAAGATCGAGGGTGTCACTTGGTCGCTCAC
ACAGGGTGTCGTGAAGAACATCATCCCTGCCATCGCATCGACGAACGCTATCATCGC

> Uba3 (FG844)_*Trametes versicolor*
TCCGTACTAGAGTGGCCACGGGTACATGGAGGTAGGTTCGAACCCATATTGTAAGATGG
TATATAGACAAGAAGATGGACACCGACAACCCAGAACATATCCAATGGCTGTACCAGAT
CGCGCTTCACCGCGCCCAAGAATTCAAGATTGAGGGTGTGACATATTCATTGACGCAA
GGCGTCGTGAAGAACATCATTCCTGCTATCGCCTCTACCAACGCAATCATCGC

> Uba3 (FG844)_*Phanerochaete carnosa*
TCGGTCTTAGAATGGCCGCGCGTCCATGGAGGTAATGTATACTTTCATGTCGGGAACGC
ATCTCCAGACAAGAAGATGGACACGGACGATCCTGAACATATCGGATGGCTGTACAAG
ACGGCTCTCGCTCGTGGCAAAGAGTTCAACATCGAGGGTGTAACATACTCGCTCACAC
AAGGTGTCGTAAAGAACATTATTCCTGCCATCGCCTCAACCAATGCCATTATTGC

> Uba3 (FG844)_*Stereum hirsutum*
TCAGTGCTCGAGTGGCCCAAGGTCCAAGGCGGTGAGGACGCCACTCATTTCTACTCCC
ACATCTCCAGACAAGAAACTGGACACCGATGACCCTGATCATATCACCTGGCTGTATAC
CGTCGCTGCCGCACGAGCCAAGGAGTTCAAGATCGAAGGTGTGACTTGGTCGCTGAC
GCAGGGTGTGGTGAAGAACATCATCCCGGCCATCGCATCGACAAACGCCATCATCGC

> Uba3 (FG844)_*Lactarius quietus*

TCCGTGCTAGAGTGGCCAAGGGTGAAAGGAGGTACGTGTAACGCTATCACAGTGCGA
CAGTCCAATAGACACAAAACTCGATACGGATAACCCTGAACACATCCAGTGGTTGTAC
GAGGTCGCTCTCAAGAGGGCCAAAGACTTCAAAATCGAAGGCATCTCGTGGTCGTTAA
CGCAAGGCGTTGTCAAGAACATTATCCCCGCGATCGCCTCTACCAATGCCATCATCGC

> Uba3 (FG844)_*Fomitiporia mediterranea*
TCCGTATTAGAATGGCCTCGCGTATTCGGCGGTTCGTTTTGCCTTCTCGAAGCTTTAAA
AATGTTTAGAGAAAAAACTGGACACTGACGATCCGGAACATATCAACTGGTTATACAC
CACCGCCCTCGCTCGCGCAACTCAATTCAACATCGAAGGTGTAACCTGGTCCCTAACG
CAGGGAGTCGTAAAAAACATCATCCCAGCCATCGCTTCAACAAACGCCATCATCGC

> Uba3 (FG844)_*Laccaria bicolor*
TCAGTTCTGGAATGGCCCCGGGTCCATGGAGGTTTGTTCCCGCACAAACACAATTTTTT
CACTTACAGATAAGAAGATGGACACGGATGATCCGGAGCACATTGGATGGCTTTACAA
GGTAGCGGCAGCTCGGGCACAGGATTTCAAGATTGAGGGCGTTACGTGGTCACTCACA
CAAGGAGTGGTGAAGAACATCATTCCGGCCATAGCCTCAACAAACGCTATCATTGC

> Uba3 (FG844)_*Serpula lacrymans* var. *lacrymans*
TCTGTATTAGAATGGCCCCGTGTTCATGCAGGTGAATAAATATTTTTCCCATTACCTTGT
CCCTTCAGATAAGAAATTAGATACCGATGACCCAGAGCATATATCATGGCTTTATACTAT
TGCTGCGGCACGCGCAAAAGAATTCAAGATTGAAGGTGTTACATGGTCTCTTACGCAA
GGAGTTGTCAAGAATGTCATTCCTGCTATTGCGTCAACCAATGCTATTATAGC

> Uba3 (FG844)_*Agaricus bisporus* var. *burnettii*
TCCGTTCTAGAGTGGCCCCGCGTACAAGGAGGTATAGTTGAACGTTGCGGTTTTCTTAA
TGCTTACAGATAAGAAAATGGATACAGACGACCCGGAACATATTTCTTGGCTATACAAA
ATAGCGGCAGCAAGGGCTCAAGAATTTAATATCGAAGGCGTGACATGGTCTCTTACTCA
AGGCGTCGTCAAAAACATTATACCATCCATTGCATCCACCAATGCTATCATTGC

> Uba3 (FG844)_*Auricularia delicata*
TCCGTGCTTGAGTGGCCGCGCCAACGAGGCGGTAAGCATCCTGTCGCGTAGCTCTCTC
GTGACCTCAGAGGAAAAACTCGACACCGACAACCCGGACCACATTACGTGGCTGTATA
ACCTCGCTGCTGCGCGCGCGAAGGAGTTCAAGATTGAAGGCGTGACATGGACGCTGA
CACAAGGCGTCGTCAAGAACATCATCCCCGCCATTGCAAGCACGAATGCAATCATTGC

> Uba3 (FG844)_*Agaricostilbum hyphaenes*
TCTGTACTGGAATGGCCCCGCGTAGGACAAGAAGATGGACACTGATGATCCAGATGAT
ATTCAATGGCTCTTTGATCAGGCTTCGGCGCGAGCTCATGAGTTTGGCATCGAGGGTGT
GACTTGGTCTTTGACGCAGGGCGTTGTCAAGAACATTATTCCAGCCATTGCTTCGACCA
ACGCCGTCATCGC

> Uba3 (FG844)_*Rhizopus microsporus*
TCCGTCTTGGAATGGCCCCGTGTGTGGGGATCTAAAAAGTATGATACAGACAACCCAG
AGGACATTAATTGGCTATACCAGCAAGCTCTAACAAGAGCAAAACAGTTCAATATAACT

GGTGTCACTTATTCCCTCACTCAGGGTGTAGTCAAAAACATCATTCCCGCAATCGCATC
CACCAATGCAATTATTGC

> Uba3 (FG844)_*Asperigillus triticus*
CACCAGATCGCCTGGCAGGAGCAGCGCAGGATGACCCCTTCGACAGCGACGACATGG
AGCACATCGGCTGGGTCTACCACACCGCCCTCGAGAGAGCAAAGCAATTCAACATCTC
CGGCGTCACCTTCCAGATGGCCCAGGGTGTCGTGAAAAACATCATCCCCGCCATCGCC
TCAACCAACGCTGTCATCGC

>Ygr207c (FG757)_*Agrocybe pediades*
ATCGACGACGACTTGGGCGTAACTGGTCAGATGCTTGCCGGTCTCCTTGGCTGGAGTC
AAGCTACCTTCGCTAGCAAGGTCGAGGTCGATTTGGCCAAGAAAGAGGCGTTTGTTGT
CAGGGAAATTGACGGCGGAGGAGAGGAGATTCAGTGCAAGCTTCCTTTGGTTGTGAC
CACTGACCTTCGGTGAGATTCCTCAATAGTATTCCATATTTTACAGATTGAACGAACCTC
GTTACGCCTCGCTACCCAA

> Ygr207c (FG757)_*Laccaria bicolor*
ATCGACGACGATCTAGGCGTTACAGGCCAGATGCTCGCGGGTTTGCTGGGCTGGAGTC
AAGCCACATTTGCATCCAAAATCGAAGTGGACGTAGAGAAGAAGCAGGCGTTGGTTA
CACGTGAAATCGACGGTGGTTCAGAGGAACTCAGGTGCTCGTTGCCCATTGTGATTAC
CACCGACCTGAGGTACGTTTCTCCCTCAATACATGAAGTAGTGGTGGCATAGACTCAAC
GAACCGCGGTACGCGTCTTTGCCTAA

> Ygr207c (FG757)_*Coprinopsis cinerea*
ATCGACGACGACTACGGCGTGACGGGCCAAATGCTCGCAGGGCTGATGGGCTGGTCG
CAAGCGACGTTTGCGAGCAAAGTCGAGTTGGACGCTGCGAAGAAGGAGGTGAAAGTT
ACTCGGGAGATTGATGGTGGTGGAGAGGAGGTGTTGTGTAAATTGCCGGTGGTTATTA
CTACTGATTTGAGGTGCGTTGTTTTTTTTTCGTTTCATTTCTCCCTCCCCCTCCCTCCCAA
TCCCACACCCACACCCAATCACTCACTCACTCACACCCACTCATCAACAGGCTCAACG
AACCCCGCTACGCCTCCCTCCCCAA

> Ygr207c (FG757)_*Fomitopsis pinicola*
ATCGACGACGACGCGGGCCAGACGGGCCAGATGCTCGCGGGCCTCATGGACTGGGCG
CAGGCGACGTTCGCGAGCAAGGTCGAGCTGGATGCGGACAAGAATGAGGCGAGCGTG
ACTCGGGAGATTGATGGCGGGCTGGAGGAGCTGCGGTGTCGGTTGCCGATGGTGGTC
ACGACGGATCTTCGGTGAGTGCGCGTGTTAGGACAGCACGAGCTGACCATGGCTTCTG
CAGGCTGAACGGTATGTTGGGTAACGTTTCGAGGACGCTTTAATAACCTTGCGCGCAG
AACCACGCTACGCCTCATTGCCTAA

> Ygr207c (FG757)_*Trametes versicolor*
ATCGACGACGACGCGGGGCAGACGGGCCAGATGCTCGCGGGCCTCATGGACTGGGCG
CAGGCGACGTTCGCGAGCAAGCTCGTGGTGGACCCGAAGGCGAAGGAGGCGTCGGT
CACGCGGGAGATTGACGGTGGGCTGGAGGAGCTGAAGTGCCGTCTACCATTGGTTGTC
ACGACCGACCTACGGTGAGTCCCATCCGGTCATGCGATATACCTGAGTGCGCATTTTTG

CAGGTTAAACGGTACGTCCTATGAGCAAGTTGCCCCGGAGGCATGACATTCTTCGCAG
AGCCCCGGTATGCTTCATTGCCGAA

> Ygr207c (FG757)_*Schizophyllum commune*
ATCGACGATGACGCTGGACAGACGGGTCAGATGCTTGCGGGGCTGCTGGACTGGCCG
CAGGCGACGTTCATCAGCAAGCTGGACGTCGACCCGGCGAAGAAGGAGGCGTTGGTG
ACCCGGGAGATCGACGGCGGCGGGGAGGAGTTGAAGTGCAGGCTGCCGCTTGTAGTG
ACGACAGACTTGCGGTGCGTGTGATGCTCAGGATTGGGGTTTTTGACTATTCAGGCTG
AACGAGCCGCGGTATGCTTCATTGCCGAA

> Ygr207c (FG757)_*Mycena crocata*
ATCGACGACGACGCCGGCCAGACGGGTCAGATGCTCGCGGGGCTGATGGGATGGGGA
CAGGCGACGTTCGCCAGCAAGGTCGACGTCGATGTGGGTGCGAACTCTGCGACGGTG
ATGCGTGAGATTGACGGCGGGTCAGAGGAAATTCGGTGTCGGCTGCCGTTAGTCGTCA
CCACAGATCTGCGGTGCGTTGCTTCATCGTTCCCTAGCCAATCCTTAAACATATCTACAG
ATTAAATGGTATGCCTCCCCACAAAATGACCTCTCCGCTATGACCGGTTCTCCCAGAGC
CGCGCTACGCCTCCCTCCCCAA

> Ygr207c (FG757)_*Hygrophoropsis aurantiaca*
ATCGACGATGATGCGGGACAAACAGGACAAATGTTGGCTGGGCTCATGGGCTGGTCAC
AGGCGACGTTCGCAAGCAAGGTCGTTGTTGACGCAGAAAACAAGTCTGCCAATATCA
CGCGGGAGATTGACGGTGGGCTGCAAGAGTTGAAGTGCAGACTACCACTCATTGTCAC
GACTGATCTTCGGTAAGAGCTCCACTATATGCACCACAAGTTATTTACATTTATCTCCAG
ACTCAACGGTGGGTAATTTACTATTTTCGTTGCCCATTTCTTCAAACGTGTTCAGAGCCT
CGCTACGCTTCACTACCGAA

> Ygr207c (FG757)_*Boletus edulis*
ATCGACGACGATGCAGGCCAGACGGGCCAGATGCTCGCTGGGCTCATGGGTTGGGCA
CAGGCGACGTTTGCGAGCAAGGTGGACGTCGATGCGGCACAAAAAGTGGCACATGTC
ACTCGTGAAATCGACGGTGGATTGGAAGAGATTCGGTGTAGGCTTCCTCTTGTGGTCA
CCACGGATCTGAGGTGAGCCTCTTGTAGGGGATGAAGTAGGCTGATCCATTACCTCCTA
GATTGAATGGTACGTGATGTCCTAGCATCAAGCGTGTTCACGCCTCCGTTTCTTCTCAG
AGCCTAGATATGCGTCTTTGCCTAA

> Ygr207c (FG757)_*Phanerochaete carnosa*
ATCGACGATGATGCGGGACAAACTGGTCAGATGCTCGCCGGACTGCTTGACTGGCCTC
AAGCGACCTTCGCTAGCAAACTTGTTGTGGACCCGACGAAGAAGGAAGCGAACATTA
CCCAGGAAATTGATGGAGGGCTGCAGGAGGTGAAGTGCAAATTACCTTTAATTGTGAC
AACAGACCTCCGGTACGTTCATCGCCACATCATTCATTACCCAACTCATATTCTCCGCAG
GTTGAATGGTGCGTTTATGGTCCACGTATCCGTAGAGCACTATTTTTTTTTCTTAGAGCC
GCGCTACGCTTCATTACCGAA
> Ygr207c (FG757)_*Auricularia delicata*
ATCGACGACGACGCGGGCCAGACGGGTCAGATGCTCGCGGGGCTCTTGGGATGGAGC
CAGGCGACGTTTGCGAGCAAGCTCGACGTCGACGAGCCGAACAAGACCGCTGTTGTG

GTGCGCGAAATCGACGGCGGTGGTGAAGAGATCAAATGCAAGCTGCCTCTTGTGTTGA
CCACGGACTTGCGGTGCGTGTCGGTTACTGTGGACCGTTTATTTACTGGAATTTTTGTA
GTCTAAACCGTGAGTGTCACCGCTCAGATGGATAACTGACCCTCTGCAGTGCCGCGTTA
TGCGTCCCTGCCCAA

> Ygr207c (FG757)_*Marasmius fiardii*
ATCGATGATGACTTGGGTCAGACAGGGCAAATGTTGGCTGGTCTACTCGGATGGTCTC
AGGCTACGTACGCGAGTAAAGTGGAGCTGGACATTCCAAAGAAGGAGGCGACGGTGG
TAAGAGAGATCGACGGCGGCTCAGAGGAAGTGAGGTGTAAGCTGCCGCTGGTGGTCA
CTACGGATCTTAGGTTGAATGGTGTGTTTCTGTTGTCTCTCGGTTTTCCCCTATTATTCA
CGCGTTGTAGAGCCCCGATACGCCTCACTTCCCAA

> Ygr207c (FG757)_*Gymnopus androsaceus*
ATCGATGACGATTTGGGACAGACAGGGCAGATGCTTGCTGGGCTGCTGGGTTGGGGTC
AGGCGACTTACGCGTCCAAGGTGGAGCTGGACTTGGAAAAGAAGAATGCGGTTGTAA
CGAGGGAGATTGACGGTGGTTCTGAGCAAGTGCGAGTCAAGTTTCCTATGATCGTAAC
GACAGACCTACGGCTGAACGGTGTGTTGTGGAATAATATTGTTTTCCTTTCTCTACCATA
TTTTTCAGAACCGCGTTATGCTTCTTTGCCCAA

> Ygr207c (FG757)_*Pluteus cervinus*
ATTGACGACGATTTGGGTATGACTGGACAAATGCTTGCTGGACTCTTGGGTTGGAGTC
AAGCGACTTTCGCGAGTAAGGTAGAGATTGACGTGGAGAAGAAGGAAGCGAGGGTTA
TTCGAGAGATAGATGGCGGTTCAGAGGAAATTAAATGTCGGCTACCTGTCATTATAACC
ACTGACCTCCGGTATGTCATTCCTTATCCCCATCATTGACTGACCATCTTTCATCATAGG
CTGAATGGTAAGCTTACCTTCTACGTCATCACGTTTCTTACCTTAGCCGAATGCCAGAG
CCTCGATATGCTACATTACCCAG

> Ygr207c (FG757)_*Tricholoma matsutake*
ATAGATGATGACCTGGGTATGACGGGGCAGATGTTAGCAGGATTGCTCGGCTGGGGCC
AGGCGACGTTTGCTAGCAAGGTGGATGTGGACGTGCAGAAGAAAGAGGCCGTCGTGA
CCAGGGAGATTGATGGAGGCGGCGAGGAGATCCGGTGTCGGTTGCCTTTGGTGGTGA
CGACTGATCTGCGGTGAGCGATGCAACCCTTGTTGACCTAACGGACTGGTTGCGTCAT
AGCCTCAATGGTGATATTTTCCGTTTACGGGGCGTGACTGATTATTACCCTTTACTACAG
AGCCACGCTATGCTTCACTCCCAAA

> Ygr207c (FG757)_*Gymnopilus chrysopellus*
ATTGATGACGATTTGGGAGTGACGGGACAGATGCTAGCTGGTTTGATGGACTGGAGCC
AGGCTACGTTCGCGAGCAAGGTCGAAATTGATACAGACAAGAAAGAGGCCTTCGTGG
TCAGAGAGATTGACGGTGGAGGTGAAGAAATCAAGTGCCGGCTTCCTCTTGTAATCAC
TACGGACCTGAGGTGGGTAGATCATAATATTCTCCCCTGCTGAACCGTCTTTTTCACAG
ATTAAATGGTTAGCGTCTTGACTATTTCCGCTTCAAATCTCACGAGAATTATCCAGAGCC
CCGCTATGCCTCGCTCCCCAA

> Ygr207c (FG757)_*Amanita muscaria*

ATTGATGATGACCTTGGTCTTACGGGTCAGATGCTTGCTGGACTGTTGGAATGGCCGCA
AGCCACTTTTGCAAGCAAATTGGACCTTGATGTGGATAAGAAAGAGGCTGTTATCGTT
CGTGAAATTGATGGTGGAGGTGAAGAGATCAAGTGTAAGCTGCCGTTGGTGGTTACTA
CAGATTTGCGGTGAGCTTCCCCAGCACTTACCCACTTATGGACAACCACTTTCATTAGC
TTGAATGGTAAGACAAAGTTATTATCTAATTTACGCTTGTGAAAATAGAGCCAAGATATG
CATCGCTCCCCAA

> Ygr207c (FG757)_*Agaricus bisporus* var. *burnettii*
ATTGACGATGATCTCGGACTGACGGGACAAATGCTTTCTGGATTGATGGGCTGGAGTC
AGGCGACTTTTGCGAGCAAAGTCGATGTAGACATTGCTAAAAAAGAGGCTGTAGTGAC
TAGGGAGATCGACGGTGGTGGCGAGGAAGTGAGGTGTAAGCTGCCGTTAATTGTGAC
AACAGACTTGAGGTGAGTTGTCTGCTTGAAGGATCAATGGCTGATTACATGGTGCTTTA
GGCTTAATGGTAAGCCCGTTCTCTTTCCTAATTTCGCAGCTAACTTCTTTTCACAGAACC
TCGTTACGCGTCATTGCCTAA

> Ygr207c (FG757)_*Calocera cornea*
ATAGACGACGATGCCGGGCAGACGGGGCAGATGCTTGCTGGGCTGCTGGGCTGCGCG
CAGGCGACGTTTGCGAGCAAGCTCGACGTCGACGTCGGCGCGGGGACGGCGGACGTC
ACCCGGGAGATTGACGGCGGGCTCGAGCAGGTTAGGGTCAAGCTGCCCGCGATTGTTA
CGGCGGATCTGAGGTGAGTGGGGCGCATACATCTCATGCTTGTGCAGGTTGAACGGTA
CGCCCCAGTAGTAAGTGCAGCCGCTAACATTGTCCAGAACCCCGATACGCCTCCCTCC
CCAA

> Ygr207c (FG757)_*Fomitiporia mediterranea*
ATTGATGACGATGCAGGCCAGACTGGCCAAATCCTTGCTGGCTTACTTGATTGGCCTCA
AGCAACGTTTGCGAGCAAGGTCACTGTAGATGTAGACAAGAAGACAGCAAATGTTGT
GAGAGAAATAGATGGCGGGCTACAGGAGGTCAAATGCAACCTACCCCTCGTCGTTACT
ACGGATCTTCGGTGCGCTGGAGCTATGATTAATCTTTTGCTGATAGTTCTCGGGTCCAGA
TTAAACGGTATTGGCTATCGTCTTACGGATTTCACGCTCACTAAGAAATGTTACCAGAA
CCCCGATATGCTTCGCTCCCGAA

> Ygr207c (FG757)_*Serpula lacrymans* var. *lacrymans*
ATAGACGACGATGCAGGACAGACGGGGCAAATGCTCGCTGGACTTATGGGCTGGGCTC
AGGCGACATTTGCGAGTAAGGTAGAGGTGAACACGGCCGACAAGGTGGCCAACGTCA
CGAGAGAAATAGATGGAGGTCTGGAAGAGTTGAAATGTCAGCTACCTCTTGTTGTAAC
CACAGATCTCCGGTGAGTTAGTTTTTCAGCACGTTTCTTTCTGAACGGTGTCTGCATTA
GCCTTAATGGTATGTGACCGGTACTGTTGAGGCTCCCCCCTATATTGACGTTTTCATAGA
ACCTCGGTATGCCTCATTACCAAA

> Ygr207c (FG757)_*Gautieria morchelliformis*
ATCGATGACGATGCTGGACAGACTGGGCAAATGCTCGCTGGTCTGCTGAACTGGGCGC
AAGCTACATATGCGAGCAAGGTAGTCATCTCTGACGACGAAAGAGAAGTGAACGTTAC
CCGGGAAGTGGATGGTGGCGCAGAGGAGCTTCAATGCCGCTTGCCAGCCATTGTTACG
ACTGATTTGAGGTATGTGTTTTGTCGTTTTCTAGGATGTCTGAAATTTACCATTAATAGAT

TAAACGGCATGTCTATGCCTGTGATTGTCTTACAAGCAGCTAAATCATAATGACAGAGC
CTCGATATGCCTCGTTGCCTAA

> Ygr207c (FG757)_*Ramaria acris*
ATCGACGATGATGCGGGGCAAACAGGTCAGATGCTTGCTGGTCTCCTGGATTGGGCGC
AGGCCACATATGCGAGCAAGGTAGTTATTGCTGATGATGAAAAGGAGGTAGGCGTTAC
ACGTGAAGTGGACGGTGGTGCAGAGGAACTGAAGTGCCGATTACCGGTCATCATAACT
ACCGATTTAAGGTTTGTCTTCCGTCTCTTCCTCACCTTGTTAACAGTGCATTTTCAAGAT
TGAACGGTGGGTTTTAAGACAACTCTGAGAGTTATGAATATATTCTTTACTGATAGAAC
CCCGTTACGCGTCCTTGCCTAA

> Ygr207c (FG757)_*Lactarius quietus*
ATCGACGACGACCTTGGACAGACGGGCCAGATGCTTGCGGCGCTGTTGGGTGCGGCG
CAGGCGACGTGTGCGAGCGCACTTGAGGTGGACGTCGAGAAACGTGTTGCGCGCGTG
CGTACGGAGATTGACGGCGGGGCGCAGGAGCTCCTCTGCACGCTGCCCGCAGTCGTTA
CTACGGATCTTCGGTGCGTGTCCCTTCGGCGTTGTCACTAACCGAACTGTAGTCTCAAT
GGTGTGTGCGGAAAGTCCCTGATACTATAACATGGGTAAAGAGCCGCGGTATGCGTCG
CTACCTAA

> Ygr207c (FG757)_*Stereum hirsutum*
ATTGATGATGATTTGGGACAGACGGGGCAGATGTTGGCGGCGCTGTGGGGGGAGGCG
CAGGCGACGTATGCGAGTAAGGTGGAGGTGGATGTGGAGGAGGGGGTGGCGAGGGTT
ACGACGGAGATTGATGGGGGGGCGGAGAAGTTGAGGTGTCGGTTGCCGGTGGTTGTT
ACTACGGATTTGCGGTACGTTTCTTTTACTGTCGATCTCATATTGGTGCTCACTCATGTC
AGGTTAAATGGTATGTCTGGTGGTATTTTTATGCTGGTTACTGACTTCGGTCTGCTCCAG
AGCCCCGCTACGCTTCCCTGCCTAA

> Ygr207c (FG757)_*Dacryopinax primogenitus*
ATTGATGACGATTATGGCCAGACGGGGCAGATGCTGGCCGGGTTGCTAGGGTGGGGGC
AGGCGACGTACGCTAGTGGGGTGGAGCTAGATTTGGACAAGGGTTGGGCGGATGTAA
CGCGGGAGGTGGATGAGGGGAGTGAACTGGTCAGGGTGAGACTACCGGCGGTTATCA
CTACTGATCTGAGGTGCGTGCACTTGCATGTAATGCATGAACTGACCCTTCTCTGTGTA
GGCTGAATCGTACCCCCCCTTCCCTGTTCGTCACAACTGACCCCCGCAGAACCCCGCT
ACGCCTCCCTACCGAA

> Ygr207c (FG757)_*Asperigillus triticus*
ATCGACGGCGACCAGGGCCAGACGGGCCAGATGCTGGCGGGGTTGCTGAACTGGCCG
CAGGCGACGCAGGCCAGCAAGGTCGACATCAAGGATGACAAGGGCACCGTCGAGGT
GACGCACGAGGTGGACGGGGGTGTGGAGACGCTGCGGATGCAGCTGCCGCTGGTGAT
CACGACGGACCTGCGACTGAACCAGCCGCGGTACGCCAGTCTGCCGAA

> Ygr207c (FG757)_*Neurospora crassa*
ATCGACGACGACGCCGGCCAGACGGGCCAAATGCTGGCCGGTCTGCTGGGCTGGGCG
CAGGCCACCCAGGCGAGCAAGATCGAGTTCACCGGCGGCGATTCTGTGCAGGTGACG

AGGGAGGTGGATGGCGGTGTTGAGACGCTCAAGGCCAAGCTGCCCATGGTCATCACG
ACCGACTTGCGCCTCAACGAGCCCCGGTACGCGAGCTTGCCCAA

> Ygr207c (FG757)_*Rhizopus microsporus*
ATTGATGATGACGCCAGTCAAACTGGACAGATGTTGGCTGGGTTACTCAAGTGGCCAC
AGGCCACATTCGCTTCTAAAGTGGATATCAATGACAAGTCACTCAAGGTTGTACGTGA
AATCGATGGTGGTCTTGAGACAGTGTCTACTAAATTACCTGCTGTTATCACAACTGATTT
ACGGTATGTGTTTTTATTGACGTGCAAGAGACTCATTATGCTTTGTATAGTTTGAATGAA
CCCCGCTATGCTTCTCTTCCCAA

> Ygr207c (FG757)_*Agaricostilbum hyphaenes*
ATTGATGACGATGCATCCCAAGTAGGCAGCATGCTCGGCGGTCTGCTCAACTGGCCGC
AAGCCAATTTCGCTAGCAAGGTCGATCTGGATCCTGCAGCCAGCAAGGTCACAGTGGA
CAGAGAGATGGATGGTGGTATCGAGACGCTCGAGATGAGCTTGCCTGCGTTGATGTGA
GCGGGTGTGGAGTAGTTACTGCGTCTACAGCGCATCTGACTTGCGATTGAACGAGCCT
CGATTCGCATCTCTACCAAA

>Yhm2 (FG524)_*Agrocybe pediades*
GCCGAAATCACCCGCGCTAAGACCGCTGCTACTGGTGTCAAACCTCCCTCGACTTGGG
CTGTCTTTGCTGACATCTATCGTCGTGAAGGTCTTGCTGGCGTTAACAAGGGTGTTAAT
GCAGTGGCTGTCCGTCAATGTACTAACTGGGGTTCACGGTAAGCAATCCATTGGTTATT
TTTCCACTAAAAATCTGACACTTGAACTAGTATGGGTTTCGCTCGTCTGGCTGAGGACT
CTATCCGTAAAATCAGGGGCAAGGGCGAGGCCGACAAGCTCGGTGCGATGGATAAAAT
TATTGCGTCCTCGATT

> Yhm2 (FG524)_*Coprinopsis cinerea*
GCTGAAATTACGCGCGCAAAGACCGCTGCCACCGGTGTGAAGCCTCCATCGACATGGG
CTGTGTTCGCCGACATCTACCGGAGAGAGGGTCTGGCTGGCGTCAACAAGGGTGTGA
ACGCTGTGGCCGTCCGACAGTGCACTAACTGGGGTTCCCGGTACGCCCATATCGGCATA
TCAATTAGTTTCGACCTAAATTCCTGTTTTCAGAATGGGTTTCGCTCGCCTGGCCGAGG
ATATGATCCGCAAAGTCAAGGGCAAGACCGAAGGCGAGAGCCTCGGAGCTGTTGACA
AGATTATTGCCTCGACTGTT

> Yhm2 (FG524)_*Gymnopilus chrysopellus*
GTCGAAATTACGCGAGCCAAAACTGCCGCTAGTGGTGCCAAGCCTCAATCGACCTGGG
CAGTCTTTGCCGATATATATCGCAGAGAAGGTCTGGCTGGTATTAACAAGGGAGTCAAT
GCTGTCGCCGTTCGTCAATGTACGAATTGGGGTTCCCGGTATGTTTTTGACTTTGTATCA
ACGTTGGGCCTCCAAGTGACCTATCAAAGTATGGGTTTCGCTCGCTTGGCAGAAGATG
TTATCCGCAAAGTCAGAGGCAAAGGCGAAACCGAGAAGCTCGGAGCTATGGATAAGA
TTCTTGCCTCATCTGTC

> Yhm2 (FG524)_*Serpula lacrymans* var. *lacrymans*
GCCGAAATTACTCGTCATAAACAGGCTGCTACTGGTGCAAAGCCCCTTTCTACATGGGC
TGTGTTCATGGACATCTACCGTCGGGAAGGTATTCGAGGGATTAACAAGGGTGTTAAC

GCTGTCGCTGTGCGTCAGTGCACAAACTGGGGTTCACGGTAAGCGACTCTCAAATAGG
TCAATCGCGGTTTCAATTGATTACCTATGGCTAGTATGGGCTTTGCTCGTCTTGCCGAAA
CGACTATTCGGAATATCCGGGGCAAGGGAGAAAATGATAAGCTTGGTGCTATGGACAA
GATTATTGCATCTTCGATT

> Yhm2 (FG524)_*Trametes sanguinea*
GCGGAGATCACGCGGCACAAACAAGCAGCGTCCGGGATCAAGCCTCCTTCGACGTGG
GCCGTCTTCGCAGATATCTGGCGGCGTGAAGGTATCCGCGGCATCAACAAGGGCGTGA
ACGCGGTCGCTGTCCGGCAGTGCACGAACTGGGGTTCGCGCATGGGCTTTGCGCGTCT
CGCGGAGGACACCATCCGGAAGATGAAGGGCAAGGGTGAGCACGACAAGCTCTCGCC
ATATGAGAAGATCTTGGCGTCCTCGATT

> Yhm2 (FG524)_*Phanerochaete carnosa*
GCTGAAATCACGCGCCACAAGCAGGCCGCTGCCGGCGTTAAGCCGCCATCGACGTGG
GCTGTTTTCGCCGACATTTATCGCCGCGAAGGTATTGCAGGCATTAACAAGGGCGTTAA
TGCTGTCGCAGTGCGTCAATGTACGAACTGGGGCTCACGGTGAGTAGAGCTGGCTGGC
CACGGTGTATGCGGGGTTGACATTAAATATCTAGTATGGGATTCGCTCGCCTGGCTGAG
AGCACAATACGGAAAGTCAAGGGTAAGGGTGAGAACGAGAAACTCACAGCGCCCGA
GAAGATTTTGGCTTCAAGTATT

> Yhm2 (FG524)_*Fomitiporia mediterranea*
GCTGAAATCACACGACACAAGCAGGCCGCTGCGGGCATCAAGCCGCCGTCGACGTGG
GCTGTCTTCGCGGACATCTACCGCAGAGAAGGTATTGCTGGTATTAACAAGGGCGTTAA
TGCGGTGGCAGTCCGCCAGTGCACGAACTGGGGCTCACGGTGAGTTTGATCTGATGCG
GCCGAGAATTACGATTTTGATAGCACACCGTTAGTATGGGCTTTGCCCGTCTTGCAGAG
GCTACCATCCGGAAAATCCGTGGAAAGACTGAGAATGACAAGCTCGGTGCTCTTGACA
AGATCCTGGCTTCGTCGATA

> Yhm2 (FG524)_*Fomitopsis pinicola*
GCGGAGATTACGCGGCACAAGCAGGCTGCGTCGGGTGCTAAGCTTCCCTCGACCTGG
GCTGTCTTTATGGACATTTACCGCCGGGAGGGTATCAAGGGCATCAACAAGGGCGTTA
ACGCGGTCGCCGTCAGGCAAATGACCAACTGGGGCTCTCGGTACGTGCTCGGGCTACA
GTATACGGAATGGGGTAATTGAGAGGGACGTAGAATGGGTTTCGCGCGGTTAGCTGAA
AGCAGTATCAAGAAGCTGAAGGGCAAGGGCGAGAACGACAAGCTTACCCCTCTGGAG
AAGATCGCATCCTCGTCAGTA

> Yhm2 (FG524)_*Calocera cornea*
GCCGAAATCACACGGCATAAGCAAGCCGCCTCGGGGGTCAAGCCGCCCAGCACTTGG
GCGGTGTTCATGGACATCTACAGGCGCGAGGGCATTGCGGGTATCAACAAGGGTGTGA
ACGCCGTCGCAGTGCGGCAATGCACCAACTGGGGCTCGCGGTGCGTCTTTCCATTCCA
ATTAAGAAGTTGTAGCTGATTCTTTGCGCGTACAGGATGGGCTTTGCGCGCCTGGCCGA
GGACTCGATCCGCAAGATGCGCGGGAAGAAGGAGGGCGAGAAACTGGGTGCGATGG
ACAAGATCCTCTCCTCCTCCATC

> Yhm2 (FG524)_*Dacryopinax primogenitus*
GTCGAGATCACACGGCATAAACAGGCAGCGTCGGGTATCAAGCCCCCGAGCACGTTTG
CTGTGTTTATGGATATCTATCGGAGAGAGGGTATTGCTGGCATCAACAAGGGTGTCAAC
GCAGTCGCAGTGCGCCAGTGCACAAACTGGGGTTCTCGGTGCGTTCCTCCACAGCTCC
CACTCCTCTCTGACTAACTGATCTGATGTAGGATGGGTTTCGCCCGCCTGGCGGAAGAC
ACAATCCGAAGCATGCGGGGAAAGAAAGAGGGCGAAAAGCTCGCTCCGATGGACAA
GATCCTCTCTTCCTCTATC

> Yhm2 (FG524)_*Laccaria bicolor*
GCGGAAATTACTCGTGCGAAAACTGCATCGACGGGAATCAAGCCACCTTCAACGTGGG
CCGTCTTTGCGGACATTTATCGTCGGGAGGGTCTCGCCGGTGTTAACAAGGGTGTCAA
CGCAGTCGCCGTTCGACAATGCACAAACTGGGGATCTCGGTAAGAAATGCTGGACACG
TTGGACTCGAATTCAGTCTAAACATTGGGCAGTATGGGTTTCGCGCGTCTTGCAGAGGA
TTCCATTCGCAAAATCAGGGGCAGAGGAGAGCATGAACCGGGTAGCGCAGTCGACAA
AATTCTCTCTTCGTCGATT

> Yhm2 (FG524)_*Amanita muscaria*
GTCGAGATTACGAGACAAAAAACCGTAGCTACGGGTGCCAAACCACCCTCAACATGG
GCTGTTTTCGGTGACATCTACCGTCGTGAAGGTCTTGCTGGAGTGAACAAGGGTGTCA
ACGCTGTAGCAGTAAGGCAATGTACAAACTGGGGTTCTCGGTAAGCTTTGAATTTCGT
GTCTGCTGCTTGCCATGTTGAATTGATTTCCCAAGGATGGGTTTTGCTCGGTTAGCTGA
GGATATGATCCGCAAAGTGAGGAACAAGCGCGAGGATGAGAACCTGGGTGCGATGGA
TAAAATCTTTGCTTCCTCTGTC

> Yhm2 (FG524)_*Stereum hirsutum*
GCCGAGATTACAAGACACAAACAGGCGGCTGCGGGCATCAAGCCACCATCAACATGG
GCTCTCTTTGCTGAGATCTTCCGCAAGGAGGGTCTCCGTGGTGTCAACAAGGGTGTGA
ACGCCGTGGCTGTCCGTCAATGCACTAACTGGGGATCTCGGTATGTATCTGTTCCAACG
GAGTAAGACAGTAGAAACTGACCGTTGCTTGTCAGTATGGGCTTTGCTCGGCTCGCTG
AGTCGTCTATTCGGTCAATTCGTGGCAAGGGCGAGAAGGACTCGTTGAGCGCTGTCGA
CAAGATCATTGCATCTTCAATC

> Yhm2 (FG524)_*Lactarius quietus*
GCTGAAATAACTCGTCATAAACAAGCCGCTGCGGGTGTGAAGCCACCTTCGACGTGGG
CATTATTCTTTGAGATCTTCCGAAAAGAAGGCATTCGTGGTGTTAACAAGGGTGTGAAT
GCTGTTGCGGTGCGCCAATGTACAAACTGGGGCTCTCGGCAAGTGGACTATCTTACGC
TGTCATATTGTGAGGACCACCCTTTGCTCAGAATCGGCCTTGCTCGACTTGCGGAAACG
TCGATTCGTAGAGTGCGTGGCAAGGAGGAGGGTCAATCTCTCGGTGCCCTAGACAAAA
TTCTCGCATCATCGATC

> Yhm2 (FG524)_*Pluteus cervinus*
GTTGAGATTACCCGGCATAAGCAAGCCGCGACAGGAGCAAAACCACAGTCGACATGG
GCTGTCTTCGCAGACATATACCGCCGAGAAGGTCTTGCCGGTGTTAACAAGGGTGTAA

ACGCGGTTGCTGTTCGTCAATGCACGAATTGGGGTTCACGGTGTGTCCCTTTTTAACTT
GACGGCCAGCAAGGTGAAGTTACTCCTTCTAGAATGGGTTTCGCACGTTTAGCGGAGT
CCAGCATCCGAAAGGTTCGAGGAAAGAGCGAAACTGACAGCCTCGGCGCTCTCGACA
AAGTGTTGGCCTCTTCTATT

> Yhm2 (FG524)_*Schizophyllum commune*
GCTGAGATCACTCGCGCGAAGACTGCGGCCTCGGGCGTCAAGCCCCCCTCAACTTGG
GCTGTCTTCGCCGACATCTACCGGCGTGAGGGTCTGGCCGGCGTGAACAAGGGTGTGA
ACGCCGTCGCGGTCCGCCAGTGCACGAACTGGGGTTCTCGGTACGCCGTGCCGCTTCA
TGTGATAATCGTTCTGGACTGACCGATGGCAATCAGGATGGGCTTTGCGCGTCTCGCGG
AGACGGCTATCCGGCGGTTACGGGGAGTGGGTGATAATGAGAAGCTCGGCGCCCTGGA
CAAGATTGCGGCGTCCACAGTC

> Yhm2 (FG524)_*Gymnopus androsaceus*
GCTGAAATTACCCGTCACAAAACGGCTGCTACAGGCGTTAAGCCGCCTTCGACCTGGG
CACTCTTTGCCGAGATATACCGCAAGGAAGGTCTCGCAGGCGTGAACAAGGGTGTCAA
TGCGGTCGCCGTCCGTCAATGTACGAATTGGGGTTCTAGGTTCGTCTTGTTCTTTTTCA
CAAGCTTCCGTTGTTATGCTTGCACTTGCACAGAATGGGATTCGCCCGTTTGGCGGAAA
CAAGCATCCGAAAACTTCGGGGAAAGACTGAGGACGATAAACTAGGTGCAATGGACA
AAATCTTGGCTTCATCAATA

> Yhm2 (FG524)_*Marasmius fiardii*
GCTGAAATCACCCGTCAGAAAACCGCTGCCTCGGGTGTCAAACCCCCGTCAACCTGG
GCTCTTTTCATGGAGATTTACCGCAAAGAAGGCATTGCGGGCGTCAACAAAGGTGTCA
ATGCAGTGGCTGTTCGTCAGTGCACCAACTGGGGCTCTCGGTGAGTAAAGTCGGATCA
AATTAAAACGCTAACACATCTCTCACAGCATGGGTTTTGCCCGCCTTGCAGAGTCCACC
ATCCGTAAGATTCGCGGTAAGACTGAAAGCCAAAATCTTTCTGCGATGGATAAGATTCT
TGCTTCTTCGATT

> Yhm2 (FG524)_*Mycena crocata*
GCCGAAATCACGCGCCAAAAGACGGCCGCTACTGGGGTCAAACCACCATCGACATGG
GCGTTGTTTGCCGATATGTACCGTCGGGAAGGGCTTGCAGGGGTGAACAAGGGCGTAA
ACGCTGTTGCGGTCCGCCAGTGCACCAACTGGGGTTCTCGGTGCGATCCTTTGACCGC
GACTTGTCTTGATTCTAGCCAATCCCACTCAGCATGGGCTTCGCCCGTCTTGCAGAGAC
TGGAATCCGGAGACTTCGGGGCAAATCTGACACCGACTATCTCGCTCCAATGGACAAA
ATTCTCGCTTCCTCCATT

> Yhm2 (FG524)_*Tricholoma matsutake*
GTCGAGATAACTCGGCATAAGACTGCGGTGACTGGGGTCAAACCTCCATCGACATGGG
CCGTTTTTGCTGACATATATCGACGCGAAGGCCTTGCCGGAGTTAACAAGGGCGTGAAT
GCCGTTGCAGTTCGCCAGTGCACAAACTGGGGATCTAGGTAAGGAACTGTAATCCTTG
TTTGTATTAAAGTGCTCTGACTCGTCTAGAATGGGTTTTGCTCGCCTGACAGAATCTTC
AATTCGCAAAATCAAAGGTAAAACCGAATCGGACAGTTTAGGAGCTTTCGACAAAATC
CTTTCTTCGACGGTT

> Yhm2 (FG524)_*Hygrophoropsis aurantiaca*
GTCGAGATTACCCGTCATAAAGCAGCTGCAGCTGGTGTAGAACAGACAACGTTCCAGG
TCTTCATGGATATCTACCGTCGGGAAGGTATCAGAGGCATAAACAAGGGTGTAAACGCC
GTTGCTGTACGGCAATGCACTAACTGGGGGTCACGGTAAGACATTTATATACGAAAATG
ATCCAACTAACGTTCGATGACCAGAATGGGTTTCGCTCGTTTGGCTGAAACAATGATCC
GGAATGTTCGGGGCAAGGGGGAGAAAGACCAATTGGGGGCGATGGACAAAATCCTCG
CTTCGTCAATA

> Yhm2 (FG524)_*Boletus edulis*
GTCGAAATCACTCGACACAAAGAAGCCGCAAGTGGTGTACAGCGGTCAACCTTTCAA
GTATTCATGGACATATACCGCAAGGATGGTATTCGCGGAATTAACAAAGGTGTCAACGC
GGTTGCTGTGCGTCAATGTACCAACTGGGGCTCACGGTGCGTTGTTTTATTTAATTTTAT
TGATGATGTTTTAGTATGGGATTTGCCCGGCTGGCTGAGACAATGATCTCGAATATGCGT
GGCAAAGGTGAACACGAGAAACTGAGTGCAGCCGACAAGATCCTT
TCCTCTTCAATT

> Yhm2 (FG524)_*Agaricus subrufescens*
GTCGAGATTACTAGGGCCAAGACCGCTGCTTCGGGAGTTAAGCCCCCATCAACATGGG
CCGTGTTCATGGATATCTATCGCCGCGAAGGTATCGCTGGTGTGAATAAGGGAGTAAAT
GCCGTCGCAGTCCGCCAGTGCACTAACTGGGGTTCACGTATGGGATTCGCCGCCTCGC
GGAATCGTATGATTCGTAAAGTTCGTGGCAAGGGCCAGGATGAGAGCCTCAGCGCTTT
CGAAAAGNTACTCAGCCTCGACTGT

> Yhm2 (FG524)_*Gautieria morchelliformis*
GCTGAAATCACAAGGCACAAACAGGCTGCAGCGGGCGTGAAACCACCTTCCACTTGG
GCACTATTTGCTGACATATACCGTCGAGAGGGTATTAAGGGTATCAACAAAGGTGTCAA
TGCCGTAGCTGTCCGTCAGTGTACCAACTGGGGTTCTAGGTACGCCGGAATACATTTTG
CAAGGGCGATAATAAAGGTCATGATACAGGATGGGCTTCGCTCGTTTAGCTGAAGACC
TGATACGTACAACACGGGGCAAGCAGCAAGGTGAGTCCTTGAGTGCGCTCGACAAGA
TTCTCAGTTCGGCAATT

> Yhm2 (FG524)_*Ramaria acris*
GCTGAGATCACCCGCCATAAACAAGCTGCTTCGGGCGTGAAACCACCATCAACATGGG
CTCTGTTTGCTGACATTTACCGTCGAGAGGGTATCAAGGGCATCAACAAGGGTGTTAA
CGCCGTGGCCGTACGCCAATGCACTAACTGGGGGTCCAGGTGAGCTGAAAATCACTTG
ATAAAGTGGCTGCCCCTGACCACCGTCTTCTAGGATGGGTTTCGCTCGCTTAGCAGAG
GACATGATTCGCAAGGCACGGGGCAAGCAACACGGTGAATCACTGAGCGCCCCCGAC
AAAATTTTCAGTTCAGCAATC

> Yhm2 (FG524)_*Auricularia delicata*
GCAGAAATTACGCGCCACAAGCAGGCCGAGTCCGGTATCAAGCCCCCGTCGACCTGG
CAAGTTTTCGCCGATATCTATAGGCGTGAGGGTATTGCGGGCATCAACAAGGGCGTCAA

CGCGGTGGCCATCCGGCAGTGCACGAACTGGGGCGCCCGGTGCGTGCTCAGACGCAC
ATTCCCACCGTGCGAGCTGAACTGGCGGACAGCATTGGTCTCGCGCGCTTAGCTGAGA
GCTCGATCCGGAAACTCCAGGGCAAGACGGAGAAGGAGCAGCTTGGCGCGTTTGAGA
AGATTGCAAGTTCAACCATC

> Yhm2 (FG524)_*Asperigillus triticus*
GTGGAGATCACCAAGCACAAGATGGCTGCCCAGGGCGTGAAGCCGCCCAGCACCTTT
GCGACCTTCATGGACATCTACCGCCGCGAGGGCATCCGGGGTATCAACCGTGGTGTGA
ACGCCGTCGCCATCCGCCAGACGACGAACTGGGGTTCTCGCTTCGGTCTCTCTCGTCT
GGCCGAGTCGGCCATCCGTAGCGTGACGGGCAAGGACGACAAGGAGAAGCTGGGCG
CCGCGGAGAAGGTCCTCGCCTCGGGTCTG

> Yhm2 (FG524)_*Neurospora crassa*
GTCGAGATTACCAAGCACAAGATGAGCGCCGCCGGCCAGAAGGCCCCCGGCACTTGG
GCTACCTTCATGGACATCTACCGCCGCGAGGGTATCCGCGGTATCAACAAGGGCGTCA
ACGCCGTTGCCATTCGCCAGATGACCAACTGGGGTTCCCGTTTCGGTCTCTCCCGCCTT
GCTGAGCAGGGTATCCGCAAGGCCACTGGCAAGGAGGAGGGACAGAAGCTGGCCGC
GTGGGAGAAGATTCTCGCCTCCGCCCTT

> Yhm2 (FG524)_*Agaricostilbum hyphaenes*
GCCGAAATTACAAGACACAAACAAGCTGAAGCTGGTCTCAAACCTCCTGGTACTATGG
AGACTTTCATGAACATCTACAGGAAGGAGGGTCTGGCTGGCATCAACAAGGGTGTCAA
CGCTGTCGCGTAGGTTCGATGCCCGACAATAAGCCCATCTTCAGCTTATCGTGGGTGAT
GTCGAACAGGATTCGACAAATATTGGGGATCTCGCATGGGCTTTGCAAGATTGGCAGA
GCAGAGCATTCGCAAAGCTCGAGGCAAGAGGGAAGACGAGAAGCTGGGTGCTTTGGA
TCGTGTCTTTGCTAGCTCTGTC

> Yhm2 (FG524)_*Rhizopus microsporus*
GTCGAAGTGACAAGACAAAAATCTGTTGCAGCTGGCACAGAGTCTACTTTTGCAATTG
CTGGTAAAATATTTAAGAAGGAAGGTATCAGAGGAATGAATAAGGGTGTGAATGCGGT
AGCCCTTAGACAATGTACCAACTGGGCATCCAGATTTGGTATTGCGCGTTTTGCTGAAG
AAGCCATCGTTAAACTAAGACATGGCGAAAAGGGTGTAGCAGAAGGAACTGACAAAG
CACTGGCATCTGTAGTA
