# Supplementary material for: The saprotrophic Pleurotus ostreatus species complex: late Eocene origin in East Asia, multiple dispersal, and complex speciation
Source: IMA Fungus. 2020 Jun 8;11:10. doi: 10.1186/s43008-020-00031-1 (PMC7325090; doi:10.1186/s43008-020-00031-1)
Supplement: Supplementary file 5 — Additional file 5: R Commands to perform the analyses of diversification rates. [file 43008_2020_31_MOESM5_ESM.pdf]

R Commands to perform the analyses for the analyses of diversification rates

```
########################################################################
# Diversification rate analysis using "BAMMtools" package for R
########################################################################
library(BAMMtools)
tree <- read.nexus("Pleurotus-tree.tre")
is.ultrametric(tree)
is.binary.tree(tree)
min(tree$edge.length)
# Decide burnin percentage
mcmcout <- read.csv("C:/bamm/mcmc_out.txt")
plot(mcmcout$logLik ~ mcmcout$generation)
# Check if the post burnin effective sampling size is >>200
burnstart <- floor(0.2 * nrow(mcmcout))
postburn <- mcmcout[burnstart:nrow(mcmcout), ]
effectiveSize(postburn$N_shifts)
effectiveSize(postburn$logLik)
#Load the main BAMM output: the event data; Use the determined burnin value.
edata <- getEventData(tree, eventdata = "C:/bamm-/event_data.txt", burnin=0.2)
summary(edata)
plot.bammdata(edata, tau=0.001,breaksmethod="quantile",lwd=2, pal="temperature",legend=T)
#View 95% most credible shift set
edata <- getEventData(tree, eventdata = "C:/bamm/event_data.txt", burnin=0.2)
css <- credibleShiftSet(edata, expectedNumberOfShifts = 1, threshold=5, set.limit=0.95)
css$number.distinct
summary(css)
plot.credibleshiftset(css, lwd=1.7,plotmax=4)
#Macroevolutionary cohort matrix
cmat <- getCohortMatrix(edata)
cohorts(cmat, edata, lwd=3, pal="temperature", use.plot.bammdata=TRUE)
# Plot net diversification rate-through time for the specific clades
tree <- read.nexus("Pleurotus-tree.tre")
edata <- getEventData(tree, eventdata = "C:/bamm/event_data.txt", burnin=0.2)
plot.new()
st<-max(branching.times(tree))
plotRateThroughTime(edata,intervalCol="red",avgCol="red",start.time=st,ylim=c(0,0.15),cex.axis
=2)
########################################################################
# Lineages through time (LTT) analysis using “Ape” package for R
########################################################################
library(ape)
tree<-read.nexus("Pleurotus-tree.tre")
tree_1<-read.nexus("Pleurotus-tree.nxs.trees.txt")
tree_2<-tree_1[5000: 9999]
```

```
ltt.plot<-function(tree,tree_2)
{
pdf("Pleurotus_ltt_result.pdf")
mltt.plot(tree_2,dcol=TRUE,dlty=FALSE,legend=FALSE,xlab="Time(million-year)",ylab="The
number of lineages",log="y")
ltt.lines(tree,lwd=3,co="red")
title(main="Lineages Through Time Plot")
dev.off()
}
ltt.plot(tree,tree_2)
######################################################################
# “Laser” analysis using “geiger” and “laser” packages for R
######################################################################
library(geiger);
library(laser);
data<-read.tree("C:/ Pleurotus-tree.newick")
data1<-getBtimes(string = write.tree(data))
data1
result <- fitdAICrc(data1, modelset = c("pureBirth", "bd","DDX", "DDL","yule2rate"), ints = 100)
```
