# Supplementary figures and images for: The saprotrophic Pleurotus ostreatus species complex: late Eocene origin in East Asia, multiple dispersal, and complex speciation
Source: IMA Fungus. 2020 Jun 8;11:10. doi: 10.1186/s43008-020-00031-1 (PMC7325090; doi:10.1186/s43008-020-00031-1)

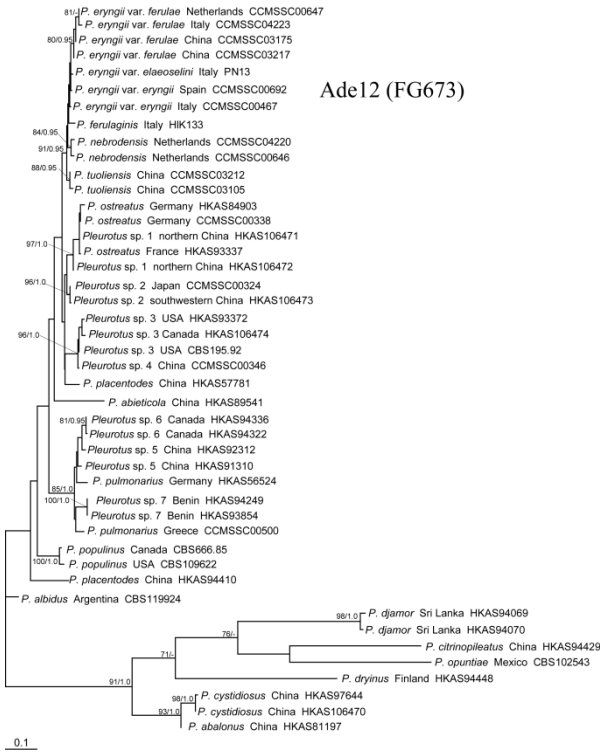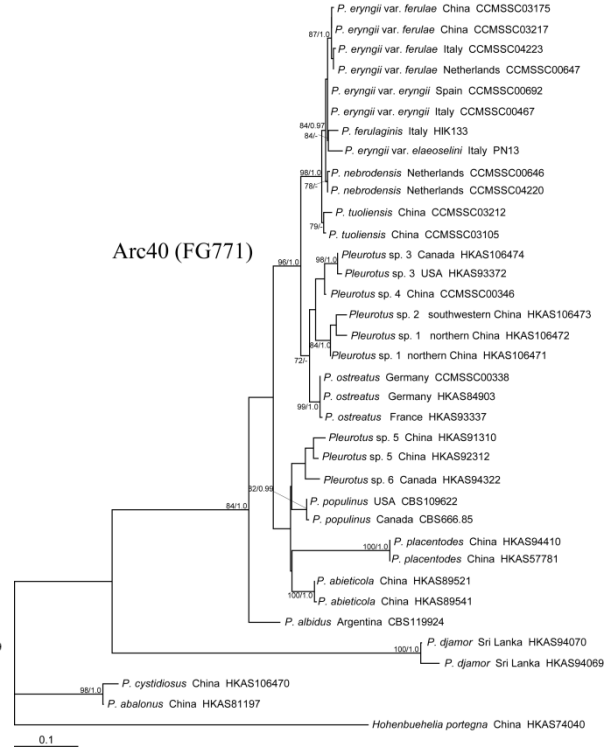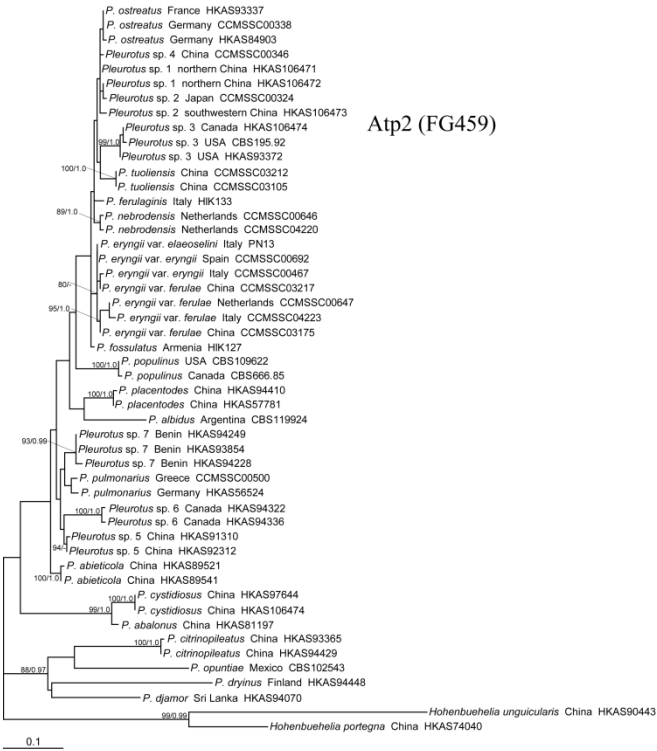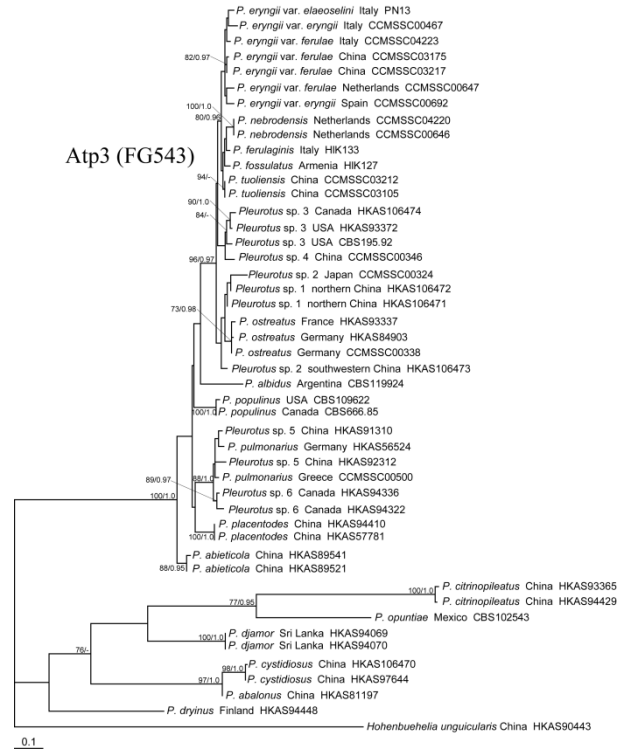

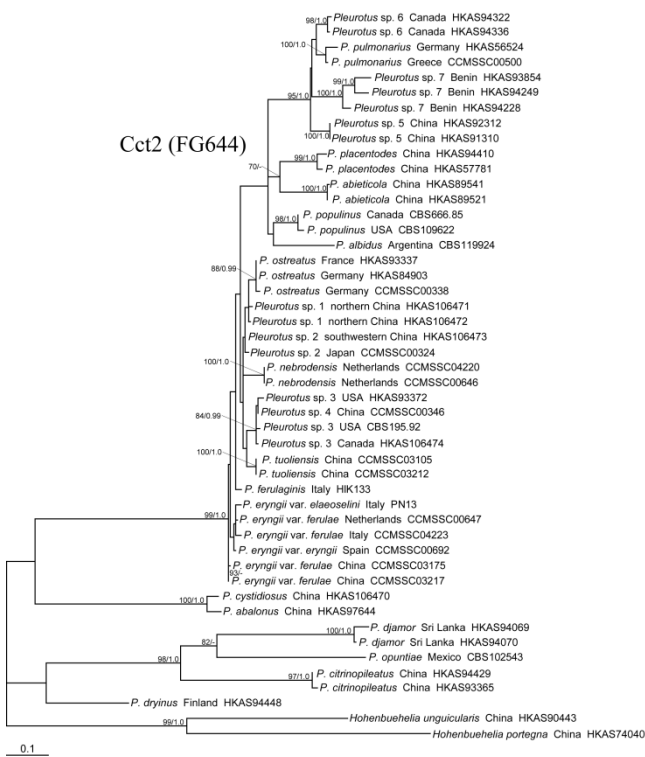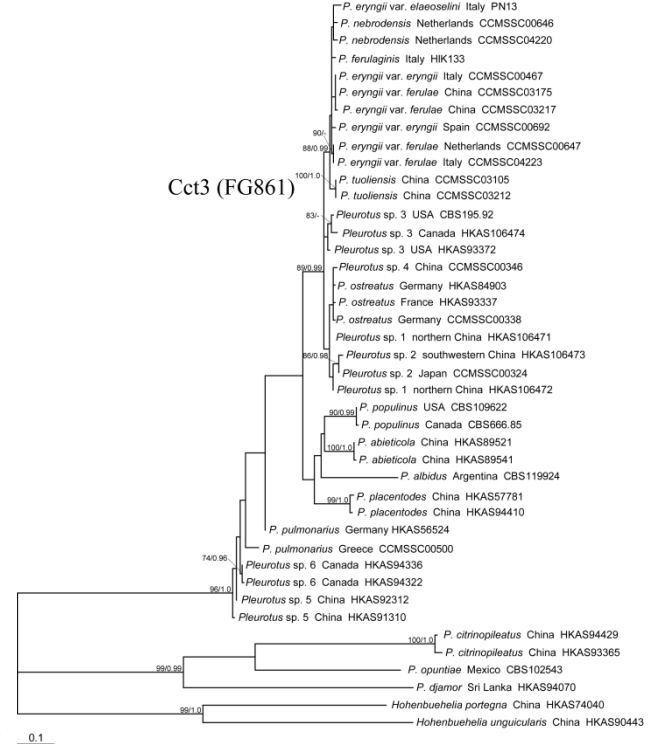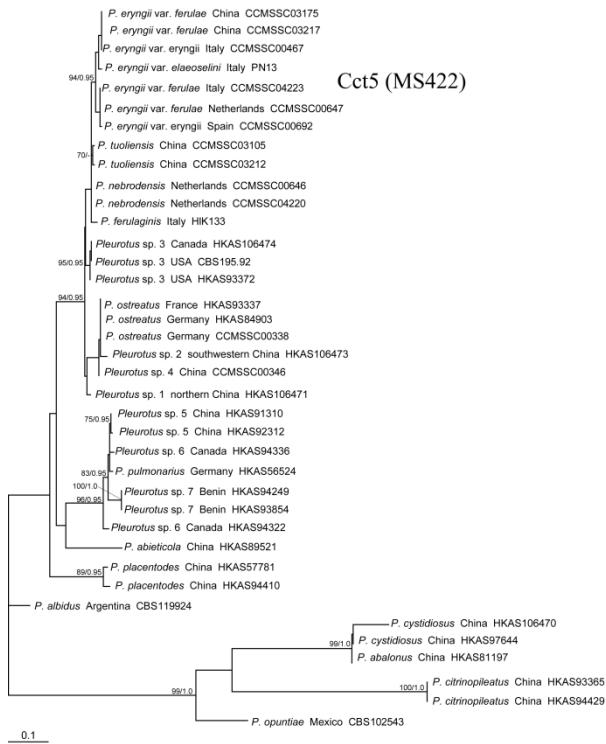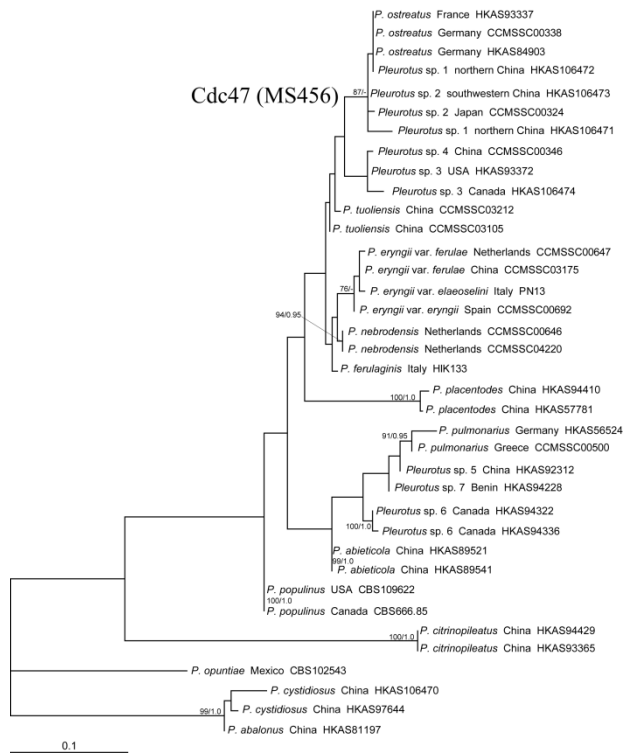

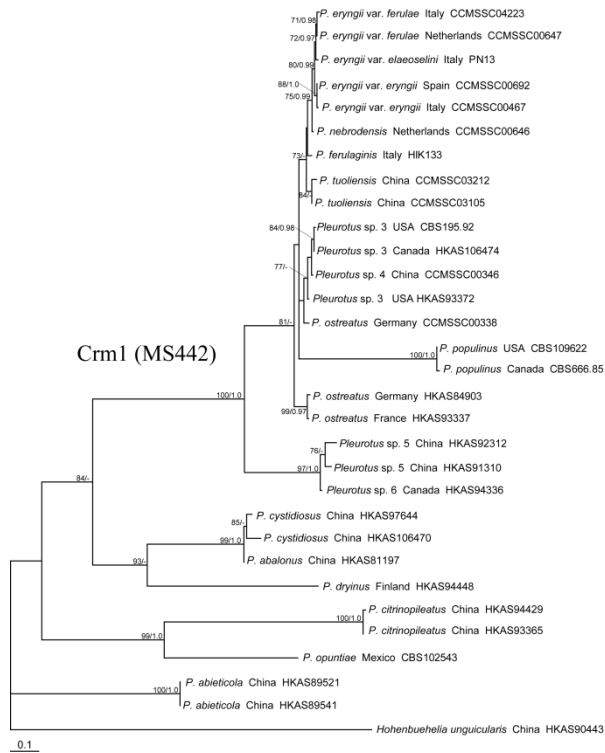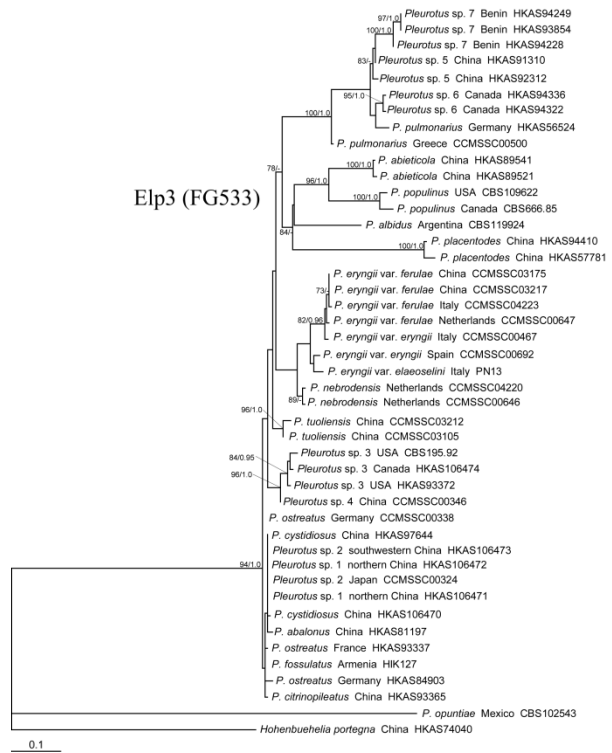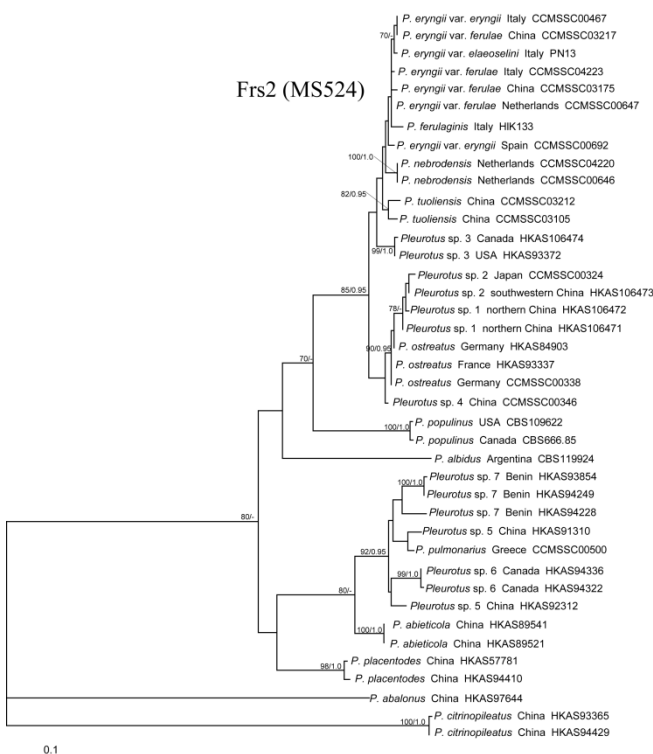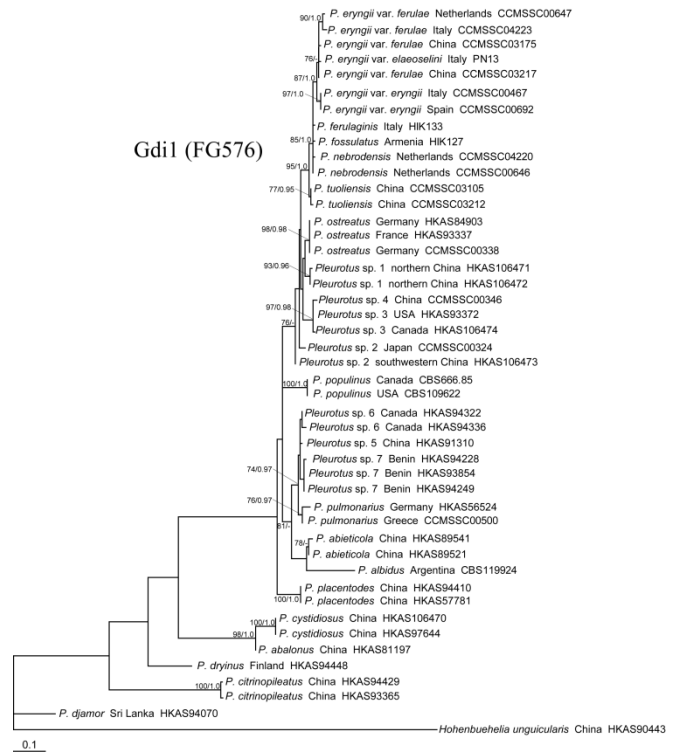

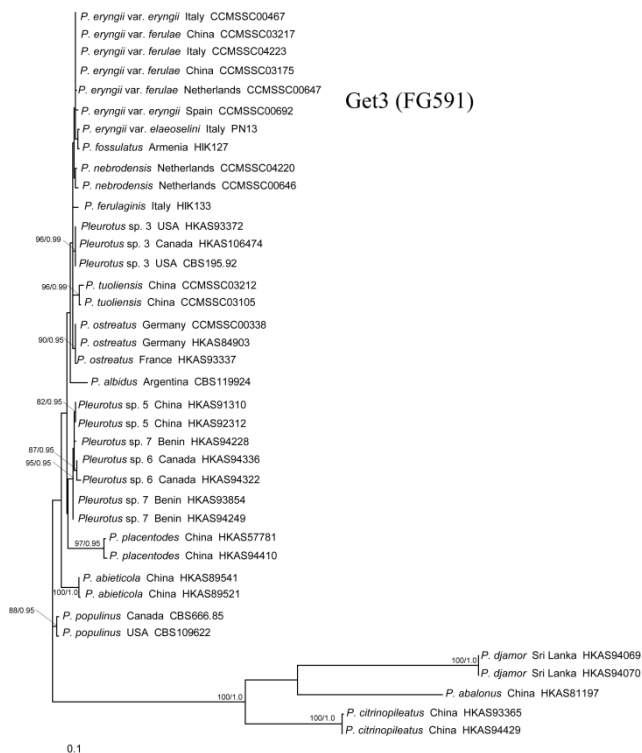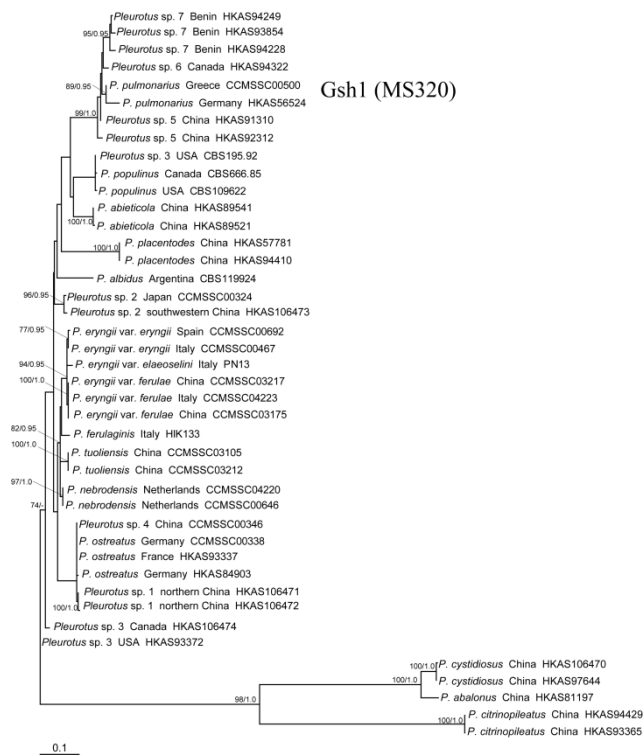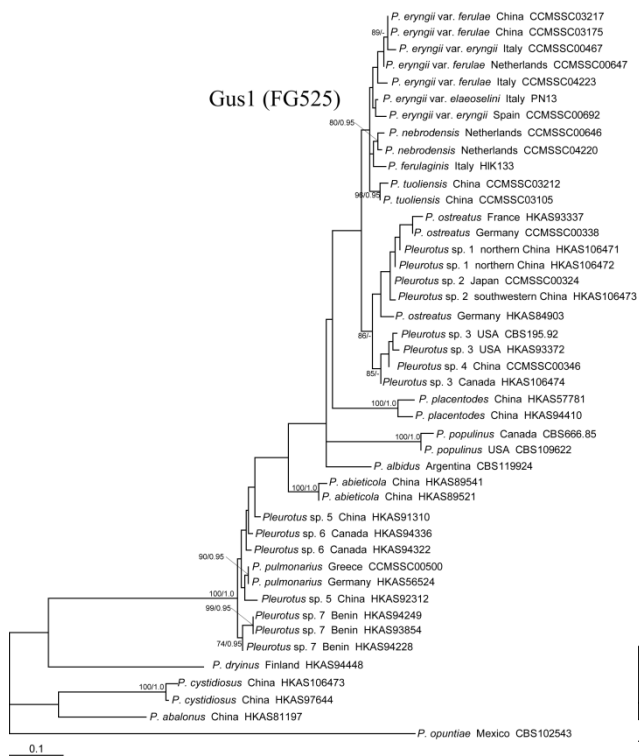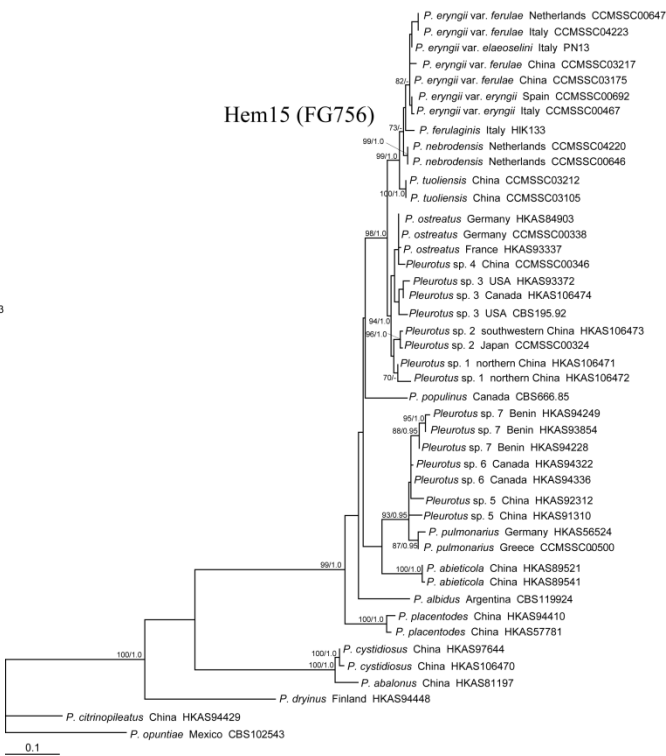

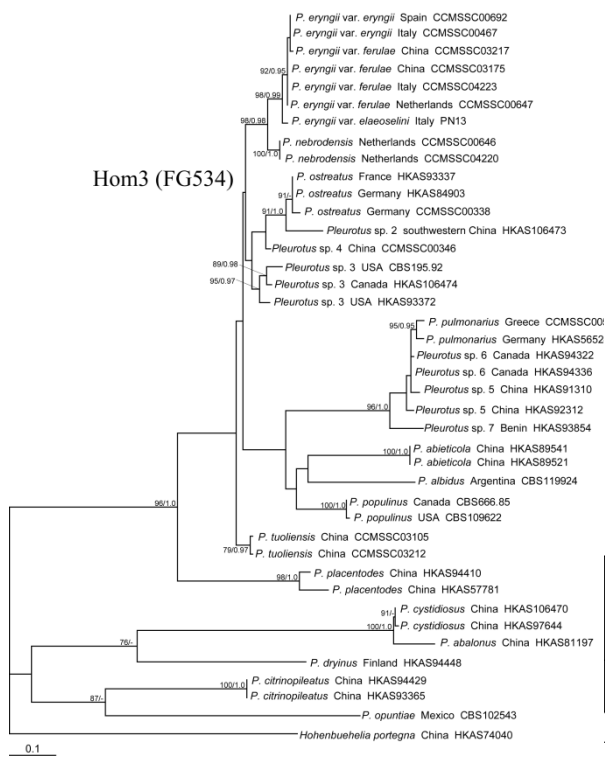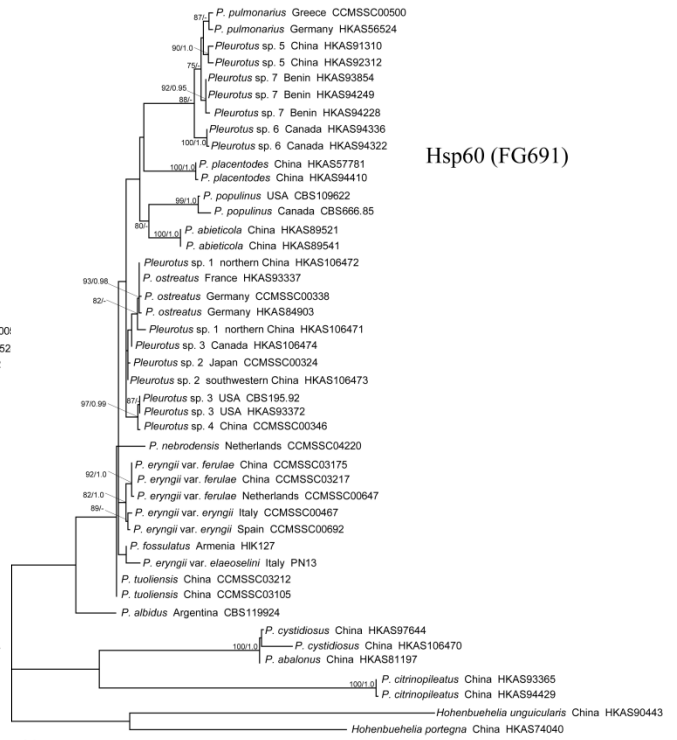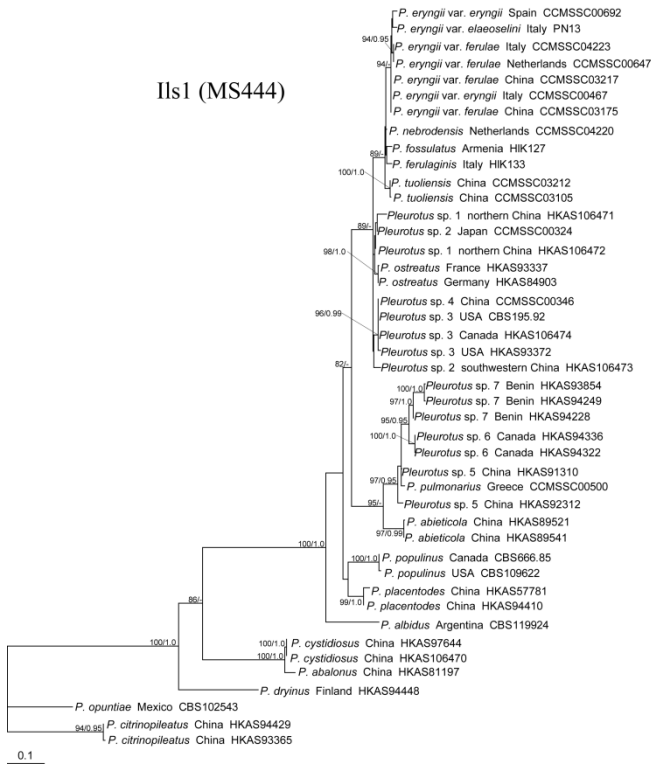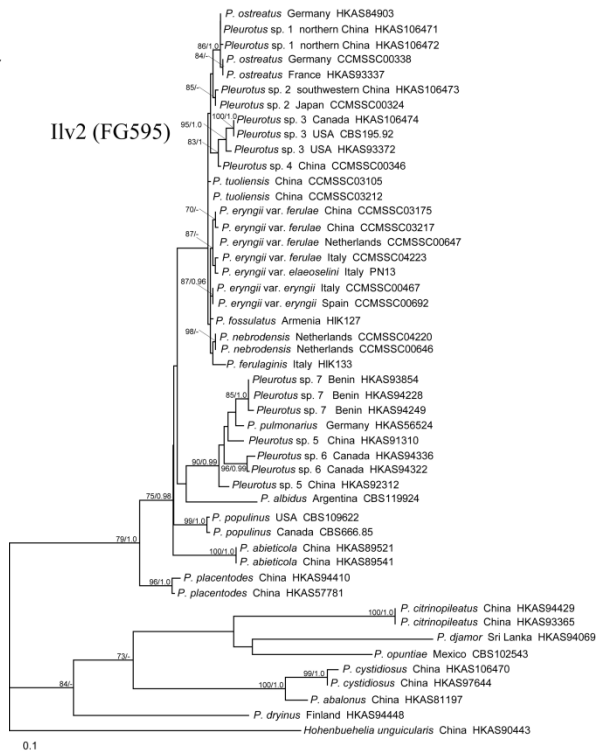

Krr1 (FG695)

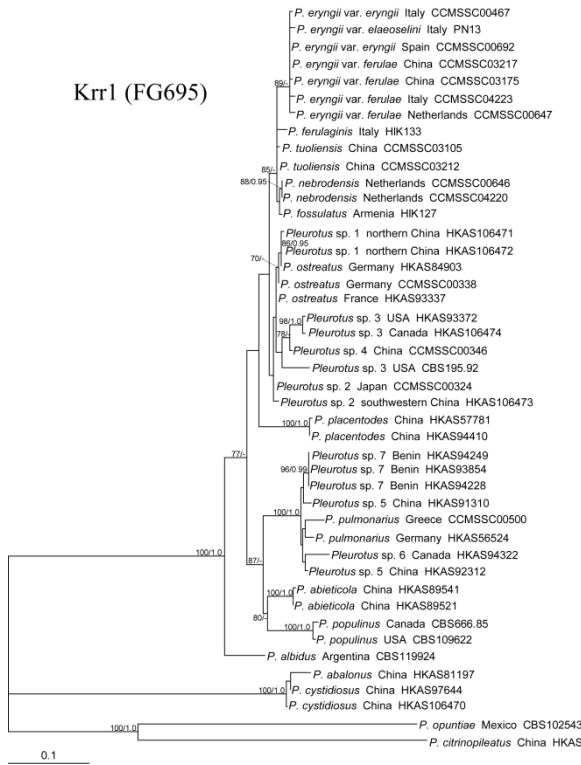

Mcm2 (MS463)

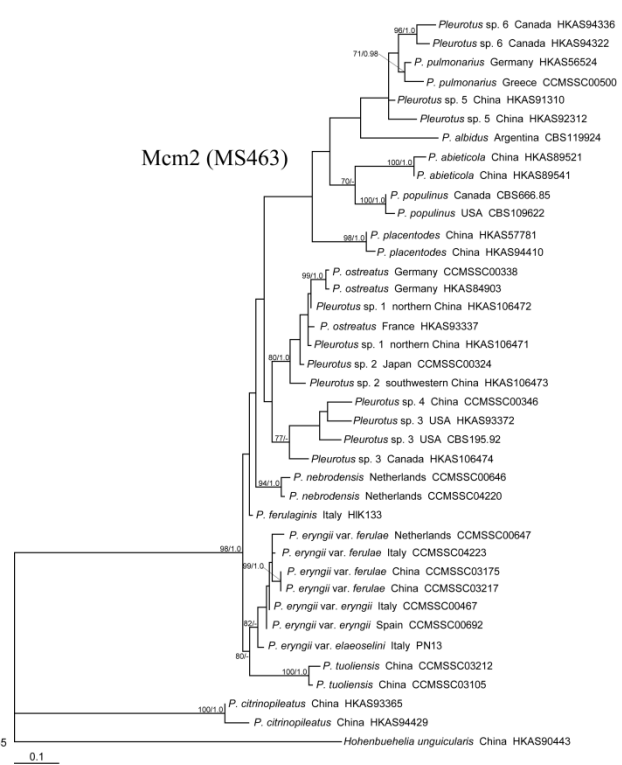

Met6 (FG740)

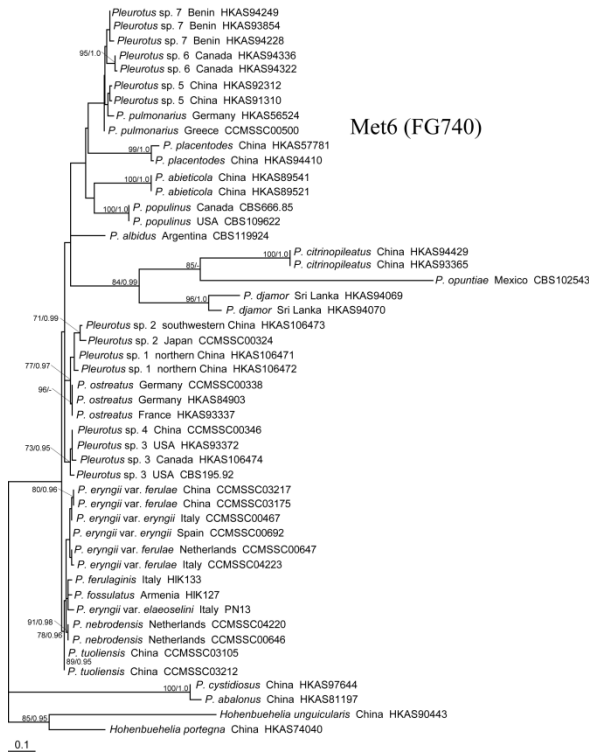

Pdb1 (FG855)

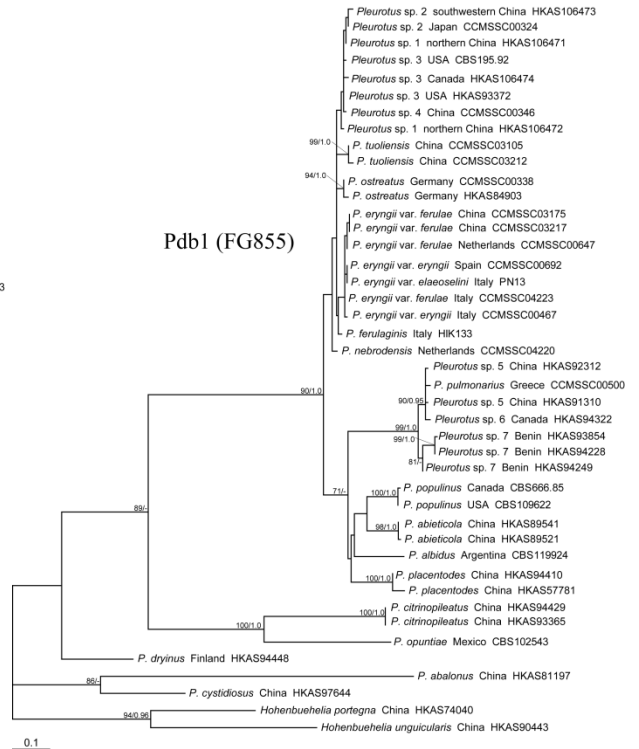

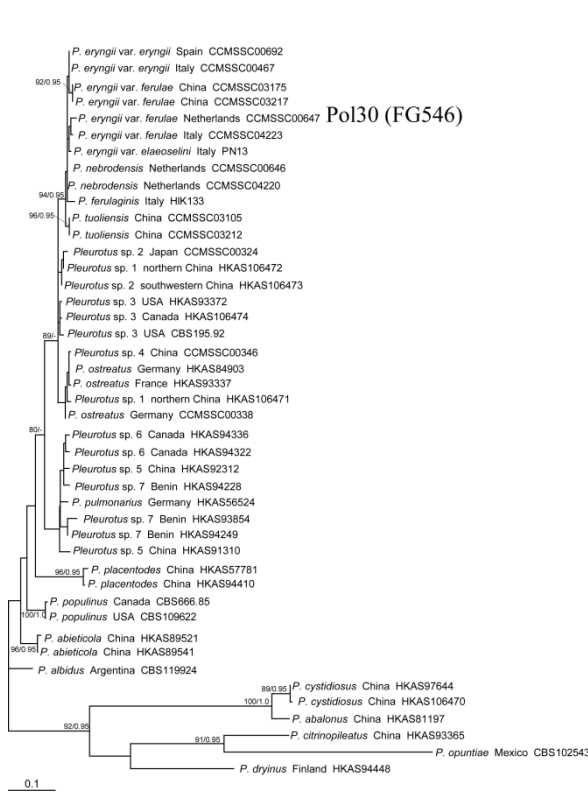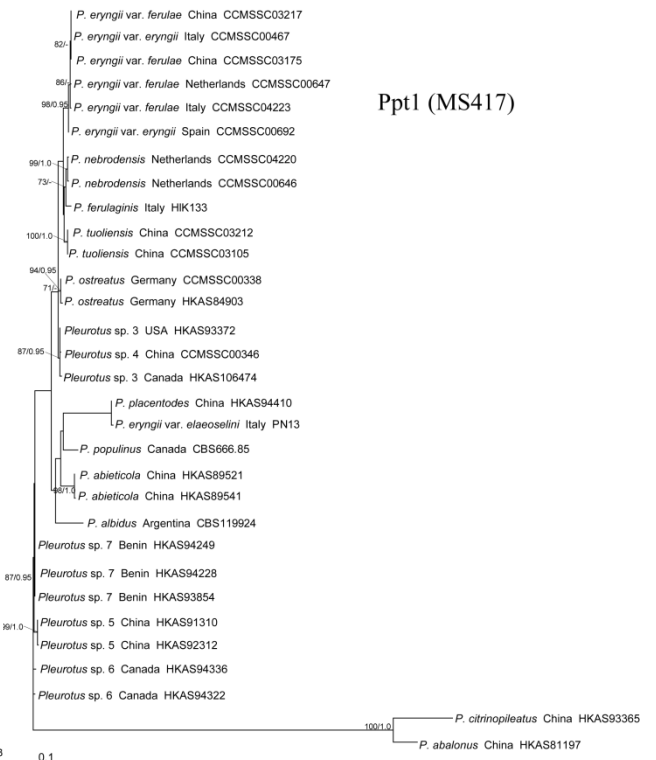

Ppt1 (MS417)

Pre8 (MS429)

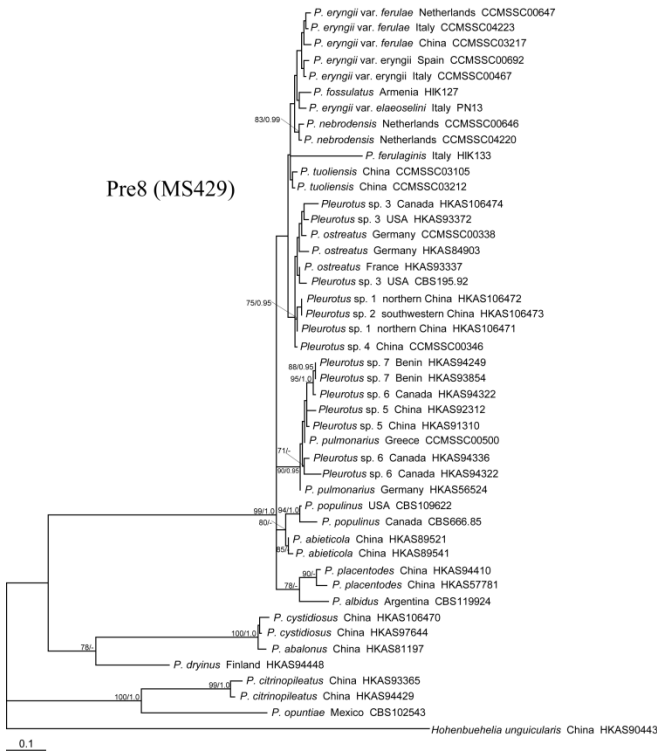

Qns1 (FG747)

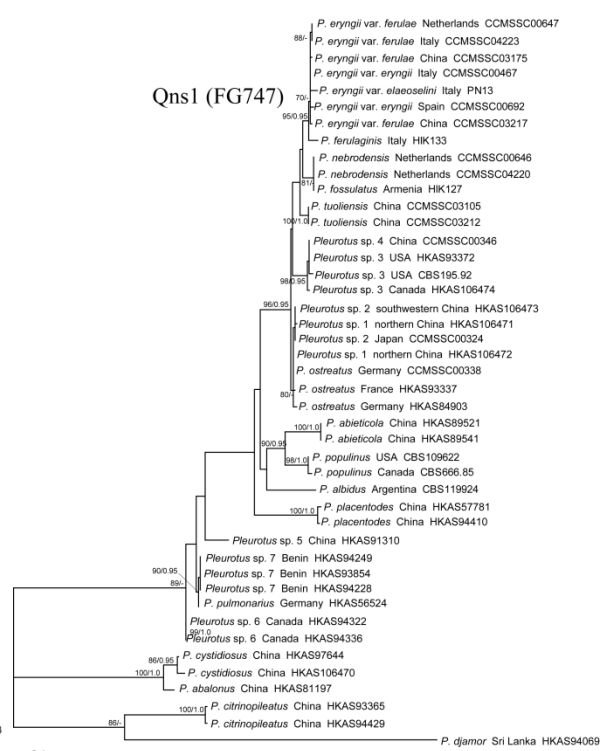

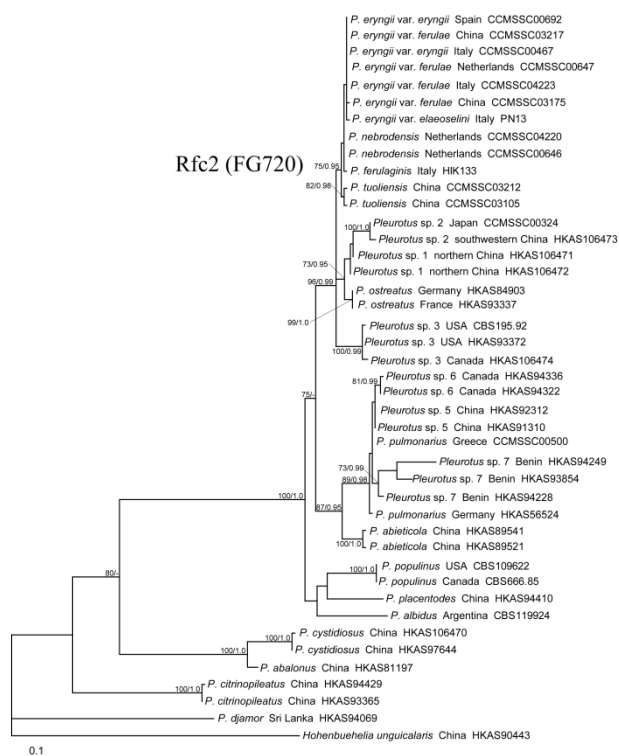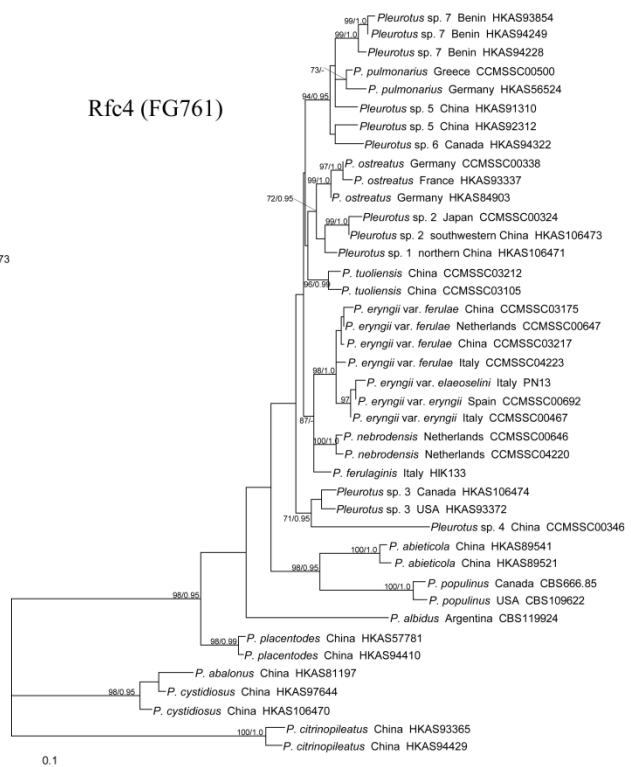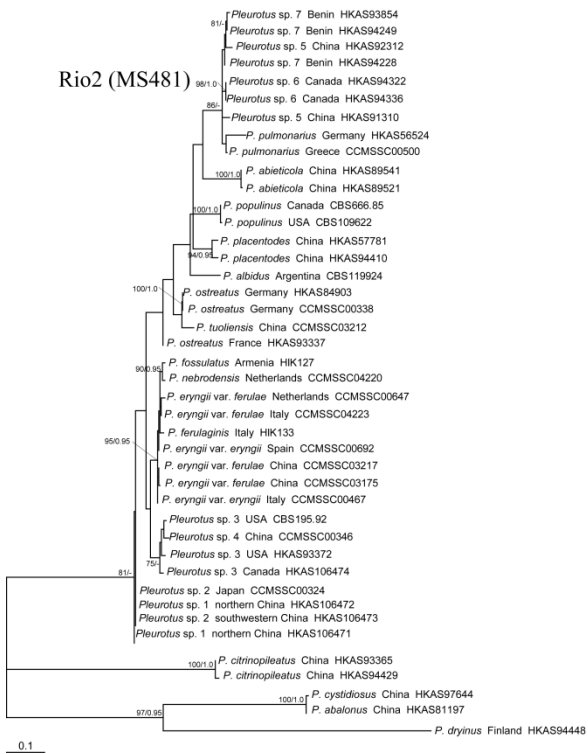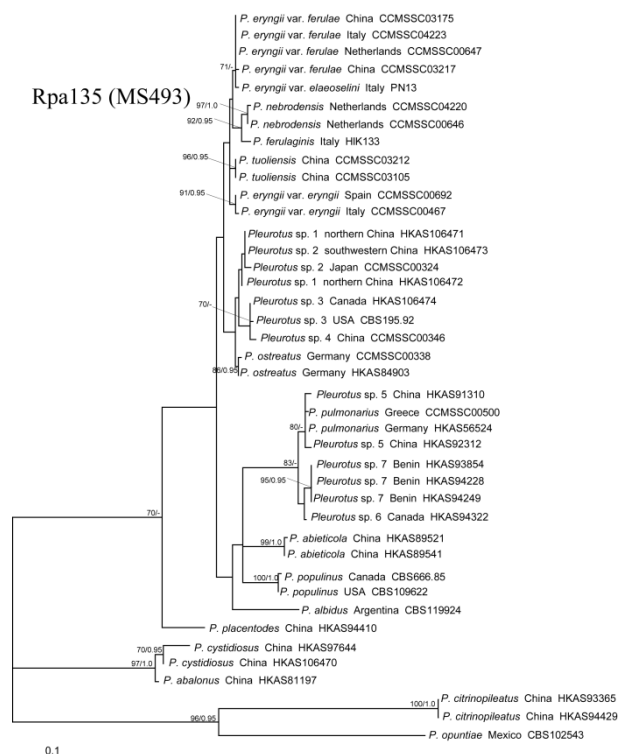

Sac6 (FG975)

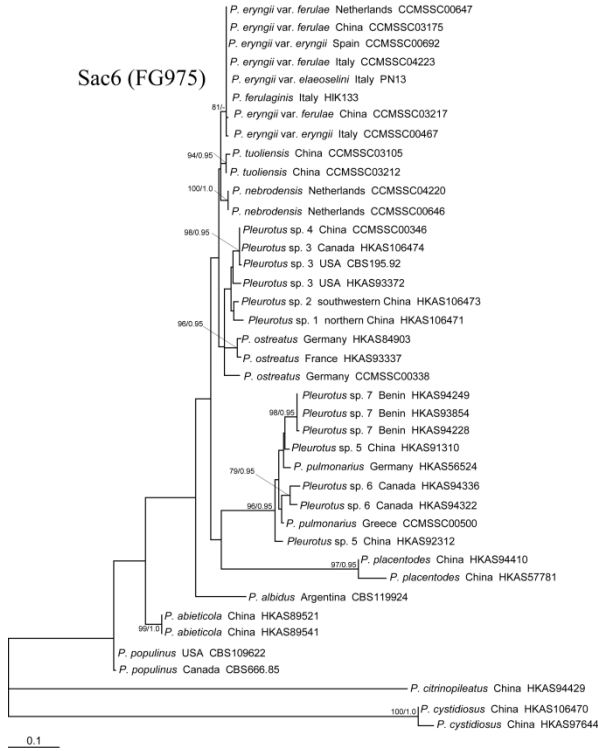

Stt3 (MS561)

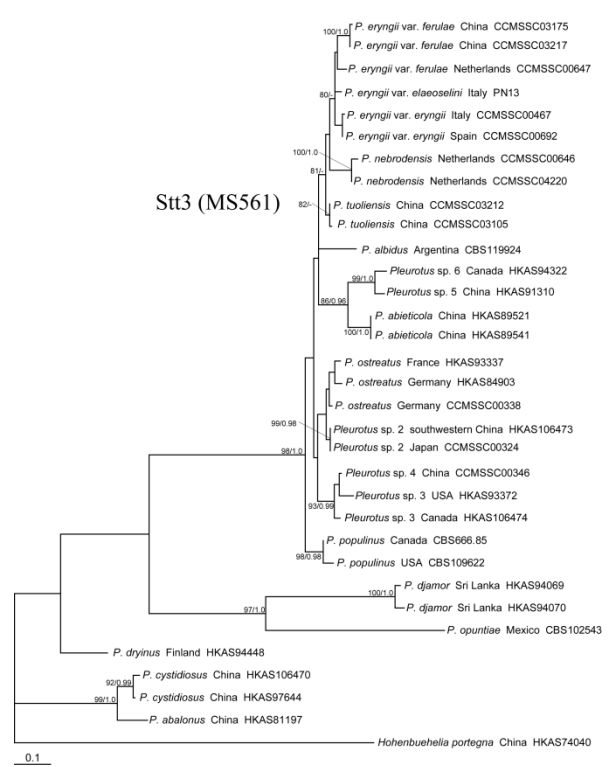

Tcp1 (FG850)

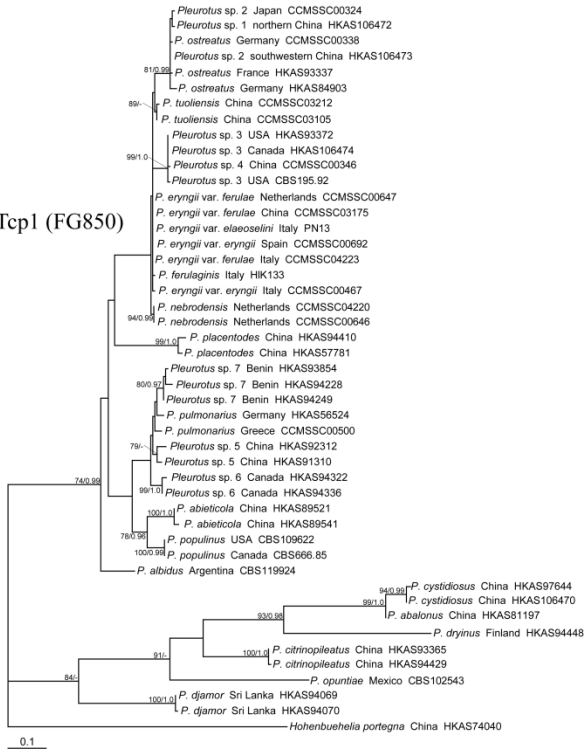

Trp2 (MS353)

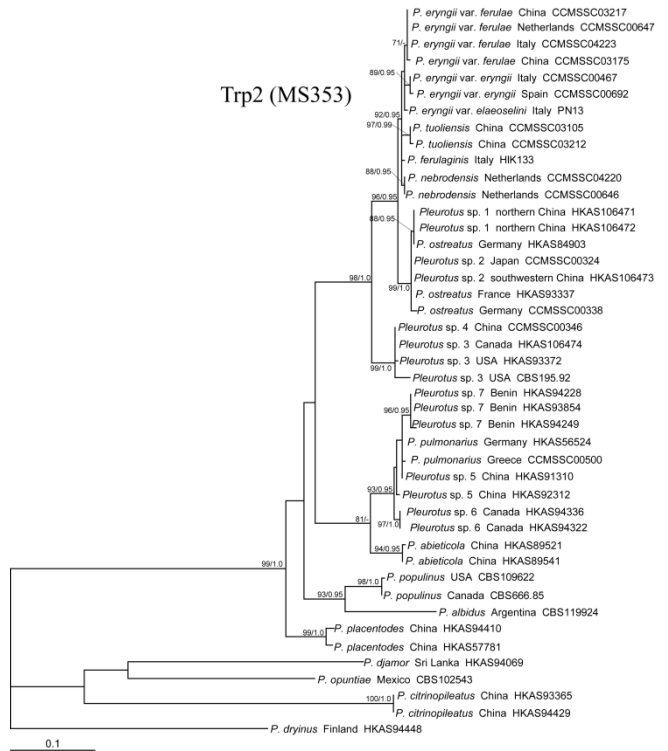

Uba1 (FG848)

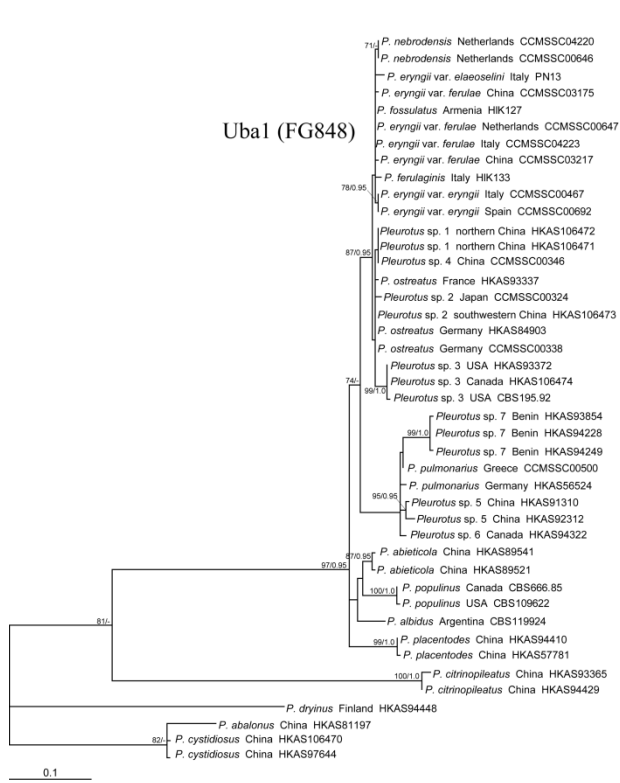

Uba3 (FG844)

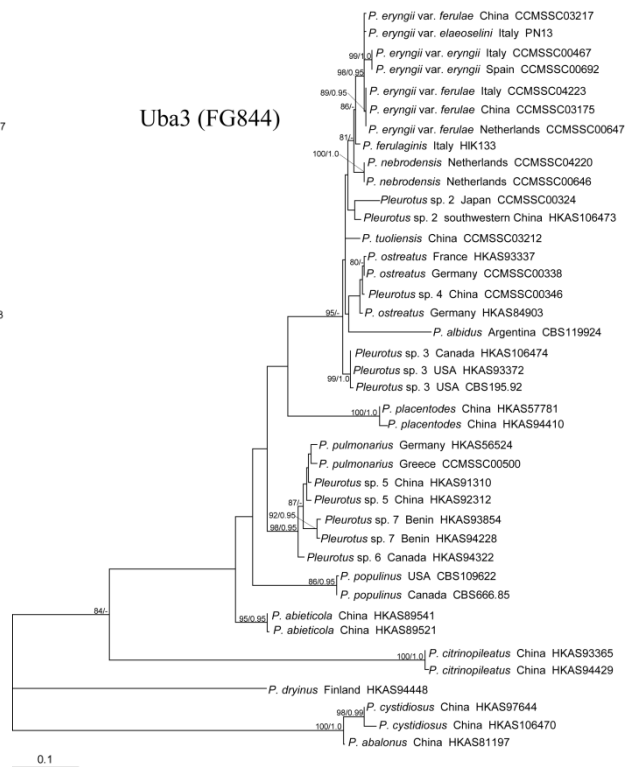

Ygr207c (FG757)

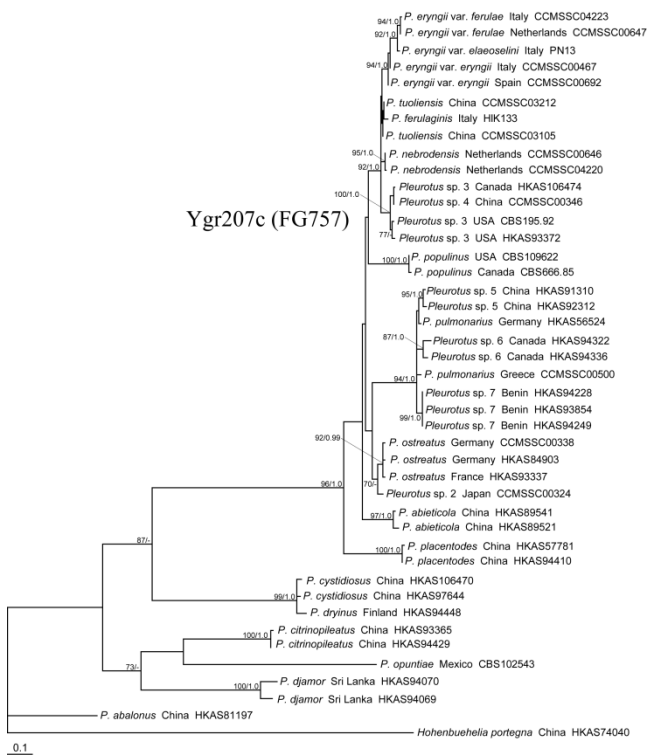

Yhm2 (FG524)

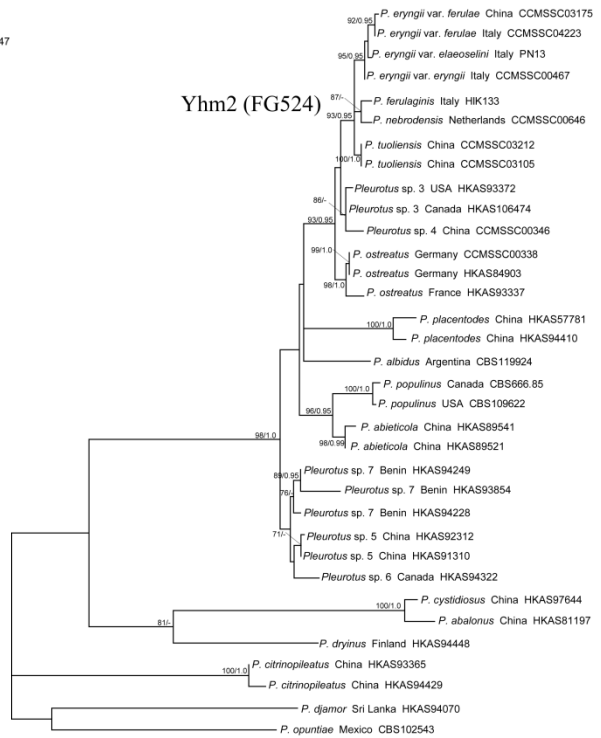

Supplement: Supplementary file 6 — Additional file 6: Phylogenetic tree inferred from ML analysis based on each single-copy gene. Branch support values are indicated by numbers above branches (MLB ≥ 70%, BPP ≥ 0.95). Taxon labels are listed in Table 1. [file 43008_2020_31_MOESM6_ESM.pdf]

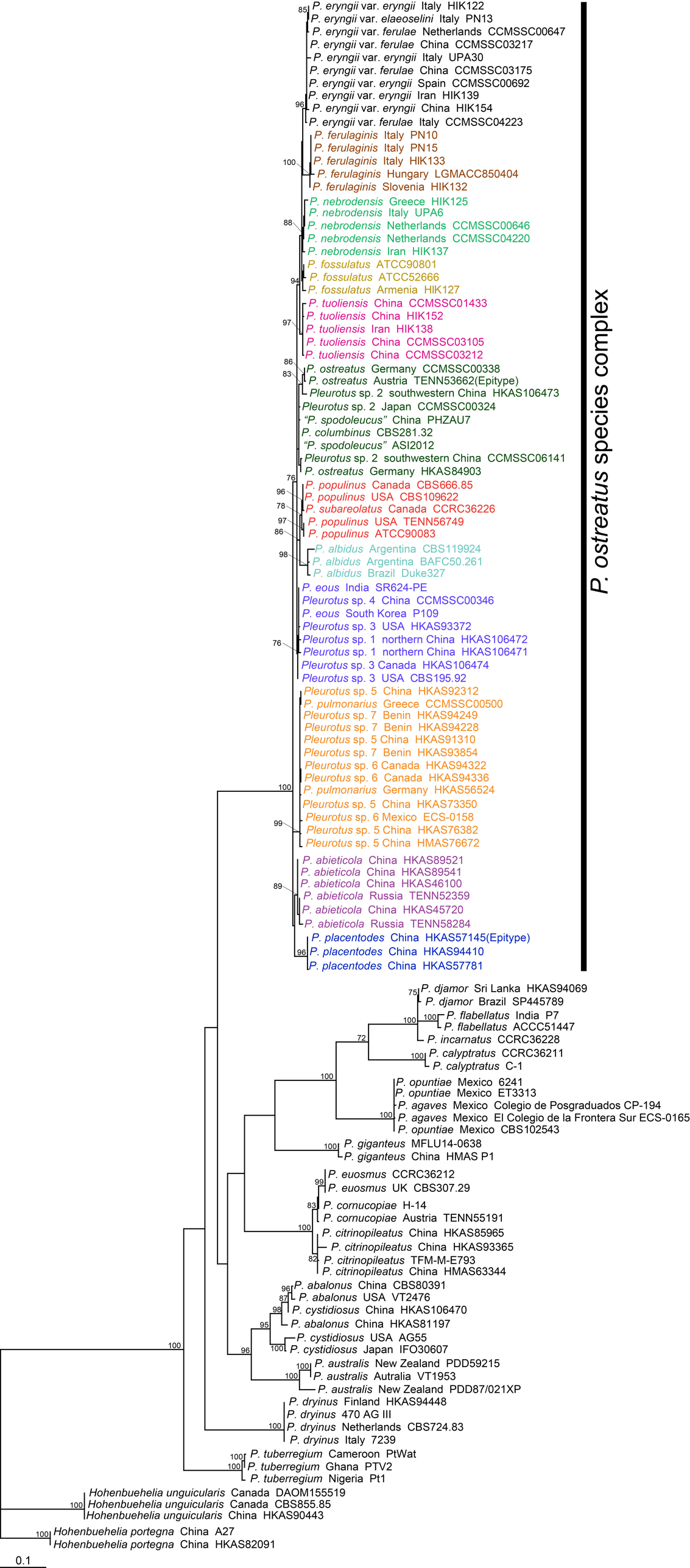

Supplement: Supplementary file 7 — Additional file 7: Phylogenetic relationships of Pleurotus inferred from ITS sequences using ML analysis. Branch support values are indicated by numbers above branches (MLB ≥ 70%). Accession numbers for sequences retrieved from GenBank database are listed in Additional file 2. [file 43008_2020_31_MOESM7_ESM.jpg]

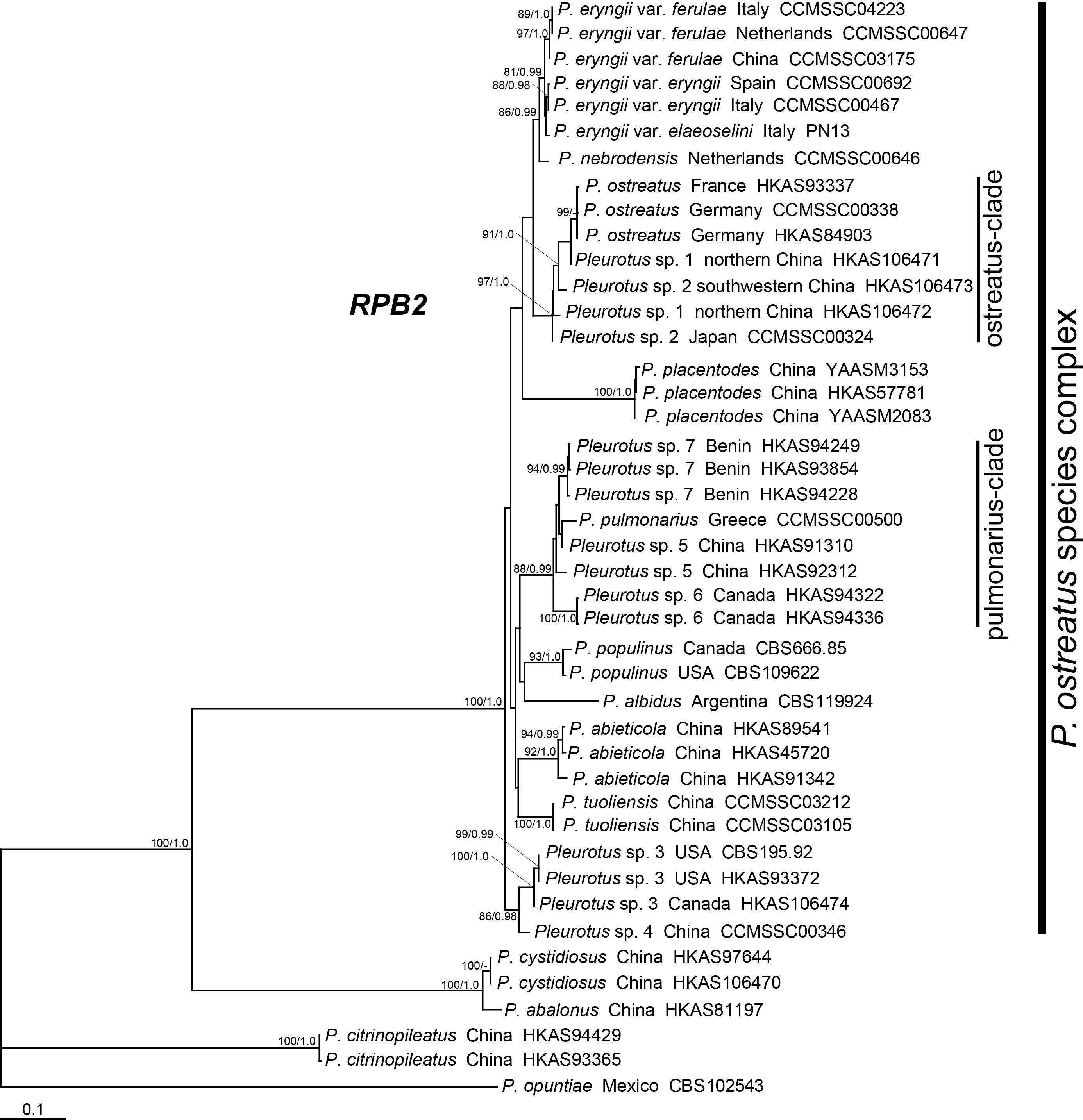

Supplement: Supplementary file 9 — Additional file 9: Phylogenetic relationships of the P. ostreatus species complex inferred from RPB2 sequences using ML analysis. Branch support values are indicated by numbers above branches (MLB ≥ 70%, BPP ≥ 0.95). Provisionally adopted names (based on tree topology) are listed. Accession numbers for sequences generated newly and retrieved from GenBank database are listed in Table 1. [file 43008_2020_31_MOESM9_ESM.jpg]
