# Supplementary material for: The saprotrophic Pleurotus ostreatus species complex: late Eocene origin in East Asia, multiple dispersal, and complex speciation
Source: IMA Fungus. 2020 Jun 8;11:10. doi: 10.1186/s43008-020-00031-1 (PMC7325090; doi:10.1186/s43008-020-00031-1)
Supplement: Supplementary file 8 — Additional file 8: Average evolutionary divergence over RPB2 sequences pairs within and between groups (provincially adopted phylogenetic species) calculated by MEGA 5. [file 43008_2020_31_MOESM8_ESM.doc]

Additional file 8 Average evolutionary divergence over *RPB2* sequences pairs within and between groups (provincially adopted phylogenetic species) calculated by MEGA 5

| Provincially adopted species | Inter-specific variations (between groups) | | | | | | | | | | | | Intra-specific variations (within groups) |
| --- | --- | --- | --- | --- | --- | --- | --- | --- | --- | --- | --- | --- | --- |
| “ostreatus-clade” |  |  |  |  |  |  |  |  |  |  |  |  | 0.0108 |
| *P. eryngii* var. *ferulae* | 0.0357 |  |  |  |  |  |  |  |  |  |  |  | 0.0015 |
| *P. eryngii* var. *eryngii* | 0.0347 | 0.0091 |  |  |  |  |  |  |  |  |  |  | 0.0012 |
| *P. eryngii* var. *elaeoselini* | 0.0339 | 0.0082 | 0.0052 |  |  |  |  |  |  |  |  |  | n/c |
| *P. nebrodensis* | 0.0361 | 0.0169 | 0.0131 | 0.0153 |  |  |  |  |  |  |  |  | n/c |
| *Pleurotus* sp. 4 | 0.0239 | 0.0302 | 0.0284 | 0.0268 | 0.0287 |  |  |  |  |  |  |  | n/c |
| *Pleurotus* sp. 3 | 0.0567 | 0.0559 | 0.0501 | 0.0515 | 0.0509 | 0.0174 |  |  |  |  |  |  | 0.0019 |
| “pulmonarius-clade” | 0.0705 | 0.0608 | 0.0611 | 0.0619 | 0.0619 | 0.0469 | 0.0588 |  |  |  |  |  | 0.0181 |
| *P. tuoliensis* | 0.0592 | 0.0558 | 0.0541 | 0.0569 | 0.0537 | 0.0374 | 0.0418 | 0.0607 |  |  |  |  | 0 |
| *P. populinus* | 0.0718 | 0.0639 | 0.0640 | 0.0652 | 0.0615 | 0.0409 | 0.0587 | 0.0599 | 0.0614 |  |  |  | 0.0079 |
| *P. abieticola* | 0.0717 | 0.0627 | 0.0609 | 0.0624 | 0.0593 | 0.0528 | 0.0673 | 0.0682 | 0.0597 | 0.0709 |  |  | 0.0093 |
| *P. albidus* | 0.0787 | 0.0711 | 0.0709 | 0.0704 | 0.0640 | 0.0586 | 0.0791 | 0.0799 | 0.0741 | 0.0762 | 0.0838 |  | n/c |
| *P. placentodes* | 0.0984 | 0.0921 | 0.0924 | 0.0947 | 0.0922 | 0.0821 | 0.1007 | 0.1091 | 0.0983 | 0.1057 | 0.1135 | 0.1190 | 0.0024 |
