# Supplementary material for: The saprotrophic Pleurotus ostreatus species complex: late Eocene origin in East Asia, multiple dispersal, and complex speciation
Source: IMA Fungus. 2020 Jun 8;11:10. doi: 10.1186/s43008-020-00031-1 (PMC7325090; doi:10.1186/s43008-020-00031-1)
Supplement: Supplementary file 10 — Additional file 10: Criteria for phylogenetic species recognized by genealogical concordance in individual gene and in the combined dataset [file 43008_2020_31_MOESM10_ESM.doc]

Additional file 10 Criteria for phylogenetic species recognized by genealogical concordance in individual gene and in the combined dataset

| Single-copy gene | *P. abieticola* | *P. eryngii*  var. *eryngii* | *P. eryngii* var. *ferulae* | *P. nebrodensis* | *P. ostreatus* | *P. placentodes* | *P. populinus* | *P. pulmonarius* | *P. tuoliensis* | *Pleurotus* sp. 1 | *Pleurotus* sp. 2 | *Pleurotus* sp. 3 | *Pleurotus* sp. 5 | *Pleurotus* sp. 6 | *Pleurotus* sp. 7 |
| --- | --- | --- | --- | --- | --- | --- | --- | --- | --- | --- | --- | --- | --- | --- | --- |
| Ade12 | #/# | -/- | 80/0.95 | 84/0.95 | -/- | -/- | 100/1.0 | -/- | 88/0.95 | -/- | 96/1.0 | -/- | -/- | 81/0.95 | 100/1.0 |
| Arc40 | 100/1.0 | -/- | 87/1.0 | 78/- | 99/1.0 | 100/1.0 | 82/0.99 | #/# | 79/- | -/- | -/- | 98/1.0 | -/- | #/# | #/# |
| Atp2 | 100/1.0 | -/- | -/- | 89/1.0 | -/- | 100/1.0 | 100/1.0 | -/- | 100/1.0 | -/- | -/- | 99/1.0 | 94/- | 100/1.0 | 93/0.99 |
| Atp3 | 88/0.95 | -/- | -/- | 100/1.0 | 73/0.98 | 100/1.0 | 100/1.0 | -/- | 94/- | -/- | -/- | 84/- | -/- | 89/0.97 | #/# |
| Cct2 | 100/1.0 | #/# | -/- | 100/1.0 | 88/0.99 | 99/1.0 | 98/1.0 | 100/1.0 | 100/1.0 | -/- | -/- | -/- | 100/1.0 | 98/1.0 | 100/1.0 |
| Cct3 | 100/1.0 | -/- | -/- | -/- | -/- | 99/1.0 | 90/0.99 | -/- | 100/1.0 | -/- | 86/0.98 | 83/- | -/- | 74/0.96 | #/# |
| Cct5 | #/# | -/- | -/- | -/- | -/- | 89/0.95 | #/# | #/# | 70/- | #/# | #/# | 95/0.95 | 75/0.95 | -/- | 100/1.0 |
| Elp3 | 100/1.0 | -/- | -/- | 89/- | -/- | 100/1.0 | 100/1.0 | -/- | 96/1.0 | -/- | -/- | 84/0.95 | -/- | 95/1.0 | 100/1.0 |
| FG850 | 100/1.0 | -/- | -/- | 94/0.99 | -/- | 99/1.0 | 100/0.99 | -/- | 89/- | #/# | -/- | -/- | 79/- | 99/1.0 | 80/0.97 |
| Frs2 | 100/1.0 | -/- | -/- | 100/1.0 | -/- | 98/1.0 | 100/1.0 | #/# | 82/0.95 | -/- | -/- | 99/1.0 | -/- | 99/1.0 | 100/1.0 |
| Gdi1 | 78/- | 97/1.0 | -/- | -/- | 98/0.98 | 100/1.0 | 100/1.0 | 76/0.97 | 77/0.95 | 93/0.96 | -/- | -/- | #/# | -/- | -/- |
| Get3 | 100/1.0 | -/- | -/- | -/- | 90/0.95 | 97/0.95 | 88/0.95 | #/# | 96/0.99 | #/# | #/# | 96/0.99 | 82/0.95 | 87/0.95 | -/- |
| Gsh1 | 100/1.0 | 77/0.95 | 100/1.0 | 97/1.0 | -/- | 100/1.0 | -/- | 89/0.95 | 100/1.0 | 100/1.0 | 96/0.95 | -/- | -/- | #/# | 95/0.95 |
| Gus1 | 100/1.0 | -/- | -/- | 80/0.95 | -/- | 100/1.0 | 100/1.0 | 90/0.95 | 98/0.95 | -/- | -/- | -/- | -/- | -/- | 74/0.95 |
| Hem15 | 100/1.0 | -/- | -/- | 99/1.0 | -/- | 100/1.0 | #/# | 87/0.95 | 100/1.0 | 70/- | 96/1.0 | -/- | -/- | -/- | 88/0.95 |
| Hom3 | 100/1.0 | -/- | -/- | 100/1.0 | 91/- | 98/1.0 | 100/1.0 | 95/0.95 | 79/0.97 | #/# | #/# | 95/0.97 | -/- | -/- | #/# |
| Hsp60 | 100/1.0 | 89/- | 92/1.0 | #/# | -/- | 100/1.0 | 99/1.0 | 87/- | -/- | -/- | -/- | 87/- | 90/1.0 | 100/1.0 | 92/0.95 |
| Ils1 | 97/0.99 | -/- | -/- | #/# | 98/1.0 | 99/1.0 | 100/1.0 | #/# | 100/1.0 | -/- | -/- | -/- | -/- | 100/1.0 | 97/1.0 |
| Ilv2 | 100/1.0 | 87/0.96 | -/- | 98/- | -/- | 96/1.0 | 99/1.0 | #/# | -/- | -/- | 85/- | 95/1.0 | -/- | -/- | 85/1.0 |
| Krr1 | 100/1.0 | -/- | -/- | 88/0.95 | -/- | 100/1.0 | 100/1.0 | -/- | -/- | 86/0.95 | -/- | -/- | -/- | #/# | 96/0.99 |
| Mcm2 | 100/1.0 | -/- | -/- | 94/1.0 | -/- | 98/1.0 | 100/1.0 | 71/0.98 | 100/1.0 | -/- | -/- | -/- | -/- | 96/1.0 | #/# |
| Met6 | 100/1.0 | -/- | -/- | 91/0.98 | 96/- | 99/1.0 | 100/1.0 | -/- | 89/0.95 | -/- | 71/0.99 | -/- | -/- | 95/1.0 | -/- |
| MS417 | 98/1.0 | -/- | -/- | 99/1.0 | 94/0.95 | #/# | #/# | #/# | 100/1.0 | #/# | #/# | -/- | 99/1.0 | -/- | 87/0.95 |
| MS429 | 85/- | -/- | -/- | 83/0.99 | -/- | 90/- | 94/1.0 | -/- | -/- | -/- | -/- | -/- | -/- | 71/- | 88/0.95 |
| MS442 | 100/1.0 | 88/1.0 | 71/0.98 | #/# | -/- | #/# | 100/1.0 | #/# | 84/- | #/# | #/# | -/- | 76/- | #/# | #/# |
| MS456 | 99/1.0 | #/# | -/- | 94/0.95 | -/- | 100/1.0 | 100/1.0 | 91/0.95 | -/- | -/- | -/- | -/- | #/# | 100/1.0 | #/# |
| Pdb1 | 98/1.0 | -/- | -/- | #/# | 94/1.0 | 100/1.0 | 100/1.0 | #/# | 99/1.0 | -/- | -/- | -/- | -/- | #/# | 81/- |
| Pol30 | 96/0.95 | -/- | -/- | -/- | -/- | 96/0.95 | 100/1.0 | #/# | 96/0.95 | -/- | -/- | -/- | -/- | -/- | -/- |
| Qns1 | 100/1.0 | -/- | -/- | -/- | -/- | 100/1.0 | 98/1.0 | #/# | 100/1.0 | -/- | -/- | -/- | #/# | 99/1.0 | -/- |
| Rfc2 | 100/1.0 | -/- | -/- | -/- | 99/1.0 | #/# | 100/1.0 | -/- | 82/0.98 | 86/0.95 | 100/1.0 | 100/0.99 | -/- | 81/0.99 | 73/0.99 |
| Rfc4 | 100/1.0 | -/- | -/- | 100/1.0 | 99/1.0 | 98/0.99 | 100/1.0 | 73/- | 96/0.99 | #/# | 99/1.0 | -/- | -/- | #/# | 99/1.0 |
| Rio2 | 100/1.0 | -/- | -/- | #/# | -/- | 94/0.95 | 100/1.0 | -/- | #/# | -/- | -/- | -/- | -/- | 98/1.0 | -/- |
| Rpa135 | 99/1.0 | 91/0.95 | -/- | 97/1.0 | 86/0.95 | #/# | 100/1.0 | -/- | 96/0.95 | -/- | -/- | -/- | -/- | #/# | 95/0.95 |
| Sac6 | 99/1.0 | -/- | -/- | 100/1.0 | -/- | 97/0.95 | -/- | -/- | 94/0.95 | #/# | #/# | -/- | -/- | 79/0.95 | 98/0.95 |
| Stt3 | 100/1.0 | -/- | -/- | 100/1.0 | -/- | #/# | 98/0.98 | #/# | 82/- | #/# | 99/0.98 | -/- | #/# | #/# | #/# |
| Trp2 | 94/0.95 | 89/0.95 | 71/- | 88/0.95 | -/- | 99/1.0 | 98/1.0 | -/- | 97/0.99 | -/- | -/- | -/- | -/- | 97/1.0 | 96/0.95 |
| Uba1 | 87/0.95 | 78/0.95 | -/- | 71/- | -/- | 99/1.0 | 100/1.0 | -/- | #/# | -/- | -/- | 99/1.0 | 95/0.95 | #/# | 99/1.0 |
| Uba3 | 95/0.95 | 99/1.0 | -/- | 100/1.0 | -/- | 100/1.0 | 86/0.95 | -/- | #/# | #/# | -/- | 99/1.0 | -/- | #/# | 92/0.95 |
| Ygr207 | 97/1.0 | -/- | 94/1.0 | 95/1.0 | 92/0.99 | 100/1.0 | 100/1.0 | -/- | -/- | #/# | #/# | -/- | 95/1.0 | 87/1.0 | 99/1.0 |
| Yhm2 | 98/0.99 | #/# | 92/0.95 | #/# | 98/1.0 | 100/1.0 | 100/1.0 | #/# | 100/1.0 | #/# | #/# | -/- | 71/- | #/# | 76/- |
| Combined  dataset | 100/1.0 | 100/1.0 | 100/1.0 | 100/1.0 | 100/1.0 | 100/1.0 | 100/1.0 | 100/1.0 | 100/1.0 | 70/1.0 | 100/1.0 | 100/1.0 | 100/1.0 | 100/1.0 | 100/1.0 |
| Criteria satisfied | ① | ② | ② | ② | ② | ① | ① | ② | ② | ② | ② | ② | ② | ② | ② |
| Phylogenetic species | *P. abieticola* | *P. eryngii*  var. *eryngii* | *P. eryngii* var. *ferulae* | *P. nebrodensis* | *P. ostreatus* | *P. placentodes* | *P. populinus* | *P. pulmonarius* | *P. tuoliensis* | *Pleurotus* sp. 1 | *Pleurotus* sp. 2 | *Pleurotus* sp. 3 | *Pleurotus* sp. 5 | *Pleurotus* sp. 6 | *Pleurotus* sp. 7 |

Support values are shown as MLB/BPP. - and # represent phylogenetic species with low support (MLB < 70 %, BPP < 0.95) and lacking counterpart sequences respectively. Support values are not acquired for the following five potential phylogenetic species represented by single collection, which are therefore not included in the table: *P. albidus*, *P. eryngii* var. *elaeoselini*, *P. ferulaginis*, *P. fossulatus*, *Pleurotus* sp. 4.
